# Impaired Genome Maintenance Suppresses the Growth Hormone–Insulin-Like Growth Factor 1 Axis in Mice with Cockayne Syndrome

**DOI:** 10.1371/journal.pbio.0050002

**Published:** 2006-12-12

**Authors:** Ingrid van der Pluijm, George A Garinis, Renata M. C Brandt, Theo G. M. F Gorgels, Susan W Wijnhoven, Karin E. M Diderich, Jan de Wit, James R Mitchell, Conny van Oostrom, Rudolf Beems, Laura J Niedernhofer, Susana Velasco, Errol C Friedberg, Kiyoji Tanaka, Harry van Steeg, Jan H. J Hoeijmakers, Gijsbertus T. J van der Horst

**Affiliations:** 1 Department of Genetics, Center for Biomedical Genetics, Erasmus University Medical Center, Rotterdam, The Netherlands; 2 National Institute of Public Health and the Environment (RIVM), Laboratory of Toxicology, Pathology and Genetics (TOX), Bilthoven, The Netherlands; 3 Laboratory of Molecular Pathology, Department of Pathology, University of Texas Southwestern Medical Center, Dallas, Texas, United States of America; 4 Division of Cellular Genetics, Institute for Molecular and Cellular Biology, Osaka University, Osaka, Japan; Lawrence Berkeley National Lab, United States of America

## Abstract

Cockayne syndrome (CS) is a photosensitive, DNA repair disorder associated with progeria that is caused by a defect in the transcription-coupled repair subpathway of nucleotide excision repair (NER). Here, complete inactivation of NER in *Csb^m/m^*/*Xpa^−/−^* mutants causes a phenotype that reliably mimics the human progeroid CS syndrome. Newborn *Csb^m/m^/Xpa^−/−^* mice display attenuated growth, progressive neurological dysfunction, retinal degeneration, cachexia, kyphosis, and die before weaning. Mouse liver transcriptome analysis and several physiological endpoints revealed systemic suppression of the growth hormone/insulin-like growth factor 1 (GH/IGF1) somatotroph axis and oxidative metabolism, increased antioxidant responses, and hypoglycemia together with hepatic glycogen and fat accumulation. Broad genome-wide parallels between *Csb^m/m^/Xpa^−/−^* and naturally aged mouse liver transcriptomes suggested that these changes are intrinsic to natural ageing and the DNA repair–deficient mice. Importantly, wild-type mice exposed to a low dose of chronic genotoxic stress recapitulated this response, thereby pointing to a novel link between genome instability and the age-related decline of the somatotroph axis.

## Abstract 2

Studies in mice defective in two DNA repair pathways (global NER and TCR; an animal model for Cockayne syndrome) highlight a link between aging, a failure to repair DNA lesions, and metabolic alterations.

## Author Summary

Normal metabolism routinely produces reactive oxygen species that damage DNA and other cellular components and is thought to contribute to the ageing process. Although DNA damage is typically kept in check by a variety of enzymes, several premature ageing disorders result from failure to remove damage from active genes. Patients with Cockayne syndrome (CS), a genetic mutation affecting one class of DNA repair enzymes, display severe growth retardation, neurological symptoms, and signs of premature ageing followed by an early death. Whereas mouse models for CS exhibit relatively mild deficits, we show that concomitant inactivation of a second DNA repair gene elicits severe CS pathology and ageing. Moreover, a few days after birth, these mice undergo systemic suppression of genes controlling growth, an unexpected decrease in oxidative metabolism, and an increased antioxidant response. Similar physiological changes are also triggered in normal mice by chronic exposure to DNA-damaging oxidative stress. From these findings, we conclude that DNA damage triggers a response aimed at limiting oxidative DNA damage levels (and associated tissue degeneration) to extend lifespan and promote healthy ageing. Better understanding of the ageing process will help to delineate intervention strategies to combat age-associated pathology.

## Introduction

A prevailing hypothesis to explain the molecular basis of ageing is Harman's “free-radical theory of ageing”, which states that endogenous reactive oxygen species (ROS), which result from cellular metabolism, continually damage biomolecules [1]. In line with this hypothesis, it has been shown that increased resistance to oxidative stress (e.g., by improved antioxidant defense) extends the lifespan of *Caenorhabditis elegans, Drosophila,* and rodents [2–4], whereas hypersensitivity to oxygen considerably reduces the lifespan of nematodes [5]. A key macromolecule at risk for ROS-mediated damage is nuclear DNA [1], which is evident from the wide range of oxidative DNA lesions that accumulate gradually in rodents and humans with advancing age [6,7].

In humans, the causative role of DNA damage in ageing is supported by a variety of progeroid disorders with defects in DNA repair pathways [8,9]. One such condition is Cockayne syndrome (CS) (affected genes: *CSA* or *CSB*), a photosensitive disorder that originates from a defect in transcription-coupled repair (TCR), which specifically removes DNA lesions that obstruct RNA polymerases, allowing resumption of transcription and promoting cellular survival from DNA damage. TCR of helix-distorting DNA damage is a dedicated subpathway of the multi-step “cut-and-patch” nucleotide excision repair (NER) system, and is designated TC-NER [10] to distinguish it from the so-called global genome NER (GG-NER) subpathway that operates genome-wide to eliminate distorting damage. Available evidence suggests that CS cells are also defective in TCR of non–helix distorting DNA lesions that block transcription such as transcription-blocking oxidative DNA lesions [11,12], which are normally genome-wide removed by base excision repair. We will use the term TCR when referring to transcription-coupled repair in general.

CS patients present with growth failure (cachectic dwarfism), progressive neurological abnormalities (including delayed psychomotor development, mental retardation, microcephaly, gait ataxia, sensorineural hearing loss, retinal degeneration), along with impaired sexual development, kyphosis, osteoporosis, and severely reduced lifespan (mean age of death: 12.5 y) [13,14]. A related yet distinct disorder is trichothiodystrophy (TTD) (affected genes: *XPB, XPD,* or *TTDA*). TTD patients are partially defective in TCR as well as in the GG-NER, and share the symptoms associated with CS. In addition, these patients have a partial defect in transcription itself, causing additional symptoms such as ichthyosis and brittle hair and nails [15]. Many of the CS and TTD features are progressive and resemble premature ageing. Because patients develop some but not all aspects of normal ageing in an accelerated manner, CS and TTD are considered “segmental progeroid syndromes” [8].

Mouse mutants for CS-A and CS-B reliably mimic the UV sensitivity of CS patients and show accelerated photoreceptor loss, reduced body weight, and mild neurologic abnormalities [16,17]. Similarly, mice homozygous for a causative TTD point mutation in the *Xpd* gene faithfully mirror the symptoms in TTD patients [9], whereas complete inactivation of NER (by concurrent inactivation of the *Xpa* gene) dramatically aggravates the CS features of partially NER-defective TTD mice [9]. These observations, together with the notion that DNA lesions can provoke a permanent cell cycle arrest or apoptosis, led us to propose that ageing can result from (oxidative) DNA lesions that interfere with transcription and/or replication causing cell death or cellular senescence, ultimately leading to the loss of tissue homeostasis and the onset of age-related diseases [18–20].

Here we report that mice with engineered mutations in both *Csb* and *Xpa* genes display many CS features in a dramatic form, including postnatal growth attenuation, progressive kyphosis, ataxia, retinal degeneration, motor dysfunction, and premature death. Importantly, full genome transcriptome analysis of the *Csb^m/m^/Xpa^−/−^* mouse liver at the age of 15 d uncovered a systemic response seen also in wild-type (wt) mice exposed to chronic oxidative stress. These findings disclose a novel link between DNA damage, compromised genome maintenance, and the somatotrophic axis that determines lifespan and shed new light on the etiology of Cockayne syndrome and natural ageing.

## Results

### Attenuated Growth and Perinatal Death in *Csb^m/m^/Xpa^−/−^* and *Csb^m/m^/Xpc^−/−^* Mice

**Figure 1:**
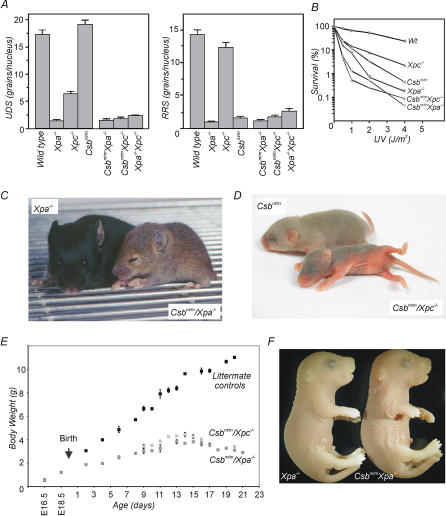
Growth Retardation, Cachexia, and Premature Death in *Csb^m/m^/Xpa^−/−^* and *Csb^m/m^/Xpc^−/−^* Mice (A) UV repair characteristics of wt, single-mutant, and double-mutant primary MEFs. UV-induced UDS (left panel) and recovery of RNA Synthesis (RRS, right panel) are indicative for GG-NER and TC-NER capacity, respectively. For a detailed explanation of the procedure, see Methods. Error bars indicate standard error of the mean (SEM). (B) Survival of primary MEFs exposed to increasing doses of UV-C light (254 nm), as determined using the with [^3^H]-thymidine incorporation assay. Error bars (in most cases smaller than symbols used) indicate SEM. (C) Photograph of a 14-d-old *Csb^m/m^/Xpa^−/−^* mouse with an *Xpa^−/−^* littermate (hybrid 129Ola/C57BL/6J background). (D) Photograph of an 8-d-old *Csb^m/m^/Xpc^−/−^* mouse with a *Csb^m/m^* littermate (hybrid 129Ola/C57BL/6J background). (E) Body weight curve of *Csb^m/m^/Xpa^−/−^* and *Csb^m/m^/Xpc^−/−^* mice (*n* = 7) compared to those defective in a single NER gene (*n* = 7) all in a hybrid 129Ola/C57BL/6J background. The arrow indicates birth. Error bars (in most cases smaller than symbols used) indicate SEM. (F) Photographs of day 18.5 *Csb^m/m^/Xpa^−/−^* and *Xpa^−/−^* embryos (C57BL/6J).

TCR-defective *Csb^m/m^* mutant mice [16] were intercrossed with GG-NER-defective *Xpc^−/−^* [21] and GG/TC-NER-defective *Xpa^−/−^* [22] animals to investigate whether an increase in the endogenous burden of unrepaired DNA damage, as provoked by the inactivation of GG-NER, enhances the phenotype, including progeroid features. Analysis of UV-induced repair synthesis and RNA synthesis recovery (indicative for GG-NER and TC-NER capacity, respectively) confirmed complete inactivation of NER in *Csb^m/m^/Xpa^−/−^* and *Csb^m/m^/Xpc^−/−^* animals (Figure 1A). As expected on the basis of previous work, *Xpa^−/−^* cells display the highest UV sensitivity, whereas *Xpc^−/−^* and *Csb^m/m^* cells show intermediate sensitivities (*Xpa^−/−^* > *Csb^m/m^* > *Xpc^−/−^* > wt; see Figure 1B). Interestingly, inactivation of GG-NER in *Csb^m/m^* mouse embryonic fibroblasts (MEFs) (as in *Csb^m/m^/Xpa^−/−^* and *Csb^m/m^/Xpc^−/−^* cells) renders cells more UV-sensitive than already completely NER-deficient *Xpa^−/−^* MEFs. We attribute this enhanced sensitivity to the absence of CSB-mediated TCR of UV-induced lesions that do not form a substrate for NER. Thus, the repair defect in the double mutant appears to be more severe than that of the single mutants. We could not detect a similar increased sensitivity to ionizing radiation in double-mutant cells above that of *Csb^m/m^* cells [12] (unpublished data), supporting the notion that MEFs in culture are already under high oxygen stress [23,24].

As evident from their overall appearance and weight (Figure 1C–1E), *Csb^m/m^/Xpa^−/−^* and *Csb^m/m^/Xpc^−/−^* pups (hybrid C57BL/6Jx129ola genetic background) displayed a strikingly attenuated growth, resulting in pronounced dwarfism. Whereas the number of double mutant pups was ∼3-fold below that expected for Mendelian inheritance (Table S1), embryonic day 18/5 (E18.5) *Csb^m/m^/Xpa^−/−^* and *Csb^m/m^/Xpc^−/−^* embryos were present at Mendelian frequency, pointing to considerable lethality during or shortly after birth. Importantly, double-mutant embryos were morphologically and histologically indistinguishable from wt and single-mutant embryos (Figure 1F and unpublished data), indicating that the growth defect was postnatal and did not reflect impaired embryonic development per se. In the third week of life, however, *Csb^m/m^/Xpa^−/−^* and *Csb^m/m^/Xpc^−/−^* pups developed progressive cachexia (evident from the weight loss after day 15; see Figure 1E), ultimately resulting in death before postnatal day 22. Neither moistening of food pellets (to facilitate intake of solids) nor removal of wt or single-mutant pups from the litter (to reduce competition for breast milk) improved the physical condition or the lifespan of *Csb^m/m^/Xpa^−/−^* and *Csb^m/m^/Xpc^−/−^* pups. Necropsy revealed milk or solid food in the stomach, indicating that insufficient access to supplied nutrition was not the underlying cause of growth retardation, weight loss, and early death. Importantly, progressive growth retardation, cachexia, and short life expectation (∼12.5 y) are also observed in human patients with CS [13]. Combined inactivation of *Xpa* and *Xpc* rendered mice without any overt phenotype (unpublished data), leading us to conclude that the dramatic phenotype of *Csb^m/m^/Xpa^−/−^* and *Csb^m/m^/Xpc^−/−^* pups results from a combined GG-NER/TC-NER/TCR defect.

### Growth and Neurological Abnormalities in *Csb^m/m^/Xpa^−/−^* Mice

**Figure 2:**
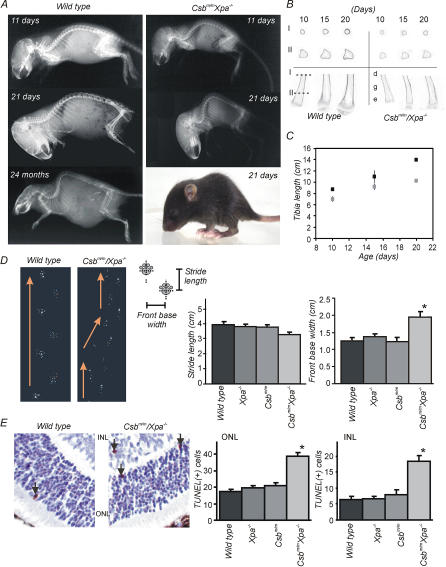
Skeletal and Neurological Abnormalities in *Csb^m/m^/Xpa^−/−^* Mice (A) Radiographs of wt and *Csb^m/m^/Xpa^−/−^* mice (age as indicated) and photograph of a 15-d-old *Csb^m/m^/Xpa^−/−^* mouse (C57BL/6J). (B) 2-D images of micro-CT scans of tibiae taken from *Csb^m/m^/Xpa^−/−^* animals and wt littermate controls (age indicated in the figure). Horizontal sections are shown of the upper and lower part of the tibiae. e, epiphysis; g, growth plate; and d, diaphysis. I indicates the section through the smaller part of the diaphysis, II indicates section through the broader part of the diaphysis. (C) Growth of tibiae, taken from *Csb^m/m^/Xpa^−/−^* animals (light squares) and wt littermate controls (dark squares). (D) Representative footprint patterns of 19-d-old wt and *Csb^m/m^/Xpa^−/−^* mice. Arrows indicate the trajectory of each mouse. Stride length and front base width measurements on 15-d-old wt, *Xpa^−/−^, Csb^m/m^,* and *Csb^m/m^/Xpa^−/−^* mice. The significantly (asterisk; *p* < 0.001) greater base width in the double mutant mouse indicates ataxia. (E) Representative pictures of a TUNEL staining in the retina of wt and *Csb^m/m^/Xpa^−/−^* mice and quantification of the number of TUNEL positive cells. Arrows indicate TUNEL positive cells in the ONL and INL. Note the significantly higher number of TUNEL-positive cells in both the ONL and the INL in the retina of *Csb^m/m^/Xpa^−/−^* compared to wt mice (asterisk; *p* < 0.05).

Further analysis of the *Csb^m/m^/Xpa^−/−^* phenotype, performed in an isogenic C57BL/6J background, revealed a near-normal size of the skull at day 11 and 21 (autoradiographs shown in Figure 2A), implying that the (postnatal) growth defect is restricted to the trunk, and to a lesser extent, the extremities. All 21-d-old double-mutant animals showed kyphosis (abnormal curvature of the spinal column, Figure 2A, middle left and bottom right), which was also observed in younger *Csb^m/m^/Xpa^−/−^* pups, indicating that it is not determined by terminal illness. The normal appearance of the spine in 11-d-old double-mutant pups excluded a prenatal developmental defect and further pointed to an extremely accelerated onset of kyphosis, a feature observed in naturally aged (2-y old) C57BL/6J mice (see Figure 2A, bottom left panel). Two-dimensional images of proximal end-to-mid-diaphysis micro–computed tomography (micro-CT) scans of fixed tibiae from 10-, 15-, and 20-d-old wt and *Csb^m/m^/Xpa^−/−^* mice revealed retarded, yet steady, longitudinal as well as radial (perimeter) growth, along with a thinner bone cortex and a less developed growth plate (Figure 2B). In line with this observation, we observed a reduction in tibia length (Figure 2C). Notably, whereas *Csb^m/m^/Xpa^−/−^* pups lose weight in the third week of life, bone growth proceeds, resulting in relatively large extremities, a representative feature of CS and TTD [13].

Motor coordination problems, manifesting as tremors and abnormal posture of the hind limbs (flexion rather than extension in tail suspension test), became evident around day 10 in *Csb^m/m^/Xpa^−/−^* mice (unpublished data). Foot print analysis revealed a disturbed gait from day 15 onwards. Whereas wt and single-mutant animals maintained a straight path with regular alternating strides, *Csb^m/m^/Xpa^−/−^* mice demonstrated a nonuniform alternating left-right step pattern and unevenly spaced shorter strides (Figure 2D). Despite their runted size, the front base width of *Csb^m/m^/Xpa^−/−^* animals was significantly greater than that of wt and single-mutant littermates, which likely illustrates an attempt to maintain balance (Figure 2D). These data are consistent with the profound early postnatal ataxia and abnormal cerebellar development in *Csb^m/m^/Xpa^−/−^* mice [25] and the progressive neurodegeneration observed in human CS patients [26].

We next examined the retinas of 15-d-old *Csb^m/m^/Xpa^−/−^* pups for the presence of apoptotic cells, because retinal degeneration is a prominent neurological feature of CS patients [27] and adult CS mice (T. Gorgels, I. van der Pluijm, R. Brandt, G. Garinis, H. van Steeg, et al., unpublished data). At this age, cell loss occurs as part of the normal development of the retina. Yet, as shown by terminal deoxynucleotidyltransferase-mediated dUTP nick end labeling (TUNEL) (Figure 2E) and caspase-3 staining (unpublished data), the number of apoptotic cells in the outer nuclear layer (ONL) and inner nuclear layer (INL) of the retinas of *Csb^m/m^/Xpa^−/−^* pups was significantly increased (analysis of variance [ANOVA], S-N-K posthoc test, *p* < 0.05), as compared to wt and single-mutant littermates (Figure 2E). Thus, the *Xpa* defect enhanced the apoptotic sensitivity of photoreceptor cells in *Csb^m/m^* mice, thereby pointing to DNA damage as a trigger for age-related retinal degeneration. Because 15-d-old *Csb^m/m^* mice still have wt levels of apoptotic cells, spontaneous photoreceptor loss in the *Csb^m/m^* mouse initiates in the second/third month of life.

**Figure 3:**
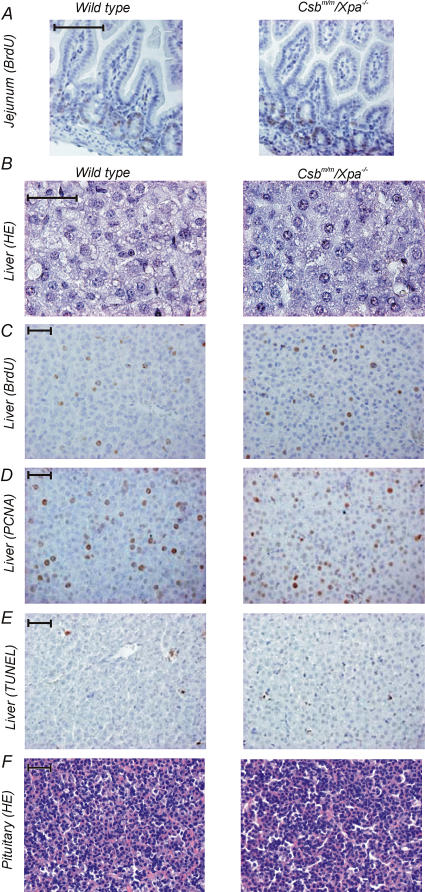
Histological Examination of *Csb^m/m^/Xpa^−/−^* Tissues (A) BrdU staining of the jejunum of *Csb^m/m^/Xpa^−/−^* and littermate control mice, showing normal proliferative capacity of the intestine in the double mutant mouse. (B–E) Histological examination of liver sections of 15-d-old *Csb^m/m^/Xpa^−/−^* and littermate control mice, stained with HE (B), immunostained for the proliferation markers (incorporated) BrdU (C) and PCNA protein (D), or TUNEL-stained for the presence of apoptotic cells (E). Quantification of the number of proliferative or apoptotic cells did not reveal significant differences between *Csb^m/m^/Xpa^−/−^* and wt littermate mice. (F) HE staining of the pituitary of *Csb^m/m^/Xpa^−/−^* and littermate control mice.

Visual inspection and histological analysis of most internal organs of 15-d-old *Csb^m/m^/Xpa^−/−^* mice did not reveal any obvious pathological abnormalities (unpublished data), with the exception of substantial loss of abdominal fat. Because we did not find any sign of infections, necrosis, or abnormal cellular proliferation (as determined by bromodeoxyuridine [BrdU] staining) in the gastrointestinal tract of 15- and 21-d-old *Csb^m/m^/Xpa^−/−^* animals, intestinal malfunction is an unlikely cause of the growth defect (Figure 3A). In addition, the liver had a normal histological appearance (Figure 3B), whereas neither BrdU (Figure 3C), proliferating cell nuclear antigen (PCNA) (Figure 3D), and Ki67 staining (unpublished data), nor TUNEL (Figure 3E) and caspase-3 staining (unpublished data) revealed any significant difference between *Csb^m/m^/Xpa^−/−^* and wt livers. This finding indicates that aberrant cell proliferation or apoptosis in the liver does not likely contribute to the *Csb^m/m^/Xpa^−/−^* phenotype. Moreover, inactivation of the *p53* tumor-suppressor gene failed to rescue the mutant phenotype, because *Csb^m/m^/Xpa^−/−^/p53^−/−^* triple-mutant pups appeared indistinguishable from *Csb^m/m^/Xpa^−/−^* pups (unpublished data). Thus, the precise etiology of the overall physical deterioration and the cause of death of *Csb^m/m^/Xpa^−/−^* mice remain unknown.

### Enhanced Ionizing Radiation Sensitivity of the *Csb^m/m^/Xpa^−/−^* Mouse Retina

**Figure 4:**
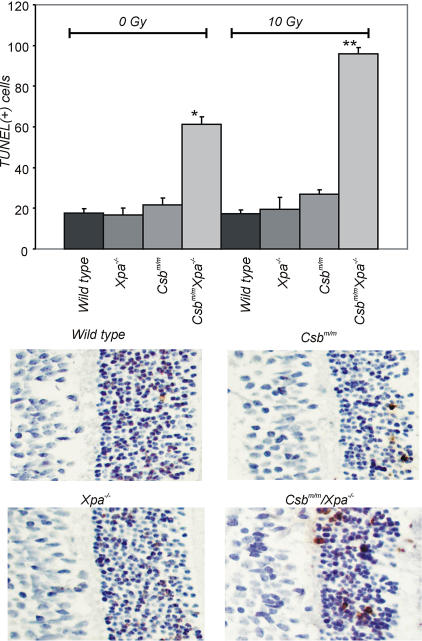
Enhanced Sensitivity of *Csb^m/m^/Xpa^−/−^* Retinal Photoreceptor Cells to Genotoxic Insults Representative pictures of TUNEL stained retinas of 19-d-old wt, *Csb^m/^* ^m^ *, Xpa^−/−^,* and *Csb^m/m^/Xpa^−/−^* mice (right panel), 20 h after exposure of animals to 10 Gy of ionizing radiation, and quantification of the number of TUNEL positive cells in the ONL (left panel). Note the significantly higher number of TUNEL-positive cells in the retina of *Csb^m/m^/Xpa^−/−^* mice, as compared to wt and single-mutant littermate controls (*p* < 0.05). Single asterisks indicate statistically significant differences between unirradiated *Csb^m/m^/Xpa^−/−^* and littermate control mice (*p* < 0.05), double asterisks indicate statistically significant differences between unirradiated and irradiated *Csb^m/m^/Xpa^−/−^* mice (*p* < 0.05).

The spontaneous, age-related and ionizing radiation (IR)–induced loss of post-mitotic photoreceptor cells in *Csb^m/m^* mice underscores the relevance of DNA repair in the removal of (oxidative) DNA damage for the long-term survival of terminally differentiated cells in the retina (T. Gorgels, I. van der Pluijm, R. Brandt, G. Garinis, H. van Steeg, et al., unpublished data). To test whether *Csb^m/m^/Xpa^−/−^* animals are more sensitive to genotoxic insults than are single-mutant *Csb^m/m^* and *Xpa^−/−^* animals, we next examined if the additional *Xpa* defect further enhances the IR sensitivity of the *Csb^m/m^* retina. To this end, we exposed 18-d-old *Csb^m/m^/Xpa^−/−^* pups and wt and single-mutant littermates to γ rays (10 Gy) and quantified the number of apoptotic cells in the retina by TUNEL staining 20 h after exposure. As shown in Figure 4, the number of apoptotic cells in the ONL of untreated (19-d-old) *Csb^m/m^/Xpa^−/−^* pups further increased, as compared to 15-d-old double-mutant animals (see Figure 2E). Whereas IR exposure did not increase the frequency of apoptotic photoreceptors in the ONL of wt and *Xpa^−/−^* animals, *Csb^m/m^* mice already show a tendency to increased photoreceptor loss, as characteristic for mature *Csb^m/m^* animals (T. Gorgels, I. van der Pluijm, R. Brandt, G. Garinis, H. van Steeg, et al., unpublished data). In contrast, the retinas of IR-exposed *Csb^m/m^/Xpa^−/−^* animals showed an almost 2-fold increase in the level of TUNEL-positive photoreceptor cells (Student's *t*-test; *p* = 0.021). Taken together, these findings not only further point to unrepaired DNA damage (likely originating from oxidative stress) as the underlying trigger for photoreceptor loss, but importantly, also show that inactivation of *Xpa* further enhances the sensitivity of *Csb^m/m^* mice to genotoxic stress.

### Analysis of the *Csb^m/m^/Xpa^−/−^* Mouse Liver Transcriptome

To investigate whether a disturbance in growth and metabolism could explain the pronounced accelerated organismal deterioration seen in *Csb^m/m^/Xpa^−/−^* mice, we evaluated the liver transcriptome of 15-d-old wt, single-mutant, and double-mutant mice (*n* = 4 mice). At this age, the *Csb^m/m^/Xpa^−/−^* pups have not yet become cachectic. Two-tailed *t* test analysis Affymetrix full mouse genome arrays revealed 1,865 genes with significantly changed expression patterns between wt and *Csb^m/m^/Xpa^−/−^* livers (*p* **≤** 0.01, 1.2-fold change up- or down-regulated, Table S2), a number that significantly exceeds the ∼80 genes that are expected to occur by chance under these selection criteria. Among the set of 1,865 genes, we identified those gene ontology (GO)–classified biological processes with a significantly disproportionate number of responsive genes relative to those printed on microarrays (false detection rate ≤ 0.10). This unbiased approach revealed processes implicated in the derivation of energy from oxidation of organic compounds, homeostasis of energy reserves, cell growth and maintenance, and the redox status of the cell.

Subsequent analysis of these processes led us to identify the following four results.

(1) A profound attenuation of the somatotroph axis, as evidenced by the consistent down-regulation of genes encoding main components of the GH/IGF1 axis (e.g., *IGF1*, *Igfbp3, Igfbp4, Igfals, Ghr*), as well as lactotroph (e.g., *Prlr*) and thyrotroph functions (e.g., *Dio1*) in *Csb^m/m^/Xpa^−/−^* livers, in addition to a decrease in the expression of several genes associated with a variety of mitogenic signals (e.g., *Esr1, Fgf1, Fgfr3, Fgfr4*) (Table 1 and Table S2).

**Table 1:**
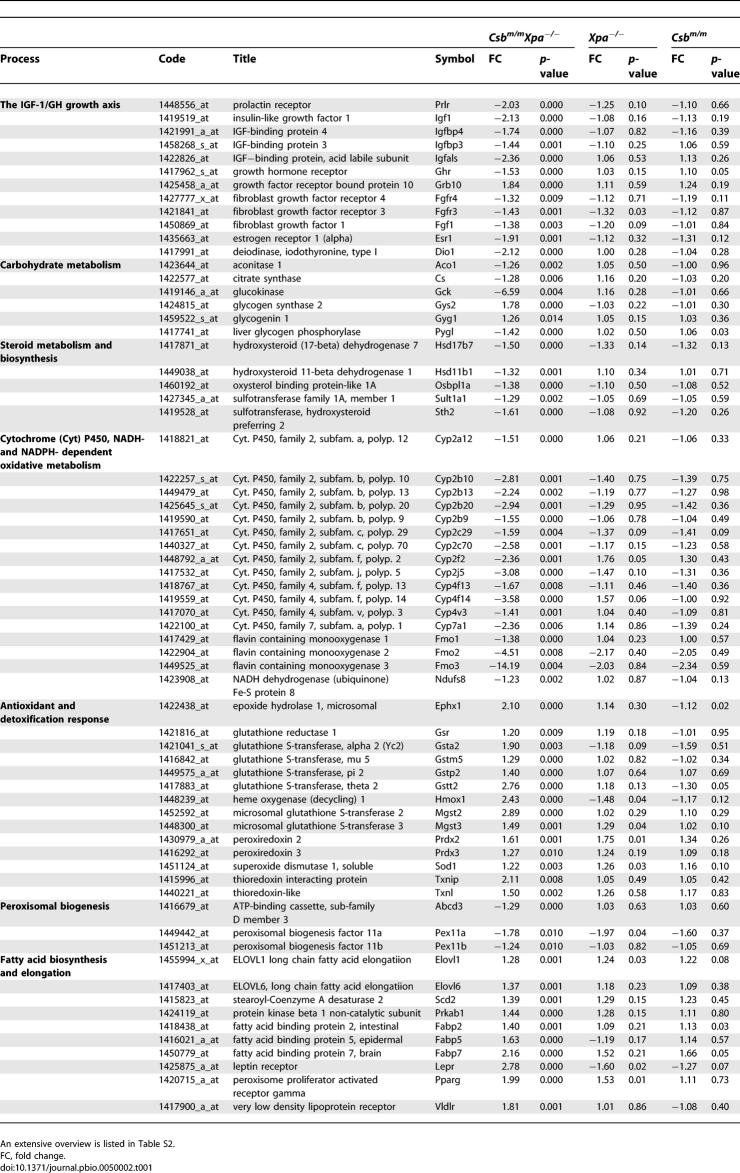
Significant Gene Expression Changes in the Livers of *Csb^m/m^Xpa^−/−^, Csb^m/m^,* and *Xpa^−/−^* Mice Compared to Livers from Littermate Controls

(2) An extensive suppression of catabolic metabolism in the *Csb^m/m^/Xpa^−/−^* liver, as evident from the significant down-regulation of key genes involved in glycolysis, the tricarboxylic acid cycle, and oxidative phosphorylation pathways (Table 1 and Table S2), coupled with a significant up-regulation of genes associated with glycogen synthesis (e.g., *Gyg1* and *Gys2*) and down-regulation of glycogen phosphorylase *(Pygl),* suggesting that the *Csb^m/m^/Xpa^−/−^* liver stores glucose into glycogen, rather than burn it for energy derivation. These changes were further accompanied by the broad down-regulation of genes associated with electron transport and oxidative phosphorylation (e.g., several cytochrome P450 monooxygenases, the NADH dehydrogenase complex, and the NADPH-dependent oxidative metabolism) (Table 1 and Table S2) and the significant down-regulation of several genes associated with peroxisomal biosynthesis (Table 1). Apparently, the complete catabolic metabolism is restrained in the *Csb^m/m^/Xpa^−/−^* liver.

(3) A broad up-regulation of genes associated with fatty acid synthesis and transport (several genes listed in Table 1 and Table S2), the up-regulation of the receptor for the adipocyte hormone leptin *(Lepr),* and the central fat regulator peroxisome proliferator-activated receptor-gamma *(Ppar*γ*)*. Thus, similar to their reserved glucose utilization and enhanced glycogen synthesis, *Csb^m/m^/Xpa^−/−^* mice attempt to store rather than burn fat.

(4) An up-regulation of genes encoding key enzymatic and nonenzymatic low–molecular mass scavengers and antioxidant defense enzymes (e.g., *Sod1, Prdx2* and *3, Txnip, Ephx1, Hmox1* and five components of the glutathione system) (Table 1), suggesting that *Csb^m/m^/Xpa^−/−^* mice try to minimize the induction of (DNA) damage by counteracting ROS.

**Figure 5:**
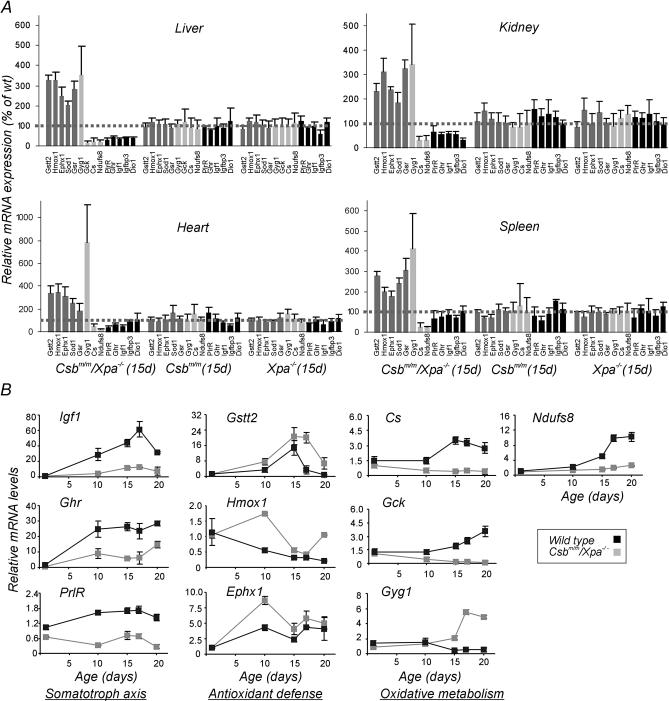
Expression Levels of Genes Associated with the Somatotroph Axis, Antioxidant Defense, and Metabolism in Mutant and wt Mouse Liver and Other Organs at Various Ages **(**A**)** Q-PCR evaluation of mRNA levels of genes associated with antioxidant defense (dark gray bars), oxidative metabolism (light gray bars), and the GH/IGF1 axis (black bars) in the liver, kidney, heart, and spleen of 15-d-old *Csb^m/m^/Xpa^−/−^, Csb^m/m^,* and *Xpa^−/−^* pups. For each gene, expression levels in the mutant tissue are plotted relative to that of age-matched wt control tissues (dotted line). Error bars indicate SEM between replicates (*n* ≥ 3). (B) Relative mRNA expression levels (fold changes, relative to embryonic day 18) of genes involved in the GH/IGF1 growth axis, antioxidant defense, and oxidative metabolism in the liver of wt and *Csb^m/m^/Xpa^−/−^* pups, plotted as a function of time. Error bars indicate SEM between replicates (*n* ≥ 3).

None of these genes were identified as significantly differentially expressed in the livers of *Csb^m/m^* or *Xpa^−/−^* littermate controls (Table 1). Quantitative real-time PCR (Q-PCR) evaluation of the expression levels of key genes involved in the somatotroph axis, energy metabolism, and antioxidant defense in the livers of *Csb^m/m^/Xpa^−/−^* mice, and wt*, Csb^m/m^*, and *Xpa^−/−^* littermates, as well as further biochemical analysis (see below), confirmed the validity of the microarray data (Figure 5A, upper left panel).

### Postnatal Systemic Changes in Somatotroph Axis, Energy Metabolism, and Antioxidant Defense in *Csb^m/m^/Xpa^−/−^* Mice

Next we analyzed whether the onset of the aforementioned transcriptional changes paralleled the progressive postnatal growth attenuation as well as the weight loss observed later. Consistent with the normal embryonic development, the expression levels of genes involved in the somatotroph axis *(Ghr, Igf1, Prlr)*, antioxidant defense *(Gstt2, Hmox1, Ephx1),* and oxidative metabolism *(Gck, Gyg1, Cs, Ndufs8)* did not differ significantly between wt and *Csb^m/m^/Xpa^−/−^* livers at postnatal day 1 (Figure 5B). In contrast, during the first 2 wk of life, wt mice exhibited, as expected, a robust up-regulation in *Igf1, Ghr,* and *Prlr* gene expression, a response that was virtually absent in *Csb^m/m^/Xpa^−/−^* animals (Figure 5B, left panels); this explains well the severe growth retardation of double-mutant pups after birth. Analysis of *Gstt2, Hmox1,* and *Ephx1* mRNA levels revealed that the up-regulation of the antioxidant defense system in the *Csb^m/m^/Xpa^−/−^* liver already initiated before postnatal day 10, and thus occurs well ahead of the initiation of the physiological decline (i.e., weight loss) (Figure 5B, middle panels). When comparing mRNA levels of key genes in glycolysis *(Gck),* tricarboxylic acid cycle *(Cs),* and mitochondrial oxidative phosphorylation *(Ndufs8),* we noticed that beginning postnatal day 10, *Csb^m/m^/Xpa^−/−^* livers do not show the prominent up-regulation of these catabolic genes seen in the wt liver (instead, expression levels continued to decline), whereas they up-regulate glycogen synthesis *(Gyg1)* (Figure 2B, right panels). In agreement, the enzymatic activity of citrate synthase was significantly lower (*p* ≤ 0.01) in the livers of 15-d-old *Csb^m/m^/Xpa^−/−^* mice (119 ± 15 mU/mg protein), as compared to wt littermate controls (70 ± 13 mU/mg protein).

We next determined the expression levels of aforementioned genes in the kidney, heart, and spleen of the same set of animals used in the microarray experiment. Expression levels markedly mirrored the deviant expression patterns observed in the liver, whereas mRNA levels in *Csb^m/m^* and *Xpa^−/−^* tissues were not significantly different from wt animals (Figure 5A). Thus, attenuation of the GH/IGF1 axis and down-regulation of metabolism, along with the enhanced antioxidant/detoxification response, represents a systemic rather than liver-specific response of the *Csb^m/m^/Xpa^−/−^* pups to the DNA repair defect. Interestingly, when 96-wk-old wt livers were tested for expression levels of this same set of *Csb^m/m^/Xpa^−/−^* responsive genes, we noticed a remarkable resemblance (Figure S1).

### Comparison of the *Csb^m/m^/Xpa^−/−^* and Naturally Aged Mouse Liver Transcriptomes

The previous result prompted us to investigate whether and to which extent the gene expression changes in the *Csb^m/m^/Xpa^−/−^* mouse liver overlap with those observed in a natural aged liver. To this end, we first compared the full mouse liver transcriptome of adult 16-, 96- and 130-wk-old wt C57Bl/6J mice (*n* = 4) with that of adult 8-wk-old wt C57Bl/6J mice (*n* = 4) (Tables S3–S5). Using the same analytical method as applied to the *Csb^m/m^/Xpa^−/−^* mouse livers, we identified homeostasis of energy reserves, oxidative metabolism, along with cell growth and maintenance to be significantly overrepresented in 96- and 130-wk-old wt mice, but not in 16-wk-old animals (Table S6). These findings fit well with previous studies, suggesting the repression of oxidative metabolism to represent a conserved response shared by highly diverged species [28].

**Figure 6:**
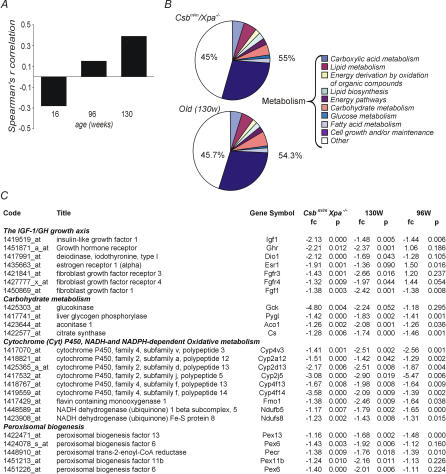
Transcriptome Similarities between *Csb^m/m^/Xpa^−/−^* and Naturally Aged Mice (A) Spearman's *r* correlation of 16-, 96- and 130-wk-old mice with 15-d-old *Csb^m/m^/Xpa^−/−^* mice, where −1.0 is a perfect negative (inverse) correlation, 0.0 is no correlation, and +1.0 is a perfect positive correlation. (B) Similarities between significantly overrepresented biological processes. Note that in both *Csb^m/m^/Xpa^−/−^* and naturally aged mice, transcriptional changes were mostly associated with metabolic processes. (C) Correlation in significant expression changes of genes associated with the GH/IGF1 axis and oxidative metabolism in the livers of *Csb^m/m^/Xpa^−/−^* and naturally aged (96- and 130-wk-old) mice. An extensive overview is listed in Tables S3 and S4.

Next we implemented a previously described method [29] to evaluate the extent of genome-wide similarity between the liver transcriptomes of 2-wk-old *Csb^m/m^/Xpa^−/−^*mice and wt animals of various ages. We first classified all significantly differentially expressed genes in the *Csb^m/m^/Xpa^−/−^* liver transcriptome as having increased or decreased expression (as compared to wt), and we asked how many of these genes respond in a similar direction in the 16/8 wk, 96/8 wk, and 130/8 wk datasets. If the *Csb^m/m^/Xpa^−/−^* liver resembles an aged liver, one expects the Spearman's rank correlation coefficient (*r*, +1.0 or −1.0 in case of perfect similarity or dissimilarity, respectively, and 0.0 in case of no correlation) to increase with age. Notably, whereas the liver transcriptome of *Csb^m/m^/Xpa^−/−^* mutant mice was dissimilar to that of 16-wk-old wt mice (Spearman's *r* = −0.28), as it was with 15-d-old littermates, this turned into a positive correlation when the comparison was made between the *Csb^m/m^/Xpa^−/−^* and 96-wk-old mouse liver transcriptomes (*r* = +0.15) and even more with the 130-wk-old wt mouse group (*r* = + 0.44, *p* **≤** 0.0001) (Figure 6A). Comparable results were obtained when the same approach was applied over the whole mouse transcriptome (including all Affymetrix probe sets with signals above the detection cutoff value; see Materials and Methods), thus avoiding any initial preselection or introduction of bias. Using the same approach, we did not find a significant correlation between the liver transcriptomes of 15-d-old *Csb^m/m^* or *Xpa^−/−^*mice and aged wt mice.

The genome-wide resemblance between the short-lived *Csb^m/m^/Xpa^−/−^* mice and the 130-wk-old mice was substantially higher (>90%) when the comparison was restricted to those functional categories that were significantly overrepresented in the double-mutant and 130-wk-old mice, such as the GH/IGF1 axis, oxidative metabolism (i.e., glycolysis, Krebs and oxidative phosphorylation), cytochrome P450 electron transport, and peroxisomal biogenesis (Figure 6B and 6C and Table S7). Despite the occurrence of dissimilarities between the liver transcriptome of *Csb^m/m^/Xpa^−/−^* pups and aged wt mice (the latter animals showing over-representation of genes involved in the immune and inflammatory responses, ATP biosynthesis, and protein glycosylation, along with a lack of the anti-oxidant response), these findings strongly underline the genome-wide parallels between the *Csb^m/m^/Xpa^−/−^* repair mutants and natural ageing, thereby validating the progeria in the double mutant pups.

### Reduced IGF1 Serum Levels, Glucose, and Fat Use in *Csb^m/m^/Xpa^−/−^* Mice

**Figure 7:**
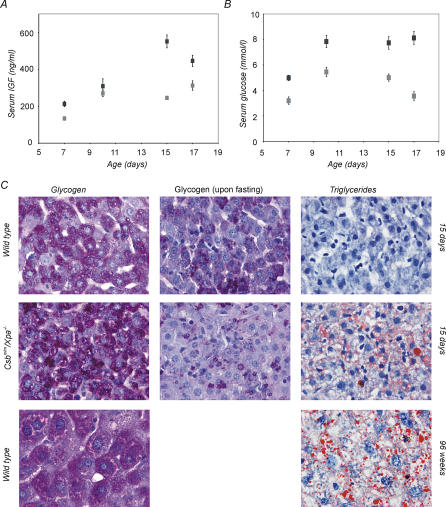
Carbohydrate/Fat Metabolism and IGF1 Serum Levels IGF1 (A) and glucose (B) in the serum of 7-, 10-, 15-, and 17-d-old wt, *Xpa^−/−^, Csb^m/m^,* and *Csb^m/m^/Xpa^−/−^* mice (*n* = 6). The levels of IGF1 (ng/ml) and glucose (mmol/l) in the serum of *Csb^m/m^/Xpa^−/−^* mice are significantly lower than that of control littermates (*p* < 0.0004 and *p* < 0.04, respectively). (C) PAS staining for glycogen and Oil Red O staining for triglycerides in livers of 15-d-old wt and *Csb^m/m^/Xpa^−/−^* mice and 96-wk-old wt mice. Pictures were taken at 100× magnification. Note the large polyploid nuclei in the 96-wk-old wt mouse liver and the reduced glycogen levels in the *Csb^m/m^/Xpa^−/−^* liver after overnight fasting.

In agreement with the down-regulation of *Igf1* gene expression in the liver (the main source of circulating IGF1 [30]), we observed a significant reduction (*p* < 0.004) in serum IGF1 levels in *Csb^m/m^/Xpa^−/−^* mice (Figure 7A) together with significantly lower blood glucose levels (*p* < 0.04) (Figure 7B). Following an initial reduction of ∼30% (*p* < 0.04) in 7- and 10-d-old *Csb^m/m^/Xpa^−/−^* mice, blood glucose levels started to drop at day 15, gradually reaching low levels in 17-d-old *Csb^m/m^/Xpa^−/−^* mice (∼3 mM), contrasting the steady blood glucose levels (∼ 9 mM) in littermate controls (Figure 7B). The presence of milk and food in the stomach of the double-mutant pups along with the normal appearance of the intestinal epithelium (Figure 3A) indicates that the hypoglycemia is not due to food intake. Even more, the suppression of the somatotroph axis and subsequent decreased IGF1 production in 15-d-old *Csb^m/m^/Xpa^−/−^* mice appeared not to originate from a pituitary dysfunction as histological examination (Figure 3F) and TUNEL staining of sections from the pituitary pars distalis, intermedia, and nervosa did not reveal any abnormalities (unpublished data). Moreover, serum GH levels in 15-d-old *Csb^m/m^/Xpa^−/−^* mice (15.2 ± 4.2 ng/ml, *n* = 8) did not differ significantly from wt littermates (12.8 ± 2.8 ng/ml, *n* = 6). Interestingly, the normal serum GH levels together with the significant systemic down regulation of GH receptor gene expression likely point to growth hormone resistance in 15-d-old *Csb^m/m^/Xpa^−/−^* mice.

Periodic acid-Schiff (PAS) staining of liver sections from 10- to 20-d-old pups and naturally aged mice revealed enhanced accumulation of glycogen in unusually large vesicles in *Csb^m/m^/Xpa^−/−^* pups and 96-wk-old mice when compared to wt littermates and 8-wk-old wt mice (Figure 7C)***.*** This observation fits our microarray data, suggesting that both the *Csb^m/m^/Xpa^−/−^* and naturally aged mice store rather than use glucose. Overnight fasting of *Csb^m/m^/Xpa^−/−^* pups and littermate controls resulted in a near-to-complete depletion of liver glycogen (Figure 7D), indicating that the glycogen accumulation is not due to inability to split glycogen into its constitutive glucose monomers.

Consistent with the broad up-regulation of genes associated with fatty acid synthesis (Table 1), Oil Red O staining of liver sections from 15-d-old pups and naturally aged mice revealed enhanced accumulation of triacylglycerides in both compared to control littermates and 8-wk-old mice (Figure 7C), indicating hepatic steatosis. This and the absence of adipose tissue suggest that *Csb^m/m^/Xpa^−/−^* mice display generalized lipodystrophy (loss and abnormal redistribution of body fat) [31].

### Systemic Changes in Somatotroph Axis and Antioxidant Defense in DEHP-Treated wt Mice

**Figure 8:**
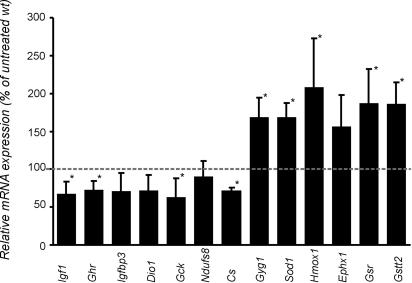
Expression Levels of Genes Associated with the GH/IGF1 Axis, Oxidative Metabolism, and Antioxidant Defense in DEHP-Treated wt Mice Relative mRNA levels of genes involved in the GH/IGF1 growth axis, oxidative metabolism, and antioxidant defense in 13-wk-old wt mice treated with a low dose of the pro-oxidant DEHP. For each gene, expression levels in the treated wt mouse livers are plotted relative to that of age-matched untreated wt littermate controls (dotted line). Error bars indicate SEM. Asterisks indicate statistically significant differences (one-tailed *p* ≤ 0.05, see also Text S1).

To test whether the presence of endogenous (oxidative) DNA damage can provoke the somatotrophic drop and enhanced antioxidant potential, wt C57BL/6J mice (*n* = 6; 4-wk-old) were fed ad libitum for 9 wk with standard food containing subtoxic levels of an oxidative DNA damage-inducing agent [di(2-ethylhexyl)phthalate (DEHP), 1500 ppm] [32]. Neither body weight nor appetite and food intake of DEHP-exposed animals deviated from that of untreated control animals. As shown in Figure 8, subsequent analysis revealed suppression of the expression of genes associated with the somatotroph axis (*Igf1, Igfbp3, Ghr,* and *Dio1)* and oxidative metabolism *(Gck, Cs,* and *Ndufs8),* along with the up-regulation of glycogenin 1 (*Gyg1,* Figure 5A) in DEHP-exposed animals. Consistent with the ability of DEHP to generate ROS-induced DNA damage in the liver, we also noticed a significant up-regulation of genes associated with the antioxidant and detoxification responses *(Hmox1, Ephx1, Gsr, Sod1, Gstt2)*. These findings suggest that the accumulation of unrepaired (oxidative) DNA damage is likely one of the causes underlying the observed suppression of the GH/IGF1 and oxidative metabolism in *Csb^m/m^/Xpa^−/−^* mice (Text S1).

## Discussion

*Csb^m/m^* mice exhibit several CS features (e.g., attenuated growth, blindness, neurological dysfunction), but their phenotype is overall milder than the human syndrome [16] despite the fact that the truncation in the N-terminal part (mimicking a mutant allele of CS-B patient CS1AN) completely inactivates the protein and TC-NER [16]. Although the severity of clinical features in humans does not seem to correlate with the severity of the molecular defect [33], the absence of the complete spectrum of CS features in the *Csb^m/m^* mouse model is likely to originate from human-mouse differences (i.e., adaptation to stress, tolerance to DNA damage/genome instability), rather than from the nature of the *Csb^m/m^* mutation. This idea is supported by our observations that *Xpd^TTD^* and *Xpd^XPCS^* mice (all carrying causative point mutations) also fail to show the severe CS features associated with XPCS and TTD [9,34].

Yet, the present study reveals that inactivation of GG-NER or complete abrogation of NER (through inactivation of *Xpc* or *Xpa,* respectively) in TCR-deficient *Csb^m/m^* mice dramatically aggravates the *Csb^m/m^* mouse phenotype. Because animals were not exposed to exogenous genotoxic agents, we attribute this effect to enhanced levels of unrepaired endogenous (oxidative) DNA damage. In further support of this, we have shown that *Csb^m/m^/Xpc^−/−^* and *Csb^m/m^/Xpa^−/−^* MEFs, as well as *Csb^m/m^/Xpa^−/−^* retinal photoreceptor cells, are more sensitive to environmental genotoxic insults (i.e., UV light and ionizing radiation) than their single mutant counterparts. A comparable phenotypic deterioration has been noticed when *Xpa* was inactivated in *Xpd^TTD^* [9], *Xpd^XPCS^* [34], compound heterozygous *Xpd^TTD/XPCS^* animals (carrying causative mutations for TTD and combined XP/CS) [35] or *Xpg^deltaEx15^* mice [36].

Importantly, *Csb^m/m^/Xpa^−/−^* mice appeared normal at birth, indicating a normal intra-uterine development and ruling out that this condition is in fact an embryonic developmental disorder. Instead, after birth, the *Csb^m/m^/Xpa^−/−^* pups displayed progressive kyphosis, cachexia, photoreceptor loss, and motor dysfunction, all common postnatal manifestations of CS [13], as well as of natural mammalian ageing [37–39]. Also similar to human CS patients (average age 12.5 y), *Csb^m/m^/Xpa^−/−^* pups fail to grow into adulthood and die before weaning. The relation between (residual) repair capacity, time, and severity of a particular phenotype is well illustrated by the retinal photoreceptor loss in the *Csb^m/m^* mouse models. Whereas ageing C57Bl/6J mice lose about 5%–10% of their rods and cones in 30 months, TCR-deficient *Csb^m/m^* mice have already lost about 50% of their photoreceptor cells by the age of 16 months. This spontaneous retinal degeneration in *Csb^m/m^* mice originates from enhanced apoptotic sensitivity of photoreceptor cells (T. Gorgels, I. van der Pluijm, R. Brandt, G. Garinis, H. van Steeg, et al, unpublished data), evolving in the first 1 or 2 months after weaning. Interestingly, further crippling of NER in *Csb^m/m^* animals by inactivation of *Xpa* accelerates the onset of photoreceptor loss, which becomes visible as early as postnatal day 15, and progressively increases thereafter. The strong correlation between the severity of the repair deficiency and the onset of photoreceptor loss, as well as the enhanced ionizing radiation hypersensitivity of photoreceptor cells of *Csb^m/m^/Xpa^−/−^* mice (as compared to age-matched *Csb^m/m^* animals), well support the hypothesis that (oxidative) DNA damage likely underlies the retinal degeneration.

Full genome transcriptome analysis of the *Csb^m/m^/Xpa^−/−^* mouse liver, aiming at unraveling the etiology of the severe double-mutant phenotype, led us to identify significant genome-wide parallels between the 2-wk-old *Csb^m/m^/Xpa^−/−^* and 130-wk, but not 16-wk-old, wt animals at the fundamental level of gene expression. This resemblance was largely attributable to the substantial down-regulation of genes associated with processes implicated in oxidative energy and growth metabolism that were previously revealed by others to represent a conserved transcriptional response in ageing [28].

The down-regulation of genes associated with the GH/IGF1 growth axis in the liver, the systemic reduction in *GH receptor* mRNA levels, and the impaired *Igf1* gene expression in liver and other tissues (resulting in low serum IGF1 levels) likely underlie the postnatal growth defect in *Csb^m/m^/Xpa^−/−^* pups. These changes were not due to reduced GH serum levels or pituitary abnormalities. A steady decline in the GH/IGF1 somatotroph axis was also observed in rodents and humans during natural ageing [40]. Furthermore*, Csb^m/m^/Xpa^−/−^* pups failed to up-regulate metabolism; instead, they displayed a sharp systemic reduction in the expression levels of genes involved in glycolysis, tricarboxylic acid cycle (including decreased citrate synthase activity), and oxidative respiration, which coincided with the onset of weight loss (cachexia). In addition, *Csb^m/m^/Xpa^−/−^* pups up-regulated genes associated with glycogen and fatty acid synthesis, leading to increased hepatic glycogen storage and fat accumulation (steatosis) and pronounced hypoglycemia. Simultaneously, subcutaneous fat tissue was virtually absent. Given that in mammals, the GH/IGF1 signaling pathway is one of the major regulators of energy homeostasis to integrate metabolism with growth [30,41,42], it is tempting to speculate that reduced IGF1 signaling is responsible for the postnatal metabolic shift and growth defect seen in *Csb^m/m^/Xpa^−/−^* mice. Interestingly, several CS patients have been previously reported with hypoglycemia and low IGF1 serum levels [43,44], low metabolic rate [45], and abnormal fat deposition [46].

Paradoxically, however, the systemic suppression of the somatotrophic axis and energy metabolism, along with the up-regulation of antioxidant defenses, low IGF1 serum levels, and low blood glucose levels observed in the *Csb^m/m^/Xpa^−/−^* mouse, are all associated with increased longevity rather than with the short lifespan of this mouse model. In lower paradigms for lifespan extension *(C. elegans, D. melanogaster),* genetic interference in the insulin-signaling pathway can prolong life multi-fold [47,48]. In mammals, IGF1-deficient, Ames and Snell dwarf mice (characterized by defects in the development of the anterior pituitary due to mutations in the *Prop-1* and *Pit1* loci and diminished levels of GH, thyroid stimulating hormone, and prolactin hormone) combine hypoglycemia, low body temperature, and increased storage of carbohydrates and lipids [40,42] with up-regulation of antioxidant defense capacity and extended lifespan [49,50]. Conversely, GH-overexpressing transgenic mice display reduced lifespan and antioxidant responses [51]. These findings have also been recently confirmed by our identification of genome-wide parallels between the extremely short-lived DNA repair mutants (*Csb^m/m^/Xpa^−/−^* and *Ercc1^−/−^*) and the extremely long-lived Ames and Snell dwarfs and growth hormone receptor knockout *(Ghr^−/−^)* mice (Garinis *et al*. manuscript in preparation). Last but not least, IGF1 plasma levels decline with age in humans and rodents [52–54]. Along with this hormonal shift, ageing cells surmount an intricate antioxidant defense response [55,56] that is thought to prevent the detrimental consequences of oxidative stress. Interestingly, the progressive, age-related decrease in the somatotroph axis has been suggested to confer a selective advantage by postponing the onset of age-related disease and prolonging lifespan through the reduction of toxic free radicals [40].

**Figure 9:**
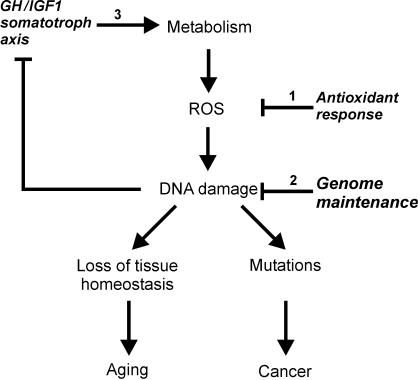
The Proposed Link between DNA Damage and the Decline of the GH/IGF1 Somatotroph Axis ROS are natural byproducts of metabolism and can injure several macromolecules including DNA, thus contributing to a slow but steady accumulation of damage, transcriptional stress, impaired replication, and eventually the progressive loss of tissue homeostasis and gradual organismal deterioration. Cells and tissues will respond by (1) up-regulating their antioxidant defense responses that would moderate the harmful effects of ROS, (2) using a battery of genome maintenance pathways that would repair or remove damaged macromolecules and help to resist the oxidative stress, and (3) suppressing their GH/IGF1 somatotroph axis along with the oxidative metabolism, thus substantially moderating their metabolic activity that would otherwise lead to high oxygen consumption and increased generation of oxidants. To this end, the physiologic reduction of the somatotroph axis and oxidative metabolism is envisaged to be beneficial in terms of net lifespan.

How would repair-deficient mice benefit from such a response? During development, the mitogenic action of GH and IGF1 fuels cellular metabolism, thereby promoting tissue growth and function [40,57,58]. A high metabolic activity, however, leads to higher oxygen consumption [40] and may also increase the ROS burden through the parallel increase of mitochondrial electron transport, peroxisomal fatty acid metabolism, and/or microsomal cytochrome *P-450* enzymes [59]. Despite antioxidant defense and DNA repair, oxidative DNA damage will still accumulate, leading to transcriptional stress, impaired replication, cellular senescence, malfunction or death and eventually to progressive loss of tissue homeostasis and organismal decline (Figure 9, model). We hypothesize that complete abrogation of NER (by inactivation of *Xpa*) renders TCR-deficient *Csb^m/m^* mice unable to adequately cope with the increased burden of DNA damage in the transcribed strand of active genes. This triggers an adaptive response; i.e., reduction of metabolic activity through down-regulation of the GH/IGF1 axis to relieve the pressure on their genome. We interpret this as an attempt to limit the deleterious effects of arrested transcription, such as cellular senescence and death causing accelerated ageing. As a consequence, the initially normal growth becomes arrested soon after birth, leading to severe growth retardation. This scenario provides a plausible explanation for the growth defect in CS patients. However, this response is unable to fully compensate for the repair defect, and thus damage still accumulates to critical levels and triggers apoptosis and/or senescence, thereby leading to ageing-associated pathology such as neurodegeneration (as illustrated by the photoreceptor cells in *Csb^m/m^/Xpa^−/−^* mice).

The conceptual link between DNA damage and the systemic adaptive response is supported by our observation that chronic exposure of wt mice to a sub-toxic dose of DEHP (a pro-oxidant that enhances the DNA damage load; [60]) triggers a response similar to that observed in (untreated) *Csb^m/m^/Xpa^−/−^* mice. Although DEHP at much higher concentrations has been previously documented to affect the endocrine function of the pituitary, proteome analysis revealed that synthesis of prolactin and growth hormone appears unaffected in DEHP-treated rats [61]. This suggests that the observed suppression of genes associated with the somatotroph axis and oxidative metabolism in the liver of DEHP-exposed mice is triggered by DNA damage in the liver, rather than by a pituitary defect or hypothalamic defect.

As one would predict, other short-lived NER mouse models (e.g., *Xpg* and *Xpf* mice [62,63]) or NER mutant mice with a milder progeroid phenotype could also show accelerated attenuation of the somatotrophic axis in response to their DNA repair defect. Indeed, *Ercc1^−/−^* animals, carrying a combined NER/crosslink DNA repair defect and a lifespan of only a few weeks, demonstrate a remarkable genome-wide similarity in liver gene expression profiles with *Csb^m/m^/Xpa^−/−^* mice (L. Niedernhofer, G. Garinis, A. Raams, A. Lalai, A. Rasile Robinson, et al., unpublished data), whereas *Xpd^XPCS^/Xpa^−/−^* and compound heterozygous *Xpd^TTD/XPCS^/Xpa^−/−^* mice contain lower serum IGF1 levels [35]. Furthermore, *Xpd^TTD^* mice, which manifest accelerated ageing in many (but not all) organs and tissues, have recently been shown to display features related to a caloric restricted–like phenotype and suppression of the GH/IGF1 axis in a limited set of organs and tissues, stressing the segmental nature that is characteristic of all progeroid syndromes and the systemic nature of the response [64]. Finally, proper glucose homeostasis and normal IGF1 levels were recently shown to require activity of Sirt6, a chromatin deacetylase that may promote DNA repair [65]. Because ROS-mediated DNA damage appears to be the underlying cause of the *Csb^m/m^/Xpa^−/−^* progeria, it is tempting to speculate that one can attenuate the premature onset of age-related features by directly counteracting the harmful byproducts of metabolism (ROS) and, consequently, DNA damage. An antioxidant-based nutraceutical intervention pilot study with *Csb^m/m^/Xpa^−/−^* mice, aiming at extending lifespan and delaying onset of pathology, yielded promising results (I. van der Pluijm, R. Brandt, J. Hoeijmakers, G. van der Horst, unpublished data).

## Materials and Methods

### Animals.

The generation and characterization of NER-deficient *Xpa^−/−^, Xpc^−/−^,* and *Csb^m/m^* mice has been previously described [16,22,66,67]. *p53^ −/−^* mice [68] were kindly provided by T. Jacks (Massachusetts Institute of Technology, Cambridge, Massachusetts, United States). Unless stated otherwise, all mice were kept in a C57BL/6J genetic background. In the DEHP exposure study, 4-wk-old male wt mice (C57BL/6J; *n* = 6) were put on a DEHP (1500 ppm; Sigma, St. Louis, Missouri, United States) containing diet or on a regular diet for 9 wk. Animals were screened daily for discomfort and weighed once a week. Food consumption was registered by weighing the food. In the ionizing irradiation exposure study, 18-d-old *Csb^m/m^/Xpa^−/−^* and littermate control animals (*n* = 4–6/genotype) were exposed to 10 Gy, killed 20 h after exposure, and their eyes were further processed. Additional information on the isolation and processing of the eyes is provided in the Text S1. As required by Dutch law, all animal studies were approved by an independent Animal Ethical Committee (Dutch equivalent of the Institutional Animal Care and Use Committee). Further information on mouse crossing, genotyping, housing, and macroscopic examination is described in the Text S1.

### Cellular sensitivity studies.

UV sensitivity was determined as described [69]. Sparsely seeded Petri dish cultures were exposed to different doses of UV (254 nm, Phillips TUV lamp). After 4 d, the number of proliferating cells was estimated from the amount of radioactivity incorporated during a 2-hr pulse with [^3^H] thymidine. Cell survival was expressed as the percentage of radioactivity in exposed cells in relation to the radioactivity in untreated cells. UV-induced GG repair was assayed using the unscheduled DNA synthesis (UDS) method described in [70]. In brief, coverslip-grown cells were exposed to 16 J/m^2^ of 254-nm UV light and labeled with [^3^H] thymidine. Repair capacity was quantified by grain counting after autoradiography. RNA synthesis recovery was measured according to [71]. In short, coverslip-grown cells were exposed to 10 J/m^2^ of 254-nm UV light, allowed to recover for 16 hr, labeled with [^3^H] uridine, and processed for autoradiography. The relative rate of RNA synthesis was expressed as *G*_UV_/*G*_C_ (percentage), where *G*_UV_ and *G*_C_ represent the number of grains over UV-exposed and nonexposed nuclei, respectively.

Ionizing radiation sensitivity of immortalized MEFs was determined using a colony assay. Cells were plated in 6-cm-diameter dishes at various densities. After 16 h, cells were exposed to a single dose of ionizing radiation (^137^Cs source; dose range of 0 to 8 Gy. Cells were grown for another 5 to 14 d, and after fixation and staining, colonies were counted. All experiments were performed in triplicate.

### Immunohistological examination and blood parameters.

Detailed histopathological examination was performed on all organs and tissues. Paraffin-embedded tissues were sectioned at 5 μm and stained with haematoxylin/eosin (HE) solution. Liver sections were stained with PAS or Oil Red O (cryosections) to detect glycogen and triglycerides, respectively. Detailed information on the immunohistochemical procedures is described in Text S1. Apoptotic cells were detected using a TUNEL assay as described by the manufacturer (Apoptag Plus Peroxidase In Situ Apoptosis Detection Kit, Chemicon, Temecula, California, United States). For retinal evaluation, eyes were marked nasally with Alcian blue (5% Alcian blue in 96% ethanol), enucleated, fixed in 4 % paraformaldehyde in 0.1 M phosphate buffer, washed in PBS, and embedded in paraffin. Horizontal sections (5 μm thick) of the retina were cut, and sections in the middle of the retina were selected by Alcian blue marking and proximity of the optic nerve. Sections were stained for degenerating cells by TUNEL according to the manufacturer's instructions (Apoptag Plus Peroxidase In Situ Apoptosis Detection Kit, Chemicon). For quantification, the number of TUNEL-positive cells in the INL and ONL were counted in six whole sections per mouse. Differences between the genotypes were tested for statistical significance using multivariate ANOVA, followed by a posthoc test of Student-Newman-Keuls (S-N-K). Significance was set at *p* < 0.05. Serum IGF1 and GH levels were determined with the active mouse/rat IGF1 enzyme-linked immunosorbent assay (ELISA) and active mouse/rat GH ELISA kits respectively, as described by the manufacturer (Diagnostic Systems Laboratories Inc., Texas, United States). Blood glucose was measured using a Freestyle mini blood glucose measurement device (Abbott Diabetes Care, Amersfoort, The Netherlands).

### Radiography and microcomputed tomography.

Mice were anaesthetized by intraperitoneal injection of ketalin and rompun (120 and 7.5 μg/g body weight, respectively). Lateral films were taken at 2× magnification using a CGR Senograph 500T x-ray system operated at 30 kV and 32 mAS [9]. Formalin fixed tibiae from wt and mutant mice were scanned from proximal end to mid-diaphysis, using a SkyScan 1072 microtomograph (SkyScan, Antwerp, Belgium) with a voxel size of 8.82 μm. Scans were processed, and 2-D images of the bones were obtained.

### Footprint studies.

Footprint analysis was performed by painting the hind and fore paws of the mice with different colors of water-soluble nontoxic paints. Animals were allowed to walk along a 30 × 7 cm walled runway, lined with paper, into a darkened, enclosed space. Tests were performed in duplicate at day 15 and 19. Footprint patterns were analyzed for (1) stride length, measured as the average distance between each stride; (2) front base width; and (3) hind base width, measured as the average distance between contralateral footprints [72].

### Microarray analysis and Q-PCR evaluation.

Standard procedures were used to obtain total RNA (Qiagen, Valencia, California, United States) from the liver of wt, *Xpa^−/−^, Csb^m/m^,* and *Csb^m/m^/Xpa^−/−^* mice (*n* = 4) at postnatal day 15 and from the liver of 8-, 16-, 96-, and 130-wk-old mice (*n* = 4). Synthesis of double stranded cDNA and biotin-labeled cRNA was performed according to the instructions of the manufacturer (Affymetrix, Santa Clara, California, United States). Fragmented cRNA preparations were hybridized to full mouse genome oligonucleotide arrays (430 V2.0; Affymetrix). Q-PCR was performed with a DNA Engine Opticon device (Bio-Rad Laboratories B.V., Veenendaal, The Netherlands). Detailed information on microarray hybridization, microarray data analysis, gene ontology classification, and analysis of overrepresented biological themes, as well as on Q-PCR data analysis and used primer pair sequences is described in Text S1. Microarrays were complied with the Minimum Information for Microarray Experiments (MIAME, E-MEXP-835 and E-MEXP-839).

## Supporting Information

Figure S1 Q-PCR Evaluation of mRNA Levels of Genes Associated with the GH/IGF1 Axis, Antioxidant Defense, and Oxidative Metabolism in 2-wk-Old *Csb^m/m^/Xpa^−/−^* and 96-wk-Old wt Mice (114 KB TIF)

Table S1 Frequency of Viable *Csb^m/m^/Xpa^−/−^* and *Csb^m/m^/Xpc^−/−^* Mice and Parallels of Human and Mouse CS Symptoms (A) Frequency of viable *Csb^m/m^/Xpa^−/−^* mice; (B) frequency of viable *Csb^m/m^/Xpc^−/−^* mice; (C) parallels of human CS symptoms with the mild *Csb^m/m^* and severe *Csb^m/m^/Xpa^−/−^* mouse models.
(13 KB PDF)

Table S2 Extensive Overview of Significant Expression Changes in *Csb^m/m^/Xpa^−/−^, Csb^m/m^,* and *Xpa^−/−^* Mice Compared to wt Littermate Controls (129 KB PDF)

Table S3 Extensive Overview of Significant Expression Changes in 130-wk-Old wt Mice Compared to 8-wk-Old Mice (92 KB PDF)

Table S4 Extensive Overview of Significant Expression Changes in 96-wk-Old wt Mice Compared to 8-wk-Old Mice (121 KB PDF)

Table S5 Extensive Overview of Significant Expression Changes in 16-wk-Old wt Mice Compared to 8-wk-Old Mice (106 KB PDF)

Table S6 Extensive Overview of Gene Expression Profiles Associated with Significantly Over-represented Biological Processes in 96- and 130-wk-Old Naturally Aged Mice (13 KB PDF)

Table S7 Comparison of Gene Expression Profiles Associated with Significantly Over-represented Biological Processes between *Csb^m/m^/Xpa^−/−^, Csb^m/m^,* and *Xpa^−/−^* Mice and 96- and 130-wk-Old Naturally Aged Mice (11 KB PDF)

Text S1 Supplementary Methods (42 KB DOC)

## Abbreviations

ANOVA: analysis of variance
BrdU: bromodeoxyuridine
CS: Cockayne syndrome
DEHP: di(2-ethylhexyl)phthalate
GG-NER: global genome–nucleotide excision repair
GH: growth hormone
IGF1: insulin-like growth factor 1
INL: inner nuclear layer
IR: ionizing radiation
MEF: mouse embryonic fibroblast
NER: nucleotide excision repair
ONL: outer nuclear layer
PAS: periodic acid-Schiff
ROS: reactive oxygen species
SEM: standard error of the mean
TC-NER: transcription-coupled nucleotide excision repair
TCR: transcription-coupled repair
TTD: trichothiodystrophy
TUNEL: terminal deoxynucleotidyltransferase-mediated dUTP nick end labeling
XP: xeroderma pigmentosum
wt: wild-type
