# Correction: Impaired Genome Maintenance Suppresses the Growth Hormone–Insulin-Like Growth Factor 1 Axis in Mice with Cockayne Syndrome

**DOI:** 10.1371/journal.pbio.0060304

**Published:** 2008-11-25

**Authors:** Ingrid van der Pluijm, George A Garinis, Renata M. C Brandt, Theo G. M. F Gorgels, Susan W Wijnhoven, Karin E. M Diderich, Jan de Wit, James R Mitchell, Conny van Oostrom, Rudolf Beems, Laura J Niedernhofer, Susana Velasco, Errol C Friedberg, Kiyoji Tanaka, Harry van Steeg, Jan H. J Hoeijmakers, Gijsbertus T. J van der Horst

Correction for:

van der Pluijm I, Garinis GA, Brandt RMC, Gorgels TGMF, Wijnhoven SW, et al. (2007) Impaired genome maintenance suppresses the growth hormone–insulin-like growth factor 1 axis in mice with Cockayne syndrome. PLoS Biol 5(1): e2. doi:10.1371/journal.pbio.0050002

J. H. J. Hoeijmakers wishes to declare the following competing interest, which should have been indicated in the research article. Dr. Hoeijmakers owned shares in DNage, a company set up by Erasmus Medical Center, and subsequently acquired by Pharming. However, for clarification, no materials or support were received from either company, and no agreements were in place concerning the execution or publication of this work.

