# Supplementary material for: Impaired Genome Maintenance Suppresses the Growth Hormone–Insulin-Like Growth Factor 1 Axis in Mice with Cockayne Syndrome
Source: PLoS Biol. 2006 Dec 12;5(1):e2. doi: 10.1371/journal.pbio.0050002 (PMC1698505; doi:10.1371/journal.pbio.0050002)
Supplement: Supplementary file 1 [file pbio.0050002.st001.pdf]

Supplementary Table S1.

A. Frequency of viable *Csb*$^{m/m}$*Xpa*$^{-/-}$ mice

Csb$^{m/m}$*Xpa*$^{+/-}$ x *Csb*$^{m/m}$*Xpa*$^{+/-}$

Xpa	*Csb*	number	expected (%)	observed (%)
+/+	m/m	20	25	26
+/-	m/m	45	50	60
-/-	m/m	11	25	14*
		76	100	100

*p<0.01

B. Frequency of viable *Csb*$^{m/m}$*Xpc*$^{-/-}$ mice

Csb$^{+/m}$*Xpc*$^{-/-}$ x *Csb*$^{+/m}$*Xpc*$^{-/-}$

Xpc	*Csb*	number	expected (%)	observed (%)
-/-	+/+	14	25	23
-/-	+/m	41	50	69
-/-	m/m	5	25	8*
		60	100	100

*p<0.001

C. Parallels of human CS symptoms with the mild *Csb*$^{m/m}$ and severe *Csb*$^{m/m}$*Xpa*$^{-/-}$ mouse models

**CS phenotype**	***Csb*$^{m/m}$**	***Csb*$^{m/m}$*Xpa*$^{-/-}$**
**UV sensitivity**	+	++
**cachexia**	-	+
**premature death**	-	+
**growth retardation**	+	++
**relatively long limbs**	-	+
**kyphosis**	-	+
**ataxia**	-	+
**dystonia**	-	+
**Purkinje cell loss**	-	+
**retinal degeneration**	+	++
