# Supplementary material for: Impaired Genome Maintenance Suppresses the Growth Hormone–Insulin-Like Growth Factor 1 Axis in Mice with Cockayne Syndrome
Source: PLoS Biol. 2006 Dec 12;5(1):e2. doi: 10.1371/journal.pbio.0050002 (PMC1698505; doi:10.1371/journal.pbio.0050002)
Supplement: Supplementary file 2 [file pbio.0050002.st002.pdf]

Supplementary Table S2

Extensive overview of significant expression profiles of $Csb^{m/m}/Xpa^{-/-}$, $Csb^{m/m}$ and $Xpa^{-/-}$ mice compared to *wt* littermate controls. FC: fold change, p: p-value.

Code	Gene Title	Gene Symbol	*Csb m/m Xpa -/-*		*Csb m/m*		*Xpa -/-*	
			FC	p	FC	p	FC	p
1419197_x_at	hepcidin antimicrobial peptide 1	Hamp1	10.70	0.003	1.35	0.295	-1.25	0.527
1442339_at	stefin A2 like 1	MGI:3524944	6.49	0.000	1.15	0.082	1.42	0.023
1423556_at	aldo-keto reductase family 1, member B7	Akr1b7	6.30	0.000	-1.14	0.561	1.28	0.187
1417761_at	apolipoprotein A-IV	Apoa4	5.25	0.000	-1.08	0.468	1.30	0.113
1415938_at	serine protease inhibitor, Kazal type 3	Spink3	4.80	0.001	1.67	0.018	1.90	0.003
1448595_a_at	reduced expression 3	Rex3	4.41	0.000	-1.12	0.602	-1.28	0.291
1419762_at	ubiquitin D	Ubd	4.21	0.000	1.22	0.099	1.44	0.001
1419136_at	aldo-keto reductase family 1, member C18	Akr1c18	4.04	0.000	1.70	0.028	1.68	0.022
1420394_s_at	glycoprotein 49 A /// leukocyte immunoglobulin-like receptor, subfamily B, member 4	Gp49a /// Lilrb4	3.75	0.000	1.72	0.003	1.87	0.000
1417388_at	brain expressed X-linked 2	Bex2	3.71	0.000	1.21	0.263	1.43	0.060
1419149_at	serine (or cysteine) proteinase inhibitor, clade E, member 1	Serpine1	3.71	0.006	1.26	0.466	1.61	0.284
1416474_at	neighbor of Punc E11	Nope	3.65	0.000	1.00	0.870	1.00	0.786
1419647_a_at	immediate early response 3	Ier3	3.46	0.001	-1.01	0.954	1.21	0.418
1455457_at	cytochrome P450, family 2, subfamily c, polypeptide 50 /// cytochrome P450	Cyp2c50 /// Cyp2c5	3.23	0.006	-1.30	0.292	1.16	0.455
1433924_at	Paternally expressed 3	Peg3	3.17	0.000	1.29	0.117	1.09	0.605
1458297_s_at	macrophage receptor with collagenous structure	Marco	3.01	0.000	1.34	0.162	1.17	0.471
1456156_at	leptin receptor	Lepr	2.93	0.000	-1.25	0.460	-1.79	0.141
1417441_at	DnaJ (Hsp40) homolog, subfamily C, member 12	Dnajc12	2.91	0.000	1.34	0.097	1.24	0.213
1452592_at	microsomal glutathione S-transferase 2	Mgst2	2.89	0.000	1.10	0.101	1.02	0.116
1448964_at	S100 calcium binding protein G	S100g	2.79	0.002	-1.13	0.475	1.31	0.111
1415977_at	myo-inositol 1-phosphate synthase A1	MGI:1919030	2.76	0.000	1.22	0.309	1.25	0.139
1436881_x_at	alpha fetoprotein	Afp	2.76	0.000	1.01	0.919	1.10	0.567
1417883_at	glutathione S-transferase, theta 2	Gstt2	2.76	0.000	-1.30	0.034	1.18	0.063
1416332_at	cold inducible RNA binding protein	Cirbp	2.73	0.000	-1.08	0.433	1.15	0.150
1451095_at	asparagine synthetase	Asns	2.72	0.000	1.33	0.055	1.32	0.008
1417370_at	trefoil factor 3, intestinal	Tff3	2.65	0.001	-1.00	0.988	1.17	0.382
1416022_at	fatty acid binding protein 5, epidermal	Fabp5	2.62	0.000	1.43	0.069	1.00	0.998
1452905_at	GTL2, imprinted maternally expressed untranslated mRNA /// latrophilin 1	Gtl2 /// Lphn1	2.62	0.000	1.28	0.058	-1.11	0.510
1454607_s_at	phosphoserine aminotransferase 1	Psat1	2.62	0.000	1.37	0.016	1.22	0.126
1420984_at	Phosphatidylcholine transfer protein	Pctp	2.58	0.000	1.54	0.048	1.60	0.021
1439426_x_at	P lysozyme structural	Lzp-s	2.53	0.005	-1.01	0.957	-1.04	0.878
1428842_a_at	nerve growth factor receptor (TNFRSF16) associated protein 1	Ngfrap1	2.51	0.000	1.24	0.160	1.11	0.391
1455269_a_at	coronin, actin binding protein 1A	Coro1a	2.51	0.000	1.09	0.506	1.45	0.008

1448152_at	insulin-like growth factor 2	Igf2	2.50	0.001	2.21	0.025	1.30	0.431
1428484_at	oxysterol binding protein-like 3	Osbpl3	2.47	0.000	-1.03	0.571	-1.04	0.429
1454971_x_at	transforming growth factor beta 1 induced transcript 4	Tgfb1i4	2.44	0.000	1.28	0.109	1.60	0.002
1456003_a_at	solute carrier family 1 member 4	Slc1a4	2.43	0.000	1.98	0.001	1.87	0.001
1456212_x_at	suppressor of cytokine signaling 3	Socs3	2.43	0.010	1.42	0.044	-1.14	0.680
1448239_at	heme oxygenase (decycling) 1	Hmox1	2.43	0.000	-1.17	0.531	-1.48	0.168
1426808_at	lectin, galactose binding, soluble 3	Lgals3	2.41	0.000	1.72	0.002	1.32	0.081
1420697_at	solute carrier family 15, member 3	Slc15a3	2.41	0.000	1.88	0.001	1.51	0.012
1460014_at	triggering receptor expressed on myeloid cells-like 4	Treml4	2.40	0.000	1.60	0.018	1.53	0.014
1423467_at	membrane-spanning 4-domains, subfamily A, member 4B	Ms4a4b	2.40	0.000	1.24	0.080	1.13	0.253
1419004_s_at	B-cell leukemia/lymphoma 2 related protein A1a	Bcl2a1a /// Bcl2a1b	2.38	0.000	1.85	0.002	1.53	0.029
1433485_x_at	G protein-coupled receptor 56	Gpr56	2.37	0.000	1.39	0.112	1.09	0.388
1427981_a_at	cysteine sulfinic acid decarboxylase	Csad	2.36	0.000	-1.25	0.239	-1.22	0.308
1449009_at	T-cell specific GTPase	Tgtp	2.36	0.005	2.03	0.020	1.27	0.353
1427076_at	macrophage expressed gene 1	Mpeg1	2.35	0.000	1.91	0.001	1.46	0.014
1440240_at	neuropeptide B	MGI:2387153	2.28	0.000	1.22	0.015	1.11	0.165
1437247_at	fos-like antigen 2	Fosl2	2.28	0.000	1.33	0.233	1.27	0.295
1437811_x_at	Coactosin-like 1 (Dictyostelium)	Cotl1	2.28	0.000	1.62	0.008	1.52	0.007
1455309_at	transmembrane protein 16F	Tmem16f	2.26	0.000	1.42	0.241	1.88	0.006
1455439_a_at	lectin, galactose binding, soluble 1	Lgals1	2.23	0.000	1.27	0.100	1.30	0.065
1435176_a_at	inhibitor of DNA binding 2	Id2	2.23	0.000	1.28	0.060	1.26	0.104
1450138_a_at	serine (or cysteine) proteinase inhibitor, clade B, member 6a	Serpinb6a	2.23	0.000	1.19	0.131	1.41	0.006
1422411_s_at	eosinophil-associated, ribonuclease A family, member 1	Ear1 /// Ear2 /// Ear3	2.22	0.000	1.91	0.006	1.74	0.002
1447845_s_at	vanin 1	Vnn1	2.21	0.000	1.13	0.385	1.11	0.396
1419394_s_at	S100 calcium binding protein A8 (calgranulin A)	S100a8	2.21	0.010	-1.17	0.339	-1.01	0.966
1448169_at	keratin complex 1, acidic, gene 18	Krt1-18	2.20	0.000	1.01	0.919	1.06	0.473
1418930_at	chemokine (C-X-C motif) ligand 10	Cxcl10	2.20	0.006	1.98	0.045	1.50	0.096
1426284_at	keratin 20	Krt20	2.20	0.003	1.08	0.471	1.27	0.024
1415822_at	stearoyl-Coenzyme A desaturase 2	Scd2	2.19	0.000	1.18	0.083	1.21	0.024
1429523_a_at	solute carrier family 39 (metal ion transporter), member 5	Slc39a5	2.18	0.000	1.31	0.088	1.36	0.042
1448986_x_at	deoxyribonuclease II alpha	Dnase2a	2.18	0.000	-1.24	0.130	-1.19	0.247
1451527_at	procollagen C-endopeptidase enhancer 2	Pcolce2	2.18	0.000	1.50	0.023	1.57	0.006
1448883_at	legumain	Lgmn	2.17	0.000	1.70	0.018	1.69	0.001
1450779_at	fatty acid binding protein 7, brain	Fabp7	2.16	0.000	1.66	0.002	1.52	0.031
1425895_a_at	inhibitor of DNA binding 1	Id1	2.16	0.000	1.16	0.271	-1.13	0.375
1437325_x_at	aldehyde dehydrogenase 18 family, member A1	Aldh18a1	2.15	0.000	1.60	0.015	1.24	0.132
1457063_at	Phosphatase, orphan 1	Phospho1	2.14	0.000	1.58	0.000	1.19	0.125
1419714_at	CD274 antigen	Cd274	2.13	0.000	1.71	0.011	1.14	0.004
1435549_at	transient receptor potential cation channel, subfamily M, member 4	Trpm4	2.13	0.000	1.16	0.219	1.05	0.703
1452445_at	solute carrier family 41, member 2	Slc41a2	2.12	0.004	1.24	0.133	1.34	0.038
1452352_at	cytotoxic T lymphocyte-associated protein 2 beta	Ctla2b	2.12	0.000	1.58	0.006	1.40	0.048

1415996_at	thioredoxin interacting protein	Txnip	2.11	0.008	1.05	0.765	1.05	0.761
1422438_at	epoxide hydrolase 1, microsomal	Ephx1	2.10	0.000	-1.12	0.268	1.14	0.169
1447096_at	Pre B-cell leukemia transcription factor 1	Pbx1	2.10	0.000	1.56	0.036	-1.03	0.895
1417813_at	inhibitor of kappaB kinase epsilon	Ikbke	2.09	0.001	1.34	0.100	1.28	0.131
1418123_at	unc-119 homolog (C. elegans)	Unc119	2.09	0.000	1.21	0.075	1.33	0.000
1449222_at	Epstein-Barr virus induced gene 3	Ebi3	2.09	0.000	1.60	0.006	1.55	0.003
1450061_at	ectodermal-neural cortex 1	Enc1	2.09	0.000	1.08	0.460	1.06	0.623
1426208_x_at	Pleiomorphic adenoma gene-like 1	Plagl1	2.09	0.000	1.41	0.030	1.28	0.063
1450830_a_at	phosphodiesterase 6C, cGMP specific, cone, alpha prime	Pde6c	2.09	0.003	1.34	0.236	1.39	0.180
1451206_s_at	pleckstrin homology, Sec7 and coiled-coil domains, binding protein	Pscdbp	2.08	0.001	1.22	0.242	1.15	0.515
1417079_s_at	lectin, galactose-binding, soluble 2	Lgals2	2.08	0.004	-1.04	0.794	-1.02	0.918
1455318_at	T-cell immunoglobulin and mucin domain containing 4	Timd4	2.07	0.000	1.88	0.002	1.63	0.005
1434376_at	CD44 antigen	Cd44	2.06	0.000	1.87	0.002	1.48	0.029
1460218_at	CD52 antigen	Cd52	2.06	0.000	1.91	0.001	1.54	0.011
1448748_at	pleckstrin	Plek	2.05	0.000	1.33	0.035	1.58	0.002
1419582_at	cytochrome P450, family 2, subfamily c, polypeptide 55	Cyp2c55	2.04	0.009	-1.10	0.776	1.40	0.242
1460351_at	S100 calcium binding protein A11 (calizzarin)	S100a11	2.03	0.000	1.32	0.051	1.23	0.051
1460329_at	UDP-Gal:betaGlcNAc beta 1,4-galactosyltransferase, polypeptide 6	B4galt6	2.03	0.000	1.69	0.001	1.47	0.004
1459338_at	Autism susceptibility candidate 2	Auts2	2.03	0.000	1.37	0.001	-1.03	0.452
1425951_a_at	C-type lectin domain family 4, member n	Clec4n	2.03	0.000	1.71	0.001	1.64	0.000
1448620_at	Fc receptor, IgG, low affinity III	Fcgr3	2.03	0.000	1.47	0.045	1.76	0.004
1424245_at	carboxylesterase 2	Ces2	2.02	0.000	1.16	0.172	1.39	0.021
1416460_at	aldehyde reductase (aldose reductase)-like 6	Aldrl6	2.01	0.000	1.26	0.195	1.35	0.061
1447830_s_at	regulator of G-protein signaling 2	Rgs2	2.00	0.002	1.67	0.006	1.56	0.024
1457724_at	cathepsin L	Ctsl	2.00	0.000	1.20	0.137	1.16	0.160
1451563_at	EGF-like module containing, mucin-like, hormone receptor-like sequence 4	Emr4	2.00	0.000	1.56	0.009	1.69	0.006
1441946_at	inter-alpha (globulin) inhibitor H5	Itih5	2.00	0.000	-1.21	0.341	1.10	0.648
1418204_s_at	allograft inflammatory factor 1	Aif1	2.00	0.001	1.74	0.002	1.54	0.011
1420715_a_at	peroxisome proliferator activated receptor gamma	Pparg	1.99	0.000	1.11	0.221	1.53	0.000
1439631_at	zinc finger, CCHC domain containing 11	Zcchc11	1.99	0.002	1.30	0.236	1.20	0.451
1423700_at	replication factor C (activator 1) 3	Rfc3	1.98	0.000	1.46	0.051	1.81	0.003
1449193_at	CD5 antigen-like	Cd5l	1.98	0.000	1.69	0.006	1.32	0.092
1448377_at	secretory leukocyte protease inhibitor	Slpi	1.97	0.003	-1.22	0.162	-1.20	0.182
1434449_at	aquaporin 4	Aqp4	1.96	0.001	1.31	0.023	1.31	0.046
1416066_at	CD9 antigen	Cd9	1.96	0.000	-1.13	0.131	-1.07	0.396
1454942_at	niban protein	Niban	1.95	0.000	1.40	0.024	1.47	0.008
1420404_at	CD86 antigen	Cd86	1.95	0.001	1.47	0.036	1.57	0.011
1454268_a_at	cytochrome b-245, alpha polypeptide	Cyba	1.94	0.000	1.51	0.005	1.49	0.004
1448510_at	ephrin A1	Efna1	1.94	0.002	1.16	0.519	1.85	0.011
1424923_at	serine (or cysteine) proteinase inhibitor, clade A, member 3G	Serpina3g	1.94	0.001	2.04	0.002	1.44	0.077
1417860_a_at	spondin 2, extracellular matrix protein	Spon2	1.93	0.000	1.38	0.016	1.40	0.027

1416950_at	tumor necrosis factor, alpha-induced protein 8	Tnfaip8	1.93	0.000	1.29	0.051	1.27	0.054
1421855_at	fibrinogen-like protein 2	Fgl2	1.92	0.000	1.42	0.002	1.28	0.006
1417025_at	histocompatibility 2, class II antigen E beta	H2-Eb1	1.92	0.000	1.08	0.384	1.19	0.101
1454078_a_at	galactose-3-O-sulfotransferase 1	Gal3st1	1.92	0.000	-1.17	0.169	1.05	0.610
1434079_s_at	minichromosome maintenance deficient 2 mitotin (S. cerevisiae)	Mcm2	1.92	0.000	1.48	0.019	1.27	0.138
1437726_x_at	complement component 1, q subcomponent, beta polypeptide	C1qb	1.91	0.000	1.50	0.001	1.20	0.145
1418712_at	CDC42 effector protein (Rho GTPase binding) 5	Cdc42ep5	1.90	0.000	1.56	0.011	1.49	0.003
1453009_at	carboxypeptidase M	Cpm	1.90	0.000	1.12	0.445	1.32	0.042
1424713_at	calmodulin-like 4	Calml4	1.90	0.000	-1.21	0.275	-1.12	0.520
1421041_s_at	glutathione S-transferase, alpha 2 (Yc2)	Gsta2	1.90	0.003	-1.59	0.083	-1.18	0.458
1426306_a_at	melanoma antigen, family D, 2	Maged2	1.90	0.000	1.47	0.016	1.42	0.019
1421375_a_at	S100 calcium binding protein A6 (calcyclin)	S100a6	1.90	0.000	1.11	0.284	1.12	0.274
1423547_at	lysozyme	Lyzs	1.89	0.000	1.21	0.022	1.46	0.001
1456037_x_at	prolactin regulatory element binding	Preb	1.89	0.000	1.46	0.025	1.51	0.002
1449259_at	RAB3D, member RAS oncogene family	Rab3d	1.89	0.000	1.16	0.176	1.29	0.003
1450792_at	TYRO protein tyrosine kinase binding protein	Tyrobp	1.88	0.000	1.64	0.006	1.63	0.002
1416811_s_at	cytotoxic T lymphocyte-associated protein 2 alpha	Ctla2a /// Ctla2b	1.87	0.000	1.35	0.020	1.47	0.005
1456227_x_at	retinoblastoma binding protein 7	Rbbp7	1.87	0.000	1.14	0.022	-1.09	0.192
1452771_s_at	acyl-CoA synthetase long-chain family member 3	Acsl3	1.87	0.008	1.52	0.069	1.66	0.025
1455990_at	kinesin family member 23	Kif23	1.87	0.002	1.79	0.002	1.25	0.330
1445781_at	SH3 domain protein D19	Sh3d19	1.86	0.000	1.39	0.016	1.20	0.204
1452205_x_at	T-cell receptor beta, variable 13	Tcrb-V13	1.86	0.000	1.11	0.343	1.18	0.120
1460373_a_at	open reading frame 21	ORF21	1.86	0.000	1.23	0.071	1.23	0.095
1449846_at	eosinophil-associated, ribonuclease A family, member 2	Ear2	1.86	0.000	1.50	0.009	1.27	0.036
1455454_at	similar to 3(20)alpha-hydroxysteroid/dihydrodiol/indanol dehydrogenase	LOC432720	1.85	0.000	1.07	0.522	1.26	0.111
1448756_at	S100 calcium binding protein A9 (calgranulin B)	S100a9	1.85	0.010	-1.10	0.495	1.05	0.752
1437724_x_at	phosphatidylinositol membrane-associated 1	Pitpnm1	1.84	0.000	1.26	0.043	1.30	0.014
1420678_a_at	interleukin 17 receptor B	Il17rb	1.84	0.000	1.42	0.003	1.28	0.012
1435989_x_at	keratin complex 2, basic, gene 8	Krt2-8	1.84	0.000	-1.05	0.750	1.04	0.783
1456169_at	similar to KAT protein	LOC226654	1.84	0.000	1.29	0.024	1.03	0.823
1450352_at	melatonin receptor 1A	Mtnr1a	1.84	0.001	-1.09	0.313	1.07	0.574
1435906_x_at	guanylate nucleotide binding protein 2	Gbp2	1.84	0.001	1.60	0.022	1.01	0.973
1456483_at	zinc finger protein 9	Zfp9	1.84	0.000	1.13	0.390	-1.16	0.303
1449363_at	activating transcription factor 3	Atf3	1.84	0.006	1.24	0.191	-1.05	0.763
1419700_a_at	prominin 1	Prom1	1.84	0.001	1.34	0.128	1.04	0.865
1448111_at	cytidine 5'-triphosphate synthase 2	Ctps2	1.83	0.000	1.07	0.347	1.12	0.091
1448561_at	neutrophil cytosolic factor 2	Ncf2	1.82	0.000	1.38	0.014	1.27	0.046
1417267_s_at	FK506 binding protein 11	Fkbp11	1.82	0.000	1.15	0.246	1.34	0.025
1418340_at	Fc receptor, IgE, high affinity I, gamma polypeptide	Fcer1g	1.82	0.000	1.66	0.004	1.65	0.002
1436448_a_at	prostaglandin-endoperoxide synthase 1	Ptgs1	1.82	0.000	1.43	0.009	1.38	0.016
1424302_at	paired-Ig-like receptor B	Pirb	1.81	0.000	1.17	0.185	1.39	0.004

1417900_a_at	very low density lipoprotein receptor	Vldlr	1.81	0.001	-1.08	0.408	1.01	0.920
1450070_s_at	p21 (CDKN1A)-activated kinase 1	Pak1	1.81	0.001	1.28	0.111	1.56	0.001
1460245_at	killer cell lectin-like receptor, subfamily D, member 1	Klrd1	1.81	0.000	1.17	0.048	1.26	0.008
1451814_a_at	HIV-1 tat interactive protein 2, homolog (human)	Htatip2	1.81	0.000	-1.00	0.957	1.11	0.221
1422903_at	lymphocyte antigen 86	Ly86	1.81	0.010	1.79	0.016	1.49	0.085
1449049_at	toll-like receptor 1	Tlr1	1.81	0.000	1.20	0.020	1.27	0.001
1427912_at	carbonyl reductase 3	Cbr3	1.80	0.000	1.22	0.090	1.01	0.876
1452016_at	arachidonate 5-lipoxygenase activating protein	Alox5ap	1.80	0.000	1.09	0.475	1.18	0.127
1420699_at	C-type lectin domain family 7, member a	Clec7a	1.80	0.000	1.53	0.009	2.07	0.001
1419125_at	protein tyrosine phosphatase, non-receptor type 18	Ptpn18	1.80	0.000	1.24	0.089	1.44	0.003
1437660_at	Natural killer tumor recognition sequence	Nktr	1.80	0.000	1.02	0.849	-1.38	0.018
1419091_a_at	annexin A2	Anxa2	1.80	0.000	1.29	0.008	1.35	0.000
1418196_at	telomerase associated protein 1	Tep1	1.80	0.009	1.68	0.018	1.61	0.028
1423192_at	paraspeckle protein 1	Pspc1	1.79	0.003	1.04	0.833	1.14	0.384
1457356_at	Insulin-like growth factor 2 receptor	Igf2r	1.79	0.001	-1.25	0.163	-1.20	0.257
1448617_at	CD53 antigen	Cd53	1.79	0.000	1.56	0.007	1.52	0.019
1427123_s_at	coatomer protein complex, subunit gamma 2, antisense 2	Copg2as2	1.78	0.001	1.04	0.764	1.44	0.014
1424815_at	glycogen synthase 2	Gys2	1.78	0.000	-1.01	0.888	-1.03	0.699
1434881_s_at	potassium channel tetramerisation domain containing 12	Kctd12	1.78	0.000	1.33	0.020	1.37	0.009
1434897_a_at	solute carrier family 25, member 4	Slc25a4	1.78	0.000	1.07	0.637	1.01	0.930
1418131_at	SAM domain and HD domain, 1	Samhd1	1.78	0.000	1.51	0.014	1.29	0.066
1422124_a_at	protein tyrosine phosphatase, receptor type, C	Ptprc	1.78	0.000	1.42	0.007	1.38	0.009
1418649_at	EGL nine homolog 3 (C. elegans)	Egln3	1.77	0.002	1.10	0.501	1.37	0.025
1419132_at	toll-like receptor 2	Tlr2	1.77	0.000	1.18	0.139	1.21	0.029
1420249_s_at	chemokine (C-C motif) ligand 6	Ccl6	1.77	0.000	1.20	0.074	1.16	0.126
1456307_s_at	Adenylate cyclase 7	Adcy7	1.77	0.000	1.44	0.004	1.42	0.000
1451036_at	spastic paraplegia 21 homolog (human)	Spg21	1.77	0.000	1.22	0.122	1.55	0.001
1440822_x_at	RalBP1 associated Eps domain containing protein	Reps1	1.76	0.000	1.03	0.704	1.05	0.536
1456907_at	chemokine (C-X-C motif) ligand 9	Cxcl9	1.75	0.006	1.17	0.035	1.19	0.050
1426622_a_at	glutaminyl-peptide cyclotransferase (glutaminyl cyclase)	Qpct	1.75	0.001	1.26	0.196	1.27	0.153
1434194_at	Microtubule-associated protein 2	Mtap2	1.75	0.000	1.17	0.021	-1.02	0.492
1426604_at	ribonuclease L (2', 5'-oligoisoadenylate synthetase-dependent)	Rnasel	1.75	0.000	1.36	0.005	1.12	0.302
1455678_at	Semaphorin 4B	Sema4b	1.75	0.006	1.05	0.734	1.06	0.718
1424576_s_at	cytochrome P450, family 2, subfamily c, polypeptide 44	Cyp2c44	1.75	0.000	1.04	0.777	1.12	0.298
1419349_a_at	cytochrome P450, family 2, subfamily d, polypeptide 9	Cyp2d9	1.75	0.000	1.03	0.802	1.18	0.156
1440637_at	Intersectin 1 (SH3 domain protein 1A)	Itsn1	1.75	0.002	1.12	0.422	-1.44	0.077
1449007_at	B-cell translocation gene 3	Btg3	1.75	0.000	1.14	0.075	1.03	0.630
1452384_at	ectonucleotide pyrophosphatase/phosphodiesterase 3	Enpp3	1.74	0.000	1.08	0.420	1.10	0.290
1455106_a_at	creatine kinase, brain	Ckb	1.74	0.001	1.43	0.016	1.34	0.069
1453473_a_at	t-complex testis expressed 1	Tctex1	1.74	0.003	1.43	0.062	1.63	0.010
1422953_at	formyl peptide receptor, related sequence 2	Fpr-rs2	1.73	0.001	1.32	0.017	1.06	0.556

1423848_at	M phase phosphoprotein 6	Mphosph6	1.73	0.001	-1.06	0.696	1.24	0.141
1433504_at	brain glycogen phosphorylase	Pygb	1.73	0.000	1.05	0.712	1.08	0.660
1422013_at	C-type lectin domain family 4, member a2	Clec4a2	1.73	0.001	1.31	0.004	1.32	0.002
1429177_x_at	SRY-box containing gene 17	Sox17	1.73	0.001	1.44	0.018	1.08	0.598
1417346_at	PYD and CARD domain containing	Pycard	1.73	0.001	1.36	0.069	1.60	0.001
1418261_at	spleen tyrosine kinase	Syk	1.73	0.001	1.49	0.013	1.50	0.007
1439478_at	mitochondrial acyl-CoA thioesterase 1	MGI:2159605	1.73	0.003	1.01	0.934	1.14	0.205
1457551_at	Activin A receptor, type 1	Acvr1	1.73	0.004	1.21	0.304	-1.04	0.835
1443540_at	mitogen activated protein kinase kinase kinase 1	Map3k1	1.72	0.000	-1.03	0.579	1.13	0.134
1443870_at	ATP-binding cassette, sub-family C (CFTR/MRP), member 4	Abcc4	1.72	0.000	1.31	0.024	1.13	0.258
1440935_at	Growth factor receptor bound protein 10	Grb10	1.72	0.000	1.94	0.010	2.34	0.000
1423606_at	periostin, osteoblast specific factor	Postn	1.72	0.001	1.41	0.025	1.27	0.081
1449455_at	hemopoietic cell kinase	Hck	1.72	0.000	1.57	0.006	1.37	0.017
1419296_at	Rho GTPase activating protein 4	Arhgap4	1.72	0.001	1.49	0.000	1.21	0.057
1425407_s_at	C-type lectin domain family 4, member a2 /// C-type lectin domain family 4, member b	Clec4a2 /// Clec4b	1.71	0.000	1.19	0.030	1.43	0.000
1417649_at	cyclin-dependent kinase inhibitor 1C (P57)	Cdkn1c	1.71	0.001	1.14	0.183	1.25	0.096
1451230_a_at	WW domain binding protein 5	Wbp5	1.71	0.000	1.09	0.307	-1.04	0.700
1451651_at	V-set and immunoglobulin domain containing 4	Vsig4	1.70	0.000	1.37	0.017	1.58	0.001
1421811_at	thrombospondin 1	Thbs1	1.70	0.002	1.22	0.195	1.25	0.131
1442884_at	hepatocyte growth factor	Hgf	1.70	0.002	-1.05	0.795	1.07	0.739
1417697_at	sterol O-acyltransferase 1	Soat1	1.70	0.000	1.22	0.127	1.08	0.563
1460337_at	SH3-domain kinase binding protein 1	Sh3kbp1	1.70	0.000	1.23	0.059	1.40	0.015
1434400_at	TGFB-induced factor 2	Tgif2	1.70	0.000	1.08	0.461	1.18	0.194
1448315_a_at	pyrroline-5-carboxylate reductase family, member 2	Pycr2	1.70	0.001	1.19	0.210	1.62	0.001
1419435_at	aldehyde oxidase 1	Aox1	1.69	0.000	-1.01	0.914	1.34	0.035
1445282_at	Muskelin 1, intracellular mediator containing kelch motifs	Mkln1	1.69	0.002	1.16	0.374	1.05	0.762
1424754_at	membrane-spanning 4-domains, subfamily A, member 7	Ms4a7	1.69	0.001	1.45	0.007	1.45	0.025
1421182_at	C-type lectin domain family 1, member b	Clec1b	1.69	0.000	1.40	0.017	1.33	0.023
1452899_at	RNA imprinted and accumulated in nucleus	Rian	1.69	0.000	1.07	0.065	-1.00	0.566
1455495_at	V-abl Abelson murine leukemia viral oncogene 2 (arg, Abelson-related gene)	AA536808	1.69	0.000	1.31	0.041	1.15	0.230
1418213_at	keratin complex 1, acidic, gene 23	Krt1-23	1.68	0.003	1.09	0.587	1.31	0.048
1428733_at	guanine nucleotide binding protein, gamma transducing activity polypeptide 2	Gngt2	1.68	0.001	1.53	0.007	1.40	0.018
1456328_at	B-cell scaffold protein with ankyrin repeats 1	Bank1	1.68	0.003	1.53	0.015	1.56	0.015
1425350_a_at	myelin basic protein expression factor 2, repressor	Myef2	1.68	0.001	1.57	0.015	1.18	0.244
1453181_x_at	phospholipid scramblase 1	Plscr1	1.68	0.000	1.17	0.119	1.21	0.054
1422256_at	somatostatin receptor 2	Sstr2	1.68	0.000	1.15	0.100	1.25	0.006
1430295_at	guanine nucleotide binding protein, alpha 13	Gna13	1.67	0.000	1.09	0.262	1.12	0.145
1438001_x_at	deleted in polyposis 1	Dp1	1.67	0.001	1.25	0.095	1.20	0.177
1454728_s_at	ATPase, aminophospholipid transporter (APLT), class I, type 8A, member 1	Atp8a1	1.66	0.000	1.59	0.001	1.58	0.000
1448162_at	vascular cell adhesion molecule 1	Vcam1	1.66	0.001	1.26	0.117	1.11	0.502
1456739_x_at	armadillo repeat containing, X-linked 2	Armcx2	1.66	0.000	1.45	0.004	1.18	0.047

1449164_at	CD68 antigen	Cd68	1.66	0.003	1.71	0.004	1.45	0.023
1456006_at	BCL2-like 11 (apoptosis facilitator)	Bcl2l11	1.66	0.001	-1.01	0.929	1.10	0.384
1448490_at	aarF domain containing kinase 4	Adck4	1.66	0.001	1.24	0.100	1.17	0.233
1424531_a_at	transcription elongation factor A (SII), 3	Tcea3	1.65	0.006	1.43	0.022	1.58	0.006
1446167_at	Homeodomain interacting protein kinase 2	Hipk2	1.65	0.000	1.27	0.074	-1.02	0.878
1418142_at	potassium inwardly-rectifying channel, subfamily J, member 8	Kcnj8	1.65	0.000	1.27	0.020	1.56	0.000
1460243_at	serine palmitoyltransferase, long chain base subunit 2	Sptlc2	1.65	0.001	1.15	0.331	1.22	0.186
1451386_at	biliverdin reductase B (flavin reductase (NADPH))	Blvrb	1.65	0.000	-1.07	0.288	1.04	0.538
1418394_a_at	CD97 antigen	Cd97	1.65	0.000	1.35	0.018	1.31	0.016
1441324_at	Zinc finger protein 395	Zfp395	1.65	0.004	1.00	1.000	1.06	0.759
1456700_x_at	Myristoylated alanine rich protein kinase C substrate	Marcks	1.65	0.000	1.15	0.038	1.08	0.348
1460273_a_at	baculoviral IAP repeat-containing 1b	Birc1b	1.65	0.004	1.18	0.215	1.32	0.022
1418703_at	RNA binding motif, single stranded interacting protein 1	Rbms1	1.64	0.000	1.04	0.701	-1.11	0.300
1435343_at	dedicator of cytokinesis 10	Dock10	1.64	0.000	1.37	0.009	1.20	0.133
1441259_s_at	WD repeat domain 10	Wdr10	1.64	0.000	1.08	0.344	-1.05	0.546
1449852_a_at	EH-domain containing 4	Ehd4	1.64	0.000	1.48	0.017	1.31	0.005
1459098_at	Dystonin	Dst	1.63	0.000	1.06	0.367	1.05	0.595
1415983_at	lymphocyte cytosolic protein 1	Lcp1	1.63	0.000	1.35	0.009	1.41	0.004
1460559_at	Ankyrin repeat domain 25	Ankrd25	1.63	0.000	1.41	0.002	1.53	0.000
1424067_at	intercellular adhesion molecule	Icam1	1.63	0.000	1.11	0.263	1.23	0.025
1433568_at	PAP associated domain containing 4	Papd4	1.63	0.001	1.26	0.208	1.20	0.166
1422997_s_at	cytosolic acyl-CoA thioesterase 1 /// mitochondrial acyl-CoA thioesterase 1	Cte1 /// MGI:21596(	1.63	0.002	1.21	0.092	1.25	0.091
1428154_s_at	phosphatidic acid phosphatase type 2 domain containing 1	Ppapdc1	1.63	0.000	1.12	0.304	1.17	0.055
1438882_at	Rho GTPase activating protein 18	Arhgap18	1.63	0.000	1.03	0.669	-1.12	0.258
1451461_a_at	aldolase 3, C isoform	Aldoc	1.63	0.001	1.11	0.183	1.08	0.470
1425425_a_at	Wnt inhibitory factor 1	Wif1	1.63	0.007	-1.87	0.031	-1.75	0.047
1416591_at	RAB34, member of RAS oncogene family	Rab34	1.63	0.000	1.11	0.249	1.20	0.035
1444746_at	Polypyrimidine tract binding protein 2	Ptbp2	1.62	0.002	-1.23	0.314	-1.26	0.254
1452907_at	galactosylceramidase	Galc	1.62	0.001	1.26	0.095	1.49	0.010
1428018_a_at	Cd300C antigen	Cd300c	1.62	0.000	1.28	0.050	1.53	0.002
1450355_a_at	capping protein (actin filament), gelsolin-like	Capg	1.62	0.000	1.32	0.006	1.17	0.124
1442320_at	Phosphatidylethanolamine binding protein	Pbp	1.62	0.001	1.11	0.513	-1.07	0.687
1416046_a_at	fucosidase, alpha-L- 2, plasma	Fuca2	1.62	0.000	1.31	0.031	1.60	0.003
1426454_at	Rho, GDP dissociation inhibitor (GDI) beta	Arhgdib	1.62	0.000	1.22	0.015	1.14	0.056
1450648_s_at	histocompatibility 2, class II antigen A, beta 1 /// response to metastatic cancers 1	H2-Ab1 /// Rmcs1	1.61	0.000	1.15	0.156	1.08	0.215
1448937_at	solute carrier family 35, member B3	Slc35b3	1.61	0.000	1.06	0.427	1.15	0.048
1459897_a_at	suprabasin	MGI:2446326	1.61	0.000	-1.05	0.524	1.15	0.184
1451160_s_at	poliovirus receptor	Pvr	1.61	0.004	1.21	0.213	1.14	0.436
1436933_at	A kinase (PRKA) anchor protein 13	Akap13	1.61	0.004	1.41	0.023	1.03	0.880
1419111_at	inhibitor of growth family, member 1-like	Ing1l	1.61	0.001	1.15	0.151	1.21	0.056
1430979_a_at	peroxiredoxin 2	Prdx2	1.61	0.001	1.34	0.025	1.75	0.000

1444735_at	Zinc and ring finger 2	D6Ertd365e	1.61	0.000	1.23	0.066	1.27	0.016
1424674_at	solute carrier family 39 (metal ion transporter), member 6	Slc39a6	1.61	0.000	1.13	0.094	1.05	0.518
1460378_a_at	testis derived transcript	Tes	1.61	0.000	1.01	0.945	-1.05	0.641
1451161_a_at	EGF-like module containing, mucin-like, hormone receptor-like sequence 1	Emr1	1.61	0.001	1.49	0.003	1.37	0.006
1419267_at	nuclear transcription factor-Y beta	Nfyb	1.61	0.010	1.45	0.024	1.55	0.006
1417620_at	RAS-related C3 botulinum substrate 2	Rac2	1.60	0.000	1.36	0.004	1.21	0.036
1425079_at	transmembrane 6 superfamily member 2	Tm6sf2	1.60	0.000	1.22	0.080	1.22	0.030
1438945_x_at	gap junction membrane channel protein alpha 1	Gja1	1.60	0.007	1.04	0.842	-1.07	0.678
1450852_s_at	coagulation factor II (thrombin) receptor	F2r	1.60	0.000	1.15	0.112	1.12	0.199
1452457_a_at	protein kinase, AMP-activated, beta 1 non-catalytic subunit	Prkab1	1.60	0.007	1.04	0.810	1.50	0.035
1454997_at	Methionine sulfoxide reductase B3	Msrb3	1.60	0.000	1.17	0.027	1.20	0.005
1455385_at	SEC15-like 1 (S. cerevisiae)	Sec15l1	1.60	0.001	1.13	0.224	1.13	0.227
1423931_s_at	anaphase promoting complex subunit 4	Anapc4	1.59	0.000	1.29	0.020	1.27	0.019
1451139_at	solute carrier family 39 (zinc transporter), member 4	Slc39a4	1.59	0.001	-1.04	0.679	-1.06	0.547
1438377_x_at	solute carrier family 13 (sodium-dependent dicarboxylate transporter), member 3	Slc13a3	1.59	0.000	1.07	0.383	-1.26	0.102
1416656_at	chloride intracellular channel 1	Clic1	1.59	0.000	1.28	0.007	1.26	0.004
1419178_at	CD3 antigen, gamma polypeptide	Cd3g	1.59	0.000	1.16	0.079	1.08	0.272
1441484_at	Transcription factor 2	Tcf2	1.59	0.002	1.08	0.549	-1.03	0.799
1429527_a_at	phospholipid scramblase 1 /// similar to phospholipid scramblase 1	Plscr1 /// LOC43332	1.59	0.000	1.03	0.745	1.00	0.975
1436890_at	UDP-N-acteylglucosamine pyrophosphorylase 1-like 1	Uap1l1	1.59	0.000	1.10	0.228	1.07	0.402
1437185_s_at	thymosin, beta 10	Tmsb10	1.59	0.000	1.38	0.004	1.22	0.085
1418884_x_at	tubulin, alpha 1	Tuba1	1.59	0.001	1.07	0.539	1.13	0.283
1425519_a_at	Ia-associated invariant chain	Ii	1.59	0.002	-1.23	0.093	-1.05	0.689
1415906_at	thymosin, beta 4, X chromosome	Tmsb4x	1.59	0.000	1.35	0.002	1.21	0.032
1456933_at	Transcribed locus, similar to Als2 protein [Mus musculus]	---	1.58	0.005	1.22	0.225	1.05	0.787
1416357_a_at	melanoma cell adhesion molecule	Mcam	1.58	0.007	1.25	0.177	1.30	0.101
1455660_at	Colony stimulating factor 2 receptor, beta 1, low-affinity (granulocyte-macrophage)	Csf2rb1	1.58	0.000	1.25	0.064	1.00	0.970
1425225_at	Fc fragment of IgG, low affinity IIIa, receptor	Fcgr3a	1.58	0.007	1.77	0.003	1.22	0.207
1451352_s_at	metastasis associated 3	Mta3	1.58	0.002	1.27	0.112	1.74	0.001
1417292_at	interferon gamma inducible protein 47	Ifi47	1.58	0.001	1.66	0.013	1.52	0.002
1449401_at	complement component 1, q subcomponent, gamma polypeptide	C1qg	1.58	0.004	1.64	0.005	1.28	0.074
1418641_at	lymphocyte cytosolic protein 2	Lcp2	1.58	0.002	1.47	0.009	1.63	0.001
1457117_at	nuclear factor, erythroid derived 2, like 2	Nfe2l2	1.58	0.005	1.06	0.098	1.14	0.010
1454809_at	nuclear receptor coactivator 7	Ncoa7	1.58	0.003	1.71	0.010	1.39	0.031
1450424_a_at	interleukin 18 binding protein	Il18bp	1.57	0.008	1.50	0.024	1.57	0.007
1455940_x_at	WD repeat domain 6	Wdr6	1.57	0.000	1.05	0.412	1.07	0.433
1454808_at	EF hand domain family A1	Efha1	1.57	0.000	1.37	0.001	1.37	0.000
1430165_at	serine/threonine kinase 17b (apoptosis-inducing)	Stk17b	1.57	0.000	1.06	0.562	1.05	0.620
1448605_at	ras homolog gene family, member C	Rhoc	1.57	0.000	1.20	0.037	1.11	0.300
1418097_a_at	thymic stromal-derived lymphopoietin, receptor	Tslpr	1.57	0.000	1.25	0.013	1.30	0.003
1433741_at	CD38 antigen	Cd38	1.57	0.000	1.19	0.018	1.02	0.741

1446750_at	imprinted and ancient	Impact	1.57	0.004	-1.28	0.192	-1.06	0.746
1424759_at	arrestin domain containing 4	Arrdc4	1.57	0.000	1.12	0.384	1.08	0.500
1430575_a_at	tripeptidyl peptidase II	Tpp2	1.57	0.000	1.17	0.115	1.24	0.019
1452117_a_at	FYN binding protein	Fyb	1.57	0.009	1.34	0.097	1.25	0.196
1438643_at	Calcium/calmodulin-dependent protein kinase ID	Camk1d	1.57	0.001	1.15	0.182	1.06	0.614
1449968_s_at	acyl-Coenzyme A thioesterase 2, mitochondrial	MGI:1928939 /// MG	1.57	0.000	1.42	0.002	1.32	0.031
1428988_at	ATP-binding cassette, sub-family C (CFTR/MRP), member 3	Abcc3	1.57	0.001	-1.09	0.485	-1.08	0.532
1454830_at	fibrillin 2	Fbn2	1.57	0.002	1.38	0.000	1.00	0.946
1424653_at	tetraspanin 15	Tspan15	1.57	0.002	1.25	0.047	1.28	0.028
1417893_at	sideroflexin 3	Sfxn3	1.57	0.001	-1.09	0.420	1.06	0.575
1425214_at	pyrimidinergic receptor P2Y, G-protein coupled, 6	P2ry6	1.57	0.001	1.19	0.144	1.28	0.042
1426810_at	jumonji domain containing 1A	Jmjd1a	1.56	0.001	1.22	0.110	1.50	0.003
1456659_at	AT rich interactive domain 1B (Swi1 like)	Arid1b	1.56	0.003	-1.05	0.803	-1.02	0.909
1427137_at	carboxylesterase 5	Ces5	1.56	0.001	1.32	0.016	1.22	0.074
1417039_a_at	cullin 7	Cul7	1.56	0.000	1.08	0.427	1.08	0.384
1451707_s_at	solute carrier family 41, member 3	Slc41a3	1.56	0.001	1.35	0.027	1.19	0.157
1458617_at	protein kinase C binding protein 1	Prkcbp1	1.56	0.001	1.44	0.010	1.27	0.064
1456226_x_at	discoidin domain receptor family, member 1	Ddr1	1.56	0.008	-1.03	0.824	1.16	0.299
1452279_at	properdin factor, complement	Pfc	1.56	0.004	1.64	0.006	1.54	0.008
1415673_at	phosphoserine phosphatase	Psph	1.56	0.004	1.21	0.143	1.31	0.037
1448061_at	Macrophage scavenger receptor 1	Msr1	1.56	0.000	1.13	0.116	1.12	0.125
1458710_at	Polymerase (RNA) II (DNA directed) polypeptide A	Polr2a	1.56	0.001	-1.04	0.799	-1.08	0.646
1416808_at	nidogen 1	Nid1	1.56	0.000	1.19	0.033	-1.03	0.739
1416514_a_at	fascin homolog 1, actin bundling protein (Strongylocentrotus) purpuratus)	Fscn1	1.55	0.000	1.22	0.015	1.15	0.142
1435031_at	transmembrane protein induced by tumor necrosis factor alpha	MGI:2686991	1.55	0.000	-1.06	0.439	-1.00	0.950
1427005_at	polo-like kinase 2 (Drosophila)	Plk2	1.55	0.002	1.10	0.463	1.36	0.011
1448471_a_at	cytotoxic T lymphocyte-associated protein 2 alpha	Ctla2a	1.55	0.002	1.19	0.112	1.07	0.522
1415802_at	solute carrier family 16 (monocarboxylic acid transporters), member 1	Slc16a1	1.55	0.001	1.27	0.028	1.16	0.138
1417376_a_at	immunoglobulin superfamily, member 4A	Igsf4a	1.55	0.002	1.33	0.006	1.34	0.005
1426677_at	filamin, alpha	Flna	1.55	0.001	1.31	0.022	1.19	0.167
1450625_at	procollagen, type V, alpha 2	Col5a2	1.55	0.003	1.32	0.073	1.14	0.362
1429958_x_at	hydroxyacylglutathione hydrolase-like	Haghl	1.55	0.003	1.21	0.141	1.29	0.017
1419492_s_at	defensin beta 1	Defb1	1.55	0.000	1.15	0.164	1.19	0.150
1452500_at	trimethyllysine hydroxylase, epsilon	Tmlhe	1.55	0.005	1.05	0.731	-1.34	0.150
1419873_s_at	colony stimulating factor 1 receptor	Csf1r	1.55	0.002	1.42	0.008	1.63	0.001
1424095_at	RNA terminal phosphate cyclase domain 1	Rtcd1	1.55	0.002	1.20	0.142	1.38	0.010
1420911_a_at	milk fat globule-EGF factor 8 protein	Mfge8	1.55	0.000	1.02	0.817	1.05	0.655
1453283_at	phosphoglucomutase 1	Pgm1	1.55	0.001	1.29	0.021	1.31	0.015
1439154_at	nucleoporin 98	Nup98	1.55	0.004	-1.01	0.932	1.16	0.283
1439436_x_at	inner centromere protein	Incenp	1.55	0.001	1.47	0.004	1.15	0.232
1448863_a_at	tumor necrosis factor, alpha-induced protein 1 (endothelial)	Tnfaip1	1.54	0.000	1.18	0.057	1.17	0.073

1456698_s_at	heterogeneous nuclear ribonucleoprotein D-like	Hnrpdl	1.54	0.000	1.10	0.134	-1.07	0.296
1456388_at	ATPase, class VI, type 11A	Atp11a	1.54	0.000	1.33	0.029	1.09	0.447
1456653_a_at	methylenetetrahydrofolate dehydrogenase (NADP+ dependent) 1-like	Mthfd1l	1.54	0.001	1.03	0.778	-1.07	0.600
1443695_at	hyaluronic acid binding protein 2	Habp2	1.54	0.000	-1.09	0.487	-1.10	0.389
1425548_a_at	leukocyte specific transcript 1	Lst1	1.54	0.002	1.39	0.016	1.27	0.047
1457658_x_at	annexin A4	Anxa4	1.54	0.004	1.39	0.001	1.35	0.005
1419599_s_at	membrane-spanning 4-domains, subfamily A, member 11	Ms4a11	1.54	0.002	1.69	0.000	1.13	0.385
1417786_a_at	regulator of G-protein signaling 19	Rgs19	1.54	0.000	1.38	0.010	1.39	0.002
1453840_at	poly A binding protein, cytoplasmic 1	Pabpc1	1.54	0.000	1.06	0.584	-1.04	0.764
1445438_at	DDHD domain containing 1	Ddhd1	1.54	0.004	1.14	0.392	-1.05	0.796
1458469_at	Casitas B-lineage lymphoma b	Cblb	1.54	0.002	-1.12	0.285	-1.12	0.303
1434745_at	cyclin D2	Ccnd2	1.54	0.000	1.25	0.007	1.17	0.037
1449732_at	zinc finger proliferation 1	Zipro1	1.54	0.001	-1.03	0.795	1.00	0.990
1441945_s_at	abhydrolase domain containing 14A	Abhd14a	1.53	0.000	1.24	0.030	1.26	0.015
1436908_at	pericentriolar material 1	Pcm1	1.53	0.000	1.18	0.029	1.21	0.016
1450638_at	programmed cell death 5	Pdcd5	1.53	0.000	1.16	0.005	-1.02	0.725
1418809_at	paired-Ig-like receptor A1	Pira1	1.53	0.008	1.35	0.060	1.70	0.013
1443937_at	Interleukin 2 receptor, beta chain	Il2rb	1.53	0.004	1.09	0.230	-1.04	0.654
1429028_at	dedicator of cytokinesis 11	Dock11	1.53	0.000	1.27	0.008	1.21	0.035
1455181_at	RAS p21 protein activator 2	Rasa2	1.53	0.000	1.11	0.138	1.27	0.005
1436038_a_at	Down syndrome critical region homolog 5 (human)	Dscr5	1.53	0.002	1.22	0.105	1.21	0.113
1456292_a_at	vimentin	Vim	1.53	0.000	1.12	0.262	1.04	0.718
1453586_at	ectonucleoside triphosphate diphosphohydrolase 1	Entpd1	1.53	0.002	1.01	0.885	-1.03	0.352
1434265_s_at	Ankyrin 2, brain	Ank2	1.53	0.003	1.06	0.599	1.15	0.160
1455316_x_at	CCR4 carbon catabolite repression 4-like (S. cerevisiae)	Ccrn4l	1.53	0.001	1.40	0.012	1.37	0.009
1453200_at	retinoic acid induced 1	Rai1	1.53	0.000	1.00	0.970	1.11	0.096
1416410_at	platelet-activating factor acetylhydrolase, isoform 1b, alpha1 subunit	Pafah1b3	1.53	0.000	1.26	0.049	1.37	0.002
1445629_at	Solute carrier family 35 member D1	Slc35d1	1.52	0.006	1.16	0.206	-1.17	0.251
1416807_at	ribosomal protein L36a	Rpl36a	1.52	0.001	1.07	0.560	1.07	0.542
1441917_s_at	transmembrane protein 40	Tmem40	1.52	0.001	-1.02	0.816	1.07	0.495
1449660_s_at	coronin, actin binding protein 1C	Coro1c	1.52	0.000	1.13	0.082	1.13	0.163
1458589_at	Interferon gamma induced GTPase	Igtp	1.52	0.002	1.47	0.026	1.04	0.590
1421408_at	immunoglobulin superfamily, member 6	Igsf6	1.52	0.001	1.39	0.015	1.41	0.020
1448049_at	Jumonji domain containing 1C	Jmjd1c	1.52	0.000	1.19	0.056	-1.04	0.677
1439283_at	Oxysterol binding protein-like 9	Osbpl9	1.52	0.001	1.14	0.326	-1.02	0.870
1455991_at	cysteine conjugate-beta lyase 2	Ccbl2	1.52	0.000	-1.01	0.945	1.04	0.591
1450429_at	calpain 6	Capn6	1.52	0.000	1.18	0.124	-1.13	0.123
1434027_at	Down syndrome critical region gene 1-like 2	Dscr1l2	1.52	0.000	1.30	0.002	1.22	0.006
1428572_at	brain abundant, membrane attached signal protein 1	Basp1	1.52	0.001	1.34	0.023	1.34	0.007
1437176_at	similar to nucleotide-binding oligomerization domains 27	LOC434341	1.52	0.002	1.67	0.002	1.15	0.204
1449360_at	colony stimulating factor 2 receptor, beta 2, low-affinity (granulocyte-macrophage)	Csf2rb2	1.51	0.003	1.26	0.155	1.15	0.276

1434399_at	UDP-N-acetyl-alpha-D-galactosamine	Galnt6	1.51	0.000	1.02	0.499	-1.01	0.598
1446510_at	Pleckstrin homology domain interacting protein	Phip	1.51	0.000	1.00	#DIV/0!	1.00	#DIV/0!
1450702_at	hemochromatosis	Hfe	1.51	0.000	1.34	0.007	1.23	0.010
1444787_at	Chromodomain helicase DNA binding protein 1	Chd1	1.51	0.000	1.32	0.005	1.02	0.165
1460053_at	SET and MYND domain containing 4	Smyd4	1.51	0.004	1.30	0.058	1.33	0.044
1434295_at	RAS guanyl releasing protein 1	Rasgrp1	1.51	0.000	1.25	0.024	1.26	0.021
1421525_a_at	baculoviral IAP repeat-containing 1e	Birc1e	1.51	0.006	1.80	0.000	1.27	0.069
1438155_x_at	phosphatidylinositol glycan, class O	Pigo	1.51	0.001	1.22	0.057	1.27	0.047
1435363_at	pleckstrin homology domain containing, family G (with RhoGef domain) member 1	Plekhg1	1.51	0.002	1.20	0.073	1.11	0.306
1428168_at	myelin protein zero-like 1	Mpzl1	1.51	0.000	1.02	0.806	-1.00	0.964
1429942_at	glucocorticoid modulatory element binding protein 1	Gmeb1	1.51	0.001	1.02	0.850	1.15	0.170
1419598_at	membrane-spanning 4-domains, subfamily A, member 6D	Ms4a6d	1.51	0.001	1.75	0.000	1.37	0.006
1436037_at	Integrin alpha 4	Itga4	1.50	0.010	1.55	0.014	1.44	0.018
1419554_at	CD47 antigen (Rh-related antigen, integrin-associated signal transducer)	Cd47	1.50	0.000	1.39	0.006	1.17	0.076
1456547_at	Sloan-Kettering viral oncogene homolog	Ski	1.50	0.002	1.02	0.905	-1.24	0.234
1443894_at	ecotropic viral integration site 2b	Evi2b	1.50	0.000	1.14	0.111	1.01	0.854
1420688_a_at	sarcoglycan, epsilon	Sgce	1.50	0.000	1.11	0.161	1.10	0.170
1454854_at	organic solute transporter beta	Ostb	1.50	0.003	1.01	0.955	1.27	0.107
1423130_a_at	splicing factor, arginine/serine-rich 5 (SRp40, HRS)	Sfrs5	1.50	0.004	1.10	0.513	1.38	0.035
1428282_at	tubulin-specific chaperone e	Tbce	1.50	0.001	1.18	0.176	1.38	0.010
1417756_a_at	lymphocyte specific 1	Lsp1	1.50	0.001	1.17	0.126	1.08	0.469
1456174_x_at	N-myc downstream regulated gene 1	Ndrg1	1.50	0.001	1.17	0.187	-1.14	0.231
1418301_at	interferon regulatory factor 6	Irf6	1.50	0.009	1.30	0.036	1.34	0.031
1416111_at	CD83 antigen	Cd83	1.50	0.002	1.20	0.033	1.45	0.000
1439612_at	calcium channel, voltage-dependent, N type, alpha 1B subunit	Cacna1b	1.50	0.009	-1.22	0.183	-1.20	0.214
1443231_at	BAI1-associated protein 1	Baiap1	1.50	0.001	-1.16	0.311	-1.27	0.236
1460196_at	carbonyl reductase 1	Cbr1	1.50	0.000	-1.13	0.013	1.05	0.483
1450961_a_at	transcription elongation factor A (SII)-like 3/5	Tceal3 /// LOC3315	1.50	0.000	1.00	#DIV/0!	1.00	#DIV/0!
1431145_a_at	CUE domain containing 2	Cuedc2	1.50	0.000	1.21	0.024	1.20	0.016
1448715_x_at	CCR4 carbon catabolite repression 4-like (S. cerevisiae)	Ccrn4l /// Iap /// 311	1.50	0.000	1.37	0.011	1.34	0.004
1453752_at	ribosomal protein L17	Rpl17	1.50	0.004	-1.14	0.409	1.25	0.144
1419455_at	interleukin 10 receptor, beta	Il10rb	1.50	0.000	1.48	0.001	1.70	0.000
1433575_at	SRY-box containing gene 4	Sox4	1.50	0.004	1.14	0.245	1.13	0.319
1415961_at	integral membrane protein 2C	Itm2c	1.50	0.000	1.15	0.062	1.07	0.445
1436356_at	Sterile alpha motif domain containing 4	Samd4	1.50	0.001	-1.15	0.250	-1.15	0.284
1447270_at	RING1 and YY1 binding protein	Rybp	1.49	0.003	1.00	0.970	-1.00	0.972
1439645_at	adrenergic receptor, alpha 2b	Adra2b	1.49	0.000	1.17	0.039	1.54	0.000
1429173_at	deoxyribonuclease 1-like 1	Dnase1l1	1.49	0.000	1.14	0.184	1.14	0.222
1416414_at	elastin microfibril interfacer 1	Emilin1	1.49	0.008	1.46	0.016	1.24	0.120
1429247_at	annexin A6	Anxa6	1.49	0.003	1.12	0.347	-1.02	0.841
1455900_x_at	transglutaminase 2, C polypeptide	Tgm2	1.49	0.000	1.28	0.001	1.12	0.099

1448300_at	microsomal glutathione S-transferase 3	Mgst3	1.49	0.001	1.02	0.781	1.29	0.009
1434308_at	solute carrier family 43, member 2	Slc43a2	1.49	0.000	1.02	0.864	1.08	0.454
1423627_at	NAD(P)H dehydrogenase, quinone 1	Nqo1	1.49	0.001	1.04	0.658	1.35	0.019
1433590_at	hect domain and RLD 3	Herc3	1.49	0.004	1.00	0.997	1.04	0.725
1431931_a_at	RAB, member of RAS oncogene family-like 2A	Rabl2a	1.49	0.007	1.38	0.028	1.38	0.018
1425811_a_at	cysteine and glycine-rich protein 1	Csrp1	1.49	0.004	1.23	0.154	1.15	0.298
1434158_at	GDP-mannose 4, 6-dehydratase	Gmds	1.49	0.001	1.07	0.472	1.19	0.030
1451648_a_at	folate receptor 2 (fetal)	Folr2	1.49	0.008	1.69	0.002	1.64	0.004
1459839_x_at	BTB (POZ) domain containing 11	Btbd11	1.49	0.000	1.11	0.220	1.00	0.950
1424629_at	breast cancer 1	Brca1	1.49	0.007	1.35	0.049	1.32	0.032
1459494_at	Nucleoporin 88	Nup88	1.49	0.006	1.15	0.160	-1.02	0.777
1419194_s_at	glia maturation factor, gamma	Gmfg	1.49	0.000	1.20	0.018	1.26	0.005
1422449_s_at	reticulocalbin 2	Rcn2	1.49	0.010	1.39	0.035	1.42	0.026
1436097_x_at	Rho GTPase activating protein 9	Arhgap9	1.49	0.000	1.48	0.000	1.28	0.013
1434807_s_at	metaxin 3	Mtx3	1.49	0.008	1.03	0.781	1.00	0.958
1417647_at	sorting nexin 5	Snx5	1.49	0.001	1.27	0.025	1.19	0.083
1450928_at	inhibitor of DNA binding 4	Id4	1.49	0.006	1.04	0.780	-1.05	0.706
1418203_at	phorbol-12-myristate-13-acetate-induced protein 1	Pmaip1	1.49	0.000	1.30	0.009	1.02	0.131
1417492_at	cathepsin B	Ctsb	1.49	0.000	1.34	0.005	1.42	0.001
1436420_a_at	importin 4	Ipo4	1.48	0.001	1.21	0.055	1.12	0.175
1451899_a_at	general transcription factor II I repeat domain-containing 1	Gtf2ird1	1.48	0.005	-1.02	0.849	-1.04	0.790
1434047_x_at	heterogeneous nuclear ribonucleoprotein A2/B1	Hnrpa2b1	1.48	0.001	-1.00	0.983	-1.22	0.071
1438498_at	chemokine (C-X-C motif) ligand 16	Cxcl16	1.48	0.000	1.12	0.209	1.07	0.403
1442798_x_at	hexokinase 3	Hk3	1.48	0.007	1.42	0.014	1.41	0.012
1417856_at	avian reticuloendotheliosis viral (v-rel) oncogene related B	Relb	1.48	0.004	-1.10	0.330	1.08	0.462
1416326_at	cysteine-rich protein 1 (intestinal)	Crip1	1.48	0.001	1.22	0.035	1.14	0.160
1448498_at	ribosomal protein S6 kinase, polypeptide 4	Rps6ka4	1.48	0.010	1.60	0.007	1.35	0.033
1421358_at	histocompatibility 2, M region locus 3	H2-M3	1.48	0.002	1.60	0.001	1.41	0.004
1456126_at	mucosa associated lymphoid tissue lymphoma translocation gene 1	Malt1	1.48	0.001	1.35	0.014	1.28	0.016
1435284_at	reticulon 4	Rtn4	1.48	0.008	1.48	0.007	1.19	0.325
1434998_at	IQ motif containing GTPase activating protein 1	Iqgap1	1.48	0.001	1.23	0.036	1.10	0.286
1428101_at	ring finger protein 38	Rnf38	1.48	0.000	1.28	0.009	1.14	0.141
1454961_at	synaptojanin 1	Synj1	1.47	0.000	1.30	0.003	1.24	0.023
1448559_at	flotillin 1	Flot1	1.47	0.000	1.10	0.220	1.03	0.465
1419476_at	ADAM-like, decysin 1	Adamdec1	1.47	0.001	1.47	0.003	1.50	0.000
1448147_at	tumor necrosis factor receptor superfamily, member 19	Tnfrsf19	1.47	0.001	-1.02	0.456	1.33	0.002
1417230_at	Ral GEF with PH domain and SH3 binding motif 2	Ralgps2	1.47	0.008	-1.00	0.397	1.14	0.024
1456691_s_at	steroid 5 alpha-reductase 2-like	Srd5a2l	1.47	0.004	1.35	0.010	1.30	0.025
1451973_at	ubiquitin-activating enzyme E1-domain containing 1	Ube1dc1	1.47	0.004	1.10	0.446	1.33	0.020
1418102_at	hairy and enhancer of split 1 (Drosophila)	Hes1	1.47	0.005	1.10	0.439	-1.16	0.263
1419988_at	mitogen activated protein kinase kinase kinase 7	Map3k7	1.47	0.002	1.29	0.025	1.27	0.041

1436534_at	Sjogren syndrome antigen A2	Ssa2	1.47	0.008	1.15	0.203	1.17	0.182
1422476_at	interferon gamma inducible protein 30	Ifi30	1.47	0.002	1.36	0.009	1.30	0.018
1434705_at	C-terminal binding protein 2	Ctbp2	1.47	0.001	1.09	0.257	1.06	0.489
1420913_at	solute carrier organic anion transporter family, member 2a1	Slco2a1	1.47	0.004	1.18	0.171	1.39	0.010
1458235_at	F-box and leucine-rich repeat protein 17	Fbxl17	1.47	0.007	1.11	0.436	-1.12	0.485
1439249_at	WW domain containing adaptor with coiled-coil	Wac	1.47	0.000	-1.12	0.249	-1.05	0.621
1436101_at	ring finger protein 24	Rnf24	1.47	0.005	1.09	0.361	1.11	0.237
1419100_at	serine (or cysteine) proteinase inhibitor, clade A, member 3N	Serpina3n	1.46	0.003	1.08	0.413	-1.12	0.228
1430317_at	ubiquitin-conjugating enzyme E2, J2 homolog (yeast)	Ube2j2	1.46	0.001	1.29	0.029	1.01	0.949
1451299_at	protein kinase, X-linked	Prkx	1.46	0.001	1.20	0.054	1.26	0.029
1437572_at	DCP1 decapping enzyme homolog b (S. cerevisiae)	Dcp1b	1.46	0.001	1.02	0.181	1.13	0.001
1455719_at	tubulin, beta 5	Tubb5	1.46	0.001	1.33	0.013	1.11	0.115
1436173_at	Deleted in liver cancer 1	Dlc1	1.46	0.001	1.29	0.008	1.51	0.000
1455976_x_at	diazepam binding inhibitor	Dbi	1.46	0.000	1.20	0.028	1.09	0.281
1456098_a_at	engulfment and cell motility 2, ced-12 homolog (C. elegans)	Elmo2	1.46	0.000	1.11	0.300	1.17	0.050
1437162_at	GPI-anchored membrane protein 1	Gpiap1	1.46	0.003	-1.13	0.374	-1.24	0.140
1433636_at	BMS1-like, ribosome assembly protein (yeast)	Bms1l	1.46	0.000	1.17	0.090	1.03	0.790
1421943_at	transforming growth factor alpha	Tgfa	1.46	0.006	1.24	0.025	1.26	0.015
1415855_at	kit ligand	Kitl	1.46	0.004	1.45	0.004	1.27	0.049
1452251_at	neurobeachin	Nbea	1.46	0.001	1.16	0.097	1.05	0.431
1417116_at	solute carrier family 6 (neurotransmitter transporter, creatine), member 8	Slc6a8	1.46	0.000	-1.08	0.401	-1.05	0.583
1418822_a_at	ADP-ribosylation factor 6	Arf6	1.46	0.006	1.07	0.558	-1.07	0.545
1437329_at	Protein tyrosine phosphatase-like (proline instead of catalytic arginine), member b	Ptplb	1.46	0.002	1.17	0.149	-1.00	0.976
1438097_at	RAB20, member RAS oncogene family	Rab20	1.46	0.004	1.22	0.163	1.35	0.021
1451421_a_at	leucine zipper domain protein	MGI:1913299	1.46	0.003	1.15	0.231	1.11	0.370
1431428_a_at	nitric oxide synthase interacting protein	Nosip	1.46	0.000	1.10	0.016	1.24	0.000
1421385_a_at	myosin VIIa	Myo7a	1.45	0.000	1.28	0.043	1.23	0.026
1452939_a_at	phosphatidylinositol transfer protein, cytoplasmic 1	Pitpnc1	1.45	0.003	1.35	0.055	-1.13	0.280
1416630_at	inhibitor of DNA binding 3	Id3	1.45	0.000	1.09	0.248	1.10	0.216
1452051_at	ARP3 actin-related protein 3 homolog (yeast)	Actr3	1.45	0.000	1.27	0.015	1.23	0.018
1460330_at	annexin A3	Anxa3	1.45	0.001	1.18	0.091	1.24	0.012
1449944_a_at	Sec61, alpha subunit 2 (S. cerevisiae)	Sec61a2	1.45	0.001	1.31	0.037	1.05	0.629
1450027_at	syndecan 3	Sdc3	1.45	0.005	1.51	0.006	1.25	0.095
1448467_a_at	tangerin	LOC114601	1.45	0.006	1.02	0.860	1.18	0.120
1423959_at	ropporin 1-like	Ropn1l	1.45	0.007	1.01	0.952	1.23	0.111
1416721_s_at	splicing factor, arginine/serine-rich 6	Sfrs6	1.45	0.000	1.00	0.989	1.01	0.888
1455600_at	ribosomal protein S3	Rps3	1.45	0.001	1.06	0.592	1.18	0.118
1452746_at	ATPase type 13A2	Atp13a2	1.45	0.001	1.23	0.027	1.20	0.023
1417965_at	pleckstrin homology domain containing, family A member 1	Plekha1	1.45	0.002	1.20	0.073	1.09	0.376
1449385_at	hydroxysteroid (17-beta) dehydrogenase 9	Hsd17b9	1.45	0.002	1.01	0.913	1.03	0.780
1426956_a_at	transformation related protein 53 binding protein 1	Trp53bp1	1.45	0.000	1.26	0.014	1.26	0.011

1418073_at	acyl-Coenzyme A thioesterase 2, mitochondrial	MGI:1928939	1.45	0.004	1.30	0.056	1.40	0.005
1424337_at	sorting nexin 15	Snx15	1.45	0.001	1.14	0.110	1.09	0.353
1423429_at	placentae and embryos oncofetal gene	Pem	1.45	0.004	1.07	0.338	1.06	0.311
1430025_at	protein phosphatase 3, catalytic subunit, gamma isoform	Ppp3cc	1.45	0.007	-1.01	0.923	1.09	0.531
1458715_at	Caspase 6	Casp6	1.45	0.004	-1.09	0.198	-1.05	0.470
1455476_a_at	genetic suppressor element 1	Gse1	1.45	0.001	1.19	0.110	1.26	0.016
1435776_at	similar to Hypothetical protein CBG13135	LOC433356	1.44	0.004	1.08	0.442	1.19	0.069
1417112_at	ADP-ribosylation factor-like 2 binding protein	Arl2bp	1.44	0.000	-1.20	0.006	-1.12	0.175
1459363_at	Spinocerebellar ataxia 2 homolog (human)	Sca2	1.44	0.002	-1.06	0.676	-1.16	0.384
1440169_x_at	interferon (alpha and beta) receptor 2	Ifnar2	1.44	0.010	1.25	0.083	1.09	0.489
1440051_at	Protein phosphatase 3, catalytic subunit, alpha isoform	Ppp3ca	1.44	0.001	1.06	0.557	-1.10	0.350
1456125_a_at	dynein, cytoplasmic, light chain 1	Dnclc1	1.44	0.002	1.09	0.232	-1.08	0.316
1449027_at	ras homolog gene family, member U	Rhou	1.44	0.000	1.11	0.109	1.18	0.050
1455834_x_at	transforming, acidic coiled-coil containing protein 3	Tacc3	1.44	0.005	1.46	0.002	1.15	0.217
1436994_a_at	histone 1, H1c	Hist1h1c	1.44	0.000	-1.21	0.011	1.06	0.328
1439388_s_at	breast cancer anti-estrogen resistance 1	Bcar1	1.44	0.001	1.23	0.036	1.49	0.001
1456655_at	Exostoses (multiple) 1	Ext1	1.44	0.010	1.26	0.085	-1.11	0.547
1457154_at	Membrane-associated ring finger (C3HC4) 6	F830029L24Rik	1.44	0.003	1.14	0.163	1.05	0.613
1427838_at	tubulin, beta 2	Tubb2	1.44	0.000	1.08	0.421	-1.09	0.267
1434585_at	Tubby like protein 4	Tulp4	1.43	0.001	1.20	0.081	1.44	0.001
1428156_at	guanine nucleotide binding protein (G protein), gamma 2 subunit	Gng2	1.43	0.001	1.13	0.171	1.14	0.173
1449409_at	sulfotransferase family, cytosolic, 1C, member 2	Sult1c2	1.43	0.001	1.39	0.009	1.58	0.000
1437274_at	coatomer protein complex subunit alpha	Copa	1.43	0.002	-1.31	0.125	-1.55	0.019
1426853_at	SET translocation	Set	1.43	0.000	1.17	0.056	1.15	0.083
1423605_a_at	transformed mouse 3T3 cell double minute 2	Mdm2	1.43	0.001	1.15	0.132	1.09	0.477
1440020_at	Rearranged L-myc fusion sequence	Rlf	1.43	0.001	1.08	0.514	1.26	0.032
1436740_at	similar to p47 protein isoform a	LOC434492	1.43	0.008	1.24	0.124	1.29	0.107
1416138_at	annexin A7	Anxa7	1.43	0.001	1.09	0.325	1.09	0.326
1455029_at	kinesin family member 21A	Kif21a	1.43	0.000	-1.01	0.942	-1.10	0.285
1419027_s_at	glycolipid transfer protein	MGI:1929253	1.43	0.000	1.24	0.008	1.05	0.493
1428142_at	ets variant gene 5	Etv5	1.43	0.008	1.33	0.015	1.24	0.006
1456768_a_at	multimerin 2	Mmrn2	1.43	0.006	1.19	0.158	1.22	0.107
1449439_at	Kruppel-like factor 7 (ubiquitous)	Klf7	1.43	0.000	-1.02	0.742	1.00	0.992
1449127_at	selectin, platelet (p-selectin) ligand	Selpl	1.43	0.003	1.25	0.076	1.33	0.008
1416069_at	phosphofructokinase, platelet	Pfkp	1.43	0.001	1.22	0.074	1.22	0.100
1436499_at	transmembrane protein 23	Tmem23	1.43	0.000	-1.00	0.958	-1.06	0.459
1460341_at	pleckstrin homology domain containing, family B (evectins) member 2	Plekhb2	1.43	0.002	1.38	0.011	1.17	0.094
1441835_x_at	myotubularin related protein 11	Mtmr11	1.43	0.001	1.30	0.001	1.16	0.101
1424965_at	leupaxin	Lpxn	1.43	0.005	1.37	0.029	1.36	0.013
1418402_at	a disintegrin and metalloproteinase domain 19 (meltrin beta)	Adam19	1.42	0.004	1.30	0.004	1.18	0.059
1456042_s_at	T-complex expressed gene 4	Tce4	1.42	0.001	1.13	0.172	1.09	0.384

1420895_at	transforming growth factor, beta receptor I	Tgfbr1	1.42	0.000	1.18	0.001	1.11	0.006
1460419_a_at	protein kinase C, beta 1	Prkcb1	1.42	0.000	1.14	0.104	1.24	0.006
1445391_at	diaphanous homolog 1 (Drosophila)	Diap1	1.42	0.005	1.10	0.238	1.05	0.644
1448316_at	chemokine-like factor super family 3	Cklfsf3	1.42	0.002	1.33	0.008	1.32	0.016
1459906_at	Diacylglycerol kinase, eta	Dgkh	1.42	0.002	1.14	0.002	1.12	0.059
1436984_at	abl-interactor 2	Abi2	1.42	0.001	-1.02	0.826	1.02	0.844
1452410_a_at	feline sarcoma oncogene	Fes	1.42	0.003	1.14	0.267	1.52	0.001
1438370_x_at	downstream of Stk11	Dos	1.42	0.002	1.06	0.362	1.11	0.175
1447980_s_at	RUN and TBC1 domain containing 2	Rutbc2	1.42	0.006	1.24	0.081	1.19	0.084
1451969_s_at	poly (ADP-ribose) polymerase family, member 3	Parp3	1.42	0.000	1.24	0.047	1.18	0.057
1434058_at	phosphatidylinositol-3-phosphatase associated protein	Pip3ap	1.42	0.002	1.12	0.318	1.06	0.618
1454772_at	Activating signal cointegrator 1 complex subunit 3-like 1	Ascc3l1	1.42	0.008	1.12	0.350	1.23	0.082
1426594_at	FERM domain containing 4B	Frmd4b	1.42	0.007	1.23	0.168	1.18	0.216
1450241_a_at	ecotropic viral integration site 2a	Evi2a	1.42	0.003	1.58	0.004	1.30	0.017
1448770_a_at	ATPase inhibitory factor 1	Atpif1	1.42	0.001	1.20	0.045	1.15	0.136
1450764_at	acyloxyacyl hydrolase	Aoah	1.42	0.001	1.17	0.042	1.09	0.230
1446968_at	Phosphatidylinositol binding clathrin assembly protein	Picalm	1.42	0.000	1.08	0.398	1.00	0.964
1448180_a_at	hematological and neurological expressed sequence 1	Hn1	1.41	0.002	1.26	0.011	1.03	0.780
1434624_x_at	ribosomal protein S9	Rps9	1.41	0.002	1.05	0.719	1.01	0.967
1436779_at	cytochrome b-245, beta polypeptide	Cybb	1.41	0.004	1.31	0.052	1.20	0.106
1460437_at	pleckstrin homology, Sec7 and coiled/coil domains 4	Pscd4	1.41	0.000	1.04	0.491	1.07	0.204
1439079_a_at	Erbb2 interacting protein	Erbb2ip	1.41	0.005	1.05	0.698	-1.28	0.047
1440662_at	Ral guanine nucleotide dissociation stimulator,-like 1	Rgl1	1.41	0.004	1.09	0.257	1.07	0.311
1441056_at	Ubiquitin specific protease 3	Usp3	1.41	0.006	-1.00	0.985	1.05	0.713
1426946_at	RAN binding protein 5	Ranbp5	1.41	0.000	1.04	0.420	1.05	0.306
1427134_at	splicing factor, arginine/serine-rich 12	Sfrs12	1.41	0.000	1.27	0.004	1.22	0.004
1440576_at	carboxypeptidase N, polypeptide 1	Cpn1	1.41	0.004	1.25	0.038	1.24	0.083
1416635_at	sphingomyelin phosphodiesterase, acid-like 3A	Smpdl3a	1.41	0.000	1.29	0.005	1.30	0.001
1428512_at	basic helix-loop-helix domain containing, class B9	Bhlhb9	1.41	0.000	1.10	0.091	1.06	0.356
1432394_a_at	apoptosis antagonizing transcription factor	Aatf	1.41	0.001	1.01	0.857	1.14	0.042
1456251_x_at	benzodiazepine receptor, peripheral	Bzrp	1.41	0.007	1.10	0.450	1.13	0.321
1425142_a_at	heterogeneous nuclear ribonucleoprotein D	Hnrpd	1.41	0.001	1.06	0.583	-1.07	0.541
1451227_a_at	solute carrier family 10 (sodium/bile acid cotransporter family), member 3	Slc10a3	1.41	0.000	-1.11	0.198	1.03	0.734
1416335_at	macrophage migration inhibitory factor	Mif	1.41	0.000	1.08	0.329	1.19	0.022
1451286_s_at	fusion, derived from t(12;16) malignant liposarcoma (human)	Fus	1.40	0.000	-1.07	0.464	-1.08	0.455
1429376_s_at	anaphase promoting complex subunit 10	Anapc10	1.40	0.003	1.12	0.265	1.35	0.013
1435653_at	Abhydrolase domain containing 2	Abhd2	1.40	0.004	1.26	0.082	1.20	0.002
1455039_a_at	transcriptional regulator, SIN3B (yeast)	Sin3b	1.40	0.000	1.05	0.502	1.13	0.057
1460675_at	immunoglobulin superfamily, member 8	Igsf8	1.40	0.002	1.13	0.300	-1.10	0.303
1430520_at	copine VIII	Cpne8	1.40	0.009	1.01	0.888	1.14	0.206
1435375_at	triple functional domain (PTPRF interacting)	Trio	1.40	0.004	1.25	0.030	1.49	0.005

1417995_at	protein tyrosine phosphatase, non-receptor type 22 (lymphoid)	Ptpn22	1.40	0.008	1.08	0.184	1.05	0.522
1442365_at	Reticulon 3	Rtn3	1.40	0.003	1.06	0.602	-1.14	0.244
1448519_at	TEA domain family member 2	Tead2	1.40	0.003	1.12	0.368	1.09	0.414
1437234_x_at	Heterogeneous nuclear ribonucleoprotein methyltransferase-like 1 (S. cerevisiae)	Hrmt1l1	1.40	0.007	1.15	0.241	1.30	0.027
1433908_a_at	cortactin	Cttn	1.40	0.000	1.15	0.019	1.05	0.506
1449575_a_at	glutathione S-transferase, pi 1	Gstp1	1.40	0.000	1.07	0.242	1.07	0.218
1418438_at	fatty acid binding protein 2, intestinal	Fabp2	1.40	0.001	1.13	0.038	1.09	0.185
1419509_a_at	N-acetylglucosamine kinase	Nagk	1.40	0.001	1.13	0.117	1.25	0.004
1422286_a_at	TG interacting factor	Tgif	1.40	0.005	1.19	0.079	1.57	0.000
1454708_at	actin-binding LIM protein 1	Ablim1	1.40	0.000	-1.01	0.810	1.07	0.265
1419759_at	ATP-binding cassette, sub-family B (MDR/TAP), member 1A	Abcb1a	1.40	0.004	-1.11	0.380	1.03	0.764
1417177_at	galactokinase 1	Galk1	1.40	0.001	-1.10	0.160	-1.08	0.303
1427565_a_at	ATP-binding cassette, sub-family C (CFTR/MRP), member 5	Abcc5	1.40	0.001	1.26	0.212	1.23	0.059
1421346_a_at	solute carrier family 6 (neurotransmitter transporter, taurine), member 6	Slc6a6	1.40	0.001	-1.02	0.818	1.32	0.004
1426716_at	tudor domain containing 7	Tdrd7	1.40	0.005	1.39	0.005	1.34	0.002
1442298_at	Bicaudal C homolog 1 (Drosophila)	Bicc1	1.40	0.010	1.04	0.727	-1.01	0.893
1437226_x_at	MARCKS-like protein	Mlp	1.40	0.002	1.38	0.006	1.08	0.202
1443649_at	HECT, UBA and WWE domain containing 1	Ureb1	1.40	0.001	1.20	0.093	-1.08	0.257
1425553_s_at	huntingtin interacting protein 1 related	Hip1r	1.39	0.001	1.14	0.166	1.04	0.685
1426572_at	malic enzyme 2, NAD(+)-dependent, mitochondrial	Me2	1.39	0.000	1.47	0.000	1.42	0.001
1438127_at	BAT2 domain containing 1	1810043M20Rik	1.39	0.002	1.28	0.030	-1.09	0.442
1448142_x_at	ribosomal protein S13	Rps13	1.39	0.006	1.12	0.319	1.29	0.031
1441075_at	nitric oxide synthase trafficker	LOC329416	1.39	0.008	1.24	0.028	1.26	0.019
1429883_at	actin-like 6A	Actl6a	1.39	0.003	1.03	0.810	1.06	0.631
1449041_a_at	thyroid hormone receptor interactor 6	Trip6	1.39	0.000	-1.04	0.627	-1.01	0.882
1456236_s_at	COMM domain containing 10	Commd10	1.39	0.002	1.12	0.193	1.30	0.014
1416934_at	X-linked myotubular myopathy gene 1	Mtm1	1.39	0.008	-1.02	0.831	1.14	0.192
1420376_a_at	H3 histone, family 3B	H3f3b	1.39	0.000	1.11	0.053	-1.05	0.411
1425094_a_at	LIM homeobox protein 6	Lhx6	1.39	0.000	1.20	0.118	1.31	0.004
1448393_at	claudin 7	Cldn7	1.39	0.004	1.03	0.654	1.22	0.029
1416389_a_at	chromosome condensation 1-like	Chc1l	1.39	0.005	1.27	0.042	1.45	0.001
1436900_x_at	leptin receptor overlapping transcript	Leprot	1.39	0.000	1.08	0.273	-1.09	0.275
1449346_s_at	RIO kinase 1 (yeast)	Riok1	1.39	0.000	1.11	0.144	-1.04	0.647
1448318_at	adipose differentiation related protein	Adfp	1.39	0.000	1.22	0.013	1.30	0.000
1424894_at	RAB13, member RAS oncogene family	Rab13	1.39	0.000	1.05	0.595	1.11	0.132
1453191_at	procollagen, type XXVII, alpha 1	Col27a1	1.39	0.000	-1.01	0.934	-1.14	0.071
1417251_at	palmdelphin	Palmd	1.39	0.000	-1.11	0.169	1.19	0.054
1434450_s_at	Adrenergic receptor kinase, beta 2	Adrbk2	1.39	0.002	1.44	0.002	1.39	0.002
1460096_at	Lysosomal membrane glycoprotein 1	Lamp1	1.39	0.006	1.07	0.531	-1.15	0.184
1435057_x_at	RNA polymerase I associated factor	MGI:1929022	1.39	0.008	-1.09	0.364	1.27	0.012
1415670_at	coatomer protein complex, subunit gamma	Copg	1.39	0.000	1.16	0.085	1.20	0.008

1460586_at	EGF-like-domain, multiple 4	Egfl4	1.39	0.000	-1.00	0.973	1.10	0.151
1420760_s_at	N-myc downstream regulated-like	Ndrl	1.39	0.001	1.05	0.521	-1.07	0.463
1418390_at	PHD finger protein 21A	Phf21a	1.39	0.008	-1.07	0.467	-1.09	0.571
1424927_at	GLI pathogenesis-related 1 (glioma)	Glipr1	1.38	0.007	1.14	0.160	1.34	0.001
1452148_at	low density lipoprotein receptor-related protein associated protein 1	Lrpap1	1.38	0.000	1.08	0.190	1.30	0.000
1416150_a_at	splicing factor, arginine/serine-rich 3 (SRp20)	Sfrs3	1.38	0.010	1.17	0.188	1.13	0.306
1443954_at	RAD18 homolog (S. cerevisiae)	Rad18	1.38	0.002	1.22	0.018	-1.00	0.981
1449836_x_at	Bcl2-interacting killer-like	Biklk	1.38	0.007	1.43	0.024	1.44	0.012
1443158_at	Sex comb on midleg homolog 1	Scmh1	1.38	0.002	1.04	0.397	1.02	0.697
1455871_s_at	Tax1 (human T-cell leukemia virus type I) binding protein 3 /// ribosomal protein L13	Tax1bp3 /// Rpl13	1.38	0.001	1.05	0.470	1.05	0.422
1416026_a_at	ribosomal protein L12	Rpl12	1.38	0.000	1.06	0.440	1.27	0.005
1416303_at	LPS-induced TN factor	Litaf	1.38	0.000	1.17	0.042	1.08	0.186
1423543_at	SWA-70 protein	MGI:1298390	1.38	0.001	1.21	0.073	1.49	0.001
1419488_at	TNFAIP3 interacting protein 2	Tnip2	1.38	0.007	1.14	0.008	1.13	0.143
1437740_at	pleckstrin homology domain containing, family M (with RUN domain) member 2	Plekhm2	1.38	0.004	1.35	0.005	1.45	0.001
1434895_s_at	protein phosphatase 1, regulatory (inhibitor) subunit 13B	Ppp1r13b	1.38	0.002	1.15	0.141	1.19	0.083
1431708_a_at	cytotoxic granule-associated RNA binding protein 1	Tia1	1.38	0.000	1.19	0.069	1.13	0.258
1426859_at	inhibin beta-B	Inhbb	1.38	0.007	1.11	0.433	1.12	0.432
1416673_at	beta-site APP-cleaving enzyme 2	Bace2	1.38	0.002	1.24	0.034	1.15	0.170
1433956_at	cadherin 5	Cdh5	1.38	0.001	1.27	0.007	1.07	0.311
1451075_s_at	CTD small phosphatase 2	Ctdsp2	1.38	0.002	-1.01	0.937	-1.06	0.635
1441931_x_at	glutathione synthetase	Gss	1.38	0.005	-1.04	0.647	-1.00	0.965
1420703_at	colony stimulating factor 2 receptor, alpha, low-affinity (granulocyte-macrophage)	Csf2ra	1.38	0.006	1.44	0.006	1.39	0.004
1421392_a_at	baculoviral IAP repeat-containing 3	Birc3	1.38	0.006	1.15	0.157	1.14	0.197
1426914_at	MARVEL (membrane-associating) domain containing 2	Marveld2	1.38	0.003	1.19	0.102	1.33	0.002
1416453_x_at	ribosomal protein S12 /// similar to ribosomal protein S12	Rps12 /// LOC4348	1.38	0.000	1.01	0.903	1.14	0.031
1436193_at	mannosidase, alpha, class 1C, member 1	Man1c1	1.38	0.003	1.36	0.035	1.24	0.070
1436549_a_at	Heterogeneous nuclear ribonucleoprotein A1	Hnrpa1	1.38	0.008	-1.05	0.641	-1.10	0.312
1449404_at	phosphatidylinositol-4-phosphate 5-kinase, type II, alpha	Pip5k2a	1.38	0.000	1.00	0.979	1.06	0.380
1417403_at	ELOVL family member 6, elongation of long chain fatty acids (yeast)	Elovl6	1.37	0.001	1.09	0.070	1.18	0.033
1440536_at	Solute carrier family 22 (organic cation transporter), member 5	Slc22a5	1.37	0.001	1.02	0.831	-1.06	0.337
1457638_x_at	Replication factor C (activator 1) 2	Rfc2	1.37	0.001	-1.09	0.406	-1.02	0.832
1436822_x_at	ribosomal protein L17	Rpl17	1.37	0.001	-1.11	0.315	1.07	0.393
1419494_a_at	tumor protein D52	Tpd52	1.37	0.004	1.28	0.023	1.28	0.023
1455978_a_at	matrilin 2	Matn2	1.37	0.009	1.33	0.025	1.05	0.640
1448995_at	chemokine (C-X-C motif) ligand 4	Cxcl4	1.37	0.003	1.14	0.191	1.10	0.359
1426529_a_at	transgelin 2	Tagln2	1.37	0.002	1.40	0.007	1.24	0.014
1431771_a_at	interleukin-1 receptor-associated kinase 1 binding protein 1	Irak1bp1	1.37	0.000	1.21	0.005	1.13	0.059
1416257_at	calpain 2	Capn2	1.37	0.003	1.23	0.034	1.23	0.021
1416714_at	interferon consensus sequence binding protein 1	Icsbp1	1.37	0.001	1.33	0.010	1.26	0.008
1448591_at	cathepsin S	Ctss	1.37	0.006	1.35	0.009	1.24	0.049

1423854_a_at	RAS-like, family 11, member B	Rasl11b	1.37	0.002	1.13	0.323	1.10	0.370
1427941_at	Dicer1, Dcr-1 homolog (Drosophila)	Dicer1	1.37	0.000	1.06	0.458	1.10	0.191
1433512_at	Friend leukemia integration 1	Fli1	1.37	0.004	1.09	0.430	-1.05	0.643
1455319_x_at	ribosomal protein S8	Rps8	1.37	0.000	-1.05	0.472	1.02	0.728
1424495_a_at	chemokine-like factor	Cklf	1.37	0.002	1.44	0.017	1.29	0.000
1450882_s_at	transmembrane 7 superfamily member 1	Tm7sf1	1.37	0.000	1.18	0.045	1.13	0.140
1440285_at	protein phosphatase 1, regulatory (inhibitor) subunit 9A	Ppp1r9a	1.37	0.001	1.19	0.013	1.04	0.609
1424048_a_at	NAD(P)H:quinone oxidoreductase type 3, polypeptide A2	Nqo3a2	1.37	0.001	1.17	0.111	1.23	0.008
1460259_s_at	chloride channel calcium activated 1/2	Clca1/2	1.37	0.001	1.44	0.002	1.10	0.292
1420928_at	beta galactoside alpha 2,6 sialyltransferase 1	St6gal1	1.37	0.000	1.26	0.008	1.21	0.018
1456390_at	protein phosphatase 2a, catalytic subunit, alpha isoform	Ppp2ca	1.37	0.000	1.09	0.188	1.18	0.029
1418098_at	adenylate cyclase 4	Adcy4	1.37	0.007	1.13	0.242	1.44	0.002
1424950_at	SRY-box containing gene 9	Sox9	1.37	0.000	1.21	0.079	1.24	0.095
1430522_a_at	vesicle-associated membrane protein 5	Vamp5	1.37	0.003	1.17	0.084	1.32	0.002
1420477_at	nucleosome assembly protein 1-like 1	Nap1l1	1.37	0.002	1.08	0.341	1.08	0.328
1434518_at	phosphorylase kinase alpha 2	Phka2	1.37	0.002	1.03	0.572	1.07	0.147
1420664_s_at	protein C receptor, endothelial	Procr	1.37	0.000	-1.02	0.413	1.11	0.054
1423570_at	ATP-binding cassette, sub-family G (WHITE), member 1	Abcg1	1.37	0.000	1.21	0.006	-1.06	0.379
1450851_at	WD repeat domain 1	Wdr1	1.36	0.008	1.38	0.009	1.18	0.171
1433490_s_at	erythrocyte protein band 4.1-like 2	Epb4.1l2	1.36	0.000	1.30	0.017	1.21	0.017
1456397_at	cadherin 4	Cdh4	1.36	0.000	-1.01	0.921	1.02	0.783
1416528_at	SH3 domain binding glutamic acid-rich protein-like 3	Sh3bgrl3	1.36	0.000	1.16	0.050	-1.01	0.922
1416486_at	small inducible cytokine subfamily E, member 1	Scye1	1.36	0.000	1.11	0.156	1.25	0.006
1454870_x_at	G protein-coupled receptor 172B	Gpr172b	1.36	0.003	1.02	0.815	1.13	0.199
1416226_at	actin related protein 2/3 complex, subunit 1B	Arpc1b	1.36	0.001	1.20	0.267	1.97	0.001
1434525_at	protein kinase N3	Pkn3	1.36	0.005	1.22	0.033	1.27	0.036
1435645_at	monocyte to macrophage differentiation-associated	Mmd	1.36	0.003	1.06	0.507	1.13	0.168
1454788_at	ADP-ribosylation factor-like 7	Arl7	1.36	0.000	1.25	0.009	1.25	0.003
1455482_at	adaptor protein complex AP-2, alpha 2 subunit	Ap2a2	1.36	0.006	1.26	0.035	1.40	0.000
1447447_s_at	serine/arginine repetitive matrix 1	Srrm1	1.36	0.000	1.03	0.721	-1.01	0.852
1416499_a_at	dynactin 6	Dctn6	1.36	0.001	1.25	0.008	1.04	0.633
1424138_at	rhomboid family 1 (Drosophila)	Rhbdf1	1.36	0.000	1.10	0.283	1.09	0.349
1427891_at	GTPase, IMAP family member 6	Gimap6	1.36	0.000	1.03	0.629	1.16	0.031
1417077_at	B-cell receptor-associated protein 29	Bcap29	1.36	0.000	1.06	0.436	1.21	0.024
1416068_at	lysyl-tRNA synthetase	Kars	1.36	0.000	1.11	0.087	1.19	0.013
1453366_at	tudor and KH domain containing protein	Tdrkh	1.36	0.001	1.03	0.674	-1.01	0.855
1418110_a_at	inositol polyphosphate-5-phosphatase D	Inpp5d	1.36	0.001	1.36	0.001	1.14	0.163
1460701_a_at	mitochondrial ribosomal protein L52	Mrpl52	1.36	0.002	1.00	0.964	1.02	0.875
1460637_s_at	prefoldin 5	Pfdn5	1.36	0.000	1.03	0.538	1.29	0.000
1455019_x_at	Cytoskeleton-associated protein 4	Ckap4	1.36	0.005	1.24	0.008	1.03	0.677
1434070_at	jagged 1	Jag1	1.36	0.006	1.05	0.671	-1.05	0.702

1437380_x_at	Phosphogluconate dehydrogenase	Pgd	1.36	0.000	1.24	0.004	1.05	0.400
1434339_at	formin binding protein 1-like	Fnbp1l	1.36	0.000	1.09	0.085	1.01	0.715
1428260_at	spastic paraplegia 3A homolog (human)	Spg3a	1.36	0.003	1.07	0.106	1.13	0.010
1433481_at	FK506 binding protein 14	Fkbp14	1.36	0.003	1.12	0.190	1.11	0.201
1435517_x_at	v-ral simian leukemia viral oncogene homolog B (ras related)	Ralb	1.36	0.003	1.10	0.282	1.06	0.582
1448276_at	tetraspanin 4	Tspan4	1.36	0.002	1.04	0.575	1.14	0.094
1448130_at	farnesyl diphosphate farnesyl transferase 1	Fdft1	1.36	0.005	1.14	0.129	1.34	0.002
1454882_at	l(3)mbt-like 3 (Drosophila)	L3mbtl3	1.36	0.001	1.08	0.254	1.06	0.373
1418031_at	myosin IXb	Myo9b	1.35	0.001	1.15	0.131	1.28	0.009
1452237_at	HIV-1 Rev binding protein	Hrb	1.35	0.002	1.16	0.105	1.19	0.041
1433508_at	Kruppel-like factor 6	Copeb	1.35	0.004	1.15	0.145	1.16	0.106
1452833_at	Rap guanine nucleotide exchange factor (GEF) 2	Rapgef2	1.35	0.003	1.09	0.223	1.09	0.173
1419768_at	CD22 antigen	Cd22	1.35	0.003	1.48	0.013	1.15	0.226
1450719_at	meprin 1 alpha	Mep1a	1.35	0.002	1.03	0.685	-1.10	0.300
1450767_at	neural precursor cell expressed, developmentally down-regulated gene 9	Nedd9	1.35	0.002	1.00	#DIV/0!	1.16	0.003
1422906_at	ATP-binding cassette, sub-family G (WHITE), member 2	Abcg2	1.35	0.002	1.05	0.599	1.06	0.493
1452787_a_at	heterogeneous nuclear ribonucleoproteins methyltransferase-like 2 (S. cerevisiae)	Hrmt1l2	1.35	0.000	-1.49	0.024	-1.51	0.021
1417068_a_at	protein tyrosine phosphatase, non-receptor type 1	Ptpn1	1.35	0.005	-1.03	0.800	-1.21	0.140
1440816_x_at	DEAD (Asp-Glu-Ala-Asp) box polypeptide 1	Ddx1	1.35	0.003	1.00	0.947	-1.09	0.337
1422731_at	LIM domains containing 1	Limd1	1.35	0.000	1.00	0.973	1.07	0.468
1437148_at	actin related protein 2/3 complex, subunit 2	Arpc2	1.35	0.000	-1.01	0.860	-1.08	0.155
1455795_at	squamous cell carcinoma antigen recognized by T cells 2	Sart2	1.35	0.004	1.14	0.085	1.16	0.072
1417056_at	proteasome (prosome, macropain) 28 subunit, alpha	Psme1	1.35	0.001	1.20	0.022	1.13	0.040
1441989_at	BCL2/adenovirus E1B 19kDa-interacting protein 1, NIP2	Bnip2	1.35	0.004	1.06	0.432	1.01	0.920
1451318_a_at	Yamaguchi sarcoma viral (v-yes-1) oncogene homolog	Lyn	1.35	0.001	1.22	0.017	1.16	0.061
1423768_at	unc-93 homolog B1 (C. elegans)	Unc93b1	1.35	0.007	1.27	0.055	1.17	0.123
1460569_x_at	claudin 3	Cldn3	1.35	0.001	1.06	0.517	1.22	0.026
1429787_x_at	ZW10 interactor	Zwint	1.35	0.000	1.02	0.658	1.00	0.989
1423090_x_at	SEC61, gamma subunit	Sec61g	1.35	0.009	1.12	0.302	1.26	0.038
1441677_at	SMC4 structural maintenance of chromosomes 4-like 1 (yeast)	Smc4l1	1.35	0.008	1.08	0.599	1.06	0.684
1435903_at	CD300A antigen	Cd300a	1.35	0.009	1.26	0.031	1.24	0.055
1447754_x_at	THAP domain containing 4	Thap4	1.35	0.007	-1.04	0.628	1.05	0.647
1454714_x_at	3-phosphoglycerate dehydrogenase /	Phgdh /// LOC4342	1.34	0.006	1.23	0.064	1.23	0.074
1454890_at	angiomotin	Amot	1.34	0.009	1.05	0.645	1.07	0.509
1455950_x_at	ribosomal protein L35	Rpl35	1.34	0.000	1.02	0.756	1.07	0.285
1437565_a_at	guanine nucleotide binding protein-like 2 (nucleolar)	Gnl2	1.34	0.003	-1.11	0.141	-1.05	0.537
1424356_a_at	meteorin, glial cell differentiation regulator-like	Metrnl	1.34	0.003	1.05	0.644	1.40	0.001
1416273_at	tumor necrosis factor, alpha-induced protein 2	Tnfaip2	1.34	0.005	1.13	0.382	1.22	0.037
1434096_at	Solute carrier family 4 (anion exchanger), member 4	Slc4a4	1.34	0.001	1.22	0.070	1.26	0.027
1453686_x_at	nephronophthisis 1 (juvenile) homolog (human)	Nphp1	1.34	0.010	1.07	0.481	1.31	0.009
1439325_at	Bleomycin hydrolase	Blmh	1.34	0.000	1.01	0.864	1.16	0.049

1438091_a_at	H2A histone family, member Z	H2afz	1.34	0.005	1.21	0.018	1.08	0.327
1416194_at	cytochrome P450, family 4, subfamily b, polypeptide 1	Cyp4b1	1.34	0.006	1.19	0.077	1.26	0.078
1424650_at	protein disulfide isomerase-associated 5	Pdia5	1.34	0.002	1.11	0.231	1.14	0.117
1420361_at	solute carrier family 11 (proton-coupled divalent metal ion transporters), member 1	Slc11a1	1.34	0.004	1.21	0.047	1.38	0.001
1425059_at	HMT1 hnRNP methyltransferase-like 6 (S. cerevisiae)	Hrmt1l6	1.34	0.009	1.11	0.383	1.21	0.056
1426863_at	RNA binding motif protein, X chromosome	Rbmx	1.34	0.006	1.25	0.079	1.19	0.128
1427104_at	zinc finger protein 612	Zfp612	1.34	0.000	1.07	0.029	1.03	0.043
1434928_at	growth arrest-specific 2 like 1	Gas2l1	1.34	0.000	1.11	0.074	1.13	0.032
1416013_at	phospholipase D3	Pld3	1.34	0.001	1.13	0.211	1.09	0.186
1435342_at	potassium inwardly-rectifying channel, subfamily K, member 6	Kcnk6	1.34	0.001	1.10	0.102	-1.03	0.589
1452527_a_at	purinergic receptor P2X, ligand-gated ion channel 4	P2rx4	1.34	0.001	1.14	0.162	1.29	0.013
1455664_at	Reticulon 4 receptor-like 1	Rtn4rl1	1.34	0.001	-1.02	0.848	1.29	0.003
1460227_at	tissue inhibitor of metalloproteinase 1	Timp1	1.34	0.000	-1.03	0.444	-1.00	1.000
1423316_at	transmembrane protein 39a	Tmem39a	1.34	0.007	1.30	0.123	1.16	0.072
1438848_at	Oxysterol binding protein	Osbp	1.34	0.004	1.03	0.617	-1.03	0.636
1430781_at	adenylate kinase 7	Ak7	1.34	0.003	1.00	#DIV/0!	1.00	0.098
1449134_s_at	Spi-C transcription factor (Spi-1/PU.1 related)	Spic	1.34	0.000	1.05	0.320	1.10	0.059
1455009_at	carboxypeptidase D	Cpd	1.34	0.003	1.10	0.175	-1.05	0.493
1452973_at	protein phosphatase 1K (PP2C domain containing)	Ppm1k	1.33	0.002	1.08	0.394	1.20	0.021
1452422_a_at	U2 small nuclear ribonucleoprotein B	Snrpb2	1.33	0.007	1.09	0.409	1.28	0.011
1428172_at	PRP39 pre-mRNA processing factor 39 homolog (yeast)	Prpf39	1.33	0.004	1.10	0.173	1.02	0.735
1436680_s_at	damage specific DNA binding protein 2	Ddb2	1.33	0.005	1.19	0.041	1.49	0.000
1422763_at	regulator of G-protein signaling 19 interacting protein 1	Rgs19ip1	1.33	0.000	1.17	0.036	1.36	0.000
1418747_at	SFFV proviral integration 1	Sfpi1	1.33	0.009	1.32	0.045	1.35	0.017
1450731_s_at	tumor necrosis factor receptor superfamily, member 21	Tnfrsf21	1.33	0.001	1.35	0.010	1.15	0.206
1452740_at	myosin, heavy polypeptide 10, non-muscle	Myh10	1.33	0.006	1.24	0.043	1.14	0.144
1454995_at	dimethylarginine dimethylaminohydrolase 1	Ddah1	1.33	0.002	1.01	0.871	1.05	0.465
1436598_at	inducible T-cell co-stimulator	Icos	1.33	0.002	1.36	0.000	1.38	0.000
1427196_at	protein kinase, lysine deficient 4	Prkwnk4	1.33	0.001	1.00	0.638	-1.00	0.566
1435155_at	cingulin	Cgn	1.33	0.003	1.41	0.011	1.44	0.000
1452169_a_at	diacylglycerol kinase zeta	Dgkz	1.33	0.002	1.08	0.277	1.05	0.557
1422106_a_at	gene rich cluster, C9 gene	Grcc9	1.33	0.004	1.16	0.039	1.25	0.005
1449278_at	eukaryotic translation initiation factor 2 alpha kinase 3	Eif2ak3	1.33	0.002	1.15	0.077	1.06	0.432
1421654_a_at	lamin A	Lmna	1.33	0.002	-1.09	0.288	1.06	0.515
1448632_at	proteasome (prosome, macropain) subunit, beta type 10	Psmb10	1.33	0.006	1.19	0.056	1.12	0.141
1448145_at	WW domain containing E3 ubiquitin protein ligase 2	Wwp2	1.33	0.006	1.11	0.226	1.08	0.452
1415699_a_at	G protein pathway suppressor 1	Gps1	1.33	0.006	1.04	0.616	1.12	0.112
1457664_x_at	Complement component 2 (within H-2S)	C2	1.33	0.004	1.25	0.024	1.22	0.045
1428270_at	glycosyltransferase 8 domain containing 1	Glt8d1	1.33	0.001	1.12	0.157	1.06	0.342
1426852_x_at	nephroblastoma overexpressed gene	Nov	1.33	0.003	1.09	0.389	1.04	0.674
1456175_a_at	coatomer protein complex, subunit beta 2 (beta prime)	Copb2	1.33	0.000	1.15	0.013	1.15	0.055

1450921_at	aprataxin	Aptx	1.33	0.001	1.22	0.039	1.24	0.019
1436121_a_at	non-SMC element 1 homolog (S. cerevisiae)	Nsmce1	1.33	0.009	1.25	0.034	1.31	0.016
1436062_at	archain 1	Arcn1	1.33	0.009	1.13	0.223	1.12	0.290
1455534_s_at	oxysterol binding protein-like 11	Osbpl11	1.33	0.004	-1.04	0.656	-1.18	0.102
1422608_at	cAMP-regulated phosphoprotein 19	Arpp19	1.33	0.003	1.02	0.745	1.00	0.974
1447234_s_at	sorting nexin 6	Snx6	1.33	0.001	1.22	0.008	1.14	0.097
1434833_at	mitogen activated protein kinase kinase kinase kinase 2	Map4k2	1.33	0.001	1.26	0.000	1.07	0.168
1435791_x_at	ribosomal protein L17	Rpl17 /// LOC43530	1.32	0.001	-1.04	0.610	1.12	0.099
1422993_s_at	THO complex 4	Thoc4	1.32	0.008	1.17	0.064	1.18	0.038
1460004_x_at	syntaxin 6	Stx6	1.32	0.001	1.06	0.398	1.11	0.180
1433893_s_at	sperm associated antigen 5	Spag5	1.32	0.006	1.23	0.036	1.12	0.177
1426799_at	RAB8B, member RAS oncogene family	Rab8b	1.32	0.002	1.18	0.013	1.08	0.238
1418396_at	G-protein signalling modulator 3 (AGS3-like, C. elegans)	Gpsm3	1.32	0.002	1.24	0.021	1.18	0.046
1449353_at	wild-type p53-induced gene 1	Wig1	1.32	0.004	1.23	0.091	1.34	0.002
1453228_at	syntaxin 11	Stx11	1.32	0.001	1.12	0.134	-1.12	0.072
1422638_s_at	Ras association (RalGDS/AF-6) domain family 5	Rassf5	1.32	0.005	1.06	0.493	1.01	0.931
1453022_at	GPI-anchored HDL-binding protein 1	MGI:1915703	1.32	0.000	1.15	0.059	1.03	0.696
1425784_a_at	olfactomedin 1	Olfm1	1.32	0.005	-1.02	0.784	1.12	0.151
1455434_a_at	kinectin 1	Ktn1	1.32	0.000	-1.05	0.460	1.01	0.851
1460175_at	ribosomal protein S23	Rps23	1.32	0.000	1.06	0.180	1.14	0.003
1451804_a_at	leucine rich repeat containing 16	Lrrc16	1.32	0.003	1.20	0.025	1.05	0.435
1444073_at	avian musculoaponeurotic fibrosarcoma (v-maf) AS42 oncogene homolog	Maf	1.32	0.004	1.44	0.011	1.34	0.014
1453197_at	mediator of RNA polymerase II transcription, subunit 25 homolog (yeast)	Med25	1.32	0.002	1.00	0.980	1.04	0.662
1417785_at	phospholipase A1 member A	Pla1a	1.32	0.004	1.32	0.004	1.27	0.006
1455364_a_at	ribosomal protein S7	Rps7	1.32	0.000	1.07	0.262	1.22	0.010
1420171_s_at	myosin, heavy polypeptide 9, non-muscle	Myh9	1.32	0.001	1.12	0.150	1.07	0.258
1422860_at	neurotensin	Nts	1.32	0.006	1.00	#DIV/0!	1.00	#DIV/0!
1451142_at	SEC3-like 1 (S. cerevisiae)	Sec3l1	1.32	0.009	1.25	0.040	1.38	0.009
1448405_a_at	CREBBP/EP300 inhibitory protein 1	Cri1	1.32	0.003	-1.08	0.329	-1.09	0.253
1422768_at	synaptotagmin binding, cytoplasmic RNA interacting protein	Syncrip	1.32	0.001	-1.05	0.444	-1.03	0.638
1434285_at	FERM domain containing 4A	Frmd4a	1.32	0.009	1.29	0.007	-1.03	0.638
1439557_s_at	LIM domain binding 2	Ldb2	1.32	0.008	1.23	0.017	1.06	0.593
1450626_at	mannosidase, beta A, lysosomal	Manba	1.32	0.005	1.16	0.055	1.08	0.383
1433770_at	dihydropyrimidinase-like 2	Dpysl2	1.32	0.001	1.17	0.066	1.15	0.069
1434654_at	component of oligomeric golgi complex 3	Cog3	1.31	0.002	1.07	0.386	1.14	0.109
1457228_x_at	GLE1 RNA export mediator-like (yeast	Gle1l	1.31	0.001	1.04	0.516	-1.02	0.566
1455297_at	spindlin-like	Spin2	1.31	0.000	1.07	0.370	1.32	0.003
1420609_at	membrane-associated ring finger (C3HC4) 7	7-Mar	1.31	0.000	-1.03	0.622	-1.00	0.970
1460188_at	hemopoietic cell phosphatase	Hcph	1.31	0.008	1.29	0.028	1.36	0.007
1451555_at	neurolysin (metallopeptidase M3 family)	Nln	1.31	0.005	1.00	0.957	1.13	0.129
1419107_at	oligophrenin 1	Ophn1	1.31	0.007	1.03	0.781	1.05	0.597

1452280_at	FERM, RhoGEF (Arhgef) and pleckstrin domain protein 1 (chondrocyte-derived)	Farp1	1.31	0.000	1.21	0.027	1.03	0.640
1454018_at	tousled-like kinase 2 (Arabidopsis)	Tlk2	1.31	0.002	1.01	0.855	1.03	0.644
1416520_x_at	ribosomal protein L36	Rpl36	1.31	0.003	-1.05	0.565	1.06	0.493
1451573_a_at	syntaxin 4A (placental)	Stx4a	1.31	0.000	1.16	0.067	1.07	0.174
1442103_at	Nipped-B homolog (Drosophila)	Nipbl	1.31	0.008	-1.07	0.571	-1.16	0.257
1427193_at	bromodomain containing 8	Brd8	1.31	0.007	1.39	0.011	1.08	0.374
1449254_at	secreted phosphoprotein 1	Spp1	1.31	0.008	1.05	0.546	1.02	0.848
1460198_a_at	proteasome (prosome, macropain) subunit, beta type 3	Psmb3	1.31	0.008	1.02	0.855	1.01	0.896
1429453_a_at	mitochondrial ribosomal protein L55	Mrpl55	1.31	0.006	1.10	0.254	1.41	0.001
1417138_s_at	polymerase (RNA) II (DNA directed) polypeptide E	Polr2e	1.31	0.006	1.12	0.225	1.39	0.001
1442959_at	baculoviral IAP repeat-containing 6	Birc6	1.31	0.000	1.04	0.098	1.00	#DIV/0!
1447838_x_at	echinoderm microtubule associated protein like 4	Eml4	1.31	0.005	1.04	0.420	-1.05	0.317
1418458_at	anaphase promoting complex subunit 7	Anapc7	1.31	0.004	-1.10	0.329	1.09	0.254
1452985_at	uveal autoantigen with coiled-coil domains and ankyrin repeats	Uaca	1.31	0.003	1.02	0.811	1.19	0.043
1444578_at	Speckle-type POZ protein	Spop	1.31	0.000	1.00	#DIV/0!	1.02	0.098
1451135_at	general transcription factor IIB	Gtf2b	1.31	0.001	1.02	0.787	1.08	0.412
1458910_at	Transforming growth factor beta regulated gene 3	Tbrg3	1.31	0.000	1.38	0.005	1.11	0.025
1416837_at	Bcl2-associated X protein	Bax	1.31	0.001	1.03	0.534	1.08	0.083
1452759_s_at	PTPRF interacting protein, binding protein 1 (liprin beta 1)	Ppfibp1	1.31	0.000	1.01	0.867	-1.02	0.620
1422896_at	vesicle-associated membrane protein 4	Vamp4	1.31	0.000	1.22	0.017	1.06	0.212
1437924_at	Son cell proliferation protein	Son	1.31	0.007	1.06	0.535	1.02	0.823
1447241_at	H2A histone family, member Y3	H2afy3	1.31	0.009	1.06	0.390	1.18	0.085
1416212_at	mago-nashi homolog, proliferation-associated (Drosophila)	Magoh	1.31	0.001	1.17	0.052	1.29	0.003
1420514_at	transmembrane protein 47	Tmem47	1.31	0.003	-1.06	0.364	-1.19	0.045
1420635_a_at	T-cell, immune regulator 1	Tcirg1	1.30	0.004	1.41	0.003	1.14	0.133
1416935_at	transient receptor potential cation channel, subfamily V, member 2	Trpv2	1.30	0.002	1.23	0.002	1.13	0.188
1456113_at	ornithine aminotransferase-like 1	Oatl1	1.30	0.009	1.18	0.148	-1.01	0.894
1435003_at	phosphatidylinositol 4-kinase, catalytic, alpha polypeptide	Pik4ca	1.30	0.001	-1.06	0.466	-1.01	0.917
1424375_s_at	GTPase, IMAP family member 4	Gimap4	1.30	0.001	1.21	0.001	1.04	0.435
1426397_at	transforming growth factor, beta receptor II	Tgfbr2	1.30	0.001	1.11	0.203	1.09	0.181
1417100_at	CD320 antigen	Cd320	1.30	0.009	-1.05	0.583	1.04	0.708
1460231_at	interferon regulatory factor 5	Irf5	1.30	0.008	1.30	0.009	1.44	0.005
1417189_at	proteasome (prosome, macropain) 28 subunit, beta	Psme2	1.30	0.006	1.21	0.017	1.16	0.064
1420091_s_at	zinc finger, CW-type with coiled-coil domain 3	Zcwcc3	1.30	0.001	1.25	0.001	1.00	0.969
1448819_at	eukaryotic translation initiation factor 2, subunit 2 (beta)	Eif2s2	1.30	0.003	1.09	0.032	1.16	0.002
1437151_at	ubiquitin specific protease 22	Usp22	1.30	0.002	1.24	0.000	1.20	0.001
1424089_a_at	transcription factor 4	Tcf4	1.30	0.002	1.23	0.023	1.24	0.037
1448736_a_at	hypoxanthine guanine phosphoribosyl transferase 1	Hprt1	1.30	0.002	1.07	0.312	1.20	0.020
1416457_at	dimethylarginine dimethylaminohydrolase 2	Ddah2	1.30	0.004	1.23	0.043	1.03	0.708
1416770_at	serine/threonine kinase 25 (yeast)	Stk25	1.30	0.005	1.24	0.034	1.32	0.004
1454805_at	Wilms' tumour 1-associating protein	Wtap	1.30	0.000	1.14	0.042	1.21	0.024

1450107_a_at	renin binding protein	Renbp	1.30	0.003	1.20	0.084	1.24	0.023
1451643_a_at	RAB4B, member RAS oncogene family	Rab4b	1.30	0.007	1.16	0.130	1.19	0.043
1433651_at	WT1-interacting protein	Wtip	1.30	0.001	1.19	0.048	1.14	0.125
1449734_s_at	Bardet-Biedl syndrome 4 homolog (human)	Bbs4	1.29	0.009	1.15	0.135	1.02	0.777
1449243_a_at	ribosomal protein S19	Rps19	1.29	0.005	-1.08	0.349	1.06	0.453
1458679_a_at	TatD DNase domain containing 1	Tatdn1	1.29	0.004	1.04	0.609	1.14	0.056
1438951_x_at	nucleoporin 54	Nup54	1.29	0.003	1.06	0.492	-1.02	0.795
1460001_at	pyroglutamyl-peptidase I	Pgpep1	1.29	0.007	1.02	0.815	1.19	0.057
1423332_at	syndecan binding protein	Sdcbp	1.29	0.002	1.21	0.030	1.14	0.084
1437379_x_at	TNF receptor-associated protein 1	Trap1	1.29	0.009	1.13	0.258	1.25	0.067
1437649_x_at	peptidylprolyl isomerase B	Ppib	1.29	0.006	1.09	0.117	1.07	0.312
1433929_at	NHL repeat containing 2	Nhlrc2	1.29	0.000	1.10	0.032	-1.01	0.871
1444659_at	Thyroid hormone receptor interactor 11	6030460N08Rik	1.29	0.000	1.05	0.098	1.00	#DIV/0!
1438459_x_at	splicing factor proline/glutamine rich	Sfpq	1.29	0.008	-1.02	0.847	-1.17	0.148
1454992_at	solute carrier family 7 (cationic amino acid transporter, y+ system), member 1	Slc7a1	1.29	0.002	-1.05	0.548	-1.02	0.768
1448967_at	nipsnap homolog 3A (C. elegans)	Nipsnap3a	1.29	0.004	1.11	0.162	1.07	0.394
1424345_s_at	ubiquitin-conjugating enzyme E2M (UBC12 homolog, yeast)	Ube2m	1.29	0.002	1.14	0.069	1.11	0.192
1435187_at	Translocase of outer mitochondrial membrane 20 homolog (yeast)	Tomm20	1.29	0.001	1.04	0.473	1.02	0.708
1449407_at	carnitine deficiency-associated gene expressed in ventricle 1	Cdv1	1.29	0.004	1.11	0.275	1.13	0.019
1450069_a_at	CUG triplet repeat,RNA binding protein 2	Cugbp2	1.29	0.001	1.21	0.006	1.11	0.128
1416791_a_at	nuclear RNA export factor 1 homolog (S. cerevisiae)	Nxf1	1.29	0.003	1.23	0.016	1.11	0.212
1428585_at	actinin, alpha 1	Actn1	1.29	0.001	1.25	0.000	1.12	0.017
1421729_a_at	fer (fms/fps related) protein kinase, testis specific 2	Fert2	1.29	0.009	1.18	0.044	1.17	0.046
1428319_at	PDZ and LIM domain 7	Pdlim7	1.29	0.005	1.15	0.071	-1.00	0.987
1419526_at	Gardner-Rasheed feline sarcoma viral (Fgr) oncogene homolog	Fgr	1.29	0.002	1.14	0.145	-1.02	0.755
1432108_at	ring finger protein 134	Rnf134	1.29	0.007	-1.04	0.722	-1.26	0.030
1423753_at	BMP and activin membrane-bound inhibitor, homolog (Xenopus laevis)	Bambi	1.29	0.006	1.09	0.427	1.15	0.149
1448592_at	cartilage associated protein	Crtap	1.29	0.000	1.28	0.000	1.11	0.078
1445589_at	Solute carrier family 23 (nucleobase transporters), member 2	Slc23a2	1.29	0.000	1.13	0.121	1.27	0.000
1416228_at	protein (peptidyl-prolyl cis/trans isomerase) NIMA-interacting 1	Pin1	1.29	0.010	1.15	0.124	1.14	0.162
1416842_at	glutathione S-transferase, mu 5	Gstm5	1.29	0.000	-1.02	0.621	1.02	0.585
1427578_a_at	integrin beta 4 binding protein	Itgb4bp	1.29	0.000	1.10	0.071	1.01	0.907
1438932_at	Similar to calcium and DAG-regulated guanine nucleotide exchange factor I	LOC381240	1.28	0.005	1.19	0.026	1.02	0.782
1460359_at	armadillo repeat containing, X-linked 3	Armcx3	1.28	0.006	1.15	0.037	1.10	0.124
1428315_at	EBNA1 binding protein 2	Ebna1bp2	1.28	0.004	1.09	0.276	1.28	0.008
1416115_at	origin recognition complex, subunit 3-like (S. cerevisiae)	Orc3l	1.28	0.000	1.13	0.119	1.34	0.001
1434310_at	bone morphogenic protein receptor, type II (serine/threonine kinase)	Bmpr2	1.28	0.007	1.02	0.820	1.00	0.978
1428819_at	microtubule-associated protein, RP/EB family, member 1	Mapre1	1.28	0.001	1.12	0.073	1.07	0.338
1416189_a_at	Sec61 alpha 1 subunit (S. cerevisiae)	Sec61a1	1.28	0.002	1.06	0.305	1.01	0.890
1449094_at	gap junction membrane channel protein alpha 7	Gja7	1.28	0.002	1.23	0.021	1.17	0.036
1423052_at	ADP-ribosylation factor 4	Arf4	1.28	0.008	1.10	0.149	1.01	0.886

1448199_at	ankyrin repeat domain 10	Ankrd10	1.28	0.003	1.29	0.000	1.13	0.011
1441477_at	calumenin	Calu	1.28	0.003	1.13	0.214	-1.04	0.572
1455994_x_at	elongation of very long chain fatty acids-like 1	Elovl1	1.28	0.001	1.22	0.015	1.24	0.005
1416146_at	heat shock protein 4	Hspa4	1.28	0.007	-1.01	0.880	1.07	0.282
1426410_at	pyruvate dehydrogenase kinase, isoenzyme 3	Pdk3	1.28	0.003	1.05	0.131	1.02	0.178
1427245_at	ADP-ribosylation factor GTPase activating protein 1	Arfgap1	1.28	0.001	1.11	0.129	1.01	0.915
1451628_a_at	ankyrin 3, epithelial	Ank3	1.28	0.008	-1.26	0.031	1.26	0.013
1455815_a_at	tyrosine 3-mono/ase/tryptophan 5-monooxygenase activation protein, b polypeptide	Ywhab	1.28	0.000	1.33	0.020	1.28	0.003
1456497_x_at	ribosomal protein S10	Rps10	1.28	0.000	-1.02	0.748	1.01	0.790
1455816_a_at	potassium channel tetramerisation domain containing 3	Kctd3	1.28	0.002	1.01	0.899	-1.11	0.222
1442080_at	CAMP responsive element binding protein 3-like 2	Creb3l2	1.28	0.003	-1.04	0.337	-1.05	0.287
1434487_at	myocyte enhancer factor 2D	Mef2d	1.28	0.003	-1.01	0.883	-1.03	0.741
1448846_a_at	ribosomal protein L29	Rpl29 /// LOC24036	1.28	0.000	1.05	0.388	1.07	0.308
1437706_x_at	ribosomal protein S14	Rps14	1.28	0.003	1.06	0.409	1.11	0.143
1428364_at	sodium channel modifier 1	Scnm1	1.28	0.010	1.28	0.017	1.38	0.002
1422516_a_at	fibroblast growth factor (acidic) intracellular binding protein	Fibp	1.28	0.003	1.12	0.082	1.13	0.098
1418296_at	FXYD domain-containing ion transport regulator 5	Fxyd5	1.28	0.000	1.13	0.032	-1.08	0.181
1435376_at	DDHD domain containing 2	Ddhd2	1.28	0.005	-1.03	0.726	1.05	0.566
1433495_at	glycosyltransferase 25 domain containing 1	Glt25d1	1.28	0.001	1.09	0.162	1.18	0.020
1427689_a_at	TNFAIP3 interacting protein 1	Tnip1	1.28	0.002	1.03	0.654	1.00	0.948
1436507_at	interleukin-1 receptor-associated kinase 2	Irak2	1.28	0.008	1.24	0.085	1.40	0.005
1452059_at	solute carrier family 35, member F5	Slc35f5	1.28	0.000	1.14	0.028	1.21	0.001
1431086_s_at	Protein-L-isoaspartate (D-aspartate) O-methyltransferase 1	Pcmt1	1.27	0.001	1.05	0.482	1.02	0.822
1459942_at	Open reading frame 34	ORF34	1.27	0.001	1.30	0.001	-1.02	0.715
1417328_at	excision repair cross-complementing rodent repair deficiency, compl. group 1	Ercc1	1.27	0.001	1.17	0.032	1.21	0.016
1452648_at	transforming growth factor beta regulated gene 1	Tbrg1	1.27	0.000	1.04	0.421	-1.03	0.514
1454627_a_at	ribosomal protein L29	Rpl29 /// LOC24036	1.27	0.000	1.01	0.867	-1.04	0.406
1421891_at	ST3 beta-galactoside alpha-2,3-sialyltransferase 2	St3gal2	1.27	0.001	1.17	0.064	1.21	0.071
1417501_at	F-box only protein 6b	Fbxo6b	1.27	0.001	1.21	0.009	1.30	0.015
1416529_at	epithelial membrane protein 1	Emp1	1.27	0.009	1.24	0.043	-1.03	0.681
1443783_x_at	Histocompatibility 2, class II antigen A, alpha	H2-Aa	1.27	0.009	1.00	#DIV/0!	1.00	#DIV/0!
1419938_s_at	Rho guanine nucleotide exchange factor (GEF) 17	Arhgef17	1.27	0.006	1.09	0.255	1.14	0.140
1418539_a_at	protein tyrosine phosphatase, receptor type, E	Ptpre	1.27	0.001	-1.01	0.396	-1.00	0.820
1419096_at	apolipoprotein M	Apom	1.27	0.000	1.10	0.091	-1.02	0.699
1435525_at	potassium channel tetramerisation domain containing 17	Kctd17	1.27	0.004	1.16	0.035	1.05	0.526
1438229_at	protein geranylgeranyltransferase type I, beta subunit	Pggt1b	1.27	0.000	1.05	0.343	1.14	0.031
1453957_a_at	insulin-like growth factor 2, binding protein 3	Igf2bp3	1.27	0.008	-1.01	0.738	1.03	0.556
1416508_at	mediator of RNA polymerase II transcription, subunit 28 homolog (yeast)	Med28	1.27	0.000	1.08	0.107	1.06	0.216
1416471_at	choline/ethanolaminephosphotransferase 1	Cept1	1.27	0.004	1.06	0.343	1.20	0.020
1427269_at	splicing factor, arginine/serine-rich 11	Sfrs11	1.27	0.001	1.09	0.135	1.08	0.160
1419128_at	integrin alpha X	Itgax	1.27	0.008	1.15	0.220	1.11	0.272

1434316_at	carbohydrate (chondroitin) synthase 1	Chsy1	1.27	0.009	1.26	0.008	1.08	0.349
1416928_at	RNA binding motif protein 12	Rbm12	1.27	0.004	1.18	0.053	1.21	0.037
1450914_at	protein phosphatase 1, regulatory (inhibitor) subunit 14B	Ppp1r14b	1.27	0.003	1.03	0.673	1.14	0.132
1428847_a_at	microtubule-actin crosslinking factor 1	Macf1	1.27	0.001	1.07	0.264	1.01	0.883
1455611_at	protein inhibitor of activated STAT 1	Pias1	1.27	0.008	1.24	0.019	1.20	0.050
1440320_at	Nuclear respiratory factor 1	Nrf1	1.27	0.001	1.14	0.077	-1.05	0.400
1420196_s_at	TBC1 domain family, member 14	Tbc1d14	1.27	0.000	1.11	0.026	1.08	0.076
1423687_a_at	mannosidase, alpha, class 2C, member 1	Man2c1	1.27	0.002	-1.00	0.989	1.01	0.865
1424018_at	histidine triad nucleotide binding protein 1	Hint1	1.27	0.001	1.08	0.214	1.12	0.071
1417269_at	cyclin-dependent kinase 9 (CDC2-related kinase)	Cdk9	1.27	0.001	1.00	0.939	-1.03	0.598
1456424_s_at	phospholipid transfer protein	Pltp	1.27	0.010	1.07	0.458	-1.10	0.306
1423119_at	radial spokehead-like 2	Rshl2	1.26	0.001	1.10	0.151	1.18	0.009
1448128_at	protective protein for beta-galactosidase	Ppgb	1.26	0.002	1.07	0.411	1.12	0.175
1418069_at	apolipoprotein C-II	Apoc2	1.26	0.001	1.04	0.402	1.05	0.294
1418029_at	Fas apoptotic inhibitory molecule	Faim	1.26	0.002	1.17	0.013	1.28	0.004
1417914_at	RAP2B, member of RAS oncogene family	Rap2b	1.26	0.002	1.05	0.561	1.05	0.440
1423805_at	disabled homolog 2 (Drosophila)	Dab2	1.26	0.005	1.15	0.032	1.10	0.131
1427043_s_at	cytosolic ovarian carcinoma antigen 1	Cova1	1.26	0.004	1.19	0.039	1.15	0.075
1456250_x_at	transforming growth factor, beta induced	Tgfbi	1.26	0.003	1.17	0.039	-1.02	0.760
1444506_at	Translocating chain-associating membrane protein 1	Tram1	1.26	0.008	-1.01	0.906	-1.12	0.317
1437172_x_at	hydr/cyl-CoA dehydr/ase/3-ketoacyl-CoA thiolase/enoyl-CoA hydratase b subunit	Hadhb	1.26	0.003	-1.06	0.330	-1.13	0.082
1417040_a_at	Bcl-2-related ovarian killer protein	Bok	1.26	0.003	1.21	0.009	1.17	0.025
1426794_at	protein tyrosine phosphatase, receptor type, S	Ptprs	1.26	0.000	1.18	0.053	1.11	0.132
1417242_at	DEAD (Asp-Glu-Ala-Asp) box polypeptide 48	Ddx48	1.26	0.002	1.17	0.028	1.11	0.044
1425419_a_at	v-raf-1 leukemia viral oncogene 1	Raf1	1.26	0.001	1.09	0.133	1.09	0.117
1452674_a_at	eukaryotic translation initiation factor 3, subunit 12	Eif3s12	1.26	0.008	1.12	0.187	1.22	0.035
1458802_at	human immunodeficiency virus type I enhancer binding protein 3	Hivep3	1.26	0.003	1.03	0.561	1.18	0.073
1425048_a_at	high mobility group box 1	Hmgb1	1.26	0.006	1.14	0.087	1.15	0.016
1426913_at	lanosterol synthase	Lss	1.26	0.002	1.16	0.023	-1.03	0.553
1422506_a_at	cystatin B	Cstb	1.26	0.001	-1.01	0.861	-1.01	0.832
1418243_at	ficolin A	Fcna	1.26	0.005	1.45	0.003	1.35	0.004
1433754_at	muscleblind-like 2	Mbnl2	1.26	0.004	1.11	0.127	-1.01	0.941
1423600_a_at	Treacher Collins Franceschetti syndrome 1, homolog	Tcof1	1.26	0.006	1.24	0.000	1.25	0.000
1417317_s_at	ribosomal protein L35a /// RIKEN cDNA 4632409L19 gene	Rpl35a /// 4632409L	1.26	0.000	1.01	0.763	1.10	0.041
1432260_at	G protein-coupled receptor 39	Gpr39	1.25	0.001	1.13	0.090	1.20	0.004
1452377_at	myeloid/lymphoid or mixed-lineage leukemia	Mll	1.25	0.005	1.18	0.147	1.17	0.075
1423587_a_at	exosome component 10	Exosc10	1.25	0.002	1.11	0.081	-1.04	0.617
1448351_at	coronin, actin binding protein 1B	Coro1b	1.25	0.002	1.26	0.013	1.26	0.005
1453096_x_at	ribosomal protein L27	Rpl27	1.25	0.000	-1.05	0.337	-1.15	0.081
1453467_s_at	ribosomal protein S15a	Rps15a	1.25	0.008	-1.05	0.552	-1.01	0.879
1433654_at	meningioma expressed antigen 5 (hyaluronidase)	Mgea5	1.25	0.009	1.04	0.653	1.11	0.236

1418574_a_at	split hand/foot deleted gene 1	Shfdg1	1.25	0.007	-1.02	0.839	-1.08	0.397
1417252_at	5',3'-nucleotidase, cytosolic	Nt5c	1.25	0.010	1.02	0.832	1.16	0.089
1455556_at	Notch gene homolog 2 (Drosophila)	Notch2	1.25	0.000	1.09	0.156	1.06	0.237
1417848_at	glucocorticoid induced gene 1	MGI:2180715	1.25	0.007	1.01	0.915	-1.05	0.490
1426761_at	amine oxidase (flavin containing) domain 2	Aof2	1.25	0.001	-1.03	0.597	1.15	0.027
1423568_at	proteasome (prosome, macropain) subunit, alpha type 7	Psma7	1.25	0.003	-1.06	0.319	1.07	0.216
1416394_at	Bcl2-associated athanogene 1	Bag1	1.25	0.001	-1.03	0.685	-1.02	0.768
1449255_a_at	ribosomal protein L15	Rpl15	1.25	0.000	1.07	0.060	1.08	0.048
1440965_at	Phosphatidylinositol glycan, class L	Pigl	1.25	0.002	1.03	0.804	1.09	0.233
1458370_at	BMP2 inducible kinase	Bmp2k	1.25	0.007	1.06	0.430	1.04	0.597
1417684_at	THUMP domain containing 3	Thumpd3	1.25	0.001	-1.02	0.703	1.11	0.110
1424285_s_at	ADP-ribosylation factor-like 6 interacting protein 4	Arl6ip4	1.25	0.005	-1.01	0.937	-1.03	0.757
1433518_at	leucine carboxyl methyltransferase 2	Lcmt2	1.25	0.005	1.05	0.467	1.27	0.022
1415814_at	ATPase, H+ transporting, V1 subunit B, isoform 2	Atp6v1b2	1.25	0.005	1.22	0.013	1.12	0.119
1417891_at	T-complex expressed gene 1	Tce1	1.25	0.001	1.14	0.047	1.19	0.019
1423721_at	tropomyosin 1, alpha	Tpm1	1.25	0.004	1.20	0.059	1.18	0.012
1439485_at	zinc finger protein 608	Zfp608	1.25	0.004	1.11	0.313	1.13	0.118
1421547_at	lymphocyte antigen 78	Ly78	1.25	0.004	1.26	0.020	1.09	0.302
1416532_at	transformation/transcription domain-associated protein	Trrap	1.25	0.004	-1.04	0.611	1.06	0.469
1428806_at	casein kinase 1, gamma 1	Csnk1g1	1.25	0.008	1.15	0.181	1.40	0.001
1456628_x_at	ribosomal protein S24	Rps24	1.25	0.003	-1.08	0.398	-1.14	0.125
1435725_x_at	similar to 60S ribosomal protein L12	LOC241053 /// Rpl1	1.25	0.001	-1.05	0.394	1.01	0.826
1448491_at	enoyl coenzyme A hydratase 1, peroxisomal	Ech1	1.25	0.000	1.01	0.907	1.04	0.443
1428082_at	acyl-CoA synthetase long-chain family member 5	Acsl5	1.25	0.002	1.12	0.043	1.05	0.285
1421926_at	mitogen-activated protein kinase 11	Mapk11	1.25	0.001	1.07	0.241	1.05	0.503
1426743_at	Dip3 beta	MGI:2384914	1.25	0.005	1.16	0.087	1.09	0.330
1434003_a_at	deoxyhypusine synthase	Dhps	1.25	0.002	1.07	0.307	-1.01	0.945
1433983_at	Cnksr family member 3	Cnksr3	1.25	0.001	-1.11	0.019	-1.02	0.605
1415907_at	cyclin D3	Ccnd3	1.25	0.004	1.01	0.942	-1.05	0.558
1417615_a_at	ribosomal protein L11	Rpl11	1.25	0.000	1.06	0.163	1.00	0.986
1444040_at	leukocyte-associated Ig-like receptor 1	Lair1	1.25	0.000	1.18	0.004	1.17	0.017
1441386_at	Rap guanine nucleotide exchange factor (GEF) 1	Rapgef1	1.25	0.003	1.02	0.435	1.02	0.487
1460669_at	interleukin enhancer binding factor 3	Ilf3	1.25	0.009	1.19	0.071	1.12	0.043
1454620_x_at	ribosomal protein S6	Rps6	1.25	0.000	-1.08	0.173	-1.02	0.728
1454889_x_at	transmembrane and coiled coil domains 3	Tmcc3	1.25	0.008	-1.02	0.831	-1.07	0.465
1438815_at	histone 2, H2aa2	Hist2h2aa2	1.25	0.009	1.01	0.077	1.01	0.098
1433593_at	yippee-like 5 (Drosophila)	Ypel5	1.25	0.001	1.08	0.130	1.14	0.012
1448124_at	glucuronidase, beta	Gusb	1.24	0.010	1.19	0.043	1.14	0.034
1452285_a_at	eukaryotic translation initiation factor 3, subunit 5 (epsilon)	Eif3s5	1.24	0.000	-1.04	0.481	1.02	0.665
1417875_at	DEAD (Asp-Glu-Ala-Asp) box polypeptide 50	Ddx50	1.24	0.000	1.08	0.054	1.02	0.654
1435669_at	zinc finger protein 532	Zfp532	1.24	0.007	1.17	0.024	1.29	0.002

1435077_at	additional sex combs like 1 (Drosophila)	Asxl1	1.24	0.005	1.05	0.524	1.01	0.874
1424275_s_at	tripartite motif-containing 41	Trim41	1.24	0.001	-1.01	0.842	1.02	0.791
1451068_s_at	ribosomal protein S25	Rps25	1.24	0.008	1.02	0.772	1.24	0.002
1417435_at	like-glycosyltransferase	Large	1.24	0.003	1.01	0.820	1.08	0.289
1424258_at	polymerase (RNA) II (DNA directed) polypeptide D	Polr2d	1.24	0.007	1.08	0.275	1.12	0.080
1449712_s_at	ATPase, H+ transporting, V1 subunit E isoform 1	Atp6v1e1	1.24	0.001	1.04	0.580	1.07	0.305
1420930_s_at	catenin alpha-like 1	Catnal1	1.24	0.009	-1.01	0.837	1.11	0.271
1460444_at	arrestin, beta 1	Arrb1	1.24	0.002	1.11	0.140	1.14	0.069
1422797_at	mitogen activated protein binding protein interacting protein	MGI:1932697	1.24	0.010	1.02	0.810	1.10	0.200
1437972_s_at	splicing factor 3b, subunit 5	Sf3b5	1.24	0.006	-1.03	0.674	1.05	0.639
1460582_x_at	Nonagouti	a	1.24	0.009	1.26	0.009	1.18	0.005
1422675_at	SWI/SNF related, matrix associated, subfamily e, member 1	Smarce1	1.24	0.008	1.09	0.272	1.01	0.935
1447181_s_at	solute carrier family 7 (cationic amino acid transporter, y+ system), member 7	Slc7a7	1.24	0.004	1.13	0.300	1.08	0.252
1448822_at	proteasome (prosome, macropain) subunit, beta type 6	Psmb6	1.24	0.009	1.04	0.610	1.16	0.048
1427944_at	C1q domain containing 1	C1qdc1	1.24	0.004	1.21	0.034	1.39	0.002
1417718_at	eukaryotic translation initiation factor 3, subunit 4 (delta)	Eif3s4	1.24	0.001	-1.02	0.763	-1.07	0.323
1426908_at	UDP-N-acetyl-alpha-D-galactosamine	Galnt7	1.24	0.003	-1.34	0.001	-1.34	0.001
1418071_s_at	chromodomain protein, Y chromosome-like	Cdyl	1.24	0.007	1.08	0.354	1.04	0.573
1416054_at	ribosomal protein S5	Rps5	1.24	0.002	-1.06	0.318	1.02	0.766
1455662_x_at	ribosomal protein S17	Rps17	1.24	0.000	1.02	0.666	1.03	0.488
1427407_s_at	thyroid hormone receptor interactor 11	Trip11	1.24	0.005	1.08	0.329	-1.12	0.208
1434434_s_at	transcription elongation regulator 1 (CA150)	Tcerg1	1.24	0.003	-1.04	0.478	-1.07	0.169
1439455_x_at	capping protein (actin filament) muscle Z-line, alpha 1	Capza1	1.24	0.005	1.03	0.640	1.05	0.312
1417140_a_at	protein tyrosine phosphatase, non-receptor type 2	Ptpn2	1.24	0.002	1.04	0.486	1.03	0.641
1428471_at	sorbin and SH3 domain containing 1	Sorbs1	1.24	0.005	1.17	0.039	1.25	0.002
1455854_a_at	slingshot homolog 1 (Drosophila)	Ssh1	1.24	0.002	1.60	0.013	1.85	0.000
1460708_s_at	cell division cycle 42 homolog (S. cerevisiae)	Cdc42	1.24	0.000	1.09	0.070	1.03	0.527
1416291_at	proteasome (prosome, macropain) 26S subunit, ATPase, 4	Psmc4	1.24	0.006	1.03	0.556	1.11	0.067
1460581_a_at	ribosomal protein L13	Rpl13	1.24	0.005	-1.10	0.219	-1.08	0.377
1419737_a_at	lactate dehydrogenase 1, A chain	Ldh1	1.24	0.000	1.15	0.012	1.10	0.047
1436699_x_at	ribosomal protein L18	Rpl18	1.23	0.003	-1.08	0.347	-1.02	0.779
1417608_a_at	ribosomal protein L13a	Rpl13a	1.23	0.002	1.06	0.266	1.15	0.021
1423345_at	degenerative spermatocyte homolog 1 (Drosophila)	Degs1	1.23	0.006	1.10	0.288	1.21	0.005
1416268_at	E26 avian leukemia oncogene 2, 3' domain	Ets2	1.23	0.006	1.01	0.902	1.10	0.164
1435494_s_at	desmoplakin	Dsp	1.23	0.002	1.07	0.164	1.14	0.017
1450879_at	ATPas, class II, type 9B	Atp9b	1.23	0.002	1.29	0.001	1.14	0.048
1425283_a_at	metallothionein-like 5, testis-specific (tesmin)	Mtl5	1.23	0.005	-1.00	0.935	1.23	0.000
1417551_at	ceroid lipofuscinosis, neuronal 3, juvenile (Batten, Spielmeyer-Vogt disease)	Cln3	1.23	0.008	1.29	0.006	1.19	0.028
1416580_a_at	STIP1 homology and U-Box containing protein 1	Stub1	1.23	0.000	1.05	0.195	1.01	0.865
1428153_at	mitochondrial ribosomal protein S10	Mrps10	1.23	0.009	1.06	0.369	1.18	0.020
1423684_at	heterogeneous nuclear ribonucleoprotein K	Hnrpk	1.23	0.000	-1.01	0.838	-1.05	0.422

1459832_s_at	adaptor-related protein complex AP-1, mu subunit 1	Ap1m1	1.23	0.007	1.22	0.153	1.39	0.002
1455349_at	RAS related protein 1b	Rap1b	1.23	0.002	1.22	0.000	1.17	0.004
1455767_x_at	ribosomal protein L21	Rpl21	1.23	0.000	-1.08	0.165	-1.13	0.070
1416404_s_at	ribosomal protein S16	Rps16	1.23	0.002	-1.02	0.689	1.05	0.378
1436179_a_at	DnaJ (Hsp40) homolog, subfamily C, member 5	Dnajc5	1.23	0.000	1.14	0.004	1.11	0.005
1460184_at	L-3-hydroxyacyl-Coenzyme A dehydrogenase, short chain	Hadhsc	1.23	0.007	1.14	0.067	1.16	0.032
1428193_at	ubiquitin specific protease 9, X chromosome	Usp9x	1.23	0.009	-1.01	0.839	1.11	0.090
1418115_s_at	interferon alpha responsive gene	MGI:1929890	1.23	0.010	1.36	0.010	1.29	0.021
1435567_at	Phosphorylase kinase alpha 1	Phka1	1.23	0.002	1.13	0.041	1.01	0.890
1450135_at	frizzled homolog 3 (Drosophila)	Fzd3	1.23	0.000	-1.00	0.821	-1.00	0.674
1426100_a_at	thymidine kinase 2, mitochondrial	Tk2	1.23	0.002	-1.02	0.767	1.15	0.024
1452693_at	DEAH (Asp-Glu-Ala-His) box polypeptide 35	Dhx35	1.23	0.007	1.02	0.797	1.25	0.028
1426793_a_at	ribosomal protein L14	Rpl14	1.23	0.004	-1.04	0.553	-1.00	0.983
1448503_at	myeloid cell leukemia sequence 1	Mcl1	1.23	0.000	-1.07	0.234	1.01	0.768
1451266_at	mitochondrial ribosomal protein L50	Mrpl50	1.23	0.004	1.07	0.186	1.09	0.181
1434489_at	engulfment and cell motility 3, ced-12 homolog (C. elegans)	Elmo3	1.23	0.003	1.05	0.466	1.22	0.007
1450048_a_at	isocitrate dehydrogenase 2 (NADP+), mitochondrial	Idh2	1.22	0.001	-1.01	0.817	1.08	0.152
1455927_x_at	Non-SMC element 1 homolog (S. cerevisiae)	2510027N19Rik	1.22	0.001	1.01	0.784	1.12	0.028
1428407_at	heterogeneous nuclear ribonucleoprotein A0	Hnrpa0	1.22	0.001	1.02	0.816	1.00	0.972
1448754_at	retinol binding protein 1, cellular	Rbp1	1.22	0.000	1.08	0.158	1.03	0.628
1448746_at	nibrin	Nbn	1.22	0.008	1.00	0.990	1.17	0.068
1418128_at	adenylate cyclase 6	Adcy6	1.22	0.005	1.12	0.165	-1.11	0.164
1451104_a_at	U1 small nuclear ribonucleoprotein polypeptide A	Snrp70	1.22	0.004	-1.07	0.401	-1.11	0.105
1460324_at	DNA methyltransferase 3A	Dnmt3a	1.22	0.002	1.14	0.057	1.13	0.082
1428843_at	membrane-associated ring finger (C3HC4) 5	5-Mar	1.22	0.000	-1.01	0.882	-1.01	0.841
1454778_x_at	Ribosomal protein S28	Rps28	1.22	0.000	1.00	0.973	1.01	0.793
1459854_s_at	t-complex-associated-testis-expressed 1-like	Tcte1l	1.22	0.005	1.06	0.158	1.07	0.123
1415856_at	embigin	Emb	1.22	0.006	1.11	0.255	-1.04	0.560
1419649_s_at	myosin IC	Myo1c	1.22	0.006	1.03	0.664	-1.05	0.374
1450959_at	predicted gene ICRFP703B1614Q5.6	ICRFP703B1614Q5	1.22	0.003	1.07	0.198	1.16	0.014
1448948_at	recombination activating gene 1 gene activation	Rga	1.22	0.003	1.01	0.786	1.06	0.303
1438653_x_at	spinocerebellar ataxia 10 homolog (human)	Sca10	1.22	0.000	1.03	0.389	-1.01	0.853
1449256_a_at	RAB11a, member RAS oncogene family	Rab11a	1.22	0.006	1.14	0.068	1.17	0.022
1440795_x_at	rabaptin, RAB GTPase binding effector protein 2	Rabep2	1.22	0.006	1.14	0.057	1.19	0.015
1459843_s_at	MAD homolog 1 (Drosophila)	Smad1	1.22	0.001	-1.05	0.460	-1.08	0.260
1428412_at	SM-11044 binding protein	MGI:1914262	1.22	0.002	-1.03	0.439	1.06	0.201
1450081_x_at	glucose phosphate isomerase 1	Gpi1	1.22	0.007	1.03	0.636	1.11	0.258
1434821_at	bromodomain containing 1	Brd1	1.22	0.003	1.13	0.033	-1.05	0.286
1418927_a_at	hyaluronic acid binding protein 4	Habp4	1.22	0.008	1.19	0.014	1.36	0.000
1426393_a_at	stromal cell derived factor 4	Sdf4	1.22	0.000	1.11	0.012	1.19	0.001
1460639_a_at	ATX1 (antioxidant protein 1) homolog 1 (yeast)	Atox1	1.22	0.010	1.13	0.104	1.24	0.008

1448262_at	proteasome (prosome, macropain) subunit, beta type 2	Psmb2	1.22	0.001	1.04	0.421	1.20	0.000
1460182_at	sorting nexin 4	Snx4	1.22	0.006	1.11	0.042	1.12	0.062
1449415_at	chromodomain helicase DNA binding protein 1-like	Chd1l	1.22	0.000	1.04	0.439	-1.06	0.261
1448402_at	talin 1	Tln1	1.22	0.009	1.17	0.071	1.16	0.058
1448942_at	guanine nucleotide binding protein (G protein), gamma 11	Gng11	1.22	0.001	1.07	0.345	1.08	0.160
1455897_x_at	high mobility group nucleosomal binding domain 1	Hmgn1	1.22	0.006	1.08	0.190	-1.08	0.280
1437146_x_at	coronin 7	Coro7	1.22	0.002	1.09	0.160	1.11	0.107
1416546_a_at	ribosomal protein L6	Rpl6	1.22	0.001	1.05	0.184	1.04	0.280
1417892_a_at	sirtuin 3 (silent mating type information regulation 2, homolog) 3 (S. cerevisiae)	Sirt3	1.22	0.000	1.02	0.555	1.22	0.001
1418039_at	RAB, member of RAS oncogene family-like 3	Rabl3	1.22	0.002	1.08	0.239	1.03	0.567
1437976_x_at	ribosomal protein L23a	Rpl23a	1.22	0.001	1.05	0.273	1.03	0.584
1435712_a_at	ribosomal protein S18	Rps18	1.22	0.002	-1.01	0.800	1.06	0.339
1454905_at	inhibitor of Bruton agammaglobulinemia tyrosine kinase	Ibtk	1.22	0.010	1.07	0.176	-1.01	0.800
1416498_at	peptidylprolyl isomerase C	Ppic	1.22	0.004	1.22	0.017	1.23	0.007
1415723_at	eukaryotic translation initiation factor 5	Eif5	1.22	0.003	1.09	0.247	1.01	0.905
1451294_s_at	small nuclear ribonucleoprotein E	Snrpe	1.22	0.002	1.03	0.571	1.01	0.857
1455245_x_at	ribosomal protein L13	Rpl13	1.22	0.001	-1.05	0.417	-1.10	0.133
1452683_at	DnaJ (Hsp40) homolog, subfamily C, member 8	Dnajc8	1.22	0.000	1.17	0.009	1.20	0.001
1424013_at	eukaryotic translation termination factor 1	Etf1	1.22	0.000	-1.00	0.997	1.09	0.051
1451740_at	polyadenylate binding protein-interacting protein 1	Paip1	1.21	0.000	-1.01	0.678	-1.10	0.005
1415918_a_at	triosephosphate isomerase 1	Tpi1	1.21	0.001	1.12	0.055	1.20	0.001
1417579_x_at	GDP-mannose pyrophosphorylase A	Gmppa	1.21	0.000	1.09	0.081	1.10	0.032
1460224_at	sorting nexin 2	Snx2	1.21	0.008	1.20	0.013	1.08	0.235
1417250_at	ring finger protein 12	Rnf12	1.21	0.001	-1.12	0.099	-1.02	0.703
1423807_a_at	calmodulin 2	Calm2	1.21	0.009	1.21	0.010	1.14	0.063
1435881_at	poly(rC) binding protein 2	Pcbp2	1.21	0.006	-1.05	0.393	-1.09	0.180
1460722_at	sterol O-acyltransferase 2	Soat2	1.21	0.004	1.07	0.264	1.21	0.002
1437539_at	Protein kinase, AMP-activated, alpha 1 catalytic subunit	Prkaa1	1.21	0.003	1.07	0.345	-1.01	0.840
1424595_at	F11 receptor	F11r	1.21	0.000	1.01	0.808	1.12	0.014
1439456_x_at	ATPase, H+ transporting, lysosomal accessory protein 2	Atp6ap2	1.21	0.005	-1.00	0.940	-1.01	0.802
1434358_x_at	ribosomal protein S21	Rps21	1.21	0.000	-1.03	0.597	1.01	0.825
1454916_s_at	ADP-ribosylation factor interacting protein 1	Arfip1	1.21	0.003	1.08	0.226	1.03	0.643
1436510_a_at	leucine rich repeat (in FLII) interacting protein 2	Lrrfip2	1.21	0.008	1.07	0.292	-1.03	0.659
1455141_at	trinucleotide repeat containing 6a	Tnrc6a	1.21	0.002	1.05	0.426	-1.14	0.049
1428131_a_at	CDC42 small effector 1	Cdc42se1	1.21	0.005	1.09	0.202	1.15	0.053
1448580_at	golgi apparatus protein 1	Glg1	1.21	0.002	-1.10	0.136	1.01	0.905
1448960_at	CXXC finger 5	Cxxc5	1.21	0.009	1.15	0.138	1.22	0.011
1458182_at	RNA binding motif protein 18	Rbm18	1.21	0.009	1.07	0.358	-1.00	0.957
1420907_at	CD2-associated protein	Cd2ap	1.21	0.003	1.01	0.851	-1.01	0.808
1452147_at	SEC24 related gene family, member C (S. cerevisiae)	Sec24c	1.21	0.000	1.03	0.527	1.02	0.512
1460623_at	Src family associated phosphoprotein 2	Scap2	1.21	0.001	1.12	0.063	1.11	0.086

1423781_at	amyloid beta precursor protein binding protein 1	Appbp1	1.21	0.005	1.09	0.158	1.18	0.020
1460402_at	bromodomain and PHD finger containing, 1	Brpf1	1.21	0.007	-1.11	0.165	-1.24	0.023
1421873_s_at	RAB24, member RAS oncogene family	Rab24	1.21	0.001	1.17	0.003	1.32	0.000
1448422_at	transmembrane emp24 protein transport domain containing 4	Tmed4	1.21	0.000	1.06	0.258	1.13	0.016
1452026_a_at	phospholipase A2, group XIIA	Pla2g12a	1.21	0.001	-1.19	0.005	-1.05	0.413
1457024_x_at	solute carrier family 35 (UDP-galactose transporter), member 2	Slc35a2	1.21	0.007	1.11	0.109	1.17	0.013
1433648_at	sperm associated antigen 9	Spag9	1.21	0.002	1.10	0.061	1.04	0.518
1418448_at	Harvey rat sarcoma oncogene, subgroup R	Rras	1.21	0.002	1.11	0.142	1.08	0.163
1418993_s_at	coagulation factor X	F10	1.21	0.001	-1.01	0.895	1.05	0.363
1457271_at	gene model 131, (NCBI)	Gm131	1.21	0.007	1.22	0.004	1.05	0.317
1418337_at	ribose 5-phosphate isomerase A	Rpia	1.21	0.003	1.18	0.005	1.06	0.430
1448987_at	acetyl-Coenzyme A dehydrogenase, long-chain	Acadl	1.21	0.000	1.03	0.513	1.02	0.673
1448206_at	proteasome (prosome, macropain) subunit, alpha type 2	Psma2	1.21	0.003	1.07	0.201	1.11	0.072
1432176_a_at	endoglin	Eng	1.21	0.000	1.01	0.731	-1.01	0.659
1424662_at	peptidase (mitochondrial processing) alpha	Pmpca	1.21	0.002	-1.01	0.837	1.00	0.940
1456061_at	GTPase, IMAP family member 8	Gimap8	1.21	0.007	1.01	0.901	1.04	0.567
1455105_at	protein tyrosine phosphatase, non-receptor type 12	Ptpn12	1.20	0.000	1.10	0.032	-1.03	0.487
1423057_at	capping protein (actin filament) muscle Z-line, alpha 2	Capza2	1.20	0.000	1.09	0.015	1.05	0.089
1427060_at	mitogen activated protein kinase 3	Mapk3	1.20	0.000	1.14	0.015	1.11	0.007
1448495_at	tissue specific transplantation antigen P35B	Tsta3	1.20	0.003	-1.03	0.556	1.04	0.485
1428263_a_at	transcription elongation factor B (SIII), polypeptide 2	Tceb2	1.20	0.004	1.06	0.279	1.05	0.501
1417666_at	deoxynucleotidyltransferase, terminal, interacting protein 1	Dnttip1	1.20	0.001	-1.10	0.046	-1.03	0.567
1451854_a_at	shroom	Shrm	1.20	0.009	1.00	#DIV/0!	1.15	0.009
1456205_x_at	tubulin cofactor a	Tbca	1.20	0.002	1.01	0.859	1.02	0.755
1420624_a_at	vesicle-associated membrane protein 8	Vamp8	1.20	0.003	1.01	0.844	1.15	0.018
1460543_x_at	ribosomal protein L37a	Rpl37a	1.20	0.002	1.01	0.893	-1.02	0.685
1423370_a_at	casein kinase 1, gamma 2	Csnk1g2	1.20	0.004	1.02	0.654	1.10	0.042
1419638_at	ephrin B2	Efnb2	1.20	0.005	1.27	0.004	1.40	0.005
1418545_at	WASP family 1	Wasf1	1.20	0.002	1.21	0.005	1.08	0.117
1437735_at	protein phosphatase 1, regulatory (inhibitor) subunit 12A	Ppp1r12a	1.20	0.000	-1.01	0.870	-1.09	0.068
1448282_at	pleiotropic regulator 1, PRL1 homolog (Arabidopsis)	Plrg1	1.20	0.002	1.15	0.069	1.20	0.003
1452186_at	RNA binding motif protein 5	Rbm5	1.20	0.004	1.11	0.061	1.10	0.109
1424370_s_at	proteasome (prosome, macropain) inhibitor subunit 1	Psmf1	1.20	0.004	1.06	0.271	1.05	0.408
1433522_at	protein serine kinase H1	Pskh1	-1.20	0.003	-1.11	0.030	-1.02	0.617
1451714_a_at	mitogen activated protein kinase kinase 3	Map2k3	-1.20	0.009	-1.06	0.317	1.05	0.411
1420712_a_at	hepsin	Hpn	-1.20	0.000	1.01	0.689	-1.05	0.137
1449211_at	bisphosphate 3'-nucleotidase 1	Bpnt1	-1.20	0.002	-1.08	0.137	-1.11	0.066
1454932_at	REST corepressor 1	Rcor1	-1.20	0.005	1.00	0.962	-1.07	0.213
1434765_at	E1A binding protein p300	Ep300	-1.20	0.004	-1.01	0.831	-1.04	0.532
1416771_at	trafficking protein particle complex 3	Trappc3	-1.20	0.003	-1.43	0.007	-1.45	0.002
1424235_at	ORM1-like 2 (S. cerevisiae)	Ormdl2	-1.20	0.001	1.05	0.282	1.09	0.038

1428240_at	neurexin I	Nrxn1	-1.20	0.010	-1.08	0.233	-1.16	0.030
1451396_at	protein-O-mannosyltransferase 2	Pomt2	-1.21	0.009	-1.11	0.057	-1.04	0.418
1451512_s_at	3-hydroxyisobutyryl-Coenzyme A hydrolase	Hibch	-1.21	0.001	-1.07	0.093	1.05	0.309
1435430_at	transmembrane protein 1	Tmem1	-1.21	0.006	-1.11	0.091	-1.03	0.451
1415767_at	YTH domain family 1	Ythdf1	-1.21	0.003	-1.12	0.008	-1.21	0.002
1449955_at	calcium channel, voltage-dependent, alpha 1F subunit	Cacna1f	-1.21	0.009	-1.10	0.170	-1.04	0.665
1453342_at	cell division cycle 40 homolog (yeast)	Cdc40	-1.21	0.003	-1.07	0.288	-1.15	0.030
1450237_at	deoxyribonuclease II beta	Dnase2b	-1.21	0.000	1.00	0.913	1.04	0.464
1435446_a_at	choline phosphotransferase 1	Chpt1	-1.21	0.007	-1.14	0.031	1.06	0.445
1419243_at	RAB14, member RAS oncogene family	Rab14	-1.21	0.009	-1.02	0.791	1.03	0.589
1454891_at	CDP-diacylglycerol synthase (phosphatidate cytidylyltransferase) 2	Cds2	-1.21	0.009	-1.06	0.432	-1.01	0.870
1418150_at	myotubularin related protein 4	Mtmr4	-1.21	0.010	1.10	0.251	1.03	0.569
1454840_at	methylcrotonoyl-Coenzyme A carboxylase 2 (beta)	Mccc2	-1.21	0.001	-1.01	0.718	-1.16	0.004
1447557_at	RAB interacting factor	Rabif	-1.21	0.006	1.10	0.073	-1.08	0.355
1428394_at	phytanoyl-CoA dioxygenase domain containing 1 /// leucine rich repeat containing 8	Phyhd1 /// Lrrc8	-1.21	0.006	1.05	0.291	1.23	0.009
1418127_a_at	programmed cell death 8	Pdcd8	-1.21	0.003	1.00	0.991	-1.05	0.411
1450028_a_at	LanC (bacterial lantibiotic synthetase component C)-like 2	Lancl2	-1.21	0.004	1.05	0.252	1.14	0.005
1460194_at	phytanoyl-CoA hydroxylase	Phyh	-1.22	0.009	-1.07	0.276	-1.06	0.320
1416372_at	phosphatidylserine synthase 1	Ptdss1	-1.22	0.004	-1.04	0.485	-1.10	0.085
1448621_a_at	sphingomyelin phosphodiesterase 1, acid lysosomal	Smpd1	-1.22	0.001	1.00	0.918	-1.03	0.539
1448630_a_at	succinate dehydrogenase complex, subunit C, integral membrane protein	Sdhc	-1.22	0.007	-1.00	0.951	1.08	0.283
1449001_at	isovaleryl coenzyme A dehydrogenase	Ivd	-1.22	0.001	1.14	0.004	1.20	0.001
1424247_at	Rab6 interacting protein 2	Rab6ip2	-1.22	0.009	-1.01	0.882	1.01	0.907
1424338_at	solute carrier family 6 (neurotransmitter transporter, GABA), member 13	Slc6a13	-1.22	0.001	1.03	0.421	1.19	0.005
1415688_at	ubiquitin-conjugating enzyme E2G 1	Ube2g1	-1.22	0.000	1.01	0.879	1.01	0.783
1426757_at	adenosine monophosphate deaminase 2 (isoform L)	Ampd2	-1.22	0.004	1.17	0.003	1.23	0.002
1451848_a_at	crystallin, zeta	Cryz	-1.22	0.007	-1.01	0.881	1.12	0.124
1449333_at	splicing factor 3a, subunit 1	Sf3a1	-1.22	0.000	1.08	0.157	1.02	0.710
1451504_at	coiled-coil-helix-coiled-coil-helix domain containing 3	Chchd3	-1.22	0.000	-1.02	0.602	1.03	0.572
1425557_x_at	TSC22 domain family 3	Tsc22d3	-1.22	0.010	1.02	0.826	-1.04	0.673
1455432_at	TAO kinase 1	Taok1	-1.23	0.002	-1.01	0.884	-1.12	0.031
1429550_at	ectonucleoside triphosphate diphosphohydrolase 8	Entpd8	-1.23	0.003	1.21	0.006	1.29	0.003
1425195_a_at	acetyl-Coenzyme A acetyltransferase 2	Acat2 /// Acat3	-1.23	0.006	-1.02	0.747	1.13	0.054
1419618_at	butyrobetaine (gamma), 2-oxoglutarate dioxygenase 1	Bbox1	-1.23	0.001	-1.04	0.452	1.14	0.066
1417397_at	solute carrier family 9 (sodium/hydrogen exchanger), member 1	Slc9a1	-1.23	0.009	-1.01	0.865	-1.08	0.268
1434934_at	ATP synthase mitochondrial F1 complex assembly factor 1	Atpaf1	-1.23	0.010	1.11	0.069	1.10	0.144
1420584_at	phospholipase A2, group IIC	Pla2g2c	-1.23	0.006	-1.17	0.005	-1.23	0.001
1455108_at	eukaryotic translation initiation factor 4E member 2	Eif4e2	-1.23	0.007	-1.31	0.015	-1.17	0.060
1426599_a_at	solute carrier family 2 (facilitated glucose transporter), member 1	Slc2a1	-1.23	0.003	1.12	0.031	-1.08	0.221
1424530_at	SEC14-like 2 (S. cerevisiae)	Sec14l2	-1.23	0.000	1.08	0.145	1.20	0.008
1418865_at	zinc finger protein 385	Zfp385	-1.23	0.010	1.02	0.855	-1.08	0.312

1449062_at	ketohexokinase	Khk	-1.23	0.002	-1.00	0.959	1.01	0.843
1423910_at	centaurin, gamma 3	Centg3	-1.23	0.005	1.10	0.146	1.08	0.150
1420593_a_at	TEA domain family member 3	Tead3	-1.23	0.009	-1.05	0.507	-1.24	0.021
1424147_at	AHA1, activator of heat shock 90kDa protein ATPase homolog 1 (yeast)	Ahsa1	-1.23	0.000	-1.08	0.078	-1.08	0.091
1455551_at	Signaling molecule ATTP	Attp	-1.23	0.008	1.04	0.539	1.07	0.244
1448609_at	thiosulfate sulfurtransferase, mitochondrial	Tst	-1.24	0.003	1.02	0.743	-1.04	0.387
1416712_at	peptidase 4	Pep4	-1.24	0.006	1.01	0.863	1.06	0.309
1448153_at	cytochrome c oxidase, subunit Va	Cox5a	-1.24	0.000	-1.04	0.301	1.01	0.732
1417765_a_at	amylase 1, salivary	Amy1	-1.24	0.010	-1.17	0.048	-1.12	0.107
1456427_at	Glycoprotein Ib, beta polypeptide	Gp1bb	-1.24	0.003	-1.03	0.592	-1.00	0.952
1435562_at	PDZ domain containing 8	Pdzk8	-1.24	0.005	-1.14	0.039	-1.07	0.267
1419456_at	dicarbonyl L-xylulose reductase	Dcxr	-1.24	0.002	-1.06	0.237	-1.05	0.344
1434510_at	3'-phosphoadenosine 5'-phosphosulfate synthase 2	Papss2	-1.24	0.002	1.03	0.558	-1.06	0.248
1419321_at	coagulation factor VII	F7	-1.24	0.003	-1.11	0.044	-1.09	0.087
1435841_s_at	succinate-Coenzyme A ligase, GDP-forming, beta subunit	Suclg2	-1.24	0.007	1.01	0.888	-1.11	0.099
1417480_at	f-box only protein 9	Fbxo9	-1.24	0.001	1.02	0.719	1.10	0.080
1436351_at	coenzyme Q3 homolog, methyltransferase (yeast)	Coq3	-1.24	0.003	1.02	0.768	1.08	0.316
1424197_s_at	Fanconi anemia, complementation group E	Fance	-1.24	0.010	-1.07	0.483	1.09	0.246
1421880_at	myotubularin related protein 1	Mtmr1	-1.24	0.008	1.09	0.117	1.07	0.384
1423277_at	protein tyrosine phosphatase, receptor type, K	Ptprk	-1.24	0.005	1.01	0.825	1.05	0.464
1451274_at	oxoglutarate dehydrogenase (lipoamide)	Ogdh	-1.24	0.004	-1.23	0.009	-1.14	0.040
1418570_at	nicastrin	Ncstn	-1.24	0.007	1.22	0.028	1.06	0.422
1434002_at	checkpoint supressor 1	Ches1	-1.24	0.001	1.05	0.233	1.11	0.009
1415791_at	ring finger protein 34	Rnf34	-1.24	0.007	1.01	0.826	-1.02	0.778
1450632_at	similar to aplysia ras-related homolog A2	LOC433749	-1.24	0.003	-1.16	0.093	-1.22	0.025
1451493_at	Nedd4 family interacting protein 1	Ndfip1	-1.24	0.009	-1.01	0.937	1.18	0.049
1460179_at	DnaJ (Hsp40) homolog, subfamily A, member 1	Dnaja1	-1.24	0.000	-1.05	0.358	-1.03	0.430
1417066_at	chaperone, ABC1 activity of bc1 complex like (S. pombe)	Cabc1	-1.25	0.001	-1.06	0.244	1.20	0.006
1415769_at	itchy	Itch	-1.25	0.006	-1.10	0.212	-1.13	0.075
1439148_a_at	phosphofructokinase, liver, B-type	Pfkl	-1.25	0.009	1.08	0.358	-1.05	0.495
1417284_at	mitogen-activated protein kinase associated protein 1	Mapkap1	-1.25	0.002	-1.01	0.813	1.03	0.658
1435328_at	cysteine and histidine rich 1	Cyhr1	-1.25	0.009	-1.01	0.916	-1.02	0.814
1426445_at	meningioma expressed antigen 6 (coiled-coil proline-rich)	Mgea6	-1.25	0.004	-1.09	0.171	-1.13	0.051
1460303_at	nuclear receptor subfamily 3, group C, member 1	Nr3c1	-1.25	0.008	1.08	0.197	1.04	0.561
1436192_at	brefeldin A-inhibited	Arfgef2	-1.25	0.003	-1.16	0.018	-1.04	0.543
1416861_at	signal transducing adaptor molecule (SH3 domain and ITAM motif) 1	Stam	-1.25	0.001	-1.02	0.667	-1.07	0.133
1426300_at	activated leukocyte cell adhesion molecule	Alcam	-1.25	0.004	-1.21	0.054	-1.15	0.247
1419760_a_at	open reading frame 5	ORF5	-1.26	0.001	-1.06	0.300	-1.01	0.798
1424876_s_at	spastic paraplegia 20, spartin (Troyer syndrome) homolog (human)	Spg20	-1.26	0.005	-1.07	0.341	-1.14	0.071
1448288_at	nuclear factor I/B	Nfib	-1.26	0.001	-1.36	0.001	-1.39	0.000
1433891_at	G protein-coupled receptor 48	Gpr48	-1.26	0.008	1.01	0.918	-1.02	0.735

1426723_at	WD repeat domain 48	Wdr48	-1.26	0.005	-1.07	0.267	-1.06	0.299
1424179_at	pleckstrin homology domain containing, family J member 1	Plekhj1	-1.26	0.000	1.01	0.806	1.07	0.150
1423644_at	aconitase 1	Aco1	-1.26	0.002	-1.00	0.987	1.05	0.457
1449118_at	dihydrolipoamide branched chain transacylase E2	Dbt	-1.26	0.003	-1.37	0.004	-1.37	0.001
1448346_at	cofilin 1, non-muscle	Cfl1	-1.26	0.006	1.06	0.169	1.04	0.274
1434513_at	gene model 542, (NCBI)	Gm542	-1.26	0.005	-1.10	0.158	-1.18	0.027
1454753_at	arginyl aminopeptidase (aminopeptidase B)-like 1	Rnpepl1	-1.26	0.009	-1.04	0.618	-1.04	0.560
1438173_x_at	polyamine-modulated factor 1	Pmf1	-1.26	0.007	-1.05	0.466	-1.26	0.009
1443896_at	TBC1 domain family, member 5	Tbc1d5	-1.26	0.000	-1.06	0.248	1.06	0.161
1421076_at	SERTA domain containing 3	Sertad3	-1.26	0.002	-1.14	0.073	-1.44	0.000
1451066_at	leukocyte receptor cluster (LRC) member 4	Leng4	-1.26	0.002	-1.02	0.711	-1.12	0.105
1439153_at	IBR domain containing 2	Ibrdc2	-1.26	0.001	1.10	0.078	1.08	0.129
1435043_at	phospholipase C, beta 1	Plcb1	-1.26	0.000	-1.20	0.003	-1.24	0.001
1452140_at	TBC1 domain family, member 20	Tbc1d20	-1.27	0.001	-1.26	0.004	-1.19	0.014
1418706_at	solute carrier family 38, member 3	Slc38a3	-1.27	0.003	-1.01	0.920	-1.02	0.709
1428613_at	lactate dehydrogenase D	Ldhd	-1.27	0.004	-1.16	0.027	1.04	0.588
1452309_at	cingulin-like 1	Cgnl1	-1.27	0.009	-1.04	0.543	1.10	0.193
1416825_at	syntrophin, acidic 1	Snta1	-1.27	0.002	-1.01	0.901	-1.06	0.283
1417908_s_at	ubiquitin-conjugating enzyme E2L 3	Ube2l3	-1.27	0.000	-1.14	0.032	-1.06	0.132
1420622_a_at	heat shock protein 8	Hspa8	-1.27	0.009	-1.06	0.524	-1.25	0.019
1427073_at	lactation elevated 1	Lace1	-1.27	0.000	-1.08	0.102	1.09	0.107
1418771_a_at	carboxypeptidase B2 (plasma)	Cpb2	-1.27	0.001	-1.05	0.399	-1.12	0.045
1423474_at	topoisomerase (DNA) I	Top1	-1.27	0.004	-1.05	0.620	-1.25	0.014
1460189_at	WD repeat domain 23	Wdr23	-1.27	0.000	1.00	0.986	-1.01	0.853
1437747_at	ubiquitination factor E4A, UFD2 homolog (S. cerevisiae)	Ube4a	-1.27	0.005	-1.11	0.101	-1.12	0.035
1438843_x_at	mitochondrial carrier homolog 2 (C. elegans)	Mtch2	-1.27	0.002	-1.09	0.156	-1.04	0.495
1433992_at	apical protein, Xenopus laevis-like	Apxl	-1.27	0.005	-1.03	0.680	-1.15	0.069
1455992_at	vestigial like 4 (Drosophila)	Vgll4	-1.27	0.002	-1.04	0.535	-1.29	0.003
1448893_at	nuclear receptor co-repressor 2	Ncor2	-1.27	0.004	-1.01	0.911	-1.01	0.943
1455084_x_at	serine hydroxymethyl transferase 2 (mitochondrial)	Shmt2	-1.27	0.008	1.14	0.043	1.15	0.054
1451112_s_at	death-associated protein	Dap	-1.27	0.001	1.05	0.109	-1.00	0.940
1422577_at	citrate synthase	Cs	-1.28	0.006	-1.03	0.690	1.16	0.042
1449180_at	potassium channel modulatory factor 1	Kcmf1	-1.28	0.010	-1.28	0.056	-1.42	0.010
1419399_at	microsomal triglyceride transfer protein	Mttp	-1.28	0.002	-1.06	0.356	-1.02	0.736
1419814_s_at	S100 calcium binding protein A1	S100a1	-1.28	0.010	1.04	0.607	1.25	0.021
1417422_at	glycine N-methyltransferase	Gnmt	-1.28	0.001	1.03	0.326	1.11	0.055
1460717_at	testis-specific protein, Y-encoded-like 1	Tspyl1	-1.28	0.000	-1.09	0.154	-1.18	0.009
1452307_at	Cdk5 and Abl enzyme substrate 2	Cables2	-1.28	0.003	1.04	0.483	1.13	0.028
1452055_at	CTD small phosphatase 1	Ctdsp1	-1.28	0.008	-1.07	0.385	-1.16	0.036
1449628_s_at	START domain containing 7	Stard7	-1.28	0.002	-1.03	0.631	-1.09	0.198
1434326_x_at	coronin, actin binding protein, 2B	Coro2b	-1.28	0.005	-1.07	0.364	-1.06	0.464

1427253_s_at	suppressor of zeste 12 homolog (Drosophila)	Suz12	-1.28	0.003	-1.24	0.016	-1.20	0.020
1422766_at	staufen (RNA binding protein) homolog 1 (Drosophila)	Stau1	-1.28	0.005	-1.11	0.148	1.00	0.955
1449419_at	dedicator of cytokinesis 8	Dock8	-1.28	0.006	1.14	0.127	1.46	0.002
1460681_at	CEA-related cell adhesion molecule 2	Ceacam2	-1.28	0.006	1.08	0.269	1.18	0.041
1424172_at	hydroxyacyl glutathione hydrolase	Hagh	-1.28	0.003	1.07	0.231	1.10	0.156
1415757_at	golgi-specific brefeldin A-resistance factor 1	Gbf1	-1.28	0.005	1.10	0.148	1.02	0.807
1451092_a_at	RAN GTPase activating protein 1	Rangap1	-1.28	0.003	-1.07	0.317	-1.14	0.129
1417831_at	SMC (structural maintenance of chromosomes 1)-like 1 (S. cerevisiae)	Smc1l1	-1.28	0.003	-1.33	0.014	-1.34	0.013
1457268_at	DOT1-like, histone H3 methyltransferase (S. cerevisiae)	Dot1l	-1.28	0.009	-1.25	0.025	-1.43	0.001
1451397_at	trinucleotide repeat containing 15	Tnrc15	-1.29	0.006	-1.44	0.026	-1.51	0.005
1451638_s_at	armadillo repeat containing 1	Armc1	-1.29	0.002	-1.36	0.006	-1.23	0.016
1416679_at	ATP-binding cassette, sub-family D (ALD), member 3	Abcd3	-1.29	0.000	1.03	0.580	1.03	0.573
1420852_a_at	UDP-GlcNAc:betaGal beta-1,3-N-acetylglucosaminyltransferase 1	B3gnt1	-1.29	0.003	-1.01	0.849	1.04	0.676
1419560_at	lipase, hepatic	Lipc	-1.29	0.000	1.04	0.281	1.10	0.065
1452158_at	glutamyl-prolyl-tRNA synthetase	Eprs	-1.29	0.007	-1.33	0.067	-1.36	0.014
1417080_a_at	signaling intermediate in Toll pathway-evolutionarily conserved	MGI:1349469	-1.29	0.004	-1.08	0.242	-1.11	0.126
1417125_at	S-adenosylhomocysteine hydrolase	Ahcy	-1.29	0.003	-1.16	0.030	-1.08	0.209
1418172_at	heme binding protein 1	Hebp1	-1.29	0.002	-1.52	0.008	-1.31	0.012
1418604_at	arginine vasopressin receptor 1A	Avpr1a	-1.29	0.010	-1.09	0.291	1.00	0.990
1455564_at	breakpoint cluster region homolog	Bcr	-1.29	0.005	-1.07	0.418	-1.09	0.278
1433487_at	Chloride channel 3	Clcn3	-1.29	0.007	1.02	0.811	-1.18	0.041
1455073_at	cytidine and dCMP deaminase domain containing 1	Cdadc1	-1.29	0.004	-1.14	0.096	-1.03	0.621
1427345_a_at	sulfotransferase family 1A, phenol-preferring, member 1	Sult1a1	-1.29	0.002	-1.05	0.451	-1.05	0.522
1459546_s_at	ectonucleotide pyrophosphatase/phosphodiesterase 1	Enpp1	-1.29	0.000	-1.11	0.105	-1.05	0.321
1452247_at	fragile X mental retardation gene 1, autosomal homolog	Fxr1h	-1.29	0.003	-1.08	0.231	-1.16	0.036
1451339_at	sulfite oxidase	Suox	-1.29	0.001	1.07	0.285	1.06	0.303
1455020_at	sorting nexin 25	Snx25	-1.29	0.002	1.05	0.433	-1.24	0.011
1451744_a_at	zinc binding alcohol dehydrogenase, domain containing 1	Zadh1	-1.29	0.006	1.07	0.432	1.14	0.094
1441850_x_at	transcobalamin 2	Tcn2	-1.29	0.007	-1.12	0.112	1.00	0.996
1448717_at	glutaryl-Coenzyme A dehydrogenase	Gcdh	-1.29	0.001	-1.08	0.117	1.06	0.281
1421578_at	chemokine (C-C motif) ligand 4	Ccl4	-1.29	0.001	-1.17	0.030	-1.28	0.005
1459003_at	four and a half LIM domains 1	Fhl1	-1.30	0.008	1.06	0.583	-1.23	0.035
1416049_at	glycine decarboxylase	Gldc	-1.30	0.001	-1.02	0.631	-1.06	0.254
1442166_at	copine V	Cpne5	-1.30	0.008	-1.17	0.106	-1.33	0.006
1423106_at	ubiquitin-conjugating enzyme E2B, RAD6 homology (S. cerevisiae)	Ube2b	-1.30	0.001	-1.04	0.481	1.06	0.337
1425593_at	transportin 2 (importin 3, karyopherin beta 2b)	Tnpo2	-1.30	0.010	-1.06	0.648	-1.05	0.718
1428279_a_at	ataxin 7-like 4	Atxn7l4	-1.30	0.005	1.21	0.026	1.14	0.092
1421726_at	Adaptor-related protein complex AP-4, beta 1	1810038H16Rik	-1.30	0.003	-1.12	0.178	-1.09	0.252
1418946_at	ST3 beta-galactoside alpha-2,3-sialyltransferase 1	St3gal1	-1.30	0.001	1.11	0.026	-1.02	0.595
1448143_at	aldehyde dehydrogenase 2, mitochondrial	Aldh2	-1.30	0.001	-1.25	0.023	-1.35	0.000
1427940_s_at	c-myc binding protein	Mycbp	-1.31	0.008	-1.03	0.730	1.02	0.829

1416511_a_at	CDC42 effector protein (Rho GTPase binding) 4	Cdc42ep4	-1.31	0.009	-1.04	0.632	-1.08	0.325
1427459_at	carboxypeptidase N, polypeptide 2	Cpn2	-1.31	0.004	-1.03	0.594	1.01	0.920
1422799_at	HLA-B associated transcript 2	Bat2	-1.31	0.004	-1.35	0.014	-1.14	0.055
1448014_s_at	ubiquitin specific protease 24	Usp24	-1.31	0.002	-1.01	0.807	1.02	0.736
1416411_at	glutathione S-transferase, mu 2	Gstm2	-1.31	0.004	1.07	0.287	1.32	0.022
1420524_a_at	mannan-binding lectin serine protease 2	Masp2	-1.31	0.003	-1.03	0.584	1.15	0.052
1448993_at	APG3 autophagy 3-like (S. cerevisiae)	Apg3l	-1.31	0.004	1.10	0.202	1.18	0.037
1435664_at	zinc finger protein 397	Zfp397	-1.31	0.008	-1.17	0.086	-1.12	0.181
1422485_at	MAD homolog 4 (Drosophila)	Smad4	-1.31	0.008	-1.45	0.001	-1.44	0.001
1424142_at	inhibitor of kappa light polypeptide enhancer in B-cells	Ikbkap	-1.31	0.008	-1.43	0.001	-1.25	0.007
1452269_at	spectrin beta 3	Spnb3	-1.32	0.007	-1.00	0.998	-1.13	0.151
1434219_at	stromal interaction molecule 2	Stim2	-1.32	0.001	-1.02	0.782	-1.00	1.000
1433843_at	HS1 binding protein 3	Hs1bp3	-1.32	0.006	1.06	0.552	1.03	0.779
1428574_a_at	chimerin (chimaerin) 2	Chn2	-1.32	0.005	-1.08	0.450	1.13	0.084
1451465_at	ubiquitin-like 7 (bone marrow stromal cell-derived)	Ubl7	-1.32	0.002	-1.06	0.336	-1.08	0.203
1434395_at	mannosidase, alpha, class 1A, member 2	Man1a2	-1.32	0.000	-1.18	0.003	-1.06	0.303
1448389_at	WD repeat domain 5	Wdr5	-1.32	0.002	1.06	0.441	1.04	0.586
1448767_s_at	gap junction membrane channel protein beta 1	Gjb1	-1.32	0.001	-1.12	0.058	1.04	0.412
1452702_at	chloride channel 7	Clcn7	-1.32	0.005	-1.11	0.177	-1.12	0.165
1426488_at	bifunctional apoptosis regulator	Bfar	-1.32	0.008	-1.16	0.100	-1.08	0.350
1429425_at	ring finger protein 139	Rnf139	-1.32	0.002	-1.01	0.838	1.13	0.122
1426582_at	activating transcription factor 2	Atf2	-1.32	0.006	-1.05	0.634	-1.08	0.318
1448350_at	argininosuccinate lyase	Asl	-1.32	0.009	-1.08	0.331	-1.10	0.207
1417555_at	ATPase family, AAA domain containing 1	Atad1	-1.32	0.004	-1.00	0.973	1.02	0.811
1419549_at	arginase 1, liver	Arg1	-1.32	0.001	-1.10	0.040	-1.04	0.036
1427777_x_at	fibroblast growth factor receptor 4	Fgfr4	-1.32	0.009	-1.19	0.039	-1.12	0.233
1428502_at	ARP6 actin-related protein 6 homolog (yeast)	Actr6	-1.32	0.000	-1.04	0.598	-1.03	0.534
1448400_a_at	SWI/SNF related, matrix associated subfamily d, member 2	Smarcd2	-1.32	0.007	-1.12	0.186	-1.17	0.060
1435180_at	podocan	Podn	-1.33	0.007	1.04	0.551	1.01	0.916
1428393_at	neuritin 1	Nrn1	-1.33	0.002	1.01	0.841	-1.04	0.557
1435824_at	YY1 transcription factor	Yy1	-1.33	0.000	-1.06	0.318	-1.08	0.179
1435967_s_at	3-hydroxyisobutyrate dehydrogenase	Hibadh	-1.33	0.001	-1.05	0.435	-1.09	0.197
1455595_at	similar to UDP-glucuronosyltransferase 2 family, member 5	LOC231396	-1.33	0.000	-1.00	0.943	-1.02	0.799
1427899_at	ring finger protein (C3H2C3 type) 6	Rnf6	-1.33	0.002	-1.15	0.054	-1.19	0.025
1451920_a_at	replication factor C 1	Recc1	-1.33	0.009	-1.11	0.415	-1.11	0.282
1419069_at	RAB guanine nucleotide exchange factor (GEF) 1	Rabgef1	-1.33	0.004	-1.27	0.040	-1.15	0.088
1435245_at	glutaminase 2 (liver, mitochondrial)	Gls2	-1.33	0.002	1.19	0.031	1.18	0.009
1427971_at	hyperparathyroidism 2 homolog (human)	Hrpt2	-1.33	0.006	-1.09	0.254	-1.21	0.025
1424413_at	opioid growth factor receptor-like 1	Ogfrl1	-1.33	0.008	1.03	0.754	-1.07	0.498
1420654_a_at	glucan (1,4-alpha-), branching enzyme 1	Gbe1	-1.33	0.007	1.01	0.870	1.06	0.606
1423475_at	cyclin M2	Cnnm2	-1.33	0.007	1.13	0.170	1.10	0.292

1424065_at	ER degradation enhancer, mannosidase alpha-like 1	Edem1	-1.33	0.000	-1.04	0.317	-1.02	0.294
1416537_at	cysteine-rich with EGF-like domains 1	Creld1	-1.33	0.005	-1.10	0.170	1.02	0.756
1456170_x_at	calreticulin	Calr	-1.33	0.001	-1.03	0.680	-1.17	0.015
1448154_at	N-myc downstream regulated gene 2	Ndrg2	-1.33	0.001	-1.08	0.152	-1.04	0.367
1429617_at	cylindromatosis (turban tumor syndrome)	Cyld	-1.34	0.005	1.15	0.067	1.02	0.862
1452052_s_at	eukaryotic translation initiation factor 3, subunit 1 alpha	Eif3s1	-1.34	0.001	-1.10	0.128	-1.02	0.724
1419255_at	spectrin beta 2	Spnb2	-1.34	0.006	-1.40	0.008	-1.52	0.010
1425533_a_at	staufen (RNA binding protein) homolog 2 (Drosophila)	Stau2	-1.34	0.010	-1.25	0.032	-1.14	0.167
1456209_x_at	Kruppel-like factor 13	Klf13	-1.34	0.005	1.03	0.777	-1.37	0.006
1455142_at	suppressor of cytokine signaling 4	Socs4	-1.34	0.001	-1.13	0.049	-1.16	0.025
1421071_at	von Hippel-Lindau syndrome homolog	Vhlh	-1.34	0.006	1.02	0.829	1.03	0.775
1423300_at	zinc finger, DHHC domain containing 6	Zdhhc6	-1.34	0.006	-1.28	0.010	-1.24	0.020
1421135_a_at	CCR4-NOT transcription complex, subunit 8	Cnot8	-1.34	0.008	-1.08	0.349	1.17	0.056
1428676_at	transmembrane serine protease 6	Tmprss6	-1.34	0.001	-1.09	0.165	-1.05	0.395
1436514_at	glypican 4	Gpc4	-1.34	0.002	1.18	0.060	1.15	0.043
1418815_at	cadherin 2	Cdh2	-1.34	0.004	-1.19	0.089	-1.32	0.012
1417430_at	cerebellar degeneration-related 2	Cdr2	-1.34	0.005	-1.24	0.043	-1.22	0.043
1437402_x_at	transmembrane protein 41B	Tmem41b	-1.35	0.007	-1.05	0.543	-1.19	0.070
1449287_at	src-related kinase lacking C-terminal regulatory tyrosine and N-term. myr/ion sites	Srms	-1.35	0.007	-1.15	0.107	-1.13	0.333
1418236_s_at	autophagy 5-like (S. cerevisiae)	Apg5l	-1.35	0.001	-1.28	0.011	-1.04	0.530
1452385_at	ubiquitin specific protease 53	Usp53	-1.35	0.009	-1.09	0.410	-1.29	0.025
1419173_at	aminoacylase 1	Acy1	-1.35	0.007	-1.13	0.132	1.05	0.394
1447778_x_at	c6.1a protein	MGI:2389572	-1.35	0.004	-1.12	0.161	-1.13	0.123
1417987_at	biotinidase	Btd	-1.35	0.005	-1.19	0.047	-1.19	0.044
1443962_at	transcription factor Dp 2	Tfdp2	-1.35	0.006	-1.23	0.025	-1.10	0.239
1425001_at	enoyl Coenzyme A hydratase domain containing 1	Echdc1	-1.35	0.006	-1.29	0.008	-1.19	0.054
1450665_at	GA repeat binding protein, alpha	Gabpa	-1.35	0.004	-1.10	0.192	1.08	0.243
1418446_at	solute carrier family 16 (monocarboxylic acid transporters), member 2	Slc16a2	-1.35	0.008	-1.17	0.124	-1.35	0.018
1425461_at	F-box and WD-40 domain protein 11	Fbxw11	-1.35	0.010	1.02	0.829	1.05	0.597
1432417_a_at	tetraspan 2	Tspan2	-1.35	0.009	-1.15	0.153	-1.32	0.014
1436615_a_at	ornithine transcarbamylase	Otc	-1.35	0.005	-1.09	0.303	-1.14	0.148
1423942_a_at	calcium/calmodulin -dependent protein kinase II gamma	Camk2g	-1.35	0.004	-1.17	0.022	-1.26	0.046
1416836_at	low-density lipoprotein receptor-related protein 10	Lrp10	-1.35	0.003	-1.25	0.014	-1.27	0.009
1459679_s_at	myosin IB	Myo1b	-1.35	0.000	-1.04	0.491	1.00	0.984
1426149_at	serine protease inhibitor 15	Spi15	-1.35	0.007	1.01	0.866	-1.10	0.381
1417687_at	lectin, galactose binding, soluble 12	Lgals12	-1.35	0.001	1.05	0.547	-1.04	0.672
1443681_at	Similar to fibrillarin	MGC62475	-1.35	0.007	-1.26	0.009	-1.31	0.006
1456898_at	Purine rich element binding protein A	Pura	-1.35	0.002	1.09	0.084	-1.00	0.960
1431226_a_at	fibronectin type III domain containing 4	Fndc4	-1.35	0.004	-1.00	0.960	1.05	0.517
1451405_at	propionyl-Coenzyme A carboxylase, alpha polypeptide	Pcca	-1.35	0.000	1.00	0.882	1.03	0.390
1450643_s_at	acyl-CoA synthetase long-chain family member 1	Acsl1	-1.35	0.000	-1.07	0.296	-1.07	0.292

1448545_at	syndecan 2	Sdc2	-1.35	0.003	-1.33	0.034	-1.42	0.007
1416705_at	ribulose-5-phosphate-3-epimerase	Rpe	-1.35	0.005	-1.02	0.812	1.09	0.268
1436618_at	sideroflexin 5	Sfxn5	-1.36	0.002	-1.27	0.018	-1.09	0.259
1426664_x_at	prostate cancer associated protein 6	Pcanap6	-1.36	0.007	-1.09	0.403	-1.18	0.087
1436765_at	par-3 (partitioning defective 3) homolog (C. elegans)	Pard3	-1.36	0.005	-1.24	0.045	-1.13	0.205
1451260_at	aldehyde dehydrogenase 1 family, member B1	Aldh1b1	-1.36	0.004	1.05	0.552	1.06	0.475
1434329_s_at	adiponectin receptor 2	Adipor2	-1.36	0.000	1.04	0.395	1.08	0.180
1451818_at	mindbomb homolog 1 (Drosophila)	Mib1	-1.36	0.002	-1.04	0.592	-1.01	0.853
1448842_at	cysteine dioxygenase 1, cytosolic	Cdo1	-1.36	0.000	-1.09	0.073	-1.08	0.058
1451459_at	embryonic large molecule derived from yolk sac	MGI:1915033	-1.36	0.001	-1.08	0.237	-1.12	0.095
1417701_at	protein phosphatase 1, regulatory (inhibitor) subunit 14c	Ppp1r14c	-1.36	0.001	-1.07	0.326	1.03	0.688
1450071_at	ash1 (absent, small, or homeotic)-like (Drosophila)	Ash1l	-1.36	0.002	-1.22	0.011	-1.50	0.001
1436848_x_at	inositol (myo)-1(or 4)-monophosphatase 1	Impa1	-1.36	0.003	-1.03	0.733	-1.04	0.677
1418118_at	solute carrier family 22 (organic cation transporter), member 1	Slc22a1	-1.37	0.002	-1.11	0.175	-1.05	0.570
1433628_at	gene model 1, (NCBI)	Gm1	-1.37	0.001	1.00	0.974	1.07	0.326
1428764_at	GTL2, imprinted maternally expressed untranslated mRNA	Gtl2	-1.37	0.002	-1.12	0.133	-1.24	0.026
1451393_at	peroxisome biogenesis factor 26	Pex26	-1.37	0.004	-1.19	0.058	-1.07	0.411
1438067_at	neurofibromatosis 1	Nf1	-1.37	0.006	-1.11	0.233	-1.22	0.042
1424585_at	RAN binding protein 10	Ranbp10	-1.37	0.001	-1.06	0.303	1.07	0.347
1437645_at	activating transcription factor 7	Atf7	-1.37	0.001	-1.38	0.000	-1.39	0.000
1452853_at	carbohydrate kinase-like	Carkl	-1.37	0.007	-1.01	0.900	1.27	0.021
1424676_s_at	SEC14-like 4 (S. cerevisiae)	Sec14l4	-1.37	0.006	-1.15	0.080	-1.01	0.947
1425568_a_at	transmembrane protein 33	Tmem33	-1.38	0.001	-1.08	0.315	-1.00	0.959
1448910_at	peroxisomal trans-2-enoyl-CoA reductase	Pecr	-1.38	0.009	1.09	0.299	1.14	0.144
1460192_at	oxysterol binding protein-like 1A	Osbpl1a	-1.38	0.000	-1.08	0.133	-1.10	0.135
1450869_at	fibroblast growth factor 1	Fgf1	-1.38	0.003	-1.01	0.890	-1.20	0.033
1421032_a_at	DnaJ (Hsp40) homolog, subfamily B, member 12	Dnajb12	-1.38	0.001	-1.16	0.053	1.09	0.218
1430307_a_at	malic enzyme, supernatant	Mod1	-1.38	0.007	-1.21	0.066	-1.11	0.289
1429560_at	zinc finger protein 422, related sequence 1	MGI:3028594	-1.38	0.001	-1.31	0.029	-1.23	0.030
1417429_at	flavin containing monooxygenase 1	Fmo1	-1.38	0.000	1.00	0.914	1.04	0.375
1434106_at	EPM2A (laforin) interacting protein 1	Epm2aip1	-1.39	0.009	-1.49	0.015	-1.40	0.010
1448856_a_at	methionine sulfoxide reductase A	Msra	-1.39	0.000	1.00	0.902	1.19	0.003
1416818_at	parvin, alpha	Parva	-1.39	0.003	1.05	0.608	1.15	0.189
1427097_at	WW domain containing E3 ubiquitin protein ligase 1	Wwp1	-1.39	0.004	-1.10	0.281	-1.12	0.207
1449214_a_at	optic atrophy 1 homolog (human)	Opa1	-1.39	0.000	-1.06	0.162	1.01	0.878
1436056_at	kinesin family member 13B	Kif13b	-1.39	0.000	1.06	0.140	1.06	0.156
1439278_at	zinc finger and BTB domain containing 20	Zbtb20	-1.39	0.010	-1.24	0.075	-1.26	0.076
1448853_at	synaptojanin 2 binding protein	Synj2bp	-1.39	0.000	-1.14	0.048	-1.23	0.004
1451016_at	interferon-related developmental regulator 2	Ifrd2	-1.39	0.002	-1.06	0.421	1.09	0.224
1455543_at	kelch-like 18 (Drosophila)	Klhl18	-1.39	0.002	1.11	0.173	-1.01	0.925
1417032_at	ubiquitin-conjugating enzyme E2G 2	Ube2g2	-1.39	0.009	-1.12	0.338	-1.33	0.013

1425204_s_at	DEAD (Asp-Glu-Ala-Asp) box polypeptide 19a	Ddx19a /// Ddx19b	-1.39	0.005	1.10	0.260	1.13	0.172
1430849_a_at	cell growth regulator with ring finger domain 1	Cgrrf1	-1.39	0.001	-1.04	0.610	1.02	0.823
1448692_at	ubiquilin 4	Ubqln4	-1.39	0.000	1.02	0.825	-1.09	0.192
1434477_at	headcase homolog (Drosophila)	Heca	-1.39	0.008	-1.28	0.027	-1.19	0.077
1435282_at	gene model 967, (NCBI)	Gm967	-1.39	0.001	-1.17	0.054	-1.23	0.022
1417927_at	DEAD (Asp-Glu-Ala-Asp) box polypeptide 19a	Ddx19a	-1.39	0.001	-1.08	0.263	-1.09	0.206
1436308_at	zinc finger protein 292	Zfp292	-1.40	0.008	-1.08	0.459	-1.25	0.053
1424129_at	major facilitator superfamily domain containing 1	Mfsd1	-1.40	0.003	-1.07	0.394	1.08	0.382
1417110_at	mannosidase 1, alpha	Man1a	-1.40	0.000	-1.12	0.061	-1.10	0.114
1450699_at	selenium binding protein 1	Selenbp1	-1.40	0.001	-1.08	0.320	-1.16	0.065
1451226_at	peroxisomal biogenesis factor 6	Pex6	-1.40	0.000	-1.13	0.059	-1.11	0.093
1443579_s_at	DEP domain containing 6	Depdc6	-1.40	0.005	-1.04	0.666	1.12	0.202
1448155_at	programmed cell death 6 interacting protein	Pdcd6ip	-1.40	0.001	-1.39	0.012	-1.30	0.009
1423075_at	lectin, mannose-binding 2	Lman2	-1.40	0.003	1.03	0.699	1.13	0.186
1424970_at	purine-rich element binding protein G	Purg	-1.40	0.003	1.13	0.150	1.15	0.145
1428673_at	symplekin	Sympk	-1.40	0.004	-1.31	0.076	-1.19	0.090
1423493_a_at	nuclear factor I/X	Nfix	-1.40	0.001	-1.22	0.063	-1.15	0.092
1416261_at	transmembrane protein 19	Tmem19	-1.40	0.000	-1.05	0.412	1.04	0.555
1429539_at	BCL2-like 13 (apoptosis facilitator)	Bcl2l13	-1.40	0.005	-1.18	0.105	-1.27	0.025
1434554_at	tripartite motif protein 37	Trim37	-1.41	0.001	1.06	0.542	1.08	0.279
1416667_at	phenylalkylamine Ca2+ antagonist (emopamil) binding protein	Ebp	-1.41	0.000	1.02	0.686	1.13	0.020
1425178_s_at	serine hydroxymethyl transferase 1 (soluble)	Shmt1	-1.41	0.001	1.07	0.302	1.21	0.042
1417070_at	cytochrome P450, family 4, subfamily v, polypeptide 3	Cyp4v3	-1.41	0.001	-1.09	0.354	1.04	0.606
1424441_at	solute carrier family 27 (fatty acid transporter), member 4	Slc27a4	-1.41	0.005	-1.05	0.625	-1.19	0.087
1423948_at	Bcl2-associated athanogene 2	Bag2	-1.41	0.002	-1.02	0.817	-1.01	0.931
1425688_a_at	dihydropyrimidinase	Dpys	-1.42	0.000	-1.13	0.038	-1.06	0.301
1437778_at	RNA binding motif protein 15B	Rbm15b	-1.42	0.003	-1.19	0.071	-1.31	0.015
1418668_at	butyryl Coenzyme A synthetase 1	Bucs1	-1.42	0.000	-1.12	0.103	1.02	0.815
1428612_at	autophagy 7-like (S. cerevisiae)	Apg7l	-1.42	0.001	-1.08	0.315	1.02	0.792
1460743_at	tigger transposable element derived 5	Tigd5	-1.42	0.002	-1.26	0.023	-1.10	0.342
1429369_at	transportin 3	Tnpo3	-1.42	0.002	-1.51	0.009	-1.18	0.116
1450519_a_at	protein kinase, cAMP dependent, catalytic, alpha	Prkaca	-1.42	0.001	1.04	0.671	1.04	0.674
1417741_at	liver glycogen phosphorylase	Pygl	-1.42	0.000	1.06	0.367	1.02	0.707
1425504_at	myosin, light polypeptide kinase	Mylk	-1.42	0.003	1.08	0.509	-1.16	0.155
1423102_a_at	ring finger protein 10	Rnf10	-1.42	0.001	-1.35	0.022	-1.09	0.220
1460328_at	bromodomain containing 3	Brd3	-1.42	0.000	-1.47	0.000	-1.33	0.002
1429514_at	phosphatidic acid phosphatase type 2B	Ppap2b	-1.42	0.003	-1.18	0.067	-1.22	0.041
1434062_at	RAB GTPase activating protein 1-like	Rabgap1l	-1.42	0.002	-1.14	0.110	-1.14	0.078
1429772_at	plexin A2	Plxna2	-1.43	0.006	-1.12	0.350	1.01	0.948
1417220_at	fumarylacetoacetate hydrolase	Fah	-1.43	0.001	-1.03	0.612	-1.11	0.150
1421841_at	fibroblast growth factor receptor 3	Fgfr3	-1.43	0.001	-1.12	0.122	-1.32	0.004

1428743_at	Bri3 binding protein	Bri3bp	-1.43	0.005	-1.25	0.041	-1.13	0.256
1419180_at	B-cell CLL/lymphoma 9-like	Bcl9l	-1.43	0.008	-1.12	0.302	-1.28	0.047
1432372_a_at	sepiapterin reductase	Spr	-1.43	0.004	-1.30	0.018	-1.26	0.021
1417668_at	reticulon 4 interacting protein 1	Rtn4ip1	-1.43	0.000	1.01	0.912	-1.05	0.550
1421992_a_at	insulin-like growth factor binding protein 4	Igfbp4	-1.43	0.000	-1.08	0.183	-1.09	0.150
1450006_at	nuclear receptor coactivator 4	Ncoa4	-1.43	0.009	-1.32	0.017	-1.19	0.110
1449966_s_at	calcium binding protein 39-like	Cab39l	-1.43	0.002	-1.20	0.054	-1.07	0.465
1420556_at	oxytocin	Oxt	-1.43	0.008	-1.25	0.072	-1.35	0.014
1426824_at	proteasome (prosome, macropain) activator subunit 4	Psme4	-1.44	0.002	-1.10	0.367	1.00	0.954
1452944_at	synaptogyrin 2	Syngr2	-1.44	0.001	-1.00	1.000	-1.02	0.803
1447908_x_at	tetratricopeptide repeat domain 3	Ttc3	-1.44	0.007	1.08	0.410	-1.27	0.047
1434930_at	two pore channel 1	Tpcn1	-1.44	0.006	1.08	0.553	-1.26	0.012
1419034_at	casein kinase II, alpha 1 polypeptide	Csnk2a1	-1.44	0.007	-1.42	0.021	-1.31	0.060
1423117_at	pumilio 1 (Drosophila)	Pum1	-1.44	0.004	-1.61	0.005	-1.37	0.012
1453836_a_at	monoglyceride lipase	Mgll	-1.44	0.000	-1.09	0.410	1.02	0.781
1424586_at	EH domain binding protein 1	Ehbp1	-1.44	0.007	-1.05	0.667	-1.17	0.147
1422548_at	dehydrodolichyl diphosphate synthase	Dhdds	-1.44	0.003	1.02	0.837	1.00	0.960
1449552_at	zinc finger RNA binding protein	Zfr	-1.44	0.009	1.06	0.559	1.02	0.870
1458268_s_at	insulin-like growth factor binding protein 3	Igfbp3	-1.44	0.001	1.06	0.463	-1.10	0.161
1417135_at	serine/arginine-rich protein specific kinase 2	Srpk2	-1.45	0.009	-1.53	0.004	-1.32	0.046
1448426_at	Sarcosine dehydrogenase	Sardh	-1.45	0.000	1.01	0.894	1.04	0.378
1449834_at	PDZ domain containing, X chromosome	Pdzx	-1.45	0.003	-1.00	0.969	1.18	0.013
1429005_at	malignant fibrous histiocytoma amplified sequence 1	Mfhas1	-1.45	0.000	1.00	0.960	-1.02	0.701
1437040_at	ethanolamine kinase 2	Etnk2	-1.45	0.004	-1.13	0.146	-1.25	0.011
1419002_s_at	bile acid-Coenzyme A: amino acid N-acyltransferase	Baat	-1.45	0.003	-1.09	0.423	1.53	0.003
1434681_at	CXORF15	4932441K18	-1.45	0.001	-1.06	0.280	-1.05	0.303
1418344_at	transmembrane protein 8 (five membrane-spanning domains)	Tmem8	-1.45	0.003	-1.23	0.078	-1.20	0.065
1437119_at	Endoplasmic reticulum (ER) to nucleus signalling 1	Ern1	-1.45	0.002	-1.01	0.922	-1.03	0.717
1428092_at	cell division cycle 5-like (S. pombe)	Cdc5l	-1.46	0.004	-1.27	0.035	-1.08	0.377
1449956_at	protein kinase C, epsilon	Prkce	-1.46	0.002	-1.27	0.051	-1.33	0.008
1433408_a_at	minichromosome maintenance deficient 10 (S. cerevisiae)	Mcm10	-1.46	0.009	-1.07	0.485	1.15	0.182
1417621_at	nuclear factor of activated T-cells, cytoplasmic, calcineurin-dependent 1	Nfatc1	-1.46	0.009	-1.12	0.294	-1.11	0.328
1419922_s_at	attractin like 1	Atrnl1	-1.46	0.000	-1.15	0.043	-1.14	0.032
1443832_s_at	serum deprivation response	Sdpr	-1.46	0.007	-1.41	0.011	-1.24	0.052
1417632_at	ATPase, H+ transporting, lysosomal V0 subunit a isoform 1	Atp6v0a1	-1.46	0.001	-1.05	0.552	-1.06	0.518
1429431_at	zinc finger protein, subfamily 1A, 5	Zfpn1a5	-1.46	0.001	-1.27	0.026	-1.37	0.004
1431252_a_at	zinc finger protein 655	Zfp655	-1.46	0.003	-1.28	0.037	-1.02	0.820
1452497_a_at	nuclear factor of activated T-cells, cytoplasmic, calcineurin-dependent 3	Nfatc3	-1.46	0.005	-1.45	0.009	-1.05	0.723
1448489_at	platelet-activating factor acetylhydrolase 2	Pafah2	-1.46	0.000	-1.09	0.220	-1.02	0.708
1426429_at	THAP domain containing 7	Thap7	-1.46	0.002	1.05	0.524	-1.20	0.101
1452441_at	PHD finger protein 3	Phf3	-1.47	0.005	-1.19	0.104	-1.39	0.002

1418654_at	hydroxyacid oxidase (glycolate oxidase) 3	Hao3	-1.47	0.003	1.04	0.653	1.13	0.192
1418490_at	serine dehydratase-like	Sdsl	-1.47	0.004	1.06	0.492	1.07	0.401
1436053_at	TBC1 domain family, member 22B	Tbc1d22b	-1.47	0.004	1.05	0.607	-1.39	0.009
1450261_a_at	solute carrier family 10 (sodium/bile acid cotransporter family), member 1	Slc10a1	-1.47	0.001	1.06	0.456	1.09	0.465
1450083_at	CCR4-NOT transcription complex, subunit 4	Cnot4	-1.47	0.006	-1.45	0.020	-1.26	0.061
1426989_at	calsyntenin 3	Clstn3	-1.47	0.000	-1.12	0.145	-1.06	0.449
1426463_at	gephyrin	Gphn	-1.47	0.001	-1.17	0.052	-1.05	0.588
1416983_s_at	forkhead box O1	Foxo1	-1.47	0.004	-1.29	0.061	-1.60	0.001
1423417_at	SWI/SNF related, matrix associated, subfamily c, member 1	Smarcc1	-1.47	0.001	-1.20	0.043	-1.06	0.496
1460452_at	motile sperm domain containing 3	Mospd3	-1.47	0.005	-1.00	1.000	1.06	0.516
1421085_at	retinoschisis 1 homolog (human)	Rs1h	-1.48	0.000	-1.27	0.014	-1.39	0.001
1451627_a_at	solute carrier family 1 (glial high affinity glutamate transporter), member 2	Slc1a2	-1.48	0.006	-1.08	0.454	1.29	0.111
1450576_a_at	splicing factor 3a, subunit 2	Sf3a2	-1.48	0.008	-1.04	0.758	-1.27	0.036
1454718_at	N-acetylglucosamine-1-phosphodiester alpha-N-acetylglucosaminidase	Nagpa	-1.48	0.001	-1.08	0.323	-1.08	0.419
1424207_at	SWI/SNF related, matrix associated, subfamily a	Smarca5	-1.48	0.006	-1.11	0.373	1.05	0.486
1452434_s_at	DiGeorge syndrome critical region gene 6	Dgcr6	-1.48	0.001	-1.23	0.035	1.11	0.194
1428690_at	trypsin domain containing 1	Tysnd1	-1.49	0.002	-1.18	0.142	1.01	0.905
1460673_at	folylpolyglutamyl synthetase	Fpgs	-1.49	0.000	1.03	0.599	1.02	0.703
1422053_at	inhibin beta-A	Inhba	-1.49	0.003	-1.48	0.038	-1.15	0.285
1427393_at	coagulation factor IX	F9	-1.49	0.001	-1.02	0.834	-1.09	0.270
1426744_at	sterol regulatory element binding factor 2	Srebf2	-1.49	0.000	-1.08	0.292	-1.17	0.005
1425845_a_at	soc-2 (suppressor of clear) homolog (C. elegans)	Shoc2	-1.49	0.008	-1.08	0.473	-1.16	0.232
1459864_at	G protein-coupled receptor 146	Gpr146	-1.49	0.006	-1.17	0.119	-1.23	0.053
1422678_at	diacylglycerol O-acyltransferase 2	Dgat2	-1.49	0.003	-1.09	0.366	-1.14	0.179
1422842_at	5'-3' exoribonuclease 2	Xrn2	-1.49	0.001	-1.50	0.004	-1.25	0.015
1425314_at	monogenic, audiogenic seizure susceptibility 1	Mass1	-1.50	0.004	-1.08	0.493	-1.11	0.427
1427051_at	tankyrase 1 binding protein 1	Tnks1bp1	-1.50	0.004	1.05	0.649	-1.03	0.777
1417871_at	hydroxysteroid (17-beta) dehydrogenase 7	Hsd17b7	-1.50	0.000	-1.32	0.008	-1.33	0.009
1451977_at	dual-specificity tyrosine-(Y)-phosphorylation regulated kinase 1a	Dyrk1a	-1.50	0.001	1.01	0.921	1.08	0.397
1428497_at	SECIS binding protein 2	Secisbp2	-1.50	0.002	-1.39	0.023	-1.24	0.054
1450854_at	proliferation-associated 2G4	Pa2g4	-1.50	0.003	1.08	0.575	-1.31	0.019
1418998_at	kynurenine 3-monooxygenase (kynurenine 3-hydroxylase)	Kmo	-1.50	0.000	-1.06	0.298	-1.08	0.189
1449373_at	DnaJ (Hsp40) homolog, subfamily C, member 3	Dnajc3	-1.50	0.000	-1.14	0.075	-1.28	0.005
1450556_at	spectrin beta 1	Spnb1	-1.50	0.006	-1.10	0.365	-1.03	0.814
1418821_at	cytochrome P450, family 2, subfamily a, polypeptide 12	Cyp2a12	-1.51	0.000	-1.06	0.195	1.06	0.051
1456309_x_at	LIM and SH3 protein 1	Lasp1	-1.51	0.000	-1.37	0.005	-1.52	0.000
1451371_at	melanocortin 2 receptor accessory protein	Mrap	-1.51	0.002	-1.07	0.521	1.10	0.256
1431035_at	dishevelled associated activator of morphogenesis 1	Daam1	-1.51	0.008	-1.30	0.091	1.05	0.691
1460421_at	zinc finger protein 133	Zfp133	-1.51	0.010	-1.04	0.742	-1.20	0.171
1437171_x_at	gelsolin	Gsn	-1.51	0.007	-1.29	0.063	-1.34	0.041
1451501_a_at	Growth hormone receptor	Ghr	-1.51	0.001	1.02	0.816	1.11	0.168

1422894_at	Scm-like with four mbt domains 1	Sfmbt1	-1.51	0.001	-1.32	0.026	-1.30	0.019
1418278_at	apolipoprotein C-III	Apoc3	-1.51	0.000	-1.05	0.142	1.05	0.109
1423725_at	plastin 3 (T-isoform)	Pls3	-1.52	0.000	-1.03	0.669	-1.08	0.339
1427946_s_at	dihydropyrimidine dehydrogenase	Dpyd	-1.52	0.000	-1.06	0.412	-1.04	0.548
1442061_at	BTB (POZ) domain containing 7	Btbd7	-1.52	0.009	-1.48	0.011	-1.52	0.005
1452826_s_at	F-box and leucine-rich repeat protein 20	Fbxl20	-1.52	0.003	1.06	0.626	1.01	0.908
1436368_at	solute carrier family 16 (monocarboxylic acid transporters), member 10	Slc16a10	-1.52	0.003	-1.13	0.160	-1.15	0.152
1448185_at	homocysteine-inducible, ubiquitin-like domain member 1	Herpud1	-1.52	0.000	-1.24	0.025	-1.19	0.005
1426896_at	Zinc finger protein 191	Zfp191	-1.52	0.005	-1.28	0.068	-1.09	0.432
1452494_s_at	solute carrier organic anion transporter family, member 1b2	Slco1b2	-1.52	0.008	-1.42	0.027	-1.21	0.146
1423523_at	aminoadipate-semialdehyde synthase	Aass	-1.53	0.004	-1.20	0.094	-1.19	0.068
1425989_a_at	eyes absent 3 homolog (Drosophila)	Eya3	-1.53	0.003	-1.14	0.184	-1.15	0.135
1428563_at	DEAD (Asp-Glu-Ala-Asp) box polypeptide 10	Ddx10	-1.53	0.004	-1.16	0.247	-1.12	0.352
1427284_a_at	tocopherol (alpha) transfer protein	Ttpa	-1.53	0.000	-1.14	0.050	-1.09	0.227
1451438_s_at	C-type lectin domain family 2, member h	Clec2h	-1.54	0.001	1.06	0.530	1.46	0.002
1418518_at	furin (paired basic amino acid cleaving enzyme)	Furin	-1.54	0.006	-1.26	0.079	-1.49	0.007
1422061_at	aldo-keto reductase family 1, member C20	Akr1c20	-1.54	0.009	1.01	0.959	1.14	0.258
1429362_a_at	splicing factor 3b, subunit 2	Sf3b2	-1.54	0.006	-1.39	0.027	-1.31	0.039
1451499_at	Ca2+-dependent activator protein for secretion 2	Cadps2	-1.54	0.004	1.02	0.875	-1.01	0.952
1428885_at	synaptogyrin 2 /// arylformamidase	Syngr2 /// Afmid	-1.54	0.004	-1.29	0.051	-1.16	0.164
1432499_a_at	ubiquitination factor E4B, UFD2 homolog (S. cerevisiae)	Ube4b	-1.54	0.002	-1.25	0.077	-1.16	0.134
1460724_at	adaptor protein complex AP-2, alpha 1 subunit	Ap2a1	-1.55	0.002	-1.11	0.349	-1.12	0.250
1419590_at	cytochrome P450, family 2, subfamily b, polypeptide 9	Cyp2b9	-1.55	0.000	-1.04	0.528	-1.06	0.385
1435072_at	zinc finger, FYVE domain containing 1	Zfyve1	-1.55	0.002	-1.14	0.182	-1.06	0.558
1452353_at	G protein-coupled receptor 155	Gpr155	-1.55	0.002	1.17	0.075	1.26	0.019
1417982_at	insulin induced gene 2	Insig2	-1.55	0.009	-1.21	0.119	-1.37	0.014
1432492_a_at	3-hydroxyanthranilate 3,4-dioxygenase	Haao	-1.55	0.000	-1.01	0.849	1.11	0.099
1442684_x_at	high mobility group 20A	Hmg20a	-1.55	0.005	-1.21	0.179	-1.77	0.001
1428547_at	5' nucleotidase, ecto	Nt5e	-1.56	0.000	1.05	0.478	1.02	0.794
1423869_s_at	thioredoxin reductase 3	Txnrd3	-1.56	0.007	-1.19	0.141	-1.12	0.351
1417042_at	solute carrier family 37 (glycerol-6-phosphate transporter), member 4	Slc37a4	-1.56	0.005	-1.31	0.036	-1.36	0.023
1434212_at	NADH dehydrogenase (ubiquinone) Fe-S protein 8	Ndufs8	-1.56	0.000	-1.41	0.009	-1.18	0.041
1460387_a_at	yolk sac gene 2	Ysg2	-1.56	0.001	-1.21	0.099	-1.01	0.941
1424273_at	cytochrome P450, family 2, subfamily c, polypeptide 70	Cyp2c70	-1.56	0.000	-1.12	0.065	-1.03	0.587
1416239_at	argininosuccinate synthetase 1	Ass1	-1.56	0.000	-1.04	0.283	1.05	0.177
1422183_a_at	adrenergic receptor, alpha 1b	Adra1b	-1.57	0.001	-1.30	0.138	-1.22	0.063
1421424_a_at	alanyl (membrane) aminopeptidase	Anpep	-1.58	0.000	1.01	0.932	-1.15	0.064
1427472_a_at	complement component 8, beta subunit	C8b	-1.58	0.001	-1.02	0.791	1.17	0.073
1449302_at	ATP-binding cassette, sub-family A (ABC1), member 2	Abca2	-1.58	0.003	-1.41	0.035	-1.05	0.657
1419105_at	nuclear receptor subfamily 1, group H, member 4	Nr1h4	-1.58	0.000	-1.03	0.698	1.05	0.616
1427411_s_at	deleted in lymphocytic leukemia, 2	Dleu2	-1.58	0.001	-1.03	0.765	1.25	0.064

1425668_a_at	ST3 beta-galactoside alpha-2,3-sialyltransferase 4	St3gal4	-1.58	0.000	1.01	0.840	1.10	0.218
1452962_at	transmembrane protein 25	Tmem25	-1.58	0.002	1.10	0.394	1.11	0.282
1450191_a_at	SRY-box containing gene 13	Sox13	-1.58	0.004	-1.51	0.020	-1.16	0.207
1418766_s_at	T-cell immunoglobulin and mucin domain containing 2	Timd2	-1.58	0.003	-1.08	0.433	-1.02	0.877
1423162_s_at	sprouty protein with EVH-1 domain 1, related sequence	Spred1	-1.59	0.000	-1.28	0.002	-1.37	0.001
1420384_at	procollagen, type IV, alpha 3 (Goodpasture antigen) binding protein	Col4a3bp	-1.59	0.001	-1.31	0.108	-1.30	0.041
1436731_at	zinc finger protein 533	Zfp533	-1.59	0.001	-1.12	0.221	-1.35	0.003
1418888_a_at	selenoprotein X 1	Sepx1	-1.59	0.000	1.06	0.302	1.09	0.196
1417651_at	cytochrome P450, family 2, subfamily c, polypeptide 29	Cyp2c29	-1.59	0.004	-1.41	0.018	-1.37	0.024
1460591_at	estrogen receptor 1 (alpha)	Esr1	-1.59	0.000	-1.02	0.772	-1.02	0.780
1419277_at	ubiquitin specific protease 48	Usp48	-1.59	0.009	-1.19	0.256	1.02	0.860
1438677_at	plakophilin 4	Pkp4	-1.59	0.004	-1.50	0.013	-1.18	0.173
1451071_a_at	ATPase, Na+/K+ transporting, alpha 1 polypeptide	Atp1a1	-1.59	0.003	-1.32	0.045	-1.52	0.009
1450489_at	sal-like 1 (Drosophila)	Sall1	-1.60	0.001	-1.13	0.233	-1.01	0.934
1452646_at	tumor protein p53 inducible nuclear protein 2	Trp53inp2	-1.60	0.000	-1.04	0.650	-1.21	0.012
1425004_s_at	molybdenum cofactor synthesis 1	Mocs1	-1.60	0.008	-1.15	0.292	-1.25	0.133
1429515_at	ubiquitin protein ligase E3 component n-recognin 2	Ubr2	-1.60	0.002	-1.20	0.091	-1.36	0.014
1439802_at	Serine/threonine kinase 35	Stk35	-1.60	0.005	-1.19	0.207	-1.36	0.042
1428130_at	lectin, mannose-binding, 1	Lman1	-1.60	0.002	-1.71	0.013	-1.52	0.026
1416481_s_at	hypoxia induced gene 1	MGI:1930666	-1.61	0.000	-1.09	0.326	-1.01	0.885
1449585_at	interleukin 1 receptor accessory protein	Il1rap	-1.61	0.000	-1.03	0.570	1.00	0.940
1419528_at	sulfotransferase family 2A, dehydroepiandrosterone (DHEA)-preferring, member 2	Sult2a2	-1.61	0.000	-1.20	0.021	-1.08	0.222
1434832_at	forkhead box O3a	Foxo3a	-1.61	0.000	-1.16	0.181	-1.31	0.009
1454842_a_at	UDP-GalNAc:betaGlcNAc beta 1,3-galactosaminyltransferase, polypeptide 2	B3galnt2	-1.61	0.000	-1.00	0.982	-1.11	0.312
1421267_a_at	Cbp/p300-interacting transactivator, with Glu/Asp-rich carboxy-terminal domain, 2	Cited2	-1.61	0.009	-1.21	0.201	1.10	0.447
1420619_a_at	amino-terminal enhancer of split	Aes	-1.62	0.000	-1.02	0.532	-1.01	0.834
1418427_at	kinesin family member 5B	Kif5b	-1.62	0.009	-1.67	0.005	-1.47	0.025
1443860_at	protein tyrosine phosphatase, receptor type, D	Ptprd	-1.62	0.003	-1.17	0.313	-1.17	0.271
1415859_at	eukaryotic translation initiation factor 3, subunit 8	Eif3s8	-1.62	0.006	-2.02	0.001	-1.36	0.097
1454665_at	interferon regulatory factor 2 binding protein 2	Irf2bp2	-1.62	0.003	-1.44	0.045	-1.33	0.012
1434766_at	Protein kinase, AMP-activated, alpha 2 catalytic subunit	Prkaa2	-1.62	0.000	-1.16	0.081	-1.09	0.267
1452375_at	aldehyde dehydrogenase 4 family, member A1	Aldh4a1	-1.62	0.004	-1.18	0.162	-1.05	0.654
1424671_at	pleckstrin homology domain containing, family F (with FYVE domain) member 1	Plekhf1	-1.62	0.002	-1.02	0.875	1.01	0.933
1424709_at	sterol-C5-desaturase (fungal ERG3, delta-5-desaturase) homolog (S. cerevisae)	Sc5d	-1.62	0.009	1.10	0.414	1.07	0.583
1420410_at	nuclear receptor subfamily 5, group A, member 2	Nr5a2	-1.63	0.002	-1.45	0.003	-1.30	0.018
1419578_at	mannose binding lectin, liver (A)	Mbl1	-1.63	0.000	1.02	0.694	1.06	0.351
1422565_s_at	nuclear factor I/C	Nfic	-1.63	0.001	-1.10	0.354	1.19	0.154
1417671_at	selenocysteine lyase	Scly	-1.63	0.006	1.12	0.483	-1.05	0.682
1456599_at	nuclear transport factor 2-like export factor 2	Nxt2	-1.63	0.001	-1.12	0.321	1.04	0.656
1451480_at	E2F transcription factor 4	E2f4	-1.63	0.008	1.10	0.414	-1.03	0.809
1418982_at	CCAAT/enhancer binding protein (C/EBP), alpha	Cebpa	-1.64	0.007	-1.03	0.847	1.00	0.992

1429169_at	RNA binding motif protein 3	Rbm3	-1.64	0.002	1.12	0.250	-1.11	0.358
1427099_at	MYC-associated zinc finger protein (purine-binding transcription factor)	Maz	-1.64	0.005	-1.18	0.245	-1.15	0.273
1449051_at	peroxisome proliferator activated receptor alpha	Ppara	-1.64	0.008	-1.71	0.020	-1.78	0.012
1438735_at	hepatitis B virus x associated protein	Hbxap	-1.64	0.004	-1.63	0.004	-1.51	0.010
1456316_a_at	acyl-Coenzyme A binding domain containing 3	Acbd3	-1.64	0.004	-1.47	0.041	-1.38	0.035
1431722_a_at	arylformamidase	Afmid	-1.64	0.003	1.11	0.351	1.23	0.066
1450849_at	heterogeneous nuclear ribonucleoprotein U	Hnrpu	-1.65	0.007	-1.51	0.014	-1.42	0.024
1443889_at	LY antigen-like	LOC105892	-1.65	0.000	1.08	0.277	1.27	0.006
1455151_at	A kinase (PRKA) anchor protein (yotiao) 9	Akap9	-1.65	0.003	-1.72	0.003	-1.44	0.065
1430570_at	kynureninase (L-kynurenine hydrolase)	Kynu	-1.65	0.000	1.07	0.429	1.11	0.229
1449881_a_at	calcium-sensing receptor	Casr	-1.65	0.001	-1.40	0.020	-1.44	0.008
1416794_at	ADP-ribosylation factor-like 6 interacting protein 2	Arl6ip2	-1.65	0.005	-1.21	0.179	-1.17	0.217
1424504_at	RAB22A, member RAS oncogene family	Rab22a	-1.65	0.009	-1.52	0.015	-1.48	0.018
1448132_at	solute carrier family 19 (sodium/hydrogen exchanger), member 1	Slc19a1	-1.66	0.001	-1.34	0.018	-1.38	0.010
1418313_at	zinc finger protein (C2H2 type) 276	Zfp276	-1.66	0.001	-1.07	0.477	-1.08	0.488
1451496_at	metastasis suppressor 1	Mtss1	-1.66	0.001	-1.25	0.069	-1.45	0.009
1454014_a_at	McKusick-Kaufman syndrome protein	Mkks	-1.66	0.003	-1.20	0.173	-1.06	0.621
1421912_at	solute carrier family 23 (nucleobase transporters), member 1	Slc23a1	-1.66	0.006	1.06	0.625	1.16	0.206
1424401_at	formyltetrahydrofolate dehydrogenase	Fthfd	-1.66	0.001	-1.00	0.978	-1.42	0.005
1423201_at	nuclear receptor co-repressor 1	Ncor1	-1.67	0.002	-1.78	0.015	-1.54	0.012
1416180_a_at	radixin	Rdx	-1.67	0.002	-1.52	0.031	-1.09	0.440
1450906_at	plexin C1	Plxnc1	-1.67	0.005	-1.64	0.004	-1.57	0.014
1425050_at	isochorismatase domain containing 1	Isoc1	-1.67	0.001	-1.22	0.108	-1.05	0.650
1449292_at	RB1-inducible coiled-coil 1	Rb1cc1	-1.67	0.005	-1.41	0.032	-1.74	0.002
1452823_at	glutathione S-transferase kappa 1	Gstk1	-1.67	0.001	-1.10	0.357	1.07	0.467
1418767_at	cytochrome P450, family 4, subfamily f, polypeptide 13	Cyp4f13	-1.67	0.008	-1.40	0.046	-1.11	0.487
1423331_a_at	poliovirus receptor-related 3	Pvrl3	-1.67	0.000	-1.17	0.112	-1.05	0.507
1416661_at	eukaryotic translation initiation factor 3, subunit 10 (theta)	Eif3s10	-1.68	0.003	-1.32	0.095	-1.53	0.006
1435784_at	APG9 autophagy 9-like 1 (S. cerevisiae)	Apg9l1	-1.68	0.000	-1.11	0.287	-1.12	0.215
1431812_a_at	solute carrier family 6 (neurotransmitter transporter, glycine), member 9	Slc6a9	-1.68	0.001	-1.13	0.220	-1.17	0.169
1431142_s_at	apoptosis-inducing factor (AIF)-like mitochondrion-associated inducer of death	Amid	-1.68	0.004	-1.09	0.493	-1.07	0.588
1448825_at	pyruvate dehydrogenase kinase, isoenzyme 2	Pdk2	-1.69	0.007	-1.24	0.126	-1.10	0.470
1442615_at	Adenosine kinase	Adk	-1.69	0.005	-1.48	0.037	-1.67	0.008
1454654_at	disrupted in renal carcinoma 2 (human)	Dirc2	-1.69	0.005	-1.40	0.027	-1.35	0.038
1456074_at	orphan short chain dehydrogenase/reductase	MGI:1917311	-1.70	0.007	-1.01	0.967	1.13	0.415
1421946_at	C-reactive protein, petaxin related	Crp	-1.70	0.002	-1.12	0.306	-1.22	0.102
1438226_at	similar to hypothetical protein MGC955	BC035522	-1.70	0.000	-1.06	0.344	1.02	0.784
1437354_at	Ubiquitin protein ligase E3A	Ube3a	-1.71	0.005	-1.13	0.356	-1.20	0.178
1423674_at	ubiquitin specific protease 1	Usp1	-1.71	0.002	-1.62	0.060	-1.71	0.028
1451639_at	CCAAT/enhancer binding protein (C/EBP), gamma	Cebpg	-1.71	0.008	-1.46	0.033	-1.23	0.182
1435520_at	Musashi homolog 2 (Drosophila)	Msi2h	-1.72	0.007	-1.79	0.005	-2.01	0.001

1455698_at	translocation protein 1	Tloc1	-1.73	0.002	1.01	0.948	1.07	0.561
1418853_at	apolipoprotein N	Apon	-1.73	0.004	1.29	0.023	1.43	0.008
1448309_at	adaptor-related protein complex 3, mu 1 subunit	Ap3m1	-1.73	0.007	1.05	0.704	1.06	0.676
1450224_at	procollagen, type IV, alpha 3	Col4a3	-1.73	0.005	-1.10	0.487	-1.13	0.438
1418645_at	histidine ammonia lyase	Hal	-1.73	0.000	-1.07	0.280	1.05	0.357
1416999_at	sphingomyelin phosphodiesterase 2, neutral	Smpd2	-1.73	0.005	-1.00	0.984	-1.06	0.687
1438271_at	LIM domain containing preferred translocation partner in lipoma	Lpp	-1.73	0.004	-1.67	0.004	-1.76	0.002
1423995_at	kinesin family member 1B	Kif1b	-1.73	0.000	-1.44	0.015	-1.35	0.040
1417293_at	heparan sulfate 6-O-sulfotransferase 1	Hs6st1	-1.74	0.001	1.03	0.784	-1.14	0.202
1418078_at	proteaseome (prosome, macropain) 28 subunit, 3	Psme3	-1.75	0.000	-1.00	0.996	-1.14	0.136
1427844_a_at	CCAAT/enhancer binding protein (C/EBP), beta	Cebpb	-1.75	0.008	-1.37	0.121	-1.61	0.007
1450226_at	prolactin receptor	Prlr	-1.76	0.001	1.02	0.813	1.21	0.062
1417190_at	pre-B-cell colony-enhancing factor 1	Pbef1	-1.76	0.000	-1.20	0.048	-1.29	0.013
1456102_a_at	cullin 5	Cul5	-1.76	0.001	-1.60	0.007	-1.52	0.034
1434020_at	PDGFA associated protein 1	Pdap1	-1.76	0.001	-1.90	0.007	-1.82	0.018
1451255_at	liver-specific bHLH-Zip transcription factor	MGI:1927471	-1.76	0.000	-1.13	0.102	-1.02	0.820
1423152_at	vesicle-associated membrane protein, associated protein B and C	Vapb	-1.77	0.002	-1.57	0.031	-1.76	0.010
1449442_at	peroxisomal biogenesis factor 11a	Pex11a	-1.78	0.010	-1.60	0.055	-1.97	0.006
1440966_at	Membrane-associated ring finger (C3HC4) 7	Axot	-1.79	0.007	-1.74	0.019	-2.05	0.003
1416365_at	heat shock protein 1, beta	Hspcb	-1.79	0.002	-1.58	0.009	-1.31	0.051
1418311_at	fructosamine 3 kinase	Fn3k	-1.79	0.006	-1.32	0.101	1.25	0.249
1439847_s_at	Kruppel-like factor 12	Klf12	-1.79	0.005	-1.60	0.040	-1.77	0.012
1428289_at	basic transcription element binding protein 1	Bteb1	-1.80	0.007	-1.55	0.021	-1.62	0.016
1425088_at	sodium channel, nonvoltage-gated, type I, alpha	Scnn1a	-1.80	0.000	1.00	0.984	1.03	0.782
1458129_at	RAR-related orphan receptor alpha	Rora	-1.81	0.000	-1.35	0.021	-1.48	0.006
1448973_at	sulfotransferase family 1D, member 1	Sult1d1	-1.81	0.002	-1.46	0.014	-1.40	0.017
1426545_at	trinucleotide repeat containing 6b	Tnrc6b	-1.81	0.002	-1.82	0.005	-1.60	0.027
1450035_a_at	formin binding protein 3	Fnbp3	-1.82	0.007	-1.49	0.060	-1.31	0.334
1417130_s_at	angiopoietin-like 4	Angptl4	-1.82	0.007	-1.57	0.024	-1.56	0.032
1455007_s_at	glutamic pyruvate transaminase (alanine aminotransferase) 2	Gpt2	-1.83	0.004	-1.31	0.099	-1.26	0.173
1442176_at	AT rich interactive domain 5B (Mrf1 like)	Arid5b	-1.84	0.002	-1.11	0.493	-1.02	0.843
1455770_at	Tryptophan 2,3-dioxygenase	Tdo2	-1.84	0.000	-1.22	0.020	-1.16	0.015
1422910_s_at	SMC6 structural maintenance of chromosomes 6-like 1 (yeast)	Smc6l1	-1.85	0.001	-1.87	0.001	-1.38	0.077
1451782_a_at	solute carrier family 29 (nucleoside transporters), member 1	Slc29a1	-1.86	0.000	-1.17	0.136	-1.09	0.369
1456656_at	lin 7 homolog a (C. elegans)	Lin7a	-1.87	0.003	-1.63	0.024	-1.65	0.015
1452788_at	protein phosphatase 2, regulatory subunit B (B56), epsilon isoform	Ppp2r5e	-1.87	0.000	-1.52	0.009	-1.51	0.002
1434736_at	hepatic leukemia factor	Hlf	-1.89	0.001	-1.73	0.004	-1.61	0.007
1431362_a_at	SPARC related modular calcium binding 2	Smoc2	-1.89	0.002	-1.66	0.011	-1.09	0.540
1451737_at	phosphatidylinositol 3-kinase, regulatory subunit, polypeptide 1 (p85 alpha)	Pik3r1	-1.90	0.003	-1.38	0.172	-1.11	0.501
1424744_at	serine dehydratase	Sds	-1.90	0.005	-1.00	0.973	-1.21	0.142
1424367_a_at	homer homolog 2 (Drosophila)	Homer2	-1.91	0.001	-1.25	0.179	-1.09	0.459

1416521_at	selenoprotein W, muscle 1	Sepw1	-1.92	0.001	-1.01	0.878	-1.02	0.838
1423891_at	glutathione S-transferase, theta 3	Gstt3	-1.93	0.001	-1.05	0.654	1.15	0.284
1425518_at	Rap guanine nucleotide exchange factor (GEF) 4	Rapgef4	-1.94	0.001	-1.34	0.043	-1.64	0.005
1448667_x_at	transducer of ERBB2, 2	Tob2	-1.94	0.000	-1.29	0.066	-1.45	0.006
1448927_at	potassium intermediate subfamily N, member 2	Kcnn2	-1.95	0.000	-1.11	0.270	-1.13	0.263
1425496_at	ATP-binding cassette, sub-family A (ABC1), member 3	Abca3	-1.95	0.005	-1.25	0.202	-1.05	0.747
1450494_x_at	CEA-related cell adhesion molecule 1	Ceacam1	-1.96	0.006	-1.18	0.321	1.30	0.097
1425993_a_at	heat shock protein 105	Hsp105	-1.96	0.001	1.01	0.946	1.03	0.827
1452416_at	interleukin 6 receptor, alpha	Il6ra	-1.97	0.001	-1.73	0.025	-2.09	0.003
1450434_s_at	phosphate cytidylyltransferase 1, choline, alpha isoform	Pcyt1a	-1.98	0.001	-1.46	0.015	-1.24	0.072
1452014_a_at	insulin-like growth factor 1	Igf1	-2.02	0.000	1.04	0.657	1.25	0.028
1419747_at	asialoglycoprotein receptor 2	Asgr2	-2.04	0.006	-1.35	0.107	-1.36	0.098
1431803_at	cytochrome P450, family 2, subfamily d, polypeptide 13	Cyp2d13	-2.05	0.001	-1.12	0.404	-1.03	0.774
1460174_at	dexamethasone-induced transcript	Dexi	-2.06	0.000	-1.06	0.551	1.02	0.769
1442367_at	Atpase, class VI, type 11C	Atp11c	-2.06	0.004	-1.68	0.022	-1.62	0.020
1459980_x_at	RAB3A, member RAS oncogene family	Rab3a	-2.07	0.009	-1.61	0.039	-1.30	0.185
1448506_at	serine (or cysteine) proteinase inhibitor, clade A, member 6	Serpina6	-2.07	0.000	1.13	0.094	1.14	0.102
1456112_at	translocated promoter region	Tpr	-2.07	0.008	-2.73	0.004	-2.18	0.020
1435749_at	guanine deaminase	Gda	-2.07	0.002	-1.51	0.043	-1.69	0.012
1452741_s_at	glycerol phosphate dehydrogenase 2, mitochondrial	Gpd2	-2.07	0.005	-1.40	0.088	-1.37	0.104
1417991_at	deiodinase, iodothyronine, type I	Dio1	-2.12	0.000	-1.04	0.751	1.00	0.973
1449042_at	CCCTC-binding factor	Ctcf	-2.12	0.000	-1.74	0.004	-1.35	0.029
1431829_a_at	ral guanine nucleotide dissociation stimulator-like 3	Rgl3	-2.12	0.000	-1.34	0.044	-1.09	0.515
1417513_at	ecotropic viral integration site 5	Evi5	-2.16	0.009	-2.58	0.007	-2.34	0.010
1451787_at	cytochrome P450, family 2, subfamily b, polypeptide 10	Cyp2b10	-2.22	0.005	-1.22	0.278	-1.16	0.426
1460256_at	carbonic anhydrase 3	Car3	-2.23	0.003	1.15	0.379	1.52	0.026
1455232_at	camello-like 2	Cml2	-2.23	0.001	1.39	0.008	1.79	0.005
1449479_at	cytochrome P450, family 2, subfamily b, polypeptide 13	Cyp2b13	-2.24	0.002	-1.27	0.180	-1.19	0.345
1428043_a_at	cytidine monophospho-N-acetylneuraminic acid hydroxylase	Cmah	-2.25	0.004	-1.23	0.369	-1.23	0.271
1417168_a_at	ubiquitin specific protease 2	Usp2	-2.25	0.001	-1.38	0.082	-1.61	0.005
1424684_at	RAB5C, member RAS oncogene family	Rab5c	-2.26	0.006	-1.37	0.224	-1.50	0.131
1450798_at	tenascin XB	Tnxb	-2.26	0.002	-1.90	0.007	-1.30	0.137
1448854_s_at	murinoglobulin, pseudogene 1	Mug-ps1 /// Mug1 ///	-2.28	0.001	1.06	0.735	1.36	0.053
1438743_at	cytochrome P450, family 7, subfamily a, polypeptide 1	Cyp7a1	-2.28	0.000	-1.51	0.052	-1.02	0.883
1418493_a_at	synuclein, alpha	Snca	-2.28	0.005	-1.90	0.022	-1.67	0.044
1437733_at	Eukaryotic translation initiation factor 4E binding protein 2	Eif4ebp2	-2.30	0.001	-1.10	0.500	-1.34	0.062
1425107_a_at	leukemia inhibitory factor receptor	Lifr	-2.32	0.000	-1.04	0.710	1.10	0.345
1438236_at	nuclear factor I/A	Nfia	-2.35	0.006	-1.62	0.065	-2.00	0.014
1422826_at	insulin-like growth factor binding protein, acid labile subunit	Igfals	-2.36	0.000	1.06	0.527	1.13	0.259
1448792_a_at	cytochrome P450, family 2, subfamily f, polypeptide 2	Cyp2f2	-2.36	0.001	1.30	0.054	1.76	0.001
1417835_at	murinoglobulin 1	Mug1	-2.36	0.001	-1.11	0.575	1.14	0.387

1426014_a_at	mucin and cadherin like	Mucdhl	-2.39	0.001	-1.12	0.420	-1.33	0.090
1418188_a_at	receptor (calcitonin) activity modifying protein 2	Ramp2	-2.41	0.006	-1.79	0.055	-2.33	0.023
1426873_s_at	junction plakoglobin	Jup	-2.41	0.003	-1.25	0.252	-1.75	0.020
1453435_a_at	flavin containing monooxygenase 2	Fmo2	-2.46	0.000	-1.33	0.055	-1.51	0.009
1458327_x_at	solute carrier family 26 (sulfate transporter), member 1	Slc26a1	-2.50	0.006	-1.13	0.546	-1.48	0.103
1418746_at	brain protein 17	MGI:1930773	-2.52	0.000	1.02	0.608	1.06	0.338
1451460_a_at	solute carrier family 22 (organic anion transporter), member 7	Slc22a7	-2.52	0.001	1.20	0.144	1.13	0.343
1420405_at	solute carrier organic anion transporter family, member 1a4	Slco1a4	-2.54	0.001	-1.42	0.048	-1.28	0.123
1424598_at	DEAD (Asp-Glu-Ala-Asp) box polypeptide 6	Ddx6	-2.57	0.000	-2.30	0.002	-1.87	0.045
1416833_at	kidney expressed gene 1	Keg1	-2.57	0.000	1.07	0.596	1.16	0.233
1419319_at	serum amyloid A 4	Saa4	-2.59	0.001	1.17	0.428	1.27	0.218
1436746_at	protein kinase, lysine deficient 1	Prkwnk1	-2.61	0.004	-2.46	0.008	-2.57	0.009
1436590_at	protein phosphatase 1, regulatory (inhibitor) subunit 3B	Ppp1r3b	-2.62	0.000	-1.22	0.226	-1.24	0.142
1460725_at	xeroderma pigmentosum, complementation group A	Xpa	-2.75	0.001	-2.01	0.006	-2.75	0.001
1433933_s_at	solute carrier organic anion transporter family, member 2b1	Slco2b1	-2.79	0.001	1.01	0.940	1.33	0.160
1424534_at	monocyte to macrophage differentiation-associated 2	Mmd2	-2.95	0.005	1.45	0.044	1.73	0.007
1419525_at	carbonic anhydrase 5a, mitochondrial	Car5a	-2.97	0.000	-1.32	0.105	-1.36	0.052
1417532_at	cytochrome P450, family 2, subfamily j, polypeptide 5	Cyp2j5	-3.08	0.000	-1.31	0.213	-1.47	0.024
1423522_at	nucleoplasmin 3	Npm3	-3.10	0.002	-1.91	0.032	-1.97	0.030
1418013_at	camello-like 1	Cml1	-3.12	0.001	1.00	0.989	1.44	0.035
1426502_s_at	glutamic pyruvic transaminase 1, soluble	Gpt1	-3.12	0.000	-1.12	0.375	1.21	0.135
1451776_s_at	homeobox only domain	MGI:1916782	-3.37	0.001	-1.53	0.051	-1.34	0.137
1451600_s_at	esterase 31-like /// esterase 31	LOC13909 /// Es31	-3.46	0.001	1.28	0.232	1.45	0.035
1425686_at	CASP8 and FADD-like apoptosis regulator	Cflar	-3.48	0.006	-2.43	0.023	-2.47	0.022
1419559_at	cytochrome P450, family 4, subfamily f, polypeptide 14	Cyp4f14	-3.58	0.000	-1.00	0.980	1.57	0.019
1423866_at	serine (or cysteine) proteinase inhibitor, clade A, member 3K	Serpina3k	-4.11	0.000	-1.57	0.030	-1.21	0.247
1425303_at	glucokinase	Gck	-4.80	0.004	-1.04	0.903	-1.17	0.563
1449525_at	flavin containing monooxygenase 3	Fmo3	-14.19	0.004	-2.34	0.058	-2.03	0.077
