# Supplementary material for: Impaired Genome Maintenance Suppresses the Growth Hormone–Insulin-Like Growth Factor 1 Axis in Mice with Cockayne Syndrome
Source: PLoS Biol. 2006 Dec 12;5(1):e2. doi: 10.1371/journal.pbio.0050002 (PMC1698505; doi:10.1371/journal.pbio.0050002)
Supplement: Supplementary file 3 [file pbio.0050002.st003.pdf]

Supplementary Table S3

Extensive overview of significant expression changes in 130-week old wild-type mice compared to 8-week-old mice. FC: fold change, p: p-value.

			130W	
**Code**	**Title**	**Symbol**	**FC**	**p**
1452592_at	microsomal glutathione S-transferase 2	Mgst2	11.71	0.000
1435681_s_at	similar to hypothetical protein FLJ10432	LOC234374	8.75	0.002
1449876_at	protein kinase, cGMP-dependent, type I	Prkg1	5.62	0.004
1431320_a_at	myosin Va	Myo5a	5.55	0.001
1425526_a_at	paired related homeobox 1	Prrx1	5.34	0.008
1423323_at	tumor-associated calcium signal transducer 2	Tacstd2	5.24	0.000
1449610_at	E1A binding protein p400	Ep400	5.14	0.001
1450301_at	histocompatibility 28	H28	4.94	0.004
1424155_at	fatty acid binding protein 4, adipocyte	Fabp4	4.89	0.006
1455258_at	potassium voltage gated channel, Shaw-related subfamily, member 2	Kcnc2	4.55	0.005
1441358_at	protocadherin beta 16	Pcdhb16	4.41	0.000
1447498_at	ectodysplasin-A receptor	Edar	4.26	0.004
1459296_at	DNA segment, Chr 11, KL Mohlke 35	D11Moh35	4.15	0.003
1452478_at	alpha-kinase 2	Alpk2	4.13	0.004
1447859_at	aarF domain containing kinase 2	Adck2	4.07	0.000
1435792_at	component of Sp100-rs	Csprs	4.00	0.000
1443999_at	NIMA (never in mitosis gene a)-related expressed kinase 2	Nek2	3.86	0.009
1460693_a_at	procollagen, type IX, alpha 3	Col9a3	3.73	0.009
1447348_at	Wiskott-Aldrich syndrome protein interacting protein	Waspip	3.72	0.008
1435612_at	opioid binding protein/cell adhesion molecule-like	Opcml	3.45	0.008
1417890_at	pyridoxal (pyridoxine, vitamin B6) phosphatase	Pdxp	3.34	0.006
1436861_at	interleukin 7	Il7	3.30	0.004
1424167_a_at	phosphomannomutase 1	Pmm1	3.30	0.000
1425947_at	interferon gamma	Ifng	3.17	0.005
1451839_a_at	phosphodiesterase 7A	Pde7a	3.15	0.004
1418422_at	serine (or cysteine) proteinase inhibitor, clade B, member 9g	Serpinb9g	3.12	0.002
1457082_at	Protein phosphatase 1, regulatory (inhibitor) subunit 3F	Ppp1r3f	2.85	0.009
1459852_x_at	tribbles homolog 2 (Drosophila)	Trib2	2.79	0.002
1423530_at	serine/threonine kinase 32C	Stk32c	2.79	0.007
1431402_at	kin of IRRE like 3 (Drosophila)	Kirrel3	2.75	0.003
1423166_at	CD36 antigen	Cd36	2.74	0.001
1426633_s_at	potassium channel tetramerisation domain containing 14	Kctd14	2.72	0.005
1449468_at	ST6 sialyltransferase 5	St6galnac5	2.71	0.006
1440568_at	Protection of telomeres 1	Pot1	2.69	0.005
1455811_at	eukaryotic translation initiation factor 3, subunit 6 interacting protein	Eif3s6ip	2.67	0.005
1418738_at	sodium channel, voltage-gated, type I, beta	Scn1b	2.67	0.007
1420353_at	lymphotoxin A	Lta	2.64	0.004
1425617_at	DEAH (Asp-Glu-Ala-His) box polypeptide 9	Dhx9	2.60	0.005
1446718_at	nuclear factor of kappa light chain gene enhancer in B-cells inhibitor, beta	Nfkbib	2.55	0.002
1421919_a_at	chemokine (C-C motif) receptor 9	Ccr9	2.54	0.007
1438789_s_at	dihydropyrimidinase-like 3	Dpysl3	2.53	0.005
1456392_at	neuronal growth regulator 1	Negr1	2.48	0.005
1456178_at	BMP and activin membrane-bound inhibitor, pseudogene (Xenopus laevis)	Bambi-ps1	2.48	0.002
1455772_at	progesterone receptor	Pgr	2.47	0.000
1427596_at	X (inactive)-specific transcript, antisense	Tsix	2.45	0.000
1437515_at	proliferin	Plf	2.43	0.007
1448870_at	latent transforming growth factor beta binding protein 1	Ltbp1	2.41	0.001
1446637_at	PR domain containing 10	Prdm10	2.39	0.003
1447194_at	Ras interacting protein 1	Rasip1	2.38	0.006

1425772_at	procollagen, type IV, alpha 4	Col4a4	2.38	0.007
1446787_at	COMM domain containing 1	Commd1	2.36	0.004
1450298_at	tumor necrosis factor (ligand) superfamily, member 14	Tnfsf14	2.32	0.000
1438609_x_at	troponin I, skeletal, fast 2	Tnni2	2.29	0.003
1449577_x_at	tropomyosin 2, beta	Tpm2	2.27	0.010
1420192_at	DNA segment, Chr 16, Brigham & Women's Genetics 1494 expressed	D16Bwg1494e	2.26	0.007
1446156_at	Dystrophin, muscular dystrophy	Dmd	2.26	0.002
1428640_at	heat shock transcription factor 2 binding protein	Hsf2bp	2.24	0.010
1422562_at	Ras-related associated with diabetes	Rrad	2.20	0.003
1446684_at	GTPase activating RANGAP domain-like 1	Garnl1	2.19	0.007
1449219_at	fatty acid desaturase 3	Fads3	2.18	0.002
1428055_at	RNA imprinted and accumulated in nucleus	Rian	2.17	0.005
1416737_at	glycogen synthase 3, brain	Gys3	2.17	0.006
1437601_at	orthodenticle homolog 1 (Drosophila)	Otx1	2.04	0.010
1448964_at	S100 calcium binding protein G	S100g	2.04	0.007
1439653_at	a disintegrin and metalloprotease domain 6	Adam6	2.03	0.004
1445228_at	Sprouty protein with EVH-1 domain 1, related sequence	Spred1	1.92	0.003
1434776_at	semaphorin 5A	Sema5a	1.91	0.009
1443870_at	ATP-binding cassette, sub-family C (CFTR/MRP), member 4	Abcc4	1.88	0.007
1439211_at	DNA segment, Chr 17, ERATO Doi 488, expressed	D17Ertd488e	1.87	0.006
1442341_at	kalirin, RhoGEF kinase	Kalrn	1.86	0.008
1427183_at	epidermal growth factor-containing fibulin-like extracellular matrix protein 1	Efemp1	1.85	0.005
1440503_at	B-cell receptor-associated protein 29	Bcap29	1.83	0.002
1421441_at	angiopoietin 1	Angpt1	1.82	0.001
1422952_at	Ng23 protein	MGI:2388100	1.81	0.004
1421651_at	ankyrin repeat domain 22	Ankrd22	1.80	0.009
1451191_at	cellular retinoic acid binding protein II	Crabp2	1.78	0.005
1449548_at	ephrin B2	Efnb2	1.77	0.007
1421786_at	protein phosphatase 3, regulatory subunit B, alpha isoform (calcineurin B, type I)	Ppp3r1	1.74	0.007
1438612_a_at	colipase, pancreatic	Clps	1.71	0.004
1445726_at	zinc finger protein 456	Zfp456	1.65	0.005
1441531_at	Phospholipase C, beta 4	Plcb4	1.60	0.006
1420282_s_at	implantation serine protease 2	MGI:2149952	1.56	0.002
1451301_at	tropomodulin 2	Tmod2	1.55	0.007
1448432_at	phospholipase C, delta 1	Plcd1	1.52	0.003
1423428_at	receptor tyrosine kinase-like orphan receptor 2	Ror2	1.51	0.000
1420432_at	taste receptor, type 1, member 1	Tas1r1	1.48	0.009
1447702_x_at	immunoglobulin superfamily, member 1	Igsf1	1.48	0.001
1450635_at	alkaline phosphatase 5	Akp5	1.47	0.001
1457231_at	Hypoxia inducible factor 1, alpha subunit	Hif1a	1.42	0.004
1441958_s_at	advanced glycosylation end product-specific receptor	Ager	1.39	0.000
1425336_x_at	histocompatibility 2, K1, K region	H2-K1	1.36	0.007
1447573_at	Solute carrier family 32 (GABA vesicular transporter), member 1	Viaat	1.35	0.006
1422603_at	ribonuclease, RNase A family 4	Rnase4	1.34	0.001
1419380_at	zinc finger protein 423	Zfp423	1.33	0.000
1425790_a_at	glutamate receptor, ionotropic, kainate 2 (beta 2)	Grik2	1.33	0.002
1428079_at	fibrinogen, B beta polypeptide	Fgb	1.32	0.005
1423027_at	Forkhead box L1	Foxl1	1.28	0.006
1416101_a_at	histone 1, H1c	Hist1h1c	1.25	0.003
1431627_at	kinesin family member 27	Kif27	1.21	0.006
1452032_at	protein kinase, cAMP dependent regulatory, type I, alpha	Prkar1a	-1.20	0.008

1451175_at	signal peptidase complex subunit 3 homolog (S. cerevisiae)	Spcs3	-1.20	0.001
1418896_a_at	ribophorin II	Rpn2	-1.22	0.008
1438413_at	SUMO1/sentrin specific protease 7	Senp7	-1.22	0.007
1450845_a_at	basic leucine zipper and W2 domains 1	Bzw1	-1.22	0.004
1451356_at	acidic (leucine-rich) nuclear phosphoprotein 32 family, member E	Anp32e	-1.22	0.003
1448206_at	proteasome (prosome, macropain) subunit, alpha type 2	Psma2	-1.22	0.006
1459885_s_at	cytochrome c oxidase, subunit VIIc	Cox7c	-1.23	0.002
1448304_a_at	RAB6, member RAS oncogene family	Rab6	-1.24	0.001
1418841_s_at	cell division cycle 2-like 1	Cdc2l1	-1.24	0.003
1415742_at	ancient ubiquitous protein	Aup1	-1.24	0.005
1460198_a_at	proteasome (prosome, macropain) subunit, beta type 3	Psmb3	-1.24	0.008
1415987_at	high density lipoprotein (HDL) binding protein	Hdlbp	-1.24	0.001
1427173_a_at	mitochondrial ribosomal protein S33	Mrps33	-1.24	0.002
1416485_at	translocase of inner mitochondrial membrane 23 homolog (yeast)	Timm23	-1.24	0.002
1416300_a_at	solute carrier family 25 (mitochondrial carrier, phosphate carrier), member 3	Slc25a3	-1.24	0.001
1448135_at	activating transcription factor 4	Atf4	-1.24	0.006
1423069_at	activity-dependent neuroprotective protein	Adnp	-1.26	0.006
1424604_s_at	sulfatase modifying factor 1	Sumf1	-1.26	0.009
1431832_x_at	exosome component 10	Exosc10	-1.26	0.006
1435440_at	PDZ domain containing 8	Pdzk8	-1.26	0.001
1416440_at	CD164 antigen	Cd164	-1.26	0.007
1425850_a_at	NIMA (never in mitosis gene a)-related expressed kinase 6	Nek6	-1.27	0.009
1422241_a_at	NADH dehydrogenase (ubiquinone) 1 alpha subcomplex, 1	Ndufa1	-1.27	0.005
1459151_x_at	interferon-induced protein 35	Ifi35	-1.27	0.009
1416903_at	nucleobindin 1	Nucb1	-1.27	0.003
1438000_x_at	zinc finger protein 622	Zfp622	-1.27	0.010
1417004_at	tumor susceptibility gene 101	Tsg101	-1.27	0.002
1415827_a_at	DNA segment, Chr 3, University of California at Los Angeles 1	D3Ucla1	-1.28	0.007
1446861_at	glucosamine (N-acetyl)-6-sulfatase	Gns	-1.28	0.001
1423187_at	gamma-aminobutyric acid (GABA-A) receptor-associated protein-like 2	Gabarapl2	-1.28	0.008
1451162_at	heat shock factor binding protein 1	Hsbp1	-1.28	0.004
1429476_s_at	DnaJ (Hsp40) homolog, subfamily A, member 2	Dnaja2	-1.28	0.010
1448653_at	embryonic ectoderm development	Eed	-1.28	0.007
1417124_at	destrin	Dstn	-1.29	0.001
1448684_at	protein phosphatase 1, regulatory (inhibitor) subunit 2	Ppp1r2	-1.29	0.003
1448493_at	polyadenylate-binding protein-interacting protein 2	Paip2	-1.29	0.005
1451208_at	eukaryotic translation termination factor 1	Etf1	-1.29	0.006
1424275_s_at	tripartite motif-containing 41	Trim41	-1.29	0.002
1422106_a_at	gene rich cluster, C9 gene	Grcc9	-1.29	0.002
1426912_at	ring finger and WD repeat domain 2	Rfwd2	-1.29	0.000
1416506_at	proteasome (prosome, macropain) subunit, alpha type 6	Psma6	-1.30	0.003
1428194_at	ubiquitin specific protease 9, X chromosome	Usp9x	-1.30	0.000
1450777_at	5'-3' exoribonuclease 2	Xrn2	-1.30	0.006
1455024_at	tousled-like kinase 1	Tlk1	-1.30	0.004
1416027_at	programmed cell death 6	Pdcd6	-1.30	0.008
1435429_x_at	ribosomal protein S27-like	Rps27l	-1.30	0.009
1416176_at	high mobility group box 1	Hmgb1	-1.30	0.002
1431411_a_at	retinoic acid induced 12	Rai12	-1.31	0.006
1421874_a_at	mitochondrial ribosomal protein S23	Mrps23	-1.31	0.008
1449095_at	vacuolar protein sorting 54 (yeast)	Vps54	-1.31	0.010
1417064_at	jagunal homolog 1 (Drosophila)	Jagn1	-1.31	0.006
1416461_at	GPI-anchored membrane protein 1	Gpiap1	-1.31	0.001
1416057_at	nuclear protein 15.6	Np15	-1.31	0.009
1451974_at	oxysterol binding protein-like 2	Osbpl2	-1.31	0.009
1421751_a_at	proteasome (prosome, macropain) 26S subunit, non-ATPase, 14	Psmd14	-1.31	0.009

1415817_s_at	chaperonin subunit 7 (eta)	Cct7	-1.31	0.008
1448203_at	ATP synthase, H+ transporting, mitochondrial F0 complex, subunit g	Atp5l	-1.31	0.006
1447277_s_at	prenylcysteine oxidase 1	Pcyox1	-1.31	0.002
1448875_at	zinc fingers and homeoboxes protein 1	Zhx1	-1.32	0.009
1415727_at	apolipoprotein A-I binding protein	Apoa1bp	-1.32	0.004
1454973_at	activating transcription factor 7 interacting protein	Atf7ip	-1.32	0.009
1426946_at	RAN binding protein 5	Ranbp5	-1.32	0.006
1450746_at	kelch-like ECH-associated protein 1	Keap1	-1.32	0.005
1451509_at	TAF9 RNA polymerase II, TATA box binding protein (TBP)-associated factor	Taf9	-1.32	0.006
1418887_a_at	DNA segment, Chr 11, Wayne State University 99, expressed	D11Wsu99e	-1.32	0.001
1426491_at	hect domain and RCC1 (CHC1)-like domain (RLD) 2	Herc2	-1.33	0.005
1421971_a_at	mitochondrial ribosomal protein S34	Mrps34	-1.33	0.006
1428178_s_at	trafficking protein particle complex 6B	Trappc6b	-1.33	0.008
1428440_at	solute carrier family 25 (mitochondrial carrier, Aralar), member 12	Slc25a12	-1.33	0.004
1415700_a_at	signal sequence receptor, gamma	Ssr3	-1.33	0.010
1449934_at	purine rich element binding protein A	Pura	-1.33	0.003
1452091_a_at	RNA binding motif protein 28	Rbm28	-1.33	0.003
1416786_at	activin A receptor, type 1	Acvr1	-1.33	0.004
1425977_a_at	STE20-like kinase (yeast)	Slk	-1.33	0.002
1452147_at	SEC24 related gene family, member C (S. cerevisiae)	Sec24c	-1.33	0.010
1458247_s_at	dynactin 5	Dctn5	-1.34	0.002
1419287_at	HSPC171 protein	MGI:1913570	-1.34	0.004
1431827_a_at	tousled-like kinase 2 (Arabidopsis)	Tlk2	-1.34	0.009
1437050_s_at	DNA segment, Chr 1, ERATO Doi 396, expressed	D1Ertd396e	-1.34	0.003
1452820_at	WD repeat domain 11	Wdr11	-1.34	0.007
1428621_a_at	DNA segment, Chr 11, Wayne State University 68, expressed	D11Wsu68e	-1.34	0.009
1448198_a_at	NADH dehydrogenase (ubiquinone) 1 beta subcomplex 8	Ndufb8	-1.34	0.004
1449930_a_at	signal sequence receptor, beta	Ssr2	-1.34	0.009
1443495_at	ATP synthase, H+ transporting, mitochondrial F0 complex, subunit f, isoform 2	Atp5j2	-1.34	0.002
1436405_at	Dedicator of cytokinesis 4	Dock4	-1.34	0.009
1417177_at	galactokinase 1	Galk1	-1.34	0.002
1454852_at	trans-acting transcription factor 1	Sp1	-1.34	0.002
1416952_at	ATPase, H+ transporting, V1 subunit D	Atp6v1d	-1.34	0.004
1448287_at	RNA polymerase 1-3	Rpo1-3	-1.34	0.004
1421846_at	WD repeat and SOCS box-containing 2	Wsb2	-1.35	0.009
1428412_at	SM-11044 binding protein	MGI:1914262	-1.35	0.009
1452066_a_at	Nedd4 family interacting protein 2	Ndfip2	-1.35	0.000
1442660_at	Yippee-like 1 (Drosophila)	Ypel1	-1.35	0.007
1431232_a_at	MAX gene associated	Mga	-1.35	0.005
1424189_at	phosphatidylinositol glycan, classC	Pigc	-1.35	0.007
1426403_at	ARP1 actin-related protein 1 homolog B (yeast)	Actr1b	-1.35	0.009
1416175_a_at	voltage-dependent anion channel 3	Vdac3	-1.35	0.009
1419812_s_at	DNA segment, Chr 11, ERATO Doi 99, expressed	D11Ertd99e	-1.35	0.003
1417891_at	T-complex expressed gene 1	Tce1	-1.35	0.003
1424381_at	splicing factor 4	Sf4	-1.35	0.008
1431037_a_at	ELAV (embryonic lethal, abnormal vision, Drosophila)-like 1 (Hu antigen R)	Elavl1	-1.35	0.006
1435764_a_at	gem (nuclear organelle) associated protein 7	Gemin7	-1.35	0.002
1415681_at	mitochondrial ribosomal protein L43	Mrpl43	-1.36	0.001
1423236_at	UDP-N-acetyl-alpha-D-galactosamine	Galnt1	-1.36	0.008
1416207_at	tafazzin	Taz	-1.36	0.004
1424160_at	asparagine-linked glycosylation 5 homolog	Alg5	-1.36	0.009

1460737_at	immunoglobulin (CD79A) binding protein 1	Igbp1	-1.36	0.006
1424469_a_at	cleavage and polyadenylation specific factor 4	Cpsf4	-1.36	0.004
1426821_at	component of oligomeric golgi complex 8	Cog8	-1.37	0.007
1417279_at	inositol 1,4,5-triphosphate receptor 1	Itpr1	-1.37	0.002
1423997_at	DNA segment, Chr 3, MJeffers 1	D3Jfr1	-1.37	0.000
1448659_at	caspase 7	Casp7	-1.37	0.004
1433562_s_at	ATP synthase, H+ transporting, mitochondrial F0 complex, subunit b, isoform 1	Atp5f1	-1.37	0.007
1438229_at	protein geranylgeranyltransferase type I, beta subunit	Pggt1b	-1.37	0.007
1416315_at	abhydrolase domain containing 4	Abhd4	-1.37	0.002
1417410_s_at	protein kinase C, iota	Prkci	-1.37	0.009
1454811_a_at	tumor differentially expressed 2	Tde2	-1.37	0.000
1449694_s_at	COMM domain containing 5	Commd5	-1.37	0.009
1426671_a_at	RNA-binding region (RNP1, RRM) containing 2	Rnpc2	-1.37	0.010
1423911_at	protein phosphatase 2, regulatory subunit B (B56), alpha isoform	Ppp2r5a	-1.37	0.007
1432367_a_at	ubiquitin fusion degradation 1 like	Ufd1l	-1.37	0.007
1428473_at	protein phosphatase 3, catalytic subunit, beta isoform	Ppp3cb	-1.38	0.009
1454036_a_at	ubiquitin specific protease 15	Usp15	-1.38	0.005
1423210_a_at	nucleolar protein family A, member 3	Nola3	-1.38	0.000
1417113_at	germ cell-less homolog (Drosophila)	Gcl	-1.38	0.000
1415882_at	growth hormone inducible transmembrane protein	Ghitm	-1.38	0.002
1416696_at	DNA segment, Chr 17, Wayne State University 104, expressed	D17Wsu104e	-1.38	0.006
1439184_s_at	thioredoxin-like 5	Txnl5	-1.38	0.005
1423743_at	archain 1	Arcn1	-1.38	0.002
1451731_at	ATP-binding cassette, sub-family A (ABC1), member 3	Abca3	-1.38	0.005
1455042_at	Transducin (beta)-like 1 X-linked	Tbl1x	-1.38	0.006
1451169_at	nodal modulator 1	Nomo1	-1.39	0.003
1422489_at	glucosidase 1	Gcs1	-1.39	0.009
1436027_at	oxysterol binding protein-like 11	Osbpl11	-1.39	0.010
1416194_at	cytochrome P450, family 4, subfamily b, polypeptide 1	Cyp4b1	-1.39	0.006
1416339_a_at	protein kinase C substrate 80K-H	Prkcsh	-1.39	0.006
1416197_at	U1 small nuclear ribonucleoprotein 1C	Snrp1c	-1.39	0.001
1425188_s_at	Sel1 (suppressor of lin-12) 1 homolog (C. elegans)	Sel1h	-1.39	0.006
1418506_a_at	peroxiredoxin 2	Prdx2	-1.39	0.001
1428645_at	guanine nucleotide binding protein, alpha inhibiting 3	Gnai3	-1.39	0.001
1415699_a_at	G protein pathway suppressor 1	Gps1	-1.39	0.003
1416278_a_at	ATP synthase, H+ transporting, mitochondrial F1 complex, O subunit	Atp5o	-1.39	0.001
1415818_at	annexin A6	Anxa6	-1.39	0.010
1423235_at	adaptor-related protein complex 3, beta 1 subunit	Ap3b1	-1.39	0.007
1417111_at	mannosidase 1, alpha	Man1a	-1.39	0.000
1419041_at	DNA segment, Chr 8, Wayne State University 49, expressed	D8Wsu49e	-1.39	0.008
1423873_at	LSM1 homolog, U6 small nuclear RNA associated (S. cerevisiae)	Lsm1	-1.40	0.007
1416291_at	proteasome (prosome, macropain) 26S subunit, ATPase, 4	Psmc4	-1.40	0.001
1455403_at	mannosidase, endo-alpha	Manea	-1.40	0.006
1417515_at	U7 snRNP-specific Sm-like protein LSM10	Lsm10	-1.40	0.003
1434251_at	CCR4-NOT transcription complex, subunit 1	Cnot1	-1.40	0.001
1426275_a_at	UDP-glucuronate decarboxylase 1	Uxs1	-1.40	0.000
1422959_s_at	zinc finger protein 313	Zfp313	-1.40	0.001
1434865_a_at	exocyst complex component 7	Exoc7	-1.41	0.000
1420157_s_at	ATP-binding cassette, sub-family F (GCN20), member 1	Abcf1	-1.41	0.006
1448210_at	RAB1, member RAS oncogene family	Rab1	-1.41	0.001
1416142_at	ribosomal protein S6	Rps6 /// LOC214738 /// LOC384383	-1.41	0.004
1426662_at	cytidine monophospho-N-acetylneuraminic acid synthetase	Cmas	-1.41	0.007

1417202_s_at	ubiquitin-activating enzyme E1C	Ube1c	-1.41	0.009
1422715_s_at	acid phosphatase 1, soluble	Acp1	-1.41	0.003
1423609_a_at	mannoside acetylglucosaminyltransferase 1	Mgat1	-1.41	0.002
1433708_at	signal recognition particle 68	Srp68	-1.41	0.007
1420829_a_at	tyrosine 3-monooxygenase/tryptophan 5-monooxygenase activation protein	Ywhaq	-1.41	0.009
1447585_s_at	mitochondrial ribosomal protein S6	Mrps6	-1.41	0.005
1417998_at	telomerase binding protein, p23	MGI:1929282	-1.41	0.005
1421900_at	eukaryotic translation initiation factor 2 alpha kinase 1	Eif2ak1	-1.41	0.002
1460303_at	nuclear receptor subfamily 3, group C, member 1	Nr3c1	-1.42	0.002
1456039_at	sorting nexin 14	Snx14	-1.42	0.003
1416655_at	C1GALT1-specific chaperone 1	C1galt1c1	-1.42	0.009
1433830_at	heterogeneous nuclear ribonucleoprotein A2/B1	Hnrpa2b1	-1.42	0.000
1429534_a_at	inner membrane protein, mitochondrial	Immt	-1.42	0.002
1418844_at	disrupted in bipolar disorder 1 homolog (human)	Dibd1	-1.42	0.007
1453014_a_at	SEC31-like 1 (S. cerevisiae)	Sec31l1	-1.42	0.006
1454888_at	prefoldin 4	Pfdn4	-1.42	0.010
1422759_a_at	exportin 6	Xpo6	-1.42	0.000
1427245_at	ADP-ribosylation factor GTPase activating protein 1	Arfgap1	-1.42	0.001
1453406_a_at	RAB28, member RAS oncogene family	Rab28	-1.42	0.009
1450638_at	programmed cell death 5	Pdcd5	-1.42	0.009
1435921_at	DNA segment, Chr 8, ERATO Doi 587, expressed	D8Ertd587e	-1.43	0.010
1436950_at	v-crk sarcoma virus CT10 oncogene homolog (avian)-like	Crkl	-1.43	0.001
1451017_at	serologically defined breast cancer antigen 84	Sdbcag84	-1.43	0.001
1416938_at	coiled-coil-helix-coiled-coil-helix domain containing 1	Chchd1	-1.43	0.006
1454652_at	zinc finger protein 265	Zfp265	-1.43	0.001
1448672_a_at	zinc finger protein 289	Zfp289	-1.43	0.006
1448356_at	ubiquitin-conjugating enzyme E2D 2	Ube2d2	-1.43	0.000
1429528_at	RAE1 RNA export 1 homolog (S. pombe)	Rae1	-1.43	0.008
1460357_at	YTH domain family 2	Ythdf2	-1.44	0.004
1417193_at	superoxide dismutase 2, mitochondrial	Sod2	-1.44	0.004
1458509_at	Meiotic recombination 11 homolog A (S. cerevisiae)	Mre11a	-1.44	0.001
1417535_at	F-box only protein 25	Fbxo25	-1.44	0.010
1451362_at	RAB7, member RAS oncogene family-like 1	Rab7l1	-1.44	0.004
1426586_at	solute carrier family 25 (mitochondrial carrier oxoglutarate carrier), member 11	Slc25a11	-1.44	0.002
1416395_at	guanylate kinase 1	Guk1	-1.44	0.010
1435859_x_at	proteasome (prosome, macropain) 26S subunit, ATPase 2	Psmc2	-1.44	0.001
1424769_s_at	caldesmon 1 /// RIKEN cDNA C920027I18 gene	Cald1 /// C920027I18Rik	-1.44	0.002
1434515_at	nuclear receptor coactivator 1	Ncoa1	-1.44	0.009
1456686_at	Zinc finger homeobox 1a	Zfhx1a	-1.44	0.003
1452116_s_at	activating transcription factor 2	Atf2	-1.44	0.003
1434112_at	latrophilin 2	Lphn2	-1.45	0.000
1449940_a_at	eukaryotic translation initiation factor 2B, subunit 4 delta	Eif2b4	-1.45	0.009
1457268_at	DOT1-like, histone H3 methyltransferase (S. cerevisiae)	Dot1l	-1.45	0.009
1456430_at	DNA segment, Chr 3, ERATO Doi 789, expressed	D3Ertd789e	-1.45	0.002
1449839_at	caspase 3, apoptosis related cysteine protease	Casp3	-1.45	0.005
1452148_at	low density lipoprotein receptor-related protein associated protein 1	Lrpap1	-1.45	0.006
1436380_at	Cdc42 binding protein kinase alpha	Cdc42bpa	-1.45	0.003
1415967_at	NADH dehydrogenase (ubiquinone) flavoprotein 1	Ndufv1	-1.46	0.008
1419927_s_at	RAB interacting factor	Rabif	-1.46	0.005
1424120_at	ring finger protein 8	Rnf8	-1.46	0.008
1415743_at	histone deacetylase 5	Hdac5	-1.46	0.004
1448331_at	NADH dehydrogenase (ubiquinone) 1 beta subcomplex, 7	Ndufb7	-1.46	0.006

1433982_at	ubiquitin specific protease 28	Usp28	-1.46	0.008
1451462_a_at	interferon (alpha and beta) receptor 2	Ifnar2	-1.46	0.005
1429519_at	fucose-1-phosphate guanylyltransferase	Fpgt	-1.46	0.006
1426138_a_at	ubiquitin-conjugating enzyme E2, J2 homolog (yeast)	Ube2j2	-1.46	0.004
1415713_a_at	DEAD (Asp-Glu-Ala-Asp) box polypeptide 24	Ddx24	-1.47	0.010
1450431_a_at	neural precursor cell expressed, developmentally down-regulted gene 4	Nedd4	-1.47	0.004
1432016_a_at	isocitrate dehydrogenase 3 (NAD+) alpha	Idh3a	-1.47	0.001
1421816_at	glutathione reductase 1	Gsr	-1.47	0.000
1437151_at	ubiquitin specific protease 22	Usp22	-1.47	0.010
1451074_at	ring finger protein 13	Rnf13	-1.47	0.001
1416017_at	coatomer protein complex, subunit gamma	Copg	-1.47	0.007
1416524_at	speckle-type POZ protein	Spop	-1.47	0.003
1426456_a_at	Msx-interacting-zinc finger	Miz1	-1.47	0.001
1418198_a_at	transmembrane 9 superfamily member 1	Tm9sf1	-1.47	0.004
1428845_at	BCL2-associated transcription factor 1	Bclaf1	-1.47	0.000
1429103_at	translocase of outer mitochondrial membrane 22 homolog (yeast)	Tomm22	-1.48	0.005
1419519_at	insulin-like growth factor 1	Igf1	-1.48	0.009
1416366_at	NADH dehydrogenase (ubiquinone) 1, subcomplex unknown, 2	Ndufc2	-1.48	0.001
1416990_at	retinoid X receptor beta	Rxrb	-1.48	0.009
1418260_at	hormonally upregulated Neu-associated kinase	Hunk	-1.48	0.001
1452857_at	HCF-binding transcription factor Zhangfei	MGI:2675296	-1.48	0.007
1426084_a_at	hypothetical protein MGC6357	MGC6357	-1.48	0.004
1419375_at	WW domain binding protein 4	Wbp4	-1.48	0.002
1416510_at	mitochondrial ribosomal protein L4	Mrpl4	-1.48	0.001
1451641_at	debranching enzyme homolog 1 (S. cerevisiae)	Dbr1	-1.48	0.000
1429218_at	de-etiolated homolog 1 (Arabidopsis)	Det1	-1.49	0.001
1416472_at	synapse associated protein 1	Syap1	-1.49	0.004
1434022_at	zinc finger and BTB domain containing 33	Zbtb33	-1.49	0.007
1415956_a_at	PCTAIRE-motif protein kinase 1	Pctk1	-1.49	0.000
1454699_at	sestrin 1	Sesn1	-1.49	0.003
1426457_at	sarcolemma associated protein	Slmap	-1.49	0.000
1433593_at	yippee-like 5 (Drosophila)	Ypel5	-1.49	0.007
1455286_at	BTB (POZ) domain containing 1	Btbd1	-1.49	0.003
1417398_at	related RAS viral (r-ras) oncogene homolog 2	Rras2	-1.49	0.001
1434435_s_at	cytochrome c oxidase, subunit XVII assembly protein homolog (yeast)	Cox17	-1.49	0.003
1433574_at	cell division cycle 37 homolog (S. cerevisiae)-like 1	Cdc37l1	-1.49	0.000
1438984_x_at	proteasome (prosome, macropain) subunit, beta type 4	Psmb4	-1.50	0.002
1434721_at	DNA segment, Chr 5, ERATO Doi 585, expressed	D5Ertd585e	-1.50	0.004
1416317_a_at	Stam binding protein	Stambp	-1.50	0.001
1417967_at	MMS19 (MET18 S. cerevisiae)-like	Mms19l	-1.50	0.004
1426288_at	low density lipoprotein receptor-related protein 4	Lrp4	-1.50	0.002
1448670_at	ubiquitin-conjugating enzyme E2E 3, UBC4/5 homolog (yeast)	Ube2e3	-1.50	0.008
1418295_s_at	diacylglycerol O-acyltransferase 1	Dgat1	-1.50	0.007
1417665_a_at	cleavage and polyadenylation specific factor 1	Cpsf1	-1.50	0.003
1423346_at	degenerative spermatocyte homolog 1 (Drosophila)	Degs1	-1.50	0.005
1452052_s_at	eukaryotic translation initiation factor 3, subunit 1 alpha	Eif3s1	-1.50	0.007
1418501_a_at	oxidation resistance 1	Oxr1	-1.50	0.001
1451312_at	NADH dehydrogenase (ubiquinone) Fe-S protein 7	Ndufs7	-1.50	0.004
1433886_at	eukaryotic translation initiation factor 2B, subunit 5 epsilon	Eif2b5	-1.50	0.003
1428949_at	exportin, tRNA (nuclear export receptor for tRNAs)	Xpot	-1.50	0.007
1448629_at	Hermansky-Pudlak syndrome 4 homolog (human)	Hps4	-1.50	0.010
1440490_at	Membrane protein, palmitoylated 6 (MAGUK p55 subfamily member 6)	Mpp6	-1.50	0.005
1449929_at	t-complex-associated-testis-expressed 1-like	Tcte1l	-1.51	0.000
1415756_a_at	SNAP-associated protein	Snapap	-1.51	0.007
1450893_a_at	ubiquitin-associated protein 1	Ubap1	-1.51	0.005

1434117_at	transcription elongation factor B (SIII), polypeptide 3	Tceb3	-1.51	0.001
1435768_at	AT rich interactive domain 4B (Rbp1 like)	Arid4b	-1.51	0.009
1427098_at	WW domain containing E3 ubiquitin protein ligase 1	Wwp1	-1.51	0.009
1423727_at	cornichon homolog (Drosophila)	Cnih	-1.51	0.000
1434879_at	cell division cycle 34 homolog (S. cerevisiae)	Cdc34	-1.51	0.006
1448434_at	ring finger protein 103	Rnf103	-1.51	0.006
1418566_s_at	NudC domain containing 2	Nudcd2	-1.51	0.005
1438832_x_at	DEAH (Asp-Glu-Ala-His) box polypeptide 30	Dhx30	-1.51	0.008
1450674_at	cyclin-dependent kinase 5	Cdk5	-1.51	0.002
1460652_at	Estrogen related receptor, alpha	Esrra	-1.51	0.009
1455493_at	synaptic nuclear envelope 1	Syne1	-1.51	0.001
1423204_at	transmembrane 9 superfamily protein member 4	Tm9sf4	-1.51	0.002
1424202_at	SEH1-like (S. cerevisiae	Seh1l	-1.52	0.006
1452749_at	PAP associated domain containing 1	Papd1	-1.52	0.000
1437350_at	candidate tumor suppressor OVCA2	MGI:2179725	-1.52	0.002
1455158_at	integrin alpha 3	Itga3	-1.52	0.002
1416170_at	TNF receptor-associated protein 1	Trap1	-1.52	0.005
1437533_at	Baculoviral IAP repeat-containing 4	Birc4	-1.52	0.007
1450701_a_at	general transcription factor II H, polypeptide 2	Gtf2h2	-1.52	0.005
1452044_at	actin related protein 2/3 complex, subunit 5-like	Arpc5l	-1.52	0.004
1450278_at	tachykinin receptor 3	Tacr3	-1.52	0.008
1428307_at	zinc finger, DHHC domain containing 13	Zdhhc13	-1.52	0.005
1417728_at	methyl-CpG binding domain protein 3	Mbd3	-1.52	0.006
1434884_at	Metadherin	Mtdh	-1.52	0.008
1443881_at	Protein O-fucosyltransferase 1	Pofut1	-1.52	0.007
1460326_at	phosphatidylinositol 3-kinase, catalytic, alpha polypeptide	Pik3ca	-1.52	0.003
1416721_s_at	splicing factor, arginine/serine-rich 6	Sfrs6	-1.52	0.009
1434311_at	CCR4-NOT transcription complex, subunit 6-like	Cnot6l	-1.52	0.009
1446110_at	DNA segment, Chr 3, ERATO Doi 711, expressed	D3Ertd711e	-1.53	0.009
1417624_at	Ngfi-A binding protein 1	Nab1	-1.53	0.001
1428405_at	host cell factor C1 regulator 1 (XPO1-dependent)	Hcfc1r1	-1.53	0.000
1460544_at	MAK10 homolog, amino-acid N-acetyltransferase subunit, (S. cerevisiae)	Mak10	-1.53	0.010
1434833_at	mitogen activated protein kinase kinase kinase kinase 2	Map4k2	-1.53	0.006
1424052_at	THAP domain containing 4	Thap4	-1.53	0.001
1454763_at	ankyrin repeat domain 17	Ankrd17	-1.53	0.008
1417361_at	ankyrin repeat and SOCS box-containing protein 3	Asb3	-1.53	0.008
1419984_s_at	zinc finger protein 644	Zfp644	-1.53	0.005
1417749_a_at	tight junction protein 1	Tjp1	-1.54	0.000
1451466_at	DNA segment, Chr 16, ERATO Doi 472, expressed	D16Ertd472e	-1.54	0.001
1421733_a_at	protein-tyrosine sulfotransferase 1	Tpst1	-1.54	0.009
1425473_at	cofactor required for Sp1 transcriptional activation, subunit 6	Crsp6	-1.54	0.005
1450062_a_at	melanoma antigen, family D, 1	Maged1	-1.54	0.001
1426453_at	pitrilysin metalloprotease 1	Pitrm1	-1.54	0.001
1418491_a_at	pseudouridine synthase 3	Pus3	-1.54	0.007
1440488_at	Protein tyrosine phosphatase, non-receptor type 4	Ptpn4	-1.54	0.007
1417296_at	activating transcription factor 1	Atf1	-1.54	0.001
1416377_at	programmed cell death protein 7	Pdcd7	-1.54	0.009
1433439_at	copine I	Cpne1	-1.55	0.000
1417418_s_at	cytochrome c oxidase, subunit VI a, polypeptide 1	Cox6a1	-1.55	0.003
1424280_at	motile sperm domain containing 1	Mospd1	-1.55	0.006
1447909_s_at	selenoprotein K	MGI:1931466	-1.55	0.001
1455113_at	armadillo repeat containing 8	Armc8	-1.55	0.000
1437009_a_at	zinc finger protein 364	Zfp364	-1.55	0.002

1436896_at	DNA segment, Chr 14, ERATO Doi 231, expressed	D14Ertd231e	-1.55	0.004
1425981_a_at	retinoblastoma-like 2	Rbl2	-1.55	0.002
1438677_at	plakophilin 4	Pkp4	-1.55	0.001
1429145_at	NHL repeat containing 2	Nhlrc2	-1.55	0.000
1433665_at	vacuolar protein sorting 41 (yeast)	Vps41	-1.55	0.008
1450504_a_at	1-acylglycerol-3-phosphate O-acyltransferase 3	Agpat3	-1.56	0.008
1416242_at	kelch-like 13 (Drosophila)	Klhl13	-1.56	0.003
1447679_s_at	BMS1-like, ribosome assembly protein (yeast)	Bms1l	-1.56	0.005
1416902_a_at	cytochrome c oxidase, subunit Vb	Cox5b	-1.56	0.001
1424235_at	ORM1-like 2 (S. cerevisiae)	Ormdl2	-1.56	0.005
1418335_a_at	diptheria toxin resistance protein required for diphthamide biosynthesis	Dph2l1	-1.56	0.000
1439788_at	hypothetical MGC86034	MGC86034	-1.56	0.003
1421032_a_at	DnaJ (Hsp40) homolog, subfamily B, member 12	Dnajb12	-1.56	0.004
1419641_at	purine rich element binding protein B	Purb	-1.56	0.008
1455728_at	phosphatase and tensin homolog	Pten	-1.56	0.007
1435010_at	ankyrin repeat and SOCS box-containing protein 7	Asb7	-1.56	0.001
1448320_at	stromal interaction molecule 1	Stim1	-1.57	0.000
1444731_at	Phosphodiesterase 4B, cAMP specific	Pde4b	-1.57	0.006
1455552_at	small nuclear RNA activating complex, polypeptide 4	Snapc4	-1.57	0.005
1454075_s_at	nudix (nucleoside diphosphate linked moiety X)-type motif 13	Nudt13	-1.57	0.001
1441038_at	Utrophin	Utrn	-1.57	0.004
1416479_a_at	transmembrane protein 14C	Tmem14c	-1.57	0.005
1416943_at	ubiquitin-conjugating enzyme E2E 1, UBC4/5 homolog (yeast)	Ube2e1	-1.57	0.001
1452211_at	proteasome (prosome, macropain) activator subunit 4	Psme4	-1.57	0.001
1422770_at	RAD51-like 3 (S. cerevisiae)	Rad51l3	-1.57	0.004
1451638_s_at	armadillo repeat containing 1	Armc1	-1.57	0.004
1426842_at	YTH domain family 3	Ythdf3	-1.57	0.003
1438647_x_at	centrin 2	Cetn2	-1.57	0.001
1455829_at	ubiquitin specific protease 14	Usp14	-1.57	0.004
1428385_at	membrane-associated ring finger (C3HC4) 8	Mert 8	-1.57	0.005
1433704_s_at	translocation protein 1	Tloc1	-1.57	0.008
1448829_at	SMC6 structural maintenance of chromosomes 6-like 1 (yeast)	Smc6l1	-1.58	0.009
1435702_s_at	tyrosine 3-monooxygenase/tryptophan 5-monooxygenase activation protein, epsilon polypeptide	Ywhae	-1.58	0.002
1426269_at	synaptobrevin like 1	Sybl1	-1.58	0.003
1451397_at	trinucleotide repeat containing 15	Tnrc15	-1.58	0.008
1426515_a_at	torsin family 1, member A (torsin A)	Tor1a	-1.58	0.003
1423191_at	formin binding protein 4	Fnbp4	-1.58	0.008
1440590_at	USP6 N-terminal like	Usp6nl	-1.58	0.003
1416478_a_at	malate dehydrogenase 2, NAD (mitochondrial)	Mdh2	-1.58	0.002
1415672_at	golgi autoantigen, golgin subfamily a, 7	Golga7	-1.58	0.004
1427406_at	thyroid hormone receptor interactor 11	Trip11	-1.58	0.004
1434135_at	UDP-GalNAc:betaGlcNAc beta 1,3-galactosaminyltransferase, polypeptide 2	B3galnt2	-1.58	0.004
1433622_at	gem (nuclear organelle) associated protein 4	Gemin4	-1.58	0.005
1436113_a_at	Suppression of tumorigenicity 13	St13	-1.59	0.001
1417771_a_at	proteasome (prosome, macropain) 26S subunit, ATPase, 6	Psmc6	-1.59	0.005
1423969_at	nucleoporin 37	Nup37	-1.59	0.003
1448391_at	RAB9, member RAS oncogene family	Rab9	-1.59	0.003
1434674_at	lysosomal trafficking regulator	Lyst	-1.59	0.008
1428702_at	DEAD (Asp-Glu-Ala-Asp) box polypeptide 28	Ddx28	-1.59	0.002
1456807_at	protein phosphatase 1, regulatory (inhibitor) subunit 3E	Ppp1r3e	-1.59	0.001
1422622_at	nitric oxide synthase 3, endothelial cell	Nos3	-1.59	0.000
1460602_at	Deleted in liver cancer 1	Dlc1	-1.59	0.003
1417509_at	ring finger protein (C3HC4 type) 19	Rnf19	-1.59	0.002
1455556_at	Notch gene homolog 2 (Drosophila)	Notch2	-1.59	0.003

1423916_s_at	myeloid leukemia factor 2	Mlf2	-1.59	0.000
1417611_at	transmembrane protein 37	Tmem37	-1.59	0.001
1433723_s_at	Small EDRK-rich factor 2	Serf2	-1.59	0.002
1450395_at	solute carrier family 22 (organic cation transporter), member 5	Slc22a5	-1.60	0.007
1417099_at	Ftsj homolog 1 (E. coli)	Ftsj1	-1.60	0.009
1438951_x_at	nucleoporin 54	Nup54	-1.60	0.001
1450385_at	karyopherin (importin) alpha 3	Kpna3	-1.60	0.009
1451214_at	kelch repeat and BTB (POZ) domain containing 2	Kbtbd2	-1.60	0.009
1456264_at	COMM domain containing 7	Commd7	-1.60	0.001
1426769_s_at	mastermind like 1 (Drosophila)	Maml1	-1.60	0.001
1422742_at	human immunodeficiency virus type I enhancer binding protein 1	Hivep1	-1.60	0.001
1455184_at	hypothetical protein B230364F10	B230364F10	-1.60	0.001
1423242_at	mitochondrial ribosomal protein S36	Mrps36	-1.60	0.000
1416173_at	pescadillo homolog 1, containing BRCT domain (zebrafish)	Pes1	-1.60	0.002
1423273_at	polymerase (DNA directed), gamma	Polg	-1.60	0.005
1424299_at	OMA1 homolog, zinc metallopeptidase (S. cerevisiae)	Oma1	-1.61	0.003
1422845_at	calnexin	Canx	-1.61	0.009
1441733_s_at	nucleoporin 153	Nup153	-1.61	0.006
1418597_at	topoisomerase (DNA) III alpha	Top3a	-1.61	0.010
1438631_x_at	tetratricopeptide repeat domain 13	Ttc13	-1.61	0.003
1417661_at	RAD52 homolog B (S. cerevisiae)	Rad52b	-1.61	0.002
1455873_a_at	vacuolar protein sorting 18 (yeast)	Vps18	-1.61	0.008
1433518_at	leucine carboxyl methyltransferase 2	Lcmt2	-1.61	0.005
1423355_at	synaptosomal-associated protein	Snap29	-1.61	0.002
1427150_at	myeloid/lymphoid or mixed-lineage leukemia 3	Mll3	-1.61	0.009
1447778_x_at	c6.1a protein	MGI:2389572	-1.61	0.003
1416604_at	cytochrome c-1	Cyc1	-1.61	0.001
1420843_at	protein tyrosine phosphatase, receptor type, F	Ptprf	-1.61	0.005
1422031_a_at	zinc finger, A20 domain containing 3	Za20d3	-1.61	0.008
1451151_s_at	zinc finger protein 410	Zfp410	-1.61	0.009
1419743_s_at	coactivator-associated arginine methyltransferase 1	Carm1	-1.61	0.005
1429587_at	DNA segment, Chr 8, ERATO Doi 233, expressed	D8Ertd233e	-1.61	0.005
1418526_at	FUS interacting protein (serine-arginine rich) 1	Fusip1	-1.62	0.007
1442240_at	SH2 domain binding protein 1 (tetratricopeptide repeat containing)	Sh2bp1	-1.62	0.002
1452490_a_at	adaptor protein complex AP-2, alpha 2 subunit	Ap2a2	-1.62	0.000
1423894_a_at	DALR anticodon binding domain containing 3	Dalrd3	-1.62	0.010
1422796_at	prolyl endopeptidase	Prep	-1.62	0.000
1460354_a_at	mitochondrial ribosomal protein L13	Mrpl13	-1.62	0.006
1437769_at	Epidermal growth factor receptor pathway substrate 15, related sequence	Eps15-rs	-1.62	0.008
1423716_s_at	ATP synthase, H+ transporting, mitochondrial F1 complex, delta subunit	Atp5d	-1.62	0.001
1442321_at	COMM domain containing 2	Commd2	-1.62	0.004
1419300_at	FMS-like tyrosine kinase 1	Flt1	-1.62	0.005
1436562_at	DEAD (Asp-Glu-Ala-Asp) box polypeptide 58	Ddx58	-1.62	0.009
1418819_at	ADP-ribosylation factor-like 10C	Arl10c	-1.63	0.005
1438045_at	Early endosome antigen 1	Eea1	-1.63	0.001
1452774_at	heterogeneous nuclear ribonucleoprotein A3	Hnrpa3	-1.63	0.005
1429048_at	biogenesis of lysosome-related organelles complex-1, subunit 2	Bloc1s2	-1.63	0.000
1449086_at	ring finger protein 141	Rnf141	-1.63	0.001
1450968_at	ubiquinol-cytochrome c reductase, Rieske iron-sulfur polypeptide 1	Uqcrfs1	-1.63	0.000
1426052_at	mutL homolog 3 (E coli)	Mlh3	-1.63	0.006
1436684_a_at	RIO kinase 2 (yeast)	Riok2	-1.63	0.004
1416071_at	DEAD (Asp-Glu-Ala-Asp) box polypeptide 18	Ddx18	-1.63	0.004
1428087_at	dynamin 1-like	Dnm1l	-1.63	0.005
1450898_at	hippocampus abundant gene transcript 1	Hiat1	-1.63	0.004

1423038_at	syntaxin 6	Stx6	-1.63	0.007
1454765_at	similar to General transcription factor 3C polypeptide 3	LOC227109	-1.63	0.006
1419452_at	ubiquitin carboxyl-terminal esterase L5	Uchl5	-1.63	0.007
1423599_a_at	phosducin-like	Pdcl	-1.64	0.002
1426969_at	tripartite motif protein 23	Trim23	-1.64	0.004
1421870_at	tripartite motif-containing 44	Trim44	-1.64	0.002
1455664_at	Reticulon 4 receptor-like 1	Rtn4rl1	-1.64	0.002
1424345_s_at	ubiquitin-conjugating enzyme E2M (UBC12 homolog, yeast)	Ube2m	-1.64	0.002
1423082_at	Der1-like domain family, member 1	Derl1	-1.64	0.010
1457627_x_at	ropporin 1-like	Ropn1l	-1.64	0.008
1428669_at	brain expressed myelocytomatosis oncogene	Bmyc	-1.64	0.000
1416567_s_at	ATP synthase, H+ transporting, mitochondrial F1 complex, epsilon subunit	Atp5e	-1.65	0.001
1433486_at	chloride channel 3	Clcn3	-1.65	0.001
1436739_at	angiotensin receptor 1	Agtr1	-1.65	0.002
1428877_at	signal recognition particle 72	Srp72	-1.65	0.000
1423502_at	bromodomain containing 2	Brd2	-1.65	0.004
1427558_s_at	asparagine-linked glycosylation 12 homolog (yeast, alpha-1,6-mannosyltransferase)	Alg12	-1.65	0.002
1417847_at	Unc-51 like kinase 2 (C. elegans)	Ulk2	-1.65	0.002
1457478_at	Spermatid perinuclear RNA binding protein	Strbp	-1.65	0.003
1416198_at	TH1-like homolog (Drosophila)	Th1l	-1.65	0.003
1423680_at	fatty acid desaturase 1	Fads1	-1.65	0.008
1415737_at	riboflavin kinase	Rfk	-1.65	0.001
1420862_at	dynactin 4	Dctn4	-1.65	0.001
1428368_at	Rho GTPase activating protein 21	Arhgap21	-1.65	0.000
1419042_at	interferon inducible GTPase 1	Iigp1	-1.65	0.002
1433606_at	MAD homolog 4 interacting transcription coactivator 1	Mitc1	-1.66	0.004
1426504_a_at	ring finger protein 121	Rnf121	-1.66	0.000
1438242_at	ubiquitin specific protease 40	Usp40	-1.66	0.007
1426976_at	ubiquitin specific protease 47	Usp47	-1.66	0.002
1442059_at	fragile X mental retardation gene 1, autosomal homolog	Fxr1h	-1.66	0.000
1435965_at	CCR4-NOT transcription complex, subunit 3	Cnot3	-1.66	0.005
1423046_s_at	nuclear cap binding protein subunit 2	Ncbp2	-1.66	0.006
1423738_at	oxidase assembly 1-like	Oxa1l	-1.66	0.007
1448967_at	nipsnap homolog 3A (C. elegans)	Nipsnap3a	-1.66	0.003
1449845_a_at	Eph receptor B4	Ephb4	-1.66	0.004
1448709_at	AT rich interactive domain 1A (Swi1 like)	Arid1a	-1.66	0.005
1422819_at	mitochondrial ribosomal protein L36	Mrpl36	-1.66	0.002
1433592_at	calmodulin 1	Calm1	-1.66	0.005
1419165_at	zinc finger protein 260	Zfp260	-1.66	0.002
1424021_at	ADP-ribosylation factor-like 6 interacting protein 6	Arl6ip6	-1.66	0.002
1421025_at	1-acylglycerol-3-phosphate O-acyltransferase 1	Agpat1	-1.66	0.000
1417457_at	CDC28 protein kinase regulatory subunit 2	Cks2	-1.66	0.005
1454826_at	Zinc finger and BTB domain containing 11	Zbtb11	-1.66	0.002
1434796_at	vesicle-associated membrane protein 4	Vamp4	-1.66	0.000
1425676_a_at	elongation of very long chain fatty acids (FEN1/Elo2, SUR4/Elo3, yeast)-like 1	Elovl1	-1.66	0.006
1418503_at	heat shock protein 9A	Hspa9a	-1.66	0.001
1423960_at	gene rich cluster, C3f gene	Grcc3f	-1.67	0.009
1434934_at	ATP synthase mitochondrial F1 complex assembly factor 1	Atpaf1	-1.67	0.002
1416531_at	glutathione S-transferase omega 1	Gsto1	-1.67	0.009
1423769_at	pentatricopeptide repeat domain 2	Ptcd2	-1.67	0.009
1423159_at	dihydrolipoamide dehydrogenase	Dld	-1.67	0.002
1456542_s_at	glutaminyl-tRNA synthase (glutamine-hydrolyzing)-like 1	Qrsl1	-1.67	0.002
1416984_at	mitochondrial ribosomal protein S18A	Mrps18a	-1.67	0.000
1426688_at	succinate dehydrogenase complex, subunit A, flavoprotein (Fp)	Sdha	-1.67	0.005

1438890_at	F-box and leucine-rich repeat protein 11	Fbxl11	-1.67	0.001
1429453_a_at	mitochondrial ribosomal protein L55	Mrpl55	-1.67	0.008
1417480_at	f-box only protein 9	Fbxo9	-1.67	0.008
1455605_at	DNA segment, Chr 5, Brigham & Women's Genetics 0860 expressed	D5Bwg0860e	-1.67	0.004
1430849_a_at	cell growth regulator with ring finger domain 1	Cgrrf1	-1.67	0.005
1454958_at	glycogen synthase kinase 3 beta	Gsk3b	-1.67	0.009
1426796_at	Taxilin	Txln	-1.67	0.008
1433481_at	FK506 binding protein 14	Fkbp14	-1.67	0.001
1423749_s_at	RAN GTPase activating protein 1	Rangap1	-1.67	0.002
1415688_at	ubiquitin-conjugating enzyme E2G 1	Ube2g1	-1.67	0.002
1416547_at	NADH dehydrogenase (ubiquinone) 1 beta subcomplex 3	Ndufb3	-1.67	0.008
1455483_at	zinc finger protein 148	Zfp148	-1.67	0.007
1417931_at	N-deacetylase/N-sulfotransferase (heparan glucosaminyl) 2	Ndst2	-1.67	0.002
1425143_a_at	NADH dehydrogenase (ubiquinone) Fe-S protein 1	Ndufs1	-1.67	0.010
1422609_at	cAMP-regulated phosphoprotein 19	Arpp19	-1.68	0.008
1422661_at	lectin, galactose binding, soluble 8	Lgals8	-1.68	0.007
1450422_a_at	KDEL (Lys-Asp-Glu-Leu) containing 1	Kdelc1	-1.68	0.001
1448796_s_at	transforming growth factor beta regulated gene 4	Tbrg4	-1.68	0.006
1421055_at	large tumor suppressor 2 /// exportin 4	Lats2 /// Xpo4	-1.68	0.010
1418843_at	solute carrier family 30 (zinc transporter), member 4	Slc30a4	-1.68	0.003
1420139_s_at	HIV-1 Rev binding protein 2	Hrb2	-1.68	0.001
1437698_at	myosin Vb	Myo5b	-1.68	0.002
1455659_at	transmembrane and coiled coil domains 1	Tmcc1	-1.68	0.001
1441397_at	Par-3 (partitioning defective 3) homolog (C. elegans)	Pard3	-1.68	0.007
1456516_x_at	UDP-N-acetylglucosamine pyrophosphorylase 1	Uap1	-1.68	0.006
1418520_at	trans-golgi network protein	Tgoln1	-1.68	0.008
1454044_a_at	peroxisomal biogenesis factor 3	Pex3	-1.68	0.005
1422972_s_at	GCN5 general control of amino acid synthesis-like 2 (yeast)	Gcn5l2	-1.68	0.006
1426486_at	UBX domain containing 2	Ubxd2	-1.68	0.005
1422853_at	src homology 2 domain-containing transforming protein C1	Shc1	-1.68	0.010
1452432_at	tissue factor pathway inhibitor	Tfpi	-1.68	0.009
1434757_at	core-binding factor, runt domain, alpha subunit 2, translocated to, 2 homolog	Cbfa2t2h	-1.68	0.004
1439267_x_at	cytochrome c oxidase, subunit Va	Cox5a	-1.68	0.000
1429047_at	gene trap locus 7	Gtl7	-1.68	0.007
1460557_at	suppressor of var1, 3-like 1 (S. cerevisiae)	Supv3l1	-1.69	0.004
1423737_at	NADH dehydrogenase (ubiquinone) Fe-S protein 3	Ndufs3	-1.69	0.003
1452055_at	CTD small phosphatase 1	Ctdsp1	-1.69	0.005
1416500_at	SAC1 (supressor of actin mutations 1, homolog)-like (S. cerevisiae)	Sacm1l	-1.69	0.002
1416556_at	sarcoma amplified sequence	Sas	-1.69	0.000
1440225_at	G protein-coupled receptor 116	Gpr116	-1.69	0.007
1418768_at	optic atrophy 1 homolog (human)	Opa1	-1.69	0.002
1418659_at	circadian locomoter output cycles kaput	Clock	-1.69	0.004
1418913_at	betaine-homocysteine methyltransferase 2	Bhmt2	-1.69	0.010
1424889_at	nucleoporin like 2	Nupl2	-1.69	0.005
1450937_at	lin 7 homolog c (C. elegans)	Lin7c	-1.69	0.005
1447209_at	Forkhead box P1	Foxp1	-1.69	0.003
1431593_a_at	leukocyte receptor cluster (LRC) member 5	Leng5	-1.69	0.007
1453804_a_at	origin recognition complex, subunit 4-like (S. cerevisiae)	Orc4l	-1.69	0.002
1434706_at	valosin containing protein (p97)/p47 complex interacting protein 1	Vcpip1	-1.69	0.009
1443199_at	Leucine-rich repeats and calponin homology (CH) domain containing 3	Lrch3	-1.69	0.009
1454895_at	cytochrome b, ascorbate dependent 3	Cybasc3	-1.70	0.007
1449518_at	glutaminyl-peptide cyclotransferase-like	Qpctl	-1.70	0.005
1455228_at	Wolf-Hirschhorn syndrome candidate 1 (human)	Whsc1	-1.70	0.007
1416349_at	mitochondrial ribosomal protein L34	Mrpl34	-1.70	0.001

1444318_at	Coiled-coil-helix-coiled-coil-helix domain containing 7	Chchd7	-1.70	0.008
1452615_s_at	tRNA splicing 2' phosphotransferase 1 homolog (S. cerevisiae)	Tpt1h	-1.70	0.002
1454695_at	WD repeat domain 18	Wdr18	-1.70	0.006
1421139_a_at	zinc finger protein 386 (Kruppel-like)	Zfp386	-1.70	0.006
1452692_a_at	NADH dehydrogenase (ubiquinone) flavoprotein 2	Ndufv2	-1.70	0.001
1420711_a_at	peroxisomal membrane protein 3	Pxmp3	-1.70	0.001
1431125_a_at	threonyl-tRNA synthetase-like 1	Tarsl1	-1.70	0.003
1429046_at	SMAD specific E3 ubiquitin protein ligase 2	Smurf2	-1.70	0.001
1416465_a_at	vesicle-associated membrane protein, associated protein A	Vapa	-1.71	0.008
1422433_s_at	isocitrate dehydrogenase 1 (NADP+), soluble	Idh1	-1.71	0.005
1415757_at	golgi-specific brefeldin A-resistance factor 1	Gbf1	-1.71	0.002
1435757_a_at	ubiquinol cytochrome c reductase core protein 2	Uqcrc2	-1.71	0.010
1460648_at	nuclear receptor subfamily 2, group F, member 6	Nr2f6	-1.71	0.002
1433603_at	NADH dehydrogenase (ubiquinone) Fe-S protein 6	Ndufs6	-1.71	0.009
1422752_at	polymerase (RNA) III (DNA directed) polypeptide K	Polr3k	-1.71	0.004
1449157_at	nuclear receptor subfamily 2, group C, member 1	Nr2c1	-1.71	0.010
1426046_a_at	Rab geranylgeranyl transferase, a subunit	Rabggta	-1.71	0.000
1434119_at	DNA segment, Chr 2, Wayne State University 81, expressed	D2Wsu81e	-1.71	0.005
1423581_at	N-myristoyltransferase 2	Nmt2	-1.71	0.005
1420839_at	pleckstrin homology domain-containing, family A member 3	Plekha3	-1.71	0.003
1424238_at	sirtuin 7 (silent mating type information regulation 2, homolog) 7 (S. cerevisiae)	Sirt7	-1.71	0.000
1415767_at	YTH domain family 1	Ythdf1	-1.72	0.010
1417582_s_at	dihydroorotate dehydrogenase	Dhodh	-1.72	0.002
1427929_a_at	pyridoxal (pyridoxine, vitamin B6) kinase	Pdxk	-1.72	0.001
1429914_at	Enhancer of polycomb homolog 1 (Drosophila)	Epc1	-1.72	0.003
1424224_at	ankyrin repeat and SOCS box-containing protein 8	Asb8	-1.72	0.008
1449441_a_at	WW domain binding protein 1	Wbp1	-1.72	0.003
1427680_a_at	nuclear factor I/B	Nfib	-1.72	0.003
1416346_at	translocase of inner mitochondrial membrane 8 homolog a (yeast)	Timm8a	-1.72	0.003
1448963_at	nuclear transcription factor-Y gamma	Nfyc	-1.72	0.009
1451244_a_at	zinc finger protein 422	Zfp422	-1.72	0.007
1417301_at	frizzled homolog 6 (Drosophila)	Fzd6	-1.72	0.006
1433613_at	Pantothenate kinase 3	Pank3	-1.72	0.002
1426654_at	zinc finger, C3HC type 1	Zc3hc1	-1.72	0.000
1423925_at	DEAH (Asp-Glu-Ala-His) box polypeptide 16	Dhx16	-1.72	0.004
1435001_at	phospholipase A2, activating protein	Plaa	-1.72	0.008
1436390_a_at	Mid-1-related chloride channel 1	Mclc	-1.72	0.008
1456689_at	Ring finger protein 10	Rnf10	-1.72	0.008
1451271_a_at	acetyl-Coenzyme A acetyltransferase 1	Acat1	-1.72	0.004
1460704_at	radical fringe gene homolog (Drosophila)	Rfng	-1.72	0.005
1431252_a_at	zinc finger protein 655	Zfp655	-1.72	0.001
1434056_a_at	gene model 137, (NCBI)	Gm137	-1.72	0.001
1437546_at	DnaJ (Hsp40) homolog, subfamily C, member 14	Dnajc14	-1.72	0.009
1437630_at	DNA segment, Chr 16, Brigham & Women's Genetics 1547 expressed	D16Bwg1547e	-1.72	0.002
1437838_x_at	G-rich RNA sequence binding factor 1	Grsf1	-1.72	0.006
1417350_at	pallidin	Pldn	-1.72	0.004
1455432_at	TAO kinase 1	Taok1	-1.72	0.003
1419176_at	DNA segment, Chr 8, ERATO Doi 531, expressed	D8Ertd531e	-1.72	0.008
1431694_a_at	catenin beta interacting protein 1	Catnbip1	-1.72	0.002
1417264_at	DNA segment, Chr 5, ERATO Doi 33, expressed	D5Ertd33e	-1.73	0.004
1447961_s_at	mitochondrial ribosomal protein L38	Mrpl38	-1.73	0.006
1449190_a_at	ectonucleoside triphosphate diphosphohydrolase 4	Entpd4	-1.73	0.003
1455420_at	RAD23b homolog (S. cerevisiae)	Rad23b	-1.73	0.006
1437357_at	YTH domain containing 2	Ythdc2	-1.73	0.001

1417502_at	tetraspanin 7	Tspan7	-1.73	0.007
1417129_a_at	myeloid ecotropic viral integration site-related gene 1	Mrg1	-1.73	0.009
1449027_at	ras homolog gene family, member U	Rhou	-1.73	0.008
1424057_at	ganglioside-induced differentiation-associated-protein 2	Gdap2	-1.73	0.000
1417086_at	platelet-activating factor acetylhydrolase, isoform 1b, beta1 subunit	Pafah1b1	-1.73	0.000
1448916_at	v-maf musculoaponeurotic fibrosarcoma oncogene family, protein G (avian)	Mafg	-1.73	0.003
1419499_at	Glycerol-3-phosphate acyltransferase, mitochondrial	Gpam	-1.73	0.006
1419369_at	ring finger protein 138	Rnf138	-1.73	0.002
1450994_at	Rho-associated coiled-coil forming kinase 1	Rock1	-1.73	0.006
1418694_at	potassium channel modulatory factor 1	Kcmf1	-1.73	0.009
1436554_at	zinc finger protein, subfamily 1A, 5	Zfpn1a5	-1.74	0.001
1420631_a_at	bladder cancer associated protein homolog (human)	Blcap	-1.74	0.001
1451259_at	small fragment nuclease	Smfn	-1.74	0.007
1452660_s_at	kelch-like 7 (Drosophila)	Klhl7	-1.74	0.001
1419068_at	RAB guanine nucleotide exchange factor (GEF) 1	Rabgef1	-1.74	0.000
1435235_at	thioredoxin-like 1	Txnl1	-1.74	0.001
1454899_at	LIM domain containing preferred translocation partner in lipoma	Lpp	-1.74	0.008
1444046_at	Hypothetical protein D430041B17	D430041B17	-1.74	0.009
1420966_at	solute carrier family 25 (mitochondrial carrier ornithine transporter), member 15	Slc25a15	-1.74	0.010
1455285_at	solute carrier family 31, member 1	Slc31a1	-1.74	0.009
1422577_at	citrate synthase	Cs	-1.74	0.000
1418861_at	protein inhibitor of activated STAT, 4	Pias4	-1.74	0.006
1417933_at	insulin-like growth factor binding protein 6	Igfbp6	-1.74	0.000
1418655_at	UDP-N-acetyl-alpha-D-galactosamine	Galgt1	-1.74	0.003
1418380_at	telomeric repeat binding factor 1	Terf1	-1.74	0.004
1455816_a_at	potassium channel tetramerisation domain containing 3	Kctd3	-1.74	0.010
1426585_s_at	mitogen activated protein kinase 1	Mapk1	-1.74	0.004
1430982_at	splicing factor, arginine/serine-rich 1 (ASF/SF2)	Sfrs1	-1.74	0.001
1451504_at	coiled-coil-helix-coiled-coil-helix domain containing 3	Chchd3	-1.74	0.008
1434355_at	Similar to hypothetical protein FLJ38281	Zfp617	-1.75	0.006
1460164_at	spindlin	Spin	-1.75	0.003
1434860_at	NMDA receptor-regulated gene 3	Narg3	-1.75	0.001
1448009_at	UDP-glucose ceramide glucosyltransferase-like 1	Ugcgl1	-1.75	0.005
1457332_at	heterogeneous nuclear ribonucleoprotein methyltransferase-like 3 (S. cerevisiae)	Hrmt1l3	-1.75	0.009
1426437_s_at	histone deacetylase 3	Hdac3	-1.75	0.007
1437391_x_at	mitochondrial ribosomal protein L44	Mrpl44	-1.75	0.009
1426296_at	RAD52 homolog (S. cerevisiae)	Rad52	-1.75	0.001
1435208_at	deltex 3-like (Drosophila)	Dtx3l	-1.75	0.004
1440766_at	Guanine nucleotide binding protein-like 3 (nucleolar)	C77032	-1.75	0.008
1427177_at	FYVE and coiled-coil domain containing 1	Fyco1	-1.75	0.002
1434834_at	suppressor of cytokine signaling 7	Socs7	-1.75	0.008
1450759_at	bone morphogenetic protein 6	Bmp6	-1.75	0.002
1425865_a_at	ligase III, DNA, ATP-dependent	Lig3	-1.76	0.004
1455058_at	myotubularin related protein 9	Mtmr9	-1.76	0.007
1451102_at	CCR4-NOT transcription complex, subunit 8	Cnot8	-1.76	0.005
1459942_at	Open reading frame 34	ORF34	-1.76	0.001
1427362_x_at	Homeo box C6	Hoxc6	-1.76	0.008
1416347_at	multiple endocrine neoplasia 1	Men1	-1.76	0.000
1452048_at	mitochondrial ribosomal protein L12	Mrpl12	-1.76	0.001
1448760_at	zinc finger protein 68	Zfp68	-1.76	0.002
1433660_at	DNA segment, Chr 2, ERATO Doi 435, expressed	D2Ertd435e	-1.76	0.006
1434017_at	zinc and ring finger 2	Znrf2	-1.76	0.001
1415682_at	exportin 7	Xpo7	-1.76	0.007

1429514_at	phosphatidic acid phosphatase type 2B	Ppap2b	-1.76	0.010
1423912_at	alveolar soft part sarcoma chromosome region, candidate 1 (human)	Aspscr1	-1.77	0.004
1423114_at	ubiquitin-conjugating enzyme E2D 3 (UBC4/5 homolog, yeast)	Ube2d3	-1.77	0.010
1428615_at	purinergic receptor (family A group 5)	P2y5	-1.77	0.000
1423142_a_at	GTP binding protein 4	Gtpbp4	-1.77	0.002
1423938_at	lethal giant larvae homolog 2	Llglh2	-1.77	0.003
1450031_at	lymphoid nuclear protein related to AF4-like	Laf4l	-1.77	0.007
1435403_at	Similar to Lipogenin	---	-1.77	0.009
1424329_a_at	proline-rich Gla (G-carboxyglutamic acid) polypeptide 2	Prrg2	-1.77	0.005
1434128_a_at	zinc finger protein 574	Zfp574	-1.77	0.006
1439367_x_at	ADP-ribosylation factor 4	Arf4	-1.77	0.008
1429655_at	NudC domain containing 1	Nudcd1	-1.77	0.000
1460216_at	acyl-Coenzyme A dehydrogenase, short chain	Acads	-1.77	0.002
1418257_at	solute carrier family 12, member 7	Slc12a7	-1.77	0.000
1432164_a_at	glycine cleavage system protein H (aminomethyl carrier)	Gcsh	-1.77	0.005
1423753_at	BMP and activin membrane-bound inhibitor, homolog (Xenopus laevis)	Bambi	-1.77	0.008
1424794_at	ring finger protein 186	Rnf186	-1.78	0.007
1435266_at	hypothetical protein 9330112M16	9330112M16	-1.78	0.009
1420124_s_at	T-cell leukemia translocation altered gene	Tcta	-1.78	0.000
1438058_s_at	prostate tumor over expressed gene 1	Ptov1	-1.78	0.010
1451274_at	oxoglutarate dehydrogenase (lipoamide)	Ogdh	-1.78	0.000
1424842_a_at	Rho GTPase activating protein 24	Arhgap24	-1.78	0.009
1455964_at	DNA segment, Chr 11, ERATO Doi 752, expressed	D11Ertd752e	-1.78	0.002
1426748_s_at	ATP-binding cassette, sub-family F (GCN20), member 3	Abcf3	-1.78	0.006
1424426_at	methylthioadenosine phosphorylase	Mtap	-1.78	0.006
1456079_x_at	apurinic/apyrimidinic endonuclease 1	Apex1	-1.78	0.007
1418151_at	myotubularin related protein 4	Mtmr4	-1.78	0.005
1423901_at	thyroid hormone receptor interactor 12	Trip12	-1.79	0.007
1428322_a_at	NADH dehydrogenase (ubiquinone) 1 beta subcomplex, 10	Ndufb10	-1.79	0.004
1423200_at	nuclear receptor co-repressor 1	Ncor1	-1.79	0.002
1449137_at	pyruvate dehydrogenase E1 alpha 1	Pdha1	-1.79	0.010
1428716_at	peroxisome biogenesis factor 1	Pex1	-1.79	0.006
1423533_a_at	ras homolog gene family, member T1	Rhot1	-1.79	0.009
1454875_a_at	retinoblastoma binding protein 4	Rbbp4	-1.79	0.003
1415998_at	voltage-dependent anion channel 1	Vdac1	-1.79	0.000
1420903_at	ST6 sialyltransferase 3	St6galnac3	-1.79	0.006
1417351_a_at	small nuclear ribonucleoprotein polypeptide A'	Snrpa1	-1.79	0.001
1436518_at	ubiquitin specific protease 46	Usp46	-1.79	0.001
1448589_at	NADH dehydrogenase (ubiquinone) 1 beta subcomplex, 5	Ndufb5	-1.79	0.005
1451884_a_at	LSM2 homolog, U6 small nuclear RNA associated (S. cerevisiae)	Lsm2	-1.79	0.010
1429092_at	vitamin K epoxide reductase complex, subunit 1-like 1	Vkorc1l1	-1.79	0.009
1418693_at	heterogeneous nuclear ribonucleoprotein C	Hnrpc	-1.80	0.001
1415770_at	WD repeat domain 6	Wdr6	-1.80	0.004
1424622_at	heat shock factor 1	Hsf1	-1.80	0.005
1429067_at	calpain, small subunit 2	Capns2	-1.80	0.009
1451193_x_at	tetratricopeptide repeat domain 4	Ttc4	-1.80	0.005
1417505_s_at	interleukin 11 receptor, alpha chain 1 /// interleukin 11 receptor, alpha chain 2	Il11ra1 /// Il11ra2	-1.80	0.004
1441344_at	SPFH domain family, member 1	Spfh1	-1.80	0.005
1426438_at	DEAD (Asp-Glu-Ala-Asp) box polypeptide 3, Y-linked	Ddx3y	-1.80	0.008
1436665_a_at	latent transforming growth factor beta binding protein 4	Ltbp4	-1.80	0.001
1456019_at	CWF19-like 2, cell cycle control (S. pombe)	Cwf19l2	-1.80	0.001
1436161_at	androgen-induced proliferation inhibitor	Aprin	-1.80	0.004
1423278_at	protein tyrosine phosphatase, receptor type, K	Ptprk	-1.80	0.005
1433555_at	ELL associated factor 1	Eaf1	-1.80	0.005

1460694_s_at	supervillin	Svil	-1.80	0.000
1449510_at	zinc finger protein 467	Zfp467	-1.80	0.003
1417842_at	calcium modulating ligand	Caml	-1.81	0.007
1437850_a_at	cellular nucleic acid binding protein 1	Cnbp1	-1.81	0.006
1434516_at	phosphoseryl-tRNA kinase	Pstk	-1.81	0.004
1435276_a_at	DiGeorge syndrome critical region gene 2	Dgcr2	-1.81	0.006
1436759_x_at	calponin 3, acidic	Cnn3	-1.81	0.004
1456199_x_at	RAN binding protein 9	Ranbp9	-1.81	0.010
1455173_at	G1 to S phase transition 1	Gspt1	-1.81	0.005
1452439_s_at	splicing factor, arginine/serine-rich 2 (SC-35)	Sfrs2	-1.81	0.004
1419317_x_at	small proline rich-like 1	Sprrl1	-1.81	0.002
1416960_at	beta-1,3-glucuronyltransferase 3 (glucuronosyltransferase I)	B3gat3	-1.81	0.010
1442416_at	Hypothetical protein D930001B02	D930001B02	-1.81	0.002
1448563_at	prohibitin	Phb	-1.82	0.000
1433880_at	DnaJ (Hsp40) homolog, subfamily C, member 11	Dnajc11	-1.82	0.001
1427364_a_at	ornithine decarboxylase, structural 1	Odc1	-1.82	0.007
1427482_a_at	carbonic anhydrase 8	Car8	-1.82	0.009
1427471_at	F-box and leucine-rich repeat protein 3	Fbxl3	-1.82	0.006
1419095_a_at	apolipoprotein M	Apom	-1.82	0.008
1460249_at	ligand of numb-protein X 2	Lnx2	-1.82	0.001
1422123_s_at	CEA-related cell adhesion molecule 1 /// CEA-related cell adhesion molecule 2	Ceacam1 /// Ceacam2	-1.83	0.009
1432000_a_at	death effector domain-containing	Dedd	-1.83	0.006
1438934_x_at	semaphorin 4A	Sema4a	-1.83	0.009
1416751_a_at	DEAD (Asp-Glu-Ala-Asp) box polypeptide 20	Ddx20	-1.83	0.008
1448230_at	ubiquitin specific protease 10	Usp10	-1.83	0.007
1423611_at	alkaline phosphatase 2, liver	Akp2	-1.83	0.001
1457672_at	chromodomain helicase DNA binding protein 9	Chd9	-1.83	0.001
1442424_at	DNA segment, Chr 2, ERATO Doi 485, expressed	D2Ertd485e	-1.83	0.007
1426914_at	MARVEL (membrane-associating) domain containing 2	Marveld2	-1.83	0.004
1426837_at	methionyl aminopeptidase 1	Metap1	-1.83	0.003
1427505_a_at	CASP2 and RIPK1 domain containing adaptor with death domain	Cradd	-1.83	0.008
1426111_x_at	interferon regulatory factor 3	Irf3	-1.83	0.010
1417948_s_at	interleukin enhancer binding factor 2	Ilf2	-1.83	0.002
1417741_at	liver glycogen phosphorylase	Pygl	-1.83	0.005
1432004_a_at	dynamin 2	Dnm2	-1.84	0.001
1433745_at	triple functional domain (PTPRF interacting)	Trio	-1.84	0.008
1456727_a_at	casein kinase 1, delta	Csnk1d	-1.84	0.006
1449308_at	complement component 6	C6	-1.84	0.006
1457664_x_at	Complement component 2 (within H-2S)	C2	-1.84	0.010
1448376_at	Werner helicase interacting protein 1	Wrnip1	-1.84	0.009
1416832_at	solute carrier family 39 (metal ion transporter), member 8	Slc39a8	-1.84	0.003
1419047_at	pecanex homolog (Drosophila)	Pcnx	-1.84	0.004
1435167_at	RAN binding protein 6	Ranbp6	-1.84	0.010
1416259_at	peroxisomal biogenesis factor 12	Pex12	-1.84	0.001
1420870_at	myeloid/lymphoid or mixed lineage-leukemia translocation to 10 homolog	Mllt10	-1.84	0.009
1434598_at	DNA segment, Chr 13, Wayne State University 64, expressed	D13Wsu64e	-1.84	0.001
1425505_at	myosin, light polypeptide kinase	Mylk	-1.85	0.003
1418228_at	histone cell cycle regulation defective interacting protein 5	Hirip5	-1.85	0.001
1417251_at	palmdelphin	Palmd	-1.85	0.001
1438226_at	similar to hypothetical protein MGC955	BC035522	-1.85	0.003
1450969_at	propionyl Coenzyme A carboxylase, beta polypeptide	Pccb	-1.85	0.005
1452811_at	5-aminoimidazole-4-carboxamide ribonucleotide formyltransferase	Atic	-1.85	0.008
1443933_at	Membrane targeting (tandem) C2 domain containing 1	Mtac2d1	-1.85	0.005
1423733_a_at	Flt3 interacting zinc finger protein 1	Fiz1	-1.85	0.007

1450153_at	golgi associated PDZ and coiled-coil motif containing	Gopc	-1.85	0.002
1450966_at	carnitine O-octanoyltransferase	Crot	-1.85	0.002
1452781_a_at	general transcription factor IIIC, polypeptide 2, beta	Gtf3c2	-1.86	0.000
1431980_a_at	arsenic (+3 oxidation state) methyltransferase	As3mt	-1.86	0.004
1431105_a_at	transmembrane protein 33	Tmem33	-1.86	0.005
1422186_s_at	diaphorase 1 (NADH)	Dia1	-1.86	0.001
1427114_at	tetratricopeptide repeat domain 19	Ttc19	-1.86	0.008
1424584_a_at	RAN binding protein 10	Ranbp10	-1.86	0.006
1440856_at	mitogen activated protein kinase 8	Mapk8	-1.86	0.010
1417462_at	CAP, adenylate cyclase-associated protein 1 (yeast)	Cap1	-1.86	0.008
1422472_at	peroxisomal biogenesis factor 13	Pex13	-1.86	0.010
1430798_x_at	mitochondrial ribosomal protein L15	Mrpl15	-1.86	0.009
1426593_a_at	F-box only protein 22	Fbxo22	-1.86	0.006
1425419_a_at	v-raf-1 leukemia viral oncogene 1	Raf1	-1.86	0.000
1446091_at	Acidic nuclear phosphoprotein 32 family, member B	Anp32b	-1.86	0.007
1441214_at	synaptotagmin-like homologue lacking C2 domains b	MGI:2443248	-1.86	0.010
1417673_at	growth factor receptor bound protein 14	Grb14	-1.86	0.009
1442632_at	Centaurin, gamma 2	Centg2	-1.86	0.005
1427447_a_at	TRIO and F-actin binding protein	Triobp	-1.87	0.008
1423955_a_at	longevity assurance homolog 2 (S. cerevisiae)	Lass2	-1.87	0.004
AFFX-r2-Ec-bioD-3_at	dethiobiotin synthetase	bioD	-1.87	0.005
1455979_at	AT rich interactive domain 1B (Swi1 like)	Arid1b	-1.87	0.000
1426020_at	thymopoietin	Tmpo	-1.87	0.003
1437119_at	Endoplasmic reticulum (ER) to nucleus signalling 1	Ern1	-1.87	0.003
1418052_at	mevalonate kinase	Mvk	-1.87	0.004
1460578_at	FYVE, RhoGEF and PH domain containing 5	Fgd5	-1.87	0.003
1430546_at	crystallin, zeta (quinone reductase)-like 1	Cryzl1	-1.87	0.001
1438218_at	zinc finger and BTB domain containing 24	Zbtb24	-1.87	0.009
1434606_at	V-erb-b2 erythroblastic leukemia viral oncogene homolog 3 (avian)	Erbb3	-1.87	0.004
1452821_at	Tial1 cytotoxic granule-associated RNA binding protein-like 1	Tial1	-1.87	0.003
1455197_at	Rho family GTPase 1	Rnd1	-1.87	0.010
1460403_at	PC4 and SFRS1 interacting protein 1	Psip1	-1.87	0.003
1434087_at	5,10-methylenetetrahydrofolate reductase	Mthfr	-1.87	0.009
1432332_a_at	DNA segment, Chr 7, Roswell Park 2 complex, expressed	D7Rp2e	-1.87	0.000
1429806_at	Era (G-protein)-like 1 (E. coli)	Eral1	-1.87	0.003
1425699_a_at	abhydrolase domain containing 14A	Abhd14a	-1.87	0.001
1422102_a_at	signal transducer and activator of transcription 5B	Stat5b	-1.87	0.004
1435475_at	lectin, mannose-binding 2-like	Lman2l	-1.88	0.008
1416706_at	ribulose-5-phosphate-3-epimerase	Rpe	-1.88	0.000
1426757_at	adenosine monophosphate deaminase 2 (isoform L)	Ampd2	-1.88	0.006
1429005_at	malignant fibrous histiocytoma amplified sequence 1	Mfhas1	-1.88	0.004
1419359_at	cardiac lineage protein 1	MGI:2385923	-1.88	0.001
1456315_a_at	protein tyrosine phosphatase-like (proline instead of catalytic arginine), member a	Ptpla	-1.88	0.003
1429681_a_at	glycoprotein, synaptic 2	Gpsn2	-1.88	0.001
1443142_at	Beta-transducin repeat containing protein	Btrc	-1.88	0.000
1450090_at	zinc finger protein 101	Zfp101	-1.88	0.000
1416014_at	ATP-binding cassette, sub-family E (OABP), member 1	Abce1	-1.88	0.006
1416991_at	mitochondrial translation optimization 1 homolog (S. cerevisiae)	Mto1	-1.89	0.003
1451231_a_at	cullin 2	Cul2	-1.89	0.000
1428785_at	angiomotin-like 1	Amotl1	-1.89	0.003
1446674_at	Mitogen activated protein kinase kinase 5	Map2k5	-1.89	0.001
1452915_at	protein kinase, cAMP dependent regulatory, type II alpha	Prkar2a	-1.89	0.002

1451457_at	sterol-C5-desaturase (fungal ERG3, delta-5-desaturase) homolog (S. cerevisae)	Sc5d	-1.89	0.006
1451980_at	O-acetyltransferase	MGI:2384865	-1.89	0.008
1426770_at	peroxisome biogenesis factor 5	Pex5	-1.89	0.009
1416338_at	SH3-domain GRB2-like 1	Sh3gl1	-1.89	0.000
1451300_a_at	CHMP family, member 7	Chmp7	-1.89	0.002
1452384_at	ectonucleotide pyrophosphatase/phosphodiesterase 3	Enpp3	-1.89	0.010
1424423_at	lens epithelial protein /// RIKEN cDNA A930017E24 gene	Lenep /// A930017E24 Rik	-1.90	0.002
1429384_at	casein kinase 1, gamma 3	Csnk1g3	-1.90	0.010
1426055_a_at	phosphatidylinositol glycan, class Q	Pigq	-1.90	0.003
1428789_at	Ral GEF with PH domain and SH3 binding motif 2	Ralgps2	-1.90	0.005
1435474_at	TAF5 RNA polymerase II, TATA box binding protein (TBP)-associated factor	Taf5	-1.90	0.004
1435370_a_at	carboxylesterase 3	Ces3	-1.90	0.009
1421499_a_at	protein tyrosine phosphatase, non-receptor type 14	Ptpn14	-1.90	0.002
1456638_at	WD repeat domain 59	Wdr59	-1.90	0.008
1424980_s_at	anterior pharynx defective 1a homolog (C. elegans)	Aph1a	-1.90	0.002
1450762_s_at	zinc finger protein 191	Zfp191	-1.90	0.008
1435494_s_at	desmoplakin	Dsp	-1.90	0.002
1421478_a_at	zinc finger protein 318	Zfp318	-1.90	0.004
1416224_at	zinc finger and BTB domain containing 17	Zbtb17	-1.90	0.005
1416265_at	calpain 10	Capn10	-1.90	0.002
1450403_at	signal transducer and activator of transcription 2	Stat2	-1.91	0.002
1448609_at	thiosulfate sulfurtransferase, mitochondrial	Tst	-1.91	0.005
1419053_at	peroxisomal biogenesis factor 14	Pex14	-1.91	0.003
1451765_a_at	ectonucleoside triphosphate diphosphohydrolase 5	Entpd5	-1.91	0.008
1435630_s_at	acetyl-Coenzyme A acetyltransferase 2	Acat2	-1.91	0.008
1460035_at	B-cell receptor-associated protein 37	Bcap37	-1.91	0.009
1431335_a_at	WAP four-disulfide core domain 1	Wfdc1	-1.92	0.010
1459392_at	PHD finger protein 8	Phf8	-1.92	0.003
1422484_at	cytochrome c, somatic	Cycs	-1.92	0.001
1417010_at	zinc finger protein 238	Zfp238	-1.92	0.007
1423086_at	Niemann Pick type C1	Npc1	-1.92	0.009
1425537_at	protein phosphatase 1A, magnesium dependent, alpha isoform	Ppm1a	-1.93	0.007
1428918_at	SCY1-like 3 (S. cerevisiae)	Scyl3	-1.93	0.000
1435900_at	zinc finger protein 297B	Zfp297b	-1.93	0.007
1434002_at	checkpoint supressor 1	Ches1	-1.93	0.001
1435043_at	phospholipase C, beta 1	Plcb1	-1.93	0.004
1426023_a_at	rabaptin, RAB GTPase binding effector protein 1	Rabep1	-1.93	0.008
1435101_at	Der1-like domain family, member 2	Derl2	-1.93	0.002
1447926_at	ADP-ribosylation factor-like 5	Arl5	-1.93	0.004
1435481_at	zinc finger protein 653	Zfp653	-1.93	0.001
1423213_at	plexin C1	Plxnc1	-1.93	0.002
1437164_x_at	ATP synthase, H+ transporting, mitochondrial F1 complex, O subunit	Atp5o /// LOC432676	-1.93	0.000
1424453_at	phosphate cytidylyltransferase 1, choline, alpha isoform	Pcyt1a	-1.93	0.001
1425261_at	CCAAT/enhancer binding protein (C/EBP), gamma	Cebpg	-1.93	0.007
1443779_s_at	Mblk1-related protein-2	MGI:2443930	-1.93	0.000
1440383_at	DNA cross-link repair 1B, PSO2 homolog (S. cerevisiae)	Dclre1b	-1.93	0.005
1433983_at	Cnksr family member 3	Cnksr3	-1.93	0.000
1428372_at	suppression of tumorigenicity 5	St5	-1.93	0.004
1443214_at	G protein-coupled receptor 155	Gpr155	-1.94	0.009
1448171_at	seven in absentia 2	Siah2	-1.94	0.003

1434360_s_at	protein tyrosine phosphatase, receptor type, G	Ptprg	-1.94	0.003
1448420_a_at	F-box and leucine-rich repeat protein 12	Fbxl12	-1.94	0.010
1422525_at	ATP synthase, H+ transporting, mitochondrial F1F0 complex, subunit e	Atp5k	-1.94	0.000
1431561_a_at	DEAH (Asp-Glu-Ala-His) box polypeptide 34	Dhx34	-1.94	0.003
1434192_at	zinc finger, ZZ-type with EF hand domain 1 /// similar to KIAA0399 protein	Zzef1 /// LOC380708	-1.94	0.001
1460705_at	ribosomal protein S6 kinase, polypeptide 1	Rps6kb1	-1.94	0.007
1422147_a_at	phospholipase A2, group VI	Pla2g6	-1.94	0.003
1445517_at	Junction-mediating and regulatory protein	Jmy	-1.94	0.000
1418472_at	aspartoacylase (aminoacylase) 2	Aspa	-1.94	0.006
1450044_at	frizzled homolog 7 (Drosophila)	Fzd7	-1.95	0.001
1448014_s_at	ubiquitin specific protease 24	Usp24	-1.95	0.000
1419301_at	frizzled homolog 4 (Drosophila)	Fzd4	-1.95	0.009
1416159_at	nuclear receptor subfamily 2, group F, member 2	Nr2f2	-1.95	0.002
1460006_at	AT motif binding factor 1	Atbf1	-1.95	0.001
1428401_at	zinc finger, CCHC domain containing 3	Zcchc3	-1.95	0.008
1436584_at	sprouty homolog 2 (Drosophila)	Spry2	-1.95	0.008
1424198_at	discs, large homolog 5 (Drosophila)	Dlg5	-1.95	0.003
1437865_at	spermatogenesis associated 13	Spata13	-1.95	0.002
1442102_at	Aconitase 2, mitochondrial	Aco2	-1.96	0.001
1428744_s_at	Bri3 binding protein	Bri3bp	-1.96	0.000
1427358_a_at	death associated protein kinase 1	Dapk1	-1.96	0.009
1448462_at	thymine DNA glycosylase	Tdg	-1.96	0.008
1417908_s_at	ubiquitin-conjugating enzyme E2L 3	Ube2l3	-1.96	0.006
1440489_at	BTB (POZ) domain containing 4	Btbd4	-1.96	0.010
1434258_s_at	phosphatase and actin regulator 4	Phactr4	-1.96	0.008
1419527_at	cartilage oligomeric matrix protein	Comp	-1.96	0.010
1430555_s_at	leucine-rich repeats and immunoglobulin-like domains 3	Lrig3	-1.96	0.003
1442285_at	synaptic nuclear envelope 2	Syne2	-1.96	0.001
1437941_at	phosphorylase kinase alpha 2	Phka2	-1.96	0.005
1426700_a_at	ubiquitin specific protease 52	Usp52	-1.96	0.001
1428757_at	aminoadipate-semialdehyde dehydrogenase-phosphopantetheinyl transferase	Aasdhppt	-1.97	0.005
1426265_x_at	dihydrolipoamide S-acetyltransferase	Dlat	-1.97	0.004
1426760_at	importin 8	Ipo8	-1.97	0.001
1433679_at	far upstream element (FUSE) binding protein 3	Fubp3	-1.97	0.001
1424489_a_at	tRNA isopentenyltransferase 1	Trit1	-1.97	0.004
1437474_at	GATA zinc finger domain containing 2B	Gatad2b	-1.97	0.002
1452864_at	mediator of RNA polymerase II transcription, subunit 12 homolog (yeast)-like	Med12l	-1.97	0.007
1435664_at	zinc finger protein 397	Zfp397	-1.97	0.002
1449706_s_at	nuclear receptor subfamily 5, group A, member 2	Nr5a2	-1.97	0.003
1428791_at	ubiquitin-conjugating enzyme E2H	Ube2h	-1.97	0.003
1427233_at	serologically defined colon cancer antigen 33	Sdccag33	-1.98	0.005
1425209_at	zinc finger protein 84	Zfp84	-1.98	0.005
1457608_at	GTPase, IMAP family member 8	Gimap8	-1.98	0.004
1456090_at	pyruvate dehydrogenase complex, component X	Pdhx	-1.98	0.009
1425330_a_at	protein phosphatase 1B, magnesium dependent, beta isoform	Ppm1b	-1.98	0.002
1426831_at	S-adenosylhomocysteine hydrolase-like 1	Ahcyl1	-1.98	0.000
1460189_at	WD repeat domain 23	Wdr23	-1.98	0.007
1434717_at	Cullin 3	Cul3	-1.98	0.008
1453794_at	fer-1-like 4 (C. elegans)	Fer1l4	-1.98	0.007
1426590_at	G elongation factor, mitochondrial 2	Gfm2	-1.98	0.003
1422786_at	solute carrier family 30 (zinc transporter), member 1	Slc30a1	-1.98	0.002
1452210_at	DNA2 DNA replication helicase 2-like (yeast)	Dna2l	-1.98	0.006
1422829_at	dopamine receptor 4	Drd4	-1.98	0.002
1452297_at	cyclin T2	Ccnt2	-1.99	0.010

1460682_s_at	CEA-related cell adhesion molecule 2	Ceacam2	-1.99	0.003
1454663_at	eukaryotic translation initiation factor 5	Eif5	-1.99	0.000
1448746_at	nibrin	Nbn	-1.99	0.000
1455179_at	membrane protein, palmitoylated 7 (MAGUK p55 subfamily member 7)	Mpp7	-1.99	0.001
1417801_a_at	protein tyrosine phosphatase, receptor-type, F interacting protein, binding protein 2	Ppfibp2	-1.99	0.005
1454936_at	valyl-tRNA synthetase 2-like	Vars2l	-1.99	0.004
1426172_a_at	CD209a antigen	Cd209a	-1.99	0.001
1421893_a_at	tripeptidyl peptidase II	Tpp2	-1.99	0.008
1448104_at	aldehyde dehydrogenase family 6, subfamily A1	Aldh6a1	-1.99	0.005
1450783_at	interferon-induced protein with tetratricopeptide repeats 1	Ifit1	-1.99	0.007
1434330_at	leucine rich repeat containing 35	Lrrc35	-1.99	0.005
1440641_at	Vacuolar protein sorting 29 (S. pombe)	Vps29	-2.00	0.003
1452213_at	testis expressed gene 2	Tex2	-2.00	0.002
1455324_at	hypothetical LOC433022	LOC433022	-2.00	0.006
1425605_a_at	limb region 1	Lmbr1	-2.00	0.001
1439841_at	zinc finger, FYVE domain containing 27	Zfyve27	-2.00	0.001
1430397_at	glutamate oxaloacetate transaminase 2, mitochondrial	Got2	-2.00	0.003
1422503_s_at	poly (ADP-ribose) polymerase family, member 1	Parp1	-2.00	0.005
1417566_at	abhydrolase domain containing 5	Abhd5	-2.00	0.003
1427460_at	TAF4A RNA polymerase II, TATA box binding protein (TBP)-associated factor	Taf4a	-2.00	0.002
1453111_a_at	DNA segment, Chr 11, ERATO Doi 333, expressed	D11Ertd333e	-2.00	0.003
1418788_at	endothelial-specific receptor tyrosine kinase	Tek	-2.01	0.000
1435877_at	serine/threonine kinase 38 like	Stk38l	-2.01	0.004
1448227_at	growth factor receptor bound protein 7	Grb7	-2.01	0.004
1418840_at	programmed cell death 4	Pdcd4	-2.01	0.003
1423654_a_at	ring finger protein 4	Rnf4	-2.01	0.001
1451956_a_at	opioid receptor, sigma 1	Oprs1	-2.01	0.009
1422033_a_at	ciliary neurotrophic factor	Cntf	-2.01	0.010
1434106_at	EPM2A (laforin) interacting protein 1	Epm2aip1	-2.01	0.002
1451552_at	MRNA similar to lipoyltransferase	---	-2.01	0.006
1422960_at	steroid 5 alpha-reductase 2	Srd5a2	-2.02	0.004
1450665_at	GA repeat binding protein, alpha	Gabpa	-2.02	0.001
1454684_at	Bardet-Biedl syndrome 7	Bbs7	-2.02	0.006
1415936_at	breast cancer anti-estrogen resistance 3	Bcar3	-2.02	0.006
1452079_s_at	testis derived transcript 3, pseudogene /// testis derived transcript 3	Tes3-ps /// Tes3	-2.02	0.002
1419460_at	ribonuclease P 14 subunit (human)	Rpp14	-2.02	0.010
1433713_at	GCN1 general control of amino-acid synthesis 1-like 1 (yeast)	Gcn1l1	-2.02	0.007
1460498_a_at	DnaJ (Hsp40) homolog, subfamily C, member 5	Dnajc5	-2.02	0.006
1449036_at	ring finger protein 128	Rnf128	-2.02	0.001
1440679_at	DNA segment, Chr 6, Brigham & Women's Genetics 1452 expressed	D6Bwg1452e	-2.02	0.004
1436753_at	aarF domain containing kinase 5	Adck5	-2.03	0.001
1431385_a_at	membrane-bound transcription factor protease, site 1	Mbtps1	-2.03	0.003
1446007_at	Bone morphogenetic protein 1	Bmp1	-2.03	0.010
1452292_at	Adaptor-related protein complex 2, beta 1 subunit	Ap2b1	-2.03	0.009
1447757_x_at	inositol polyphosphate-5-phosphatase F	Inpp5f	-2.03	0.003
1451219_at	ORM1-like 1 (S. cerevisiae)	Ormdl1	-2.03	0.002
1427500_at	signal peptidase complex subunit 1 homolog (S. cerevisiae)	Spcs1	-2.03	0.005
1440329_s_at	G patch domain containing 2	Gpatc2	-2.03	0.001
1450010_at	hydroxysteroid (17-beta) dehydrogenase 12	Hsd17b12	-2.04	0.001
1447882_x_at	DEAD (Asp-Glu-Ala-Asp) box polypeptide 54	Ddx54	-2.04	0.002
1427971_at	hyperparathyroidism 2 homolog (human)	Hrpt2	-2.04	0.002
1452050_at	calcium/calmodulin-dependent protein kinase ID	Camk1d	-2.04	0.004

1434180_at	pleckstrin homology domain containing, family C (with FERM domain) member 1	Plekhc1	-2.04	0.001
1445280_at	Adaptor-related protein complex 3, delta 1 subunit	Ap3d1	-2.04	0.004
1428292_at	NADPH dependent diflavin oxidoreductase 1	Ndor1	-2.04	0.004
1435786_at	kelch-like 12 (Drosophila)	Klhl12	-2.04	0.006
1455915_at	UDP-N-acetyl-alpha-D-galactosamine	Galnt4	-2.04	0.006
1435889_at	MAP/microtubule affinity-regulating kinase 2	Mark2	-2.04	0.004
1419385_a_at	ubiquilin 1	Ubqln1	-2.04	0.000
1423493_a_at	nuclear factor I/X	Nfix	-2.04	0.003
1436181_at	development and differentiation enhancing factor 2	Ddef2	-2.04	0.001
1426744_at	sterol regulatory element binding factor 2	Srebf2	-2.05	0.001
1439775_at	bromodomain and WD repeat domain containing 3	Brwd3	-2.05	0.000
1441324_at	Zinc finger protein 395	Zfp395	-2.05	0.007
1452896_at	gene trap locus 3	Gtl3	-2.05	0.004
1459842_x_at	nucleotide binding protein 2	Nubp2	-2.05	0.008
1456355_s_at	sensitivity to red light reduced homolog (Arabidopsis)	MGI:1917368	-2.05	0.004
1454303_at	epsin 2	Epn2	-2.05	0.009
1420037_at	ATP synthase, H+ transporting, mitochondrial F1 complex, alpha subunit, isoform 1	Atp5a1	-2.05	0.000
1423297_at	adducin 3 (gamma)	Add3	-2.05	0.006
1451687_a_at	transcription factor 2	Tcf2	-2.05	0.001
1451996_at	beta-amyloid binding protein precursor	Bbp	-2.05	0.007
1456576_x_at	CCR4-NOT transcription complex, subunit 2	Cnot2	-2.05	0.007
1435291_at	integral inner nuclear membrane protein MAN1	Man1	-2.06	0.008
1460740_at	clathrin, light polypeptide (Lcb)	Cltb	-2.06	0.009
1452735_at	PEST-containing nuclear protein	Pcnp	-2.06	0.001
1449569_at	thrombopoietin	Thpo	-2.06	0.006
1428003_s_at	thiamine triphosphatase	Thtpa	-2.06	0.007
1417663_a_at	N-myc downstream regulated gene 3	Ndrg3	-2.06	0.002
1434066_at	general transcription factor III C 1	Gtf3c1	-2.06	0.001
1450406_a_at	ST3 beta-galactoside alpha-2,3-sialyltransferase 3	St3gal3	-2.06	0.002
1418285_at	ephrin B1	Efnb1	-2.06	0.004
1435604_at	tripartite motif protein 37	Trim37	-2.06	0.004
1460582_x_at	Nonagouti	a	-2.06	0.008
1458056_at	splicing factor, arginine/serine-rich 12	Sfrs12	-2.06	0.007
1417784_at	amyotrophic lateral sclerosis 2 (juvenile) homolog (human)	Als2	-2.06	0.003
1422293_a_at	potassium channel tetramerisation domain containing 1	Kctd1	-2.07	0.001
1416772_at	carnitine palmitoyltransferase 2	Cpt2	-2.07	0.009
1418585_at	cyclin H	Ccnh	-2.07	0.003
1447608_x_at	BTB (POZ) domain containing 14A	Btbd14a	-2.07	0.007
1448244_at	lysophospholipase 1	Lypla1	-2.07	0.000
1449151_at	PCTAIRE-motif protein kinase 3	Pctk3	-2.07	0.009
1421097_at	endonuclease G	Endog	-2.07	0.006
1457285_at	zinc finger protein 187	Zfp187	-2.07	0.003
1416873_a_at	cyclin-dependent kinase 2	Cdk2	-2.07	0.008
1420524_a_at	mannan-binding lectin serine protease 2	Masp2	-2.08	0.007
1448915_at	zinc finger protein 524	Zfp524	-2.08	0.005
1415891_at	succinate-CoA ligase, GDP-forming, alpha subunit	Suclg1	-2.08	0.002
1430802_at	histocompatibility 2, Q region locus 8	H2-Q8	-2.08	0.000
1442656_at	DNA segment, Chr 3, ERATO Doi 343, expressed	D3Ertd343e	-2.08	0.007
1444139_at	DNA-damage-inducible transcript 4-like	Ddit4l	-2.08	0.001
1436256_at	GrpE-like 2, mitochondrial	Grpel2	-2.08	0.006
1446433_at	Acyl-Coenzyme A binding domain containing 5	Acbd5	-2.09	0.009
1417235_at	EH-domain containing 3	Ehd3	-2.09	0.002
1434600_at	tight junction protein 2	Tjp2	-2.09	0.000
1452017_at	SRY-box containing gene 15	Sox15	-2.09	0.004

1427544_a_at	poly (A) polymerase alpha	Papola	-2.09	0.008
1426556_at	suppressor of hairy wing homolog 4 (Drosophila)	Suhw4	-2.09	0.008
1439131_at	CXORF15	4932441K18	-2.09	0.002
1427472_a_at	complement component 8, beta subunit	C8b	-2.09	0.008
1454907_at	Small EDRK-rich factor 1	Serf1	-2.09	0.001
1453571_at	DEP domain containing 6	Depdc6	-2.09	0.001
1455142_at	suppressor of cytokine signaling 4	Socs4	-2.10	0.004
1427950_at	zinc finger protein 294	Zfp294	-2.10	0.006
1419031_at	fatty acid desaturase 2	Fads2	-2.10	0.005
1450097_s_at	guanine nucleotide binding protein, alpha 12	Gna12	-2.10	0.004
1422877_at	protocadherin beta 12	Pcdhb12	-2.10	0.007
1426994_at	PH domain and leucine rich repeat protein phosphatase	Phlpp	-2.10	0.003
1448499_a_at	epoxide hydrolase 2, cytoplasmic	Ephx2	-2.10	0.008
1455047_at	F-box only protein 3	Fbxo3	-2.10	0.000
1456143_at	protein kinase C binding protein 1	Prkcbp1	-2.10	0.001
1427940_s_at	c-myc binding protein	Mycbp	-2.11	0.005
1442051_at	histone 2, H3c1	Hist2h3c1	-2.11	0.002
1418399_at	potassium channel tetramerisation domain containing 9	Kctd9	-2.11	0.006
1434296_at	similar to hypothetical protein FLJ38281	LOC234413	-2.11	0.000
1420950_at	zinc and ring finger 1	Znrf1	-2.11	0.006
1426715_s_at	DNA segment, Chr 11, ERATO Doi 18, expressed	D11Ertd18e	-2.12	0.003
1451139_at	solute carrier family 39 (zinc transporter), member 4	Slc39a4	-2.12	0.002
1422061_at	aldo-keto reductase family 1, member C20	Akr1c20	-2.12	0.007
1418672_at	aldo-keto reductase family 1, member C13	Akr1c13	-2.12	0.006
1419552_at	enoyl Coenzyme A hydratase domain containing 1	Echdc1	-2.12	0.004
1416481_s_at	hypoxia induced gene 1	MGI:1930666	-2.12	0.005
1440965_at	Phosphatidylinositol glycan, class L	Pigl	-2.12	0.002
1457696_at	Rab interacting lysosomal protein	Rilp	-2.12	0.005
AFFX-r2-Ec-bioB-M_at	biotin synthesis, sulfur insertion?	bioB	-2.13	0.005
1429108_at	ring finger protein 184	Rnf184	-2.13	0.000
1437296_at	protein kinase N2	Pkn2	-2.13	0.001
1456131_x_at	dystroglycan 1	Dag1	-2.13	0.003
1448237_x_at	lactate dehydrogenase 2, B chain	Ldh2	-2.13	0.004
1437172_x_at	hydr/cyl-CoA dehydrogenase/3-ketoacyl-CoA thiolase/enoyl-CoA hydr/se b subunit	Hadhb	-2.13	0.000
1459168_at	Zinc finger protein 521	Zfp521	-2.13	0.007
1460225_at	mitochondrial ribosomal protein 63	Mrp63	-2.14	0.007
1416383_a_at	pyruvate carboxylase	Pcx	-2.14	0.004
1451711_at	wingless-type MMTV integration site 9B	Wnt9b	-2.14	0.007
1424579_at	solute carrier family 35 member 3	Slc35a3	-2.14	0.001
1447655_x_at	SRY-box containing gene 6	Sox6	-2.14	0.007
1437181_at	pellino 2	Peli2	-2.14	0.002
1452391_at	coxsackievirus and adenovirus receptor	Cxadr	-2.14	0.002
1417980_a_at	insulin induced gene 2	Insig2	-2.15	0.009
1457069_at	activating signal cointegrator 1 complex subunit 3	Ascc3	-2.15	0.008
1447720_x_at	Protein kinase, cAMP dependent, catalytic, alpha	Prkaca	-2.15	0.001
1434182_at	DEAD/H (Asp-Glu-Ala-Asp/His) box polypeptide 31	Ddx31	-2.15	0.010
1443547_at	Solute carrier family 10 (sodium/bile acid cotransporter family), member 1	Slc10a1	-2.16	0.003
1449418_s_at	F-box only protein 36	Fbxo36	-2.16	0.003
1426857_a_at	hydroxysteroid dehydrogenase like 2	Hsdl2	-2.16	0.005
1438447_at	DEAD/H (Asp-Glu-Ala-Asp/His) box polypeptide 11	Ddx11	-2.16	0.006
1428514_at	copine III	Cpne3	-2.16	0.004
1457539_at	DNA segment, Chr 10, ERATO Doi 709, expressed	D10Ertd709e	-2.16	0.009

1426670_at	agrin	Agrn	-2.16	0.003
1438030_at	RAS, guanyl releasing protein 3	Rasgrp3	-2.16	0.002
1422001_at	inhibin beta-C	Inhbc	-2.17	0.004
1419179_at	thioredoxin-like 4	Txnl4	-2.17	0.001
1448960_at	CXXC finger 5	Cxxc5	-2.17	0.001
1416049_at	glycine decarboxylase	Gldc	-2.17	0.005
1451538_at	SRY-box containing gene 9	Sox9	-2.18	0.002
1451833_a_at	SET domain, bifurcated 1	Setdb1	-2.18	0.002
1419245_at	RAB14, member RAS oncogene family	Rab14	-2.18	0.001
1430526_a_at	SWI/SNF related, subfamily a, member 2	Smarca2	-2.18	0.008
1420014_at	Lanosterol synthase	Lss	-2.18	0.002
1456630_x_at	Son cell proliferation protein	Son	-2.18	0.002
1426160_a_at	serine/threonine kinase 16	Stk16	-2.18	0.002
1434077_at	WD repeat domain 37	Wdr37	-2.18	0.000
1424470_a_at	Rap guanine nucleotide exchange factor (GEF) 3	Rapgef3	-2.18	0.010
1453187_at	OCIA domain containing 2	Ociad2	-2.18	0.008
1435234_at	nuclear receptor coactivator 2	Ncoa2	-2.18	0.002
1438370_x_at	downstream of Stk11	Dos	-2.18	0.003
1453377_at	SH2 domain containing 4A	Sh2d4a	-2.18	0.009
1429653_at	genetic suppressor element 1	Gse1	-2.18	0.000
1449010_at	heat shock 70kDa protein 4 like	Hspa4l	-2.19	0.001
1423868_at	thioredoxin reductase 3	Txnrd3	-2.19	0.002
1450948_a_at	mitochondrial ribosomal protein L1	Mrpl1	-2.19	0.003
1449403_at	phosphodiesterase 9A	Pde9a	-2.19	0.007
1424959_at	annexin A13	Anxa13	-2.19	0.001
1456728_x_at	aconitase 1	Aco1	-2.20	0.009
1417555_at	ATPase family, AAA domain containing 1	Atad1	-2.20	0.006
1428782_a_at	ubiquinol-cytochrome c reductase core protein 1	Uqcrc1	-2.20	0.002
1423229_at	inositol polyphosphate-5-phosphatase E	Inpp5e	-2.20	0.002
1449817_at	ATP-binding cassette, sub-family B (MDR/TAP), member 11	Abcb11	-2.20	0.004
1426049_a_at	telomeric repeat binding factor 2, interacting protein	Terf2ip	-2.20	0.003
1418352_at	hydroxysteroid (17-beta) dehydrogenase 2	Hsd17b2	-2.20	0.009
1420585_a_at	nuclear RNA export factor 2	Nxf2	-2.21	0.007
1458878_at	Yamaguchi sarcoma viral (v-yes) oncogene homolog 1	Yes1	-2.21	0.006
1435155_at	cingulin	Cgn	-2.21	0.000
1449407_at	carnitine deficiency-associated gene expressed in ventricle 1	Cdv1	-2.21	0.002
1431947_at	low density lipoprotein receptor	Ldlr	-2.22	0.007
1417098_s_at	nuclear receptor binding factor 1	Nrbf1	-2.22	0.002
1429692_s_at	GTP cyclohydrolase 1	Gch1	-2.22	0.008
1434650_at	pogo transposable element with ZNF domain	Pogz	-2.22	0.005
1430125_s_at	PQ loop repeat containing 1	Pqlc1	-2.22	0.005
1451743_at	DNA segment, Chr 19, Wayne State University 162, expressed	D19Wsu162e	-2.22	0.002
1437982_x_at	COX15 homolog, cytochrome c oxidase assembly protein (yeast)	Cox15	-2.22	0.003
1425932_a_at	CUG triplet repeat, RNA binding protein 1	Cugbp1	-2.22	0.003
1438812_x_at	ubiquitin specific protease 19	Usp19	-2.22	0.000
1449585_at	interleukin 1 receptor accessory protein	Il1rap	-2.23	0.006
1440822_x_at	RalBP1 associated Eps domain containing protein	Reps1	-2.23	0.004
1425050_at	isochorismatase domain containing 1	Isoc1	-2.23	0.002
1429188_at	COX11 homolog, cytochrome c oxidase assembly protein (yeast)	Cox11	-2.23	0.006
1460420_a_at	epidermal growth factor receptor	Egfr	-2.23	0.008
1443133_at	neurofilament 3, medium	Nef3	-2.24	0.009
1441017_at	zinc finger, CCHC domain containing 14	Zcchc14	-2.24	0.007
1437867_at	similar to protease	LOC385308	-2.24	0.001
1457793_a_at	Wolf-Hirschhorn syndrome candidate 1-like 1 (human)	Whsc1l1	-2.24	0.002
1447780_x_at	Tu translation elongation factor, mitochondrial	Tufm	-2.24	0.002

1425249_a_at	TYRO3 protein tyrosine kinase 3	Tyro3	-2.24	0.002
1423508_at	MYST histone acetyltransferase monocytic leukemia 4	Myst4	-2.25	0.000
1420617_at	cytoplasmic polyadenylation element binding protein 4	Cpeb4	-2.25	0.001
1424475_at	calcium/calmodulin-dependent protein kinase kinase 2, beta	Camkk2	-2.25	0.008
1425799_at	flavin containing monooxygenase 4	Fmo4	-2.25	0.002
1420411_a_at	phosphatidylinositol 4-kinase type 2 beta	Pi4k2b	-2.25	0.007
1425071_s_at	neurotrophic tyrosine kinase, receptor, type 3	Ntrk3	-2.25	0.002
1428151_x_at	cysteine conjugate-beta lyase 1	Ccbl1	-2.25	0.005
1451459_at	embryonic large molecule derived from yolk sac	MGI:1915033	-2.25	0.001
1429418_at	CDC14 cell division cycle 14 homolog B (S. cerevisiae)	Cdc14b	-2.25	0.004
1443908_at	Gene model 1609, (NCBI)	---	-2.25	0.008
1416199_at	kinesin family member C3	Kifc3	-2.25	0.002
1426164_a_at	upstream transcription factor 1	Usf1	-2.25	0.007
1423831_at	protein kinase, AMP-activated, gamma 2 non-catalytic subunit	Prkag2	-2.25	0.010
1449302_at	ATP-binding cassette, sub-family A (ABC1), member 2	Abca2	-2.26	0.009
1421057_at	deoxyribonuclease 1-like 3	Dnase1l3	-2.26	0.010
1438843_x_at	mitochondrial carrier homolog 2 (C. elegans)	Mtch2	-2.26	0.004
1424760_a_at	SET and MYND domain containing 2	Smyd2	-2.26	0.001
1424456_at	poliovirus receptor-related 2	Pvrl2	-2.26	0.002
1420663_at	T helper-inducing POZ/Krueppel factor	Thpok	-2.27	0.010
1448819_at	eukaryotic translation initiation factor 2, subunit 2 (beta)	Eif2s2	-2.27	0.008
1439860_at	eukaryotic elongation factor-2 kinase	Eef2k	-2.27	0.009
1449481_at	solute carrier family 25 member 13	Slc25a13	-2.27	0.008
1428423_at	ring finger protein 3	Rnf3	-2.27	0.001
1422558_at	guanidinoacetate methyltransferase	Gamt	-2.28	0.004
1422826_at	insulin-like growth factor binding protein, acid labile subunit	Igfals	-2.28	0.009
1418604_at	arginine vasopressin receptor 1A	Avpr1a	-2.28	0.007
1451636_at	ankyrin repeat domain 33	Ankrd33	-2.28	0.006
1433505_a_at	leucine rich repeat containing 5	Lrrc5	-2.28	0.005
1419671_a_at	interleukin 17 receptor C	Il17rc	-2.28	0.004
1434289_at	NOL1/NOP2/Sun domain family, member 5	Nsun5	-2.28	0.007
1459242_at	ELOVL family member 5, elongation of long chain fatty acids (yeast)	Elovl5	-2.28	0.009
1449341_a_at	stomatin	Stom	-2.28	0.007
1451950_a_at	CD80 antigen	Cd80	-2.29	0.004
1418510_s_at	F-box only protein 8	Fbxo8	-2.29	0.001
1434391_at	Heterogeneous nuclear ribonucleoprotein U	Hnrpu	-2.29	0.005
1454014_a_at	McKusick-Kaufman syndrome protein	Mkks	-2.29	0.006
1450384_at	beta-site APP cleaving enzyme 1	Bace1	-2.29	0.008
1435046_at	Myosin ID	Myo1d	-2.30	0.001
1436244_a_at	transducin-like enhancer of split 2, homolog of Drosophila E(spl)	Tle2	-2.30	0.004
1418547_at	tissue factor pathway inhibitor 2	Tfpi2	-2.30	0.004
1449938_at	placental protein 11 related	Pp11r	-2.30	0.003
1416275_at	solute carrier family 26, member 6	Slc26a6	-2.31	0.004
1435775_at	TPA regulated locus	Tparl	-2.31	0.001
1428931_a_at	poly (ADP-ribose) polymerase family, member 6	Parp6	-2.31	0.001
1435097_at	methylmalonic aciduria (cobalamin deficiency) type B homolog (human)	Mmab	-2.32	0.010
1438065_at	Lurcher transcript 1	BC021395	-2.32	0.001
1419400_at	microsomal triglyceride transfer protein	Mttp	-2.32	0.002
1417580_s_at	selenium binding protein 1	Selenbp1	-2.32	0.000
1427369_at	NACHT, leucine rich repeat and PYD containing 6	Nalp6	-2.32	0.009
1416579_a_at	tumor-associated calcium signal transducer 1	Tacstd1	-2.32	0.005
1451788_at	coagulation factor XI	F11	-2.32	0.007
1427005_at	polo-like kinase 2 (Drosophila)	Plk2	-2.32	0.008
1435296_at	adrenergic receptor, alpha 2c	Adra2c	-2.32	0.002

1449202_at	sema domain, immunoglobulin domain (Ig), transmembrane domain (TM) and short cytoplasmic domain, (semaphorin) 4G	Sema4g	-2.33	0.008
1427665_a_at	nuclear factor I/C	Nfic	-2.33	0.001
1456485_at	nuclear protein in the AT region	Npat	-2.33	0.006
1460325_at	pumilio 1 (Drosophila)	Pum1	-2.33	0.000
1421884_at	Son of sevenless homolog 1 (Drosophila)	Sos1	-2.33	0.001
1455100_at	aldo-keto reductase family 1, member D1	Akr1d1	-2.33	0.008
1424886_at	protein tyrosine phosphatase, receptor type, D	Ptprd	-2.33	0.002
1418066_at	cofilin 2, muscle	Cfl2	-2.33	0.004
1449443_at	2,4-dienoyl CoA reductase 1, mitochondrial	Decr1	-2.33	0.005
1426233_at	mitogen activated protein kinase kinase 4	Map2k4	-2.34	0.009
1450204_a_at	myoneurin	Mynn	-2.34	0.002
1460010_a_at	phosphatidylserine synthase 2	Ptdss2	-2.34	0.010
1440979_at	Insulin-like growth factor 2 receptor	Igf2r	-2.34	0.004
1451419_at	SPRY domain-containing SOCS box 4	MGI:2183445	-2.34	0.001
1428509_at	myosin IE	Myo1e	-2.35	0.002
1422990_at	met proto-oncogene	Met	-2.35	0.004
1419009_at	actin-like 7a	Actl7a	-2.35	0.007
1426595_at	solute carrier family 18 (vesicular monoamine), member 1	Slc18a1	-2.36	0.002
1451828_a_at	acyl-CoA synthetase long-chain family member 4	Acsl4	-2.36	0.003
1447854_s_at	histone 2, H2bb	Hist2h2bb	-2.36	0.001
1425115_at	retinoblastoma binding protein 6	Rbbp6	-2.36	0.000
1443029_at	PTPRF interacting protein, binding protein 1 (liprin beta 1)	Ppfibp1	-2.36	0.000
1455057_at	Guanine monphosphate synthetase	Gmps	-2.36	0.001
1459860_x_at	tripartite motif protein 2	Trim2	-2.37	0.001
1417553_at	placental specific protein 1	Plac1	-2.37	0.009
1442538_at	REST corepressor 3	Rcor3	-2.37	0.005
1435861_at	protein tyrosine phosphatase, receptor type, f polypeptide (PTPRF), interacting protein, alpha 1	Ppfia1	-2.37	0.003
1451871_a_at	Growth hormone receptor	Ghr	-2.37	0.002
1425276_at	fibrosin 1	Fbs1	-2.37	0.000
1442697_at	Importin 11	Ipo11	-2.37	0.009
1434838_at	similar to Potassium voltage-gated channel subfamily G member 2 (Voltage-gated potassium channel subunit Kv6.2) (Cardiac potassium channel subunit)	LOC240444	-2.37	0.003
1436318_at	TAR DNA binding protein	Tardbp	-2.38	0.001
1415888_at	hepatoma-derived growth factor	Hdgf	-2.38	0.010
1422461_at	ATPase family, AAA domain containing 3A	Atad3a	-2.38	0.010
1436697_at	Down syndrome critical region gene 3	Dscr3	-2.39	0.004
1415712_at	zinc finger, RAN-binding domain containing 1	Zranb1	-2.39	0.004
1444421_at	Zinc finger, CCHC domain containing 6	Zcchc6	-2.39	0.010
1448873_at	occludin	Ocln	-2.39	0.008
1429656_at	Rho-related BTB domain containing 1	Rhobtb1	-2.39	0.003
1419492_s_at	defensin beta 1	Defb1	-2.40	0.006
1429550_at	ectonucleoside triphosphate diphosphohydrolase 8	Entpd8	-2.40	0.009
1422833_at	forkhead box A2	Foxa2	-2.40	0.004
1419401_at	ankyrin repeat and SOCS box-containing protein 13	Asb13	-2.41	0.007
1427042_at	mal, T-cell differentiation protein 2	Mal2	-2.41	0.004
1436189_at	Receptor (TNFRSF)-interacting serine-threonine kinase 1	Serpinb6a	-2.41	0.001
1419034_at	casein kinase II, alpha 1 polypeptide	Csnk2a1	-2.41	0.008
1460684_at	transmembrane 7 superfamily member 2	Tm7sf2	-2.41	0.004
1449888_at	endothelial PAS domain protein 1	Epas1	-2.42	0.003
1448800_at	reticulon 4 interacting protein 1	Rtn4ip1	-2.42	0.001
1450869_at	fibroblast growth factor 1	Fgf1	-2.42	0.003
1452269_at	spectrin beta 3	Spnb3	-2.42	0.009
1450364_a_at	hepatitis A virus cellular receptor 1	Havcr1	-2.42	0.006

1427862_at	ADP-ribosylation factor-like 6 interacting protein 3	Arl6ip3	-2.42	0.002
1457832_at	7 days neonate cerebellum cDNA, RIKEN full-length enriched library, clone:A730031J22 product:unknown EST, full insert sequence	---	-2.43	0.007
1440904_at	SUMO/sentrin specific protease 5	Senp5	-2.43	0.008
1417203_at	ethylmalonic encephalopathy 1	Ethe1	-2.43	0.003
1442927_at	PTK2 protein tyrosine kinase 2 beta	Ptk2b	-2.43	0.002
1444873_at	Huntingtin interacting protein 1	Hip1	-2.43	0.006
1443484_at	RNA polymerase II associated protein 1	Rpap1	-2.44	0.006
1444037_at	Lectin, mannose-binding, 1	Lman1	-2.44	0.004
1433781_a_at	claudin 12	Cldn12	-2.44	0.004
1427762_x_at	histone 1, H2bp	Hist1h2bp	-2.44	0.003
1420117_at	Golgi phosphoprotein 3	Golph3	-2.44	0.005
1425041_at	LIM homeobox protein 3	Lhx3	-2.44	0.002
1427143_at	jumonji, AT rich interactive domain 1B (Rbp2 like)	Jarid1b	-2.44	0.001
1431071_at	metaxin 3	Mtx3	-2.44	0.000
1436755_at	inter-alpha (globulin) inhibitor H5	Itih5	-2.45	0.007
1441755_at	mitogen-activated protein kinase 15	Mapk15	-2.45	0.007
1433904_at	gametogenetin binding protein 1	MGI:3055306	-2.45	0.009
1445548_at	Radixin	Rdx	-2.45	0.004
1448958_at	interferon alpha responsive gene	MGI:1929890	-2.45	0.004
1431630_a_at	Kruppel-like factor 3 (basic)	Klf3	-2.46	0.007
1425675_s_at	CEA-related cell adhesion molecule 1	Ceacam1	-2.46	0.001
1419103_a_at	abhydrolase domain containing 6	Abhd6	-2.46	0.009
1444691_at	protein kinase C, nu	Prkcn	-2.46	0.006
1456009_x_at	topoisomerase (DNA) III beta	Top3b	-2.47	0.008
1460232_s_at	hydroxysteroid dehydrogenase-2	Hsd3b2	-2.47	0.008
1433647_s_at	Rho-related BTB domain containing 3	Rhobtb3	-2.47	0.000
1438908_at	mitogen activated protein kinase kinase kinase 12	Map3k12	-2.47	0.002
1437175_at	PDLIM1 interacting kinase 1 like	Pdik1l	-2.48	0.000
1453535_at	olfactory receptor 78	Olfr78	-2.48	0.008
1421189_at	gamma-aminobutyric acid (GABA-A) receptor, subunit beta 3	Gabrb3	-2.48	0.009
1451675_a_at	aminolevulinic acid synthase 2, erythroid	Alas2	-2.48	0.005
1450715_at	cytochrome P450, family 1, subfamily a, polypeptide 2	Cyp1a2	-2.48	0.002
1454219_at	DnaJ (Hsp40) homolog, subfamily C, member 2	Dnajc2	-2.48	0.004
1455244_at	dishevelled associated activator of morphogenesis 1	Daam1	-2.49	0.001
1444295_at	neogenin	Neo1	-2.49	0.005
1437400_at	neural precursor cell expressed, developmentally down-regulated gene 4-like	Nedd4l	-2.49	0.008
1423554_at	gamma-glutamyl carboxylase	Ggcx	-2.49	0.004
1451189_at	zinc finger, SWIM domain containing 1	Zswim1	-2.50	0.003
1452582_at	galactose mutarotase	Galm	-2.50	0.002
1439249_at	WW domain containing adaptor with coiled-coil	Wac	-2.50	0.000
1456767_at	leucine rich repeat and fibronectin type III domain containing 3	Lrfn3	-2.50	0.002
1449676_at	RAB2, member RAS oncogene family	Rab2	-2.50	0.006
1418351_a_at	DNA methyltransferase 3B	Dnmt3b	-2.50	0.006
1434072_at	Smith-Magenis syndrome chromosome region, candidate 7 homolog (human)	Smcr7	-2.50	0.002
1427917_s_at	single-stranded DNA binding protein 3	Ssbp3	-2.50	0.004
1426117_a_at	solute carrier family 19 (thiamine transporter), member 2	Slc19a2	-2.51	0.005
1417070_at	cytochrome P450, family 4, subfamily v, polypeptide 3	Cyp4v3	-2.51	0.004
1437791_s_at	echinoderm microtubule associated protein like 5	Eml5	-2.52	0.005
1430031_at	inositol polyphosphate multikinase	Ipmk	-2.53	0.002
1419436_at	complement component factor h-like 1	Cfhl1	-2.53	0.002
1429240_at	StAR-related lipid transfer (START) domain containing 4	Stard4	-2.54	0.004
1422850_at	poly(A) binding protein, nuclear 1	Pabpn1	-2.54	0.001

1424833_at	inositol 1,4,5-triphosphate receptor 2	Itpr2	-2.54	0.002
1456505_at	Braf transforming gene	Braf	-2.55	0.009
1434766_at	Protein kinase, AMP-activated, alpha 2 catalytic subunit	Prkaa2	-2.55	0.002
1431089_at	cleavage and polyadenylation specific factor 2	Cpsf2	-2.55	0.007
1436633_at	gene model 1567, (NCBI)	Gm1567	-2.55	0.005
1448029_at	T-box 3	Tbx3	-2.55	0.007
1453988_a_at	insulin degrading enzyme	Ide	-2.55	0.009
1429299_at	dimethylarginine dimethylaminohydrolase 1	Ddah1	-2.55	0.010
1457534_at	Ubiquitin associated protein 2-like	Ubap2l	-2.56	0.004
1424166_at	mutS homolog 3 (E. coli)	Msh3	-2.56	0.004
1452307_at	Cdk5 and Abl enzyme substrate 2	Cables2	-2.56	0.006
1419703_at	procollagen, type V, alpha 3	Col5a3	-2.56	0.002
1425252_a_at	mitotic arrest deficient 1-like 1	Mad1l1	-2.56	0.002
1431785_at	ribonuclease T2	Rnaset2	-2.57	0.004
1434454_at	DNA segment, Chr 16, Wayne State University 65, expressed	D16Wsu65e	-2.57	0.002
1420420_at	hydroxyacid oxidase 1, liver	Hao1	-2.57	0.002
1422912_at	bone morphogenetic protein 4	Bmp4	-2.57	0.007
1427590_at	zinc finger protein 39	Zfp39	-2.57	0.004
1420362_a_at	Bcl2-interacting killer-like	Biklk	-2.57	0.005
1432436_a_at	adenylate kinase 3	Ak3	-2.58	0.002
1420096_at	Zinc finger proliferation 1	Zipro1	-2.58	0.000
1416147_at	heat shock protein 4	Hspa4	-2.58	0.008
1422534_at	cytochrome P450, family 51	Cyp51	-2.59	0.005
1417880_at	glucose-6-phosphatase, catalytic	G6pc	-2.59	0.002
1439196_at	hook homolog 3 (Drosophila)	Hook3	-2.59	0.002
1423797_at	acetoacetyl-CoA synthetase	Aacs	-2.60	0.008
1430785_at	orphan short chain dehydrogenase/reductase	MGI:1917311	-2.60	0.005
1425034_at	solute carrier family 17 (sodium phosphate), member 2	Slc17a2	-2.60	0.006
1449001_at	isovaleryl coenzyme A dehydrogenase	Ivd	-2.61	0.002
1437377_a_at	polymerase (RNA) mitochondrial (DNA directed)	Polrmt	-2.61	0.005
1433942_at	myosin VI	Myo6	-2.61	0.007
1419390_at	phosphodiesterase 10A	Pde10a	-2.61	0.007
1419866_s_at	spinocerebellar ataxia 2 homolog (human)	Sca2	-2.61	0.000
1437489_x_at	succinate dehydrogenase complex, subunit D, integral membrane protein	Sdhd	-2.61	0.007
1453100_at	casein kinase II, alpha 2, polypeptide	Csnk2a2	-2.62	0.000
1449746_s_at	GLI pathogenesis-related 1 (glioma)	Glipr1	-2.62	0.001
1435540_at	immunity-related GTPase family, Q1	Irgq1	-2.62	0.000
1458847_at	Serine/threonine kinase 25 (yeast)	Stk25	-2.63	0.004
1434611_at	ring finger protein 123	Rnf123	-2.63	0.005
1449379_at	kinase insert domain protein receptor	Kdr	-2.63	0.007
1426537_at	NMDA receptor-regulated gene 2	Narg2	-2.63	0.005
1423854_a_at	RAS-like, family 11, member B	Rasl11b	-2.64	0.001
1459120_at	erythrocyte protein band 4.1-like 5	Epb4.1l5	-2.64	0.001
1437462_x_at	Matrix metalloproteinase 15	Mmp15	-2.64	0.002
1423149_at	S-phase kinase-associated protein 1A	Skp1a	-2.64	0.001
1425521_at	polyadenylate binding protein-interacting protein 1	Paip1	-2.64	0.009
1445952_at	BAI1-associated protein 1	Baiap1	-2.64	0.004
1444460_at	ubiquitin-conjugating enzyme E2R 2	Ube2r2	-2.65	0.008
1421769_at	LIM homeobox transcription factor 1 beta	Lmx1b	-2.65	0.010
1450930_at	hippocalcin	Hpca	-2.65	0.001
1439013_x_at	acylphosphatase 1, erythrocyte (common) type	Acyp1	-2.66	0.005
1435837_at	crystallin, alpha B	Cryab	-2.66	0.005
1433891_at	G protein-coupled receptor 48	Gpr48	-2.67	0.002
1429863_at	ring finger protein 127	Rnf127	-2.67	0.000

1439459_x_at	ATP citrate lyase	Acly	-2.67	0.000
1420097_at	DNA segment, Chr 13, ERATO Doi 787, expressed	D13Ertd787e	-2.67	0.007
1435647_at	inhibitor of kappaB kinase gamma	Ikbkg	-2.68	0.004
1454184_a_at	inhibitor of kappaB kinase beta	Ikbkb	-2.68	0.005
1428690_at	trypsin domain containing 1	Tysnd1	-2.68	0.008
1448043_x_at	ring finger protein 2	Rnf2	-2.68	0.008
1427708_a_at	neurofibromatosis 2	Nf2	-2.69	0.000
1451959_a_at	vascular endothelial growth factor A	Vegfa	-2.69	0.002
1417824_at	glycine C-acetyltransferase (2-amino-3-ketobutyrate-coenzyme A ligase)	Gcat	-2.69	0.000
1425661_at	cytidine and dCMP deaminase domain containing 1	Cdadc1	-2.70	0.001
1424809_at	crumbs homolog 3 (Drosophila)	Crb3	-2.70	0.002
1417066_at	chaperone, ABC1 activity of bc1 complex like (S. pombe)	Cabc1	-2.70	0.002
1419970_at	solute carrier family 35, member A5	Slc35a5	-2.71	0.004
1440131_at	Poliovirus receptor-related 1	Pvrl1	-2.72	0.003
1418001_at	integral membrane protein 2B	Itm2b	-2.72	0.006
1425140_at	lactamase, beta 2	Lactb2	-2.72	0.002
1454137_s_at	hemochromatosis type 2 (juvenile) (human homolog)	Hfe2	-2.72	0.000
1427551_at	ubiquitin specific protease 29	Usp29	-2.72	0.007
1425724_at	protein tyrosine phosphatase, receptor type, N polypeptide 2	Ptprn2	-2.73	0.009
1452610_at	zinc finger protein 99	Zfp99	-2.73	0.001
1451903_at	Kynureninase (L-kynurenine hydrolase)	Kynu	-2.73	0.000
1434898_at	trinucleotide repeat containing 6a	Tnrc6a	-2.73	0.000
1448865_at	hydroxysteroid (17-beta) dehydrogenase 7	Hsd17b7	-2.73	0.009
1428885_at	synaptogyrin 2 /// arylformamidase	Syngr2 /// Afmid	-2.74	0.008
1430034_at	chaperonin subunit 4 (delta)	Cct4	-2.74	0.009
1425631_at	protein phosphatase 1, regulatory (inhibitor) subunit 3C	Ppp1r3c	-2.75	0.002
1421765_at	paired box gene 5	Pax5	-2.75	0.009
1448340_at	DNA segment, Chr 9, Wayne State University 20, expressed	D9Wsu20e	-2.75	0.002
1416444_at	elongation of very long chain fatty acids (FEN1/Elo2, SUR4/Elo3, yeast)-like 2	Elovl2	-2.75	0.003
1426639_a_at	transcription factor 7-like 2, T-cell specific, HMG-box	Tcf7l2	-2.76	0.002
1416222_at	NAD(P) dependent steroid dehydrogenase-like	Nsdhl	-2.76	0.001
1418496_at	forkhead box A1	Foxa1	-2.76	0.010
1418301_at	interferon regulatory factor 6	Irf6	-2.76	0.000
1421835_at	microtubule-associated protein 7	Mtap7	-2.76	0.002
1422954_at	zinc finger protein 60	Zfp60	-2.76	0.007
1417843_s_at	EPS8-like 2	Eps8l2	-2.76	0.009
1452021_a_at	hairy and enhancer of split 6 (Drosophila)	Hes6	-2.77	0.002
1419913_at	Serine/threonine kinase receptor associated protein	Strap	-2.77	0.000
1453565_at	NADH dehydrogenase (ubiquinone) 1, alpha/beta subcomplex, 1	Ndufab1	-2.77	0.005
1450954_at	YME1-like 1 (S. cerevisiae)	Yme1l1	-2.78	0.002
1417689_a_at	PDZK1 interacting protein 1	Pdzk1ip1	-2.78	0.002
1432499_a_at	ubiquitination factor E4B, UFD2 homolog (S. cerevisiae)	Ube4b	-2.78	0.000
1419761_a_at	GA repeat binding protein, beta 1	Gabpb1	-2.79	0.009
1456254_at	Inositol polyphosphate-4-phosphatase, type II	Inpp4b	-2.79	0.007
1449019_at	A kinase (PRKA) anchor protein 1	Akap1	-2.79	0.002
1434363_x_at	PAX interacting (with transcription-activation domain) protein 1	Paxip1	-2.79	0.009
1446624_at	Fukuyama type congenital muscular dystrophy homolog (human)	Fcmd	-2.80	0.007
1454867_at	similar to dJ353E16.2 (meningioma (disrupted in balanced translocation) 1)	LOC433938	-2.81	0.005
1444810_at	Hypothetical protein E130101M22	Acac	-2.82	0.004
1457688_at	zinc finger protein 398	Zfp398	-2.82	0.004
1423874_at	WD repeat domain 33	Wdr33	-2.82	0.001
1431328_at	protein phosphatase 1, catalytic subunit, beta isoform	Ppp1cb	-2.83	0.008
1421255_a_at	calcium binding protein 1	Cabp1	-2.83	0.005

1419322_at	FYVE, RhoGEF and PH domain containing 6	Fgd6	-2.83	0.001
1431946_a_at	amyloid beta (A4) precursor protein-binding, family A, member 1 binding protein	Apba2bp	-2.83	0.005
1422363_at	olfactory receptor 64	Olfr64	-2.83	0.004
1450177_at	nerve growth factor receptor (TNFR superfamily, member 16)	Ngfr	-2.83	0.006
1437250_at	whn-dependent transcript 2	MGI:2151839	-2.83	0.010
1444312_at	hypothetical protein 4930477M19	4930477M19	-2.84	0.007
1430826_s_at	glucosaminyl (N-acetyl) transferase 2, I-branching enzyme	Gcnt2	-2.85	0.004
1423471_at	polypyrimidine tract binding protein 2	Ptbp2	-2.85	0.009
1419906_at	Hydroxyprostaglandin dehydrogenase 15 (NAD)	Hpgd	-2.85	0.010
1427993_at	RUN and FYVE domain-containing 2	Rufy2	-2.86	0.008
1422780_at	peroxisomal membrane protein 4	Pxmp4	-2.86	0.002
1431861_a_at	DNA segment, Chr 3, ERATO Doi 250, expressed	D3Ertd250e	-2.87	0.008
1446731_at	hypothetical protein A730016A17	A730016A17	-2.87	0.000
1449209_a_at	retinol dehydrogenase 11	Rdh11	-2.87	0.005
1443873_at	DNA segment, Chr 18, ERATO Doi 653, expressed	D18Ertd653e	-2.87	0.005
1422064_a_at	zinc finger and BTB domain containing 20	Zbtb20	-2.88	0.008
1420043_s_at	THO complex 1	Thoc1	-2.88	0.005
1427371_at	ATP-binding cassette, sub-family A (ABC1), member 8a	Abca8a	-2.89	0.002
1418863_at	GATA binding protein 4	Gata4	-2.91	0.000
1442885_at	SREBP cleavage activating protein	Scap	-2.92	0.005
1448619_at	7-dehydrocholesterol reductase	Dhcr7	-2.94	0.003
1425793_a_at	RAR-related orphan receptor gamma	Rorc	-2.95	0.003
1450014_at	claudin 1	Cldn1	-2.95	0.005
1445596_at	Adenosine deaminase, RNA-specific	Adar	-2.95	0.002
1450271_at	PTK6 protein tyrosine kinase 6	Ptk6	-2.95	0.004
1455928_x_at	leucine-zipper-like transcriptional regulator, 1	Lztr1	-2.95	0.005
1450863_a_at	double cortin and calcium/calmodulin-dependent protein kinase-like 1	Dcamkl1	-2.96	0.002
1457076_at	gene model 693, (NCBI)	Gm693	-2.96	0.005
1459769_at	Hypothetical gene supported by AK076800; BC048609	LOC433215	-2.96	0.008
1452302_at	Rho guanine nucleotide exchange factor (GEF) 10	Arhgef10	-2.97	0.001
1440241_at	Transient receptor potential cation channel, subfamily M, member 1	Trpm1	-2.97	0.009
1437669_x_at	Chemokine (C-C motif) receptor-like 1	Ccrl1	-2.97	0.010
1438183_x_at	sorbitol dehydrogenase 1	Sdh1	-2.97	0.007
1449701_at	DNA segment, Chr 6, ERATO Doi 109, expressed	D6Ertd109e	-2.98	0.002
1426334_a_at	BCL2-like 11 (apoptosis facilitator)	Bcl2l11	-2.98	0.006
1421987_at	3'-phosphoadenosine 5'-phosphosulfate synthase 2	Papss2	-2.98	0.003
1423418_at	farnesyl diphosphate synthetase	Fdps	-2.99	0.004
1430573_s_at	ethanol decreased 2	Etohd2	-2.99	0.005
1457578_at	Peroxisome proliferative activated receptor, gamma, coactivator 1 beta	Ppargc1b	-2.99	0.007
1454244_at	minichromosome maintenance deficient 10 (S. cerevisiae)	Mcm10	-3.00	0.000
1441329_at	Galanin receptor 1	Galr1	-3.00	0.003
1446462_at	Histidyl tRNA synthetase 2	Hars2	-3.00	0.002
1455685_at	hypothetical protein 9530064J02	9530064J02	-3.01	0.001
1427512_a_at	laminin, alpha 3	Lama3	-3.01	0.007
1449970_at	calpain 12	Capn12	-3.02	0.003
1439964_at	DNA segment, Chr 8, Brigham & Women's Genetics 1414 expressed	D8Bwg1414e	-3.02	0.001
1433515_s_at	ethanolamine kinase 1	Etnk1	-3.02	0.005
1455867_at	SRY-box containing gene 4	Sox4	-3.04	0.009
1441984_at	claspin homolog (Xenopus laevis)	Clspn	-3.04	0.005
1455454_at	similar to 3(20)alpha-hydroxysteroid/dihydrodiol/indanol dehydrogenase	LOC432720	-3.04	0.007
1423199_at	bromodomain containing 3	Brd3	-3.05	0.003

1422095_a_at	thymidylate kinase family LPS-inducible member	Tyki	-3.05	0.002
1441175_at	Aristaless related homeobox gene (Drosophila)	Arx	-3.06	0.002
1425014_at	nuclear receptor subfamily 2, group C, member 2	Nr2c2	-3.07	0.007
1444663_at	Vascular endothelial zinc finger 1	Vezf1	-3.07	0.002
1453027_at	discs, large (Drosophila) homolog-associated protein 1	Dlgap1	-3.08	0.002
1442601_at	glucocorticoid modulatory element binding protein 1	Gmeb1	-3.08	0.007
1442918_at	neuron navigator 3	Nav3	-3.08	0.009
1440760_at	Lymphoid enhancer binding factor 1	Lef1	-3.09	0.004
1441536_at	3-hydroxy-3-methylglutaryl-Coenzyme A synthase 1	Hmgcs1	-3.10	0.008
1450202_at	glutamate receptor, ionotropic, NMDA1 (zeta 1)	Grin1	-3.11	0.003
1426135_a_at	parkin	Park2	-3.12	0.001
1418449_at	ladinin	Lad1	-3.12	0.006
1427723_at	growth differentiation factor 11	Gdf11	-3.12	0.008
1440296_at	gene model 994, (NCBI)	Gm994	-3.12	0.002
1437877_at	tetratricopeptide repeat domain 14	Ttc14	-3.13	0.004
1450725_s_at	carbonic anhydrase 14	Car14	-3.14	0.002
1434948_at	RAB11 family interacting protein 2 (class I)	Rab11fip2	-3.14	0.004
1422143_at	A kinase (PRKA) anchor protein 7	Akap7	-3.16	0.006
1424245_at	carboxylesterase 2	Ces2	-3.17	0.008
1423078_a_at	sterol-C4-methyl oxidase-like	Sc4mol	-3.18	0.008
1421913_at	mitochondrial ribosomal protein L19	Mrpl19	-3.19	0.001
1449504_at	karyopherin (importin) alpha 1	Kpna1	-3.19	0.007
1422073_a_at	cadherin EGF LAG seven-pass G-type receptor 2	Celsr2	-3.19	0.004
1422703_at	glycerol kinase	Gyk	-3.20	0.000
1447499_s_at	mosg protein	Mosg	-3.21	0.005
1447632_at	Fanconi anemia, complementation group C	Fancc	-3.22	0.010
1427893_a_at	phosphomevalonate kinase	Pmvk	-3.25	0.003
1419293_at	Down syndrome cell adhesion molecule	Dscam	-3.25	0.008
1444352_at	zinc finger protein 287	Zfp287	-3.25	0.010
1439479_at	lactase	Lct	-3.26	0.007
1417403_at	ELOVL family member 6, elongation of long chain fatty acids (yeast)	Elovl6	-3.26	0.003
1453191_at	procollagen, type XXVII, alpha 1	Col27a1	-3.26	0.002
1444178_at	hypothetical gene supported by AK028012	LOC433237	-3.27	0.001
1422996_at	mitochondrial acyl-CoA thioesterase 1	MGI:2159605	-3.28	0.002
1421829_at	adenylate kinase 3 alpha-like 1	Ak3l1	-3.30	0.004
1430896_s_at	nudix (nucleoside diphosphate linked moiety X)-type motif 7	Nudt7	-3.31	0.001
1439092_at	Nuclear RNA export factor 1 homolog (S. cerevisiae)	Nxf1	-3.31	0.004
1422493_at	coproporphyrinogen oxidase /// similar to Cpo protein	Cpox /// LOC436366	-3.32	0.001
1419931_at	ATP-binding cassette, sub-family B (MDR/TAP), member 7	Abcb7	-3.33	0.001
1433133_at	EDAR (ectodysplasin-A receptor)-associated death domain	Edaradd	-3.34	0.010
1437718_x_at	fibromodulin	Fmod	-3.34	0.000
1432181_s_at	endothelial cell growth factor 1 (platelet-derived)	Ecgf1	-3.34	0.000
1435166_at	contactin 2	Cntn2	-3.38	0.006
1421234_at	transcription factor 1	Tcf1	-3.39	0.009
1421091_at	serine (or cysteine) proteinase inhibitor, clade A member 12	Serpina12	-3.39	0.009
1422526_at	acyl-CoA synthetase long-chain family member 1	Acsl1	-3.39	0.007
1428166_at	congenital dyserythropoietic anemia, type I (human)	Cdan1	-3.39	0.003
1450128_at	phospholipase A2, group IIA (platelets, synovial fluid)	Pla2g2a	-3.42	0.000
1425948_a_at	solute carrier family 25, member 30	Slc25a30	-3.42	0.002
1416157_at	vinculin	Vcl	-3.43	0.008
1421501_a_at	Rab6 interacting protein 2	Rab6ip2	-3.45	0.001
1444442_at	oral cancer overexpressed 1	Oraov1	-3.45	0.003
1450956_at	stearoyl-coenzyme A desaturase 3	Scd3	-3.45	0.005
1434449_at	aquaporin 4	Aqp4	-3.46	0.003

1459973_x_at	dipeptidylpeptidase 4	Dpp4	-3.47	0.000
1423290_at	hypoxia up-regulated 1	Hyou1	-3.47	0.000
1444407_at	Hypothetical protein B230205M03	B230205M03	-3.50	0.003
1420894_at	transforming growth factor, beta receptor I	Tgfbr1	-3.50	0.007
1438294_at	spinocerebellar ataxia 1 homolog (human)	Sca1	-3.51	0.001
1421609_a_at	camello-like 3	Cml3	-3.54	0.001
1419312_at	ATPase, Ca++ transporting, cardiac muscle, fast twitch 1	Atp2a1	-3.55	0.008
1449109_at	suppressor of cytokine signaling 2	Socs2	-3.55	0.009
1421163_a_at	nuclear factor I/A	Nfia	-3.55	0.002
1430286_s_at	protein phosphatase 1, regulatory (inhibitor) subunit 14c	Ppp1r14c	-3.58	0.003
1442822_at	Calcium/calmodulin-dependent protein kinase IV	Camk4	-3.62	0.005
1435760_at	cystatin A	MGI:3524930	-3.63	0.000
1422930_at	intercellular adhesion molecule 4, Landsteiner-Wiener blood group	Icam4	-3.64	0.001
1416833_at	kidney expressed gene 1	Keg1	-3.68	0.004
1421515_at	nuclear receptor subfamily 6, group A, member 1	Nr6a1	-3.69	0.010
1423804_a_at	isopentenyl-diphosphate delta isomerase	Idi1	-3.71	0.000
1446467_at	ATPas, class II, type 9B	Atp9b	-3.72	0.003
1453500_at	cytochrome P450, family 2, subfamily u, polypeptide 1	Cyp2u1	-3.74	0.003
1422177_at	interleukin 13 receptor, alpha 2	Il13ra2	-3.80	0.003
1449385_at	hydroxysteroid (17-beta) dehydrogenase 9	Hsd17b9	-3.80	0.007
1420426_at	myosin VIIb	Myo7b	-3.82	0.007
1460659_at	Phosphatidylinositol transfer protein, beta	Pitpnb	-3.86	0.006
1447561_at	Plasmacytoma variant translocation 1	Pvt1	-3.87	0.005
1432012_a_at	NOL1/NOP2/Sun domain family 6	Nsun6	-3.87	0.005
AFFX-18SRNAMur/X00686_M_at	mannosidase 2, alpha B2	Man2b2	-3.90	0.000
1438844_x_at	spermatogenesis associated 5	Spata5	-3.93	0.002
1456970_at	Cadherin 7, type 2	Cdh7	-3.94	0.003
1439197_at	phosphatidylinositol 4-kinase, catalytic, beta polypeptide	Pik4cb	-3.97	0.005
1453487_at	dihydrodiol dehydrogenase (dimeric)	Dhdh	-3.97	0.003
1450032_at	solute carrier organic anion transporter family, member 2a1	Slco2a1	-3.99	0.001
1425640_at	homolog of human MLLT2 unidentified gene	Mllt2h	-4.03	0.005
1424352_at	similar to cytochrome P450, 4a10	MGC25972	-4.04	0.006
1455223_at	insulin-like growth factor 2, binding protein 1	Igf2bp1	-4.04	0.000
1425040_at	cytochrome b reductase 1	Cybrd1	-4.10	0.009
1421484_at	UDP glycosyltransferase 2 family, polypeptide A1	Ugt2a1	-4.13	0.007
1459306_at	Beaded filament structural protein 2, phakinin	Bfsp2	-4.14	0.000
1440059_at	Mitogen-activated protein kinase kinase kinase kinase 5	Map4k5	-4.21	0.001
1441598_at	transmembrane protein with EGF-like and two follistatin-like domains 2	Tmeff2	-4.22	0.007
1427052_at	acetyl-Coenzyme A carboxylase beta	Acacb	-4.22	0.000
1422478_a_at	acetyl-Coenzyme A synthetase 2 (ADP forming)	Acas2	-4.24	0.000
1422053_at	inhibin beta-A	Inhba	-4.30	0.009
1426732_at	desmin	Des	-4.31	0.003
1445807_at	Cleavage stimulation factor, 3' pre-RNA subunit 2, tau	Cstf2t	-4.35	0.003
1419921_s_at	Ubiquitin specific protease 7	Usp7	-4.36	0.010
1445243_at	DNA segment, Chr 10, ERATO Doi 533, expressed	D10Ertd533e	-4.36	0.006
1456354_at	cholinergic receptor, nicotinic, alpha polypeptide 4	Chrna4	-4.39	0.002
1418803_a_at	Fas ligand (TNF superfamily, member 6)	Fasl	-4.41	0.005
1457432_at	prospero-related homeobox 1	Prox1	-4.42	0.000
1454267_a_at	protease, serine, 32	Prss32	-4.44	0.010
1438978_x_at	necdin	Ndn	-4.44	0.010
1436320_at	CDNA clone IMAGE:1328649	---	-4.46	0.009
1423867_at	serine (or cysteine) proteinase inhibitor, clade A, member 3K	Serpina3k	-4.48	0.000

1426153_a_at	desmoglein 2	Dsg2	-4.48	0.003
1419683_at	TP53 regulating kinase	Trp53rk	-4.51	0.005
1427020_at	scavenger receptor class A, member 3	Scara3	-4.53	0.000
1420722_at	elongation of very long chain fatty acids (FEN1/Elo2, SUR4/Elo3, yeast)-like 3	Elovl3	-4.57	0.005
1420561_at	transient receptor potential cation channel, subfamily C, member 7	Trpc7	-4.61	0.000
1438699_at	Steroid 5 alpha-reductase 1	Srd5a1	-4.63	0.000
1456573_x_at	nicotinamide nucleotide transhydrogenase	Nnt	-4.66	0.006
1443642_at	TAO kinase 2	Taok2	-4.72	0.004
1450785_at	Thyroid hormone receptor interactor 4	Trip4	-4.72	0.002
1425409_at	cholinergic receptor, nicotinic, alpha polypeptide 2 (neuronal)	Chrna2	-4.74	0.006
1444146_at	ring finger protein 20	Rnf20	-4.75	0.002
1421259_at	pyruvate kinase liver and red blood cell	Pklr	-4.76	0.009
1444438_at	calcium and integrin binding family member 3	Cib3	-4.77	0.003
1454881_s_at	uroplakin 3B	Upk3b	-4.81	0.001
1438807_at	Heterogeneous nuclear ribonucleoprotein R	Hnrpr	-4.89	0.007
1420729_at	2-cell-stage, variable group, member 1	MGI:1933437	-4.90	0.002
1459547_at	Similar to RAB5B, member RAS oncogene family	---	-4.93	0.000
1425535_at	replication initiator 1	Repin1	-4.93	0.001
1434130_at	lipoma HMGIC fusion partner-like 2	Lhfpl2	-5.00	0.003
1441919_x_at	carnitine acetyltransferase	Crat	-5.15	0.007
1449309_at	cytochrome P450, family 8, subfamily b, polypeptide 1	Cyp8b1	-5.20	0.000
1420531_at	hydroxysteroid dehydrogenase-5, delta<5>-3-beta	Hsd3b5	-5.38	0.001
1418599_at	procollagen, type XI, alpha 1	Col11a1	-5.44	0.008
1459045_at	Golgi phosphoprotein 4	Golph4	-5.57	0.000
1435132_at	dispatched homolog 1 (Drosophila)	Disp1	-5.59	0.004
1453051_at	zinc finger with KRAB and SCAN domains 1	Zkscan1	-5.68	0.007
1442837_at	---	---	-5.70	0.000
1456974_at	one cut domain, family member 1	Onecut1	-5.80	0.001
1442458_at	Transcribed locus	---	-5.84	0.008
1424673_at	C-type lectin domain family 2, member h	Clec2h	-5.98	0.002
1418213_at	keratin complex 1, acidic, gene 23	Krt1-23	-6.00	0.000
1420379_at	solute carrier organic anion transporter family, member 1a1	Slco1a1	-6.06	0.002
1447445_at	Sidekick homolog 2 (chicken)	Sdk2	-6.11	0.001
1424811_at	camello-like 5	Cml5	-7.01	0.003
1421443_at	G protein-coupled receptor 110	Gpr110	-8.57	0.008
1436207_at	Transcription factor 3	Tcf3	-13.90	0.002
1448261_at	cadherin 1	Cdh1	-17.42	0.000
1448092_x_at	serine (or cysteine) proteinase inhibitor, clade A, member 4, pseudogene 1	Serpina4-ps1	-22.24	0.003
