# Supplementary material for: Impaired Genome Maintenance Suppresses the Growth Hormone–Insulin-Like Growth Factor 1 Axis in Mice with Cockayne Syndrome
Source: PLoS Biol. 2006 Dec 12;5(1):e2. doi: 10.1371/journal.pbio.0050002 (PMC1698505; doi:10.1371/journal.pbio.0050002)
Supplement: Supplementary file 4 [file pbio.0050002.st004.pdf]

Supplementary Table S4

Extensive overview of signficant expression changes in 96-week old wild-type mice compared to 8-week-old mice, FC: fold change, p: p-value.

			96W	
**Code**	**Title**	**Symbol**	**FC**	**p**
1452463_x_at	Immunoglobulin kappa chain, constant region	Igk-V8	16.10	0.008
1452417_x_at	immunoglobulin kappa chain, constant region	Igk-C /// Igk-V28 ///	14.11	0.001
1425837_a_at	CCR4 carbon catabolite repression 4-like (S. cerevisiae)	Ccrn4l	12.44	0.000
1450184_s_at	thyrotroph embryonic factor	Tef	12.17	0.000
1427351_s_at	immunoglobulin heavy chain 6 (heavy chain of IgM)	Igh-6	11.97	0.005
1450639_at	solute carrier family 28 (sodium-coupled nucleoside transporter), member 2	Slc28a2	11.61	0.004
1460437_at	pleckstrin homology, Sec7 and coiled/coil domains 4	Pscd4	10.40	0.007
1452794_x_at	spermatogenesis associated glutamate (E)-rich protein 1, pseudogene 1	Speer1-ps1	8.03	0.002
1422967_a_at	transferrin receptor	Tfrc	7.54	0.003
1417645_at	sarcospan	Sspn	7.40	0.000
1424305_at	immunoglobulin joining chain	Igj	7.31	0.007
1437370_at	shugoshin-like 2 (S. pombe)	Sgol2	6.67	0.000
1418830_at	CD79A antigen (immunoglobulin-associated alpha)	Cd79a	6.57	0.001
1440395_at	RNA binding motif protein 21	Rbm21	6.50	0.000
1448731_at	interleukin 10 receptor, alpha	Il10ra	6.32	0.005
1421146_at	Rap guanine nucleotide exchange factor (GEF) 1	Rapgef1	6.24	0.000
1448349_at	vesicle-associated membrane protein, associated protein A	Vapa	6.06	0.001
1436237_at	tetratricopeptide repeat domain 9	Ttc9	6.02	0.001
1420816_at	3-monooxygenase/tryptophan 5-monooxygenase activation protein, gamma polypeptide	Ywhag	5.82	0.000
1439426_x_at	P lysozyme structural	Lzp-s	5.74	0.009
1420148_at	solute carrier family 6 (neurotransmitter transporter, taurine), member 6	Slc6a6	5.71	0.007
1425578_a_at	glial cell line derived neurotrophic factor family receptor alpha 2	Gfra2	5.49	0.007
1450383_at	low density lipoprotein receptor	Ldlr	5.48	0.001
1418842_at	hematopoietic cell specific Lyn substrate 1	Hcls1	5.44	0.007
1448749_at	pleckstrin	Plek	5.32	0.002
1438392_at	CD3 antigen, zeta polypeptide	Cd3z	5.31	0.000
1451849_a_at	lamin B2	Lmnb2	5.15	0.000
1422726_x_at	spermatogenesis associated glutamate (E)-rich protein 4a	Speer4a	5.13	0.005
1450471_at	MAD homolog 3 (Drosophila)	Smad3	5.11	0.006
1432170_at	Ts translation elongation factor, mitochondrial	Tsfm	4.99	0.002
1419135_at	lymphotoxin B	Ltb	4.98	0.000

1427019_at	protein tyrosine phosphatase, receptor type Z, polypeptide 1	Ptprz1	4.97	0.005
1419466_at	naked cuticle 2 homolog (Drosophila)	Nkd2	4.84	0.000
1449941_at	myosin IE	Myo1e	4.82	0.000
1436312_at	zinc finger protein, subfamily 1A, 1 (Ikaros)	Zfpn1a1	4.75	0.003
1456584_x_at	3-phosphoglycerate dehydrogenase /// similar to 3-phosphoglycerate dehydrogenase	Phgdh /// LOC4342	4.72	0.009
1455747_at	gamma-glutamyltransferase-like activity 1	Ggtla1	4.65	0.002
1421898_a_at	major histocompatibility complex, class I-related	Mr1	4.56	0.000
1450753_at	natural killer cell group 7 sequence	Nkg7	4.48	0.008
1418076_at	suppression of tumorigenicity 14 (colon carcinoma)	St14	4.46	0.000
1420025_s_at	AP2 associated kinase 1	Aak1	4.42	0.001
1427575_at	F-box and WD-40 domain protein 14	Fbxw14	4.41	0.007
1445707_at	zinc finger, DHHC domain containing 6	Zdhhc6	4.30	0.003
1416226_at	actin related protein 2/3 complex, subunit 1B	Arpc1b	4.29	0.001
1451206_s_at	pleckstrin homology, Sec7 and coiled-coil domains, binding protein	Pscdbp	4.28	0.008
1447803_x_at	Capping protein (actin filament), gelsolin-like	Capg	4.25	0.007
1460416_s_at	component of Sp100-rs /// similar to homogeneously staining region protein - mouse	Csprs /// LOC4345	4.13	0.001
1451285_at	fusion, derived from t(12;16) malignant liposarcoma (human)	Fus	4.13	0.000
1419717_at	sema domain, immunoglobulin domain (Ig), short basic domain, secreted, (semaphorin) 3E	Sema3e	4.12	0.000
1427229_at	3-hydroxy-3-methylglutaryl-Coenzyme A reductase	Hmgcr	4.09	0.001
1443687_x_at	Histocompatibility 2, class II, locus Mb1	H2-DMb1	4.09	0.009
1429517_at	zinc finger, FYVE domain containing 20	Zfyve20	4.06	0.000
1416875_at	parvin, gamma	Parvg	4.04	0.004
1421753_a_at	LIM homeobox protein 3	Lhx3	4.02	0.000
1426010_a_at	erythrocyte protein band 4.1-like 3	Epb4.1l3	4.01	0.010
1417232_at	claudin 2	Cldn2	3.97	0.000
1451452_a_at	regulator of G-protein signaling 16	Rgs16	3.96	0.007
1451417_at	breast cancer 1	Brca1	3.96	0.003
1436808_x_at	minichromosome maintenance deficient 5, cell division cycle 46 (S. cerevisiae)	Mcm5	3.94	0.004
1441275_at	T-cell activation kelch repeat protein	MGI:2661430	3.94	0.001
1446415_at	heme binding protein 2	Hebp2	3.88	0.003
1422249_s_at	zinc finger protein, autosomal /// zinc finger protein X-linked	Zfa /// Zfx	3.82	0.004
1416959_at	nuclear receptor subfamily 1, group D, member 2	Nr1d2	3.80	0.004
1425283_a_at	metallothionein-like 5, testis-specific (tesmin)	Mtl5	3.76	0.000
1424560_at	proline-serine-threonine phosphatase-interacting protein 1	Pstpip1	3.76	0.004
1448698_at	cyclin D1	Ccnd1	3.70	0.007
1446650_at	Calmodulin binding transcription activator 1	Camta1	3.69	0.002

1420591_at	G protein-coupled receptor 84	Gpr84	3.67	0.001
1422188_s_at	T-cell receptor gamma chain /// T cell receptor gamma chain	Tcrg	3.66	0.009
1422200_at	olfactory marker protein	Omp	3.63	0.001
1417051_at	protocadherin 8	Pcdh8	3.62	0.002
1451772_at	two pore channel 1	Tpcn1	3.57	0.002
1417995_at	protein tyrosine phosphatase, non-receptor type 22 (lymphoid)	Ptpn22	3.56	0.006
1421505_at	Mix1 homeobox-like 1 (Xenopus laevis)	Mixl1	3.54	0.001
1430452_at	cytochrome P450, family 20, subfamily A, polypeptide 1	Cyp20a1	3.53	0.001
1426324_at	histocompatibility 2, D region locus 1	H2-D1	3.52	0.002
1426171_x_at	killer cell lectin-like receptor, subfamily A, member 7	Klra7	3.51	0.002
1426086_a_at	fragile X mental retardation syndrome 1 homolog	Fmr1	3.49	0.001
1454438_at	RAB27A, member RAS oncogene family	Rab27a	3.46	0.007
1419128_at	integrin alpha X	Itgax	3.45	0.001
1448777_at	minichromosome maintenance deficient 2 mitotin (S. cerevisiae)	Mcm2	3.44	0.001
1435191_at	corneodesmosin	Cdsn	3.42	0.003
1458910_at	Transforming growth factor beta regulated gene 3	Tbrg3	3.41	0.001
1441579_at	doublesex and mab-3 related transcription factor like family A1	Dmrta1	3.37	0.003
1444634_at	NOL1/NOP2/Sun domain family 3	Nsun3	3.36	0.004
1457123_at	neuregulin 4	Nrg4	3.36	0.001
1456536_at	THAP domain containing 6	Thap6	3.35	0.007
1420904_at	interleukin 17 receptor	Il17r	3.35	0.002
1421973_at	glial cell line derived neurotrophic factor family receptor alpha 1	Gfra1	3.32	0.005
1415773_at	nucleolin	Ncl	3.30	0.000
1451691_at	endothelin receptor type A	Ednra	3.29	0.003
1422313_a_at	insulin-like growth factor binding protein 5	Igfbp5	3.29	0.003
1415877_at	dihydropyrimidinase-like 3	Dpysl3	3.29	0.003
1449975_a_at	parkin	Park2	3.27	0.005
1441634_at	netrin G1	Ntng1	3.27	0.004
1458952_at	WD repeat and FYVE domain containing 3	Wdfy3	3.25	0.006
1419647_a_at	immediate early response 3	Ier3	3.24	0.003
1418207_at	FXYD domain-containing ion transport regulator 4	Fxyd4	3.22	0.001
1432399_a_at	Eph receptor A1	Epha1	3.20	0.008
1457877_at	Estrogen receptor 1 (alpha)	Esr1	3.20	0.002
1415843_at	G protein beta subunit-like	MGI:1929514	3.18	0.003
1425620_at	transforming growth factor, beta receptor III	Tgfbr3	3.17	0.002
1451660_a_at	homeo box B6	Hoxb6	3.13	0.010

1432098_a_at	olfactory receptor 701	Olfr701	3.12	0.008
1418738_at	sodium channel, voltage-gated, type I, beta	Scn1b	3.12	0.001
1424566_s_at	polymerase (RNA) III (DNA directed) polypeptide D	Polr3d	3.08	0.002
1457106_at	thyroid adenoma associated	Thada	3.08	0.003
1418020_s_at	carboxypeptidase D	Cpd	3.08	0.003
1454289_at	zinc fingers and homeoboxes 3	Zhx3	3.07	0.003
1426147_s_at	claudin 10	Cldn10	3.07	0.005
1444083_at	titin	Ttn	3.06	0.004
1416562_at	glutamic acid decarboxylase 1	Gad1	3.06	0.004
1445727_at	ubiquitin protein ligase E3A	Ube3a	3.05	0.006
1428492_at	GLI pathogenesis-related 2	Glipr2	3.05	0.004
1452787_a_at	heterogeneous nuclear ribonucleoproteins methyltransferase-like 2 (S. cerevisiae)	Hrmt1l2	3.04	0.000
1455086_at	Ubiquitin carboxyl-terminal esterase L5	Uchl5	3.03	0.005
1423704_at	lysophospholipase 3	Lypla3	3.03	0.001
1431699_at	fucosyltransferase 4, pseudogene 1	Fut4-ps1	3.02	0.002
1419323_at	peptidyl arginine deiminase, type I	Padi1	3.01	0.001
1417015_at	Ras association (RalGDS/AF-6) domain family 3	Rassf3	2.99	0.004
1445573_at	Protein phosphatase 1, regulatory (inhibitor) subunit 14B	Ppp1r14b	2.99	0.005
1453913_a_at	transporter 2, ATP-binding cassette, sub-family B (MDR/TAP)	Tap2	2.99	0.001
1449708_s_at	checkpoint kinase 1 homolog (S. pombe)	Chek1	2.99	0.005
1416111_at	CD83 antigen	Cd83	2.98	0.006
1430729_at	melanocyte proliferating gene 1	MGI:1929864	2.97	0.003
1422979_at	suppressor of variegation 3-9 homolog 2 (Drosophila)	Suv39h2	2.97	0.006
1422567_at	niban protein	Niban	2.96	0.006
1425753_a_at	uracil-DNA glycosylase	Ung	2.94	0.002
1450326_at	src homology 2 domain-containing transforming protein C3	Shc3	2.94	0.007
1460613_x_at	growth hormone	Gh	2.94	0.008
1421525_a_at	baculoviral IAP repeat-containing 1e	Birc1e	2.92	0.003
1450143_at	RAS guanyl releasing protein 1	Rasgrp1	2.92	0.002
1448074_at	Relaxin 1	Rln1	2.92	0.006
1452819_at	latrophilin 3	Lphn3	2.89	0.008
1422280_at	granzyme K	Gzmk	2.89	0.000
1449508_at	interleukin 27 receptor, alpha	Il27ra	2.89	0.001
1416936_at	apoptosis-associated tyrosine kinase	Aatk	2.89	0.005
1423433_at	Sjogren syndrome antigen A2	Ssa2	2.87	0.003
1432558_a_at	myelin and lymphocyte protein, T-cell differentiation protein	Mal	2.86	0.006

1439303_at	Neuronal growth regulator 1	Negr1	2.86	0.003
1450585_at	taste receptor, type 2, member 119	Tas2r119	2.86	0.003
1420975_at	bromodomain adjacent to zinc finger domain, 1B	Baz1b	2.86	0.000
1446553_at	Hemopoietic cell kinase	Hck	2.85	0.007
1421715_at	tissue-type vomeronasal neurons putative pheromone receptor V2R2	V2R2	2.85	0.002
1449976_a_at	G protein-coupled receptor 35	Gpr35	2.84	0.006
1449580_s_at	histocompatibility 2, class II, locus Mb1 /// histocompatibility 2, class II, locus Mb2	H2-DMb1 /// H2-DI	2.84	0.009
1440310_at	CBFA2T1 identified gene homolog (human)	Cbfa2t1h	2.84	0.003
1437270_a_at	Cardiotrophin-like cytokine factor 1	Bsf3	2.84	0.003
1448268_at	transmembrane emp24 protein transport domain containing 9	Tmed9	2.83	0.000
1439407_x_at	transgelin 2	Tagln2	2.82	0.002
1419049_at	pecanex homolog (Drosophila)	Pcnx	2.82	0.000
1422969_s_at	inositol hexaphosphate kinase 1	Ihpk1	2.81	0.001
1436459_at	Gene model 208, (NCBI)	Gm208Gpr	2.81	0.001
1449365_at	endothelial differentiation, sphingolipid G-protein-coupled receptor, 8	Edg8	2.80	0.000
1439489_at	G protein-coupled receptor 120	Gpr120	2.80	0.001
1438665_at	sphingomyelin phosphodiesterase 3, neutral	Smpd3	2.80	0.009
1444233_at	G protein-coupled receptor 132	Gpr132	2.79	0.009
1426610_a_at	transcription termination factor 1	Ttf1	2.79	0.005
1426653_at	minichromosome maintenance deficient 3 (S. cerevisiae)	Mcm3	2.79	0.005
1443381_at	Ets variant gene 4 (E1A enhancer binding protein, E1AF)	Etv4	2.78	0.006
1422566_at	transcription factor EB	Tcfeb	2.78	0.002
1451604_a_at	activin A receptor, type II-like 1	Acvrl1	2.76	0.008
1417289_at	pleckstrin homology domain-containing, family A (phosphoinositide binding specific) member 2	Plekha2	2.76	0.005
1448997_at	pleckstrin homology, Sec7 and coiled-coil domains 1	Pscd1	2.76	0.009
1450379_at	moesin	Msn	2.75	0.009
1452899_at	RNA imprinted and accumulated in nucleus	Rian	2.75	0.008
1421813_a_at	prosaposin	Psap	2.74	0.002
1449220_at	GTPase, IMAP family member 3	Gimap3	2.74	0.005
1456709_at	oligophrenin 1	Ophn1	2.73	0.004
1420901_a_at	hexokinase 1	Hk1	2.72	0.005
1449015_at	resistin like alpha	Retnla	2.72	0.007
1422039_at	tumor necrosis factor receptor superfamily, member 22	Tnfrsf22	2.72	0.006
1429524_at	myosin IF	Myo1f	2.71	0.003
1426634_at	solute carrier family 5 (sodium/glucose cotransporter), member 9	Slc5a9	2.71	0.006
1416295_a_at	interleukin 2 receptor, gamma chain	Il2rg	2.70	0.005

1423048_a_at	toll interacting protein	Tollip	2.68	0.002
1427258_at	tripartite motif protein 24	Trim24	2.68	0.002
1422901_at	meningioma expressed antigen 5 (hyaluronidase)	Mgea5	2.68	0.000
1418140_at	doublecortin	Dcx	2.66	0.007
1450447_at	hepatic nuclear factor 4, alpha	Hnf4a	2.65	0.002
1458708_at	Bromodomain containing 4	Brd4	2.65	0.000
1422814_at	calmodulin binding protein 1	Calmbp1	2.64	0.002
1452410_a_at	feline sarcoma oncogene	Fes	2.64	0.003
1436482_a_at	syndecan 3	Sdc3	2.64	0.009
1421789_s_at	ADP-ribosylation factor 3	Arf3	2.62	0.003
1435180_at	podocan	Podn	2.62	0.003
1422937_at	frizzled homolog 5 (Drosophila)	Fzd5	2.61	0.003
1418422_at	serine (or cysteine) proteinase inhibitor, clade B, member 9g	Serpinb9g	2.61	0.010
1423166_at	CD36 antigen	Cd36	2.61	0.003
1448951_at	tumor necrosis factor receptor superfamily, member 1b	Tnfrsf1b	2.61	0.006
1426825_at	formin-like 3	Fmnl3	2.61	0.008
1426258_at	sortilin-related receptor, LDLR class A repeats-containing	Sorl1	2.61	0.001
1449373_at	DnaJ (Hsp40) homolog, subfamily C, member 3	Dnajc3	2.60	0.003
1434641_x_at	seizure related 6 homolog (mouse)-like 2	Sez6l2	2.59	0.005
1423228_at	UDP-Gal:betaGlcNAc beta 1,4-galactosyltransferase, polypeptide 6	B4galt6	2.59	0.009
1452205_x_at	T-cell receptor beta, variable 13	Tcrb-V13	2.58	0.006
1456431_at	Zinc finger protein 64	Zfp64	2.57	0.000
1417832_at	SMC (structural maintenance of chromosomes 1)-like 1 (S. cerevisiae)	Smc1l1	2.57	0.008
1431750_at	Usher syndrome 2A (autosomal recessive, mild) homolog (human)	Ush2a	2.57	0.009
1439617_s_at	phosphoenolpyruvate carboxykinase 1, cytosolic	Pck1	2.56	0.005
1457222_at	cAMP responsive element binding protein 5	Creb5	2.54	0.010
1427867_at	Myosin, heavy polypeptide 1, skeletal muscle, adult	Myh1	2.54	0.005
1436074_at	cDNA sequence AY078069	AY078069	2.53	0.008
1417752_at	coronin, actin binding protein 1C	Coro1c	2.53	0.005
1456288_at	schlafen 5	Slfn5	2.52	0.007
1426572_at	malic enzyme 2, NAD(+)-dependent, mitochondrial	Me2	2.52	0.009
1422546_at	interleukin enhancer binding factor 3	Ilf3	2.51	0.004
1431095_a_at	Hect domain and RLD 5	Herc5	2.50	0.009
1435802_at	gene model 157, (NCBI)	Gm157	2.50	0.001
1449347_a_at	X-linked lymphocyte-regulated 4	Xlr4	2.49	0.005
1449262_s_at	lin 7 homolog c (C. elegans)	Lin7c	2.49	0.002

1427298_at	neoplastic progression 1	Npn1	2.49	0.002
1460219_at	myelin-associated glycoprotein	Mag	2.48	0.000
1420506_a_at	syntaxin binding protein 1	Stxbp1	2.48	0.006
1421287_a_at	platelet/endothelial cell adhesion molecule 1	Pecam1	2.47	0.010
1415850_at	RAS p21 protein activator 3	Rasa3	2.47	0.002
1451484_a_at	synapsin I	Syn1	2.47	0.002
1435367_at	mitogen-activated protein kinase 4	Mapk4	2.46	0.004
1425750_a_at	Janus kinase 3	Jak3	2.45	0.002
1432344_a_at	amyloid beta (A4) precursor-like protein 2	Aplp2	2.45	0.003
1448467_a_at	tangerin	LOC114601	2.45	0.001
1424902_at	plexin domain containing 1	Plxdc1	2.45	0.002
1417025_at	histocompatibility 2, class II antigen E beta	H2-Eb1	2.44	0.008
1450747_at	kelch-like ECH-associated protein 1	Keap1	2.44	0.001
1450378_at	TAP binding protein	Tapbp	2.44	0.005
1429022_at	adenylate cyclase activating polypeptide 1 receptor 1	Adcyap1r1	2.44	0.002
1438960_at	Calcium/calmodulin-dependent protein kinase IV	Camk4	2.43	0.004
1460732_a_at	periplakin	Ppl	2.43	0.007
1428156_at	guanine nucleotide binding protein (G protein), gamma 2 subunit	Gng2	2.43	0.001
1422231_a_at	tumor necrosis factor receptor superfamily, member 25	Tnfrsf25	2.42	0.001
1450104_at	a disintegrin and metalloprotease domain 10	Adam10	2.41	0.006
1418026_at	exonuclease 1	Exo1	2.41	0.001
1435356_at	F-box protein 42	Fbxo42	2.39	0.008
1452154_at	isoleucine-tRNA synthetase	Iars	2.39	0.000
1434282_at	inhibitor of Bruton agammaglobulinemia tyrosine kinase	Ibtk	2.38	0.004
1417960_at	cytoplasmic polyadenylation element binding protein 1	Cpeb1	2.37	0.002
1427478_at	ubiquitin specific protease 12	Usp12	2.37	0.009
1441593_at	Phosphatase and tensin homolog	Pten	2.36	0.007
1446562_at	SH3-domain kinase binding protein 1	Sh3kbp1	2.36	0.003
1438682_at	phosphatidylinositol 3-kinase, regulatory subunit, polypeptide 1 (p85 alpha)	Pik3r1	2.36	0.000
1419714_at	CD274 antigen	Cd274	2.35	0.003
1450163_a_at	Werner syndrome homolog (human)	Wrn	2.35	0.004
1420408_a_at	ATP-binding cassette, sub-family C (CFTR/MRP), member 9	Abcc9	2.34	0.004
1416191_at	Sec61 alpha 1 subunit (S. cerevisiae)	Sec61a1	2.34	0.001
1438230_at	protein geranylgeranyltransferase type I, beta subunit	Pggt1b	2.34	0.003
1452457_a_at	protein kinase, AMP-activated, beta 1 non-catalytic subunit	Prkab1	2.34	0.000
1426004_a_at	transglutaminase 2, C polypeptide	Tgm2	2.33	0.007

1451987_at	arrestin, beta 2	Arrb2	2.33	0.005
1451733_at	glucosaminyl (N-acetyl) transferase 2, I-branching enzyme	Gcnt2	2.33	0.002
1417634_at	superoxide dismutase 3, extracellular	Sod3	2.33	0.008
1448541_at	kinesin 2	Kns2	2.33	0.008
1421916_at	platelet derived growth factor receptor, alpha polypeptide	Pdgfra	2.33	0.000
1446301_at	Phenylalanine-tRNA synthetase-like, alpha subunit	Farsla	2.32	0.002
1455108_at	eukaryotic translation initiation factor 4E member 2	Eif4e2	2.32	0.001
1421869_at	tripartite motif-containing 44	Trim44	2.31	0.007
1454011_a_at	replication protein A2	Rpa2	2.31	0.003
1434661_at	synaptogyrin 1	Syngr1	2.31	0.004
1433916_at	vesicle-associated membrane protein 3	Vamp3	2.31	0.004
1450703_at	solute carrier family 7 (cationic amino acid transporter, y+ system), member 2	Slc7a2	2.31	0.003
1453472_a_at	SLAM family member 7	Slamf7	2.30	0.006
1451241_at	laminin B1 subunit 1	Lamb1-1	2.30	0.002
1416988_at	mutS homolog 2 (E. coli)	Msh2	2.29	0.000
1433267_at	inhibitor of growth family, member 1	Ing1	2.29	0.010
1421344_a_at	ajuba	Jub	2.29	0.004
1453628_s_at	leucine rich repeat containing 2	Lrrc2	2.29	0.004
1437658_a_at	RNA, U22 small nucleolar	Rnu22	2.27	0.003
1426191_a_at	Bcl2-like 1	Bcl2l1	2.27	0.001
1449551_at	myosin IC	Myo1c	2.25	0.002
1422783_a_at	keratin complex 2, basic, gene 6a	Krt2-6a	2.24	0.010
1450241_a_at	ecotropic viral integration site 2a	Evi2a	2.24	0.001
1423588_at	actin related protein 2/3 complex, subunit 4	Arpc4	2.23	0.001
1429706_at	IQ motif containing F5	Iqcf5	2.23	0.003
1432236_a_at	suppressor of variegation 3-9 homolog 1 (Drosophila)	Suv39h1	2.23	0.000
1419758_at	ATP-binding cassette, sub-family B (MDR/TAP), member 1A	Abcb1a	2.22	0.000
1448607_at	pre-B-cell colony-enhancing factor 1	Pbef1	2.22	0.001
1436625_at	Fc receptor, IgG, high affinity I	Fcgr1	2.22	0.006
1450166_at	iduronate 2-sulfatase	Ids	2.22	0.006
1453666_at	ring finger protein 32	Rnf32	2.21	0.004
1456788_at	mitochondrial ribosomal protein L54	Mrpl54	2.21	0.007
1438417_at	PWWP domain containing 2	Pwwp2	2.20	0.010
1433504_at	brain glycogen phosphorylase	Pygb	2.20	0.000
1431274_a_at	heat shock protein 9A	Hspa9a	2.20	0.001
1431202_at	hect domain and RLD 3	Herc3	2.19	0.000

1448409_at	lymphoid-restricted membrane protein	Lrmp	2.19	0.008
1430984_at	ornithine decarboxylase antizyme inhibitor	Oazin	2.19	0.001
1417696_at	sterol O-acyltransferase 1	Soat1	2.18	0.009
1434677_at	Hermansky-Pudlak syndrome 5 homolog (human)	Hps5	2.18	0.003
1448144_at	heterogeneous nuclear ribonucleoprotein A/B	Hnrpab	2.17	0.010
1426415_a_at	tripartite motif protein 25	Trim25	2.17	0.001
1424143_a_at	retroviral integration site 2	Ris2	2.17	0.003
1452789_at	stannin	Snn	2.17	0.001
1455656_at	B and T lymphocyte associated	Btla	2.16	0.001
1438865_at	Histocompatibility 13	H13	2.16	0.006
1436321_at	UDP-GlcNAc:betaGal beta-1,3-N-acetylglucosaminyltransferase 7	B3gnt7	2.16	0.007
1425862_a_at	phosphatidylinositol 3-kinase, C2 domain containing, alpha polypeptide	Pik3c2a	2.15	0.003
1456486_at	zinc finger protein 574	Zfp574	2.14	0.007
1416012_at	EH-domain containing 1	Ehd1	2.14	0.006
1438453_at	Rad51 homolog c (S. cerevisiae)	Rad51c	2.13	0.003
1459009_at	Utrophin	Utrn	2.12	0.002
1451927_a_at	mitogen activated protein kinase 14	Mapk14	2.12	0.002
1422512_a_at	opioid growth factor receptor	Ogfr	2.11	0.000
1451195_a_at	thioredoxin domain containing 1	Txndc1	2.11	0.001
1418795_at	CDP-diacylglycerol synthase (phosphatidate cytidylyltransferase) 2	Cds2	2.11	0.010
1419186_a_at	ST8 alpha-N-acetyl-neuraminide alpha-2,8-sialyltransferase 4	St8sia4	2.10	0.009
1430332_a_at	glucuronidase, beta	Gusb	2.10	0.003
1426333_a_at	inhibitor of kappaB kinase beta	Ikbkb	2.10	0.003
1424317_at	solute carrier family 25 (mitochondrial deoxynucleotide carrier), member 19	Slc25a19	2.10	0.007
1450141_at	ATP-binding cassette, sub-family G (WHITE), member 3	Abcg3	2.09	0.008
1416214_at	minichromosome maintenance deficient 4 homolog (S. cerevisiae)	Mcm4	2.09	0.005
1427344_s_at	RASD family, member 2	Rasd2	2.09	0.009
1418692_at	RAB8A, member RAS oncogene family	Rab8a	2.08	0.003
1460283_at	Mediterranean fever	Mefv	2.08	0.006
1417141_at	interferon gamma induced GTPase	Igtp	2.08	0.009
1428150_at	coronin 7	Coro7	2.08	0.006
1448127_at	ribonucleotide reductase M1	Rrm1	2.08	0.000
1418082_at	N-myristoyltransferase 1	Nmt1	2.07	0.002
1427912_at	carbonyl reductase 3	Cbr3	2.07	0.001
1422701_at	zeta-chain (TCR) associated protein kinase	Zap70	2.07	0.004
1431686_a_at	glia maturation factor, beta	Gmfb	2.07	0.008

1460273_a_at	baculoviral IAP repeat-containing 1b	Birc1b	2.07	0.000
1433670_at	epithelial membrane protein 2	Emp2	2.06	0.007
1422660_at	RNA binding motif protein 3	Rbm3	2.06	0.001
1444563_at	UDP-Gal:betaGlcNAc beta 1,4-galactosyltransferase, polypeptide 5	B4galt5	2.06	0.010
1416835_s_at	S-adenosylmethionine decarboxylase 1 /// S-adenosylmethionine decarboxylase 2	Amd1 /// Amd2	2.05	0.002
1439570_at	gene model 444, (NCBI)	Gm444	2.05	0.002
1420653_at	transforming growth factor, beta 1	Tgfb1	2.05	0.006
1431030_a_at	ring finger protein 14	Rnf14	2.05	0.002
1424167_a_at	phosphomannomutase 1	Pmm1	2.05	0.002
1450106_a_at	Ena-vasodilator stimulated phosphoprotein	Evl	2.05	0.010
1416365_at	heat shock protein 1, beta	Hspcb	2.04	0.002
1417938_at	RAD51 associated protein 1	Rad51ap1	2.04	0.006
1454633_at	ethanolamine kinase 1	Etnk1	2.03	0.002
1450846_at	basic leucine zipper and W2 domains 1	Bzw1	2.03	0.004
1448624_at	CD2 antigen (cytoplasmic tail) binding protein 2	Cd2bp2	2.02	0.001
1427255_s_at	zinc finger protein 445	Zfp445	2.02	0.008
1434089_at	synaptopodin	Synpo	2.02	0.000
1431464_a_at	phosphomannomutase 2	Pmm2	2.02	0.003
1435721_at	Potassium voltage-gated channel, subfamily Q, member 4	Kcnq4	2.01	0.008
1451795_at	target of myb1-like 2 (chicken)	Tom1l2	2.01	0.009
1418194_at	UDP-N-acetyl-alpha-D-galactosamine:polypeptide N-acetylgalactosaminyltransferase 10	Galnt10	2.01	0.006
1440855_at	Machado-Joseph disease homolog (human)	Mjd	2.01	0.001
1426970_a_at	ubiquitin-activating enzyme E1-like /// RIKEN cDNA D330022A01 gene	Ube1l	2.01	0.004
1425008_a_at	interferon activated gene 203	Ifi203	2.01	0.005
1426993_at	xenotropic and polytropic retrovirus receptor 1	Xpr1	2.00	0.003
1443842_at	ADP-ribosylation factor guanine nucleotide-exchange factor 2 (brefeldin A-inhibited)	Arfgef2	1.99	0.000
1450223_at	apoptotic protease activating factor 1	Apaf1	1.98	0.005
1420899_at	RAB18, member RAS oncogene family	Rab18	1.98	0.003
1426297_at	transcription factor E2a	Tcfe2a	1.98	0.000
1450648_s_at	histocompatibility 2, class II antigen A, beta 1 /// response to metastatic cancers 1	H2-Ab1 /// Rmcs1	1.97	0.005
1436954_at	Wiskott-Aldrich syndrome protein interacting protein	Waspip	1.97	0.003
1452229_at	Sperm associated antigen 7	Spag7	1.97	0.001
1450245_at	solute carrier family 10, member 2	Slc10a2	1.97	0.004
1451256_at	G protein-coupled receptor 172B	Gpr172b	1.97	0.002
1430692_a_at	Sel1 (suppressor of lin-12) 1 homolog (C. elegans)	Sel1h	1.97	0.010
1416640_at	potassium voltage-gated channel, Isk-related family, member 1-like	Kcne1l	1.97	0.000

1418729_at	steroidogenic acute regulatory protein	Star	1.96	0.009
1441931_x_at	glutathione synthetase	Gss	1.96	0.004
1452801_at	phosphatidylinositol glycan, class K	Pigk	1.96	0.002
1446536_at	sema domain, transmembrane domain (TM), and cytoplasmic domain, (semaphorin) 6D	Sema6d	1.96	0.007
1454735_at	outer dense fiber of sperm tails 2	Odf2	1.96	0.005
1433621_at	WD repeat domain 41	Wdr41	1.95	0.000
1459546_s_at	ectonucleotide pyrophosphatase/phosphodiesterase 1	Enpp1	1.95	0.009
1451658_a_at	polymerase (RNA) III (DNA directed) polypeptide C	Polr3c	1.95	0.005
1433977_at	heparan sulfate (glucosamine) 3-O-sulfotransferase 3B1	Hs3st3b1	1.95	0.000
1422006_at	protein kinase, interferon-inducible double stranded RNA dependent	Prkr	1.95	0.000
1422827_x_at	solute carrier family 35 (UDP-galactose transporter), member 2	Slc35a2	1.95	0.000
1449511_a_at	single stranded DNA binding protein 4	Ssbp4	1.94	0.007
1426434_at	transmembrane protein 43	Tmem43	1.93	0.005
1415957_a_at	novel nuclear protein 1	Nnp1	1.93	0.001
1428132_at	CDC42 small effector 1	Cdc42se1	1.93	0.001
1445892_at	Period homolog 2 (Drosophila)	Per2	1.93	0.005
1448857_a_at	zinc finger, DHHC domain containing 12	Zdhhc12	1.92	0.000
1452233_at	ATP-binding cassette, sub-family C (CFTR/MRP), member 1	Abcc1	1.92	0.007
1459391_at	ATP-binding cassette, sub-family A (ABC1), member 5	Abca5	1.91	0.004
1421315_s_at	cortactin	Cttn	1.91	0.000
1435549_at	transient receptor potential cation channel, subfamily M, member 4	Trpm4	1.91	0.010
1436199_at	Coronin, actin binding protein 2A	Coro2a	1.91	0.003
1447606_x_at	aquaporin 11	Aqp11	1.91	0.009
1443954_at	RAD18 homolog (S. cerevisiae)	Rad18	1.91	0.005
1438040_a_at	tumor rejection antigen gp96	Tra1	1.91	0.009
1452630_at	triggering receptor expressed on myeloid cells-like 1	Treml1	1.90	0.010
1460057_at	growth differentiation factor 3	Gdf3	1.90	0.001
1417744_a_at	v-ral simian leukemia viral oncogene homolog B (ras related)	Ralb	1.90	0.003
1452629_at	scaffold attachment factor B2	Safb2	1.90	0.002
1419222_at	thromboxane A2 receptor	Tbxa2r	1.90	0.002
1424201_a_at	SEH1-like (S. cerevisiae	Seh1l	1.89	0.003
1426433_at	myc target 1	Myct1	1.89	0.002
1453612_at	NIMA (never in mitosis gene a)-related expressed kinase 1	Nek1	1.89	0.009
1425643_at	glycophorin A	Gypa	1.89	0.000
1451544_at	TAP binding protein-like	Tapbpl	1.89	0.003
1425762_a_at	retinoid X receptor alpha	Rxra	1.88	0.007

1417813_at	inhibitor of kappaB kinase epsilon	Ikbke	1.88	0.001
1431022_at	synuclein, alpha	Snca	1.88	0.008
1426049_a_at	telomeric repeat binding factor 2, interacting protein	Terf2ip	1.88	0.003
1435340_at	jumonji domain containing 2A	Jmjd2a	1.88	0.002
1425059_at	HMT1 hnRNP methyltransferase-like 6 (S. cerevisiae)	Hrmt1l6	1.87	0.003
1457256_x_at	Patched homolog 2	Ptch2	1.87	0.007
1452928_at	ABI gene family, member 3	Abi3	1.87	0.008
1451892_at	klotho	Kl	1.87	0.004
1434225_at	SWA-70 protein	MGI:1298390	1.86	0.008
1442842_at	Hspb associated protein 1	Hspbap1	1.86	0.009
1428549_at	coiled-coil domain containing 3	Ccdc3	1.86	0.008
1427094_at	polymerase (DNA directed), epsilon 2 (p59 subunit)	Pole2	1.85	0.003
1437820_at	forkhead-like 18 (Drosophila)	Fkhl18	1.85	0.005
1422681_at	SH2 domain binding protein 1 (tetratricopeptide repeat containing)	Sh2bp1	1.85	0.006
1416657_at	thymoma viral proto-oncogene 1	Akt1	1.85	0.001
1428697_at	dipeptidylpeptidase 8	Dpp8	1.84	0.000
1423172_at	N-ethylmaleimide sensitive fusion protein attachment protein beta	Napb	1.84	0.008
1426873_s_at	junction plakoglobin	Jup	1.84	0.001
1451680_at	neoplastic progression 3	Npn3	1.84	0.007
1449273_at	cytoplasmic FMR1 interacting protein 2	Cyfip2	1.84	0.010
1425880_x_at	zinc finger protein 352	Zfp352	1.83	0.001
1425670_at	regulatory factor X-associated ankyrin-containing protein	Rfxank	1.83	0.009
1421857_at	a disintegrin and metalloproteinase domain 17	Adam17	1.83	0.006
1450074_at	kinesin family member 3B	Kif3b	1.83	0.003
1456250_x_at	transforming growth factor, beta induced	Tgfbi	1.82	0.002
1439113_at	hippocalcin-like 1	Hpcal1 /// 2410018	1.82	0.000
1426471_at	zinc finger protein 52	Zfp52	1.82	0.007
1425592_at	transportin 2 (importin 3, karyopherin beta 2b)	Tnpo2	1.82	0.003
1433512_at	Friend leukemia integration 1	Fli1	1.82	0.005
1438207_at	golgi-specific brefeldin A-resistance factor 1	Gbf1	1.82	0.010
1421605_a_at	aquaporin 9	Aqp9	1.82	0.005
1426913_at	lanosterol synthase	Lss	1.81	0.006
1435039_a_at	phosphatidylinositol-4-phosphate 5-kinase, type 1 beta	Pip5k1b	1.81	0.004
1442927_at	PTK2 protein tyrosine kinase 2 beta	Ptk2b	1.81	0.000
1454667_at	sorting nexin 8	Snx8	1.81	0.007
1416550_at	solute carrier family 35, member B4	Slc35b4	1.80	0.009

1450408_at	chloride channel 7	Clcn7	1.79	0.003
1460727_at	excision repair cross-complementing rodent repair deficiency, complementation group 2	Ercc2	1.79	0.003
1421109_at	camello-like 2	Cml2	1.79	0.006
1429267_at	thioesterase, adipose associated	Thea	1.78	0.009
1439397_at	expressed sequence BB164513	BB164513	1.78	0.003
1423531_a_at	heterogeneous nuclear ribonucleoprotein A1	Hnrpa1	1.78	0.004
1424156_at	retinoblastoma-like 1 (p107)	Rbl1	1.78	0.002
1438683_at	WAS protein family, member 2	Wasf2	1.77	0.005
1427646_a_at	rho/rac guanine nucleotide exchange factor (GEF) 2	Arhgef2	1.77	0.003
1423528_at	breast carcinoma amplified sequence 3	Bcas3	1.77	0.004
1458159_at	Phosphatidylethanolamine binding protein	Pbp	1.77	0.002
1429776_a_at	DnaJ (Hsp40) homolog, subfamily B, member 6	Dnajb6	1.77	0.003
1417757_at	unc-13 homolog B (C. elegans)	Unc13b	1.77	0.005
1434357_a_at	karyopherin (importin) beta 1	Kpnb1	1.76	0.009
1452292_at	Adaptor-related protein complex 2, beta 1 subunit	Ap2b1	1.76	0.002
1448870_at	latent transforming growth factor beta binding protein 1	Ltbp1	1.76	0.007
1455758_at	Protein kinase C, gamma	Prkcc	1.75	0.007
1450403_at	signal transducer and activator of transcription 2	Stat2	1.75	0.002
1446646_at	TAF3 RNA polymerase II, TATA box binding protein (TBP)-associated factor	Taf3	1.75	0.007
1453946_a_at	serologically defined colon cancer antigen 8	Sdccag8	1.75	0.004
1449121_at	FUS interacting protein (serine-arginine rich) 1	Fusip1	1.75	0.002
1450779_at	fatty acid binding protein 7, brain	Fabp7	1.75	0.009
1427652_x_at	synaptojanin 2	Synj2	1.74	0.006
1426698_a_at	heterogeneous nuclear ribonucleoprotein M	Hnrpm	1.74	0.003
1425517_s_at	O-linked N-acetylglucosamine (GlcNAc) transferase	Ogt	1.74	0.005
1440814_x_at	heparan sulfate (glucosamine) 3-O-sulfotransferase 2	Hs3st2	1.74	0.010
1451983_at	Iroquois related homeobox 1 (Drosophila)	Irx1	1.74	0.008
1422990_at	met proto-oncogene	Met	1.74	0.006
1452006_x_at	archease	MGI:1914356	1.74	0.007
1425064_at	aryl hydrocarbon receptor nuclear translocator	Arnt	1.74	0.001
1439467_at	Microtubule-associated protein 4	Mtap4	1.73	0.008
1439251_at	Iduronidase, alpha-L-	Idua	1.73	0.004
1455955_s_at	sorting nexin 17	Snx17	1.73	0.004
1425996_a_at	SWI/SNF related, matrix associated, actin dependent regulator of chromatin, subfamily a, member 3	Smarca3	1.73	0.005
1429617_at	cylindromatosis (turban tumor syndrome)	Cyld	1.73	0.003
1417238_at	Ewing sarcoma breakpoint region 1	Ewsr1	1.73	0.004

1419444_at	Sin3-associated polypeptide 18	Sap18	1.72	0.001
1448309_at	adaptor-related protein complex 3, mu 1 subunit	Ap3m1	1.72	0.003
1453914_at	heat shock protein 1	Hspb1	1.72	0.008
1417315_at	GRIP1 associated protein 1	Gripap1	1.72	0.005
1452430_s_at	splicing factor, arginine/serine-rich 1 (ASF/SF2)	Sfrs1	1.72	0.001
1423028_at	interferon alpha family, gene 2	Ifna2	1.72	0.008
1457306_at	Polymerase (DNA directed), epsilon 3 (p17 subunit)	Pole3	1.71	0.009
1417963_at	phospholipid transfer protein	Pltp	1.71	0.009
1424918_at	TBC1 domain family, member 19	Tbc1d19	1.71	0.005
1436454_x_at	flap structure specific endonuclease 1	Fen1	1.71	0.007
1437591_a_at	WD repeat domain 1	Wdr1	1.71	0.005
1431032_at	amylo-1,6-glucosidase, 4-alpha-glucanotransferase	Agl	1.71	0.005
1416529_at	epithelial membrane protein 1	Emp1	1.71	0.003
1456080_a_at	Tumor differentially expressed 1	Tde1	1.71	0.003
1427385_s_at	actinin, alpha 1	Actn1	1.71	0.002
1455639_at	Rap2 interacting protein	Rap2ip	1.71	0.000
1424578_at	arrestin domain containing 1	Arrdc1	1.71	0.002
1452178_at	plectin 1	Plec1	1.70	0.004
1448276_at	tetraspanin 4	Tspan4	1.70	0.000
1430731_at	keratin associated protein 2-4	Krtap2-4	1.70	0.006
1454997_at	Methionine sulfoxide reductase B3	Msrb3	1.69	0.001
1420874_at	protein tyrosine kinase 9	Ptk9	1.69	0.003
1448328_at	SH3-domain binding protein 2	Sh3bp2	1.69	0.001
1427776_a_at	fibroblast growth factor receptor 4	Fgfr4	1.69	0.000
1417527_at	adaptor-related protein complex 3, mu 2 subunit	Ap3m2	1.69	0.010
1420483_at	cyclin M3	Cnnm3	1.69	0.004
1416994_at	tetratricopeptide repeat domain 1	Ttc1	1.69	0.005
1419167_at	proline-rich acidic protein 1	Prap1	1.69	0.007
1426061_x_at	Cullin 4A	Cul4a	1.68	0.010
1450399_at	presenilin 1	Psen1	1.68	0.002
1416933_at	P450 (cytochrome) oxidoreductase	Por	1.66	0.002
1455547_at	scrapie responsive gene 3	Scrg3	1.66	0.007
1449486_at	carboxylesterase 1	Ces1	1.66	0.008
1422439_a_at	cyclin-dependent kinase 4	Cdk4	1.66	0.003
1451938_a_at	syntrophin, basic 1	Sntb1	1.66	0.007
1424307_at	Rho GTPase activating protein 1	Arhgap1	1.65	0.010

1450420_at	stromal antigen 1	Stag1	1.65	0.001
1416523_at	ribonuclease, RNase A family, 1 (pancreatic)	Rnase1	1.65	0.001
1430274_a_at	STARD3 N-terminal like	Stard3nl	1.65	0.008
1420472_at	myotrophin	Mtpn	1.65	0.000
1422676_at	SWI/SNF related, matrix associated, actin dependent regulator of chromatin, subfamily e, member 1	Smarce1	1.64	0.001
1454704_at	scavenger receptor class B, member 2	Scarb2	1.64	0.000
1423356_at	synaptosomal-associated protein	Snap29	1.64	0.001
1419266_at	nuclear transcription factor-Y beta	Nfyb	1.64	0.008
1460169_a_at	PCTAIRE-motif protein kinase 1	Pctk1	1.64	0.005
1453751_at	DEAH (Asp-Glu-Ala-His) box polypeptide 38	Dhx38	1.63	0.010
1421188_at	chemokine (C-C motif) receptor 2	Ccr2	1.63	0.007
1421476_a_at	calcium activated nucleotidase 1	Cant1	1.63	0.003
1451072_a_at	ring finger protein 4	Rnf4	1.63	0.004
1451104_a_at	U1 small nuclear ribonucleoprotein polypeptide A	Snrp70	1.62	0.006
1420943_at	zinc finger protein 185	Zfp185	1.61	0.006
1451749_at	interleukin-1 receptor-associated kinase 4	Irak4	1.61	0.006
1426379_at	eukaryotic translation initiation factor 4B	Eif4b	1.61	0.000
1437453_s_at	proprotein convertase subtilisin/kexin type 9	Pcsk9	1.61	0.007
1460376_a_at	COX15 homolog, cytochrome c oxidase assembly protein (yeast)	Cox15	1.61	0.004
1424291_at	nucleoporin 93	Nup93	1.61	0.004
1419195_at	WAP four-disulfide core domain 15	Wfdc15	1.61	0.003
1449988_at	GTPase, IMAP family member 1	Gimap1	1.60	0.003
1429911_at	microcephaly, primary autosomal recessive 1	Mcph1	1.60	0.002
1449925_at	chemokine (C-X-C motif) receptor 3	Cxcr3	1.60	0.004
1423946_at	PDZ and LIM domain 2	Pdlim2	1.60	0.009
1441477_at	calumenin	Calu	1.59	0.009
1448732_at	cathepsin B	Ctsb	1.59	0.001
1449039_a_at	heterogeneous nuclear ribonucleoprotein D-like	Hnrpdl	1.59	0.007
1427183_at	epidermal growth factor-containing fibulin-like extracellular matrix protein 1	Efemp1	1.59	0.001
1424926_at	SEC63-like (S. cerevisiae)	Sec63	1.59	0.009
1424595_at	F11 receptor	F11r	1.58	0.001
1452036_a_at	thymopoietin	Tmpo	1.58	0.002
1422959_s_at	zinc finger protein 313	Zfp313	1.58	0.000
1437910_at	transmembrane protein 39b	Tmem39b	1.58	0.004
1456890_at	DEAD (Asp-Glu-Ala-Asp) box polypeptide 58	Ddx58	1.58	0.007
1449404_at	phosphatidylinositol-4-phosphate 5-kinase, type II, alpha	Pip5k2a	1.57	0.010

1449530_at	trichorhinophalangeal syndrome I (human)	Trps1	1.57	0.005
1453857_at	ring finger protein 180	Rnf180	1.57	0.001
1422389_at	taste receptor, type 2, member 105	Tas2r105	1.56	0.001
1460650_at	ATPase, H+ transporting, lysosomal V0 subunit a isoform 1	Atp6v0a1	1.55	0.008
1423061_at	armadillo repeat gene deleted in velo-cardio-facial syndrome	Arvcf	1.55	0.005
1453960_a_at	capping protein (actin filament) muscle Z-line, beta	Capzb	1.54	0.004
1416525_at	speckle-type POZ protein	Spop	1.54	0.000
1429830_a_at	CD59a antigen	Cd59a	1.54	0.000
1420612_s_at	protein tyrosine phosphatase 4a2	Ptp4a2	1.54	0.001
1420638_at	phosphoribosyl pyrophosphate synthetase 2	Prps2	1.53	0.006
1417631_at	MAP kinase-interacting serine/threonine kinase 1	Mknk1	1.53	0.008
1440904_at	SUMO/sentrin specific protease 5	Senp5	1.53	0.008
1450464_at	E4F transcription factor 1	E4f1	1.53	0.000
1433548_at	alpha globin regulatory element containing gene	Mare	1.53	0.001
1419397_at	polymerase (DNA directed), alpha 1	Pola1	1.53	0.001
1426645_at	heat shock protein 1, alpha	Hspca	1.53	0.003
1422946_a_at	DNA methyltransferase (cytosine-5) 1	Dnmt1	1.53	0.006
1422117_s_at	KH domain containing, RNA binding, signal transduction associated 2	Khdrbs2	1.52	0.005
1435518_at	RAS related protein 1b	Rap1b	1.52	0.002
1450262_at	cardiotrophin-like cytokine factor 1	Clcf1	1.52	0.004
1427718_a_at	transformed mouse 3T3 cell double minute 2	Mdm2	1.52	0.009
1427578_a_at	integrin beta 4 binding protein	Itgb4bp	1.52	0.000
1420866_at	zinc finger protein 161	Zfp161	1.52	0.006
1449503_at	karyopherin (importin) alpha 1	Kpna1	1.51	0.002
1460373_a_at	open reading frame 21	ORF21	1.51	0.002
1420879_a_at	tyrosine 3-monooxygenase/tryptophan 5-monooxygenase activation protein, beta polypeptide	Ywhab	1.51	0.009
1448580_at	golgi apparatus protein 1	Glg1	1.51	0.002
1456482_at	phosphatidylinositol 3 kinase, regulatory subunit, polypeptide 3 (p55)	Pik3r3	1.51	0.006
1426790_at	structure specific recognition protein 1	Ssrp1	1.50	0.001
1455640_a_at	thioredoxin 2	Txn2	1.50	0.000
1416435_at	lymphotoxin B receptor	Ltbr	1.50	0.000
1438498_at	chemokine (C-X-C motif) ligand 16	Cxcl16	1.50	0.007
1451841_a_at	nuclear receptor co-repressor 2	Ncor2	1.50	0.004
1416162_at	RAD21 homolog (S. pombe)	Rad21	1.49	0.003
1452527_a_at	purinergic receptor P2X, ligand-gated ion channel 4	P2rx4	1.49	0.005
1448868_at	SCAN domain-containing 1	Scand1	1.49	0.002

1437938_x_at	dynamin 2	Dnm2	1.49	0.007
1434185_at	acetyl-Coenzyme A carboxylase alpha	Acaca	1.48	0.004
1423430_at	MYB binding protein (P160) 1a	Mybbp1a	1.48	0.006
1434753_at	nuclear factor related to kappa B binding protein	Nfrkb	1.48	0.006
1436197_at	CDC42 binding protein kinase gamma (DMPK-like)	Cdc42bpg	1.48	0.007
1428158_at	AKT1 substrate 1 (proline-rich)	Akt1s1	1.48	0.003
1424484_at	MOB1, Mps One Binder kinase activator-like 1B (yeast)	Mobk1b	1.48	0.007
1451397_at	trinucleotide repeat containing 15	Tnrc15	1.47	0.003
1416618_at	protoporphyrinogen oxidase	Ppox	1.47	0.006
1431704_a_at	Ral GEF with PH domain and SH3 binding motif 2	Ralgps2	1.47	0.008
1423770_at	transmembrane channel-like gene family 6	Tmc6	1.47	0.008
1424337_at	sorting nexin 15	Snx15	1.47	0.007
1450006_at	nuclear receptor coactivator 4	Ncoa4	1.46	0.005
1423746_at	thioredoxin domain containing 5	Txndc5	1.46	0.007
1426898_at	mitogen-activated protein kinase kinase kinase 7 interacting protein 1	Map3k7ip1	1.46	0.009
1416611_at	secretory carrier membrane protein 2	Scamp2	1.46	0.001
1449862_a_at	phosphatidylinositol 4-kinase type 2 beta	Pi4k2b	1.45	0.003
1417350_at	pallidin	Pldn	1.45	0.006
1434776_at	semaphorin 5A	Sema5a	1.44	0.005
1456037_x_at	prolactin regulatory element binding	Preb	1.44	0.008
1435988_x_at	IK cytokine	Ik	1.44	0.009
1451969_s_at	poly (ADP-ribose) polymerase family, member 3	Parp3	1.43	0.001
1446048_at	Cadherin 11	Cdh11	1.43	0.006
1421501_a_at	Rab6 interacting protein 2	Rab6ip2	1.43	0.007
1436048_at	exocyst complex component 8	Exoc8	1.43	0.001
1435349_at	Neuropilin 2	Nrp2	1.43	0.006
1452435_at	WD repeat domain 22	Wdr22	1.43	0.004
1417574_at	chemokine (C-X-C motif) ligand 12	Cxcl12	1.43	0.001
1437478_s_at	EF hand domain containing 2	Efhd2	1.42	0.008
1426998_at	testis expressed gene 27	Tex27	1.42	0.000
1449297_at	caspase 12	Casp12	1.42	0.009
1425605_a_at	limb region 1	Lmbr1	1.42	0.003
1451066_at	leukocyte receptor cluster (LRC) member 4	Leng4	1.42	0.009
1424103_at	APG4 (ATG4) autophagy-related homolog B (S. cerevisiae)	Apg4b	1.42	0.008
1422886_a_at	CDC like kinase 4	Clk4	1.41	0.006
1444141_at	sorting nexin 13	Snx13	1.41	0.007

1425719_a_at	N-myc (and STAT) interactor	Nmi	1.40	0.004
1436554_at	zinc finger protein, subfamily 1A, 5	Zfpn1a5	1.40	0.009
1430857_s_at	peroxisomal biogenesis factor 11c	Pex11c	1.40	0.004
1435444_at	activating transcription factor 6	Atf6	1.39	0.008
1424601_at	X-ray repair complementing defective repair in Chinese hamster cells 4	Xrcc4	1.38	0.009
1443819_x_at	tetratricopeptide repeat domain 11	Ttc11	1.38	0.001
1423643_at	DEAD (Asp-Glu-Ala-Asp) box polypeptide 39	Ddx39	1.37	0.004
1449322_at	protein tyrosine phosphatase 4a1	Ptp4a1	1.36	0.002
1451726_at	myotubularin related protein 6	Mtmr6	1.36	0.003
1418232_s_at	LIM and senescent cell antigen-like domains 1	Lims1	1.35	0.006
1416386_a_at	mannose-6-phosphate receptor, cation dependent	M6pr	1.35	0.000
1423230_at	inositol polyphosphate-5-phosphatase E	Inpp5e	1.35	0.010
1422638_s_at	Ras association (RalGDS/AF-6) domain family 5	Rassf5	1.35	0.002
1426739_at	downstream neighbor of SON	Donson	1.34	0.005
1425497_a_at	PRP4 pre-mRNA processing factor 4 homolog B (yeast)	Prpf4b	1.33	0.001
1423652_at	HESB like domain containing 2	Hbld2	1.33	0.009
1421828_at	karyopherin (importin) alpha 3	Kpna3	1.33	0.002
1435476_a_at	Fc receptor, IgG, low affinity IIb	Fcgr2b	1.33	0.004
1452545_a_at	integrin beta 1 (fibronectin receptor beta)	Itgb1	1.33	0.005
1423428_at	receptor tyrosine kinase-like orphan receptor 2	Ror2	1.32	0.007
1423481_at	RIO kinase 2 (yeast)	Riok2	1.32	0.002
1424161_at	DEAD (Asp-Glu-Ala-Asp) box polypeptide 27	Ddx27	1.32	0.008
1460279_a_at	general transcription factor II I	Gtf2i	1.32	0.002
1423282_at	phosphatidylinositol transfer protein, alpha	Pitpna	1.31	0.001
1415748_a_at	dynactin 5	Dctn5	1.31	0.003
1422792_at	platelet-activating factor acetylhydrolase, isoform 1b, alpha2 subunit	Pafah1b2	1.30	0.007
1450722_at	nucleoporin 50	Nup50	1.29	0.007
1454996_at	hydroxysteroid dehydrogenase like 1	Hsdl1	1.29	0.003
1429206_at	Rho-related BTB domain containing 1	Rhobtb1	1.29	0.001
1429787_x_at	ZW10 interactor	Zwint	1.29	0.007
1428315_at	EBNA1 binding protein 2	Ebna1bp2	1.27	0.002
1423264_at	block of proliferation 1	Bop1	1.26	0.001
1452238_at	HIV-1 Rev binding protein	Hrb	1.25	0.008
1459884_at	cytochrome c oxidase, subunit VIIc	Cox7c	1.25	0.004
1450963_at	heterogeneous nuclear ribonucleoprotein F	Hnrpf	1.24	0.008
1422764_at	microtubule-associated protein, RP/EB family, member 1	Mapre1	1.24	0.002

1448133_at	NMD3 homolog (S. cerevisiae)	Nmd3	1.23	0.010
1422547_at	RAN binding protein 1	Ranbp1	1.23	0.009
1448647_at	mannosidase 2, alpha 1	Man2a1	1.22	0.002
1425977_a_at	STE20-like kinase (yeast)	Slk	1.21	0.000
1426450_at	phospholipase C-like 2	Plcl2	1.21	0.006
1456769_at	dual specificity phosphatase 3 (vaccinia virus phosphatase VH1-related)	Dusp3	1.20	0.005
1434250_at	p21 (CDKN1A)-activated kinase 2	Pak2	1.19	0.008
1436573_at	Secernin 3	Scrn3	1.19	0.005
1424740_at	cAMP responsive element binding protein 3	Creb3	1.19	0.010
1448159_at	RAB7, member RAS oncogene family	Rab7	1.18	0.007
1417279_at	inositol 1,4,5-triphosphate receptor 1	Itpr1	1.17	0.005
1451005_at	SMT3 suppressor of mif two 3 homolog 1 (yeast)	Sumo1	1.12	0.002
1425336_x_at	histocompatibility 2, K1, K region	H2-K1	1.11	0.009
1438839_a_at	tyrosine 3-monooxygenase/tryptophan 5-monooxygenase activation protein, epsilon polypeptide	Ywhae	-1.10	0.007
1434489_at	engulfment and cell motility 3, ced-12 homolog (C. elegans)	Elmo3	-1.11	0.007
1423113_a_at	ubiquitin-conjugating enzyme E2D 3 (UBC4/5 homolog, yeast)	Ube2d3	-1.11	0.007
1434112_at	latrophilin 2	Lphn2	-1.14	0.008
1425591_a_at	chromatin modifying protein 2A	Chmp2a	-1.15	0.004
1448206_at	proteasome (prosome, macropain) subunit, alpha type 2	Psma2	-1.15	0.008
1429077_x_at	ribosomal protein L21	Rpl21	-1.15	0.004
1426162_a_at	ribosomal protein L7	Rpl7	-1.15	0.002
1417715_a_at	glutamate oxaloacetate transaminase 2, mitochondrial	Got2	-1.15	0.006
1448387_at	ring-box 1	Rbx1	-1.15	0.003
1417086_at	platelet-activating factor acetylhydrolase, isoform 1b, beta1 subunit	Pafah1b1	-1.16	0.009
1449196_a_at	ribosomal protein S27a	Rps27a	-1.16	0.002
1455168_a_at	guanine nucleotide binding protein, beta 2, related sequence 1	Gnb2-rs1	-1.16	0.002
1417398_at	related RAS viral (r-ras) oncogene homolog 2	Rras2	-1.17	0.010
1416037_a_at	chaperonin subunit 2 (beta)	Cct2	-1.17	0.001
1420745_a_at	cyclin D-type binding-protein 1	Ccndbp1	-1.17	0.006
1418708_at	apolipoprotein C-IV	Apoc4	-1.17	0.007
1444138_at	cytochrome P450, family 2, subfamily r, polypeptide 1	Cyp2r1	-1.17	0.004
1417451_a_at	peptidylprolyl isomerase A	Ppia	-1.17	0.005
1434127_a_at	H3 histone, family 3A	H3f3a	-1.17	0.005
1449255_a_at	ribosomal protein L15	Rpl15	-1.17	0.006
1454661_at	ATP synthase, H+ transporting, mitochondrial F0 complex, subunit c (subunit 9), isoform 3	Atp5g3	-1.17	0.000
1417533_a_at	integrin beta 5	Itgb5	-1.17	0.005

1415882_at	growth hormone inducible transmembrane protein	Ghitm	-1.18	0.005
1450964_a_at	oxysterol binding protein-like 9	Osbpl9	-1.18	0.002
1418101_a_at	reticulon 3	Rtn3	-1.18	0.008
1429914_at	Enhancer of polycomb homolog 1 (Drosophila)	Epc1	-1.18	0.005
1416742_at	craniofacial development protein 1	Cfdp1	-1.18	0.002
1421743_a_at	poly(rC) binding protein 2	Pcbp2	-1.18	0.004
1434801_x_at	solute carrier family 25 (mitochondrial carrier, adenine nucleotide translocator), member 5	Slc25a5	-1.18	0.000
1451120_at	RNA polymerase 1-3	Rpo1-3	-1.18	0.005
1418119_at	RNA binding motif protein 8a	Rbm8a	-1.18	0.008
1418568_x_at	signal recognition particle 14	Srp14	-1.19	0.001
1432099_a_at	proline dehydrogenase (oxidase) 2	Prodh2	-1.19	0.006
1450778_a_at	phosphorylated adaptor for RNA export	MGI:1891839	-1.19	0.008
1428212_x_at	ribosomal protein L31	Rpl31	-1.19	0.001
1437413_x_at	ribosomal protein S29	Rps29	-1.19	0.008
1418888_a_at	selenoprotein X 1	Sepx1	-1.19	0.003
1448585_at	general transcription factor II H, polypeptide 4	Gtf2h4	-1.19	0.001
1424139_at	RAS-related protein-1a	Rap1a	-1.19	0.009
1452659_at	DEK oncogene (DNA binding)	Dek	-1.19	0.005
1452168_x_at	G1 to S phase transition 1	Gspt1	-1.19	0.000
1416291_at	proteasome (prosome, macropain) 26S subunit, ATPase, 4	Psmc4	-1.20	0.000
1416170_at	TNF receptor-associated protein 1	Trap1	-1.20	0.002
1416570_s_at	G elongation factor 1	Gfm1	-1.20	0.009
1428152_a_at	Ribosomal protein L18A	Rpl18a	-1.20	0.007
1451149_at	phosphoglucomutase 2	Pgm2	-1.20	0.000
1417608_a_at	ribosomal protein L13a	Rpl13a	-1.20	0.002
1450815_s_at	coiled-coil-helix-coiled-coil-helix domain containing 2 /// RIKEN cDNA 2410018M08 gene	Chchd2 /// 241001ε	-1.20	0.000
1451470_s_at	eukaryotic translation initiation factor 5A	Eif5a	-1.21	0.000
1433951_at	ADP-ribosylation factor-like 5	Arl5	-1.21	0.001
1417762_a_at	ribosomal protein L8	Rpl8	-1.21	0.006
1422613_a_at	ribosomal protein L7a	Rpl7a	-1.21	0.001
1422469_at	TANK-binding kinase 1	Tbk1	-1.21	0.005
1428845_at	BCL2-associated transcription factor 1	Bclaf1	-1.21	0.004
1460671_at	glutathione peroxidase 1	Gpx1	-1.22	0.007
1435362_at	forkhead box J3	Foxj3	-1.22	0.009
1416867_at	blocked early in transport 1 homolog (S. cerevisiae)	Bet1	-1.22	0.001
1456213_x_at	glutaminyl-tRNA synthetase	Qars	-1.22	0.007

1432080_s_at	MYST histone acetyltransferase 1	Myst1	-1.22	0.003
1429425_at	ring finger protein 139	Rnf139	-1.22	0.001
1415723_at	eukaryotic translation initiation factor 5	Eif5	-1.22	0.001
1426367_at	calcium binding protein 39-like	Cab39l	-1.22	0.000
1417111_at	mannosidase 1, alpha	Man1a	-1.23	0.000
1416904_at	muscleblind-like 1 (Drosophila)	Mbnl1	-1.23	0.001
1426836_s_at	methionyl aminopeptidase 1	Metap1	-1.23	0.009
1437401_at	Insulin-like growth factor 1	Igf1	-1.23	0.010
1434305_at	U2 small nuclear RNA auxiliary factor 1-like 4	U2af1l4	-1.23	0.009
1454778_x_at	Ribosomal protein S28	Rps28	-1.23	0.004
1416197_at	U1 small nuclear ribonucleoprotein 1C	Snrp1c	-1.23	0.005
1417124_at	destrin	Dstn	-1.23	0.004
1429131_at	ubiquitin-conjugating enzyme E2 variant 2	Ube2v2	-1.23	0.007
1420846_at	mitochondrial ribosomal protein S2	Mrps2	-1.23	0.001
1427955_a_at	differentially expressed in B16F10 1	Deb1	-1.23	0.004
1417085_at	aldo-keto reductase family 1, member C6	Akr1c6	-1.23	0.000
1423187_at	gamma-aminobutyric acid (GABA-A) receptor-associated protein-like 2	Gabarapl2	-1.23	0.006
1450149_a_at	protein phosphatase 1, catalytic subunit, gamma isoform	Ppp1cc	-1.23	0.003
1452701_x_at	ubiquitin A-52 residue ribosomal protein fusion product 1	Uba52	-1.24	0.000
1415970_at	cytochrome c oxidase, subunit VIc	Cox6c	-1.24	0.003
1448653_at	embryonic ectoderm development	Eed	-1.24	0.007
1448717_at	glutaryl-Coenzyme A dehydrogenase	Gcdh	-1.24	0.008
1428012_at	complement component 8, alpha polypeptide	C8a	-1.24	0.008
1427923_at	zinc metalloproteinase, STE24 homolog (S. cerevisiae)	Zmpste24	-1.24	0.005
1428029_a_at	H2A histone family, member V	H2afv	-1.24	0.007
1417157_at	ARP10 actin-related protein 10 homolog (S. cerevisiae)	Actr10	-1.24	0.001
1455024_at	tousled-like kinase 1	Tlk1	-1.24	0.001
1428615_at	purinergic receptor (family A group 5)	P2y5	-1.24	0.003
1431177_a_at	ribosomal protein L10A	Rpl10a	-1.24	0.000
1427901_at	mitochondrial ribosomal protein S18C	Mrps18c	-1.24	0.004
1419499_at	Glycerol-3-phosphate acyltransferase, mitochondrial	Gpam	-1.24	0.010
1423399_a_at	YY1 associated factor 2	Yaf2	-1.24	0.003
1450372_a_at	ribosomal protein L18	Rpl18	-1.24	0.006
1437510_x_at	ribosomal protein S17	Rps17	-1.25	0.001
1419131_at	coagulation factor XIII, beta subunit	F13b	-1.25	0.006
1418845_at	protein C	Proc	-1.25	0.001

1415867_at	chaperonin subunit 4 (delta)	Cct4	-1.25	0.002
1452211_at	proteasome (prosome, macropain) activator subunit 4	Psme4	-1.25	0.007
1428716_at	peroxisome biogenesis factor 1	Pex1	-1.25	0.005
1448677_at	neighbor of Cox4	Noc4	-1.25	0.000
1427442_a_at	amyloid beta (A4) precursor protein	App	-1.25	0.005
1417242_at	DEAD (Asp-Glu-Ala-Asp) box polypeptide 48	Ddx48	-1.25	0.001
1438179_s_at	Signal transducer and activator of transcription interacting protein 1	Statip1	-1.25	0.003
1437993_x_at	quininoid dihydropteridine reductase	Qdpr	-1.25	0.001
1436586_x_at	ribosomal protein S14	Rps14	-1.26	0.000
1418583_at	histidine triad nucleotide binding protein 3	Hint3	-1.26	0.001
1422794_at	cullin 3	Cul3	-1.26	0.000
1448739_x_at	ribosomal protein S18	Rps18	-1.26	0.005
1416247_at	dynactin 3	Dctn3	-1.26	0.000
1418029_at	Fas apoptotic inhibitory molecule	Faim	-1.26	0.006
1448503_at	myeloid cell leukemia sequence 1	Mcl1	-1.26	0.002
1448493_at	polyadenylate-binding protein-interacting protein 2	Paip2	-1.26	0.009
1448166_a_at	proteasome (prosome, macropain) subunit, beta type 1	Psmb1	-1.26	0.008
1448418_s_at	WD repeat domain 23	Wdr23	-1.26	0.000
1416556_at	sarcoma amplified sequence	Sas	-1.26	0.000
1415879_a_at	ribosomal protein, large P2	Rplp2	-1.26	0.007
1423236_at	UDP-N-acetyl-alpha-D-galactosamine:polypeptide N-acetylgalactosaminyltransferase 1	Galnt1	-1.26	0.003
1424124_at	motile sperm domain containing 2	Mospd2	-1.26	0.010
1450694_at	FK506 binding protein 2	Fkbp2	-1.26	0.000
1449706_s_at	nuclear receptor subfamily 5, group A, member 2	Nr5a2	-1.26	0.004
1416277_a_at	ribosomal protein, large, P1	Rplp1	-1.26	0.001
1417919_at	protein phosphatase 1, regulatory (inhibitor) subunit 7	Ppp1r7	-1.26	0.004
1417046_at	tumor suppressor candidate 4	Tusc4	-1.27	0.001
1415841_at	dynein, cytoplasmic, intermediate chain 2	Dncic2	-1.27	0.002
1418292_at	arsA (bacterial) arsenite transporter, ATP-binding, homolog 1	Asna1	-1.27	0.006
1460216_at	acyl-Coenzyme A dehydrogenase, short chain	Acads	-1.27	0.008
1455319_x_at	ribosomal protein S8	Rps8	-1.27	0.010
1438626_x_at	ribosomal protein L14	Rpl14	-1.27	0.007
1416508_at	mediator of RNA polymerase II transcription, subunit 28 homolog (yeast)	Med28	-1.27	0.000
1423898_a_at	thyroid hormone receptor interactor 12	Trip12	-1.27	0.006
1437014_x_at	Peroxiredoxin 1	Prdx1	-1.27	0.006
1448630_a_at	succinate dehydrogenase complex, subunit C, integral membrane protein	Sdhc	-1.27	0.009

1447277_s_at	prenylcysteine oxidase 1	Pcyox1	-1.27	0.003
1451162_at	heat shock factor binding protein 1	Hsbp1	-1.27	0.001
1417749_a_at	tight junction protein 1	Tjp1	-1.27	0.001
1436994_a_at	histone 1, H1c	Hist1h1c	-1.27	0.009
1433472_x_at	ribosomal protein L38	Rpl38	-1.27	0.001
1448361_at	tetratricopeptide repeat domain 3	Ttc3	-1.27	0.003
1418229_s_at	histone cell cycle regulation defective interacting protein 5	Hirip5	-1.27	0.004
1438510_a_at	histidyl-tRNA synthetase	Hars	-1.27	0.010
1415930_a_at	microtubule-associated protein 1 light chain 3 beta	Map1lc3b	-1.28	0.006
1426590_at	G elongation factor, mitochondrial 2	Gfm2	-1.28	0.002
1424189_at	phosphatidylinositol glycan, classC	Pigc	-1.28	0.002
1456227_x_at	retinoblastoma binding protein 7	Rbbp7	-1.28	0.009
1416142_at	ribosomal protein S6	Rps6	-1.28	0.008
1424184_at	acyl-Coenzyme A dehydrogenase, very long chain	Acadvl	-1.28	0.004
1416948_at	mitochondrial ribosomal protein L23	Mrpl23	-1.28	0.004
1418713_at	pterin 4 alpha carbinolamine dehydratase/dimerization cofactor of hepatocyte nuclear factor 1 alpha (TC	Pcbd1	-1.28	0.006
1426296_at	RAD52 homolog (S. cerevisiae)	Rad52	-1.28	0.002
1456628_x_at	ribosomal protein S24	Rps24	-1.28	0.002
1448822_at	proteasome (prosome, macropain) subunit, beta type 6	Psmb6	-1.29	0.002
1423069_at	activity-dependent neuroprotective protein	Adnp	-1.29	0.000
1425036_a_at	trinucleotide repeat containing 6a	Tnrc6a	-1.29	0.004
1418821_at	cytochrome P450, family 2, subfamily a, polypeptide 12	Cyp2a12	-1.29	0.004
1456867_x_at	serologically defined breast cancer antigen 84	Sdbcag84	-1.29	0.002
1416706_at	ribulose-5-phosphate-3-epimerase	Rpe	-1.29	0.001
1460644_at	branched chain ketoacid dehydrogenase kinase	Bckdk	-1.29	0.004
1416175_a_at	voltage-dependent anion channel 3	Vdac3	-1.29	0.000
1448673_at	poliovirus receptor-related 3	Pvrl3	-1.29	0.000
1428448_a_at	general transcription factor IIIC, polypeptide 2, beta	Gtf3c2	-1.29	0.010
1455558_at	gene model 114, (NCBI)	Gm114	-1.29	0.001
1416243_a_at	ribosomal protein L35	Rpl35	-1.29	0.007
1448508_at	Traf3 interacting protein 2	Traf3ip2	-1.29	0.007
1433782_at	claudin 12	Cldn12	-1.29	0.004
1454859_a_at	ribosomal protein L23	Rpl23	-1.29	0.006
1422576_at	spinocerebellar ataxia 10 homolog (human)	Sca10	-1.30	0.006
1448767_s_at	gap junction membrane channel protein beta 1	Gjb1	-1.30	0.004
1452674_a_at	eukaryotic translation initiation factor 3, subunit 12	Eif3s12	-1.30	0.003

1415980_at	ATP synthase, H+ transporting, mitochondrial F0 complex, subunit c (subunit 9), isoform 2	Atp5g2	-1.30	0.001
1416315_at	abhydrolase domain containing 4	Abhd4	-1.30	0.001
1426554_a_at	phosphoglycerate mutase 1	Pgam1	-1.30	0.002
1434578_x_at	RAN, member RAS oncogene family	Ran	-1.30	0.004
1424366_at	transmembrane protein 15	Tmem15	-1.30	0.010
1438890_at	F-box and leucine-rich repeat protein 11	Fbxl11	-1.30	0.007
1435046_at	Myosin ID	Myo1d	-1.30	0.002
1449935_a_at	DnaJ (Hsp40) homolog, subfamily A, member 3	Dnaja3	-1.30	0.004
1432264_x_at	cytochrome c oxidase subunit VIIa polypeptide 2-like	Cox7a2l	-1.30	0.010
1426661_at	ribosomal protein L27a	Rpl27a	-1.30	0.002
1429085_at	vascular endothelial zinc finger 1	Vezf1	-1.30	0.003
1417505_s_at	interleukin 11 receptor, alpha chain 1 /// interleukin 11 receptor, alpha chain 2	Il11ra1 /// Il11ra2	-1.30	0.004
1426912_at	ring finger and WD repeat domain 2	Rfwd2	-1.30	0.000
1439268_x_at	eukaryotic translation initiation factor 3, subunit 6	Eif3s6	-1.30	0.003
1452627_at	SUMO/sentrin specific protease 6	Senp6	-1.30	0.000
1439057_x_at	zinc finger, DHHC domain containing 6	Zdhhc6	-1.30	0.000
1430849_a_at	cell growth regulator with ring finger domain 1	Cgrrf1	-1.30	0.007
1450866_a_at	mitochondrial ribosomal protein L17	Mrpl17	-1.30	0.001
1453260_a_at	protein phosphatase 2 (formerly 2A), regulatory subunit B (PR 52), alpha isoform	Ppp2r2a	-1.30	0.003
1416789_at	isocitrate dehydrogenase 3 (NAD+), gamma	Idh3g	-1.31	0.003
1429681_a_at	glycoprotein, synaptic 2	Gpsn2	-1.31	0.003
1434814_x_at	glucose phosphate isomerase 1	Gpi1	-1.31	0.000
1459832_s_at	adaptor-related protein complex AP-1, mu subunit 1	Ap1m1	-1.31	0.006
1460244_at	ureidopropionase, beta	Upb1	-1.31	0.001
1458385_at	heat shock 70kDa protein 4 like	Hspa4l	-1.31	0.010
1437783_x_at	transmembrane protein 4	Tmem4	-1.31	0.001
1427404_x_at	enolase 1, alpha non-neuron	Eno1	-1.31	0.005
1420712_a_at	hepsin	Hpn	-1.31	0.000
1426536_at	NMDA receptor-regulated gene 2	Narg2	-1.31	0.001
1435011_x_at	aldo-keto reductase family 1, member A4 (aldehyde reductase)	Akr1a4	-1.31	0.002
1448922_at	dual specificity phosphatase 19	Dusp19	-1.31	0.006
1428326_s_at	heat-responsive protein 12	Hrsp12	-1.31	0.008
1435750_at	GTP cyclohydrolase I feedback regulator	Gchfr	-1.31	0.000
1416168_at	serine (or cysteine) proteinase inhibitor, clade F, member 1	Serpinf1	-1.31	0.008
1416510_at	mitochondrial ribosomal protein L4	Mrpl4	-1.31	0.001
1423911_at	protein phosphatase 2, regulatory subunit B (B56), alpha isoform	Ppp2r5a	-1.31	0.001

1416532_at	transformation/transcription domain-associated protein	Trrap	-1.31	0.001
1450872_s_at	lysosomal acid lipase 1	Lip1	-1.31	0.002
1455391_at	RAD23a homolog (S. cerevisiae)	Rad23a	-1.31	0.006
1455348_x_at	ribosomal protein L29	Rpl29	-1.31	0.001
1437533_at	Baculoviral IAP repeat-containing 4	Birc4	-1.31	0.008
1423223_a_at	peroxiredoxin 6	Prdx6	-1.31	0.006
1435347_at	staufen (RNA binding protein) homolog 1 (Drosophila)	Stau1	-1.31	0.000
1426762_s_at	amine oxidase (flavin containing) domain 2	Aof2	-1.32	0.001
1422858_at	Thyroid hormone receptor interactor 4	Trip4	-1.32	0.006
1421751_a_at	proteasome (prosome, macropain) 26S subunit, non-ATPase, 14	Psmd14	-1.32	0.005
1448252_a_at	eukaryotic translation elongation factor 1 beta 2	Eef1b2	-1.32	0.000
1416005_at	protease (prosome, macropain) 26S subunit, ATPase 1	Psmc1	-1.32	0.000
1416506_at	proteasome (prosome, macropain) subunit, alpha type 6	Psma6	-1.32	0.001
1436689_a_at	aldehyde dehydrogenase 9, subfamily A1	Aldh9a1	-1.32	0.005
1423717_at	adenylate kinase 3	Ak3	-1.32	0.002
1448709_at	AT rich interactive domain 1A (Swi1 like)	Arid1a	-1.32	0.007
1455951_at	methionine-tRNA synthetase	Mars	-1.32	0.006
1426457_at	sarcolemma associated protein	Slmap	-1.32	0.001
1448438_at	Der1-like domain family, member 2	Derl2	-1.32	0.001
1455047_at	F-box only protein 3	Fbxo3	-1.32	0.002
1454973_at	activating transcription factor 7 interacting protein	Atf7ip	-1.32	0.005
1448846_a_at	ribosomal protein L29	Rpl29	-1.32	0.001
1416938_at	coiled-coil-helix-coiled-coil-helix domain containing 1	Chchd1	-1.32	0.000
1417176_at	casein kinase 1, epsilon	Csnk1e	-1.32	0.001
1447917_x_at	N-terminal Asn amidase	Ntan1	-1.32	0.000
1415688_at	ubiquitin-conjugating enzyme E2G 1	Ube2g1	-1.32	0.008
1448307_at	Down syndrome critical region homolog 2 (human)	Dscr2	-1.32	0.004
1460506_s_at	NADH dehydrogenase (ubiquinone) 1, subcomplex unknown, 2	Ndufc2	-1.33	0.003
1415876_a_at	ribosomal protein S26	Rps26	-1.33	0.000
1417737_at	mitochondrial ribosomal protein S31	Mrps31	-1.33	0.005
1436292_a_at	ornithine decarboxylase antizyme	Oaz1	-1.33	0.001
1434135_at	UDP-GalNAc:betaGlcNAc beta 1,3-galactosaminyltransferase, polypeptide 2	B3galnt2	-1.33	0.010
1422807_at	ADP-ribosylation factor 5	Arf5	-1.33	0.007
1429158_at	F-box protein 28	Fbxo28	-1.33	0.001
1434822_at	periphilin 1	Pphln1	-1.33	0.004
1428086_at	dynamin 1-like	Dnm1l	-1.33	0.002

1429863_at	ring finger protein 127	Rnf127	-1.33	0.004
1452116_s_at	activating transcription factor 2	Atf2	-1.33	0.002
1460194_at	phytanoyl-CoA hydroxylase	Phyh	-1.33	0.000
1451669_at	protein phosphatase 1B, magnesium dependent, beta isoform	Ppm1b	-1.33	0.003
1421309_at	O-6-methylguanine-DNA methyltransferase	Mgmt	-1.33	0.001
1448104_at	aldehyde dehydrogenase family 6, subfamily A1	Aldh6a1	-1.33	0.004
1435112_a_at	ATP synthase, H+ transporting, mitochondrial F0 complex, subunit d	Atp5h	-1.33	0.003
1454635_at	F-box and leucine-rich repeat protein 3	Fbxl3	-1.33	0.006
1448563_at	prohibitin	Phb	-1.33	0.002
1416910_at	DnaJ (Hsp40) homolog, subfamily D, member 1	Dnajd1	-1.33	0.006
1422608_at	cAMP-regulated phosphoprotein 19	Arpp19	-1.34	0.010
1455819_at	ROD1 regulator of differentiation 1 (S. pombe)	Rod1	-1.34	0.004
1427992_a_at	RAB12, member RAS oncogene family	Rab12	-1.34	0.008
1436027_at	oxysterol binding protein-like 11	Osbpl11	-1.34	0.006
1418840_at	programmed cell death 4	Pdcd4	-1.34	0.006
1417998_at	telomerase binding protein, p23	MGI:1929282	-1.34	0.006
1449929_at	t-complex-associated-testis-expressed 1-like	Tcte1l	-1.34	0.000
1424639_a_at	3-hydroxy-3-methylglutaryl-Coenzyme A lyase	Hmgcl	-1.34	0.002
1450911_at	peptidylprolyl isomerase B	Ppib	-1.34	0.008
1455228_at	Wolf-Hirschhorn syndrome candidate 1 (human)	Whsc1	-1.34	0.000
1435859_x_at	proteasome (prosome, macropain) 26S subunit, ATPase 2	Psmc2	-1.34	0.003
1420485_at	nucleolar protein 7	Nol7	-1.34	0.006
1448264_a_at	eukaryotic translation initiation factor 3, subunit 2 (beta)	Eif3s2	-1.34	0.006
1425134_a_at	phosphatidylinositol glycan, class X	Pigx	-1.34	0.000
1423301_at	coatomer protein complex, subunit beta 1	Copb1	-1.35	0.000
1424585_at	RAN binding protein 10	Ranbp10	-1.35	0.009
1448442_a_at	proteasome (prosome, macropain) subunit, alpha type 3	Psma3	-1.35	0.000
1450634_at	ATPase, H+ transporting, V1 subunit A, isoform 1	Atp6v1a1	-1.35	0.004
1433749_at	Guanine nucleotide binding protein, alpha 13	Gna13	-1.35	0.004
1455286_at	BTB (POZ) domain containing 1	Btbd1	-1.35	0.005
1447062_at	Vacuolar protein sorting 24 (yeast)	Vps24	-1.35	0.007
1455073_at	cytidine and dCMP deaminase domain containing 1	Cdadc1	-1.35	0.001
1416212_at	mago-nashi homolog, proliferation-associated (Drosophila)	Magoh	-1.35	0.008
1449623_at	thioredoxin reductase 3	Txnrd3	-1.35	0.005
1417670_at	translocator of inner mitochondrial membrane 44	Timm44	-1.35	0.006
1426654_at	zinc finger, C3HC type 1	Zc3hc1	-1.35	0.003

1416453_x_at	ribosomal protein S12	Rps12	-1.35	0.006
1415967_at	NADH dehydrogenase (ubiquinone) flavoprotein 1	Ndufv1	-1.35	0.001
1424440_at	mitochondrial ribosomal protein S6	Mrps6	-1.35	0.009
1436885_a_at	calcium homeostasis endoplasmic reticulum protein	Cherp	-1.35	0.003
1428307_at	zinc finger, DHHC domain containing 13	Zdhhc13	-1.35	0.001
1452759_s_at	PTPRF interacting protein, binding protein 1 (liprin beta 1)	Ppfibp1	-1.35	0.005
1424242_at	biphenyl hydrolase-like (serine hydrolase, breast epithelial mucin-associated antigen)	Bphl	-1.35	0.000
1416829_at	ATP synthase, H+ transporting mitochondrial F1 complex, beta subunit	Atp5b	-1.35	0.002
1449374_at	pipecolic acid oxidase	Pipox	-1.35	0.002
1418976_s_at	cell death-inducing DNA fragmentation factor, alpha subunit-like effector B	Cideb	-1.35	0.001
1455245_x_at	ribosomal protein L13	Rpl13	-1.35	0.005
1423119_at	radial spokehead-like 2	Rshl2	-1.36	0.002
1415727_at	apolipoprotein A-I binding protein	Apoa1bp	-1.36	0.001
1448524_s_at	signal sequence receptor, delta	Ssr4	-1.36	0.005
1428714_at	progesterone receptor membrane component 2	Pgrmc2	-1.36	0.009
1425021_a_at	peroxisome biogenesis factor 16	Pex16	-1.36	0.001
1416377_at	programmed cell death protein 7	Pdcd7	-1.36	0.005
1427964_at	chemokine-like factor super family 8	Cklfsf8	-1.36	0.008
1424154_a_at	HESB like domain containing 1	Hbld1	-1.36	0.000
1433612_at	adaptor-related protein complex 2, sigma 1 subunit	Ap2s1	-1.36	0.004
1425568_a_at	transmembrane protein 33	Tmem33	-1.36	0.004
1425033_at	solute carrier family 17 (sodium phosphate), member 2	Slc17a2	-1.36	0.001
1448609_at	thiosulfate sulfurtransferase, mitochondrial	Tst	-1.36	0.001
1422241_a_at	NADH dehydrogenase (ubiquinone) 1 alpha subcomplex, 1	Ndufa1	-1.36	0.002
1450925_a_at	ribosomal protein S27-like	Rps27l	-1.36	0.002
1428341_at	zinc finger protein 655	Zfp655	-1.36	0.009
1452061_s_at	spermatid perinuclear RNA binding protein	Strbp	-1.36	0.003
1434860_at	NMDA receptor-regulated gene 3	Narg3	-1.36	0.002
1449506_a_at	eukaryotic translation elongation factor 1 delta (guanine nucleotide exchange protein)	Eef1d	-1.36	0.001
1449036_at	ring finger protein 128	Rnf128	-1.36	0.008
1417452_a_at	Finkel-Biskis-Reilly murine sarcoma virus (FBR-MuSV) ubiquitously expressed (fox derived)	Fau	-1.36	0.000
1416219_at	ribosomal protein L19	Rpl19	-1.36	0.000
1422061_at	aldo-keto reductase family 1, member C20	Akr1c20	-1.36	0.001
1418863_at	GATA binding protein 4	Gata4	-1.36	0.001
1424368_s_at	ubiquilin 1	Ubqln1	-1.36	0.000
1416393_at	gene rich cluster, C2f gene	Grcc2f	-1.36	0.001

1435897_at	ribosomal protein L32	Rpl32	-1.37	0.005
1416330_at	CD 81 antigen	Cd81	-1.37	0.002
1452585_at	mitochondrial ribosomal protein S28	Mrps28	-1.37	0.000
1448245_at	laminin receptor 1 (ribosomal protein SA)	Lamr1	-1.37	0.001
1424604_s_at	sulfatase modifying factor 1	Sumf1	-1.37	0.006
1456497_x_at	ribosomal protein S10	Rps10	-1.37	0.000
1451911_a_at	angiotensin converting enzyme	Ace	-1.37	0.004
1419061_at	ras homolog gene family, member D	Rhod	-1.37	0.008
1422510_at	CTD (carboxy-terminal domain, RNA polymerase II, polypeptide A) small phosphatase-like	Ctdspl	-1.37	0.006
1425276_at	fibrosin 1	Fbs1	-1.37	0.009
1427073_at	lactation elevated 1	Lace1	-1.37	0.001
1436291_a_at	dihydropyrimidinase	Dpys	-1.37	0.010
1436703_x_at	small nuclear RNA activating complex, polypeptide 2	Snapc2	-1.37	0.007
1448772_at	ubiquitin-conjugating enzyme E2A, RAD6 homolog (S. cerevisiae)	Ube2a	-1.37	0.001
1415681_at	mitochondrial ribosomal protein L43	Mrpl43	-1.37	0.000
1448014_s_at	ubiquitin specific protease 24	Usp24	-1.37	0.001
1423972_at	electron transferring flavoprotein, alpha polypeptide	Etfa	-1.37	0.000
1428428_at	Williams Beuren syndrome chromosome region 21	Wbscr21	-1.37	0.001
1423736_a_at	dymeclin	Dym	-1.37	0.010
1433834_at	Membrane-associated ring finger (C3HC4) 6	F830029L24Rik	-1.37	0.000
1419814_s_at	S100 calcium binding protein A1	S100a1	-1.38	0.002
1455290_at	zinc and ring finger 2	Znrf2	-1.38	0.005
1435103_x_at	phenylalanine-tRNA synthetase-like, beta subunit	Farslb	-1.38	0.000
1449183_at	catechol-O-methyltransferase	Comt	-1.38	0.001
1424235_at	ORM1-like 2 (S. cerevisiae)	Ormdl2	-1.38	0.002
1440037_at	Pre B-cell leukemia transcription factor 1	Pbx1	-1.38	0.004
1426854_a_at	SET translocation	Set	-1.38	0.003
1451205_at	proteasome (prosome, macropain) subunit, beta type 4	Psmb4	-1.38	0.001
1456614_at	ACN9 homolog (S. cerevisiae)	Acn9	-1.38	0.000
1428394_at	phytanoyl-CoA dioxygenase domain containing 1 /// leucine rich repeat containing 8	Phyhd1	-1.38	0.007
1420624_a_at	vesicle-associated membrane protein 8	Vamp8	-1.38	0.004
1455042_at	Transducin (beta)-like 1 X-linked	Tbl1x	-1.38	0.001
1458097_at	Cobl-like 1	Cobll1	-1.38	0.008
1448808_a_at	expressed in non-metastatic cells 2, protein	Nme2	-1.38	0.002
1416424_at	mannose-6-phosphate receptor binding protein 1	M6prbp1	-1.38	0.007
1425699_a_at	abhydrolase domain containing 14A	Abhd14a	-1.38	0.005

1425023_at	ubiquitin specific protease 3	Usp3	-1.38	0.005
1448244_at	lysophospholipase 1	Lypla1	-1.39	0.002
1415716_a_at	ribosomal protein S27	Rps27	-1.39	0.008
1427680_a_at	nuclear factor I/B	Nfib	-1.39	0.010
1453118_s_at	ribosomal protein L22	Rpl22	-1.39	0.006
1460544_at	MAK10 homolog, amino-acid N-acetyltransferase subunit, (S. cerevisiae)	Mak10	-1.39	0.002
1419287_at	HSPC171 protein	MGI:1913570	-1.39	0.001
1428538_s_at	retinoic acid receptor responder (tazarotene induced) 2	Rarres2	-1.39	0.003
1427337_at	aldehyde dehydrogenase 8 family, member A1	Aldh8a1	-1.39	0.000
1449445_x_at	microfibrillar-associated protein 1	Mfap1	-1.39	0.004
1449626_s_at	acyl-Coenzyme A binding domain containing 4	Acbd4	-1.39	0.006
1428172_at	PRP39 pre-mRNA processing factor 39 homolog (yeast)	Prpf39	-1.39	0.006
1456580_s_at	ATP synthase, H+ transporting, mitochondrial F1 complex, delta subunit	Atp5d	-1.39	0.006
1419559_at	cytochrome P450, family 4, subfamily f, polypeptide 14	Cyp4f14	-1.39	0.003
1451290_at	microtubule-associated protein 1 light chain 3 alpha	Map1lc3a	-1.39	0.005
1434600_at	tight junction protein 2	Tjp2	-1.39	0.000
1420967_at	solute carrier family 25 (mitochondrial carrier ornithine transporter), member 15	Slc25a15	-1.40	0.002
1424614_at	FGF receptor activating protein 1	MGI:2385286	-1.40	0.006
1426477_at	RAS p21 protein activator 1	Rasa1	-1.40	0.004
1435031_at	transmembrane protein induced by tumor necrosis factor alpha	MGI:2686991	-1.40	0.003
1423050_s_at	heterogeneous nuclear ribonucleoprotein U	Hnrpu	-1.40	0.000
1418335_a_at	diptheria toxin resistance protein required for diphthamide biosynthesis (Saccharomyces)-like 1	Dph2l1 /// MGI:217	-1.40	0.006
1460403_at	PC4 and SFRS1 interacting protein 1	Psip1	-1.40	0.000
1418506_a_at	peroxiredoxin 2	Prdx2	-1.40	0.001
1420123_at	T-cell leukemia translocation altered gene	Tcta	-1.40	0.008
1452927_x_at	triosephosphate isomerase 1	Tpi1	-1.40	0.009
1423727_at	cornichon homolog (Drosophila)	Cnih	-1.40	0.000
1434057_at	gene model 137, (NCBI)	Gm137	-1.40	0.001
1423338_at	coiled-coil domain containing 16	Ccdc16	-1.40	0.001
1448697_s_at	ribosomal protein L36a-like	Rpl36al	-1.40	0.005
1418763_at	nitrilase family, member 2	Nit2	-1.40	0.000
1448266_at	endothelial differentiation-related factor 1	Edf1	-1.40	0.003
1416485_at	translocase of inner mitochondrial membrane 23 homolog (yeast)	Timm23	-1.40	0.000
1451504_at	coiled-coil-helix-coiled-coil-helix domain containing 3	Chchd3	-1.40	0.001
1428405_at	host cell factor C1 regulator 1 (XPO1-dependent)	Hcfc1r1	-1.40	0.000
1450880_at	mitochondrial ribosomal protein L16	Mrpl16	-1.40	0.001

1435900_at	zinc finger protein 297B	Zfp297b	-1.40	0.004
1453728_a_at	mitochondrial ribosomal protein S17	Mrps17	-1.40	0.000
1436732_s_at	F-box and WD-40 domain protein 8	Fbxw8	-1.40	0.002
1423242_at	mitochondrial ribosomal protein S36	Mrps36	-1.41	0.000
1417741_at	liver glycogen phosphorylase	Pygl	-1.41	0.003
1455988_a_at	chaperonin subunit 6a (zeta)	Cct6a	-1.41	0.002
1456286_at	Component of oligomeric golgi complex 3	Cog3	-1.41	0.000
1448959_at	NADH dehydrogenase (ubiquinone) Fe-S protein 4	Ndufs4	-1.41	0.001
1440225_at	G protein-coupled receptor 116	Gpr116	-1.41	0.001
1423916_s_at	myeloid leukemia factor 2	Mlf2	-1.41	0.000
1433636_at	BMS1-like, ribosome assembly protein (yeast)	Bms1l	-1.41	0.004
1434503_s_at	lysosomal membrane glycoprotein 2	Lamp2	-1.41	0.001
1428365_a_at	protease, serine, 15	Prss15	-1.41	0.001
1423764_s_at	mitochondrial ribosomal protein L37	Mrpl37	-1.41	0.002
1437175_at	PDLIM1 interacting kinase 1 like	Pdik1l	-1.41	0.009
1433655_at	Ring finger protein 141	Rnf141	-1.41	0.005
1426269_at	synaptobrevin like 1	Sybl1	-1.41	0.005
1424877_a_at	aminolevulinate, delta-, dehydratase	Alad	-1.41	0.002
1454137_s_at	hemochromatosis type 2 (juvenile) (human homolog)	Hfe2	-1.41	0.003
1419913_at	Serine/threonine kinase receptor associated protein	Strap	-1.41	0.009
1423938_at	lethal giant larvae homolog 2	Llglh2	-1.42	0.007
1433562_s_at	ATP synthase, H+ transporting, mitochondrial F0 complex, subunit b, isoform 1	Atp5f1	-1.42	0.002
1426403_at	ARP1 actin-related protein 1 homolog B (yeast)	Actr1b	-1.42	0.000
1422647_at	ring finger protein 1	Ring1	-1.42	0.003
1450968_at	ubiquinol-cytochrome c reductase, Rieske iron-sulfur polypeptide 1	Uqcrfs1	-1.42	0.001
1449034_at	kallikrein B, plasma 1	Klkb1	-1.42	0.005
1416984_at	mitochondrial ribosomal protein S18A	Mrps18a	-1.42	0.000
1418560_at	pyruvate dehydrogenase E1 alpha 1	Pdha1	-1.42	0.008
1418986_a_at	ubiquitously expressed transcript	Uxt	-1.42	0.000
1421478_a_at	zinc finger protein 318	Zfp318	-1.42	0.003
1433774_x_at	component of oligomeric golgi complex 1	Cog1	-1.42	0.006
1450561_a_at	surfeit gene 1	Surf1	-1.42	0.000
1452213_at	testis expressed gene 2	Tex2	-1.42	0.000
1434515_at	nuclear receptor coactivator 1	Ncoa1	-1.42	0.001
1416271_at	PERP, TP53 apoptosis effector	Perp	-1.42	0.001
1416240_at	proteasome (prosome, macropain) subunit, beta type 7	Psmb7	-1.42	0.001

1449085_at	PHD finger protein 10	Phf10	-1.42	0.005
1448434_at	ring finger protein 103	Rnf103	-1.42	0.004
1426525_at	AT rich interactive domain 2 (Arid-rfx like)	Arid2	-1.42	0.001
1417326_a_at	anaphase promoting complex subunit 11 homolog (yeast)	Anapc11	-1.42	0.000
1416479_a_at	transmembrane protein 14C	Tmem14c	-1.42	0.007
1433518_at	leucine carboxyl methyltransferase 2	Lcmt2	-1.42	0.001
1430128_a_at	deleted in polyposis 1-like 1	Dp1l1	-1.43	0.003
1434890_at	optic atrophy 1 homolog (human)	Opa1	-1.43	0.007
1448227_at	growth factor receptor bound protein 7	Grb7	-1.43	0.006
1429590_at	transforming, acidic coiled-coil containing protein 1	Tacc1	-1.43	0.004
1418624_at	nuclease sensitive element binding protein 1	Nsep1	-1.43	0.000
1417846_at	Unc-51 like kinase 2 (C. elegans)	Ulk2	-1.43	0.003
1454948_at	ubiquitin specific protease 7	Usp7	-1.43	0.002
1436949_a_at	transcription elongation factor B (SIII), polypeptide 2	Tceb2	-1.43	0.002
1424746_at	Kinesin family member 1C	Kif1c	-1.43	0.001
1441733_s_at	nucleoporin 153	Nup153	-1.43	0.010
1416610_a_at	chloride channel 3	Clcn3	-1.43	0.008
1427888_a_at	spectrin alpha 2	Spna2	-1.43	0.000
1424338_at	solute carrier family 6 (neurotransmitter transporter, GABA), member 13	Slc6a13	-1.43	0.002
1455058_at	myotubularin related protein 9	Mtmr9	-1.43	0.003
1437687_x_at	FK506 binding protein 9	Fkbp9	-1.43	0.002
1451459_at	embryonic large molecule derived from yolk sac	MGI:1915033	-1.43	0.009
1452833_at	Rap guanine nucleotide exchange factor (GEF) 2	Rapgef2	-1.43	0.001
1449818_at	ATP-binding cassette, sub-family B (MDR/TAP), member 4	Abcb4	-1.43	0.004
1451512_s_at	3-hydroxyisobutyryl-Coenzyme A hydrolase	Hibch	-1.43	0.004
1455281_at	WD repeat domain 33	Wdr33	-1.43	0.005
1451462_a_at	interferon (alpha and beta) receptor 2	Ifnar2	-1.43	0.001
1422578_at	citrate synthase	Cs	-1.43	0.000
1433736_at	host cell factor C1	Hcfc1	-1.43	0.006
1417103_at	D-dopachrome tautomerase	Ddt	-1.43	0.006
1436189_at	Receptor (TNFRSF)-interacting serine-threonine kinase 1	Serpinb6a	-1.43	0.001
1423045_at	nuclear cap binding protein subunit 2	Ncbp2	-1.43	0.004
1443779_s_at	Mblk1-related protein-2	MGI:2443930	-1.43	0.003
1429582_at	BTB (POZ) domain containing 14A	Btbd14a	-1.44	0.004
1455470_x_at	LIM and SH3 protein 1	Lasp1	-1.44	0.002
1452740_at	myosin, heavy polypeptide 10, non-muscle	Myh10	-1.44	0.003

1455252_at	Tuberous sclerosis 1	Tsc1	-1.44	0.000
1417590_at	cytochrome P450, family 27, subfamily a, polypeptide 1	Cyp27a1	-1.44	0.000
1419169_at	mitogen-activated protein kinase 6	Mapk6	-1.44	0.000
1452048_at	mitochondrial ribosomal protein L12	Mrpl12	-1.44	0.003
1421014_a_at	citrate lyase beta like	Clybl	-1.44	0.006
1450705_at	RD RNA-binding protein	Rdbp	-1.44	0.002
1419557_a_at	transmembrane protein 9	Tmem9	-1.44	0.004
1433606_at	MAD homolog 4 interacting transcription coactivator 1	Mitc1	-1.44	0.006
1448943_at	neuropilin 1	Nrp1	-1.44	0.007
1434091_at	fatty acid amide hydrolase	Faah	-1.44	0.008
1448694_at	Jun oncogene	Jun	-1.44	0.004
1416395_at	guanylate kinase 1	Guk1	-1.44	0.000
1419173_at	aminoacylase 1	Acy1	-1.44	0.003
1423622_a_at	cyclin L1	Ccnl1	-1.44	0.004
1438910_a_at	stomatin	Stom	-1.44	0.004
1418137_at	mitochondrial ribosomal protein 63	Mrp63	-1.44	0.004
1449243_a_at	ribosomal protein S19	Rps19	-1.44	0.004
1434356_a_at	proteasome (prosome, macropain) subunit, alpha type 5	Psma5	-1.44	0.005
1437192_x_at	voltage-dependent anion channel 1	Vdac1	-1.44	0.005
1418950_at	dopamine receptor 2	Drd2	-1.45	0.006
1425143_a_at	NADH dehydrogenase (ubiquinone) Fe-S protein 1	Ndufs1	-1.45	0.003
1448784_at	TAF10 RNA polymerase II, TATA box binding protein (TBP)-associated factor	Taf10	-1.45	0.007
1439464_s_at	testis expressed gene 10	Tex10	-1.45	0.009
1435967_s_at	3-hydroxyisobutyrate dehydrogenase	Hibadh	-1.45	0.001
1460519_a_at	methyltransferase like 5	Mettl5	-1.45	0.002
1434604_at	eukaryotic translation initiation factor 5B	Eif5b	-1.45	0.002
1420139_s_at	HIV-1 Rev binding protein 2	Hrb2	-1.45	0.002
1424122_s_at	COMM domain containing 1	Commd1	-1.45	0.002
1418574_a_at	split hand/foot deleted gene 1	Shfdg1	-1.45	0.002
1422293_a_at	potassium channel tetramerisation domain containing 1	Kctd1	-1.45	0.003
1449269_at	coagulation factor V	F5	-1.45	0.009
1416349_at	mitochondrial ribosomal protein L34	Mrpl34	-1.45	0.001
1434513_at	gene model 542, (NCBI)	Gm542	-1.45	0.006
1438390_s_at	pituitary tumor-transforming 1	Pttg1	-1.45	0.003
1443362_at	Zinc finger protein 277	Zfp277	-1.45	0.001
1423667_at	methionine adenosyltransferase II, alpha	Mat2a	-1.45	0.000

1453725_a_at	mitchondrial ribosomal protein S7	Mrps7	-1.45	0.000
1439184_s_at	thioredoxin-like 5	Txnl5	-1.45	0.006
1448282_at	pleiotropic regulator 1, PRL1 homolog (Arabidopsis)	Plrg1	-1.45	0.001
1428159_s_at	NADH dehydrogenase (ubiquinone) 1, alpha/beta subcomplex, 1	Ndufab1	-1.46	0.001
1424576_s_at	cytochrome P450, family 2, subfamily c, polypeptide 44	Cyp2c44	-1.46	0.008
1458947_at	Fanconi anemia, complementation group C	Fancc	-1.46	0.010
1419450_at	ORM1-like 3 (S. cerevisiae)	Ormdl3	-1.46	0.003
1436947_a_at	thioredoxin-like 1	Txnl1	-1.46	0.000
1436715_s_at	CDP-diacylglycerol--inositol 3-phosphatidyltransferase (phosphatidylinositol synthase)	Cdipt	-1.46	0.001
1421374_a_at	FXYD domain-containing ion transport regulator 1	Fxyd1	-1.46	0.000
1460354_a_at	mitochondrial ribosomal protein L13	Mrpl13	-1.46	0.007
1418501_a_at	oxidation resistance 1	Oxr1	-1.46	0.000
1433540_x_at	protein phosphatase 1, catalytic subunit, beta isoform	Ppp1cb	-1.46	0.000
1422664_at	RAB10, member RAS oncogene family	Rab10	-1.46	0.000
1448286_at	hydroxyacyl-Coenzyme A dehydrogenase type II	Hadh2	-1.46	0.000
1451509_at	TAF9 RNA polymerase II, TATA box binding protein (TBP)-associated factor	Taf9	-1.46	0.005
1417673_at	growth factor receptor bound protein 14	Grb14	-1.46	0.001
1422457_s_at	SMT3 suppressor of mif two 3 homolog 3 (yeast)	Sumo3	-1.46	0.001
1431375_s_at	parvin, alpha	Parva	-1.46	0.003
1450504_a_at	1-acylglycerol-3-phosphate O-acyltransferase 3	Agpat3	-1.46	0.000
1419677_at	mannan-binding lectin serine protease 1	Masp1	-1.46	0.006
1416257_at	calpain 2	Capn2	-1.46	0.001
1417410_s_at	protein kinase C, iota	Prkci	-1.46	0.004
1455211_a_at	translocase of inner mitochondrial membrane 9 homolog (yeast)	Timm9	-1.46	0.003
1432330_at	complement factor H-like	LOC381294	-1.46	0.003
1417366_s_at	calmodulin 1	Calm1	-1.46	0.006
1448813_at	arylacetamide deacetylase (esterase)	Aadac	-1.47	0.010
1425531_at	zinc finger, HIT domain containing 1	Znhit1	-1.47	0.001
1440180_x_at	zinc finger and BTB domain containing 3	Zbtb3	-1.47	0.004
1436067_at	zinc finger and BTB domain containing 10	Zbtb10	-1.47	0.003
1433850_at	protein phosphatase 4, regulatory subunit 2	Ppp4r2	-1.47	0.001
1435682_at	leucyl-tRNA synthetase, mitochondrial	Lars2	-1.47	0.001
1417946_at	abhydrolase domain containing 3	Abhd3	-1.47	0.001
1448304_a_at	RAB6, member RAS oncogene family	Rab6	-1.47	0.000
1420619_a_at	amino-terminal enhancer of split	Aes	-1.47	0.000
1449710_s_at	ATP synthase, H+ transporting, mitochondrial F1 complex, alpha subunit, isoform 1	Atp5a1	-1.47	0.001

1422692_at	RNA polymerase II transcriptional coactivator	Rpo2tc1	-1.47	0.008
1456071_a_at	cytochrome c, somatic	Cycs	-1.47	0.008
1429108_at	ring finger protein 184	Rnf184	-1.47	0.007
1417316_at	thioesterase superfamily member 2	Them2	-1.47	0.002
1437856_at	inositol polyphosphate multikinase	Ipmk	-1.47	0.009
1449940_a_at	eukaryotic translation initiation factor 2B, subunit 4 delta	Eif2b4	-1.47	0.003
1417208_at	alpha-methylacyl-CoA racemase	Amacr	-1.47	0.000
1452206_at	succinate-Coenzyme A ligase, ADP-forming, beta subunit	Sucla2	-1.47	0.001
1430522_a_at	vesicle-associated membrane protein 5	Vamp5	-1.48	0.007
1418510_s_at	F-box only protein 8	Fbxo8	-1.48	0.006
1426773_at	mitofusin 1	Mfn1	-1.48	0.004
1450044_at	frizzled homolog 7 (Drosophila)	Fzd7	-1.48	0.003
1428257_s_at	dynein, cytoplasmic, light chain 2A	Dncl2a	-1.48	0.001
1448390_a_at	dehydrogenase/reductase (SDR family) member 3	Dhrs3	-1.48	0.002
1422471_at	peroxisomal biogenesis factor 13	Pex13	-1.48	0.000
1454640_at	coiled-coil-helix-coiled-coil-helix domain containing 7	Chchd7	-1.48	0.003
1417657_s_at	DnaJ (Hsp40) homolog, subfamily C, member 2	Dnajc2	-1.48	0.005
1443547_at	Solute carrier family 10 (sodium/bile acid cotransporter family), member 1	Slc10a1	-1.48	0.003
1416604_at	cytochrome c-1	Cyc1	-1.48	0.000
1450090_at	zinc finger protein 101	Zfp101	-1.48	0.003
1455777_x_at	hydroxysteroid (17-beta) dehydrogenase 4	Hsd17b4	-1.48	0.007
1420673_a_at	acyl-Coenzyme A oxidase 2, branched chain	Acox2	-1.48	0.007
1455991_at	cysteine conjugate-beta lyase 2	Ccbl2	-1.49	0.008
1442660_at	Yippee-like 1 (Drosophila)	Ypel1	-1.49	0.000
1448869_a_at	mitochondrial ribosomal protein S16	Mrps16	-1.49	0.000
1433720_s_at	Nur77 downstream gene 2	MGI:2143558	-1.49	0.001
1450994_at	Rho-associated coiled-coil forming kinase 1	Rock1	-1.49	0.001
1452610_at	zinc finger protein 99	Zfp99	-1.49	0.004
1449028_at	ras homolog gene family, member U	Rhou	-1.49	0.006
1416427_at	cyclin I	Ccni	-1.49	0.000
1439167_at	peroxisomal trans-2-enoyl-CoA reductase	Pecr	-1.49	0.001
1452986_at	homogentisate 1, 2-dioxygenase	Hgd	-1.49	0.001
1424912_at	solute carrier family 25 (mitochondrial carrier, peroxisomal membrane protein), member 17	Slc25a17	-1.50	0.008
1416500_at	SAC1 (supressor of actin mutations 1, homolog)-like (S. cerevisiae)	Sacm1l	-1.50	0.000
1452000_s_at	seryl-aminoacyl-tRNA synthetase 1	Sars1	-1.50	0.003
1460249_at	ligand of numb-protein X 2	Lnx2	-1.50	0.006

1438091_a_at	H2A histone family, member Z	H2afz	-1.50	0.002
1434435_s_at	cytochrome c oxidase, subunit XVII assembly protein homolog (yeast)	Cox17	-1.50	0.000
1448203_at	ATP synthase, H+ transporting, mitochondrial F0 complex, subunit g	Atp5l	-1.50	0.000
1450186_s_at	GNAS (guanine nucleotide binding protein, alpha stimulating) complex locus	Gnas	-1.50	0.001
1417296_at	activating transcription factor 1	Atf1	-1.50	0.001
1425537_at	protein phosphatase 1A, magnesium dependent, alpha isoform	Ppm1a	-1.50	0.007
1434367_s_at	nuclear transport factor 2	Nutf2	-1.50	0.001
1427269_at	splicing factor, arginine/serine-rich 11	Sfrs11	-1.50	0.008
1416088_a_at	ribosomal protein S15	Rps15	-1.50	0.007
1428935_at	calnexin	Canx	-1.50	0.000
1418472_at	aspartoacylase (aminoacylase) 2	Aspa	-1.50	0.002
1416616_s_at	caseinolytic protease, ATP-dependent, proteolytic subunit homolog (E. coli)	Clpp	-1.51	0.001
1456194_a_at	Parkinson disease (autosomal recessive, early onset) 7	Park7	-1.51	0.006
1424095_at	RNA terminal phosphate cyclase domain 1	Rtcd1	-1.51	0.002
1457627_x_at	ropporin 1-like	Ropn1l	-1.51	0.002
1417531_at	cytochrome P450, family 2, subfamily j, polypeptide 5	Cyp2j5	-1.51	0.000
1418914_s_at	betaine-homocysteine methyltransferase 2	Bhmt2	-1.51	0.004
1451515_s_at	glycine-N-acyltransferase	Glyat	-1.51	0.000
1460195_at	mitochondrial ribosomal protein S11	Mrps11	-1.51	0.000
1457398_at	Guanosine diphosphate (GDP) dissociation inhibitor 3	Gdi3	-1.51	0.008
1437668_at	chemokine (C-C motif) receptor-like 1	Ccrl1	-1.51	0.009
1416663_at	NADH dehydrogenase (ubiquinone) 1 alpha subcomplex, 9	Ndufa9	-1.51	0.000
1416337_at	ubiquinol-cytochrome c reductase binding protein	Uqcrb	-1.51	0.003
1417294_at	aldo-keto reductase family 7, member A5 (aflatoxin aldehyde reductase)	Akr7a5	-1.52	0.004
1454987_a_at	H2-K region expressed gene 6	H2-Ke6	-1.52	0.009
1416394_at	Bcl2-associated athanogene 1	Bag1	-1.52	0.005
1456436_x_at	ribosomal protein S20	Rps20	-1.52	0.001
1457473_at	chromodomain helicase DNA binding protein 1	Chd1	-1.52	0.008
1433679_at	far upstream element (FUSE) binding protein 3	Fubp3	-1.52	0.006
1436724_a_at	ligatin	Lgtn	-1.52	0.000
1418113_at	cytochrome P450, family 2, subfamily d, polypeptide 10	Cyp2d10	-1.52	0.007
1417801_a_at	protein tyrosine phosphatase, receptor-type, F interacting protein, binding protein 2	Ppfibp2	-1.52	0.001
1425129_a_at	transaldolase 1	Taldo1	-1.52	0.007
1451366_at	COP9 (constitutive photomorphogenic) homolog, subunit 6 (Arabidopsis thaliana)	Cops6	-1.52	0.008
1429048_at	biogenesis of lysosome-related organelles complex-1, subunit 2	Bloc1s2	-1.52	0.001
1425419_a_at	v-raf-1 leukemia viral oncogene 1	Raf1	-1.52	0.001

1424047_at	2-deoxyribose-5-phosphate aldolase homolog (C. elegans)	Dera	-1.52	0.002
1451555_at	neurolysin (metallopeptidase M3 family)	Nln	-1.52	0.001
1415677_at	dehydrogenase/reductase (SDR family) member 1	Dhrs1	-1.52	0.002
1426760_at	importin 8	Ipo8	-1.52	0.005
1416834_x_at	NADH dehydrogenase (ubiquinone) 1 beta subcomplex, 2	Ndufb2	-1.52	0.003
1446861_at	glucosamine (N-acetyl)-6-sulfatase	Gns	-1.52	0.003
1451346_at	methylthioadenosine phosphorylase	Mtap	-1.52	0.000
1435072_at	zinc finger, FYVE domain containing 1	Zfyve1	-1.53	0.002
1453096_x_at	ribosomal protein L27	Rpl27	-1.53	0.009
1448153_at	cytochrome c oxidase, subunit Va	Cox5a	-1.53	0.000
1433824_x_at	G-rich RNA sequence binding factor 1	Grsf1	-1.53	0.005
1427282_a_at	frataxin	Fxn	-1.53	0.002
1455057_at	Guanine monphosphate synthetase	Gmps	-1.53	0.007
1424836_a_at	CLIP associating protein 2	Clasp2	-1.53	0.000
1427729_at	EH-domain containing 2	Ehd2	-1.53	0.007
1456059_at	proteasome (prosome, macropain) 26S subunit, non-ATPase, 11	Psmd11	-1.53	0.003
1439393_x_at	protein phosphatase 2A, regulatory subunit B (PR 53)	Ppp2r4	-1.53	0.004
1416807_at	ribosomal protein L36a	Rpl36a	-1.53	0.009
1438644_x_at	COMM domain containing 9	Commd9	-1.53	0.005
1452494_s_at	solute carrier organic anion transporter family, member 1b2	Slco1b2	-1.53	0.005
1416335_at	macrophage migration inhibitory factor	Mif	-1.54	0.005
1431125_a_at	threonyl-tRNA synthetase-like 1	Tarsl1	-1.54	0.003
1417236_at	EH-domain containing 3	Ehd3	-1.54	0.006
1451012_a_at	cold shock domain protein A	Csda	-1.54	0.003
1449144_at	guanine nucleotide binding protein, alpha 11	Gna11	-1.54	0.002
1459987_s_at	chaperonin subunit 3 (gamma)	Cct3	-1.54	0.000
1428214_at	translocase of outer mitochondrial membrane 7 homolog (yeast)	Tomm7	-1.54	0.000
1452940_x_at	phosphatidylinositol transfer protein, cytoplasmic 1	Pitpnc1	-1.54	0.007
1424345_s_at	ubiquitin-conjugating enzyme E2M (UBC12 homolog, yeast)	Ube2m	-1.54	0.004
1416143_at	ATP synthase, H+ transporting, mitochondrial F0 complex, subunit F	Atp5j	-1.54	0.000
1422731_at	LIM domains containing 1	Limd1	-1.54	0.000
1423296_at	proteasome (prosome, macropain) 26S subunit, non-ATPase, 8	Psmd8	-1.54	0.001
1432000_a_at	death effector domain-containing	Dedd	-1.54	0.001
1417449_at	peroxisomal acyl-CoA thioesterase 1	Pte1	-1.54	0.001
1449001_at	isovaleryl coenzyme A dehydrogenase	Ivd	-1.54	0.002
1456314_x_at	CDK2 (cyclin-dependent kinase 2)-associated protein 1	Cdk2ap1	-1.54	0.004

1448565_at	protein phosphatase 1, regulatory (inhibitor) subunit 11	Ppp1r11	-1.54	0.005
1450897_at	Rho GTPase activating protein 5	Arhgap5	-1.55	0.001
1436848_x_at	inositol (myo)-1(or 4)-monophosphatase 1	Impa1	-1.55	0.010
1429328_at	NSFL1 (p97) cofactor (p47)	Nsfl1c	-1.55	0.003
1444598_at	Ets variant gene 6 (TEL oncogene)	Etv6	-1.55	0.004
1421441_at	angiopoietin 1	Angpt1	-1.55	0.000
1449983_a_at	NAD(P)H dehydrogenase, quinone 2	Nqo2	-1.56	0.003
1428153_at	mitochondrial ribosomal protein S10	Mrps10	-1.56	0.002
1416400_at	pyrroline-5-carboxylate reductase-like	Pycrl	-1.56	0.010
1416547_at	NADH dehydrogenase (ubiquinone) 1 beta subcomplex 3	Ndufb3	-1.56	0.000
1448792_a_at	cytochrome P450, family 2, subfamily f, polypeptide 2	Cyp2f2	-1.56	0.006
1419456_at	dicarbonyl L-xylulose reductase	Dcxr	-1.56	0.002
1428822_a_at	sorting nexing 24	Snx24	-1.56	0.004
1419043_a_at	interferon inducible GTPase 1	Iigp1	-1.56	0.000
1452680_at	small nuclear ribonucleoprotein D2	Snrpd2	-1.56	0.010
1435474_at	TAF5 RNA polymerase II, TATA box binding protein (TBP)-associated factor	Taf5	-1.56	0.005
1423149_at	S-phase kinase-associated protein 1A	Skp1a	-1.56	0.007
1418815_at	cadherin 2	Cdh2	-1.56	0.005
1438631_x_at	tetratricopeptide repeat domain 13	Ttc13	-1.56	0.006
1437016_x_at	RAP2C, member of RAS oncogene family	Rap2c	-1.56	0.000
1426769_s_at	mastermind like 1 (Drosophila)	Maml1	-1.56	0.002
1442760_x_at	Reticulon 4	Rtn4	-1.57	0.007
1459120_at	erythrocyte protein band 4.1-like 5	Epb4.1l5	-1.57	0.005
1420631_a_at	bladder cancer associated protein homolog (human)	Blcap	-1.57	0.001
1453100_at	casein kinase II, alpha 2, polypeptide	Csnk2a2	-1.57	0.003
1416074_a_at	ribosomal protein L28	Rpl28	-1.57	0.002
1438177_x_at	ectonucleoside triphosphate diphosphohydrolase 4	Entpd4	-1.57	0.010
1419363_a_at	mitochondrial ribosomal protein L35	Mrpl35	-1.57	0.001
1433983_at	Cnksr family member 3	Cnksr3	-1.57	0.000
1422872_at	bone morphogenetic protein receptor, type 1B	Bmpr1b	-1.57	0.005
1417560_at	sideroflexin 1	Sfxn1	-1.57	0.005
1454929_s_at	scaffold attachment factor B	Safb	-1.57	0.000
1460648_at	nuclear receptor subfamily 2, group F, member 6	Nr2f6	-1.57	0.003
1450401_at	nuclear receptor coactivator 6 interacting protein	Ncoa6ip	-1.57	0.008
1434358_x_at	ribosomal protein S21	Rps21	-1.57	0.000
1428998_at	PHD finger protein 3	Phf3	-1.57	0.002

1450970_at	glutamate oxaloacetate transaminase 1, soluble	Got1	-1.57	0.001
1449734_s_at	Bardet-Biedl syndrome 4 homolog (human)	Bbs4	-1.58	0.010
1448052_at	cingulin-like 1	Cgnl1	-1.58	0.008
1415891_at	succinate-CoA ligase, GDP-forming, alpha subunit	Suclg1	-1.58	0.000
1428757_at	aminoadipate-semialdehyde dehydrogenase-phosphopantetheinyl transferase	Aasdhppt	-1.58	0.001
1453106_a_at	RNA (guanine-7-) methyltransferase	Rnmt	-1.58	0.000
1451399_at	brain protein 17	MGI:1930773	-1.58	0.001
1447027_s_at	lipoic acid synthetase	Lias	-1.58	0.000
1435395_s_at	ATP synthase, H+ transporting, mitochondrial F0 complex, subunit f, isoform 2	Atp5j2	-1.58	0.000
1427914_a_at	transcription elongation factor B (SIII), polypeptide 1	Tceb1	-1.58	0.000
1417619_at	growth arrest and DNA-damage-inducible, gamma interacting protein 1	Gadd45gip1	-1.58	0.001
1427965_at	single-stranded DNA binding protein 1	Ssbp1	-1.58	0.009
1448760_at	zinc finger protein 68	Zfp68	-1.59	0.002
1433603_at	NADH dehydrogenase (ubiquinone) Fe-S protein 6	Ndufs6	-1.59	0.000
1416284_at	mitochondrial ribosomal protein L28	Mrpl28	-1.59	0.000
1419436_at	complement component factor h-like 1	Cfhl1	-1.59	0.001
1448322_a_at	cytochrome c oxidase subunit IV isoform 1	Cox4i1	-1.59	0.005
1450948_a_at	mitochondrial ribosomal protein L1	Mrpl1	-1.59	0.003
1423534_at	programmed cell death 2	Pdcd2	-1.59	0.000
1441214_at	synaptotagmin-like homologue lacking C2 domains b	MGI:2443248	-1.59	0.002
1437142_a_at	phosphatidylinositol glycan, class O	Pigo	-1.59	0.007
1448712_at	choroidermia	Chm	-1.59	0.007
1448391_at	RAB9, member RAS oncogene family	Rab9	-1.59	0.000
1454824_s_at	mitochondrial tumor suppressor 1	Mtus1	-1.59	0.000
1431232_a_at	MAX gene associated	Mga	-1.59	0.000
1429642_at	AN1, ubiquitin-like, homolog (Xenopus laevis)	Anubl1	-1.59	0.005
1452583_s_at	galactose mutarotase	Galm	-1.59	0.007
1422478_a_at	acetyl-Coenzyme A synthetase 2 (ADP forming)	Acas2	-1.59	0.004
1422780_at	peroxisomal membrane protein 4	Pxmp4	-1.59	0.005
1420913_at	solute carrier organic anion transporter family, member 2a1	Slco2a1	-1.60	0.004
1450187_a_at	galactose-1-phosphate uridyl transferase	Galt	-1.60	0.000
1457664_x_at	Complement component 2 (within H-2S)	C2	-1.60	0.005
1417434_at	glycerol phosphate dehydrogenase 2, mitochondrial	Gpd2	-1.60	0.006
1454159_a_at	insulin-like growth factor binding protein 2	Igfbp2	-1.60	0.010
1456588_x_at	cytochrome c oxidase, subunit Vb	Cox5b	-1.60	0.001
1440311_at	Sorbin and SH3 domain containing 1	Sorbs1	-1.60	0.005

1416772_at	carnitine palmitoyltransferase 2	Cpt2	-1.60	0.009
1424842_a_at	Rho GTPase activating protein 24	Arhgap24	-1.60	0.007
1434329_s_at	adiponectin receptor 2	Adipor2	-1.60	0.000
1420826_at	leucine zipper-EF-hand containing transmembrane protein 1	Letm1	-1.60	0.002
1429050_at	cysteine-rich hydrophobic domain 2	Chic2	-1.60	0.000
1435494_s_at	desmoplakin	Dsp	-1.60	0.007
1454014_a_at	McKusick-Kaufman syndrome protein	Mkks	-1.60	0.006
1423488_at	monocyte to macrophage differentiation-associated	Mmd	-1.60	0.000
1426531_at	zinc finger, MYND domain containing 11	Zmynd11	-1.60	0.001
1424172_at	hydroxyacyl glutathione hydrolase	Hagh	-1.61	0.000
1447720_x_at	Protein kinase, cAMP dependent, catalytic, alpha	Prkaca	-1.61	0.002
1437342_x_at	pituitary tumor-transforming 1 interacting protein	Pttg1ip	-1.61	0.000
1449934_at	purine rich element binding protein A	Pura	-1.61	0.000
1433470_a_at	inner membrane protein, mitochondrial	Immt	-1.61	0.002
1419006_s_at	pellino 2	Peli2	-1.61	0.010
1416020_a_at	ATP synthase, H+ transporting, mitochondrial F0 complex, subunit c (subunit 9), isoform 1	Atp5g1	-1.61	0.001
1448610_a_at	superoxide dismutase 2, mitochondrial	Sod2	-1.61	0.002
1417418_s_at	cytochrome c oxidase, subunit VI a, polypeptide 1	Cox6a1	-1.61	0.001
1425559_a_at	SA rat hypertension-associated homolog	Sah	-1.62	0.006
1460198_a_at	proteasome (prosome, macropain) subunit, beta type 3	Psmb3	-1.62	0.000
1419068_at	RAB guanine nucleotide exchange factor (GEF) 1	Rabgef1	-1.62	0.003
1417891_at	T-complex expressed gene 1	Tce1	-1.62	0.001
1429454_at	GTPase activating protein and VPS9 domains 1	Gapvd1	-1.62	0.000
1449406_at	cysteine and histidine rich 1	Cyhr1	-1.62	0.001
1448155_at	programmed cell death 6 interacting protein	Pdcd6ip	-1.62	0.005
1436757_a_at	cytochrome c oxidase, subunit VIb polypeptide 1	Cox6b1	-1.62	0.006
1418242_at	Fas-associated factor 1	Faf1	-1.62	0.000
1417080_a_at	signaling intermediate in Toll pathway-evolutionarily conserved	MGI:1349469	-1.62	0.001
1418940_at	sulfotransferase family 1B, member 1	Sult1b1	-1.62	0.008
1422070_at	alcohol dehydrogenase 4 (class II), pi polypeptide	Adh4	-1.63	0.004
1449817_at	ATP-binding cassette, sub-family B (MDR/TAP), member 11	Abcb11	-1.63	0.002
1426796_at	Taxilin	Txln	-1.63	0.008
1416971_at	cytochrome c oxidase, subunit VIIa 2	Cox7a2	-1.63	0.003
1448292_at	ubiquinol-cytochrome c reductase (6.4kD) subunit	Uqcr	-1.63	0.000
1451312_at	NADH dehydrogenase (ubiquinone) Fe-S protein 7	Ndufs7	-1.63	0.000
1416522_a_at	gene rich cluster, C10 gene	Grcc10	-1.63	0.000

1416968_a_at	hydroxy-delta-5-steroid dehydrogenase, 3 beta- and steroid delta-isomerase 7	Hsd3b7	-1.63	0.001
1448331_at	NADH dehydrogenase (ubiquinone) 1 beta subcomplex, 7	Ndufb7	-1.63	0.000
1418886_s_at	isocitrate dehydrogenase 3 (NAD+) beta	Idh3b	-1.63	0.002
1437593_x_at	apoptosis inhibitor 5	Api5	-1.64	0.002
1418585_at	cyclin H	Ccnh	-1.64	0.003
1438317_a_at	endonuclease G	Endog	-1.64	0.005
1460035_at	B-cell receptor-associated protein 37	Bcap37	-1.64	0.002
1448198_a_at	NADH dehydrogenase (ubiquinone) 1 beta subcomplex 8	Ndufb8	-1.64	0.000
1422032_a_at	zinc finger, A20 domain containing 3	Za20d3	-1.64	0.001
1430474_a_at	mitochondrial carrier homolog 2 (C. elegans)	Mtch2	-1.64	0.003
1449403_at	phosphodiesterase 9A	Pde9a	-1.64	0.003
1428744_s_at	Bri3 binding protein	Bri3bp	-1.64	0.003
1451185_at	splicing factor 3b, subunit 5	Sf3b5	-1.64	0.007
1451638_s_at	armadillo repeat containing 1	Armc1	-1.64	0.001
1456118_at	methyltransferase like 2	Mettl2	-1.65	0.000
1428729_at	cerebral cavernous malformations 1	Ccm1	-1.65	0.000
1427033_at	dynamin binding protein	Dnmbp	-1.65	0.001
1448589_at	NADH dehydrogenase (ubiquinone) 1 beta subcomplex, 5	Ndufb5	-1.65	0.000
1439435_x_at	phosphoglycerate kinase 1	Pgk1	-1.65	0.000
1429709_at	peptidase (mitochondrial processing) beta	Pmpcb	-1.65	0.000
1447432_s_at	zinc finger protein 263	Zfp263	-1.65	0.008
1424835_at	glutathione S-transferase, mu 4	Gstm4	-1.65	0.006
1419931_at	ATP-binding cassette, sub-family B (MDR/TAP), member 7	Abcb7	-1.65	0.006
1455521_at	Kruppel-like factor 12	Klf12	-1.65	0.001
1416595_at	mitochondrial ribosomal protein S22	Mrps22	-1.65	0.002
1437976_x_at	ribosomal protein L23a	Rpl23a	-1.65	0.001
1417458_s_at	CDC28 protein kinase regulatory subunit 2	Cks2	-1.65	0.000
1438000_x_at	zinc finger protein 622	Zfp622	-1.66	0.000
1425196_a_at	histidine triad nucleotide binding protein 2	Hint2	-1.66	0.002
1426271_at	SMC5 structural maintenance of chromosomes 5-like 1 (yeast)	Smc5l1	-1.66	0.002
1419650_at	zinc finger RNA binding protein	Zfr	-1.66	0.002
1417566_at	abhydrolase domain containing 5	Abhd5	-1.66	0.002
1436946_s_at	guanine nucleotide binding protein (G protein), gamma 5 subunit	Gng5	-1.66	0.001
1448629_at	Hermansky-Pudlak syndrome 4 homolog (human)	Hps4	-1.66	0.007
1418301_at	interferon regulatory factor 6	Irf6	-1.66	0.001
1416589_at	secreted acidic cysteine rich glycoprotein	Sparc	-1.66	0.006

1441253_at	Regulatory factor X, 3 (influences HLA class II expression)	Rfx3	-1.66	0.002
1452596_at	polymerase (RNA) II (DNA directed) polypeptide K	Polr2k	-1.66	0.003
1450638_at	programmed cell death 5	Pdcd5	-1.66	0.000
1423137_at	v-ral simian leukemia viral oncogene homolog A (ras related)	Rala	-1.67	0.001
1423857_at	mitochondrial ribosomal protein L30	Mrpl30	-1.67	0.001
1418236_s_at	autophagy 5-like (S. cerevisiae)	Apg5l	-1.67	0.004
1427938_at	c-myc binding protein	Mycbp	-1.67	0.007
1449443_at	2,4-dienoyl CoA reductase 1, mitochondrial	Decr1	-1.67	0.002
1452621_at	pterin 4 alpha carbinolamine dehydratase/dimerization cofactor of hepatocyte nuclear factor 1 alpha (TC	Pcbd2	-1.67	0.000
1451271_a_at	acetyl-Coenzyme A acetyltransferase 1	Acat1	-1.67	0.000
1417177_at	galactokinase 1	Galk1	-1.67	0.001
1457107_at	N-myristoyltransferase 2	Nmt2	-1.67	0.008
1416085_s_at	zinc finger, A20 domain containing 2	Za20d2	-1.67	0.002
1456279_a_at	B-cell receptor-associated protein 31	Bcap31	-1.68	0.007
1417772_at	glyoxylate reductase/hydroxypyruvate reductase	Grhpr	-1.68	0.000
1437237_x_at	zinc finger protein 110	Zfp110	-1.68	0.006
1418566_s_at	NudC domain containing 2	Nudcd2	-1.68	0.003
1435176_a_at	inhibitor of DNA binding 2	Id2	-1.68	0.006
1449585_at	interleukin 1 receptor accessory protein	Il1rap	-1.68	0.007
1452944_at	synaptogyrin 2	Syngr2	-1.68	0.005
1451695_a_at	glutathione peroxidase 4	Gpx4	-1.68	0.001
1436846_x_at	Tyrosine 3-monooxygenase/tryptophan 5-monooxygenase activation protein, theta polypeptide	Ywhaq	-1.69	0.007
1423109_s_at	solute carrier family 25 (mitochondrial carnitine/acylcarnitine translocase), member 20	Slc25a20	-1.69	0.003
1452735_at	PEST-containing nuclear protein	Pcnp	-1.69	0.000
1452184_at	NADH dehydrogenase (ubiquinone) 1 beta subcomplex, 9	Ndufb9	-1.69	0.000
1416779_at	serum deprivation response	Sdpr	-1.69	0.003
1417016_at	MAP kinase-activated protein kinase 5	Mapkapk5	-1.69	0.002
1416305_at	SH3-domain binding protein 4	Sh3bp4	-1.69	0.001
1417431_a_at	sphingosine kinase 2	Sphk2	-1.69	0.004
1418516_at	metal response element binding transcription factor 2	Mtf2	-1.69	0.006
1456748_a_at	4-nitrophenylphosphatase domain and non-neuronal SNAP25-like protein homolog 1 (C. elegans)	Nipsnap1	-1.69	0.000
1459973_x_at	dipeptidylpeptidase 4	Dpp4	-1.69	0.002
1435023_at	SH3 domain protein 1B	Sh3d1B	-1.69	0.000
1429291_at	proteasome (prosome, macropain) 26S subunit, non-ATPase, 1	Psmd1	-1.69	0.007
1419560_at	lipase, hepatic	Lipc	-1.69	0.004
1447961_s_at	mitochondrial ribosomal protein L38	Mrpl38	-1.69	0.003

1455827_at	muscleblind-like 2	Mbnl2	-1.70	0.001
1448427_at	NADH dehydrogenase (ubiquinone) 1 alpha subcomplex, 6 (B14)	Ndufa6	-1.70	0.003
1452692_a_at	NADH dehydrogenase (ubiquinone) flavoprotein 2	Ndufv2	-1.70	0.000
1452144_a_at	mitochondrial ribosomal protein L44	Mrpl44	-1.70	0.003
1438971_x_at	ubiquitin-conjugating enzyme E2H	Ube2h	-1.70	0.001
1423210_a_at	nucleolar protein family A, member 3	Nola3	-1.71	0.000
1457696_at	Rab interacting lysosomal protein	Rilp	-1.71	0.002
1451600_s_at	esterase 31-like /// esterase 31	LOC13909 /// Es31	-1.71	0.008
1435995_at	mitochondrial ribosomal protein L22	Mrpl22	-1.71	0.005
1426265_x_at	dihydrolipoamide S-acetyltransferase (E2 component of pyruvate dehydrogenase complex)	Dlat	-1.71	0.001
1423737_at	NADH dehydrogenase (ubiquinone) Fe-S protein 3	Ndufs3	-1.71	0.000
1459154_at	Eyes absent 3 homolog (Drosophila)	Eya3	-1.71	0.010
1460348_at	MAD2 mitotic arrest deficient-like 2 (yeast)	Mad2l2	-1.71	0.003
1438562_a_at	protein tyrosine phosphatase, non-receptor type 2	Ptpn2	-1.71	0.004
1453207_at	Purkinje cell protein 2 (L7)	Pcp2	-1.71	0.009
1422704_at	glycerol kinase	Gyk	-1.72	0.000
1424329_a_at	proline-rich Gla (G-carboxyglutamic acid) polypeptide 2	Prrg2	-1.72	0.000
1451219_at	ORM1-like 1 (S. cerevisiae)	Ormdl1	-1.72	0.002
1429352_at	molybdenum cofactor sulfurase	Mocos	-1.72	0.000
1420562_at	secreted Ly6/Plaur domain containing 1	Slurp1	-1.72	0.000
1431833_a_at	3-hydroxy-3-methylglutaryl-Coenzyme A synthase 2	Hmgcs2	-1.72	0.002
1426969_at	tripartite motif protein 23	Trim23	-1.72	0.000
1429057_at	NMDA receptor regulated 1-like	Narg1l	-1.72	0.000
1433510_x_at	ribosomal protein L36	Rpl36	-1.72	0.000
1421878_at	mitogen activated protein kinase 9	Mapk9	-1.72	0.004
1450865_s_at	mitochondrial ribosomal protein S24	Mrps24	-1.72	0.000
1456109_a_at	mitochondrial ribosomal protein S15	Mrps15	-1.72	0.001
1416058_s_at	ATP synthase, H+ transporting, mitochondrial F1 complex, gamma polypeptide 1	Atp5c1	-1.73	0.003
1436545_at	deltex 4 homolog (Drosophila)	Dtx4	-1.73	0.006
1423405_at	tissue inhibitor of metalloproteinase 4	Timp4	-1.73	0.007
1427042_at	mal, T-cell differentiation protein 2	Mal2	-1.73	0.009
1452790_x_at	NADH dehydrogenase (ubiquinone) 1 alpha subcomplex, 3	Ndufa3	-1.73	0.000
1460548_a_at	Era (G-protein)-like 1 (E. coli)	Eral1	-1.73	0.001
1416278_a_at	ATP synthase, H+ transporting, mitochondrial F1 complex, O subunit	Atp5o	-1.73	0.000
1453229_s_at	ubiquinol-cytochrome c reductase hinge protein	Uqcrh	-1.73	0.004
1431665_a_at	translocase of inner mitochondrial membrane 8 homolog b (yeast)	Timm8b	-1.73	0.002

1424017_a_at	histidine triad nucleotide binding protein 1	Hint1	-1.73	0.001
1440223_at	RNA binding motif protein 6	Rbm6	-1.74	0.000
1418777_at	chemokine (C-C motif) ligand 25	Ccl25	-1.74	0.002
1436012_s_at	secernin 2	Scrn2	-1.74	0.000
1434834_at	suppressor of cytokine signaling 7	Socs7	-1.74	0.002
1436938_at	RNA binding motif, single stranded interacting protein	Rbms3	-1.74	0.000
1424530_at	SEC14-like 2 (S. cerevisiae)	Sec14l2	-1.74	0.005
1450738_at	kinesin family member 21A	Kif21a	-1.74	0.004
1419640_at	purine rich element binding protein B	Purb	-1.74	0.000
1433855_at	4-aminobutyrate aminotransferase	Abat	-1.74	0.001
1449834_at	PDZ domain containing, X chromosome	Pdzx	-1.74	0.002
1449459_s_at	ankyrin repeat and SOCS box-containing protein 13	Asb13	-1.75	0.002
1445105_at	P300/CBP-associated factor	Pcaf	-1.75	0.003
1451084_at	electron transferring flavoprotein, dehydrogenase	Etfdh	-1.75	0.001
1422493_at	coproporphyrinogen oxidase /// similar to Cpo protein	Cpox /// LOC43636	-1.75	0.007
1436495_s_at	zinc finger protein 260	Zfp260	-1.75	0.008
1437155_a_at	WW domain containing transcription regulator 1	Wwtr1	-1.75	0.009
1439813_at	Signal peptide peptidase 3	Sppl3	-1.75	0.005
1436615_a_at	ornithine transcarbamylase	Otc	-1.75	0.000
1435155_at	cingulin	Cgn	-1.75	0.002
1434253_s_at	transmembrane and coiled coil domains 3	Tmcc3	-1.75	0.004
1426810_at	jumonji domain containing 1A	Jmjd1a	-1.75	0.005
1433901_at	GPI-anchored membrane protein 1	Gpiap1	-1.75	0.006
1447791_s_at	guanine nucleotide binding protein, alpha 14	Gna14	-1.75	0.008
1449014_at	lactamase, beta	Lactb	-1.75	0.008
1426083_a_at	B-cell translocation gene 1, anti-proliferative	Btg1	-1.75	0.008
1424489_a_at	tRNA isopentenyltransferase 1	Trit1	-1.75	0.001
1416765_s_at	mitochondria-associated colony-stimulating factor signal transduction	MGI:1913699	-1.75	0.000
1456746_a_at	Cd99 antigen-like 2	Cd99l2	-1.75	0.002
1418916_a_at	secreted phosphoprotein 2	Spp2	-1.75	0.001
1417823_at	glycine C-acetyltransferase (2-amino-3-ketobutyrate-coenzyme A ligase)	Gcat	-1.76	0.001
1451231_a_at	cullin 2	Cul2	-1.76	0.000
1448037_at	Zinc finger, CCHC domain containing 6	Zcchc6	-1.76	0.004
1419356_at	Kruppel-like factor 7 (ubiquitous)	Klf7	-1.76	0.001
1427482_a_at	carbonic anhydrase 8	Car8	-1.76	0.002
1440500_at	Mitogen activated protein kinase kinase kinase 10	Map3k10	-1.76	0.001

1448641_at	mbt domain containing 1	Mbtd1	-1.77	0.003
1441996_at	FLYWCH-type zinc finger 1	Flywch1	-1.77	0.008
1418209_a_at	profilin 2	Pfn2	-1.77	0.008
1425505_at	myosin, light polypeptide kinase	Mylk	-1.77	0.004
1448909_a_at	mitochondrial ribosomal protein L39	Mrpl39	-1.78	0.002
1448535_at	elongation protein 4 homolog (S. cerevisiae)	Elp4	-1.78	0.004
1436917_s_at	G-protein signalling modulator 1 (AGS3-like, C. elegans)	Gpsm1	-1.78	0.005
1443586_at	FIP1 like 1 (S. cerevisiae)	Fip1l1	-1.78	0.003
1441315_s_at	solute carrier family 19 (thiamine transporter), member 2	Slc19a2	-1.78	0.001
1455197_at	Rho family GTPase 1	Rnd1	-1.78	0.007
1421664_a_at	phosphoserine/threonine/tyrosine interaction protein	Styx	-1.78	0.000
1453836_a_at	monoglyceride lipase	Mgll	-1.78	0.006
1423462_at	mitogen-activated protein kinase kinase kinase 7 interacting protein 2	Map3k7ip2	-1.78	0.003
1438691_at	zinc finger, ZZ-type with EF hand domain 1 /// similar to KIAA0399 protein	Zzef1	-1.78	0.000
1448491_at	enoyl coenzyme A hydratase 1, peroxisomal	Ech1	-1.78	0.005
1444679_at	PHD finger protein 21A	Bhc80	-1.78	0.000
1438180_x_at	HS1 binding protein	Hs1bp1	-1.79	0.004
1454738_x_at	peroxisomal biogenesis factor 6	Pex6	-1.79	0.000
1455729_at	guanine nucleotide binding protein, alpha q polypeptide	Gnaq	-1.79	0.001
1434511_at	phosphorylase kinase beta	Phkb	-1.79	0.000
1460006_at	AT motif binding factor 1	Atbf1	-1.79	0.002
1454690_at	inhibitor of kappaB kinase gamma	Ikbkg	-1.79	0.000
1456087_at	nuclear factor I/A	Nfia	-1.79	0.001
1428322_a_at	NADH dehydrogenase (ubiquinone) 1 beta subcomplex, 10	Ndufb10	-1.79	0.001
1416494_at	NADH dehydrogenase (ubiquinone) Fe-S protein 5	Ndufs5	-1.80	0.001
1416159_at	nuclear receptor subfamily 2, group F, member 2	Nr2f2	-1.80	0.004
1418923_at	solute carrier family 17 (sodium phosphate), member 3	Slc17a3	-1.80	0.001
1422833_at	forkhead box A2	Foxa2	-1.80	0.004
1433745_at	triple functional domain (PTPRF interacting)	Trio	-1.80	0.002
1417373_a_at	tubulin, alpha 4	Tuba4	-1.80	0.003
1416480_a_at	hypoxia induced gene 1	MGI:1930666	-1.81	0.001
1428782_a_at	ubiquinol-cytochrome c reductase core protein 1	Uqcrc1	-1.81	0.005
1446802_at	ATX1 (antioxidant protein 1) homolog 1 (yeast)	Atox1	-1.81	0.003
1451240_a_at	glyoxalase 1	Glo1	-1.81	0.003
1439929_at	Malignant fibrous histiocytoma amplified sequence 1	Mfhas1	-1.81	0.001
1418005_at	succinate dehydrogenase complex, subunit B, iron sulfur (Ip)	Sdhb	-1.81	0.001

1424299_at	OMA1 homolog, zinc metallopeptidase (S. cerevisiae)	Oma1	-1.81	0.001
1434606_at	V-erb-b2 erythroblastic leukemia viral oncogene homolog 3 (avian)	Erbb3	-1.81	0.000
1450818_a_at	NADH dehydrogenase (ubiquinone) 1 alpha subcomplex, 7 (B14.5a)	Ndufa7	-1.81	0.001
1451642_at	kinesin family member 1B	Kif1b	-1.81	0.002
1440011_at	Exostoses (multiple) 1	Ext1	-1.81	0.010
1458715_at	Caspase 6	Casp6	-1.81	0.001
1447294_at	F-box and WD-40 domain protein 11	Fbxw11	-1.82	0.006
1460684_at	transmembrane 7 superfamily member 2	Tm7sf2	-1.82	0.002
1436157_at	cell division cycle and apoptosis regulator 1	Ccar1	-1.82	0.002
1459882_at	ASF1 anti-silencing function 1 homolog A (S. cerevisiae)	Asf1a	-1.82	0.006
1447854_s_at	histone 2, H2bb	Hist2h2bb	-1.82	0.003
1456374_x_at	eukaryotic translation initiation factor 3, subunit 8	Eif3s8	-1.82	0.002
1428598_at	TBC1 domain family, member 7	Tbc1d7	-1.82	0.002
1424282_at	PET112-like (yeast)	Pet112l	-1.82	0.005
1427472_a_at	complement component 8, beta subunit	C8b	-1.83	0.001
1455821_x_at	complement component 1, q subcomponent binding protein	C1qbp	-1.83	0.002
1433694_at	phosphodiesterase 3B, cGMP-inhibited	Pde3b	-1.83	0.003
1455911_x_at	nuclear protein 15.6	Np15	-1.84	0.001
1436827_at	gene model 944, (NCBI)	Gm944	-1.84	0.009
1426689_s_at	succinate dehydrogenase complex, subunit A, flavoprotein (Fp)	Sdha	-1.84	0.000
1416679_at	ATP-binding cassette, sub-family D (ALD), member 3	Abcd3	-1.84	0.005
1435446_a_at	choline phosphotransferase 1	Chpt1	-1.84	0.007
1434044_at	replication initiator 1	Repin1	-1.84	0.000
1416922_a_at	BCL2/adenovirus E1B 19kDa-interacting protein 3-like	Bnip3l	-1.85	0.000
1420095_s_at	zinc finger proliferation 1	Zipro1	-1.85	0.007
1425480_at	CCR4-NOT transcription complex, subunit 6-like	Cnot6l	-1.85	0.001
1448499_a_at	epoxide hydrolase 2, cytoplasmic	Ephx2	-1.85	0.003
1426358_at	TAO kinase 1	Taok1	-1.85	0.001
1451188_at	WD repeat domain 26	Wdr26	-1.85	0.000
1417784_at	amyotrophic lateral sclerosis 2 (juvenile) homolog (human)	Als2	-1.85	0.005
1446827_at	nuclear receptor coactivator 2	Ncoa2	-1.86	0.006
1447209_at	Forkhead box P1	Foxp1	-1.86	0.001
1456871_a_at	PHD finger protein 20-like 1	Phf20l1	-1.86	0.001
1450640_x_at	ATP synthase, H+ transporting, mitochondrial F1F0 complex, subunit e	Atp5k	-1.86	0.000
1455283_x_at	NADH dehydrogenase (ubiquinone) Fe-S protein 8	Ndufs8	-1.86	0.000
1437156_at	EF hand calcium binding protein 1	Efcbp1	-1.86	0.001

1422524_at	ATP-binding cassette, sub-family B (MDR/TAP), member 6	Abcb6	-1.86	0.000
1449130_at	CD1d1 antigen	Cd1d1	-1.86	0.007
1418366_at	histone 2, H2aa1 /// histone 1, H2ad /// histone 1, H2an /// histone 2, H2ab /// histone 2, H2ac /// histone	Hist2h2aa1 /// Hist	-1.87	0.002
1434642_at	dehydrogenase/reductase (SDR family) member 8	Dhrs8	-1.87	0.002
1425686_at	CASP8 and FADD-like apoptosis regulator	Cflar	-1.87	0.007
1448284_a_at	NADH dehydrogenase (ubiquinone) 1, subcomplex unknown, 1	Ndufc1	-1.87	0.001
1425365_a_at	cytochrome P450, family 2, subfamily d, polypeptide 13	Cyp2d13	-1.87	0.009
1437274_at	coatomer protein complex subunit alpha	Copa	-1.87	0.009
1422850_at	poly(A) binding protein, nuclear 1	Pabpn1	-1.88	0.004
1418446_at	solute carrier family 16 (monocarboxylic acid transporters), member 2	Slc16a2	-1.88	0.000
1419095_a_at	apolipoprotein M	Apom	-1.88	0.004
1457228_x_at	GLE1 RNA export mediator-like (yeast	Gle1l	-1.88	0.004
1446412_at	WW domain-containing oxidoreductase	Wwox	-1.88	0.009
1420420_at	hydroxyacid oxidase 1, liver	Hao1	-1.88	0.000
1447442_at	Helicase with zinc finger domain	Helz	-1.89	0.003
1441344_at	SPFH domain family, member 1	Spfh1	-1.89	0.001
1452497_a_at	nuclear factor of activated T-cells, cytoplasmic, calcineurin-dependent 3	Nfatc3	-1.89	0.001
1452915_at	protein kinase, cAMP dependent regulatory, type II alpha	Prkar2a	-1.89	0.001
1437211_x_at	ELOVL family member 5, elongation of long chain fatty acids (yeast)	Elovl5	-1.89	0.001
1419671_a_at	interleukin 17 receptor C	Il17rc	-1.89	0.000
1459843_s_at	MAD homolog 1 (Drosophila)	Smad1	-1.89	0.002
1448376_at	Werner helicase interacting protein 1	Wrnip1	-1.90	0.000
1460232_s_at	hydroxysteroid dehydrogenase-2, delta<5>-3-beta	Hsd3b2 /// Hsd3b3	-1.90	0.010
1429240_at	StAR-related lipid transfer (START) domain containing 4	Stard4	-1.90	0.007
1455025_at	progestin and adipoQ receptor family member IX	Paqr9	-1.90	0.003
1429360_at	Kruppel-like factor 3 (basic)	Klf3	-1.90	0.003
1445235_at	YTH domain family 3	Ythdf3	-1.90	0.004
1430039_at	CDK5 regulatory subunit associated protein 1-like 1	Cdkal1	-1.91	0.005
1429251_at	PR domain containing 2, with ZNF domain	Prdm2	-1.91	0.001
1455493_at	synaptic nuclear envelope 1	Syne1	-1.91	0.010
1458366_at	Additional sex combs like 2 (Drosophila)	Asxl2	-1.91	0.006
1441976_at	Ring finger protein (C3HC4 type) 19	Rnf19	-1.91	0.001
1419283_s_at	tensin	Tns	-1.92	0.000
1436755_at	inter-alpha (globulin) inhibitor H5	Itih5	-1.92	0.001
1426896_at	Zinc finger protein 191	Zfp191	-1.92	0.001
1456131_x_at	dystroglycan 1	Dag1	-1.92	0.000

1416567_s_at	ATP synthase, H+ transporting, mitochondrial F1 complex, epsilon subunit	Atp5e	-1.93	0.000
1436643_x_at	hepcidin antimicrobial peptide 2	Hamp2	-1.93	0.005
1431302_a_at	nudix (nucleoside diphosphate linked moiety X)-type motif 7	Nudt7	-1.93	0.009
1420618_at	cytoplasmic polyadenylation element binding protein 4	Cpeb4	-1.93	0.001
1442732_at	Hydroxyacyl-CA dehydrogenase/3-ketoacyl-CoA thiolase/enoyl-CoA hydratase, b subunit	Hadhb	-1.93	0.000
1451088_a_at	oxidase assembly 1-like	Oxa1l	-1.94	0.004
1455698_at	translocation protein 1	Tloc1	-1.94	0.009
1457528_at	Solute carrier family 4, sodium bicarbonate cotransporter, member 7	Slc4a7	-1.94	0.004
1416838_at	methylmalonyl-Coenzyme A mutase	Mut	-1.94	0.000
1423006_at	proviral integration site 1	Pim1	-1.94	0.008
1418024_at	NMDA receptor-regulated gene 1	Narg1	-1.94	0.003
1424276_at	sorting nexin 16	Snx16	-1.94	0.004
1422486_a_at	MAD homolog 4 (Drosophila)	Smad4	-1.94	0.005
1442632_at	Centaurin, gamma 2	Centg2	-1.95	0.002
1419301_at	frizzled homolog 4 (Drosophila)	Fzd4	-1.95	0.006
1442254_at	Diacylglycerol kinase, delta	Dgkd	-1.96	0.007
1450237_at	deoxyribonuclease II beta	Dnase2b	-1.96	0.001
1442538_at	REST corepressor 3	Rcor3	-1.96	0.000
1416750_at	opioid receptor, sigma 1	Oprs1	-1.96	0.005
1430317_at	ubiquitin-conjugating enzyme E2, J2 homolog (yeast)	Ube2j2	-1.96	0.003
1447411_at	UDP-glucose dehydrogenase	Ugdh	-1.96	0.002
1419435_at	aldehyde oxidase 1	Aox1	-1.96	0.004
1421720_a_at	deltex 2 homolog (Drosophila)	Dtx2	-1.96	0.009
1451903_at	Kynureninase (L-kynurenine hydrolase)	Kynu	-1.97	0.001
1441023_at	eukaryotic translation initiation factor 2, subunit 2 (beta)	Eif2s2	-1.97	0.003
1418645_at	histidine ammonia lyase	Hal	-1.97	0.002
1444693_at	Calcium channel, voltage-dependent, beta 2 subunit	Cacnb2	-1.97	0.003
1441852_x_at	APG16 autophagy 16-like (S. cerevisiae)	Apg16l	-1.97	0.009
1436380_at	Cdc42 binding protein kinase alpha	Cdc42bpa	-1.97	0.000
1440268_at	tripartite motif-containing 41	Trim41	-1.97	0.002
1418285_at	ephrin B1	Efnb1	-1.97	0.003
1455314_at	LIM domain containing preferred translocation partner in lipoma	Lpp	-1.98	0.001
1428076_s_at	NADH dehydrogenase (ubiquinone) 1 beta subcomplex 4	Ndufb4	-1.98	0.000
1428230_at	protein kinase C, nu	Prkcn	-1.98	0.000
1439917_at	PDZ domain containing 8	Pdzk8	-1.98	0.007
1418311_at	fructosamine 3 kinase	Fn3k	-1.98	0.001

1423043_s_at	DEAD/H (Asp-Glu-Ala-Asp/His) box polypeptide 3, X-linked /// fibroblast growth factor inducible 14	Ddx3x /// Fin14	-1.98	0.002
1428885_at	synaptogyrin 2 /// arylformamidase	Syngr2 /// Afmid	-1.99	0.009
1420022_s_at	Suppressor of zeste 12 homolog (Drosophila)	Suz12	-1.99	0.008
1427511_at	Beta-2 microglobulin	B2m	-1.99	0.002
1445952_at	BAI1-associated protein 1	Baiap1	-2.00	0.004
1415974_at	mitogen activated protein kinase kinase 2	Map2k2	-2.00	0.001
1455045_at	serine racemase	Srr	-2.01	0.005
1416832_at	solute carrier family 39 (metal ion transporter), member 8	Slc39a8	-2.01	0.001
1449168_a_at	A kinase (PRKA) anchor protein 2	Akap2	-2.01	0.005
1418627_at	glutamate-cysteine ligase , modifier subunit	Gclm	-2.01	0.000
1436697_at	Down syndrome critical region gene 3	Dscr3	-2.02	0.010
1452272_a_at	growth factor, erv1 (S. cerevisiae)-like (augmenter of liver regeneration)	Gfer	-2.03	0.001
1421769_at	LIM homeobox transcription factor 1 beta	Lmx1b	-2.03	0.003
AFFX-b-ActinMur/M1	actin, beta, cytoplasmic	Actb	-2.03	0.000
1441448_at	Pumilio 2 (Drosophila)	Pum2	-2.03	0.001
1427150_at	myeloid/lymphoid or mixed-lineage leukemia 3	Mll3	-2.04	0.000
1451438_s_at	C-type lectin domain family 2, member h	Clec2h	-2.04	0.006
1434180_at	pleckstrin homology domain containing, family C (with FERM domain) member 1	Plekhc1	-2.05	0.000
1415947_at	cellular repressor of E1A-stimulated genes 1	Creg1	-2.05	0.001
1435065_x_at	Vav2 oncogene	Vav2	-2.05	0.002
1450117_at	transcription factor 3	Tcf3	-2.05	0.003
1450733_at	bicaudal D homolog 2 (Drosophila)	Bicd2	-2.05	0.001
1418739_at	serum/glucocorticoid regulated kinase 2	Sgk2	-2.05	0.002
1418858_at	aldehyde oxidase 3	Aox3	-2.05	0.005
1451554_a_at	anterior pharynx defective 1a homolog (C. elegans)	Aph1a	-2.06	0.004
1431429_a_at	ADP-ribosylation factor-like 4	Arl4	-2.06	0.002
1435757_a_at	ubiquinol cytochrome c reductase core protein 2	Uqcrc2	-2.06	0.001
1420628_at	purine rich element binding protein A /// RIKEN cDNA 6330411E07 gene	Pura	-2.06	0.000
1431239_at	non-POU-domain-containing, octamer binding protein	Nono	-2.06	0.008
1436775_a_at	ankyrin repeat domain 17	Ankrd17	-2.07	0.004
1423416_at	SWI/SNF related, matrix associated, actin dependent regulator of chromatin, subfamily c, member 1	Smarcc1	-2.07	0.001
1458847_at	Serine/threonine kinase 25 (yeast)	Stk25	-2.07	0.001
1441305_at	Neural precursor cell expressed, developmentally down-regulated gene 4-like	Nedd4l	-2.07	0.004
1447263_at	Methionine aminopeptidase 2	Metap2	-2.07	0.004
1417301_at	frizzled homolog 6 (Drosophila)	Fzd6	-2.08	0.007
1421824_at	beta-site APP cleaving enzyme 1	Bace1	-2.08	0.000

1431263_at	protein kinase, AMP-activated, gamma 2 non-catalytic subunit	Prkag2	-2.08	0.010
1442381_at	Muskelin 1, intracellular mediator containing kelch motifs	Mkln1	-2.08	0.004
1458056_at	splicing factor, arginine/serine-rich 12	Sfrs12	-2.08	0.008
1435836_at	Pyruvate dehydrogenase kinase, isoenzyme 1	Pdk1	-2.08	0.002
1429418_at	CDC14 cell division cycle 14 homolog B (S. cerevisiae)	Cdc14b	-2.08	0.002
1456686_at	Zinc finger homeobox 1a	Zfhx1a	-2.09	0.002
1444886_at	Amyloid beta precursor protein (cytoplasmic tail) binding protein 2	Appbp2	-2.09	0.000
1441546_at	Membrane protein, palmitoylated 6 (MAGUK p55 subfamily member 6)	Mpp6	-2.09	0.008
1460323_at	threonyl-tRNA synthetase	Tars	-2.09	0.002
1437667_a_at	BTB and CNC homology 2	Bach2	-2.09	0.001
1453571_at	DEP domain containing 6	Depdc6	-2.10	0.000
1456610_at	jumonji domain containing 3	Jmjd3	-2.10	0.004
1431722_a_at	arylformamidase	Afmid	-2.10	0.005
1457500_at	DEP domain containing 5	Depdc5	-2.11	0.002
1445827_at	Protein kinase C binding protein 1	Prkcbp1	-2.11	0.005
1457712_at	chromodomain helicase DNA binding protein 8	Chd8	-2.11	0.001
1429488_at	zinc finger, DHHC domain containing 21	Zdhhc21	-2.11	0.002
1433574_at	cell division cycle 37 homolog (S. cerevisiae)-like 1	Cdc37l1	-2.11	0.000
1426857_a_at	hydroxysteroid dehydrogenase like 2	Hsdl2	-2.12	0.000
1449741_at	Desmoglein 2	Dsg2	-2.12	0.001
1436363_a_at	nuclear factor I/X	Nfix	-2.13	0.002
1418547_at	tissue factor pathway inhibitor 2	Tfpi2	-2.13	0.001
1440383_at	DNA cross-link repair 1B, PSO2 homolog (S. cerevisiae)	Dclre1b	-2.13	0.007
1418519_at	aminoadipate aminotransferase	Aadat	-2.13	0.005
1425823_at	complement component factor h	Cfh	-2.13	0.000
1459746_at	R3H domain (binds single-stranded nucleic acids)	R3hdm	-2.13	0.005
1449442_at	peroxisomal biogenesis factor 11a	Pex11a	-2.13	0.001
1459329_at	Tetratricopeptide repeat domain 7	Ttc7	-2.13	0.000
1441604_at	Esterase D/formylglutathione hydrolase	Esd	-2.15	0.002
1416049_at	glycine decarboxylase	Gldc	-2.15	0.004
1426756_at	UDP-N-acetyl-alpha-D-galactosamine:polypeptide N-acetylgalactosaminyltransferase 2	Galnt2	-2.16	0.001
1418352_at	hydroxysteroid (17-beta) dehydrogenase 2	Hsd17b2	-2.16	0.003
1456505_at	Braf transforming gene	Braf	-2.16	0.006
1442697_at	Importin 11	Ipo11	-2.17	0.003
1420952_at	Son cell proliferation protein	Son	-2.17	0.000
1442901_at	Tax1 (human T-cell leukemia virus type I) binding protein 1	Tax1bp1	-2.18	0.001

1429067_at	calpain, small subunit 2	Capns2	-2.18	0.002
1419867_a_at	ankyrin repeat and KH domain containing 1	Ankhd1	-2.19	0.003
1454930_at	leucine rich repeat containing 35	Lrrc35	-2.20	0.000
1437164_x_at	ATP synthase, H+ transporting, mitochondrial F1 complex, O subunit	Atp5o /// LOC4326	-2.21	0.000
1433757_a_at	nischarin	Nisch	-2.21	0.007
AFFX-18SRNAMur/X	mannosidase 2, alpha B2	Man2b2	-2.21	0.001
1431817_at	alcohol dehydrogenase 6 (class V), pseudogene 1	Adh6-ps1	-2.21	0.007
1458604_at	Rho/rac guanine nucleotide exchange factor (GEF) 18	Arhgef18	-2.22	0.005
1447071_at	Transcription factor 7-like 2, T-cell specific, HMG-box	Tcf7l2	-2.22	0.005
1460602_at	Deleted in liver cancer 1	Dlc1	-2.22	0.000
1416275_at	solute carrier family 26, member 6	Slc26a6	-2.22	0.002
1451020_at	glycogen synthase kinase 3 beta	Gsk3b	-2.23	0.001
1438130_at	TAF15 RNA polymerase II, TATA box binding protein (TBP)-associated factor	Taf15	-2.23	0.005
1417071_s_at	cytochrome P450, family 4, subfamily v, polypeptide 3	Cyp4v3	-2.23	0.001
1430037_at	sorting nexin family member 27	Snx27	-2.24	0.002
1439638_at	Erbb2 interacting protein	Erbb2ip	-2.24	0.001
1437210_a_at	bromodomain containing 2	Brd2	-2.24	0.001
1419081_at	autophagy 10-like (S. cerevisiae)	Apg10l	-2.24	0.001
1457515_at	Homeodomain interacting protein kinase 1	Hipk1	-2.24	0.010
1455129_at	Metadherin	Mtdh	-2.24	0.000
1441397_at	Par-3 (partitioning defective 3) homolog (C. elegans)	Pard3	-2.25	0.001
1416762_at	S100 calcium binding protein A10 (calpactin)	S100a10	-2.25	0.004
1448179_at	upregulated during skeletal muscle growth 5	Usmg5	-2.26	0.005
1452973_at	protein phosphatase 1K (PP2C domain containing)	Ppm1k	-2.26	0.000
1438505_s_at	ethanol induced 2	Etohi2	-2.26	0.010
1442775_at	Suppression of tumorigenicity 13	St13	-2.27	0.004
1460328_at	bromodomain containing 3	Brd3	-2.28	0.002
1452050_at	calcium/calmodulin-dependent protein kinase ID	Camk1d	-2.28	0.002
1449299_at	low density lipoprotein receptor-related protein 5	Lrp5	-2.28	0.000
1429487_at	protein phosphatase 1, regulatory (inhibitor) subunit 12A	Ppp1r12a	-2.28	0.009
1446007_at	Bone morphogenetic protein 1	Bmp1	-2.29	0.003
1458508_at	matrin 3	Matr3	-2.30	0.001
1423202_a_at	nuclear receptor co-repressor 1	Ncor1	-2.30	0.009
1445729_at	sirtuin 3 (silent mating type information regulation 2, homolog) 3 (S. cerevisiae)	Sirt3	-2.30	0.002
1455293_at	gene model 185, (NCBI)	Gm185	-2.30	0.002
1456927_at	microtubule associated serine/threonine kinase 2	Mast2	-2.31	0.009

1457930_at	Jumonji, AT rich interactive domain 1C (Rbp2 like)	Jarid1c	-2.31	0.001
1438583_at	Endoplasmic reticulum (ER) to nucleus signalling 1	Ern1	-2.33	0.000
1457434_s_at	protein tyrosine phosphatase-like (proline instead of catalytic arginine), member a	Ptpla	-2.33	0.003
1448934_at	NADH dehydrogenase (ubiquinone) 1 alpha subcomplex 10	Ndufa10	-2.34	0.001
1460316_at	Acyl-CoA synthetase long-chain family member 1	Acsl1	-2.34	0.001
1419510_at	esterase 22	Es22	-2.35	0.008
1457578_at	Peroxisome proliferative activated receptor, gamma, coactivator 1 beta	Ppargc1b	-2.35	0.008
1450715_at	cytochrome P450, family 1, subfamily a, polypeptide 2	Cyp1a2	-2.36	0.000
1423526_at	AT rich interactive domain 3B (Bright like)	Arid3b	-2.37	0.008
1456974_at	one cut domain, family member 1	Onecut1	-2.37	0.008
1429779_at	eukaryotic translation initiation factor 2C, 4	Eif2c4	-2.37	0.001
1440622_at	SEC15-like 2 (S. cerevisiae)	Sec15l2	-2.37	0.007
1423120_at	insulin degrading enzyme	Ide	-2.38	0.002
1441181_at	RAR-related orphan receptor alpha	Rora	-2.39	0.005
1421754_at	cDNA sequence AY036118	AY036118	-2.39	0.003
1428167_a_at	myelin protein zero-like 1	Mpzl1	-2.39	0.007
1456341_a_at	basic transcription element binding protein 1	Bteb1	-2.40	0.000
1426777_a_at	Wiskott-Aldrich syndrome-like (human)	Wasl	-2.40	0.000
1440790_x_at	Expressed sequence AL024069	AL024069	-2.40	0.002
1438571_at	Budding uninhibited by benzimidazoles 1 homolog (S. cerevisiae)	Bub1	-2.42	0.008
1421147_at	telomeric repeat binding factor 2	Terf2	-2.42	0.001
1439479_at	lactase	Lct	-2.42	0.003
1417865_at	tumor necrosis factor, alpha-induced protein 1 (endothelial)	Tnfaip1	-2.42	0.003
1443214_at	G protein-coupled receptor 155	Gpr155	-2.45	0.008
1448005_at	SAM and SH3 domain containing 1	Sash1	-2.46	0.003
1446462_at	Histidyl tRNA synthetase 2	Hars2	-2.47	0.001
1417860_a_at	spondin 2, extracellular matrix protein	Spon2	-2.47	0.002
1424245_at	carboxylesterase 2	Ces2	-2.48	0.005
1427564_at	diaphanous homolog 2 (Drosophila)	Diap2	-2.48	0.001
1449464_at	potassium voltage-gated channel, subfamily Q, member 1	Kcnq1	-2.49	0.002
1426545_at	trinucleotide repeat containing 6b	Tnrc6b	-2.49	0.002
1417880_at	glucose-6-phosphatase, catalytic	G6pc	-2.49	0.001
1459860_x_at	tripartite motif protein 2	Trim2	-2.50	0.000
1455497_at	leukocyte receptor cluster (LRC) member 9	Leng9	-2.50	0.004
1416501_at	3-phosphoinositide dependent protein kinase-1	Pdpk1	-2.50	0.008
1441573_at	Sex comb on midleg homolog 1	Scmh1	-2.50	0.001

1419390_at	phosphodiesterase 10A	Pde10a	-2.51	0.000
1431028_a_at	pantothenate kinase 1	Pank1	-2.52	0.000
1439260_a_at	ectonucleotide pyrophosphatase/phosphodiesterase 3	Enpp3	-2.52	0.003
1446624_at	Fukuyama type congenital muscular dystrophy homolog (human)	Fcmd	-2.52	0.004
1445188_at	Gephyrin	Gphn	-2.53	0.003
1436257_at	synovial sarcoma translocation, Chromosome 18	Ss18	-2.53	0.006
1436753_at	aarF domain containing kinase 5	Adck5	-2.54	0.000
1451458_at	transmembrane protein 2	Tmem2	-2.54	0.001
1420451_at	amiloride-sensitive cation channel 5, intestinal	Accn5	-2.54	0.004
1427377_x_at	hydroxysteroid dehydrogenase-3, delta<5>-3-beta	Hsd3b3	-2.54	0.003
1418659_at	circadian locomoter output cycles kaput	Clock	-2.55	0.000
1441460_at	FGFR1 oncogene partner 2	Fgfr1op2	-2.56	0.002
1417580_s_at	selenium binding protein 1	Selenbp1	-2.57	0.000
1427371_at	ATP-binding cassette, sub-family A (ABC1), member 8a	Abca8a	-2.57	0.000
1419349_a_at	cytochrome P450, family 2, subfamily d, polypeptide 9	Cyp2d9	-2.58	0.006
1425948_a_at	solute carrier family 25, member 30	Slc25a30	-2.58	0.003
1457582_at	Ubiquitously transcribed tetratricopeptide repeat gene, Y chromosome	Uty	-2.58	0.004
1458279_at	Forkhead box O3a	Foxo3	-2.59	0.001
1439173_at	hook homolog 1 (Drosophila)	Hook1	-2.59	0.002
1444873_at	Huntingtin interacting protein 1	Hip1	-2.59	0.003
1458520_at	Homeodomain interacting protein kinase 2	Hipk2	-2.59	0.007
1422416_s_at	pre-B lymphocyte gene 1 /// pre-B lymphocyte gene 2	Vpreb1	-2.60	0.001
1458043_at	BTB (POZ) domain containing 9	Btbd9	-2.60	0.005
1447094_at	Yamaguchi sarcoma viral (v-yes) oncogene homolog 1	Yes	-2.61	0.006
1451139_at	solute carrier family 39 (zinc transporter), member 4	Slc39a4	-2.61	0.004
1437821_at	Diaphanous homolog 1 (Drosophila)	Diap1	-2.61	0.009
1430546_at	crystallin, zeta (quinone reductase)-like 1	Cryzl1	-2.61	0.000
1438401_at	ubinuclein 1	Ubn1	-2.61	0.000
1437303_at	interleukin 6 signal transducer	Il6st	-2.63	0.002
1440579_at	Mindbomb homolog 1 (Drosophila)	Mib1	-2.64	0.001
1418562_at	splicing factor 3b, subunit 1	Sf3b1	-2.65	0.001
1451538_at	SRY-box containing gene 9	Sox9	-2.65	0.003
1444562_at	Ubiquitin protein ligase E3C	Ube3c	-2.66	0.004
1444651_at	Mitogen activated protein kinase kinase 4	Map2k4	-2.67	0.000
1446057_at	Rearranged L-myc fusion sequence	Rlf	-2.67	0.002
1437322_at	RNA binding motif protein 4	Rbm4	-2.67	0.001

1450966_at	carnitine O-octanoyltransferase	Crot	-2.67	0.000
1446713_at	Tial1 cytotoxic granule-associated RNA binding protein-like 1	Tial1	-2.68	0.003
1427143_at	jumonji, AT rich interactive domain 1B (Rbp2 like)	Jarid1b	-2.68	0.000
1435520_at	Musashi homolog 2 (Drosophila)	Msi2h	-2.69	0.008
1452058_a_at	ring finger protein 11	Rnf11	-2.69	0.000
1440966_at	Membrane-associated ring finger (C3HC4) 7	Axot	-2.69	0.001
1424398_at	DEAH (Asp-Glu-Ala-His) box polypeptide 36	Dhx36	-2.70	0.003
1438121_at	SEC24 related gene family, member A (S. cerevisiae)	Sec24a	-2.70	0.003
1416833_at	kidney expressed gene 1	Keg1	-2.71	0.007
1427480_at	liver-expressed antimicrobial peptide 2	MGI:2672795	-2.73	0.003
1441548_at	FERM domain containing 4B	Frmd4b	-2.73	0.006
1457357_at	Tousled-like kinase 2 (Arabidopsis)	Tlk2	-2.73	0.003
1457036_at	Upstream transcription factor 2	Usf2	-2.74	0.004
1459045_at	Golgi phosphoprotein 4	Golph4	-2.74	0.002
1460436_at	N-deacetylase/N-sulfotransferase (heparan glucosaminyl) 1	Ndst1	-2.75	0.000
1444647_at	phospholipase A2, activating protein	Plaa	-2.76	0.003
1426559_at	sno, strawberry notch homolog 1 (Drosophila)	Sbno1	-2.78	0.000
1432286_at	polymerase (DNA-directed), epsilon 4 (p12 subunit)	Pole4	-2.79	0.006
1443803_x_at	homeo box A5	Hoxa5	-2.79	0.007
1429944_at	trichohyalin-like 1	Thhl1	-2.80	0.001
1441440_at	APG4 (ATG4) autophagy-related homolog C (S. cerevisiae)	Apg4c	-2.82	0.008
1420947_at	alpha thalassemia/mental retardation syndrome X-linked homolog (human)	Atrx	-2.83	0.002
1447038_x_at	pleckstrin homology domain interacting protein	Phip	-2.83	0.000
1450202_at	glutamate receptor, ionotropic, NMDA1 (zeta 1)	Grin1	-2.84	0.002
1458832_at	Growth hormone receptor	Ghr	-2.84	0.001
1425476_at	procollagen, type IV, alpha 5	Col4a5	-2.84	0.006
1427676_a_at	Glutamate receptor, ionotropic, kainate 1	Grik1	-2.85	0.001
1435775_at	TPA regulated locus	Tparl	-2.86	0.000
1419322_at	FYVE, RhoGEF and PH domain containing 6	Fgd6	-2.88	0.000
1428634_at	TWIST neighbor	Twistnb	-2.88	0.002
1454525_at	selenoprotein	MGI:1927947	-2.90	0.004
1441955_s_at	polyadenylate binding protein-interacting protein 1	Paip1	-2.90	0.004
1457534_at	Ubiquitin associated protein 2-like	Ubap2l	-2.90	0.010
1449337_at	tryptophan 2,3-dioxygenase	Tdo2	-2.90	0.000
1440488_at	Protein tyrosine phosphatase, non-receptor type 4	Ptpn4	-2.91	0.001
1444454_at	Retinoblastoma 1	Rb1	-2.91	0.000

1442198_at	NIMA (never in mitosis gene a)-related expressed kinase 7	Nek7	-2.92	0.005
1419112_at	nemo like kinase	Nlk	-2.92	0.009
1445596_at	Adenosine deaminase, RNA-specific	Adar	-2.92	0.002
1440131_at	Poliovirus receptor-related 1	Pvrl1	-2.92	0.001
1443037_at	Stromal cell derived factor receptor 1	Sdfr1	-2.94	0.005
1451577_at	zinc finger and BTB domain containing 20	Zbtb20	-2.94	0.001
1459627_at	Sterol-C4-methyl oxidase-like	Sc4mol	-2.95	0.004
1441026_at	poly (ADP-ribose) polymerase family, member 4	Parp4	-2.95	0.004
1438084_at	A disintegrin and metalloprotease domain 23	Adam23	-2.97	0.000
1450725_s_at	carbonic anhydrase 14	Car14	-2.97	0.000
1418213_at	keratin complex 1, acidic, gene 23	Krt1-23	-2.97	0.005
1417896_at	tight junction protein 3	Tjp3	-2.97	0.008
1443516_at	Spinocerebellar ataxia 2 homolog (human)	Sca2	-2.98	0.003
1454649_at	Steroid 5 alpha-reductase 1	Srd5a1	-2.98	0.001
1430255_at	Kruppel-like factor 5	Klf5	-2.99	0.001
1449051_at	peroxisome proliferator activated receptor alpha	Ppara	-2.99	0.000
1443860_at	protein tyrosine phosphatase, receptor type, D	Ptprd	-2.99	0.000
1433691_at	protein phosphatase 1, regulatory (inhibitor) subunit 3C	Ppp1r3c	-3.00	0.000
1420985_at	ash1 (absent, small, or homeotic)-like (Drosophila)	Ash1l	-3.01	0.000
1451375_at	ets homologous factor	Ehf	-3.01	0.004
1429463_at	protein kinase, AMP-activated, alpha 2 catalytic subunit	Prkaa2	-3.02	0.003
1445244_at	Beta-amyloid binding protein precursor	Bbp	-3.02	0.003
1417982_at	insulin induced gene 2	Insig2	-3.03	0.001
1416488_at	cyclin G2	Ccng2	-3.03	0.009
1428372_at	suppression of tumorigenicity 5	St5	-3.05	0.000
1440104_at	RAN binding protein 2	Ranbp2	-3.05	0.000
1440517_x_at	cytidine monophospho-N-acetylneuraminic acid hydroxylase	Cmah	-3.06	0.004
1460280_at	GRB2-related adaptor protein 2	Grap2	-3.08	0.007
1439800_at	Restin (Reed-Steinberg cell-expressed intermediate filament-associated protein)	Rsn	-3.09	0.000
1456054_a_at	pumilio 1 (Drosophila)	Pum1	-3.10	0.000
1441802_at	Sprouty protein with EVH-1 domain 2, related sequence	Spred2	-3.10	0.001
1456429_at	mucosa associated lymphoid tissue lymphoma translocation gene 1	Malt1	-3.10	0.000
1456449_at	Retinitis pigmentosa GTPase regulator interacting protein 1	Supt16h	-3.11	0.007
1437473_at	avian musculoaponeurotic fibrosarcoma (v-maf) AS42 oncogene homolog	Maf	-3.11	0.001
1441375_at	Leucine-rich repeats and immunoglobulin-like domains 1	Lrig1	-3.11	0.001
1449069_at	zinc finger protein 148	Zfp148	-3.12	0.010

1437877_at	tetratricopeptide repeat domain 14	Ttc14	-3.13	0.003
1417203_at	ethylmalonic encephalopathy 1	Ethe1	-3.13	0.000
1417599_at	CD276 antigen	Cd276	-3.13	0.002
1436181_at	development and differentiation enhancing factor 2	Ddef2	-3.14	0.000
1456216_at	Casein kinase 1, alpha 1	Csnk1a1	-3.14	0.000
1448803_at	golgi autoantigen, golgin subfamily a, 4	Golga4	-3.16	0.003
1457794_at	Wolf-Hirschhorn syndrome candidate 1-like 1 (human)	Whsc1l1	-3.19	0.001
1425362_at	HIV-1 Rev binding protein-like	Hrbl	-3.20	0.000
1448595_a_at	reduced expression 3	Rex3	-3.20	0.009
1443048_at	F-box only protein 21	Fbxo21	-3.20	0.001
1437941_at	phosphorylase kinase alpha 2	Phka2	-3.20	0.001
1441992_at	RAB14, member RAS oncogene family	Rab14	-3.26	0.004
1457562_at	ribosomal protein S6 kinase, polypeptide 1	Rps6kb1	-3.26	0.005
1442222_at	Interferon (alpha and beta) receptor 1	Ifnar1	-3.26	0.002
1459635_at	Discs, large homolog 1 (Drosophila)	Dlgh1	-3.27	0.006
1441349_at	SEC24 related gene family, member B (S. cerevisiae)	Sec24b	-3.27	0.002
1445695_at	Spinocerebellar ataxia 1 homolog (human)	Sca1	-3.27	0.008
1447325_at	Expressed sequence AU017263	Ches1	-3.29	0.001
1442102_at	Aconitase 2, mitochondrial	Aco2	-3.30	0.000
1458007_at	myosin IB	Myo1b	-3.31	0.007
1443366_at	N-ethylmaleimide sensitive fusion protein attachment protein gamma /// similar to Gamma-soluble NSF a	Napg	-3.32	0.002
1452722_a_at	cullin 5	Cul5	-3.33	0.008
1436931_at	regulatory factor X, 4 (influences HLA class II expression)	Rfx4	-3.33	0.009
1425640_at	homolog of human MLLT2 unidentified gene	Mllt2h	-3.33	0.007
1426318_at	serine (or cysteine) proteinase inhibitor, clade B, member 1b	Serpinb1b	-3.33	0.003
1445676_at	Potassium intermediate/small conductance calcium-activated channel, subfamily N, member 2	Kcnn2	-3.36	0.002
1444503_at	Glioblastoma amplified sequence	Gbas	-3.37	0.005
1459791_at	DnaJ (Hsp40) homolog, subfamily C, member 1	Dnajc1	-3.37	0.005
1459530_at	Hydroxysteroid (17-beta) dehydrogenase 12	Hsd17b12	-3.39	0.000
1437543_at	far upstream element (FUSE) binding protein 1	Fubp1	-3.41	0.004
1418189_s_at	receptor (calcitonin) activity modifying protein 2	Ramp2	-3.42	0.003
1441348_at	Zinc finger protein 422, related sequence 1	Zfp422-rs1	-3.42	0.000
1437250_at	whn-dependent transcript 2	MGI:2151839	-3.44	0.003
1442885_at	SREBP cleavage activating protein	Scap	-3.45	0.009
1438420_at	RNA-binding region (RNP1, RRM) containing 2	Rnpc2	-3.47	0.007
1440641_at	Vacuolar protein sorting 29 (S. pombe)	Vps29	-3.53	0.000

1418180_at	Trans-acting transcription factor 1	Sp1	-3.55	0.004
1437179_at	Rap1 interacting factor 1 homolog (yeast)	Rif1	-3.57	0.009
1444037_at	Lectin, mannose-binding, 1	Lman1	-3.59	0.001
1438451_at	Rho GTPase-activating protein	MGI:2450166	-3.59	0.004
1423447_at	caseinolytic protease X (E.coli)	Clpx	-3.62	0.009
1438039_at	HECT domain containing 1	Hectd1	-3.62	0.000
1441823_at	Retinoic acid induced 17	Rai17	-3.63	0.005
1432181_s_at	endothelial cell growth factor 1 (platelet-derived)	Ecgf1	-3.64	0.000
1457432_at	prospero-related homeobox 1	Prox1	-3.65	0.000
1427486_at	protein tyrosine phosphatase, receptor type, B	Ptprb	-3.66	0.002
1446068_at	adenosine kinase	Adk	-3.67	0.003
1427862_at	ADP-ribosylation factor-like 6 interacting protein 3	Arl6ip3	-3.74	0.000
1449309_at	cytochrome P450, family 8, subfamily b, polypeptide 1	Cyp8b1	-3.76	0.000
1420950_at	zinc and ring finger 1	Znrf1	-3.78	0.002
1425869_a_at	presenilin 2	Psen2	-3.80	0.003
1427310_at	fetal Alzheimer antigen	Falz	-3.86	0.005
1456260_at	Retinoblastoma binding protein 4	Rbbp4	-3.89	0.007
1438268_at	membrane associated DNA binding protein	Mnab	-3.94	0.001
1441704_at	Pleckstrin homology domain containing, family A member 5	Plekha5	-3.98	0.008
1441030_at	Retinoic acid induced 14	Rai14	-4.00	0.007
1418129_at	24-dehydrocholesterol reductase	Dhcr24	-4.12	0.006
1456112_at	translocated promoter region	Tpr	-4.13	0.003
1453487_at	dihydrodiol dehydrogenase (dimeric)	Dhdh	-4.14	0.002
1417513_at	ecotropic viral integration site 5	Evi5	-4.14	0.004
1439196_at	hook homolog 3 (Drosophila)	Hook3	-4.16	0.000
1437572_at	DCP1 decapping enzyme homolog b (S. cerevisiae)	Dcp1b	-4.18	0.001
1444295_at	neogenin	Neo1	-4.18	0.000
1453313_at	sestrin 3	Sesn3	-4.23	0.000
1421907_at	peroxisome proliferator activated receptor binding protein	Pparbp	-4.23	0.003
1440373_at	ELAV (embryonic lethal, abnormal vision, Drosophila)-like 1 (Hu antigen R)	Elavl1	-4.32	0.003
1450264_a_at	choline kinase alpha	Chka	-4.34	0.003
1447099_at	Rho GTPase-activating protein	Grit	-4.38	0.003
1447527_at	Interferon regulatory factor 2	Irf2	-4.42	0.000
1452975_at	alanine-glyoxylate aminotransferase 2-like 1	Agxt2l1	-4.43	0.000
1449311_at	BTB and CNC homology 1	Bach1	-4.46	0.001
1444438_at	calcium and integrin binding family member 3	Cib3	-4.49	0.003

1436593_at	Unknown (protein for MGC:58818)	RP23-96I9.2	-4.50	0.009
1445032_at	Death associated protein kinase 1	Dapk1	-4.53	0.001
1442667_at	Cell division cycle 40 homolog (yeast)	Cdc40	-4.60	0.009
1439197_at	phosphatidylinositol 4-kinase, catalytic, beta polypeptide	Pik4cb	-4.62	0.003
1446316_at	Lipin 2	Lpin2	-4.62	0.006
1456151_at	Zinc finger protein 358	Zfp358	-4.63	0.004
1448818_at	wingless-related MMTV integration site 5A	Wnt5a	-4.64	0.002
1451906_at	UBX domain containing 3	Ubxd3	-4.64	0.002
1420379_at	solute carrier organic anion transporter family, member 1a1	Slco1a1	-4.71	0.003
1448261_at	cadherin 1	Cdh1	-4.73	0.000
1438688_at	Serine/arginine repetitive matrix 2	Srrm2	-4.75	0.000
1452125_at	thyroid hormone receptor associated protein 3	Thrap3	-4.75	0.009
1424296_at	glutamate-cysteine ligase, catalytic subunit	Gclc	-4.76	0.010
1444194_at	Myeloid/lymphoid or mixed lineage-leukemia translocation to 10 homolog (Drosophila)	Mllt10	-4.81	0.000
1419038_a_at	casein kinase II, alpha 1 polypeptide	Csnk2a1	-4.92	0.004
1447382_at	Phosphatidylinositol glycan, class T	Pigt	-4.93	0.009
1436767_at	LUC7-like 2 (S. cerevisiae)	Luc7l2	-4.93	0.008
1442986_at	ELK3, member of ETS oncogene family	Elk3	-4.98	0.003
1425099_a_at	aryl hydrocarbon receptor nuclear translocator-like	Arntl	-5.07	0.007
1436985_at	zinc finger protein 644	Zfp644	-5.17	0.004
1416707_a_at	polyamine-modulated factor 1	Pmf1	-5.19	0.007
1429432_at	BAT2 domain containing 1	Bat2d	-5.31	0.002
1417066_at	chaperone, ABC1 activity of bc1 complex like (S. pombe)	Cabc1	-5.39	0.000
1442295_at	Actin related protein 2/3 complex, subunit 2	Arpc2	-5.40	0.009
1438892_at	Quaking	Qk	-5.56	0.005
1445042_at	Aminoadipate-semialdehyde synthase	Aass	-5.59	0.004
1436983_at	CREB binding protein	Crebbp	-5.62	0.003
1441926_x_at	transmembrane inner ear	Tmie	-5.72	0.009
1443003_at	Atpase, class VI, type 11C	Atp11c	-5.88	0.008
1420531_at	hydroxysteroid dehydrogenase-5, delta<5>-3-beta	Hsd3b5	-5.99	0.000
1439108_at	myeloid/lymphoid or mixed-lineage leukemia 5	Mll5	-6.26	0.004
1430309_at	Nipped-B homolog (Drosophila)	Nipbl	-6.37	0.001
1446278_at	Ubiquitously transcribed tetratricopeptide repeat gene, X chromosome	Utx	-6.50	0.001
1440771_at	zinc finger with KRAB and SCAN domains 1	Zkscan1	-9.31	0.009
1441413_at	Catalase	Cat	-10.46	0.001
1420722_at	elongation of very long chain fatty acids (FEN1/Elo2, SUR4/Elo3, yeast)-like 3	Elovl3	-16.92	0.000
