# Supplementary material for: Impaired Genome Maintenance Suppresses the Growth Hormone–Insulin-Like Growth Factor 1 Axis in Mice with Cockayne Syndrome
Source: PLoS Biol. 2006 Dec 12;5(1):e2. doi: 10.1371/journal.pbio.0050002 (PMC1698505; doi:10.1371/journal.pbio.0050002)
Supplement: Supplementary file 5 [file pbio.0050002.st005.pdf]

Supplementary Table S5

Extensive overview of significant expression changes in 16-week old wild-type mice compared to 8-week-old mice, FC: fold change, p: p-value.

			16W	
**Code**	**Title**	**Symbol**	**FC**	**p**
1449526_a_at	glycerophosphodiester phosphodiesterase domain containing 3	Gdpd3	58.79	0.000
1435135_at	arylacetamide deacetylase-like 1	Aadacl1	24.76	0.001
1418148_at	abhydrolase domain containing 1	Abhd1	8.67	0.000
1446205_at	Nuclear transcription factor-Y gamma	Nfyc	8.27	0.001
1435188_at	similar to hypothetical protein BC017397	LOC433627	8.26	0.000
1455282_x_at	aminolevulinic acid synthase 1	Alas1	7.93	0.000
1442364_at	Mitogen activated protein kinase 14	Mapk14	7.71	0.001
1417169_at	ubiquitin specific protease 2	Usp2	7.27	0.000
1425019_at	UBX domain containing 4	Ubxd4	7.20	0.000
1421056_at	deoxyribonuclease 1-like 3	Dnase1l3	7.04	0.001
1460662_at	period homolog 3 (Drosophila)	Per3	6.89	0.001
1417714_x_at	hemoglobin alpha, adult chain 1	Hba-a1	6.30	0.001
1438211_s_at	D site albumin promoter binding protein	Dbp	6.16	0.000
1455886_at	Casitas B-lineage lymphoma	Cbl	6.13	0.000
1452677_at	polyribonucleotide nucleotidyltransferase 1	Pnpt1	6.09	0.005
1456590_x_at	aldo-keto reductase family 1, member B3 (aldose reductase)	Akr1b3	5.87	0.000
1417462_at	CAP, adenylate cyclase-associated protein 1 (yeast)	Cap1	5.58	0.006
1426366_at	eukaryotic translation initiation factor 2C, 2	Eif2c2	5.40	0.004
1420947_at	alpha thalassemia/mental retardation syndrome X-linked homolog (human)	Atrx	5.36	0.002
1429466_s_at	anterior pharynx defective 1c homolog (C. elegans)	Aph1c	5.16	0.000
1443923_at	A kinase (PRKA) anchor protein 13	Akap13	5.07	0.000
1449876_at	protein kinase, cGMP-dependent, type I	Prkg1	4.99	0.000
1457877_at	Estrogen receptor 1 (alpha)	Esr1	4.76	0.002
1445042_at	Aminoadipate-semialdehyde synthase	Aass	4.65	0.000
1440651_at	Dual specificity phosphatase 16	Dusp16	4.60	0.000
1437082_at	A kinase (PRKA) anchor protein (yotiao) 9	Akap9	4.56	0.000
1458832_at	Growth hormone receptor	Ghr	4.54	0.000
1418989_at	cathepsin E	Ctse	4.51	0.002
1446284_at	Metastasis suppressor 1	Mtss1	4.44	0.002
1436746_at	protein kinase, lysine deficient 1	Prkwnk1	4.38	0.003

1420342_at	Ganglioside-induced differentiation-associated-protein 10	Gdap10	4.34	0.000
1421704_a_at	phosphatidylinositol 3-kinase, C2 domain containing, gamma polypeptide	Pik3c2g	4.33	0.000
1430309_at	Nipped-B homolog (Drosophila)	Nipbl	4.31	0.000
1452816_at	myeloid leukemia factor 1 interacting protein	Mlf1ip	4.30	0.008
1442058_s_at	proteasome (prosome, macropain) 26S subunit, ATPase 3, interacting protein	Psmc3ip	4.21	0.002
1448975_s_at	renin 1 structural /// renin 2 tandem duplication of Ren1	Ren1 /// Ren2	4.20	0.002
1453913_a_at	transporter 2, ATP-binding cassette, sub-family B (MDR/TAP)	Tap2	3.92	0.000
1447761_x_at	superoxide dismutase 1, soluble	Sod1	3.91	0.008
1449311_at	BTB and CNC homology 1	Bach1	3.70	0.007
1452456_at	nuclear receptor interacting protein 2	Nrip2	3.65	0.010
1458699_at	CCR4 carbon catabolite repression 4-like (S. cerevisiae)	Ccrn4l	3.64	0.006
1443675_at	Enhancer of polycomb homolog 1 (Drosophila)	Epc1	3.61	0.009
1440972_at	nuclear receptor-binding SET-domain protein 1	Nsd1	3.61	0.000
1437221_at	ribonucleotide reductase M2 B (TP53 inducible)	Rrm2b	3.61	0.003
1419717_at	semaphorin 3E	Sema3e	3.58	0.000
1453189_at	ubiquitin-conjugating enzyme E2I	Ube2i	3.58	0.002
1423249_at	natural killer tumor recognition sequence	Nktr	3.57	0.008
1455057_at	Guanine monphosphate synthetase	Gmps	3.50	0.001
1444166_at	thyroid hormone responsive SPOT14 homolog (Rattus)	Thrsp	3.50	0.001
1449365_at	endothelial differentiation, sphingolipid G-protein-coupled receptor, 8	Edg8	3.49	0.000
1457123_at	neuregulin 4	Nrg4	3.48	0.001
1448523_at	nephronophthisis 1 (juvenile) homolog (human)	Nphp1	3.45	0.006
1437884_at	ADP-ribosylation factor-like 8	Arl8	3.44	0.000
1438439_at	G protein-coupled receptor 171	Gpr171	3.42	0.006
1424504_at	RAB22A, member RAS oncogene family	Rab22a	3.39	0.010
1439109_at	similar to CTCL tumor antigen se57-1	MGC54896	3.38	0.007
1425239_at	open reading frame 21	ORF21	3.38	0.001
1425795_a_at	mitogen activated protein kinase kinase kinase 7	Map3k7	3.36	0.000
1423152_at	vesicle-associated membrane protein, associated protein B and C	Vapb	3.34	0.000
1442682_at	Wee 1 homolog (S. pombe)	Wee1	3.34	0.004
1446904_at	Rho guanine nucleotide exchange factor (GEF) 11	Arhgef11	3.34	0.002
1425687_at	CASP8 and FADD-like apoptosis regulator	Cflar	3.31	0.000
1450109_s_at	ATP-binding cassette, sub-family C (CFTR/MRP), member 2	Abcc2	3.30	0.008
1456764_at	solute carrier family 35, member F3	Slc35f3	3.30	0.002
1435287_at	adducin 2 (beta)	Add2	3.30	0.002
1416490_at	transmembrane emp24 protein transport domain containing 6	Tmed6	3.29	0.004

1425617_at	DEAH (Asp-Glu-Ala-His) box polypeptide 9	Dhx9	3.29	0.001
1434020_at	PDGFA associated protein 1	Pdap1	3.29	0.007
1423138_at	WD repeat domain 4	Wdr4	3.28	0.000
1430700_a_at	phospholipase A2, group VII	Pla2g7	3.26	0.009
1460059_at	uridine phosphorylase 2	Upp2	3.25	0.000
1422230_s_at	cytochrome P450, family 2, subfamily a, polypeptide 4	Cyp2a4	3.24	0.001
1446966_at	Hypothetical protein E230025K15	E230025K15	3.23	0.006
1451807_at	nuclear receptor subfamily 1, group I, member 2	Nr1i2	3.22	0.009
1441008_at	dymeclin	Dym	3.22	0.000
1454655_at	diacylglycerol kinase, delta	Dgkd	3.22	0.004
1454200_at	zinc finger homeobox 1b	Zfhx1b	3.19	0.002
1418480_at	chemokine (C-X-C motif) ligand 7	Cxcl7	3.18	0.001
1454158_at	membrane protein, palmitoylated 7 (MAGUK p55 subfamily member 7)	Mpp7	3.17	0.002
1425638_at	centaurin, alpha 2	Centa2	3.17	0.004
1422894_at	Scm-like with four mbt domains 1	Sfmbt1	3.16	0.001
1452344_at	synaptojanin 2	Synj2	3.16	0.006
1456316_a_at	acyl-Coenzyme A binding domain containing 3	Acbd3	3.15	0.010
1443083_at	Cleavage and polyadenylation specific factor 2	Cpsf2	3.14	0.004
1429489_at	transcription elongation factor B polypeptide 3 binding protein 1	Tceb3bp1	3.13	0.008
1446807_at	Ubiquitin specific protease 8	Usp8	3.12	0.000
1438771_at	bromodomain containing 1	Brd1	3.09	0.001
1436544_at	ATPase, Class V, type 10D	Atp10d	3.05	0.002
1444931_at	Endothelial-specific receptor tyrosine kinase	Tek	3.04	0.004
1416065_a_at	ankyrin repeat domain 10	Ankrd10	3.01	0.004
1423530_at	serine/threonine kinase 32C	Stk32c	3.01	0.000
1438246_at	casein kinase 1, gamma 1	Csnk1g1	3.00	0.005
1417904_at	DNA cross-link repair 1A, PSO2 homolog (S. cerevisiae)	Dclre1a	2.99	0.000
1433780_at	ubinuclein 1	Ubn1	2.99	0.006
1424167_a_at	phosphomannomutase 1	Pmm1	2.99	0.000
1423971_at	THO complex 3	Thoc3	2.98	0.005
1431182_at	heat shock protein 8	Hspa8	2.98	0.001
1423867_at	serine (or cysteine) proteinase inhibitor, clade A, member 3K	Serpina3k	2.97	0.003
1451293_at	RNA, U3 small nucleolar interacting protein 2	Rnu3ip2	2.97	0.002
1456653_a_at	methylenetetrahydrofolate dehydrogenase (NADP+ dependent) 1-like	Mthfd1l	2.96	0.004
1418057_at	T-cell lymphoma invasion and metastasis 1	Tiam1	2.95	0.001
1439972_at	ethanolamine kinase 1	Etnk1	2.94	0.001

1416737_at	glycogen synthase 3, brain	Gys3	2.93	0.005
1439197_at	phosphatidylinositol 4-kinase, catalytic, beta polypeptide	Pik4cb	2.93	0.010
1437106_at	jumonji, AT rich interactive domain 1A (Rbp2 like)	Jarid1a	2.90	0.005
1452431_s_at	histocompatibility 2, class II antigen A, alpha	H2-Aa	2.88	0.007
1417705_at	OTU domain, ubiquitin aldehyde binding 1	Otub1	2.86	0.001
1439760_x_at	uroplakin 1B	Upk1b	2.80	0.008
1450188_s_at	lipase, endothelial	Lipg	2.80	0.003
1460212_at	guanine nucleotide binding protein, alpha transducing 1	Gnat1	2.79	0.002
1424367_a_at	homer homolog 2 (Drosophila)	Homer2	2.79	0.000
1429656_at	Rho-related BTB domain containing 1	Rhobtb1	2.78	0.003
1448506_at	serine (or cysteine) proteinase inhibitor, clade A, member 6	Serpina6	2.78	0.006
1417297_at	inositol 1,4,5-triphosphate receptor 3	Itpr3	2.78	0.005
1426758_s_at	GTL2, imprinted maternally expressed untranslated mRNA	Gtl2	2.77	0.003
1426259_at	pantothenate kinase 3	Pank3	2.76	0.010
1452708_a_at	Luc7 homolog (S. cerevisiae)-like	Luc7l	2.76	0.006
1433617_s_at	UDP-Gal:betaGlcNAc beta 1,4-galactosyltransferase, polypeptide 5	B4galt5	2.76	0.002
1456656_at	lin 7 homolog a (C. elegans)	Lin7a	2.75	0.005
1442186_at	spinocerebellar ataxia 7 homolog (human)	Sca7	2.75	0.005
1427379_at	Neuropathy target esterase	Nte	2.75	0.005
1430781_at	adenylate kinase 7	Ak7	2.74	0.008
1448147_at	tumor necrosis factor receptor superfamily, member 19	Tnfrsf19	2.73	0.001
1422415_at	angiogenin, ribonuclease A family, member 2	Ang2	2.73	0.005
1441291_at	v-abl Abelson murine leukemia oncogene 1	Abl1	2.72	0.004
1456499_at	Ring finger protein 130	Rnf130	2.71	0.003
1428951_at	nucleolar protein 8	Nol8	2.70	0.003
1451731_at	ATP-binding cassette, sub-family A (ABC1), member 3	Abca3	2.69	0.000
1438009_at	histone 1, H2ad	Hist1h2ad	2.69	0.001
1417709_at	cytochrome P450, family 46, subfamily a, polypeptide 1	Cyp46a1	2.69	0.000
1436123_at	bassoon	Bsn	2.68	0.004
1420832_at	quiescin Q6	Qscn6	2.68	0.000
1449292_at	RB1-inducible coiled-coil 1	Rb1cc1	2.68	0.005
1451983_at	Iroquois related homeobox 1 (Drosophila)	Irx1	2.68	0.001
1425477_x_at	histocompatibility 2, class II antigen A, beta 1	H2-Ab1 /// Rmcs1	2.68	0.005
1430384_at	transducin-like enhancer of split 4, homolog of Drosophila E(spl)	Tle4	2.67	0.003
1429267_at	thioesterase, adipose associated	Thea	2.67	0.000
1421099_at	basic helix-loop-helix domain containing, class B3	Bhlhb3	2.66	0.004

1451989_a_at	microtubule-associated protein, RP/EB family, member 2	Mapre2	2.65	0.001
1434499_a_at	lactate dehydrogenase 2, B chain	Ldh2	2.65	0.008
1417623_at	solute carrier family 12, member 2	Slc12a2	2.65	0.004
1457306_at	Polymerase (DNA directed), epsilon 3 (p17 subunit)	Pole3	2.65	0.007
1447791_s_at	guanine nucleotide binding protein, alpha 14	Gna14	2.64	0.003
1443003_at	Atpase, class VI, type 11C	Atp11c	2.63	0.004
1426850_a_at	mitogen activated protein kinase kinase 6	Map2k6	2.63	0.000
1416933_at	P450 (cytochrome) oxidoreductase	Por	2.61	0.009
1448978_at	neuronal guanine nucleotide exchange factor	Ngef	2.61	0.002
1451593_at	Histocompatibility 2, D region locus 1	H2-Q1	2.61	0.007
1453851_a_at	growth arrest and DNA-damage-inducible 45 gamma	Gadd45g	2.59	0.004
1419266_at	nuclear transcription factor-Y beta	Nfyb	2.56	0.005
1431316_at	itchy	Itch	2.54	0.002
1440966_at	Membrane-associated ring finger (C3HC4) 7	Axot	2.54	0.007
1441988_at	protein phosphatase 1K (PP2C domain containing)	Ppm1k	2.53	0.005
1418495_at	zinc finger CCCH type domain containing 8	Zc3hdc8	2.52	0.001
1449042_at	CCCTC-binding factor	Ctcf	2.52	0.006
1416334_at	WW domain-containing oxidoreductase	Wwox	2.52	0.000
1444430_at	Armadillo repeat containing 8	Armc8	2.52	0.000
1425317_x_at	serine threonine kinase 31	Stk31	2.52	0.002
1460651_at	linker for activation of T cells	Lat	2.51	0.001
1418431_at	kinesin family member 5B	Kif5b	2.50	0.000
1442092_at	Fanconi anemia, complementation group D2	Fancd2	2.48	0.010
1437658_a_at	RNA, U22 small nucleolar	Rnu22	2.47	0.005
1422142_at	nephrosis 1 homolog, nephrin (human)	Nphs1	2.47	0.004
1457027_at	dehydrogenase E1 and transketolase domain containing 1	Dhtkd1	2.47	0.000
1446824_at	Phosphoglucomutase 1	Pgm1	2.47	0.002
1458891_at	GTPase activating RANGAP domain-like 3	Garnl3	2.46	0.009
1416988_at	mutS homolog 2 (E. coli)	Msh2	2.46	0.003
1419391_at	myogenin	Myog	2.45	0.004
1449306_at	heat shock factor 2	Hsf2	2.44	0.004
1427995_at	carboxypeptidase A5	Cpa5	2.43	0.002
1417890_at	pyridoxal (pyridoxine, vitamin B6) phosphatase	Pdxp	2.42	0.003
1456623_at	tropomyosin 1, alpha	Tpm1	2.42	0.009
1425284_a_at	RAB27A, member RAS oncogene family	Rab27a	2.42	0.000
1425139_at	sestrin 2	Sesn2	2.41	0.006

1448759_at	interleukin 2 receptor, beta chain	Il2rb	2.40	0.003
1428294_at	zinc finger protein 259	Zfp259	2.40	0.001
1440771_at	zinc finger with KRAB and SCAN domains 1	Zkscan1	2.40	0.006
1456941_at	telomerase reverse transcriptase	Tert	2.40	0.002
1450387_s_at	adenylate kinase 3 alpha-like 1	Ak3l1	2.39	0.000
1434178_at	myeloid/lymphoid or mixed-lineage leukemia 3	Mll3	2.39	0.000
1417385_at	aminopeptidase puromycin sensitive	Npepps	2.39	0.000
1453864_at	retinol dehydrogenase 14 (all-trans and 9-cis)	Rdh14	2.38	0.000
1457350_at	period homolog 2 (Drosophila)	Per2	2.38	0.008
1418221_at	casein delta	Csnd	2.38	0.007
1433570_s_at	MAK10 homolog, amino-acid N-acetyltransferase subunit, (S. cerevisiae)	Mak10	2.38	0.001
1442511_at	Importin 7	Ipo7	2.37	0.001
1440193_at	Ankyrin repeat domain 12	Ankrd12	2.36	0.005
1456058_at	RNA binding motif protein 27	Rbm27	2.36	0.003
1429911_at	microcephaly, primary autosomal recessive 1	Mcph1	2.35	0.000
1417513_at	ecotropic viral integration site 5	Evi5	2.35	0.002
1455108_at	eukaryotic translation initiation factor 4E member 2	Eif4e2	2.35	0.005
1425824_a_at	proprotein convertase subtilisin/kexin type 4	Pcsk4	2.35	0.000
1451260_at	aldehyde dehydrogenase 1 family, member B1	Aldh1b1	2.35	0.009
1434643_at	transducin (beta)-like 1 X-linked	Tbl1x	2.34	0.006
1421061_at	guanylate cyclase activator 1a (retina)	Guca1a	2.34	0.000
1426704_at	cyclin G associated kinase	Gak	2.33	0.003
1421229_at	DNA fragmentation factor, beta subunit	Dffb	2.33	0.008
1420865_at	zinc finger protein 161	Zfp161	2.33	0.001
1425365_a_at	cytochrome P450, family 2, subfamily d, polypeptide 13	Cyp2d13	2.33	0.003
1430519_a_at	CCR4-NOT transcription complex, subunit 7	Cnot7	2.33	0.000
1429057_at	NMDA receptor regulated 1-like	Narg1l	2.33	0.000
1428449_at	general transcription factor IIIC, polypeptide 2, beta	Gtf3c2	2.32	0.008
1456416_at	core-binding factor, runt domain, alpha subunit 2	Cbfa2t2h	2.32	0.002
1457357_at	Tousled-like kinase 2 (Arabidopsis)	Tlk2	2.31	0.000
1427813_at	iduronate 2-sulfatase	Ids	2.30	0.006
1455509_at	similar to mKIAA0363 protein	LOC192950	2.29	0.005
1445824_at	zinc finger protein 458	Zfp458	2.28	0.006
1455204_at	phosphatidylinositol transfer protein, cytoplasmic 1	Pitpnc1	2.28	0.000
1439381_x_at	MARVEL (membrane-associating) domain containing 1	Marveld1	2.27	0.000
1426327_s_at	ciliary neurotrophic factor /// zinc finger protein 91	Cntf /// Zfp91	2.27	0.002

1419173_at	aminoacylase 1	Acy1	2.27	0.002
1437631_at	Kv channel interacting protein 4	Kcnip4	2.26	0.001
1452911_at	sprouty protein with EVH-1 domain 1, related sequence	Spred1	2.26	0.001
1417190_at	pre-B-cell colony-enhancing factor 1	Pbef1	2.25	0.003
1459462_at	Glucocorticoid induced gene 1	Gig1	2.25	0.001
1434271_at	glucosidase beta 2	Gba2	2.24	0.003
1456486_at	zinc finger protein 574	Zfp574	2.23	0.010
1417253_at	FSHD region gene 1	Frg1	2.22	0.000
1451920_a_at	replication factor C 1	Recc1	2.22	0.005
1438476_a_at	chromodomain helicase DNA binding protein 4	Chd4	2.21	0.010
1442735_at	Ornithine decarboxylase antizyme 2	Oaz2	2.21	0.004
1459764_x_at	Sorting nexin 10	Snx10	2.19	0.002
1434736_at	hepatic leukemia factor	Hlf	2.19	0.002
1427842_at	keratin associated protein 6-3	Krtap6-3	2.18	0.003
1417276_at	tubby-like protein 2	Tulp2	2.18	0.009
1457765_at	Proteasome (prosome, macropain) 26S subunit, non-ATPase, 14	Psmd14	2.17	0.004
1448235_s_at	high mobility group box 1	Hmgb1 /// 49324	2.17	0.003
1450931_at	dedicator of cytokinesis 9	Dock9	2.17	0.007
1431897_at	carbohydrate (N-acetylgalactosamine 4-0) sulfotransferase 9	Chst9	2.16	0.008
1449623_at	thioredoxin reductase 3	Txnrd3	2.16	0.001
1440438_at	WASP family 1	Wasf1	2.16	0.006
1459885_s_at	cytochrome c oxidase, subunit VIIc	Cox7c	2.15	0.003
1445669_at	sprouty homolog 4 (Drosophila)	Spry4	2.15	0.003
1424442_a_at	praja 2, RING-H2 motif containing	Pja2	2.15	0.000
1417353_x_at	small nuclear ribonucleoprotein polypeptide A'	Snrpa1	2.15	0.002
1458274_at	zinc finger protein 69	Zfp69	2.14	0.007
1453612_at	NIMA (never in mitosis gene a)-related expressed kinase 1	Nek1	2.13	0.006
1435275_at	cytochrome c oxidase subunit VIb polypeptide 2	Cox6b2	2.13	0.008
1455522_at	Rho guanine nucleotide exchange factor (GEF) 15	Arhgef15	2.13	0.000
1427713_x_at	POU domain, class 2, transcription factor 2	Pou2f2	2.12	0.004
1434219_at	stromal interaction molecule 2	Stim2	2.11	0.000
1452124_at	ankyrin 3, epithelial /// RIKEN cDNA 2900054D09 gene	Ank3	2.10	0.004
1456735_x_at	acid phosphatase-like 2	Acpl2	2.10	0.001
1449013_at	eukaryotic elongation factor-2 kinase	Eef2k	2.10	0.000
1443940_at	leucine rich repeat containing 22	Lrrc22	2.09	0.006
1449708_s_at	checkpoint kinase 1 homolog (S. pombe)	Chek1	2.09	0.009

1418172_at	heme binding protein 1	Hebp1	2.09	0.001
1454928_at	scaffold attachment factor B	Safb	2.09	0.005
1423704_at	lysophospholipase 3	Lypla3	2.08	0.009
1428130_at	lectin, mannose-binding, 1	Lman1	2.08	0.002
1443838_x_at	fatty acid desaturase 2	Fads2	2.07	0.001
1457767_at	Nischarin	Nisch	2.07	0.004
1423009_at	secretory blood group 1	MGI:1928893	2.07	0.006
1422624_at	REV1-like (S. cerevisiae)	Rev1l	2.07	0.006
1455726_at	gene model 71, (NCBI)	Gm71	2.07	0.000
1443163_at	solute carrier family 39 (zinc transporter), member 2	Slc39a2	2.07	0.004
1438039_at	HECT domain containing 1	Hectd1	2.07	0.005
1460285_at	integrin alpha 9	Itga9	2.07	0.004
1452339_at	a disintegrin-like and metalloprotease with thrombospondin type 1 motif, 7	Adamts7	2.06	0.000
1447274_at	Wiskott-Aldrich syndrome protein interacting protein	Waspip	2.06	0.006
1422168_a_at	brain derived neurotrophic factor	Bdnf	2.06	0.009
1423417_at	SWI/SNF related, actin dependent regulator of chromatin, subfamily c,m1	Smarcc1	2.04	0.003
1429178_at	odd Oz/ten-m homolog 3 (Drosophila)	Odz3	2.04	0.001
1460239_at	tetraspanin 13	Tspan13	2.04	0.001
1452303_at	Rho guanine nucleotide exchange factor (GEF) 10	Arhgef10	2.03	0.010
1424473_at	similar to polymerase (RNA) II (DNA directed) polypeptide H	LOC381040	2.03	0.000
1422028_a_at	E26 avian leukemia oncogene 1, 5' domain	Ets1	2.03	0.003
1427191_at	natriuretic peptide receptor 2	Npr2	2.03	0.000
1460247_a_at	S-phase kinase-associated protein 2 (p45)	Skp2	2.03	0.003
1422886_a_at	CDC like kinase 4	Clk4	2.03	0.000
1417141_at	interferon gamma induced GTPase	Igtp	2.02	0.008
1417506_at	geminin	Gmnn	2.02	0.000
1427025_at	myotubularin related protein 7	Mtmr7	2.02	0.007
1426147_s_at	claudin 10	Cldn10	2.02	0.007
1455861_at	erythrocyte protein band 4.1-like 5	Epb4.1l5	2.02	0.002
1417101_at	heat shock protein 2	Hspa2	2.02	0.006
1455065_x_at	similar to glucosamine-6-phosphate deaminase	LOC231914	2.01	0.005
1421906_at	peroxisome proliferator activated receptor binding protein	Pparbp	2.00	0.004
1415729_at	3-phosphoinositide dependent protein kinase-1	Pdpk1	2.00	0.000
1428574_a_at	chimerin (chimaerin) 2	Chn2	1.99	0.007
1457711_at	RAN binding protein 3	Ranbp3	1.99	0.003
1419393_at	ATP-binding cassette, sub-family G (WHITE), member 5	Abcg5	1.99	0.009

1432099_a_at	proline dehydrogenase (oxidase) 2	Prodh2	1.99	0.000
1438464_at	AT rich interactive domain 4A (Rbp1 like)	Arid4a	1.98	0.008
1452741_s_at	glycerol phosphate dehydrogenase 2, mitochondrial	Gpd2	1.98	0.005
1447803_x_at	Capping protein (actin filament), gelsolin-like	Capg	1.98	0.005
1455267_at	estrogen-related receptor gamma	Esrrg	1.98	0.007
1442629_at	5'-nucleotidase, cytosolic II	Nt5c2	1.97	0.003
1448539_a_at	aspartoacylase (aminoacylase) 3	Acy3	1.97	0.000
1423646_at	zinc finger, DHHC domain containing 3	Zdhhc3	1.97	0.002
1418789_at	syntrophin, gamma 2	Sntg2	1.96	0.006
1448328_at	SH3-domain binding protein 2	Sh3bp2	1.96	0.001
1449084_s_at	SH3 domain protein D19	Sh3d19	1.96	0.001
1415850_at	RAS p21 protein activator 3	Rasa3	1.95	0.001
1452639_at	ectonucleotide pyrophosphatase/phosphodiesterase 4	Enpp4	1.95	0.005
1424698_s_at	grancalcin	Gca	1.94	0.007
1419076_a_at	breast cancer 2	Brca2	1.94	0.004
1440747_at	Similar to Cpo protein	Cpox	1.93	0.002
1427089_at	cyclin T2	Ccnt2	1.93	0.001
1448065_at	UDP-Gal:betaGlcNAc beta 1,4-galactosyltransferase, polypeptide 3	B4galt3	1.93	0.010
1417764_at	signal sequence receptor, alpha	Ssr1	1.93	0.003
1430632_at	ankyrin repeat and SOCS box-containing protein 1	Asb1	1.92	0.008
1425893_a_at	fragile histidine triad gene	Fhit	1.91	0.001
1418912_at	plexin domain containing 2	Plxdc2	1.91	0.004
1448632_at	proteasome (prosome, macropain) subunit, beta type 10	Psmb10	1.91	0.006
1426690_a_at	sterol regulatory element binding factor 1	Srebf1	1.90	0.002
1423112_at	ubiquitin-conjugating enzyme E2D 3 (UBC4/5 homolog, yeast)	Ube2d3	1.90	0.003
1451104_a_at	U1 small nuclear ribonucleoprotein polypeptide A	Snrp70	1.90	0.002
1456011_x_at	acetyl-Coenzyme A acyltransferase 1	Acaa1	1.89	0.001
1421950_at	prefoldin 2	Pfdn2	1.89	0.000
1428512_at	basic helix-loop-helix domain containing, class B9	Bhlhb9	1.89	0.006
1449912_at	synovial sarcoma, X member B, breakpoint 1	Ssxb1	1.88	0.009
1455002_at	protein tyrosine phosphatase 4a1	Ptp4a1	1.88	0.007
1452224_at	zinc finger, CW-type with coiled-coil domain 3	Zcwcc3	1.87	0.001
1450657_at	peptidylprolyl isomerase E (cyclophilin E)	Ppie	1.87	0.005
1449248_at	chloride channel 2	Clcn2	1.87	0.000
1421236_at	receptor (TNFRSF)-interacting serine-threonine kinase 2	Ripk2	1.86	0.009
1429166_s_at	Calmin	Clmn	1.86	0.006

1450726_at	N-acylsphingosine amidohydrolase 2	Asah2	1.86	0.003
1427382_a_at	suppressor of variegation 3-9 homolog 1 (Drosophila)	Suv39h1	1.85	0.006
1452190_at	prolylcarboxypeptidase (angiotensinase C)	Prcp	1.85	0.000
1429888_a_at	heat shock protein 2	Hspb2	1.85	0.002
1427596_at	X (inactive)-specific transcript, antisense	Tsix	1.84	0.004
1456081_a_at	acetoacetyl-CoA synthetase	Aacs	1.84	0.000
1427604_a_at	ATPase, class II, type 9A	Atp9a	1.84	0.001
1431143_x_at	apoptosis-inducing factor (AIF)-like mitochondrion-associated inducer of death	Amid	1.84	0.000
1455056_at	LIM domain only 7	Lmo7	1.84	0.008
1438417_at	PWWP domain containing 2	Pwwp2	1.84	0.003
1434514_at	RNA binding motif protein 15	Rbm15	1.83	0.003
1416072_at	CD34 antigen	Cd34	1.83	0.007
1455129_at	Metadherin	Mtdh	1.83	0.002
1436197_at	CDC42 binding protein kinase gamma (DMPK-like)	Cdc42bpg	1.82	0.001
1425178_s_at	serine hydroxymethyl transferase 1 (soluble)	Shmt1	1.82	0.004
1424877_a_at	aminolevulinate, delta-, dehydratase	Alad	1.81	0.000
1417750_a_at	mitochondrial solute carrier protein	MGI:1914962	1.81	0.006
1448405_a_at	CREBBP/EP300 inhibitory protein 1	Cri1	1.81	0.003
1422325_at	melanoma antigen, family A, 5	Magea5	1.81	0.000
1416639_at	solute carrier family 2 (facilitated glucose transporter), member 5	Slc2a5	1.81	0.002
1451286_s_at	fusion, derived from t(12;16) malignant liposarcoma (human)	Fus	1.81	0.005
1417319_at	poliovirus receptor-related 3	Pvrl3	1.81	0.009
1424061_at	mannosidase, beta A, lysosomal-like	Manbal	1.80	0.006
1440152_x_at	endothelial differentiation-related factor 1	Edf1	1.80	0.008
1434349_at	valyl-tRNA synthetase 2-like	Vars2l	1.80	0.009
1431505_at	peptidyl prolyl isomerase H	Ppih	1.80	0.008
1416560_at	solute carrier family 13 member 3	Slc13a3	1.80	0.008
1435645_at	monocyte to macrophage differentiation-associated	Mmd	1.79	0.002
1416840_at	Mid1 interacting protein 1 (gastrulation specific G12-like (zebrafish))	Mid1ip1	1.79	0.003
1457635_s_at	Nuclear receptor subfamily 3, group C, member 1	Nr3c1	1.79	0.001
1428974_s_at	leucine zipper transcription factor-like 1	Lztfl1	1.78	0.009
1453836_a_at	monoglyceride lipase	Mgll	1.78	0.000
1422806_x_at	inhibitor of growth family, member 3	Ing3	1.77	0.000
1437067_at	putative homeodomain transcription factor 2	Phtf2	1.77	0.009
1448868_at	SCAN domain-containing 1	Scand1	1.76	0.001
1426606_at	cartilage acidic protein 1	Crtac1	1.76	0.005

1424899_at	nicotinamide nucleotide adenylyltransferase 3	Nmnat3	1.76	0.001
1450420_at	stromal antigen 1	Stag1	1.76	0.007
1415687_a_at	prosaposin	Psap	1.75	0.009
1456013_x_at	solute carrier family 35, member A4	Slc35a4	1.75	0.001
1449121_at	FUS interacting protein (serine-arginine rich) 1	Fusip1	1.75	0.002
1449486_at	carboxylesterase 1	Ces1	1.74	0.000
1429062_at	kinesin family member 16B	Kif16b	1.74	0.002
1419104_at	abhydrolase domain containing 6	Abhd6	1.74	0.005
1452702_at	chloride channel 7	Clcn7	1.74	0.004
1452257_at	3-hydroxybutyrate dehydrogenase (heart, mitochondrial)	Bdh	1.74	0.003
1427776_a_at	fibroblast growth factor receptor 4	Fgfr4	1.74	0.009
1419130_at	deaminase domain containing 1	Deadc1	1.74	0.003
1443962_at	transcription factor Dp 2	Tfdp2	1.74	0.002
1452823_at	glutathione S-transferase kappa 1	Gstk1	1.73	0.006
1440660_at	Nuclear factor I/A	Nfia	1.73	0.001
1416672_s_at	mucolipin 1	Mcoln1	1.73	0.001
1435748_at	guanine deaminase	Gda	1.72	0.001
1436167_at	similar to Shb-like adapter protein, Shf - human	LOC435684	1.72	0.003
1454640_at	coiled-coil-helix-coiled-coil-helix domain containing 7	Chchd7	1.72	0.000
1418600_at	Kruppel-like factor 1 (erythroid)	Klf1	1.72	0.008
1426383_at	cryptochrome 2 (photolyase-like)	Cry2	1.72	0.006
1423441_at	transcription factor B2, mitochondrial	Tfb2m	1.72	0.008
1457232_at	F-box and leucine-rich repeat protein 21	Fbxl21	1.72	0.006
1426782_at	G protein-coupled receptor 125	Gpr125	1.72	0.002
1450769_s_at	StAR-related lipid transfer (START) domain containing 5	Stard5	1.71	0.009
1423390_at	seven in absentia 1A	Siah1a	1.71	0.008
1435836_at	Pyruvate dehydrogenase kinase, isoenzyme 1	Pdk1	1.71	0.008
1416435_at	lymphotoxin B receptor	Ltbr	1.71	0.000
1426288_at	low density lipoprotein receptor-related protein 4	Lrp4	1.71	0.001
1429033_at	golgi coiled coil 1	Gcc1	1.71	0.003
1424703_at	HemK methyltransferase family member 1	Hemk1	1.71	0.002
1455871_s_at	Tax1 (human T-cell leukemia virus type I) binding protein 3	Tax1bp3 /// Rpl13	1.70	0.000
1426025_s_at	lysosomal-associated protein transmembrane 5	Laptm5	1.70	0.001
1458327_x_at	solute carrier family 26 (sulfate transporter), member 1	Slc26a1	1.70	0.006
1426713_s_at	glutamyl-prolyl-tRNA synthetase	Eprs	1.70	0.000
1449800_x_at	PHD finger protein 7	Phf7	1.70	0.001

1443757_x_at	Galactosidase, beta 1-like	D1Ertd161e	1.69	0.009
1452235_at	mannosidase, alpha, class 1B, member 1	Man1b1	1.69	0.006
1456467_s_at	nemo like kinase	Nlk	1.69	0.004
1431464_a_at	phosphomannomutase 2	Pmm2	1.69	0.004
1451195_a_at	thioredoxin domain containing 1	Txndc1	1.68	0.010
1456012_x_at	ribonuclease T2	Rnaset2	1.68	0.004
1456054_a_at	pumilio 1 (Drosophila)	Pum1	1.68	0.007
1416725_at	transcription factor 4	Tcf4	1.68	0.010
1432086_a_at	RIB43A domain with coiled-coils 2	Ribc2	1.68	0.003
1426924_at	membrane associated DNA binding protein	Mnab	1.68	0.008
1459170_at	Forkhead box O1	Foxo1	1.68	0.003
1434145_s_at	serine hydrolase-like	Serhl	1.68	0.006
1452831_s_at	phosphoribosyl pyrophosphate amidotransferase	Ppat	1.67	0.003
1455558_at	gene model 114, (NCBI)	Gm114	1.67	0.004
1428172_at	PRP39 pre-mRNA processing factor 39 homolog (yeast)	Prpf39	1.67	0.007
1418583_at	histidine triad nucleotide binding protein 3	Hint3	1.67	0.000
1448528_at	programmed cell death 10	Pdcd10	1.67	0.010
1456541_x_at	ATPase family, AAA domain containing 3A	Atad3a	1.66	0.007
1448385_at	solute carrier family 15, member 4	Slc15a4	1.66	0.009
1457024_x_at	solute carrier family 35 (UDP-galactose transporter), member 2	Slc35a2	1.66	0.001
1451602_at	sorting nexin 6	Snx6	1.66	0.000
1431188_a_at	target of myb1 homolog (chicken)	Tom1	1.65	0.001
1452494_s_at	solute carrier organic anion transporter family, member 1b2	Slco1b2	1.65	0.000
1435133_at	UDP-glucose ceramide glucosyltransferase	Ugcg	1.65	0.005
1460597_at	Additional sex combs like 2 (Drosophila)	Asxl2	1.65	0.010
1460670_at	RIO kinase 3 (yeast)	Riok3	1.65	0.000
1428367_at	N-deacetylase/N-sulfotransferase (heparan glucosaminyl) 1	Ndst1	1.65	0.006
1449383_at	adenylosuccinate synthetase like 1	Adssl1	1.65	0.004
1418091_at	transcription factor CP2-like 1	Tcfcp2l1	1.65	0.009
1416685_s_at	fibrillarin	Fbl	1.64	0.005
1427027_a_at	general transcription factor III A	Gtf3a	1.64	0.000
1439463_x_at	high mobility group box 1	Hmgb1 /// LOC38	1.64	0.004
1435370_a_at	carboxylesterase 3	Ces3	1.64	0.002
1419670_at	formiminotransferase cyclodeaminase	Ftcd	1.64	0.004
1442519_at	Dendritic cell protein GA17	Ga17	1.64	0.006
1434930_at	two pore channel 1	Tpcn1	1.64	0.001

1446305_at	hypothetical gene supported by AK045626	LOC432657	1.63	0.001
1417538_at	solute carrier family 35 (CMP-sialic acid transporter), member 1	Slc35a1	1.63	0.000
1434291_a_at	small EDRK-rich factor 1	Serf1	1.63	0.010
1428343_at	REST corepressor 3	Rcor3	1.63	0.000
1417919_at	protein phosphatase 1, regulatory (inhibitor) subunit 7	Ppp1r7	1.63	0.000
1426100_a_at	thymidine kinase 2, mitochondrial	Tk2	1.63	0.001
1428234_at	cleavage and polyadenylation specific factor 6	Cpsf6	1.63	0.002
1435451_at	helicase, mus308-like (Drosophila)	MGI:2176740	1.63	0.006
1448273_at	glutathione synthetase	Gss	1.62	0.001
1452375_at	aldehyde dehydrogenase 4 family, member A1	Aldh4a1	1.62	0.001
1423744_x_at	eukaryotic translation initiation factor 2, subunit 3, structural gene X-linked	Eif2s3x	1.62	0.003
1424821_at	Nedd4 family interacting protein 1	Ndfip1	1.61	0.001
1426051_a_at	centromere autoantigen B	Cenpb	1.61	0.004
1423898_a_at	thyroid hormone receptor interactor 12	Trip12	1.61	0.001
1417061_at	solute carrier family 40 (iron-regulated transporter), member 1	Slc40a1	1.61	0.001
1438993_a_at	ATPase, H+ transporting, V1 subunit D	Atp6v1d	1.61	0.005
1436269_s_at	protease, serine, 25	Prss25	1.61	0.004
1434224_at	transducin (beta)-like 2	Tbl2	1.61	0.006
1428488_at	phosphatidylinositol glycan, class K	Pigk	1.60	0.000
1441733_s_at	nucleoporin 153	Nup153	1.60	0.000
1438405_at	fibroblast growth factor 7	Fgf7	1.60	0.003
1421052_a_at	spermine synthase	Sms	1.60	0.003
1429109_at	ring finger protein 184	Rnf184	1.60	0.009
1450720_at	acid phosphatase 1, soluble	Acp1	1.60	0.003
1429515_at	ubiquitin protein ligase E3 component n-recognin 2	Ubr2	1.60	0.002
1426777_a_at	Wiskott-Aldrich syndrome-like (human)	Wasl	1.60	0.006
1417241_at	EST X83328	X83328	1.60	0.002
1425280_at	leukocyte receptor cluster (LRC) member 1	Leng1	1.60	0.006
1451121_a_at	glioma tumor suppressor candidate region gene 2	Gltscr2	1.59	0.000
1423907_a_at	NADH dehydrogenase (ubiquinone) Fe-S protein 8	Ndufs8	1.59	0.000
1451290_at	microtubule-associated protein 1 light chain 3 alpha	Map1lc3a	1.59	0.001
1436050_x_at	hairy and enhancer of split 6 (Drosophila)	Hes6	1.59	0.003
1436291_a_at	dihydropyrimidinase	Dpys	1.59	0.005
1452841_at	phosphoglucomutase 2-like 1	Pgm2l1	1.59	0.004
1451529_at	small glutamine-rich tetratricopeptide repeat (TPR)-containing, beta	Sgtb	1.59	0.007
1436946_s_at	guanine nucleotide binding protein (G protein), gamma 5 subunit	Gng5	1.59	0.004

1453136_at	F-box protein 30	Fbxo30	1.59	0.002
1423442_a_at	F-box and WD-40 domain protein 2	Fbxw2	1.59	0.001
1419105_at	nuclear receptor subfamily 1, group H, member 4	Nr1h4	1.58	0.005
1436724_a_at	ligatin	Lgtn	1.58	0.000
1449055_x_at	poly(rC) binding protein 4	Pcbp4	1.58	0.006
1429052_at	protein tyrosine phosphatase, receptor type, D	Ptprd	1.58	0.001
1439481_at	Importin 9	Ipo9	1.57	0.001
1425927_a_at	activating transcription factor 5	Atf5	1.57	0.001
1453641_at	breast carcinoma amplified sequence 1	Bcas1	1.57	0.002
1452309_at	cingulin-like 1	Cgnl1	1.57	0.000
1453071_s_at	KDEL (Lys-Asp-Glu-Leu) containing 2	Kdelc2	1.56	0.010
1450556_at	spectrin beta 1	Spnb1	1.56	0.008
1460642_at	Tnf receptor associated factor 4	Traf4	1.56	0.004
1423350_at	suppressor of cytokine signaling 5	Socs5	1.56	0.008
1421379_at	zinc finger protein 354B	Zfp354b	1.56	0.008
1417932_at	interleukin 18	Il18	1.56	0.001
1449506_a_at	eukaryotic translation elongation factor 1 delta	Eef1d	1.56	0.001
1439251_at	Iduronidase, alpha-L-	Idua	1.56	0.010
1425068_a_at	testis expressed gene 264	Tex264	1.56	0.003
1416657_at	thymoma viral proto-oncogene 1	Akt1	1.55	0.006
1417694_at	growth factor receptor bound protein 2-associated protein 1	Gab1	1.55	0.002
1460652_at	Estrogen related receptor, alpha	Esrra	1.55	0.008
1424604_s_at	sulfatase modifying factor 1	Sumf1	1.55	0.000
1426675_at	translocase of outer mitochondrial membrane 70 homolog A (yeast)	Tomm70a	1.55	0.001
1443779_s_at	Mblk1-related protein-2	MGI:2443930	1.55	0.000
1452608_at	c-myc binding protein	Mycbp	1.55	0.007
1449067_at	solute carrier family 2 (facilitated glucose transporter), member 2	Slc2a2	1.55	0.001
1416954_at	solute carrier family 25 member 10	Slc25a10	1.55	0.000
1418448_at	Harvey rat sarcoma oncogene, subgroup R	Rras	1.54	0.001
1419395_at	cytosolic acetyl-CoA hydrolase	MGI:1921406	1.54	0.005
1416523_at	ribonuclease, RNase A family, 1 (pancreatic)	Rnase1	1.54	0.002
1416942_at	type 1 tumor necrosis factor receptor shedding aminopeptidase regulator	MGI:1933403	1.54	0.002
1428410_at	Mak3 homolog (S. cerevisiae)	Mak3	1.53	0.002
1420476_a_at	nucleosome assembly protein 1-like 1	Nap1l1	1.53	0.008
1435746_at	serine/arginine-rich protein specific kinase 2	Srpk2	1.53	0.008
1422490_at	BCL2/adenovirus E1B 19kDa-interacting protein 1, NIP2	Bnip2	1.53	0.009

1422516_a_at	fibroblast growth factor (acidic) intracellular binding protein	Fibp	1.53	0.002
1437303_at	interleukin 6 signal transducer	Il6st	1.53	0.005
1426781_at	radical S-adenosyl methionine and flavodoxin domains 1	Rsafd1	1.53	0.003
1417094_at	brain acyl-CoA hydrolase	MGI:1917275	1.53	0.006
1448276_at	tetraspanin 4	Tspan4	1.53	0.000
1426773_at	mitofusin 1	Mfn1	1.53	0.007
1416418_at	gamma-aminobutyric acid (GABA(A)) receptor-associated protein-like 1	Gabarapl1	1.52	0.000
1439237_a_at	slingshot homolog 1 (Drosophila)	Ssh1	1.52	0.004
1424736_at	eukaryotic translation elongation factor 2	Eef2	1.52	0.005
1441682_s_at	exportin, tRNA (nuclear export receptor for tRNAs)	Xpot	1.52	0.001
1418444_a_at	membrane interacting protein of RGS16	MGI:1891827	1.52	0.008
1416616_s_at	caseinolytic protease, ATP-dependent, proteolytic subunit homolog (E. coli)	Clpp	1.52	0.002
1454109_a_at	phosphatidylserine receptor	Ptdsr	1.51	0.002
1424080_at	decapping enzyme, scavenger	Dcps	1.51	0.007
1453069_at	phosphatidylinositol 3-kinase, catalytic, beta polypeptide	Pik3cb	1.51	0.000
1452506_a_at	integrin-linked kinase-associated serine/threonine phosphatase 2C	Ilkap	1.50	0.001
1459476_s_at	cysteine and glycine-rich protein 2 binding protein	Csrp2bp	1.50	0.009
1421945_a_at	brix domain containing 1	Bxdc1	1.50	0.000
1419197_x_at	hepcidin antimicrobial peptide 1	Hamp1	1.50	0.002
1419526_at	Gardner-Rasheed feline sarcoma viral (Fgr) oncogene homolog	Fgr	1.50	0.006
1420368_at	density-regulated protein	Denr	1.50	0.001
1416618_at	protoporphyrinogen oxidase	Ppox	1.50	0.001
1435534_a_at	translocase of outer mitochondrial membrane 20 homolog (yeast)	Tomm20	1.50	0.002
1454840_at	methylcrotonoyl-Coenzyme A carboxylase 2 (beta)	Mccc2	1.50	0.001
1416037_a_at	chaperonin subunit 2 (beta)	Cct2	1.50	0.000
1424333_at	RNA (guanine-9-) methyltransferase domain containing 1	Rg9mtd1	1.50	0.010
1416593_at	glutaredoxin 1 (thioltransferase)	Glrx1	1.50	0.010
1453146_at	GTPase activating protein and VPS9 domains 1	Gapvd1	1.49	0.002
1448131_at	mitofusin 2	Mfn2	1.49	0.007
1448154_at	N-myc downstream regulated gene 2	Ndrg2	1.49	0.001
1428454_at	breast carcinoma amplified sequence 3	Bcas3	1.49	0.007
1428224_at	heterogeneous nuclear ribonucleoprotein D-like	Hnrpdl	1.49	0.005
1434630_at	ankyrin repeat domain 28	Ankrd28	1.49	0.003
1422568_at	nuclear distribution gene E-like homolog 1 (A. nidulans)	Ndel1	1.49	0.002
1448702_at	immediate early response 3 interacting protein 1	Ier3ip1	1.49	0.006
1459678_at	hypothetical LOC497255	LOC497255	1.49	0.008

1454795_at	Cobl-like 1	Cobll1	1.48	0.003
1428149_at	coronin 7	Coro7	1.48	0.001
1418698_a_at	ferrochelatase	Fech	1.48	0.001
1448864_at	SNF related kinase	Snrk	1.48	0.001
1428248_at	nuclear transcription factor, X-box binding 1	Nfx1	1.48	0.001
1423783_at	torsin family 2, member A	Tor2a	1.48	0.003
1424319_at	oral cancer overexpressed 1	Oraov1	1.48	0.004
1434937_at	pam, highwire, rpm 1	Phr1	1.48	0.003
1455570_x_at	calponin 3, acidic	Cnn3	1.48	0.003
1433649_at	amine oxidase, flavin containing 1	Aof1	1.48	0.000
1416155_at	high mobility group box 3	Hmgb3	1.47	0.006
1434412_x_at	STIP1 homology and U-Box containing protein 1	Stub1	1.47	0.008
1453169_a_at	general transcription factor II H, polypeptide 1	Gtf2h1	1.47	0.008
1417605_s_at	calcium/calmodulin-dependent protein kinase I	Camk1	1.47	0.003
1434113_a_at	XPMC2 prevents mitotic catastrophe 2 homolog (Xenopus laevis)	Xpmc2h	1.47	0.000
1429237_at	syntaxin 16	Stx16	1.47	0.004
1459329_at	Tetratricopeptide repeat domain 7	Ttc7	1.47	0.001
1451576_at	protein kinase, DNA activated, catalytic polypeptide	Prkdc	1.47	0.009
1450649_at	guanine nucleotide binding protein (G protein), gamma 10	Gng10	1.47	0.005
1455252_at	Tuberous sclerosis 1	Tsc1	1.47	0.004
1423047_at	toll interacting protein	Tollip	1.47	0.010
1422469_at	TANK-binding kinase 1	Tbk1	1.47	0.001
1450841_at	intergral membrane protein 1	Itm1	1.47	0.000
1424981_at	neurolysin (metallopeptidase M3 family)	Nln	1.47	0.002
1429001_at	pirin	Pir	1.47	0.000
1455696_a_at	PRP4 pre-mRNA processing factor 4 homolog B (yeast)	Prpf4b	1.47	0.004
1454836_at	transmembrane protein 18	Tmem18	1.46	0.005
1436405_at	Dedicator of cytokinesis 4	Dock4	1.46	0.003
1454781_x_at	COMM domain containing 9	Commd9	1.46	0.007
1420842_at	protein tyrosine phosphatase, receptor type, F	Ptprf	1.46	0.002
1456777_at	maltase-glucoamylase	Mgam	1.45	0.003
1434394_at	Bcl3 binding protein	MGI:2684414	1.45	0.002
1460308_a_at	immature colon carcinoma transcript 1	Ict1	1.45	0.004
1454802_x_at	ariadne homolog 2 (Drosophila)	Arih2	1.45	0.005
1428169_at	APG16 autophagy 16-like (S. cerevisiae)	Apg16l	1.45	0.002
1448460_at	activin A receptor, type 1	Acvr1	1.45	0.009

1423589_at	actin related protein 2/3 complex, subunit 4	Arpc4	1.45	0.001
1436834_x_at	malate dehydrogenase 1, NAD (soluble)	Mdh1	1.45	0.010
1440217_at	similar to hypothetical protein FLJ39743	LOC434197	1.45	0.005
1460363_at	trinucleotide repeat containing 6C	Tnrc6c	1.45	0.003
1435543_at	Adenomatosis polyposis coli	Apc	1.44	0.002
1434111_at	latrophilin 2	Lphn2	1.44	0.005
1425096_a_at	pentatricopeptide repeat domain 1	Ptcd1	1.44	0.002
1460213_at	golgi autoantigen, golgin subfamily a, 4	Golga4	1.44	0.009
1426706_s_at	xylulokinase homolog (H. influenzae)	Xylb	1.44	0.005
1430875_a_at	PAK1 interacting protein 1	Pak1ip1	1.44	0.005
1416252_at	serine/threonine kinase 38	Stk38	1.44	0.000
1450816_at	polymerase (DNA directed), gamma 2, accessory subunit	Polg2	1.44	0.001
1418305_s_at	nucleolar protein family A, member 1 (H/ACA small nucleolar RNPs)	Nola1	1.43	0.006
1418668_at	butyryl Coenzyme A synthetase 1	Bucs1	1.43	0.006
1455073_at	cytidine and dCMP deaminase domain containing 1	Cdadc1	1.43	0.002
1423770_at	transmembrane channel-like gene family 6	Tmc6	1.43	0.004
1425142_a_at	heterogeneous nuclear ribonucleoprotein D	Hnrpd	1.43	0.001
1422807_at	ADP-ribosylation factor 5	Arf5	1.43	0.000
1452033_at	GLE1 RNA export mediator-like (yeast	Gle1l	1.43	0.006
1422534_at	cytochrome P450, family 51	Cyp51	1.43	0.009
1437236_a_at	zinc finger protein 110	Zfp110	1.43	0.007
1418992_at	coagulation factor X	F10	1.43	0.002
1447655_x_at	SRY-box containing gene 6	Sox6	1.43	0.001
1455011_at	StAR-related lipid transfer (START) domain containing 4	Stard4	1.43	0.004
1452095_a_at	histocompatibility 47	H47	1.42	0.007
1420157_s_at	ATP-binding cassette, sub-family F (GCN20), member 1	Abcf1	1.42	0.000
1418656_at	LSM5 homolog, U6 small nuclear RNA associated (S. cerevisiae)	Lsm5	1.42	0.006
1423890_x_at	ATPase, Na+/K+ transporting, beta 1 polypeptide	Atp1b1	1.42	0.009
1429168_at	BTB (POZ) domain containing 4	Btbd4	1.42	0.009
1424358_at	ubiquitin-conjugating enzyme E2E 2 (UBC4/5 homolog, yeast)	Ube2e2	1.42	0.000
1417591_at	prostaglandin E synthase 2	Ptges2	1.42	0.001
1448921_a_at	mitochondrial ribosomal protein S9	Mrps9	1.42	0.002
1437131_x_at	mitochondrial ribosomal protein L11	Mrpl11	1.41	0.002
1451397_at	trinucleotide repeat containing 15	Tnrc15	1.41	0.008
1450826_a_at	serum amyloid A 3	Saa3	1.41	0.005
1448592_at	cartilage associated protein	Crtap	1.41	0.004

1424760_a_at	SET and MYND domain containing 2	Smyd2	1.41	0.002
1429778_at	optineurin	Optn	1.41	0.001
1450921_at	aprataxin	Aptx	1.41	0.002
1457672_at	chromodomain helicase DNA binding protein 9	Chd9	1.41	0.000
1416202_at	B-cell receptor-associated protein 37	Bcap37	1.41	0.006
1423684_at	heterogeneous nuclear ribonucleoprotein K	Hnrpk	1.41	0.001
1429787_x_at	ZW10 interactor	Zwint	1.41	0.000
1449374_at	pipecolic acid oxidase	Pipox	1.41	0.001
1460395_at	NudC domain containing 3	Nudcd3	1.41	0.006
1456109_a_at	mitochondrial ribosomal protein S15	Mrps15	1.40	0.008
1422000_at	aldo-keto reductase family 1, member C12	Akr1c12	1.40	0.008
1435787_at	protein phosphatase 1 (formerly 2C)-like	Ppm1l	1.40	0.001
1423295_at	transmembrane 9 superfamily member 2	Tm9sf2	1.40	0.002
1426870_at	F-box only protein 33	Fbxo33	1.40	0.004
1417558_at	Fyn proto-oncogene	Fyn	1.40	0.006
1416271_at	PERP, TP53 apoptosis effector	Perp	1.40	0.000
1424103_at	APG4 (ATG4) autophagy-related homolog B (S. cerevisiae)	Apg4b	1.40	0.010
1453564_a_at	vacuolar protein sorting 24 (yeast)	Vps24	1.40	0.002
1418503_at	heat shock protein 9A	Hspa9a	1.39	0.000
1427945_at	dihydropyrimidine dehydrogenase	Dpyd	1.39	0.005
1451247_at	major facilitator superfamily domain containing 1	Mfsd1	1.39	0.001
1452289_a_at	ring finger protein 135	Rnf135	1.39	0.000
1433461_at	splicing factor 3b, subunit 2	Sf3b2	1.39	0.007
1452434_s_at	DiGeorge syndrome critical region gene 6	Dgcr6	1.38	0.000
1426403_at	ARP1 actin-related protein 1 homolog B (yeast)	Actr1b	1.38	0.010
1439012_a_at	deoxycytidine kinase	Dck	1.38	0.008
1451728_at	WD repeat domain 13	Wdr13	1.38	0.000
1438510_a_at	histidyl-tRNA synthetase	Hars	1.38	0.001
1435347_at	staufen (RNA binding protein) homolog 1 (Drosophila)	Stau1	1.38	0.005
1448692_at	ubiquilin 4	Ubqln4	1.38	0.006
1424104_at	GCIP-interacting protein p29	MGI:1915842	1.38	0.001
1438096_a_at	deoxythymidylate kinase	Dtymk	1.38	0.008
1455940_x_at	WD repeat domain 6	Wdr6	1.38	0.002
1460631_at	O-linked N-acetylglucosamine (GlcNAc) transferase	Ogt	1.38	0.000
1456712_at	Mblk1-related protein-1	MGI:2651932	1.38	0.006
1434816_at	vacuolar protein sorting 33A (yeast)	Vps33a	1.38	0.002

1428511_at	phosphorylase kinase, gamma 2 (testis)	Phkg2	1.38	0.001
1424000_a_at	ribosomal protein S11	Rps11	1.38	0.001
1451005_at	SMT3 suppressor of mif two 3 homolog 1 (yeast)	Sumo1	1.38	0.001
1416742_at	craniofacial development protein 1	Cfdp1	1.38	0.007
1453215_at	ribonuclease, RNase A family, 10 (non-active)	Rnase10	1.38	0.008
1460703_at	activating signal cointegrator 1 complex subunit 1	Ascc1	1.37	0.005
1435887_at	serine (or cysteine) proteinase inhibitor, clade A member 11	Serpina11	1.37	0.001
1427901_at	mitochondrial ribosomal protein S18C	Mrps18c	1.37	0.001
1423701_at	Coenzyme A synthase	Coasy	1.37	0.001
1455007_s_at	glutamic pyruvate transaminase (alanine aminotransferase) 2	Gpt2	1.37	0.008
1429615_at	zinc finger protein 91	Zfp91	1.37	0.003
1429627_at	peptidylprolyl isomerase (cyclophilin)-like 3	Ppil3	1.37	0.003
1423757_x_at	insulin-like growth factor binding protein 4	Igfbp4	1.37	0.004
1427670_a_at	transcription factor 12	Tcf12	1.37	0.004
1434062_at	RAB GTPase activating protein 1-like	Rabgap1l	1.37	0.001
1450982_at	solute carrier family 9 (sodium/hydrogen exchanger), isoform 3 regulator 1	Slc9a3r1	1.37	0.001
1448869_a_at	mitochondrial ribosomal protein S16	Mrps16	1.37	0.008
1427214_at	agmatine ureohydrolase (agmatinase)	Agmat	1.37	0.009
1449316_at	cytochrome P450, family 4, subfamily f, polypeptide 15	Cyp4f15	1.37	0.001
1438004_at	polymerase (DNA directed) sigma	Pols	1.36	0.001
1433824_x_at	G-rich RNA sequence binding factor 1	Grsf1	1.36	0.002
1433781_a_at	claudin 12	Cldn12	1.36	0.003
1448887_x_at	fractured callus expressed transcript 1	Fxc1	1.36	0.010
1418152_at	nucleosome binding protein 1	Nsbp1	1.36	0.000
1435398_at	syntaxin binding protein 5 (tomosyn)	Stxbp5	1.36	0.003
1417449_at	peroxisomal acyl-CoA thioesterase 1	Pte1	1.36	0.010
1452020_a_at	Cd27 binding protein (Hindu God of destruction)	MGI:1353606	1.36	0.004
1438368_a_at	matrin 3	Matr3	1.36	0.007
1437478_s_at	EF hand domain containing 2	Efhd2	1.36	0.009
1449095_at	vacuolar protein sorting 54 (yeast)	Vps54	1.36	0.000
1422732_at	polymerase (DNA-directed), delta interacting protein 2	Poldip2	1.36	0.001
1452149_at	ubiquitin protein ligase E3B	Ube3b	1.36	0.008
1427200_at	zinc finger, RAN-binding domain containing 1	Zranb1	1.35	0.005
1418763_at	nitrilase family, member 2	Nit2	1.35	0.007
1418566_s_at	NudC domain containing 2	Nudcd2	1.35	0.007
1454060_a_at	neuroblastoma ras oncogene	Nras	1.35	0.001

1460184_at	L-3-hydroxyacyl-Coenzyme A dehydrogenase, short chain	Hadhsc	1.35	0.001
1416765_s_at	mitochondria-associated protein	MGI:1913699	1.35	0.001
1419976_s_at	nuclear factor of activated T-cells, cytoplasmic, calcineurin-dependent 3	Nfatc3	1.35	0.000
1448659_at	caspase 7	Casp7	1.34	0.009
1417368_s_at	NADH dehydrogenase (ubiquinone) 1 alpha subcomplex, 2	Ndufa2	1.34	0.002
1424840_at	ribokinase	Rbks	1.34	0.008
1428428_at	Williams Beuren syndrome chromosome region 21	Wbscr21	1.34	0.006
1448563_at	prohibitin	Phb	1.34	0.001
1427071_at	F-box protein 42	Fbxo42	1.34	0.004
1417081_a_at	synaptogyrin 2	Syngr2	1.34	0.000
1428111_at	solute carrier family 38, member 4	Slc38a4	1.33	0.000
1434782_at	ubiquitin specific protease 42	Usp42	1.33	0.002
1419546_at	ATPase, H+ transporting, V1 subunit C, isoform 1	Atp6v1c1	1.33	0.010
1418257_at	solute carrier family 12, member 7	Slc12a7	1.33	0.000
1429005_at	malignant fibrous histiocytoma amplified sequence 1	Mfhas1	1.33	0.002
1459098_at	Dystonin	Dst	1.33	0.009
1423731_at	aldehyde dehydrogenase 16 family, member A1	Aldh16a1	1.33	0.002
1452000_s_at	seryl-aminoacyl-tRNA synthetase 1	Sars1	1.33	0.009
1452327_at	IQ motif and Sec7 domain 1	Iqsec1	1.33	0.001
1416345_at	translocase of inner mitochondrial membrane 8 homolog a (yeast)	Timm8a	1.33	0.007
1428131_a_at	CDC42 small effector 1	Cdc42se1	1.33	0.004
1453271_at	PHD finger protein 14	Phf14	1.32	0.002
1422869_at	c-mer proto-oncogene tyrosine kinase	Mertk	1.32	0.006
1449849_a_at	F-box and leucine-rich repeat protein 6	Fbxl6	1.32	0.006
1451511_at	3-hydroxyisobutyryl-Coenzyme A hydrolase	Hibch	1.32	0.000
1437014_x_at	Peroxiredoxin 1	Prdx1	1.32	0.005
1424898_at	solute carrier family 10 (sodium/bile acid cotransporter family), member 1	Slc10a1	1.32	0.004
1416904_at	muscleblind-like 1 (Drosophila)	Mbnl1	1.32	0.006
1424355_a_at	transcriptional regulator, SIN3B (yeast)	Sin3b	1.32	0.010
1451274_at	oxoglutarate dehydrogenase (lipoamide)	Ogdh	1.32	0.009
1448321_at	SPARC related modular calcium binding 1	Smoc1	1.32	0.002
1433886_at	eukaryotic translation initiation factor 2B, subunit 5 epsilon	Eif2b5	1.31	0.005
1452782_a_at	thioredoxin 2	Txn2	1.31	0.010
1424723_s_at	cleavage stimulation factor, 3' pre-RNA, subunit 3	Cstf3	1.31	0.009
1424285_s_at	ADP-ribosylation factor-like 6 interacting protein 4	Arl6ip4	1.31	0.007
1439432_x_at	mortality factor 4 like 2	Morf4l2	1.31	0.005

1452039_a_at	Brca1 associated protein 1	Bap1	1.31	0.001
1425021_a_at	peroxisome biogenesis factor 16	Pex16	1.31	0.002
1416637_at	solute carrier family 4 (anion exchanger), member 2	Slc4a2	1.31	0.007
1422603_at	ribonuclease, RNase A family 4	Rnase4	1.31	0.005
1418845_at	protein C	Proc	1.30	0.000
1416647_at	branched chain ketoacid dehydrogenase E1, alpha polypeptide	Bckdha	1.30	0.006
1416831_at	neuraminidase 1	Neu1	1.30	0.002
1433612_at	adaptor-related protein complex 2, sigma 1 subunit	Ap2s1	1.30	0.001
1416533_at	EGL nine homolog 2 (C. elegans)	Egln2	1.30	0.001
1455746_at	kinesin family member 13A	Kif13a	1.30	0.002
1437301_a_at	dishevelled, dsh homolog 1 (Drosophila)	Dvl1	1.30	0.009
1451825_a_at	coatomer protein complex, subunit zeta 1	Copz1	1.30	0.007
1429830_a_at	CD59a antigen	Cd59a	1.30	0.003
1431375_s_at	parvin, alpha	Parva	1.30	0.004
1448723_at	retinol dehydrogenase 7	Rdh7	1.30	0.002
1417870_x_at	cathepsin Z	Ctsz	1.30	0.006
1428917_at	syntaxin 17	Stx17	1.29	0.003
1421148_a_at	Tial1 cytotoxic granule-associated RNA binding protein-like 1	Tial1	1.29	0.005
1428029_a_at	H2A histone family, member V	H2afv	1.29	0.002
1448418_s_at	WD repeat domain 23	Wdr23	1.29	0.004
1456604_a_at	protein-L-isoaspartate (D-aspartate) O-methyltransferase 1	Pcmt1	1.29	0.004
1460389_at	cyclin-dependent kinase 8	Cdk8	1.29	0.005
1423255_at	ATPase, H+ transporting, V1 subunit G isoform 1	Atp6v1g1	1.29	0.006
1422627_a_at	McKusick-Kaufman syndrome protein	Mkks	1.29	0.007
1454760_at	HIV TAT specific factor 1	Htatsf1	1.29	0.001
1423271_at	gap junction membrane channel protein beta 2	Gjb2	1.29	0.000
1434791_at	ATPase, H+ transporting, lysosomal V0 subunit a isoform 2	Atp6v0a2	1.29	0.005
1426823_s_at	proteasome (prosome, macropain) activator subunit 4	Psme4	1.28	0.005
1433750_at	solute carrier family 31, member 1	Slc31a1	1.28	0.007
1451351_at	tetratricopeptide repeat domain 13	Ttc13	1.28	0.008
1424441_at	solute carrier family 27 (fatty acid transporter), member 4	Slc27a4	1.28	0.010
1450937_at	lin 7 homolog c (C. elegans)	Lin7c	1.28	0.005
1426501_a_at	Traf2 binding protein	MGI:2182965	1.28	0.004
1417045_at	BH3 interacting domain death agonist	Bid	1.28	0.002
1452209_at	plakophilin 4	Pkp4	1.27	0.004
1454753_at	arginyl aminopeptidase (aminopeptidase B)-like 1	Rnpepl1	1.27	0.000

1417183_at	DnaJ (Hsp40) homolog, subfamily A, member 2	Dnaja2	1.27	0.000
1417966_at	mitochondrial ribosomal protein L39	Mrpl39	1.27	0.001
1451848_a_at	crystallin, zeta	Cryz	1.27	0.008
1438560_x_at	chaperonin subunit 4 (delta)	Cct4	1.26	0.003
1420525_a_at	ornithine transcarbamylase	Otc	1.26	0.005
1429048_at	biogenesis of lysosome-related organelles complex-1, subunit 2	Bloc1s2	1.26	0.004
1439268_x_at	eukaryotic translation initiation factor 3, subunit 6	Eif3s6	1.26	0.001
1437009_a_at	zinc finger protein 364	Zfp364	1.25	0.004
1451088_a_at	oxidase assembly 1-like	Oxa1l	1.25	0.003
1448542_at	BRCA2 and CDKN1A interacting protein	Bccip	1.25	0.005
1449244_at	cadherin 2	Cdh2	1.25	0.001
1453156_s_at	zinc binding alcohol dehydrogenase, domain containing 1	Zadh1	1.24	0.006
1423680_at	fatty acid desaturase 1	Fads1	1.24	0.003
1422032_a_at	zinc finger, A20 domain containing 3	Za20d3	1.24	0.004
1424088_at	melanocyte proliferating gene 1	MGI:1929864	1.24	0.003
1450714_at	ornithine decarboxylase antizyme inhibitor	Oazin	1.24	0.009
1451205_at	proteasome (prosome, macropain) subunit, beta type 4	Psmb4	1.24	0.000
1424746_at	Kinesin family member 1C	Kif1c	1.24	0.005
1456628_x_at	ribosomal protein S24	Rps24	1.24	0.009
1423894_a_at	DALR anticodon binding domain containing 3	Dalrd3	1.24	0.000
1423687_a_at	mannosidase, alpha, class 2C, member 1	Man2c1	1.23	0.000
1424078_s_at	peroxisomal biogenesis factor 6	Pex6	1.23	0.005
1451366_at	COP9 (constitutive photomorphogenic) homolog, subunit 6	Cops6	1.23	0.001
1427578_a_at	integrin beta 4 binding protein	Itgb4bp	1.23	0.003
1436609_a_at	low density lipoprotein receptor-related protein associated protein 1	Lrpap1	1.23	0.003
1423042_at	fibroblast growth factor inducible 14	Fin14	1.23	0.008
1448149_at	catenin alpha 1	Catna1	1.23	0.009
1448225_at	GPI anchor attachment protein 1	Gpaa1	1.23	0.004
1427918_a_at	ras homolog gene family, member Q	Rhoq	1.22	0.008
1421772_a_at	cytochrome c oxidase subunit VIIa polypeptide 2-like	Cox7a2l	1.22	0.001
1431619_a_at	dystrobrevin binding protein 1	Dtnbp1	1.22	0.006
1448253_at	glutamate dehydrogenase 1	Glud1	1.22	0.005
1448653_at	embryonic ectoderm development	Eed	1.22	0.000
1416738_at	BRCA1 associated protein	Brap	1.21	0.004
1424753_at	nudix (nucleoside diphosphate linked moiety X)-type motif 14	Nudt14	1.21	0.000
1415869_a_at	tripartite motif protein 28	Trim28	1.21	0.002

1416769_s_at	ATPase, H+ transporting, V0 subunit B	Atp6v0b	1.21	0.000
1416394_at	Bcl2-associated athanogene 1	Bag1	1.21	0.008
1438292_x_at	adenosine kinase	Adk	1.21	0.009
1456580_s_at	ATP synthase, H+ transporting, mitochondrial F1 complex, delta subunit	Atp5d	1.20	0.009
1428709_a_at	mitochondrial ribosomal protein L24	Mrpl24	1.20	0.005
1417809_at	solute carrier family 22 (organic cation transporter), member 18	Slc22a18	1.20	0.007
1424892_at	zinc finger protein 95	Zfp95	1.20	0.006
1430500_s_at	metaxin 2	Mtx2	1.20	0.008
1417452_a_at	Finkel-Biskis-Reilly murine sarcoma virus ubiquitously expressed	Fau	1.20	0.009
1421944_a_at	asialoglycoprotein receptor 1	Asgr1	1.20	0.002
1428326_s_at	heat-responsive protein 12	Hrsp12	1.20	0.003
1424461_at	dynactin 2	Dctn2	1.20	0.009
1425381_a_at	transferrin receptor 2	Trfr2	1.19	0.000
1421842_a_at	secretory carrier membrane protein 4	Scamp4	1.19	0.001
1415761_at	mitochondrial ribosomal protein L52	Mrpl52	1.19	0.005
1415678_at	protein phosphatase 1A, magnesium dependent, alpha isoform	Ppm1a	1.18	0.005
1454638_a_at	phenylalanine hydroxylase	Pah	1.17	0.001
1451149_at	phosphoglucomutase 2	Pgm2	1.17	0.002
1454763_at	ankyrin repeat domain 17	Ankrd17	1.17	0.004
1424540_at	Homeodomain interacting protein kinase 1	Hipk1	1.16	0.002
1433934_at	SEC24 related gene family, member A (S. cerevisiae)	Sec24a	1.16	0.005
1451328_at	pecanex-like 3 (Drosophila)	Pcnxl3	1.16	0.005
1417909_at	serine (or cysteine) proteinase inhibitor, clade C (antithrombin), member 1	Serpinc1	1.15	0.000
1418988_at	peroxisome biogenesis factor 7	Pex7	1.15	0.008
1415863_at	eukaryotic translation initiation factor 4, gamma 2	Eif4g2	1.15	0.005
1448336_at	developmentally regulated GTP binding protein 1	Drg1	1.14	0.004
1416605_at	nucleolar protein family A, member 2	Nola2	1.14	0.010
1423911_at	protein phosphatase 2, regulatory subunit B (B56), alpha isoform	Ppp2r5a	1.13	0.005
1423702_at	H1 histone family, member 0	H1f0	1.11	0.004
1435967_s_at	3-hydroxyisobutyrate dehydrogenase	Hibadh	1.11	0.002
1433850_at	protein phosphatase 4, regulatory subunit 2	Ppp4r2	1.10	0.005
1422693_a_at	RNA polymerase II transcriptional coactivator	Rpo2tc1	1.09	0.000
1418897_at	coagulation factor II	F2	1.07	0.008
1431766_x_at	ribosomal protein S2	Rps2	-1.08	0.002
1431177_a_at	ribosomal protein L10A	Rpl10a	-1.11	0.001
1435862_at	Son cell proliferation protein	Son	-1.11	0.004

1416170_at	TNF receptor-associated protein 1	Trap1	-1.12	0.006
1416025_at	fibrinogen, gamma polypeptide	Fgg	-1.12	0.006
1434854_a_at	ribosomal protein S10	Rps10	-1.12	0.009
1434814_x_at	glucose phosphate isomerase 1	Gpi1	-1.13	0.007
1460186_at	SM-11044 binding protein	MGI:1914262	-1.14	0.004
1428152_a_at	Ribosomal protein L18A	Rpl18a	-1.14	0.002
1416565_at	cytochrome c oxidase, subunit VIb polypeptide 1	Cox6b1	-1.14	0.001
1419364_a_at	ribosomal protein S7	Rps7	-1.14	0.008
1452032_at	protein kinase, cAMP dependent regulatory, type I, alpha	Prkar1a	-1.14	0.006
1426522_at	hydroxyacyl-CoA deh/ase/3-ketoacyl-CoA thiolase/enoyl-CoA hydratase	Hadhb	-1.15	0.005
1428868_a_at	ornithine decarboxylase antizyme	Oaz1	-1.15	0.010
1433569_x_at	RAN, member RAS oncogene family	Ran	-1.15	0.002
1415876_a_at	ribosomal protein S26	Rps26	-1.16	0.001
1424368_s_at	ubiquilin 1	Ubqln1	-1.16	0.008
1450845_a_at	basic leucine zipper and W2 domains 1	Bzw1	-1.17	0.002
1423993_at	ATPase, H+ transporting, V1 subunit F	Atp6v1f	-1.17	0.001
1422819_at	mitochondrial ribosomal protein L36	Mrpl36	-1.17	0.001
1422525_at	ATP synthase, H+ transporting, mitochondrial F1F0 complex, subunit e	Atp5k	-1.17	0.007
1424721_at	microfibrillar-associated protein 3	Mfap3	-1.18	0.004
1438000_x_at	zinc finger protein 622	Zfp622	-1.18	0.008
1423643_at	DEAD (Asp-Glu-Ala-Asp) box polypeptide 39	Ddx39	-1.18	0.005
1418101_a_at	reticulon 3	Rtn3	-1.19	0.008
1439259_x_at	abhydrolase domain containing 4	Abhd4	-1.19	0.009
1418625_s_at	similar to glyceraldehyde-3-phosphate dehydrogenase	LOC14433 /// LO	-1.19	0.009
1418016_at	pumilio 2 (Drosophila)	Pum2	-1.19	0.002
AFFX-GapdhMur/M	similar to glyceraldehyde-3-phosphate dehydrogenase	LOC14433 /// LO	-1.19	0.006
1452220_at	dedicator of cyto-kinesis 1	Dock1	-1.19	0.009
1415713_a_at	DEAD (Asp-Glu-Ala-Asp) box polypeptide 24	Ddx24	-1.19	0.006
1459832_s_at	adaptor-related protein complex AP-1, mu subunit 1	Ap1m1	-1.20	0.008
1447778_x_at	c6.1a protein	MGI:2389572	-1.20	0.009
1419984_s_at	zinc finger protein 644	Zfp644	-1.20	0.002
1422613_a_at	ribosomal protein L7a	Rpl7a	-1.20	0.003
1433648_at	sperm associated antigen 9	Spag9	-1.21	0.006
1450187_a_at	galactose-1-phosphate uridyl transferase	Galt	-1.21	0.008
1417847_at	Unc-51 like kinase 2 (C. elegans)	Ulk2	-1.21	0.006
1454778_x_at	Ribosomal protein S28	Rps28	-1.21	0.010

1434251_at	CCR4-NOT transcription complex, subunit 1	Cnot1	-1.21	0.008
1452196_a_at	NCK-associated protein 1	Nckap1	-1.21	0.009
1452545_a_at	integrin beta 1 (fibronectin receptor beta)	Itgb1	-1.21	0.000
1451074_at	ring finger protein 13	Rnf13	-1.21	0.010
1431037_a_at	ELAV (embryonic lethal, abnormal vision, Drosophila)-like 1 (Hu antigen R)	Elavl1	-1.22	0.006
1460571_at	Dicer1, Dcr-1 homolog (Drosophila)	Dicer1	-1.22	0.008
1437309_a_at	replication protein A1	Rpa1	-1.22	0.003
1420830_x_at	tyrosine 3-monooxygenase/tryptophan 5-monooxygenase activation protein	Ywhaq	-1.22	0.007
1436900_x_at	leptin receptor overlapping transcript	Leprot	-1.22	0.006
1418626_a_at	clusterin	Clu	-1.22	0.000
1455432_at	TAO kinase 1	Taok1	-1.22	0.002
1423050_s_at	heterogeneous nuclear ribonucleoprotein U	Hnrpu	-1.23	0.002
1435900_at	zinc finger protein 297B	Zfp297b	-1.23	0.000
1450668_s_at	heat shock protein 1 (chaperonin 10)	Hspe1	-1.24	0.003
1434017_at	zinc and ring finger 2	Znrf2	-1.24	0.000
1423076_at	sorting nexin 9	Snx9	-1.24	0.007
1425137_a_at	histocompatibility 2, Q region locus 10	H2-Q10	-1.24	0.000
1434066_at	general transcription factor III C 1	Gtf3c1	-1.24	0.009
1435518_at	RAS related protein 1b	Rap1b	-1.24	0.006
1418115_s_at	interferon alpha responsive gene	MGI:1929890	-1.24	0.002
1448198_a_at	NADH dehydrogenase (ubiquinone) 1 beta subcomplex 8	Ndufb8	-1.24	0.001
1416386_a_at	mannose-6-phosphate receptor, cation dependent	M6pr	-1.24	0.010
1423599_a_at	phosducin-like	Pdcl	-1.24	0.003
1415980_at	ATP synthase, H+ transporting, mitochondrial F0 complex, subunit c	Atp5g2	-1.24	0.002
1426688_at	succinate dehydrogenase complex, subunit A, flavoprotein (Fp)	Sdha	-1.25	0.000
1454979_at	diaphanous homolog 1 (Drosophila)	Diap1	-1.25	0.004
1423972_at	electron transferring flavoprotein, alpha polypeptide	Etfa	-1.25	0.003
1423754_at	interferon induced transmembrane protein 3	Ifitm3	-1.25	0.001
1426976_at	ubiquitin specific protease 47	Usp47	-1.25	0.002
1450186_s_at	GNAS (guanine nucleotide binding protein, alpha stimulating) complex locus	Gnas	-1.25	0.003
1439156_at	gene model 962, (NCBI)	Gm962	-1.25	0.009
1419399_at	microsomal triglyceride transfer protein	Mttp	-1.25	0.007
1424639_a_at	3-hydroxy-3-methylglutaryl-Coenzyme A lyase	Hmgcl	-1.25	0.005
1422848_a_at	poly(A) binding protein, nuclear 1	Pabpn1	-1.25	0.001
1433541_a_at	ubiquitin associated protein 2-like	Ubap2l	-1.26	0.001
1449930_a_at	signal sequence receptor, beta	Ssr2	-1.26	0.003

1448324_at	ribonucleic acid binding protein S1	Rnps1	-1.26	0.005
1416197_at	U1 small nuclear ribonucleoprotein 1C	Snrp1c	-1.26	0.008
1448799_s_at	mitochondrial ribosomal protein S12	Mrps12	-1.26	0.002
1450406_a_at	ST3 beta-galactoside alpha-2,3-sialyltransferase 3	St3gal3	-1.26	0.001
1426449_a_at	praja1, RING-H2 motif containing	Pja1	-1.26	0.006
1454605_a_at	phosphatidylinositol 4-kinase type 2 alpha	Pi4k2a	-1.26	0.007
1416409_at	acyl-Coenzyme A oxidase 1, palmitoyl	Acox1	-1.26	0.003
1416347_at	multiple endocrine neoplasia 1	Men1	-1.27	0.001
1428608_at	myosin light chain, regulatory B	Mylc2b	-1.27	0.005
1434851_s_at	crumbs homolog 3 (Drosophila)	Crb3	-1.27	0.007
1416335_at	macrophage migration inhibitory factor	Mif	-1.27	0.007
1438606_a_at	chloride intracellular channel 4 (mitochondrial)	Clic4	-1.27	0.005
1460341_at	pleckstrin homology domain containing, family B (evectins) member 2	Plekhb2	-1.27	0.005
1423075_at	lectin, mannose-binding 2	Lman2	-1.28	0.005
1417227_at	methylcrotonoyl-Coenzyme A carboxylase 1 (alpha)	Mccc1	-1.28	0.005
1416984_at	mitochondrial ribosomal protein S18A	Mrps18a	-1.28	0.009
1437474_at	GATA zinc finger domain containing 2B	Gatad2b	-1.28	0.009
1415672_at	golgi autoantigen, golgin subfamily a, 7	Golga7	-1.28	0.004
1452038_at	capping protein (actin filament) muscle Z-line, alpha 1	Capza1	-1.28	0.001
1454847_at	lipoma HMGIC fusion partner-like 2	Lhfpl2	-1.28	0.005
1441075_at	nitric oxide synthase trafficker	LOC329416	-1.29	0.009
1452820_at	WD repeat domain 11	Wdr11	-1.29	0.001
1415882_at	growth hormone inducible transmembrane protein	Ghitm	-1.29	0.002
1424280_at	motile sperm domain containing 1	Mospd1	-1.29	0.001
1425803_a_at	methyl-CpG binding domain protein 2	Mbd2	-1.29	0.001
1450818_a_at	NADH dehydrogenase (ubiquinone) 1 alpha subcomplex, 7 (B14.5a)	Ndufa7	-1.30	0.005
1422103_a_at	signal transducer and activator of transcription 5B	Stat5b	-1.30	0.002
1427971_at	hyperparathyroidism 2 homolog (human)	Hrpt2	-1.30	0.002
1434801_x_at	solute carrier family 25 member 5	Slc25a5	-1.30	0.001
1422480_at	sorting nexin 3	Snx3	-1.30	0.009
1456133_x_at	integrin beta 5	Itgb5	-1.31	0.002
1451318_a_at	Yamaguchi sarcoma viral (v-yes-1) oncogene homolog	Lyn	-1.31	0.002
1416556_at	sarcoma amplified sequence	Sas	-1.31	0.006
1434796_at	vesicle-associated membrane protein 4	Vamp4	-1.32	0.001
1437741_at	RAB21, member RAS oncogene family	Rab21	-1.32	0.010
1416510_at	mitochondrial ribosomal protein L4	Mrpl4	-1.32	0.002

1426238_at	bone morphogenetic protein 1	Bmp1	-1.32	0.004
1426554_a_at	phosphoglycerate mutase 1	Pgam1	-1.32	0.001
1437211_x_at	ELOVL family member 5, elongation of long chain fatty acids (yeast)	Elovl5	-1.32	0.010
1454852_at	trans-acting transcription factor 1	Sp1	-1.32	0.003
1433910_at	zinc finger, CCHC domain containing 6	Zcchc6	-1.32	0.001
1446778_at	Hormonally upregulated Neu-associated kinase	Hunk	-1.32	0.002
1421823_a_at	protein phosphatase 2a, catalytic subunit, beta isoform	Ppp2cb	-1.32	0.001
1416407_at	phosphoprotein enriched in astrocytes 15	Pea15	-1.32	0.007
1454894_at	SMAD specific E3 ubiquitin protein ligase 2	Smurf2	-1.33	0.001
1426857_a_at	hydroxysteroid dehydrogenase like 2	Hsdl2	-1.33	0.008
1434608_at	DEAD (Asp-Glu-Ala-Asp) box polypeptide 52	Ddx52	-1.33	0.008
1455164_at	Cdc42 GTPase-activating protein	Cdgap	-1.33	0.004
1438155_x_at	phosphatidylinositol glycan, class O	Pigo	-1.33	0.002
1440211_at	cytochrome P450, family 2, subfamily j, polypeptide 11	Cyp2j11	-1.34	0.002
1446618_at	hypothetical gene supported by AK038224	LOC432971	-1.34	0.006
1418151_at	myotubularin related protein 4	Mtmr4	-1.34	0.002
1446087_at	Golgi autoantigen, golgin subfamily a, 1	Golga1	-1.34	0.007
1423235_at	adaptor-related protein complex 3, beta 1 subunit	Ap3b1	-1.34	0.002
1424052_at	THAP domain containing 4	Thap4	-1.34	0.002
1427415_at	F-box protein 43	Fbxo43	-1.34	0.002
1423694_at	potassium channel tetramerisation domain containing 10	Kctd10	-1.34	0.002
1423727_at	cornichon homolog (Drosophila)	Cnih	-1.34	0.001
1416099_at	ribosomal protein L27	Rpl27	-1.34	0.003
1423210_a_at	nucleolar protein family A, member 3	Nola3	-1.34	0.005
1433631_at	eukaryotic translation initiation factor 5	Eif5	-1.35	0.003
1429528_at	RAE1 RNA export 1 homolog (S. pombe)	Rae1	-1.35	0.000
1428442_at	matrix metalloproteinase 24	Mmp24	-1.35	0.006
1421870_at	tripartite motif-containing 44	Trim44	-1.35	0.005
1416280_at	ubiquitin-like 1 (sentrin) activating enzyme E1B	Uble1b	-1.35	0.002
1450957_a_at	sequestosome 1	Sqstm1	-1.35	0.009
1451969_s_at	poly (ADP-ribose) polymerase family, member 3	Parp3	-1.35	0.008
1417086_at	platelet-activating factor acetylhydrolase, isoform 1b, beta1 subunit	Pafah1b1	-1.36	0.004
1423200_at	nuclear receptor co-repressor 1	Ncor1	-1.36	0.003
1423532_at	ring finger protein 44	Rnf44	-1.36	0.005
1421101_a_at	LIM domain binding 2	Ldb2	-1.36	0.008
1443351_at	Glycine receptor, alpha 2 subunit	Glra2	-1.36	0.010

1448726_at	small nuclear RNA activating complex, polypeptide 2	Snapc2	-1.36	0.003
1437347_at	endothelin receptor type B	Ednrb	-1.37	0.002
1417931_at	N-deacetylase/N-sulfotransferase (heparan glucosaminyl) 2	Ndst2	-1.37	0.001
1434487_at	myocyte enhancer factor 2D	Mef2d	-1.37	0.009
1421606_a_at	sulfotransferase family 4A, member 1	Sult4a1	-1.37	0.007
1415814_at	ATPase, H+ transporting, V1 subunit B, isoform 2	Atp6v1b2	-1.37	0.004
1427718_a_at	transformed mouse 3T3 cell double minute 2	Mdm2	-1.37	0.006
1448813_at	arylacetamide deacetylase (esterase)	Aadac	-1.37	0.004
1449024_a_at	hexosaminidase A	Hexa	-1.37	0.002
1455534_s_at	oxysterol binding protein-like 11	Osbpl11	-1.37	0.010
1416036_at	FK506 binding protein 1a	Fkbp1a	-1.37	0.007
1419423_at	stabilin 2	Stab2	-1.37	0.004
1453055_at	semaphorin 6D	Sema6d	-1.37	0.000
1452540_a_at	histone 1, H2b	Hist1h2bc /// Hist:	-1.37	0.004
1415936_at	breast cancer anti-estrogen resistance 3	Bcar3	-1.38	0.002
1433547_s_at	NudC domain containing 1	Nudcd1	-1.38	0.008
1426160_a_at	serine/threonine kinase 16	Stk16	-1.38	0.002
1435093_at	zinc finger, FYVE domain containing 20	Zfyve20	-1.38	0.005
1417119_at	zinc finger like protein 1	Zfpl1	-1.38	0.002
1448823_at	chemokine (C-X-C motif) ligand 12	Cxcl12	-1.38	0.000
1419495_at	inner mitochondrial membrane peptidase 2-like (S. cerevisiae)	Immp2l	-1.38	0.001
1415709_s_at	golgi-specific brefeldin A-resistance factor 1	Gbf1	-1.38	0.004
1437329_at	Protein tyrosine phosphatase-like member b	Ptplb	-1.38	0.005
1448734_at	ceruloplasmin	Cp	-1.38	0.004
1433497_at	aquarius	Aqr	-1.38	0.004
1417324_at	microtubule associated serine/threonine kinase 2	Mast2	-1.39	0.005
1451230_a_at	WW domain binding protein 5	Wbp5	-1.39	0.001
1424523_at	engulfment and cell motility 1, ced-12 homolog (C. elegans)	Elmo1	-1.39	0.005
1458247_s_at	dynactin 5	Dctn5	-1.39	0.003
1452582_at	galactose mutarotase	Galm	-1.39	0.000
1454932_at	REST corepressor 1	Rcor1	-1.39	0.003
1419244_a_at	RAB14, member RAS oncogene family	Rab14	-1.39	0.004
1424255_at	suppressor of Ty 5 homolog (S. cerevisiae)	Supt5h	-1.39	0.000
1448448_a_at	choline kinase beta	Chkb	-1.39	0.003
1451833_a_at	SET domain, bifurcated 1	Setdb1	-1.39	0.007
1448737_at	tetraspanin 7	Tspan7	-1.39	0.002

1452929_at	restin	Rsn	-1.39	0.002
1452662_a_at	eukaryotic translation initiation factor 2, subunit 1 alpha	Eif2s1	-1.39	0.004
1452214_at	SKI-like	Skil	-1.39	0.000
1415912_a_at	ribosomal protein S13	Rps13	-1.40	0.007
1417730_at	exostoses (multiple) 1	Ext1	-1.40	0.001
1454916_s_at	ADP-ribosylation factor interacting protein 1	Arfip1	-1.40	0.008
1459679_s_at	myosin IB	Myo1b	-1.40	0.002
1450011_at	hydroxysteroid (17-beta) dehydrogenase 12	Hsd17b12	-1.40	0.003
1417801_a_at	protein tyrosine phosphatase, receptor-type, F interacting protein, bp 2	Ppfibp2	-1.40	0.007
1429681_a_at	glycoprotein, synaptic 2	Gpsn2	-1.40	0.002
1450844_at	syntaxin 6	Stx6	-1.40	0.002
1438041_at	Phosphodiesterase 7A	Pde7a	-1.40	0.005
1433748_at	zinc finger, DHHC domain containing 18	Zdhhc18	-1.40	0.000
1420440_at	pancreatic polypeptide	Ppy	-1.40	0.003
1422736_at	RAN binding protein 9	Ranbp9	-1.41	0.002
1456131_x_at	dystroglycan 1	Dag1	-1.41	0.001
1448232_x_at	tubulin, alpha 6	Tuba6	-1.41	0.005
1415760_s_at	ATX1 (antioxidant protein 1) homolog 1 (yeast)	Atox1	-1.41	0.009
1428846_at	tetratricopeptide repeat domain 14	Ttc14	-1.41	0.007
1447898_s_at	splicing factor, arginine/serine-rich 6	Sfrs6	-1.41	0.008
1415887_at	Trk-fused gene	Tfg	-1.41	0.003
1448503_at	myeloid cell leukemia sequence 1	Mcl1	-1.41	0.002
1416577_a_at	ring-box 1	Rbx1	-1.42	0.004
1452272_a_at	growth factor, erv1	Gfer	-1.42	0.003
1460330_at	annexin A3	Anxa3	-1.42	0.001
1423916_s_at	myeloid leukemia factor 2	Mlf2	-1.42	0.001
1425051_at	isochorismatase domain containing 1	Isoc1	-1.42	0.005
1436363_a_at	nuclear factor I/X	Nfix	-1.42	0.001
1418436_at	syntaxin 7	Stx7	-1.43	0.001
1416026_a_at	ribosomal protein L12	Rpl12	-1.43	0.003
1441945_s_at	abhydrolase domain containing 14A	Abhd14a	-1.43	0.003
1429251_at	PR domain containing 2, with ZNF domain	Prdm2	-1.43	0.001
1448219_a_at	tyrosine 3-monooxygenase	Ywhaz	-1.43	0.000
1449001_at	isovaleryl coenzyme A dehydrogenase	Ivd	-1.43	0.001
1422601_at	serine (or cysteine) proteinase inhibitor, clade B, member 9	Serpinb9	-1.43	0.004
1422559_at	ubiquitin-conjugating enzyme E2N	Ube2n	-1.43	0.001

1416366_at	NADH dehydrogenase (ubiquinone) 1, subcomplex unknown, 2	Ndufc2	-1.43	0.001
1424057_at	ganglioside-induced differentiation-associated-protein 2	Gdap2	-1.44	0.003
1417291_at	tumor necrosis factor receptor superfamily, member 1a	Tnfrsf1a	-1.44	0.001
1425891_a_at	GH regulated TBC protein 1	Grtp1	-1.44	0.009
1417902_at	solute carrier family 19 (thiamine transporter), member 2	Slc19a2	-1.44	0.004
1425458_a_at	growth factor receptor bound protein 10	Grb10	-1.44	0.008
1457664_x_at	Complement component 2 (within H-2S)	C2	-1.44	0.006
1427546_at	ATP-binding cassette, sub-family A (ABC1), member 8b	Abca8b	-1.44	0.008
1423743_at	archain 1	Arcn1	-1.44	0.007
1419053_at	peroxisomal biogenesis factor 14	Pex14	-1.45	0.003
1416086_at	protein-tyrosine sulfotransferase 2	Tpst2	-1.45	0.001
1418502_a_at	oxidation resistance 1	Oxr1	-1.45	0.000
1426994_at	PH domain and leucine rich repeat protein phosphatase	Phlpp	-1.45	0.000
1435235_at	thioredoxin-like 1	Txnl1	-1.45	0.007
1435972_at	calpastatin	Cast	-1.45	0.009
1448812_at	hippocalcin-like 1	Hpcal1	-1.45	0.006
1417566_at	abhydrolase domain containing 5	Abhd5	-1.45	0.004
1417271_a_at	endoglin	Eng	-1.45	0.002
1439201_at	ubiquitin specific protease 14	Usp14	-1.46	0.009
1425464_at	GATA binding protein 6	Gata6	-1.46	0.008
1415674_a_at	trafficking protein particle complex 4	Trappc4	-1.46	0.007
1451244_a_at	zinc finger protein 422	Zfp422	-1.46	0.002
1416968_a_at	hydroxy-delta-5-steroid dehydrogenase, 3 beta- and steroid delta-isomerase 7	Hsd3b7	-1.46	0.005
1418510_s_at	F-box only protein 8	Fbxo8	-1.46	0.000
1425850_a_at	NIMA (never in mitosis gene a)-related expressed kinase 6	Nek6	-1.46	0.001
1450980_at	GTP binding protein 3	Gtpbp3	-1.46	0.007
1448709_at	AT rich interactive domain 1A (Swi1 like)	Arid1a	-1.47	0.010
1417826_at	aldo-keto reductase family 1, member E1	Akr1e1	-1.47	0.003
1415971_at	myristoylated alanine rich protein kinase C substrate	Marcks	-1.47	0.003
1429352_at	molybdenum cofactor sulfurase	Mocos	-1.47	0.001
1434329_s_at	adiponectin receptor 2	Adipor2	-1.47	0.002
1449027_at	ras homolog gene family, member U	Rhou	-1.47	0.004
1420631_a_at	bladder cancer associated protein homolog (human)	Blcap	-1.48	0.008
1456125_a_at	dynein, cytoplasmic, light chain 1	Dnclc1	-1.48	0.002
1454268_a_at	cytochrome b-245, alpha polypeptide	Cyba	-1.48	0.009
1428756_at	aminoadipate-semialdehyde dehydrogenase-phosphopantetheinyl transferase	Aasdhppt	-1.49	0.002

1427260_a_at	tropomyosin 3, gamma	Tpm3	-1.49	0.003
1421217_a_at	lectin, galactose binding, soluble 9	Lgals9	-1.49	0.001
1454823_at	WD repeat domain 37	Wdr37	-1.49	0.009
1434717_at	Cullin 3	Cul3	-1.49	0.004
1417783_at	amyotrophic lateral sclerosis 2 (juvenile) homolog (human)	Als2	-1.49	0.000
1418648_at	EGL nine homolog 3 (C. elegans)	Egln3	-1.50	0.007
1452262_at	GrpE-like 2, mitochondrial	Grpel2	-1.50	0.006
1418084_at	neuropilin 1	Nrp1	-1.50	0.000
1426055_a_at	phosphatidylinositol glycan, class Q	Pigq	-1.50	0.002
1444713_at	Ubiquitin specific protease 34	Usp34	-1.50	0.010
1419614_at	phospholipase A2, group XIIB	Pla2g12b	-1.50	0.002
1458684_at	Synovial sarcoma translocation, Chromosome 18	Ss18	-1.51	0.004
1449464_at	potassium voltage-gated channel, subfamily Q, member 1	Kcnq1	-1.51	0.001
1433992_at	apical protein, Xenopus laevis-like	Apxl	-1.51	0.009
1421846_at	WD repeat and SOCS box-containing 2	Wsb2	-1.51	0.002
1420673_a_at	acyl-Coenzyme A oxidase 2, branched chain	Acox2	-1.52	0.001
1416250_at	B-cell translocation gene 2, anti-proliferative	Btg2	-1.52	0.009
1419022_a_at	enolase 1, alpha non-neuron	Eno1	-1.52	0.000
1446624_at	Fukuyama type congenital muscular dystrophy homolog (human)	Fcmd	-1.52	0.001
1420617_at	cytoplasmic polyadenylation element binding protein 4	Cpeb4	-1.53	0.001
1449455_at	hemopoietic cell kinase	Hck	-1.53	0.002
1417580_s_at	selenium binding protein 1	Selenbp1	-1.53	0.003
1428785_at	angiomotin-like 1	Amotl1	-1.53	0.000
1437325_x_at	aldehyde dehydrogenase 18 family, member A1	Aldh18a1	-1.53	0.008
1418863_at	GATA binding protein 4	Gata4	-1.53	0.001
1433439_at	copine I	Cpne1	-1.54	0.001
1448629_at	Hermansky-Pudlak syndrome 4 homolog (human)	Hps4	-1.54	0.003
1422001_at	inhibin beta-C	Inhbc	-1.54	0.001
1437277_x_at	transglutaminase 2, C polypeptide	Tgm2	-1.55	0.008
1460025_at	leucine-rich repeats and immunoglobulin-like domains 2	Lrig2	-1.55	0.002
1449137_at	pyruvate dehydrogenase E1 alpha 1	Pdha1	-1.55	0.001
1445348_at	cell division cycle 40 homolog (yeast)	Cdc40	-1.55	0.003
1421210_at	class II transactivator	C2ta	-1.55	0.005
1416960_at	beta-1,3-glucuronyltransferase 3 (glucuronosyltransferase I)	B3gat3	-1.55	0.005
1438264_a_at	tripeptidyl peptidase II	Tpp2	-1.55	0.002
1431146_a_at	copine VIII	Cpne8	-1.55	0.010

1460606_at	hydroxysteroid (17-beta) dehydrogenase 13	Hsd17b13	-1.55	0.002
1456727_a_at	casein kinase 1, delta	Csnk1d	-1.56	0.000
1452490_a_at	adaptor protein complex AP-2, alpha 2 subunit	Ap2a2	-1.56	0.004
1419257_at	transcription elongation factor A (SII) 1	Tcea1	-1.56	0.001
1418634_at	Notch gene homolog 1 (Drosophila)	Notch1	-1.56	0.002
1449341_a_at	stomatin	Stom	-1.56	0.007
1420663_at	T helper-inducing POZ/Krueppel factor	Thpok	-1.56	0.009
1436760_a_at	ribosomal protein S8	Rps8	-1.56	0.004
1426484_at	UBX domain containing 2	Ubxd2	-1.56	0.005
1427283_at	myeloid/lymphoid or mixed-lineage leukemia	Mll	-1.56	0.004
1455321_at	DDHD domain containing 1	Ddhd1	-1.57	0.001
1423569_at	glycine amidinotransferase (L-arginine:glycine amidinotransferase)	Gatm	-1.57	0.003
1452338_s_at	intersectin 1 (SH3 domain protein 1A)	Itsn1	-1.57	0.001
1422620_s_at	phosphatidic acid phosphatase 2a	Ppap2a	-1.57	0.000
1441198_at	zinc finger protein 39	Zfp39	-1.57	0.001
1451459_at	embryonic large molecule derived from yolk sac	MGI:1915033	-1.57	0.000
1455942_at	F-box and leucine-rich repeat protein 11	Fbxl11	-1.58	0.001
1433777_at	l(3)mbt-like 2 (Drosophila)	L3mbtl2	-1.58	0.001
1453022_at	GPI-anchored HDL-binding protein 1	MGI:1915703	-1.58	0.002
1459973_x_at	dipeptidylpeptidase 4	Dpp4	-1.58	0.001
1428644_at	mannoside acetylglucosaminyltransferase 5	Mgat5	-1.58	0.005
1418825_at	interferon inducible protein 1	Ifi1	-1.58	0.009
1430570_at	kynureninase (L-kynurenine hydrolase)	Kynu	-1.58	0.010
1450792_at	TYRO protein tyrosine kinase binding protein	Tyrobp	-1.58	0.008
1460232_s_at	hydroxysteroid dehydrogenase-2, delta<5>-3-beta	Hsd3b2 /// Hsd3b	-1.58	0.006
1449068_at	zinc finger protein 148	Zfp148	-1.58	0.001
1416329_at	cytoplasmic FMR1 interacting protein 1	Cyfip1	-1.59	0.001
1428615_at	purinergic receptor (family A group 5)	P2y5	-1.59	0.002
1449574_a_at	cell division cycle 42 homolog (S. cerevisiae)	Cdc42	-1.59	0.004
1423278_at	protein tyrosine phosphatase, receptor type, K	Ptprk	-1.59	0.003
1447209_at	Forkhead box P1	Foxp1	-1.60	0.004
1452154_at	isoleucine-tRNA synthetase	Iars	-1.60	0.004
1456098_a_at	engulfment and cell motility 2, ced-12 homolog (C. elegans)	Elmo2	-1.60	0.004
1424344_s_at	eukaryotic translation initiation factor 1A	Eif1a	-1.60	0.010
1421884_at	Son of sevenless homolog 1 (Drosophila)	Sos1	-1.60	0.001
1456341_a_at	basic transcription element binding protein 1	Bteb1	-1.60	0.001

1420558_at	selectin, platelet	Selp	-1.61	0.006
1418547_at	tissue factor pathway inhibitor 2	Tfpi2	-1.61	0.000
1432072_at	kinesin family member 2A	Kif2a	-1.61	0.005
1422703_at	glycerol kinase	Gyk	-1.61	0.003
1436755_at	inter-alpha (globulin) inhibitor H5	Itih5	-1.61	0.002
1454654_at	disrupted in renal carcinoma 2 (human)	Dirc2	-1.61	0.006
1460700_at	signal transducer and activator of transcription 3	Stat3	-1.61	0.010
1460326_at	phosphatidylinositol 3-kinase, catalytic, alpha polypeptide	Pik3ca	-1.61	0.000
1440134_at	cytochrome P450, family 4, subfamily a, polypeptide 10	Cyp4a10 /// BC01	-1.61	0.007
1439167_at	peroxisomal trans-2-enoyl-CoA reductase	Pecr	-1.61	0.004
1433723_s_at	Small EDRK-rich factor 2	Serf2	-1.61	0.002
1449460_at	ankyrin repeat and SOCS box-containing protein 13	Asb13	-1.62	0.001
1457641_at	CREB binding protein	Crebbp	-1.62	0.007
1424034_at	RAR-related orphan receptor alpha	Rora	-1.62	0.007
1419407_at	hemolytic complement	Hc	-1.62	0.000
1428087_at	dynamin 1-like	Dnm1l	-1.63	0.000
1420747_at	per-pentamer repeat gene	MGI:1349458	-1.63	0.002
1418019_at	carboxypeptidase D	Cpd	-1.63	0.001
1452960_at	SCY1-like 3 (S. cerevisiae)	Scyl3	-1.63	0.009
1448076_at	SH2 domain binding protein 1 (tetratricopeptide repeat containing)	Sh2bp1	-1.63	0.004
1433857_at	fat tumor suppressor homolog (Drosophila)	Fath	-1.63	0.009
1452915_at	protein kinase, cAMP dependent regulatory, type II alpha	Prkar2a	-1.63	0.003
1419911_at	Coronin, actin binding protein 1C	Coro1c	-1.63	0.008
1422485_at	MAD homolog 4 (Drosophila)	Smad4	-1.63	0.010
1420361_at	solute carrier family 11 member 1	Slc11a1	-1.63	0.008
1427413_a_at	CUG triplet repeat, RNA binding protein 1	Cugbp1	-1.63	0.000
1441870_s_at	polycystic kidney disease 2	Pkd2	-1.63	0.000
1449086_at	ring finger protein 141	Rnf141	-1.63	0.003
1430869_a_at	hyaluronic acid binding protein 4	Habp4	-1.64	0.009
1452860_at	F-box and leucine-rich repeat protein 17	Fbxl17	-1.64	0.002
1456121_at	leucine-rich repeats and IQ motif containing 2	Lrriq2	-1.64	0.007
1457296_at	cartilage intermediate layer protein, nucleotide pyrophosphohydrolase	Cilp	-1.64	0.005
1449183_at	catechol-O-methyltransferase	Comt	-1.64	0.003
1438443_at	zinc finger and BTB domain containing 20	Zbtb20	-1.64	0.006
1419122_at	methyltransferase-like 1	Mettl1	-1.65	0.007
1450860_at	leucine aminopeptidase 3	Lap3	-1.65	0.001

1448431_at	ankyrin repeat and SOCS box-containing protein 6	Asb6	-1.65	0.007
1423215_at	signal peptidase complex subunit 2 homolog (S. cerevisiae)	Spcs2	-1.65	0.008
1419279_at	phosphatidylinositol-4-phosphate 5-kinase, type II, alpha	Pip5k2a	-1.65	0.003
1455849_at	neuron navigator 1	Nav1	-1.65	0.008
1417426_at	proteoglycan 1, secretory granule	Prg1	-1.65	0.000
1425188_s_at	Sel1 (suppressor of lin-12) 1 homolog (C. elegans)	Sel1h	-1.65	0.007
1428729_at	cerebral cavernous malformations 1	Ccm1	-1.66	0.003
1436059_at	regulatory factor X, 1 (influences HLA class II expression)	Rfx1	-1.66	0.007
1415952_at	MAP/microtubule affinity-regulating kinase 2	Mark2	-1.66	0.001
1453949_s_at	lysophospholipase 1	Lypla1	-1.66	0.007
1434939_at	forkhead box F1a	Foxf1a	-1.66	0.007
1419552_at	enoyl Coenzyme A hydratase domain containing 1	Echdc1	-1.66	0.000
1420897_at	synaptosomal-associated protein 23	Snap23	-1.66	0.000
1417713_at	eukaryotic translation initiation factor 2, subunit 2 (beta)	Eif2s2	-1.67	0.003
1450725_s_at	carbonic anhydrase 14	Car14	-1.67	0.006
1433592_at	calmodulin 1	Calm1	-1.67	0.000
1422578_at	citrate synthase	Cs	-1.67	0.000
1450843_a_at	serine (or cysteine) proteinase inhibitor, clade H, member 1	Serpinh1	-1.67	0.006
1424178_at	transmembrane protein 38a	Tmem38a	-1.67	0.005
1422293_a_at	potassium channel tetramerisation domain containing 1	Kctd1	-1.68	0.009
1417244_a_at	interferon regulatory factor 7	Irf7	-1.68	0.005
1436739_at	angiotensin receptor 1	Agtr1	-1.68	0.009
1455567_at	CDC2-related kinase 7	Crk7	-1.68	0.004
1416412_at	neutral sphingomyelinase (N-SMase) activation associated factor	Nsmaf	-1.68	0.004
1435751_at	ATP-binding cassette, sub-family C (CFTR/MRP), member 9	Abcc9	-1.69	0.007
1423462_at	mitogen-activated protein kinase kinase kinase 7 interacting protein 2	Map3k7ip2	-1.69	0.001
1451131_at	ADP-ribosylation factor-like 6 interacting protein 1	Arl6ip1	-1.69	0.007
1452740_at	myosin, heavy polypeptide 10, non-muscle	Myh10	-1.70	0.005
1454930_at	leucine rich repeat containing 35	Lrrc35	-1.70	0.000
1419061_at	ras homolog gene family, member D	Rhod	-1.70	0.002
1417231_at	claudin 2	Cldn2	-1.70	0.001
1431105_a_at	transmembrane protein 33	Tmem33	-1.71	0.004
1420920_a_at	ADP-ribosylation factor 1	Arf1	-1.71	0.003
1415874_at	sprouty homolog 1 (Drosophila)	Spry1	-1.71	0.000
1422661_at	lectin, galactose binding, soluble 8	Lgals8	-1.71	0.004
1439153_at	IBR domain containing 2	Ibrdc2	-1.71	0.003

1423006_at	proviral integration site 1	Pim1	-1.71	0.004
1448318_at	adipose differentiation related protein	Adfp	-1.72	0.004
1448493_at	polyadenylate-binding protein-interacting protein 2	Paip2	-1.72	0.000
1426529_a_at	transgelin 2	Tagln2	-1.72	0.009
1447187_at	Receptor interacting protein kinase 5	Ripk5	-1.72	0.007
1435136_at	Wolf-Hirschhorn syndrome candidate 1 (human)	Whsc1	-1.73	0.008
1416256_a_at	tubulin, beta 5	Tubb5	-1.73	0.003
1438320_s_at	minichromosome maintenance deficient 7 (S. cerevisiae)	Mcm7	-1.74	0.000
1416762_at	S100 calcium binding protein A10 (calpactin)	S100a10	-1.74	0.009
1449107_at	nudix (nucleoside diphosphate linked moiety X)-type motif 4	Nudt4	-1.74	0.000
1429183_at	plakophilin 2	Pkp2	-1.74	0.000
1436868_at	reticulon 4 receptor-like 1	Rtn4rl1	-1.74	0.005
1449164_at	CD68 antigen	Cd68	-1.75	0.000
1445374_at	Vesicle transport through interaction with t-SNAREs homolog 1A (yeast)	Vti1a	-1.75	0.007
1451231_a_at	cullin 2	Cul2	-1.75	0.001
1441981_at	transmembrane protease, serine 11f	Tmprss11f	-1.75	0.006
1417673_at	growth factor receptor bound protein 14	Grb14	-1.75	0.000
1449970_at	calpain 12	Capn12	-1.76	0.007
1448304_a_at	RAB6, member RAS oncogene family	Rab6	-1.76	0.001
1426441_at	solute carrier family 11 member 2	Slc11a2	-1.76	0.006
1428631_a_at	ubiquinol cytochrome c reductase core protein 2	Uqcrc2	-1.76	0.000
1446302_at	Cysteine conjugate-beta lyase 1	Ccbl1	-1.76	0.007
1460316_at	Acyl-CoA synthetase long-chain family member 1	Acsl1	-1.77	0.000
1446571_at	Mblk1-related protein-1	Mlr1	-1.77	0.001
1419913_at	Serine/threonine kinase receptor associated protein	Strap	-1.77	0.001
1421720_a_at	deltex 2 homolog (Drosophila)	Dtx2	-1.77	0.009
1452091_a_at	RNA binding motif protein 28	Rbm28	-1.78	0.003
1455967_at	Sorbin and SH3 domain containing 1	Sorbs1	-1.78	0.008
1416494_at	NADH dehydrogenase (ubiquinone) Fe-S protein 5	Ndufs5	-1.78	0.002
1416275_at	solute carrier family 26, member 6	Slc26a6	-1.78	0.010
1437629_at	Rho guanine nucleotide exchange factor (GEF) 19	Arhgef19	-1.78	0.002
1438787_at	zinc finger protein 206	Zfp206	-1.79	0.006
1455438_at	Peroxisomal membrane protein 4	Pxmp4	-1.79	0.004
1415853_at	differentially expressed in FDCP 8	Def8	-1.79	0.001
1416462_at	GPI-anchored membrane protein 1	Gpiap1	-1.79	0.001
1436380_at	Cdc42 binding protein kinase alpha	Cdc42bpa	-1.79	0.000

1436753_at	aarF domain containing kinase 5	Adck5	-1.79	0.002
1448529_at	thrombomodulin	Thbd	-1.79	0.004
1436023_at	BCL2-associated transcription factor 1	Bclaf1	-1.80	0.007
1455771_at	benzodiazapine receptor associated protein 1	Bzrap1	-1.80	0.006
1423760_at	CD44 antigen	Cd44	-1.80	0.002
1422041_at	paired immunoglobin-like type 2 receptor beta	Pilrb	-1.81	0.009
1418655_at	UDP-N-acetyl-alpha-D-galactosamine	Galgt1	-1.81	0.000
1457793_a_at	Wolf-Hirschhorn syndrome candidate 1-like 1 (human)	Whsc1l1	-1.81	0.004
1418949_at	growth differentiation factor 15	Gdf15	-1.81	0.006
1427020_at	scavenger receptor class A, member 3	Scara3	-1.82	0.007
1433883_at	tropomyosin 4	Tpm4	-1.82	0.000
1420096_at	Zinc finger proliferation 1	Zipro1	-1.83	0.003
1416838_at	methylmalonyl-Coenzyme A mutase	Mut	-1.83	0.000
1460351_at	S100 calcium binding protein A11 (calizzarin)	S100a11	-1.83	0.002
1450222_x_at	nerve growth factor, alpha	Ngfa	-1.83	0.004
1445456_at	Potassium channel modulatory factor 1	Kcmf1	-1.83	0.000
1450061_at	ectodermal-neural cortex 1	Enc1	-1.83	0.007
1417624_at	Ngfi-A binding protein 1	Nab1	-1.83	0.001
1429779_at	eukaryotic translation initiation factor 2C, 4	Eif2c4	-1.83	0.001
1449734_s_at	Bardet-Biedl syndrome 4 homolog (human)	Bbs4	-1.83	0.001
1419300_at	FMS-like tyrosine kinase 1	Flt1	-1.83	0.003
1429187_at	transmembrane emp24 protein transport domain containing 7	Tmed7	-1.83	0.004
1422909_at	SMC6 structural maintenance of chromosomes 6-like 1 (yeast)	Smc6l1	-1.84	0.004
1438390_s_at	pituitary tumor-transforming 1	Pttg1	-1.84	0.000
1450966_at	carnitine O-octanoyltransferase	Crot	-1.84	0.000
1424140_at	galactose-4-epimerase, UDP	Gale	-1.85	0.006
1460653_at	spinocerebellar ataxia 2 homolog (human)	Sca2	-1.85	0.007
1426556_at	suppressor of hairy wing homolog 4 (Drosophila)	Suhw4	-1.86	0.003
1437812_x_at	alpha glucosidase 2 alpha neutral subunit	Ganab	-1.86	0.001
1436994_a_at	histone 1, H1c	Hist1h1c	-1.86	0.002
1434834_at	suppressor of cytokine signaling 7	Socs7	-1.87	0.001
1456042_s_at	T-complex expressed gene 4	Tce4	-1.87	0.003
1448617_at	CD53 antigen	Cd53	-1.87	0.007
1419931_at	ATP-binding cassette, sub-family B (MDR/TAP), member 7	Abcb7	-1.87	0.000
1439123_at	PHD finger protein 21A	Bhc80	-1.88	0.001
1450783_at	interferon-induced protein with tetratricopeptide repeats 1	Ifit1	-1.88	0.009

1417266_at	chemokine (C-C motif) ligand 6	Ccl6	-1.88	0.010
1416194_at	cytochrome P450, family 4, subfamily b, polypeptide 1	Cyp4b1	-1.89	0.002
1419436_at	complement component factor h-like 1	Cfhl1	-1.89	0.000
1416049_at	glycine decarboxylase	Gldc	-1.89	0.000
1417203_at	ethylmalonic encephalopathy 1	Ethe1	-1.90	0.001
1418754_at	adenylate cyclase 8	Adcy8	-1.90	0.007
1438370_x_at	downstream of Stk11	Dos	-1.90	0.008
1416411_at	glutathione S-transferase, mu 2	Gstm2	-1.90	0.000
1423140_at	lysosomal acid lipase 1	Lip1	-1.90	0.001
1435155_at	cingulin	Cgn	-1.90	0.006
1419179_at	thioredoxin-like 4	Txnl4	-1.90	0.006
1436827_at	gene model 944, (NCBI)	Gm944	-1.90	0.006
1420117_at	Golgi phosphoprotein 3	Golph3	-1.90	0.001
1437161_x_at	RNA binding protein gene with multiple splicing	Rbpms	-1.91	0.004
1418301_at	interferon regulatory factor 6	Irf6	-1.91	0.001
1456667_at	Huntington disease gene homolog	Hdh	-1.91	0.001
1455001_x_at	Ribosomal protein L13a	Rpl13a	-1.92	0.000
1425619_s_at	desmoglein 2	Dsg2	-1.93	0.009
1449105_at	SH2 domain protein 2A	Sh2d2a	-1.93	0.004
1417891_at	T-complex expressed gene 1	Tce1	-1.93	0.003
1437295_at	protein kinase N2	Pkn2	-1.93	0.004
1449954_at	hyaluronidase 1 /// N-acetyltransferase 6	Hyal1 /// Nat6	-1.94	0.003
1428509_at	myosin IE	Myo1e	-1.95	0.000
1443421_s_at	protocadherin beta 15	Pcdhb15	-1.95	0.006
1420615_at	ash2 (absent, small, or homeotic)-like (Drosophila)	Ash2l	-1.95	0.002
1451899_a_at	general transcription factor II I repeat domain-containing 1	Gtf2ird1	-1.95	0.000
1438699_at	Steroid 5 alpha-reductase 1	Srd5a1	-1.96	0.003
1451749_at	interleukin-1 receptor-associated kinase 4	Irak4	-1.96	0.001
1433053_at	integrin beta 2-like	Itgb2l	-1.96	0.005
1419044_at	contactin associated protein 4	Cntnap4	-1.98	0.004
1419406_a_at	B-cell CLL/lymphoma 11A (zinc finger protein)	Bcl11a	-1.99	0.006
1449888_at	endothelial PAS domain protein 1	Epas1	-1.99	0.000
1424855_at	thioesterase domain containing 1	Thedc1	-1.99	0.007
1420924_at	tissue inhibitor of metalloproteinase 2	Timp2	-1.99	0.000
1418243_at	ficolin A	Fcna	-1.99	0.000
1449934_at	purine rich element binding protein A	Pura	-2.00	0.001

1422411_s_at	eosinophil-associated, ribonuclease A family, member 1	Ear1 /// Ear2 /// E	-2.00	0.007
1458426_at	Kinesin family member 1B	Kif1b	-2.00	0.003
1452735_at	PEST-containing nuclear protein	Pcnp	-2.00	0.001
1428660_s_at	torsin family 3, member A	Tor3a	-2.00	0.002
1458410_at	GTPase activating RANGAP domain-like 1	Garnl1	-2.01	0.004
1451847_s_at	AT rich interactive domain 4B (Rbp1 like)	Arid4b	-2.01	0.006
1452045_at	zinc finger protein 281	Zfp281	-2.02	0.004
1451018_at	leptin receptor overlapping transcript-like 1	Leprotl1	-2.02	0.004
1416321_s_at	proline arginine-rich end leucine-rich repeat	Prelp	-2.02	0.001
1450857_a_at	procollagen, type I, alpha 2	Col1a2	-2.02	0.007
1439638_at	Erbb2 interacting protein	Erbb2ip	-2.03	0.005
1460592_at	erythrocyte protein band 4.1-like 1	Epb4.1l1	-2.04	0.003
1444913_at	Hedgehog acyltransferase	Hhat	-2.04	0.008
1452324_at	plasmacytoma variant translocation 1	Pvt1	-2.04	0.003
1416530_a_at	purine-nucleoside phosphorylase	Pnp	-2.05	0.001
1443029_at	PTPRF interacting protein, binding protein 1 (liprin beta 1)	Ppfibp1	-2.05	0.004
1421028_a_at	myocyte enhancer factor 2C	Mef2c	-2.05	0.007
1458566_at	G patch domain containing 2	Gpatc2	-2.05	0.001
1436764_at	par-3 (partitioning defective 3) homolog (C. elegans)	Pard3	-2.06	0.006
1452391_at	coxsackievirus and adenovirus receptor	Cxadr	-2.06	0.008
1425567_a_at	annexin A5	Anxa5	-2.07	0.001
1419449_a_at	guanine nucleotide binding protein, alpha inhibiting 2	Gnai2	-2.07	0.000
1450004_at	thymic stromal lymphopoietin	Tslp	-2.07	0.008
1451139_at	solute carrier family 39 (zinc transporter), member 4	Slc39a4	-2.08	0.002
1458850_at	Myosin, heavy polypeptide 7, cardiac muscle, beta	Myh7	-2.08	0.009
1435647_at	inhibitor of kappaB kinase gamma	Ikbkg	-2.08	0.000
1450984_at	tight junction protein 2	Tjp2	-2.09	0.003
1447411_at	UDP-glucose dehydrogenase	Ugdh	-2.09	0.008
1441677_at	SMC4 structural maintenance of chromosomes 4-like 1 (yeast)	Smc4l1	-2.09	0.004
1417612_at	immediate early response 5	Ier5	-2.09	0.002
1449846_at	eosinophil-associated, ribonuclease A family, member 2	Ear2	-2.11	0.005
1460225_at	mitochondrial ribosomal protein 63	Mrp63	-2.11	0.003
1434314_s_at	RAB11 family interacting protein 5 (class I)	Rab11fip5	-2.11	0.009
1418456_a_at	chemokine (C-X-C motif) ligand 14	Cxcl14	-2.13	0.003
1460348_at	MAD2 mitotic arrest deficient-like 2 (yeast)	Mad2l2	-2.13	0.001
1417501_at	F-box only protein 6b	Fbxo6b	-2.15	0.004

1417023_a_at	fatty acid binding protein 4, adipocyte	Fabp4	-2.15	0.000
1418659_at	circadian locomoter output cycles kaput	Clock	-2.15	0.005
1460323_at	threonyl-tRNA synthetase	Tars	-2.15	0.003
1416199_at	kinesin family member C3	Kifc3	-2.16	0.006
1417292_at	interferon gamma inducible protein 47	Ifi47	-2.16	0.009
1450156_a_at	hyaluronan mediated motility receptor (RHAMM)	Hmmr	-2.16	0.009
1430802_at	histocompatibility 2, Q region locus 8	H2-Q8	-2.16	0.000
1419483_at	complement component 3a receptor 1	C3ar1	-2.17	0.004
1452381_at	cAMP responsive element binding protein 3-like 2	Creb3l2	-2.17	0.008
1431240_at	C-type lectin domain family 2, member h	Clec2h	-2.17	0.009
1449379_at	kinase insert domain protein receptor	Kdr	-2.17	0.004
1452907_at	galactosylceramidase	Galc	-2.18	0.010
1418090_at	plasmalemma vesicle associated protein	Plvap	-2.18	0.006
1438421_at	poliovirus receptor-related 1	Pvrl1	-2.18	0.007
1417689_a_at	PDZK1 interacting protein 1	Pdzk1ip1	-2.20	0.004
1429653_at	genetic suppressor element 1	Gse1	-2.20	0.009
1426140_x_at	killer cell lectin-like receptor, subfamily A, member 19	Klra19	-2.21	0.009
1427201_at	musculoskeletal, embryonic nuclear protein 1	Mustn1	-2.21	0.001
1440141_at	potassium voltage-gated channel, subfamily Q, member 2	Kcnq2	-2.22	0.001
1439196_at	hook homolog 3 (Drosophila)	Hook3	-2.22	0.001
1420688_a_at	sarcoglycan, epsilon	Sgce	-2.24	0.009
1435541_at	betacellulin, epidermal growth factor family member	Btc	-2.24	0.004
1430255_at	Kruppel-like factor 5	Klf5	-2.24	0.010
1425951_a_at	C-type lectin domain family 4, member n	Clec4n	-2.26	0.005
1444502_at	PET112-like (yeast)	Pet112l	-2.26	0.004
1434603_at	thyroid hormone receptor associated protein 2	Thrap2	-2.26	0.002
1424857_a_at	tripartite motif protein 34	Trim34	-2.26	0.002
1457126_at	myosin, light polypeptide 4	Myl4	-2.26	0.009
1419847_at	Protein serine kinase H1	AW539964	-2.26	0.002
1449403_at	phosphodiesterase 9A	Pde9a	-2.27	0.008
1449135_at	SRY-box containing gene 18	Sox18	-2.27	0.000
1460546_at	leucine-rich repeat LGI family, member 3	Lgi3	-2.27	0.004
1418990_at	membrane-spanning 4-domains, subfamily A, member 4D	Ms4a4d	-2.28	0.002
1448380_at	lectin, galactoside-binding, soluble, 3 binding protein	Lgals3bp	-2.28	0.000
1433893_s_at	sperm associated antigen 5	Spag5	-2.29	0.006
1438468_at	preferentially expressed antigen in melanoma like 4	Pramel4 /// Pram	-2.29	0.003

1433634_at	interferon regulatory factor 2 binding protein 2	Irf2bp2	-2.31	0.006
1459324_at	Pleckstrin homology domain containing, family C member 1	Plekhc1	-2.31	0.001
1428822_a_at	sorting nexing 24	Snx24	-2.31	0.003
1458715_at	Caspase 6	Casp6	-2.32	0.006
1451956_a_at	opioid receptor, sigma 1	Oprs1	-2.33	0.008
1454137_s_at	hemochromatosis type 2 (juvenile) (human homolog)	Hfe2	-2.34	0.000
1438372_at	myomesin 2	Myom2	-2.34	0.006
1453128_at	extra cellular link domain-containing 1	Xlkd1	-2.34	0.002
1438084_at	A disintegrin and metalloprotease domain 23	Adam23	-2.34	0.002
1455493_at	synaptic nuclear envelope 1	Syne1	-2.34	0.000
1440641_at	Vacuolar protein sorting 29 (S. pombe)	Vps29	-2.35	0.005
1425071_s_at	neurotrophic tyrosine kinase, receptor, type 3	Ntrk3	-2.35	0.001
1420982_at	RNA-binding region (RNP1, RRM) containing 2	Rnpc2	-2.35	0.003
1447491_at	Leucine rich repeat containing 5	Lrrc5	-2.36	0.001
1441017_at	zinc finger, CCHC domain containing 14	Zcchc14	-2.36	0.009
1443362_at	Zinc finger protein 277	Zfp277	-2.37	0.004
1437889_x_at	biglycan	Bgn	-2.38	0.002
1457724_at	cathepsin L	Ctsl	-2.39	0.001
1425709_at	ring finger protein 17	Rnf17	-2.40	0.007
1422151_at	opposite strand transcription unit to Stag3	Gats	-2.40	0.002
1433578_at	hypothetical protein E130304D01	E130304D01	-2.41	0.006
1418809_at	paired-Ig-like receptor A1	Pira1	-2.41	0.002
1436319_at	sulfatase 1	Sulf1	-2.41	0.003
1433745_at	triple functional domain (PTPRF interacting)	Trio	-2.41	0.004
1417961_a_at	tripartite motif protein 30	Trim30	-2.43	0.000
1428168_at	myelin protein zero-like 1	Mpzl1	-2.45	0.001
1443595_at	CCR4-NOT transcription complex, subunit 6-like	Cnot6l	-2.45	0.008
1417066_at	chaperone, ABC1 activity of bc1 complex like (S. pombe)	Cabc1	-2.46	0.002
1435760_at	cystatin A	MGI:3524930	-2.46	0.005
1417859_at	growth arrest specific 7	Gas7	-2.47	0.008
1425745_a_at	transforming, acidic coiled-coil containing protein 2	Tacc2	-2.48	0.003
1442760_x_at	Reticulon 4	Rtn4	-2.48	0.004
1452231_x_at	interferon activated gene 203	Ifi203	-2.48	0.003
1428230_at	protein kinase C, nu	Prkcn	-2.48	0.000
1439240_x_at	lin 7 homolog b (C. elegans)	Lin7b	-2.49	0.005
1436781_at	mannosidase 2, alpha B1	Man2b1	-2.51	0.003

1418623_at	RAB2, member RAS oncogene family	Rab2	-2.52	0.002
1448261_at	cadherin 1	Cdh1	-2.53	0.000
1417982_at	insulin induced gene 2	Insig2	-2.53	0.000
1457012_at	developing brain homeobox 1	Dbx1	-2.55	0.001
1441344_at	SPFH domain family, member 1	Spfh1	-2.55	0.004
1420910_at	phosphatidic acid phosphatase type 2c	Ppap2c	-2.55	0.006
1459769_at	Hypothetical gene supported by AK076800; BC048609	LOC433215	-2.55	0.009
1434502_x_at	solute carrier family 4 (anion exchanger), member 1	Slc4a1	-2.56	0.005
1429296_at	RAB10, member RAS oncogene family	Rab10	-2.56	0.007
1423555_a_at	interferon-induced protein 44	Ifi44	-2.56	0.000
1456080_a_at	Tumor differentially expressed 1	Tde1	-2.57	0.001
1420534_at	guanylate cyclase 1, soluble, alpha 3	Gucy1a3	-2.57	0.003
1415965_at	stearoyl-Coenzyme A desaturase 1	Scd1	-2.57	0.000
1450202_at	glutamate receptor, ionotropic, NMDA1 (zeta 1)	Grin1	-2.57	0.004
1432181_s_at	endothelial cell growth factor 1 (platelet-derived)	Ecgf1	-2.59	0.001
1434252_at	transmembrane and coiled coil domains 3	Tmcc3	-2.59	0.000
1456494_a_at	tripartite motif protein 30 /// tripartite motif protein 30-like	Trim30 /// LOC20	-2.59	0.000
1422423_at	melanoma antigen, family A, 7	Magea7	-2.60	0.001
1417885_at	microtubule-associated protein tau	Mapt	-2.60	0.008
1418950_at	dopamine receptor 2	Drd2	-2.62	0.001
1429806_at	Era (G-protein)-like 1 (E. coli)	Eral1	-2.65	0.004
1456046_at	complement component 1, q subcomponent, receptor 1	C1qr1	-2.66	0.000
1449872_at	heat shock protein 3	Hspb3	-2.67	0.001
1443698_at	F-box protein 39	Fbxo39	-2.68	0.002
1426433_at	myc target 1	Myct1	-2.69	0.000
1452035_at	procollagen, type IV, alpha 1	Col4a1	-2.70	0.000
1427676_a_at	Glutamate receptor, ionotropic, kainate 1	Grik1	-2.70	0.000
1436545_at	deltex 4 homolog (Drosophila)	Dtx4	-2.71	0.007
1443048_at	F-box only protein 21	Fbxo21	-2.72	0.001
1416304_at	LPS-induced TN factor	Litaf	-2.73	0.000
1426664_x_at	prostate cancer associated protein 6	Pcanap6	-2.74	0.000
1455293_at	gene model 185, (NCBI)	Gm185	-2.75	0.010
1457608_at	GTPase, IMAP family member 8	Gimap8	-2.76	0.004
1453100_at	casein kinase II, alpha 2, polypeptide	Csnk2a2	-2.76	0.003
1457762_at	Tetratricopeptide repeat domain 15	Ttc15	-2.77	0.001
1422254_a_at	dual-specificity tyrosine-(Y)-phosphorylation regulated kinase 1b	Dyrk1b	-2.77	0.005

1449309_at	cytochrome P450, family 8, subfamily b, polypeptide 1	Cyp8b1	-2.78	0.001
1454159_a_at	insulin-like growth factor binding protein 2	Igfbp2	-2.78	0.000
1444171_at	Programmed cell death 4	Pdcd4	-2.78	0.002
1434087_at	5,10-methylenetetrahydrofolate reductase	Mthfr	-2.79	0.002
1452783_at	fibronectin type III domain containing 3B	Fndc3b	-2.79	0.000
1440026_at	Trichorhinophalangeal syndrome I (human)	Trps1	-2.79	0.003
1420426_at	myosin VIIb	Myo7b	-2.80	0.003
1425505_at	myosin, light polypeptide kinase	Mylk	-2.80	0.000
1449007_at	B-cell translocation gene 3	Btg3	-2.82	0.001
1443169_at	CCR4-NOT transcription complex, subunit 4	Cnot4	-2.83	0.009
1421769_at	LIM homeobox transcription factor 1 beta	Lmx1b	-2.87	0.000
1455099_at	monoacylglycerol O-acyltransferase 2	Mogat2	-2.88	0.009
1418746_at	brain protein 17	MGI:1930773	-2.90	0.003
1442316_x_at	transformation related protein 53 binding protein 1	Trp53bp1	-2.91	0.005
1417040_a_at	Bcl-2-related ovarian killer protein	Bok	-2.94	0.000
1449368_at	decorin	Dcn	-2.95	0.000
1437801_at	LIM domain containing preferred translocation partner in lipoma	Lpp	-2.97	0.002
1426732_at	desmin	Des	-2.97	0.000
1418449_at	ladinin	Lad1	-2.98	0.002
1438063_at	M-phase phosphoprotein 9	Mphosph9	-2.99	0.009
1453571_at	DEP domain containing 6	Depdc6	-2.99	0.000
1431056_a_at	lipoprotein lipase	Lpl	-2.99	0.002
1435775_at	TPA regulated locus	Tparl	-3.00	0.000
1458617_at	protein kinase C binding protein 1	Prkcbp1	-3.00	0.000
1443795_at	transcription factor 19	Tcf19	-3.04	0.001
1421548_at	protocadherin beta 2	Pcdhb2	-3.04	0.001
1452968_at	collagen triple helix repeat containing 1	Cthrc1	-3.05	0.001
1433691_at	protein phosphatase 1, regulatory (inhibitor) subunit 3C	Ppp1r3c	-3.05	0.000
1453681_at	ATPase inhibitory factor 1	Atpif1	-3.08	0.001
1438404_at	ring finger protein 144	Rnf144	-3.10	0.003
1416589_at	secreted acidic cysteine rich glycoprotein	Sparc	-3.10	0.000
1442086_at	Metastasis associated 3	Mta3	-3.11	0.008
1418191_at	ubiquitin specific protease 18	Usp18	-3.12	0.000
1451597_at	transmembrane protease, serine 11d	Tmprss11d	-3.14	0.007
1426278_at	interferon, alpha-inducible protein 27	Ifi27	-3.14	0.001
1421678_at	inositol 1,4,5-triphosphate receptor 2	Itpr2	-3.16	0.001

1436068_at	zinc finger and BTB domain containing 10	Zbtb10	-3.17	0.007
1453528_at	leukotriene A4 hydrolase	Lta4h	-3.19	0.005
1437218_at	fibronectin 1	Fn1	-3.21	0.000
1452250_a_at	procollagen, type VI, alpha 2	Col6a2	-3.21	0.003
1450484_a_at	thymidylate kinase family LPS-inducible member	Tyki	-3.21	0.000
1417311_at	cysteine rich protein 2	Crip2	-3.23	0.003
1419703_at	procollagen, type V, alpha 3	Col5a3	-3.27	0.001
1456767_at	leucine rich repeat and fibronectin type III domain containing 3	Lrfn3	-3.34	0.006
1436058_at	radical S-adenosyl methionine domain containing 2	Rsad2	-3.38	0.005
1437400_at	neural precursor cell expressed, developmentally down-regulated gene 4-like	Nedd4l	-3.41	0.001
1427829_at	ATP-binding cassette, sub-family D (ALD), member 4	Abcd4	-3.43	0.002
1459860_x_at	tripartite motif protein 2	Trim2	-3.43	0.000
1417880_at	glucose-6-phosphatase, catalytic	G6pc	-3.48	0.000
1436778_at	cytochrome b-245, beta polypeptide	Cybb	-3.52	0.000
1421765_at	paired box gene 5	Pax5	-3.57	0.001
1442927_at	PTK2 protein tyrosine kinase 2 beta	Ptk2b	-3.58	0.000
1429298_at	dimethylarginine dimethylaminohydrolase 1	Ddah1	-3.59	0.004
1428372_at	suppression of tumorigenicity 5	St5	-3.63	0.000
1417843_s_at	EPS8-like 2	Eps8l2	-3.71	0.001
1441995_at	Neural cell adhesion molecule 1	Ncam1	-3.75	0.000
1447394_at	chloride channel calcium activated 6	Clca6	-3.77	0.009
1458641_at	Braf transforming gene	Braf	-3.78	0.004
1436181_at	development and differentiation enhancing factor 2	Ddef2	-3.79	0.000
1451538_at	SRY-box containing gene 9	Sox9	-3.80	0.007
1441704_at	Pleckstrin homology domain containing, family A member 5	Plekha5	-3.85	0.003
1445522_at	ATPase, class I, type 8B, member 4	Atp8b4	-4.02	0.000
1423447_at	caseinolytic protease X (E.coli)	Clpx	-4.05	0.008
1418440_at	procollagen, type VIII, alpha 1	Col8a1	-4.38	0.008
1437609_at	similar to hypothetical protein	LOC381534	-4.60	0.003
1459353_at	Potassium channel tetramerisation domain containing 3	Kctd3	-4.64	0.004
1439479_at	lactase	Lct	-4.79	0.000
1430015_at	peroxisomal, testis specific 1	Pxt1	-4.86	0.007
1438294_at	spinocerebellar ataxia 1 homolog (human)	Sca1	-5.26	0.000
1444438_at	calcium and integrin binding family member 3	Cib3	-5.88	0.003
1450264_a_at	choline kinase alpha	Chka	-5.91	0.004
1420722_at	elongation of very long chain fatty acids-like 3	Elovl3	-6.25	0.001

1418918_at	insulin-like growth factor binding protein 1	Igfbp1	-7.75	0.007
1425099_a_at	aryl hydrocarbon receptor nuclear translocator-like	Arntl	-12.98	0.005
1448612_at	stratifin	Sfn	-2.6	0.005
1424857_a_at	tripartite motif protein 34	Trim34	-2.6	0.000
1454902_at	protein kinase C, zeta	Prkcz	-2.6	0.009
1429883_at	actin-like 6A	Actl6a	-2.6	0.009
1419483_at	complement component 3a receptor 1	C3ar1	-2.6	0.002
1437762_at	RAB39, member RAS oncogene family	Rab39	-2.6	0.003
1443340_at	Calcium binding atopy-related autoantigen 1	Cbara1	-2.6	0.003
1425948_a_at	solute carrier family 25, member 30	Slc25a30	-2.7	0.002
1417066_at	chaperone, ABC1 activity of bc1 complex like (S. pombe)	Cabc1	-2.7	0.001
1440360_at	ADP-ribosylation factor-like 6 interacting protein 5	Arl6ip5	-2.7	0.004
1442697_at	Importin 11	Ipo11	-2.7	0.003
1428230_at	protein kinase C, nu	Prkcn	-2.7	0.000
1449135_at	SRY-box containing gene 18	Sox18	-2.7	0.000
1438121_at	SEC24 related gene family, member A (S. cerevisiae)	Sec24a	-2.7	0.008
1433745_at	triple functional domain (PTPRF interacting)	Trio	-2.7	0.000
1427551_at	ubiquitin specific protease 29	Usp29	-2.7	0.007
1448696_at	hephaestin	Heph	-2.7	0.001
1420688_a_at	sarcoglycan, epsilon	Sgce	-2.7	0.000
1427357_at	cytidine deaminase	Cda	-2.7	0.008
1417982_at	insulin induced gene 2	Insig2	-2.7	0.000
1452907_at	galactosylceramidase	Galc	-2.7	0.000
1418456_a_at	chemokine (C-X-C motif) ligand 14	Cxcl14	-2.7	0.008
1428167_a_at	myelin protein zero-like 1	Mpzl1	-2.7	0.006
1455493_at	synaptic nuclear envelope 1	Syne1	-2.7	0.000
1457231_at	Hypoxia inducible factor 1, alpha subunit	Hif1a	-2.7	0.001
1418990_at	membrane-spanning 4-domains, subfamily A, member 4D	Ms4a4d	-2.7	0.005
1415965_at	stearoyl-Coenzyme A desaturase 1	Scd1	-2.7	0.001
1447198_at	RecQ protein-like	Recql	-2.8	0.008
1459856_at	Mitochondrial ribosomal protein L13	Mrpl13	-2.8	0.003
1460225_at	mitochondrial ribosomal protein 63	Mrp63	-2.8	0.002
1427201_at	musculoskeletal, embryonic nuclear protein 1	Mustn1	-2.8	0.002
1444693_at	Calcium channel, voltage-dependent, beta 2 subunit	Cacnb2	-2.8	0.003
1417961_a_at	tripartite motif protein 30	Trim30	-2.8	0.000
1434314_s_at	RAB11 family interacting protein 5 (class I)	Rab11fip5	-2.8	0.003

1432181_s_at	endothelial cell growth factor 1 (platelet-derived)	Ecgf1	-2.8	0.000
1425745_a_at	transforming, acidic coiled-coil containing protein 2	Tacc2	-2.8	0.007
1433053_at	integrin beta 2-like	Itgb2l	-2.8	0.002
1418293_at	interferon-induced protein with tetratricopeptide repeats 2	Ifit2	-2.8	0.009
1448261_at	cadherin 1	Cdh1	-2.8	0.000
1443421_s_at	protocadherin beta 15	Pcdhb15	-2.8	0.009
1418603_at	arginine vasopressin receptor 1A	Avpr1a	-2.8	0.007
1456080_a_at	Tumor differentially expressed 1	Tde1	-2.8	0.000
1419044_at	contactin associated protein 4	Cntnap4	-2.8	0.003
1449105_at	SH2 domain protein 2A	Sh2d2a	-2.8	0.000
1421028_a_at	myocyte enhancer factor 2C	Mef2c	-2.8	0.003
1448834_at	forkhead box M1	Foxm1	-2.8	0.009
1439660_at	human immunodeficiency virus type I enhancer binding protein 3	Hivep3	-2.9	0.004
1444127_at	Ankyrin repeat and SOCS box-containing protein 1	Asb1	-2.9	0.007
1453128_at	extra cellular link domain-containing 1	Xlkd1	-2.9	0.005
1420982_at	RNA-binding region (RNP1, RRM) containing 2	Rnpc2	-2.9	0.002
1435541_at	betacellulin, epidermal growth factor family member	Btc	-2.9	0.004
1433147_at	caldesmon 1	Cald1	-2.9	0.006
1452764_at	suppressor of cytokine signaling 6	Socs6	-2.9	0.009
1446674_at	Mitogen activated protein kinase kinase 5	Map2k5	-2.9	0.000
1428396_at	SMAD specific E3 ubiquitin protein ligase 1	Smurf1	-2.9	0.005
1454159_a_at	insulin-like growth factor binding protein 2	Igfbp2	-2.9	0.000
1453077_a_at	small nuclear RNA activating complex, polypeptide 3	Snapc3	-2.9	0.000
1426318_at	serine (or cysteine) proteinase inhibitor, clade B, member 1b	Serpinb1b	-2.9	0.006
1429653_at	genetic suppressor element 1	Gse1	-2.9	0.000
1427993_at	RUN and FYVE domain-containing 2	Rufy2	-2.9	0.010
1416023_at	fatty acid binding protein 3, muscle and heart	Fabp3	-2.9	0.001
1438939_x_at	Necdin	Ndn	-2.9	0.004
1453224_at	zinc finger, A20 domain containing 2	Za20d2	-2.9	0.007
1446329_at	G kinase anchoring protein 1	Gkap1	-2.9	0.003
1419406_a_at	B-cell CLL/lymphoma 11A (zinc finger protein)	Bcl11a	-2.9	0.002
1457688_at	zinc finger protein 398	Zfp398	-2.9	0.002
1420615_at	ash2 (absent, small, or homeotic)-like (Drosophila)	Ash2l	-3.0	0.002
1443362_at	Zinc finger protein 277	Zfp277	-3.0	0.000
1441536_at	3-hydroxy-3-methylglutaryl-Coenzyme A synthase 1	Hmgcs1	-3.0	0.006
1417153_at	BTB (POZ) domain containing 14A	Btbd14a	-3.0	0.003

1459942_at	Open reading frame 34	ORF34	-3.0	0.004
1436500_at	Ribosomal protein S24	Rps24	-3.0	0.002
1420561_at	transient receptor potential cation channel, subfamily C, member 7	Trpc7	-3.0	0.006
1445918_at	Transmembrane protein 2	Tmem2	-3.0	0.000
1449309_at	cytochrome P450, family 8, subfamily b, polypeptide 1	Cyp8b1	-3.0	0.001
1438084_at	A disintegrin and metalloprotease domain 23	Adam23	-3.0	0.000
1446311_at	membrane bound C2 domain containing protein	Mbc2	-3.0	0.003
1425631_at	protein phosphatase 1, regulatory (inhibitor) subunit 3C	Ppp1r3c	-3.0	0.000
1421609_a_at	camello-like 3	Cml3	-3.1	0.010
1435061_at	nudix (nucleoside diphosphate linked moiety X)-type motif 11	Nudt11	-3.1	0.008
1425951_a_at	C-type lectin domain family 4, member n	Clec4n	-3.1	0.002
1442381_at	Muskelin 1, intracellular mediator containing kelch motifs	Mkln1	-3.1	0.001
1440641_at	Vacuolar protein sorting 29 (S. pombe)	Vps29	-3.1	0.001
1445105_at	P300/CBP-associated factor	Pcaf	-3.1	0.001
1434252_at	transmembrane and coiled coil domains 3	Tmcc3	-3.1	0.000
1418803_a_at	Fas ligand (TNF superfamily, member 6)	Fasl	-3.1	0.009
1444502_at	PET112-like (yeast)	Pet112l	-3.1	0.007
1456494_a_at	tripartite motif protein 30 /// tripartite motif protein 30-like	Trim30 /// LOC209387	-3.1	0.000
1442035_at	cholinergic receptor, nicotinic, alpha polypeptide 5	Chrna5	-3.1	0.006
1418623_at	RAB2, member RAS oncogene family	Rab2	-3.1	0.001
1446107_at	Serine/threonine kinase 24 (STE20 homolog, yeast)	Stk24	-3.1	0.002
1422327_s_at	glucose-6-phosphate dehydrogenase 2	G6pd2 /// G6pdx	-3.1	0.006
1426997_at	thyroid hormone receptor alpha	Thra	-3.1	0.005
1428051_a_at	calcium channel, voltage-dependent, L type, alpha 1D subunit	Cacna1d	-3.1	0.008
1443349_at	Dedicator of cytokinesis 10	Dock10	-3.2	0.003
1459154_at	Eyes absent 3 homolog (Drosophila)	Eya3	-3.2	0.001
1418746_at	brain protein 17	MGI:1930773	-3.2	0.000
1449368_at	decorin	Dcn	-3.2	0.000
1442192_at	thymidylate synthase	Tyms	-3.2	0.005
1456046_at	complement component 1, q subcomponent, receptor 1	C1qr1	-3.2	0.000
1423555_a_at	interferon-induced protein 44	Ifi44	-3.2	0.000
1458715_at	Caspase 6	Casp6	-3.2	0.000
1453755_at	U7 snRNP-specific Sm-like protein LSM11	Lsm11	-3.2	0.001
1451063_at	syntaxin binding protein 4	Stxbp4	-3.2	0.004

1440647_at	signal-induced proliferation-associated 1 like 1	Sipa1l1	-3.2	0.007
1453571_at	DEP domain containing 6	Depdc6	-3.2	0.000
1429806_at	Era (G-protein)-like 1 (E. coli)	Eral1	-3.2	0.000
1457126_at	myosin, light polypeptide 4	Myl4	-3.2	0.001
1455293_at	gene model 185, (NCBI)	Gm185	-3.2	0.001
1426664_x_at	prostate cancer associated protein 6	Pcanap6	-3.2	0.000
1443698_at	F-box protein 39	Fbxo39	-3.2	0.004
1450156_a_at	hyaluronan mediated motility receptor (RHAMM)	Hmmr	-3.2	0.001
1447445_at	Sidekick homolog 2 (chicken)	Sdk2	-3.2	0.010
1446167_at	Homeodomain interacting protein kinase 2	Hipk2	-3.2	0.000
1425505_at	myosin, light polypeptide kinase	Mylk	-3.2	0.000
1456354_at	cholinergic receptor, nicotinic, alpha polypeptide 4	Chrna4	-3.2	0.004
1448551_a_at	tripartite motif protein 2	Trim2	-3.3	0.004
1427095_at	CUB domain containing protein 1	Cdcp1	-3.3	0.001
1426140_x_at	killer cell lectin-like receptor, subfamily A, member 19	Klra19	-3.3	0.006
1418809_at	paired-Ig-like receptor A1	Pira1	-3.3	0.002
1442760_x_at	Reticulon 4	Rtn4	-3.3	0.001
1434087_at	5,10-methylenetetrahydrofolate reductase	Mthfr	-3.3	0.000
1438468_at	preferentially expressed antigen in melanoma like 4	Pramel4 /// Pramel5	-3.3	0.000
1443540_at	mitogen activated protein kinase kinase kinase 1	Map3k1	-3.3	0.001
1441017_at	zinc finger, CCHC domain containing 14	Zcchc14	-3.3	0.000
1420729_at	2-cell-stage, variable group, member 1	MGI:1933437	-3.3	0.005
1430255_at	Kruppel-like factor 5	Klf5	-3.3	0.000
1449007_at	B-cell translocation gene 3	Btg3	-3.3	0.002
1457369_at	Phosphatase and actin regulator 4	Phactr4	-3.3	0.001
1429402_at	glycosyltransferase 8 domain containing 2	Glt8d2	-3.3	0.007
1460546_at	leucine-rich repeat LGI family, member 3	Lgi3	-3.4	0.004
1417859_at	growth arrest specific 7	Gas7	-3.4	0.000
1437801_at	LIM domain containing preferred translocation partner in lipoma	Lpp	-3.4	0.002
1440141_at	potassium voltage-gated channel, subfamily Q, member 2	Kcnq2	-3.4	0.005
1441852_x_at	APG16 autophagy 16-like (S. cerevisiae)	Apg16l	-3.4	0.007
1451889_at	Notch gene homolog 2 (Drosophila)	Notch2	-3.4	0.007
1416589_at	secreted acidic cysteine rich glycoprotein	Sparc	-3.4	0.000
1457762_at	Tetratricopeptide repeat domain 15	Ttc15	-3.5	0.003
1417896_at	tight junction protein 3	Tjp3	-3.5	0.007

1435760_at	cystatin A	MGI:3524930	-3.5	0.000
1417040_a_at	Bcl-2-related ovarian killer protein	Bok	-3.5	0.000
1441344_at	SPFH domain family, member 1	Spfh1	-3.5	0.001
1439240_x_at	lin 7 homolog b (C. elegans)	Lin7b	-3.5	0.009
1417311_at	cysteine rich protein 2	Crip2	-3.5	0.001
1458381_at	chloride intracellular channel 5	Clic5	-3.5	0.001
1419847_at	Protein serine kinase H1	AW539964	-3.5	0.001
1418449_at	ladinin	Lad1	-3.5	0.002
1422423_at	melanoma antigen, family A, 7	Magea7	-3.5	0.002
1443048_at	F-box only protein 21	Fbxo21	-3.5	0.000
1419339_at	neuraminidase 3	Neu3	-3.5	0.003
1422254_a_at	dual-specificity tyrosine-(Y)-phosphorylation regulated kinase 1b	Dyrk1b	-3.6	0.010
1431319_at	unc-51-like kinase 4 (C. elegans)	Ulk4	-3.6	0.009
1425709_at	ring finger protein 17	Rnf17	-3.6	0.004
1448228_at	lysyl oxidase	Lox	-3.6	0.001
1457012_at	developing brain homeobox 1	Dbx1	-3.6	0.001
1418950_at	dopamine receptor 2	Drd2	-3.6	0.000
1457608_at	GTPase, IMAP family member 8	Gimap8	-3.6	0.000
1452017_at	SRY-box containing gene 15	Sox15	-3.6	0.004
1437218_at	fibronectin 1	Fn1	-3.6	0.000
1438404_at	ring finger protein 144	Rnf144	-3.7	0.000
1433133_at	EDAR (ectodysplasin-A receptor)-associated death domain	Edaradd	-3.7	0.009
1418191_at	ubiquitin specific protease 18	Usp18	-3.7	0.000
1431263_at	protein kinase, AMP-activated, gamma 2 non-catalytic subunit	Prkag2	-3.7	0.004
1427702_at	zinc finger protein 1	Zfp1	-3.7	0.004
1431056_a_at	lipoprotein lipase	Lpl	-3.7	0.005
1417880_at	glucose-6-phosphatase, catalytic	G6pc	-3.7	0.000
1421561_at	basic helix-loop-helix domain containing, class B4	Bhlhb4	-3.8	0.005
1420534_at	guanylate cyclase 1, soluble, alpha 3	Gucy1a3	-3.8	0.002
1426278_at	interferon, alpha-inducible protein 27	Ifi27	-3.8	0.002
1426294_at	hyaluronan and proteoglycan link protein 1	Hapln1	-3.8	0.005
1436319_at	sulfatase 1	Sulf1	-3.8	0.007
1419703_at	procollagen, type V, alpha 3	Col5a3	-3.9	0.002
1420426_at	myosin VIIb	Myo7b	-3.9	0.008
1456767_at	leucine rich repeat and fibronectin type III domain containing 3	Lrfn3	-3.9	0.000
1453041_at	Trp53 inducible protein 5	Trp53i5	-3.9	0.005

1450202_at	glutamate receptor, ionotropic, NMDA1 (zeta 1)	Grin1	-3.9	0.001
1419726_at	a disintegrin and metalloprotease domain 18	Adam18	-3.9	0.003
1429298_at	dimethylarginine dimethylaminohydrolase 1	Ddah1	-3.9	0.003
1422978_at	cytochrome b-245, beta polypeptide	Cybb	-3.9	0.002
1434502_x_at	solute carrier family 4 (anion exchanger), member 1	Slc4a1	-4.0	0.001
1455099_at	monoacylglycerol O-acyltransferase 2	Mogat2	-4.0	0.001
1427676_a_at	Glutamate receptor, ionotropic, kainate 1	Grik1	-4.0	0.000
1459026_at	SKI interacting protein	Skiip	-4.0	0.000
1449514_at	G protein-coupled receptor kinase 5	Gprk5	-4.1	0.009
1443630_at	Protein phosphatase 3, catalytic subunit, alpha isoform	Ppp3ca	-4.1	0.008
1444171_at	Programmed cell death 4	Pdcd4	-4.1	0.001
1428372_at	suppression of tumorigenicity 5	St5	-4.1	0.000
1436068_at	zinc finger and BTB domain containing 10	Zbtb10	-4.1	0.006
1452250_a_at	procollagen, type VI, alpha 2	Col6a2	-4.1	0.001
1421769_at	LIM homeobox transcription factor 1 beta	Lmx1b	-4.2	0.000
1417843_s_at	EPS8-like 2	Eps8l2	-4.2	0.004
1451375_at	ets homologous factor	Ehf	-4.2	0.002
1426433_at	myc target 1	Myct1	-4.3	0.000
1457551_at	Activin A receptor, type 1	Acvr1	-4.3	0.006
1453681_at	ATPase inhibitory factor 1	Atpif1	-4.3	0.000
1435756_at	sterile alpha motif domain containing 10	Samd10	-4.4	0.004
1449872_at	heat shock protein 3	Hspb3	-4.4	0.006
1424950_at	SRY-box containing gene 9	Sox9	-4.4	0.007
1423447_at	caseinolytic protease X (E.coli)	Clpx	-4.4	0.006
1436181_at	development and differentiation enhancing factor 2	Ddef2	-4.5	0.000
1453528_at	leukotriene A4 hydrolase	Lta4h	-4.5	0.009
1436593_at	Unknown (protein for MGC:58818)	RP23-96I9.2	-4.6	0.008
1451597_at	transmembrane protease, serine 11d	Tmprss11d	-4.6	0.002
1437932_a_at	claudin 1	Cldn1	-4.6	0.005
1426732_at	desmin	Des	-4.6	0.002
1444294_at	5-oxoprolinase (ATP-hydrolysing)	Oplah	-4.6	0.008
1443795_at	transcription factor 19	Tcf19	-4.6	0.001
1424811_at	camello-like 5	Cml5	-4.7	0.007
1421678_at	inositol 1,4,5-triphosphate receptor 2	Itpr2	-4.7	0.003
1421548_at	protocadherin beta 2	Pcdhb2	-4.8	0.000
1417860_a_at	spondin 2, extracellular matrix protein	Spon2	-5.0	0.001

1425544_at	pleckstrin homology domain containing, family A member 5	Plekha5	-5.0	0.006
1452968_at	collagen triple helix repeat containing 1	Cthrc1	-5.2	0.009
1442927_at	PTK2 protein tyrosine kinase 2 beta	Ptk2b	-5.2	0.000
1451906_at	UBX domain containing 3	Ubxd3	-5.3	0.001
1447394_at	chloride channel calcium activated 6	Clca6	-5.5	0.007
1427829_at	ATP-binding cassette, sub-family D (ALD), member 4	Abcd4	-5.7	0.005
1441995_at	Neural cell adhesion molecule 1	Ncam1	-5.7	0.000
1445111_at	SEC8-like 1 (S. cerevisiae)	Sec8l1	-5.8	0.001
1421765_at	paired box gene 5	Pax5	-6.0	0.000
1438294_at	spinocerebellar ataxia 1 homolog (human)	Sca1	-6.2	0.000
1450264_a_at	choline kinase alpha	Chka	-6.7	0.001
1420722_at	elongation of very long chain fatty acids-like 3	Elovl3	-6.8	0.001
1445522_at	ATPase, class I, type 8B, member 4	Atp8b4	-6.9	0.001
1436207_at	Transcription factor 3	Tcf3	-7.0	0.003
1444438_at	calcium and integrin binding family member 3	Cib3	-7.4	0.001
1459353_at	Potassium channel tetramerisation domain containing 3	Kctd3	-7.4	0.006
1439479_at	lactase	Lct	-7.8	0.000
1430015_at	peroxisomal, testis specific 1	Pxt1	-7.9	0.001
1418918_at	insulin-like growth factor binding protein 1	Igfbp1	-8.6	0.005
