# Supplementary material for: Impaired Genome Maintenance Suppresses the Growth Hormone–Insulin-Like Growth Factor 1 Axis in Mice with Cockayne Syndrome
Source: PLoS Biol. 2006 Dec 12;5(1):e2. doi: 10.1371/journal.pbio.0050002 (PMC1698505; doi:10.1371/journal.pbio.0050002)
Supplement: Supplementary file 6 [file pbio.0050002.st006.pdf]

Supplementary Table S6

Extensive overview of gene expression profiles associated with significantly overrepresented biological processes in 96- and 130-week old naturally aged mice. FC: fold change, p: p-value.

Code	Title	Symbol	130W			96W		
			FC	p*	p**	FC	p*	p**
**IGF1/GH Somatotroph axis and additional mitogenic/growth signals**								
1419519_at	insulin-like growth factor 1	Igf1	-1.48	0.009	0.005	-1.44	0.011	0.006
1434413_at	Insulin-like growth factor 1	Igf1	-1.36	0.005	0.003	-1.34	0.002	0.001
1437401_at	Insulin-like growth factor 1	Igf1	-1.37	0.001	0.000	-1.23	0.010	0.005
1417933_at	insulin-like growth factor binding protein 6	Igfbp6	-1.74	0.000	0.000	-1.14	0.217	0.109
1454159_a_at	insulin-like growth factor binding protein 2	Igfbp2	-2.22	0.012	0.006	-1.60	0.010	0.005
1451871_a_at	Growth hormone receptor	Ghr	-2.37	0.002	0.001	1.06	0.371	0.186
1423136_at	fibroblast growth factor 1	Fgf1	-1.91	0.008	0.004	-1.15	0.020	0.010
1450282_at	fibroblast growth factor 4	Fgf4	-1.67	0.015	0.007	1.10	0.451	0.225
1450869_at	fibroblast growth factor 1	Fgf1	-2.42	0.003	0.001	-1.38	0.016	0.008
1460652_at	Estrogen related receptor, alpha	Esrra	-1.51	0.009	0.004	1.02	0.914	0.457
1415882_at	growth hormone inducible transmembrane protein	Ghitm	-1.38	0.002	0.001	-1.18	0.005	0.003
1415888_at	hepatoma-derived growth factor	Hdgf	-2.38	0.010	0.005	1.01	0.966	0.483
1417658_at	transforming growth factor beta regulated gene 4	Tbrg4	-1.76	0.016	0.008	-1.20	0.256	0.128
1420894_at	transforming growth factor, beta receptor I	Tgfbr1	-3.50	0.007	0.004	2.44	0.012	0.006
1420909_at	vascular endothelial growth factor A	Vegfa	-1.91	0.017	0.008	1.28	0.099	0.049
1422826_at	insulin-like growth factor binding protein, acid labile subunit	Igfals	-2.28	0.009	0.005	-1.22	0.224	0.112
1432181_s_at	endothelial cell growth factor 1 (platelet-derived)	Ecgf1	-3.34	0.000	0.000	-3.64	0.000	0.000
1450177_at	nerve growth factor receptor (TNFR superfamily, member 16)	Ngfr	-2.83	0.006	0.003	1.04	0.846	0.423
1460420_a_at	epidermal growth factor receptor	Egfr	-2.23	0.008	0.004	-1.66	0.016	0.008
Cytochrome (Cyt) P450, NADH-and NADPH-dependent Oxidative metabolism								
**NADH oxidative metabolism**								
1416194_at	cytochrome P450, family 4, subfamily b, polypeptide 1	Cyp4b1	-1.39	0.006	0.003	-1.20	0.064	0.032
1417070_at	cytochrome P450, family 4, subfamily v, polypeptide 3	Cyp4v3	-2.51	0.004	0.002	-2.56	0.001	0.001
1418767_at	cytochrome P450, family 4, subfamily f, polypeptide 13	Cyp4f13	-1.98	0.016	0.008	-1.64	0.018	0.009
1419559_at	cytochrome P450, family 4, subfamily f, polypeptide 14	Cyp4f14	-2.09	0.018	0.009	-1.39	0.003	0.002
1421075_s_at	cytochrome P450, family 7, subfamily b, polypeptide 1	Cyp7b1	-2.63	0.018	0.009	-2.12	0.047	0.024
1422534_at	cytochrome P450, family 51	Cyp51	-2.59	0.005	0.003	1.22	0.321	0.160

1424352_at	similar to cytochrome P450, 4a10	MGC25972	-4.04	0.006	0.003	-3.25	0.008	0.004
1425365_a_at	cytochrome P450, family 2, subfamily d, polypeptide 13	Cyp2d13	-2.51	0.016	0.008	-1.87	0.009	0.004
1432081_at	cytochrome P450, family 1, subfamily b, polypeptide 1	Cyp1b1	-2.77	0.017	0.009	1.39	0.330	0.165
1440134_at	cytochrome P450, family 4, subfamily a, polypeptide 10	Cyp4a10	-1.91	0.012	0.006	-1.54	0.024	0.012
1444138_at	cytochrome P450, family 2, subfamily r, polypeptide 1	Cyp2r1	-1.89	0.016	0.008	-1.17	0.004	0.002
1444960_at	cytochrome P450, family 2, subfamily u, polypeptide 1	Cyp2u1	-2.28	0.012	0.006	-1.41	0.096	0.048
1449309_at	cytochrome P450, family 8, subfamily b, polypeptide 1	Cyp8b1	-5.20	0.000	0.000	-3.76	0.000	0.000
1450715_at	cytochrome P450, family 1, subfamily a, polypeptide 2	Cyp1a2	-2.48	0.002	0.001	-2.36	0.000	0.000
1453500_at	cytochrome P450, family 2, subfamily u, polypeptide 1	Cyp2u1	-3.74	0.003	0.002	-1.49	0.223	0.111
1415967_at	NADH dehydrogenase (ubiquinone) flavoprotein 1	Ndufv1	-1.46	0.008	0.004	-1.35	0.001	0.001
1416366_at	NADH dehydrogenase (ubiquinone) 1, subcomplex unknown, 2	Ndufc2	-1.48	0.001	0.000	-1.22	0.028	0.014
1416417_a_at	NADH dehydrogenase (ubiquinone) 1 beta subcomplex, 7	Ndufb7	-1.20	0.018	0.009	-1.51	0.002	0.001
1416547_at	NADH dehydrogenase (ubiquinone) 1 beta subcomplex 3	Ndufb3	-1.67	0.008	0.004	-1.56	0.000	0.000
1417102_a_at	NADH dehydrogenase (ubiquinone) 1 beta subcomplex, 5	Ndufb5	-1.74	0.004	0.002	-1.91	0.000	0.000
1422185_a_at	diaphorase 1 (NADH)	Dia1	-1.57	0.009	0.005	-1.15	0.121	0.061
1422186_s_at	diaphorase 1 (NADH)	Dia1	-1.86	0.001	0.000	-1.27	0.067	0.034
1422241_a_at	NADH dehydrogenase (ubiquinone) 1 alpha subcomplex, 1	Ndufa1	-1.27	0.005	0.002	-1.36	0.002	0.001
1423737_at	NADH dehydrogenase (ubiquinone) Fe-S protein 3	Ndufs3	-1.69	0.003	0.001	-1.71	0.000	0.000
1423908_at	NADH dehydrogenase (ubiquinone) Fe-S protein 8	Ndufs8	-1.43	0.017	0.008	-1.31	0.031	0.015
1424085_at	NADH dehydrogenase (ubiquinone) 1 alpha subcomplex, 4	Ndufa4	-1.52	0.019	0.009	-1.29	0.025	0.013
1425143_a_at	NADH dehydrogenase (ubiquinone) Fe-S protein 1	Ndufs1	-1.67	0.010	0.005	-1.45	0.003	0.001
1428159_s_at	NADH dehydrogenase (ubiquinone) 1, alpha/beta subcomplex, 1	Ndufab1	-1.56	0.010	0.005	-1.46	0.001	0.000
1428179_at	NADH dehydrogenase (ubiquinone) flavoprotein 2	Ndufv2	-1.28	0.016	0.008	-1.73	0.000	0.000
1428322_a_at	NADH dehydrogenase (ubiquinone) 1 beta subcomplex, 10	Ndufb10	-1.79	0.004	0.002	-1.79	0.001	0.001
1428360_x_at	NADH dehydrogenase (ubiquinone) 1 alpha subcomplex, 7 (B14.5a)	Ndufa7	-1.19	0.016	0.008	-1.87	0.000	0.000
1433603_at	NADH dehydrogenase (ubiquinone) Fe-S protein 6	Ndufs6	-1.71	0.009	0.005	-1.59	0.000	0.000
1447919_x_at	NADH dehydrogenase (ubiquinone) 1, alpha/beta subcomplex, 1	Ndufab1	-2.48	0.010	0.005	-1.46	0.094	0.047
1448198_a_at	NADH dehydrogenase (ubiquinone) 1 beta subcomplex 8	Ndufb8	-1.34	0.004	0.002	-1.64	0.000	0.000
1448284_a_at	NADH dehydrogenase (ubiquinone) 1, subcomplex unknown, 1	Ndufc1	-1.55	0.018	0.009	-1.87	0.001	0.001
1448331_at	NADH dehydrogenase (ubiquinone) 1 beta subcomplex, 7	Ndufb7	-1.46	0.006	0.003	-1.63	0.000	0.000
1448427_at	NADH dehydrogenase (ubiquinone) 1 alpha subcomplex, 6 (B14)	Ndufa6	-1.53	0.018	0.009	-1.70	0.003	0.001
1448589_at	NADH dehydrogenase (ubiquinone) 1 beta subcomplex, 5	Ndufb5	-1.79	0.005	0.002	-1.65	0.000	0.000
1448959_at	NADH dehydrogenase (ubiquinone) Fe-S protein 4	Ndufs4	-1.56	0.013	0.007	-1.41	0.001	0.001
1451312_at	NADH dehydrogenase (ubiquinone) Fe-S protein 7	Ndufs7	-1.50	0.004	0.002	-1.63	0.000	0.000
1452692_a_at	NADH dehydrogenase (ubiquinone) flavoprotein 2	Ndufv2	-1.70	0.001	0.000	-1.70	0.000	0.000
1453565_at	NADH dehydrogenase (ubiquinone) 1, alpha/beta subcomplex, 1	Ndufab1	-2.77	0.005	0.002	-1.88	0.031	0.015

1455036_s_at	NADH dehydrogenase (ubiquinone) 1, subcomplex unknown, 2	Ndufc2	-1.24	0.014	0.007	-1.41	0.000	0.000
1455283_x_at	NADH dehydrogenase (ubiquinone) Fe-S protein 8	Ndufs8	-1.14	0.014	0.007	-1.86	0.000	0.000
1417429_at	flavin containing monooxygenase 1	Fmo1	-2.46	0.019	0.009	-1.64	0.077	0.038
1425799_at	flavin containing monooxygenase 4	Fmo4	-2.25	0.002	0.001	1.14	0.545	0.273
1428292_at	NADPH dependent diflavin oxidoreductase 1	Ndor1	-2.04	0.004	0.002	-1.08	0.514	0.257
**Peroxisomal biogenesis**								
1416259_at	peroxisomal biogenesis factor 12	Pex12	-1.84	0.001	0.000	-1.35	0.013	0.006
1419053_at	peroxisomal biogenesis factor 14	Pex14	-1.91	0.003	0.001	-1.01	0.909	0.455
1420711_a_at	peroxisomal membrane protein 3	Pxmp3	-1.70	0.001	0.001	-1.06	0.435	0.218
1422471_at	peroxisomal biogenesis factor 13	Pex13	-1.68	0.005	0.002	-1.48	0.000	0.000
1422472_at	peroxisomal biogenesis factor 13	Pex13	-1.86	0.010	0.005	-1.32	0.027	0.014
1422780_at	peroxisomal membrane protein 4	Pxmp4	-2.86	0.002	0.001	-1.59	0.005	0.003
1424078_s_at	peroxisomal biogenesis factor 6	Pex6	-1.92	0.012	0.006	-1.12	0.321	0.160
1451226_at	peroxisomal biogenesis factor 6	Pex6	-2.01	0.012	0.006	-1.11	0.447	0.224
1454044_a_at	peroxisomal biogenesis factor 3	Pex3	-1.68	0.005	0.002	-1.00	0.973	0.487
1455438_at	Peroxisomal membrane protein 4	Pxmp4	-2.40	0.009	0.005	-1.55	0.033	0.017
**Citric acid cycle**								
1415891_at	succinate-CoA ligase, GDP-forming, alpha subunit	Suclg1	-2.08	0.002	0.001	-1.58	0.000	0.000
1416478_a_at	malate dehydrogenase 2, NAD (mitochondrial)	Mdh2	-1.58	0.002	0.001	-1.17	0.077	0.038
1418885_a_at	isocitrate dehydrogenase 3 (NAD+) beta	Idh3b	-1.48	0.018	0.009	-1.60	0.001	0.000
1418886_s_at	isocitrate dehydrogenase 3 (NAD+) beta	Idh3b	-1.65	0.013	0.007	-1.63	0.002	0.001
1422577_at	citrate synthase	Cs	-1.74	0.000	0.000	-1.46	0.002	0.001
1422578_at	citrate synthase	Cs	-1.49	0.001	0.000	-1.43	0.000	0.000
1423644_at	aconitase 1	Aco1	-2.08	0.003	0.001	-1.26	0.072	0.036
1432016_a_at	isocitrate dehydrogenase 3 (NAD+) alpha	Idh3a	-1.47	0.001	0.000	1.08	0.243	0.122
1436934_s_at	aconitase 2, mitochondrial	Aco2	-1.47	0.001	0.000	-1.49	0.003	0.002
1447080_at	Aconitase 1	Aco1	-4.03	0.013	0.007	-1.17	0.539	0.270
1448630_a_at	succinate dehydrogenase complex, subunit C, integral membrane protein	Sdhc	-1.55	0.016	0.008	-1.27	0.009	0.004
1451002_at	aconitase 2, mitochondrial	Aco2	-1.54	0.000	0.000	-1.37	0.016	0.008
1452206_at	succinate-Coenzyme A ligase, ADP-forming, beta subunit	Sucla2	-1.68	0.015	0.008	-1.47	0.001	0.000
1456728_x_at	aconitase 1	Aco1	-2.20	0.009	0.005	-1.10	0.587	0.293
**Glycolysis**								
1415891_at	succinate-CoA ligase, GDP-forming, alpha subunit	Suclg1	-2.08	0.002	0.001	-1.58	0.000	0.000
1416183_a_at	lactate dehydrogenase 2, B chain	Ldh2	-1.73	0.011	0.006	-1.09	0.584	0.292
1416780_at	phosphofructokinase, muscle	Pfkm	-1.57	0.019	0.009	1.32	0.020	0.010
1418560_at	pyruvate dehydrogenase E1 alpha 1	Pdha1	-1.52	0.008	0.004	-1.42	0.008	0.004

1421258_a_at	pyruvate kinase liver and red blood cell	Pklr	-3.07	0.007	0.004	1.27	0.417	0.209
1421259_at	pyruvate kinase liver and red blood cell	Pklr	-4.76	0.009	0.004	1.73	0.107	0.054
1423159_at	dihydrolipoamide dehydrogenase	Dld	-1.67	0.002	0.001	-1.05	0.542	0.271
1432829_at	enolase 1, alpha non-neuron	Eno1	-2.31	0.018	0.009	1.36	0.227	0.114
1448237_x_at	lactate dehydrogenase 2, B chain	Ldh2	-2.13	0.004	0.002	-1.14	0.325	0.162
1449137_at	pyruvate dehydrogenase E1 alpha 1	Pdha1	-1.79	0.010	0.005	1.01	0.853	0.427
1451274_at	oxoglutarate dehydrogenase (lipoamide)	Ogdh	-1.78	0.000	0.000	-1.16	0.083	0.041
1452206_at	succinate-Coenzyme A ligase, ADP-forming, beta subunit	Sucla2	-1.68	0.015	0.008	-1.47	0.001	0.000
1416737_at	glycogen synthase 3, brain	Gys3	2.17	0.006	0.003	2.03	0.160	0.080
1417741_at	liver glycogen phosphorylase	Pygl	-1.83	0.005	0.002	-1.41	0.003	0.001

p-value*=two-tail distribution
p-value=one-tail distribution**
