# Supplementary material for: Impaired Genome Maintenance Suppresses the Growth Hormone–Insulin-Like Growth Factor 1 Axis in Mice with Cockayne Syndrome
Source: PLoS Biol. 2006 Dec 12;5(1):e2. doi: 10.1371/journal.pbio.0050002 (PMC1698505; doi:10.1371/journal.pbio.0050002)
Supplement: Supplementary file 7 [file pbio.0050002.st007.pdf]

Supplementary Table S7

Comparison of gene expression profiles associated with significantly overrepresented biological processes between Csb$^{m/m}$/Xpa$^{-/-}$, Csb$^{m/m}$, Xpa$^{-/-}$ mice and 96- and 130-week old naturally aged mice. FC: fold change, p: p-value.

Code	Title	Symbol	*Csb$^{m/m}$ Xpa$^{-/-}$*		130W			96W			*Xpa$^{-/-}$*		*Csb$^{m/m}$*	
			FC	p*	FC	p*	p**	FC	p*	p**	FC	p*	FC	p*
The IGF-1/GH growth axis														
1419519_at	insulin-like growth factor 1	Igf1	-2.13	0.000	-1.48	0.009	0.005	-1.44	0.011	0.006	-1.08	0.443	-1.13	0.335
1434413_at	Insulin-like growth factor 1	Igf1	-1.82	0.000	-1.36	0.005	0.003	-1.34	0.002	0.001	1.11	0.325	1.12	0.257
1437401_at	Insulin-like growth factor 1	Igf1	-1.43	0.001	-1.37	0.001	0.000	-1.23	0.010	0.005	-1.04	0.527	-1.03	0.680
1452014_a_at	insulin-like growth factor 1	Igf1	-2.02	0.000	-1.30	0.043	0.021	-1.17	0.056	0.028	1.25	0.028	1.04	0.657
1417962_s_at	Growth hormone receptor	Ghr	-1.53	0.000	-1.51	0.142	0.071	-1.69	0.002	0.001	1.03	0.700	1.10	0.198
1451501_a_at	Growth hormone receptor	Ghr	-1.51	0.001	-1.50	0.193	0.097	-1.84	0.009	0.005	1.11	0.168	1.02	0.816
1451871_a_at	Growth hormone receptor	Ghr	-2.21	0.012	-2.37	0.002	0.001	1.06	0.371	0.186	1.10	0.607	-1.05	0.826
1458832_at	Growth hormone receptor	Ghr	-1.53	0.038	-1.88	0.023	0.012	-2.84	0.001	0.001	-1.23	0.372	-1.51	0.037
1459948_at	Growth hormone receptor	Ghr	-1.08	0.642	-3.98	0.070	0.035	-3.67	0.077	0.039	-1.16	0.584	-1.32	0.226
1425853_s_at	prolactin receptor	Prlr	-1.87	0.004	-1.20	0.464	0.232	2.54	0.151	0.075	1.19	0.194	1.04	0.761
1448556_at	prolactin receptor	Prlr	-2.03	0.000	-1.13	0.806	0.403	-1.75	0.303	0.152	-1.25	0.103	-1.10	0.638
1450226_at	prolactin receptor	Prlr	-1.76	0.001	-1.17	0.542	0.271	2.79	0.140	0.070	1.21	0.062	1.02	0.813
1421841_at	fibroblast growth factor receptor 3	Fgfr3	-1.43	0.001	-2.66	0.032	0.016	1.20	0.473	0.237	-1.32	0.004	-1.12	0.122
1427777_x_at	fibroblast growth factor receptor 4	Fgfr4	-1.32	0.009	-1.97	0.088	0.044	1.44	0.108	0.054	-1.12	0.233	-1.19	0.039
1450869_at	fibroblast growth factor 1	Fgf1	-1.38	0.003	-2.42	0.003	0.001	-1.38	0.016	0.008	-1.20	0.033	-1.01	0.890
1417991_at	deiodinase, iodothyronine, type I	Dio1	-2.12	0.000	-1.69	0.086	0.043	-1.28	0.210	0.105	1.00	0.973	-1.04	0.751
1435663_at	estrogen receptor 1 (alpha)	Esr1	-1.91	0.001	-1.36	0.180	0.090	1.50	0.031	0.016	-1.12	0.416	-1.31	0.092
1460591_at	estrogen receptor 1 (alpha)	Esr1	-1.59	0.000	-1.07	0.768	0.384	1.41	0.046	0.023	-1.02	0.780	-1.02	0.772
1421991_a_at	insulin-like growth factor binding protein 4	Igfbp4	-1.74	0.000	-1.40	0.235	0.118	1.39	0.034	0.017	-1.07	0.528	-1.16	0.155
1421992_a_at	insulin-like growth factor binding protein 4	Igfbp4	-1.43	0.000	-1.16	0.462	0.231	1.06	0.263	0.131	-1.09	0.150	-1.08	0.183
Carbohydrate metabolism														
1419146_a_at	glucokinase	Gck	-6.59	0.004	-1.92	0.138	0.069	-1.02	0.953	0.476	1.16	0.570	-1.01	0.983
1425303_at	glucokinase	Gck	-4.80	0.004	-2.24	0.104	0.052	-1.18	0.589	0.295	-1.17	0.563	-1.04	0.903
1459522_s_at	glycogenin 1	Gyg1	1.26	0.014	1.37	0.438	0.219	1.10	0.644	0.322	1.05	0.585	1.03	0.732
1417741_at	liver glycogen phosphorylase	Pygl	-1.42	0.000	-1.83	0.005	0.002	-1.41	0.003	0.001	1.02	0.707	1.06	0.367
1423644_at	aconitase 1	Aco1	-1.26	0.002	-2.08	0.003	0.001	-1.26	0.072	0.036	1.05	0.457	-1.00	0.987
1456728_x_at	aconitase 1	Aco1	-1.15	0.016	-2.20	0.009	0.005	-1.10	0.587	0.293	-1.18	0.010	-1.06	0.432
1423644_at	aconitase 1	Aco1	-1.26	0.002	-2.08	0.003	0.001	-1.26	0.072	0.036	1.05	0.457	-1.00	0.987

1422577_at	citrate synthase	Cs	-1.28	0.006	-1.74	0.000	0.000	-1.46	0.002	0.001	1.16	0.042	-1.03	0.690
Cytochrome (Cyt) P450, NADH-and NADPH-dependent Oxidative metabolism														
1417070_at	Cyt. P450, family 4, subfamily v, polypeptide 3	Cyp4v3	-1.41	0.001	-2.51	0.004	0.002	-2.56	0.001	0.001	1.04	0.606	-1.09	0.354
1417531_at	Cyt. P450, family 2, subfamily j, polypeptide 5	Cyp2j5	-2.51	0.000	-1.59	0.051	0.026	-1.51	0.000	0.000	-1.02	0.812	-1.04	0.545
1417532_at	Cyt. P450, family 2, subfamily j, polypeptide 5	Cyp2j5	-3.08	0.000	-2.90	0.039	0.019	-5.47	0.012	0.006	-1.47	0.024	-1.31	0.213
1417651_at	Cyt. P450, family 2, subfamily c, polypeptide 29	Cyp2c29	-1.59	0.004	-1.28	0.485	0.242	-1.46	0.151	0.075	-1.37	0.024	-1.41	0.018
1418767_at	Cyt. P450, family 4, subfamily f, polypeptide 13	Cyp4f13	-1.67	0.008	-1.98	0.016	0.008	-1.64	0.018	0.009	-1.11	0.487	-1.40	0.046
1418821_at	Cyt. P450, family 2, subfamily a, polypeptide 12	Cyp2a12	-1.51	0.000	-1.42	0.085	0.042	-1.29	0.004	0.002	1.06	0.051	-1.06	0.195
1419559_at	Cyt. P450, family 4, subfamily f, polypeptide 14	Cyp4f14	-3.58	0.000	-2.09	0.018	0.009	-1.39	0.003	0.002	1.57	0.019	-1.00	0.980
1419590_at	Cyt. P450, family 2, subfamily b, polypeptide 9	Cyp2b9	-1.55	0.000	14.44	0.041	0.021	27.28	0.094	0.047	-1.06	0.385	-1.04	0.528
1422100_at	Cyt. P450, family 7, subfamily a, polypeptide 1	Cyp7a1	-2.36	0.006	-2.12	0.109	0.055	2.14	0.026	0.013	1.14	0.549	-1.39	0.202
1422257_s_at	Cyt. P450, family 2, subfamily b, polypeptide 10	Cyp2b10	-2.81	0.001	-3.04	0.294	0.147	-5.00	0.211	0.106	-1.40	0.110	-1.39	0.109
1424273_at	Cyt. P450, family 2, subfamily c, polypeptide 70	Cyp2c70	-1.56	0.000	-1.02	0.910	0.455	-1.27	0.160	0.080	-1.03	0.587	-1.12	0.065
1424576_s_at	Cyt. P450, family 2, subfamily c, polypeptide 44	Cyp2c44	1.75	0.000	-1.59	0.049	0.025	-1.46	0.008	0.004	1.12	0.298	1.04	0.777
1425365_a_at	Cyt. P450, family 2, subfamily d, polypeptide 13	Cyp2d13	-2.17	0.006	-2.51	0.016	0.008	-1.87	0.009	0.004	1.22	0.289	-1.14	0.494
1425645_s_at	Cyt. P450, family 2, subfamily b, polypeptide 10	Cyp2b10	-2.94	0.001	-2.35	0.410	0.205	-6.60	0.229	0.115	-1.29	0.209	-1.42	0.095
1431803_at	Cyt. P450, family 2, subfamily d, polypeptide 13	Cyp2d13	-2.05	0.001	-2.86	0.030	0.015	-1.62	0.115	0.057	-1.03	0.774	-1.12	0.404
1438743_at	Cyt. P450, family 7, subfamily a, polypeptide 1	Cyp7a1	-2.28	0.000	-1.37	0.653	0.326	1.85	0.037	0.019	-1.02	0.883	-1.51	0.052
1448792_a_at	Cyt. P450, family 2, subfamily f, polypeptide 2	Cyp2f2	-2.36	0.001	-1.42	0.271	0.136	-1.56	0.006	0.003	1.76	0.001	1.30	0.054
1449479_at	Cyt. P450, family 2, subfamily b, polypeptide 13	Cyp2b13	-2.24	0.002	9.53	0.108	0.054	7.00	0.133	0.067	-1.19	0.345	-1.27	0.180
1451787_at	Cyt. P450, family 2, subfamily b, polypeptide 10	Cyp2b10	-2.22	0.005	-2.23	0.437	0.218	-6.30	0.240	0.120	-1.16	0.426	-1.22	0.278
1423908_at	NADH dehydrogenase Fe-S protein 8	Ndufs8	-1.23	0.002	-1.43	0.017	0.008	-1.31	0.031	0.015	1.02	0.693	-1.04	0.394
1434212_at	NADH dehydrogenase Fe-S protein 8	Ndufs8	-1.56	0.000	-1.87	0.188	0.094	1.26	0.438	0.219	-1.18	0.041	-1.41	0.009
1434213_x_at	NADH dehydrogenase Fe-S protein 8	Ndufs8	-1.16	0.001	-1.31	0.021	0.011	-1.46	0.001	0.000	-1.04	0.234	-1.11	0.035
1448589_at	NADH dehydrogenase 1 beta subcomplex, 5	Ndufb5	-1.17	0.007	-1.79	0.005	0.002	-1.65	0.000	0.000	1.09	0.084	-1.00	0.986
1417429_at	flavin containing monooxygenase 1	Fmo1	-1.38	0.000	-2.46	0.019	0.009	-1.64	0.077	0.038	1.04	0.375	1.00	0.914
1422904_at	flavin containing monooxygenase 2	Fmo2	-4.51	0.008	-1.56	0.216	0.108	1.75	0.285	0.142	-2.17	0.056	-2.05	0.066
1422905_s_at	flavin containing monooxygenase 2	Fmo2	-3.23	0.001	-1.02	0.933	0.466	1.08	0.677	0.339	-2.48	0.002	-1.94	0.017
1449525_at	flavin containing monooxygenase 3	Fmo3	-14.19	0.004	-1.46	0.609	0.305	10.11	0.382	0.191	-2.03	0.077	-2.34	0.058
1453435_a_at	flavin containing monooxygenase 2	Fmo2	-2.46	0.000	-1.14	0.554	0.277	1.80	0.024	0.012	-1.51	0.009	-1.33	0.055
Peroxisomal biogenesis														
1419365_at	peroxisomal biogenesis factor 11a	Pex11a	-1.61	0.003	-1.80	0.075	0.038	1.22	0.370	0.185	-1.37	0.022	-1.13	0.358
1422471_at	peroxisomal biogenesis factor 13	Pex13	-1.16	0.000	-1.68	0.005	0.002	-1.48	0.000	0.000	1.07	0.081	-1.13	0.003
1424078_s_at	peroxisomal biogenesis factor 6	Pex6	-1.43	0.003	-1.92	0.012	0.006	-1.12	0.321	0.160	-1.13	0.196	-1.11	0.251
1448910_at	peroxisomal trans-2-enoyl-CoA reductase	Pecr	-1.38	0.009	-1.76	0.036	0.018	-1.39	0.020	0.010	1.14	0.144	1.09	0.299
1449442_at	peroxisomal biogenesis factor 11a	Pex11a	-1.78	0.010	-1.36	0.368	0.184	-2.13	0.001	0.001	-1.97	0.006	-1.60	0.055

1451213_at	peroxisomal biogenesis factor 11b	Pex11b	-1.24	0.010	-2.16	0.021	0.011	-1.13	0.452	0.226	-1.03	0.693	-1.05	0.518
1451226_at	peroxisomal biogenesis factor 6	Pex6	-1.40	0.000	-2.01	0.012	0.006	-1.11	0.447	0.224	-1.11	0.093	-1.13	0.059

*=Two-tail distribution

**=One-tail distribution
