# Supplementary material for: Impaired Genome Maintenance Suppresses the Growth Hormone–Insulin-Like Growth Factor 1 Axis in Mice with Cockayne Syndrome
Source: PLoS Biol. 2006 Dec 12;5(1):e2. doi: 10.1371/journal.pbio.0050002 (PMC1698505; doi:10.1371/journal.pbio.0050002)
Supplement: Supplementary file 8 [file pbio.0050002.sd001.doc]

Supplementary Methods

**Animals**

Since *Xpa^-/-^*, *Xpc^-/-^* and *Csb^m/m^* mice are fertile, *Csb^m/m^/Xpa^-/-^*, *Csb^m/m^/Xpa^-/-^*and *Xpa^-/-^/Xpc^-/-^* mice were obtained from crossings between homozygous/heterozygous double mutant animals (e.g. *Csb^m/m^/Xpa^+/-^* x *Csb^+/m^/Xpa^-/-^*) to increase the expected proportion of double-mutant mice from 1:16 to 1:4. *Csb^m/m^/Xpa^-/-^*/*p53^-/-^* triple-mutant mice were obtained in a similar way after the *Xpa* and *Csb* mutant alleles had first been crossed into a *p53*-deficient background. Mice were genotyped by PCR using a three-primer mix that amplifies both the wt and targeted allele in a single reaction (see supplementary section). Animals are housed at the Animal Resource Center (Erasmus University Medical Center) and the National Institute of Public Health and the Environment (RIVM), which operate in compliance with the “Animal Welfare Act” of the Dutch government, using the “Guide for the Care and Use of Laboratory Animals” as its standard. As required by Dutch law, formal permission to generate and use genetically modified animals was obtained from the responsible local and national authorities. All animal studies were approved by an independent Animal Ethical Committee (Dutch equivalent of the IACUC).

**Exposure to Ionizing Irradiation**

For the ionizing irradiation study, 19-day old *Csb^m/m^/Xpa^-/-^* and littermate control animals (n=4-6/genotype) were exposed with 10Gy. Animals were sacrificed 20 hours after exposure and eyes were processed for further analysis. For the isolation of the eyes, animals were anaesthetized by CO_2_ inhalation, followed by cervical dislocation. Eyes were marked on the nasal side with Alcian blue (5% Alcian blue in 96% ethanol), subsequently enucleated and fixed in 4 % paraformaldehyde in 0.1 M phosphate buffer. Paraffinized sections of 5 μm thickness were subjected to TUNEL staining (see methods), eyes were fixed in 4 % paraformaldehyde in 0.1 M phosphate buffer and embedded in paraffin.

**Macroscopic Examination / Blood Parameters**

Newborn *Csb^m/m^/Xpa^-/-^* mice and their littermates were observed and weighed daily between 11.00 and 13.00 hours. Animals were euthanized at defined time points (as indicated in the text) or when displaying evident worsening of health condition (indicative for imminent death). Internal organs were isolated and either fixed in 10% phosphate-buffered formalin and paraffin-embedded (histopathological analysis) or snap-frozen in liquid N_2_ (for transcription profiling by microarray analysis or quantitative PCR). In some cases animals were given an intra-peritoneal injection with bromo-deoxy-uridine (BrdU; 50 μg/gram bodyweight) 45 min. prior to euthanasia in order to measure cell proliferation.

Immunohistochemistry

Immunohistochemical procedures: Paraffin sections (5 μm) were dewaxed and rehydrated, followed by an antigen retrieval step, comprising 1 x 7 minute and 2 x 3 minute incubations of tissue sections with a 0.01M sodium citrate buffer in a 800 Watt microwave. Sections were washed in phosphate buffered saline (PBS) for 2 minutes. Endogenous peroxidase activity was blocked by incubating the sections for 30 minutes in PBS, containing 30% H_2_O_2_ and 12.5% sodium azide. Sections were washed in PBS (1 x 2 minutes) and PBS, containing 0.5% milk powder, and 0.15% glycine (PBS+ buffer, 2 x 2 minutes).

Immunohistochemical staining was performed by incubating sections with primary antibodies against PCNA (PC10, Abcam, dilution 1:1000) or BrdU (Bu20a, DAKO, dilution 1:100) in PBS+ (16 hr; 4 ^o^C). In case of BrdU, sections were first covered with 0.1M HCl for 60 minutes at 37 ^o^C and rinsed with PBS, prior to incubation with the primary antibody. Next, sections were washed in PBS+ (3 x 5 minutes) and incubated for 1 hour at room temperature with rat-anti-mouse Immunoglobulins antibody (1:1000) coupled to horseradish peroxidase (DAKO). After sections were washed in PBS+ (3 X 5 minutes) and PBS 1 x 2 minutes), color was developed for 8 minutes in 3, 3’-diaminobenzidine solution (DAKO Liquid DAB substrate-chromogen system), all according to the instructions of the manufacturer. Sections were counterstained in haematoxylin before dehydration and mounting. All histology images were acquired at 40x objective magnification on an Olympus BX40 microscope (London, United Kingdom), equipped with a CCD camera.

# **Citrate Synthase Enzyme Activity**

Frozen livers were transferred into ice-cold buffer (SHE) consisting of 250 mM sucrose, 10 mM HEPES, pH 7.4, and minced. Fresh SHE was added and homogenized by 12 passes in a tight-fitting glass/Teflon power-driven Potter-Elvejehem homogenizer. Citrate synthase activity of the homogenate was measured by monitoring the CoA-coupled conversion of Ellman’s reagent into TNB at 412 nm at 37ºC in the presence of 0.005% (v/v) Lubrol-WX to solubilize the mitochondrial inner membrane and expressed in mU as nmol 5-thio-2-nitrobenzoate (TNB)/min per mg protein. Protein concentrations were determined by the Bio-Rad DC protein assay (Bio-Rad Laboratories, Inc., Veenendaal, the Netherlands) with BSA as a standard.

# **Microarray Analysis**

## Standard procedures were used to obtain total RNA (Qiagen) from the liver of wt, *Xpa^-/-^*, *Csb^m/m^*, and *Csb^m/m^/Xpa^-/-^* mice (n=4) at postnatal day 15 and from the liver of 8-, 16-, 96- and 130-week old mice (n=4). Synthesis of double stranded cDNA and biotin labeled cRNA was performed according to the instructions of the manufacturer (Affymetrix, USA). Fragmented cRNA preparations were hybridized to full mouse genome oligonucleotide arrays (Affymetrix, mouse expression 430 V2.0 arrays), using Affymetrix hybridization Oven 640 (Affymetrix, USA), washed, and subsequently scanned on a GeneChip Scanner 3000 (Affymetrix, USA). Initial data extraction and normalization within each array was performed by means of the GCOS software (Affymetrix). By implementing the R statistical package that is based on an open-sourced language, data intensities were log transformed and normalized within and/or between groups of arrays by means of the quantile normalization method. For all 15-day old livers, normalization was performed within and between groups of mice based on the assumption that most genes do not change between the various genotypes (*Csb^m/m^, Xpa^-/-^, Csb^m/m^/Xpa^-/-^* and wt). We also took into account, however, that this assumption may not be true for expression data derived from very distinct groups of mice (e.g. 130-week old compared to 8-week old mice). In those cases, expression data were normalized within age groups. This approach was shown to minimize the variance within each group of mice, thereby increasing the statistical power between groups of mice. The two normalization approaches used in this study (i.e. within and between groups or only within groups) did not affect the Spearman’s rank correlation measures (see below) on the categorical data. Based on the quality report files generated by the manufacturer (Affymetrix, USA), the threshold background noise was set at 40. For each probe set, signals were considered to be valid when they were marked as “Present” and exhibit a signal higher than 40 in at least one microarray experiment. All probe sets with a signal below 40 were set to be equal to 40. Where appropriate, two-tail, pair wise analysis or two-way analysis of variance was employed by means of the Spotfire Decision Site software package 7.2 v10.0 (Spotfire Inc., MA, USA) to extract the statistically significant data from each of the four individual microarrays obtained for each genotype (wt, *Xpa^-/-^*, *Csb^m/m^*, and *Csb^m/m^/Xpa^-/-^* livers) as well as from the four microarrays in each of the three groups of naturally aged mice (16-, 96- and 130-week old mice, n=4) as compared to 8-week old mice. The criteria for significance were set at p≤0.010 and a ≥ ±1.2-fold change with the exception of glycogenin 1 where after Q-PCR the difference in expression (1.26-fold up regulation) was considered to be significant despite the slightly deviant p-value of 0.012 (the cut-off was set at 0.010). All correlations were calculated by Spearman’s rank correlation coefficient, a non-parametric correlation method and were based on categorical values derived from all significant probe sets (see above). For all correlations, significance was set at p ≤ 0.001.

# **Gene Ontology Classification and Overrepresentation of Biological Themes**

All significant gene entries were subjected to GO classification ([**http://www.geneontology.org**](http://www.geneontology.org/)). Significant over-representation of GO-classified biological processes was assessed by comparing the number of pertinent genes in a given biological process to the total number of the relevant genes printed on the array for that particular biological process (Fisher exact test, p≤0.01, False discovery rate (FDR) ≤0.1) using the publicly accessible software Ease (Hosack et al. 2003).

### **Quantitative PCR Evaluation**

Total RNA was isolated from liver, heart, kidney and spleen of wt, *Xpa^-/-^*, *Csb^m/m^*, *Csb^m/m^/Xpa^-/-^* mice at postnatal day 15 using a Total RNA isolation kit (Qiagen) as described by the manufacturer. Quantitative PCR (Q-PCR) was performed with a DNA Engine Opticon device according to the instructions of the manufacturer (MJ Research). Primer pair designed to generate intron-spanning products of 180-210bp were as follows: *Sod1*: 5’-GGG ACA ATA CAC AAG GCT GT-3’ and 5’-GCC AAT GAT GGA ATG CTC TC-3’; *Gsr1*: 5’- CCG CCT GAA CAC CAT CTA T -3’ and 5’-TTC CCA TTG ACT TCC ACC G-3’; *Ghr*: 5’-ATT CAC CAA GTG TCG TTC CC-3’ and 5’-TCC ATT CCT GGG TCC ATT CA-3’; *Igf1*: 5’-TGC TTG CTC ACC TTC ACC A-3’ and 5’-CAA CAC TCA TCC ACA ATG CC-3’; *Prlr*: 5’-GCA TCT TTC CAC CAG TTC CG-3’ and 5’-GCT CGT CCT CAT TGT CAT CC-3’; *Gstt2*: 5’-GCC CAA GTC CAC GAA TAC CT-3’ and 5’-CTC TGT TCC GTT CCA CCT TC-3’; *Ephx1*: 5’-CAG CCA AAG AAG ATG AGA GCA-3’ and 5’-AGC CAT AGT GGA AGC GAC T-3’; *Hmox1*: 5’-AAC ACT CTG GAG ATG ACA CCT-3’ and 5’-TGT GAG GGA CTC TGG TCT TTG-3’; *ApoA4*: 5’-CAC CGT TTC TTC TGA CTC CG-3’ and 5’-AAT CCC ACA CCA CAT TGG C-3’; *Igfbp3*: 5’-GTG ACC GAT TCC AAG TTC CA-3’ and 5’-TGT CCT CCA TTT CTC TGC GG-3’; *Dio1*: 5’-CCC TGG TGT TGA ACT TTG GC-3’ and 5’-TGA GGA AAT CGG CTG TGG A-3’. The generation of specific PCR products was confirmed by melting curve analysis (which measures product specificity by the decrease in fluorescence signal when the PCR product is denatured) and gel electrophoresis (using Roche Agarose MS for analyzing small PCR products). Each primer pair was tested with a logarithmic dilution of a cDNA mix to generate a linear standard curve (crossing point (CP) plotted versus log of template concentration), which was used to calculate the primer pair efficiency (E = 10^(-1/slope)^). Hypoxanthine guanine phosphoribosyltransferase1 (*Hprt-1*) mRNA was used as an external standard. For data analysis, the second derivative maximum method was applied: (E_1gene of interest_ ^ΔCP (cDNA of wt mice - cDNA of^ *^Xpa-/-^* ^or^ *^Csbm/m^* ^or^ *^Csbm/m/Xpa-/-^*^) gene of interest^)/ (E_hprt-1_ ^ΔCP (cDNA wt mice- cDNA of^ *^Xpa-/-^* ^or^ *^Csbm/m^* ^or^ *^Csbm/m/Xpa-/-^*^) hprt-1^). All Q-PCR experiments were repeated at least three times. In case of the DEHP experiment, we also used the glyceraldehyde-3-phosphate dehydrogenase as an additional housekeeping gene. P values (*) report the one-tail t-test probability that a gene has the same direction in the expression with that shown for the 15-day old *Csb^m/m^/Xpa^-/-^* mouse liver as compared to corresponding littermate controls. In addition, we also calculated the probability that all genes examined by Q-PCR demonstrate the same direction in the expression with those previously shown in the liver of *Csb^m/m^/Xpa^-/-^* mice by microarrays. Assuming these are independent events, the probability of each gene being up- or down regulated is 0.5 (1/2) whereas the probability of all independent events (13 genes examined) occurring in sequence is 0.00012 (0.5^13^).
